# Difluorinative Cyclopropene Rearrangement by I(I)/I(III) Catalysis: Regio‐ and Stereoselective Synthesis of Allyl Difluorides

**DOI:** 10.1002/anie.202518520

**Published:** 2025-10-11

**Authors:** Zi‐Xuan Wang, Nele A. Heckmann, Constantin G. Daniliuc, Ryan Gilmour

**Affiliations:** ^1^ University of Münster Institute for Organic Chemistry Corrensstraße 36 48149 Münster Germany

**Keywords:** Allylic compounds, Fluorination, Hypervalent iodine, Organocatalysis, Stereoselectivity

## Abstract

Allyl difluorides are pervasive in the pharmaceutical arena, but synthetic challenges in the construction of highly substituted derivatives impede chemical space exploration. Consequently, efforts to develop general approaches that display high levels of regio‐ and stereo‐selectivity continue to be intensively pursued. To contribute to this vibrant area of contemporary organofluorine chemistry, a highly efficient difluorinative rearrangement of densely substituted cyclopropenes is disclosed under the auspices of I(I)/I(III) catalysis. This platform leverages a highly intuitive ring opening model that enables di‐, tri‐, and tetra‐substituted allyl difluorides to be generated with high levels of stereoselectivity where the transient I(III) center serves as a traceless directing group. X‐ray crystal structural analysis is described together with facile post‐reaction modifications that include expedient access to fluorinated indenes. Given the ubiquity of the allyl difluoride chemotype in drug discovery, it is envisaged that this operationally simple, organocatalytic platform will expedite bioisostere design.

## Graphical abstract

A difluorinative rearrangement of substituted cyclopropenes is disclosed using I(I)/I(III) catalysis. This platform enables di‐, tri‐, and tetra‐substituted allyl difluorides to be generated with high levels of stereoselectivity, with the I(III) center serving as a traceless directing group. X‐ray crystal structural analysis is described together with facile post‐reaction modifications that include expedient access to fluorinated indenes.

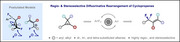

## Introduction

**Figure 1:**
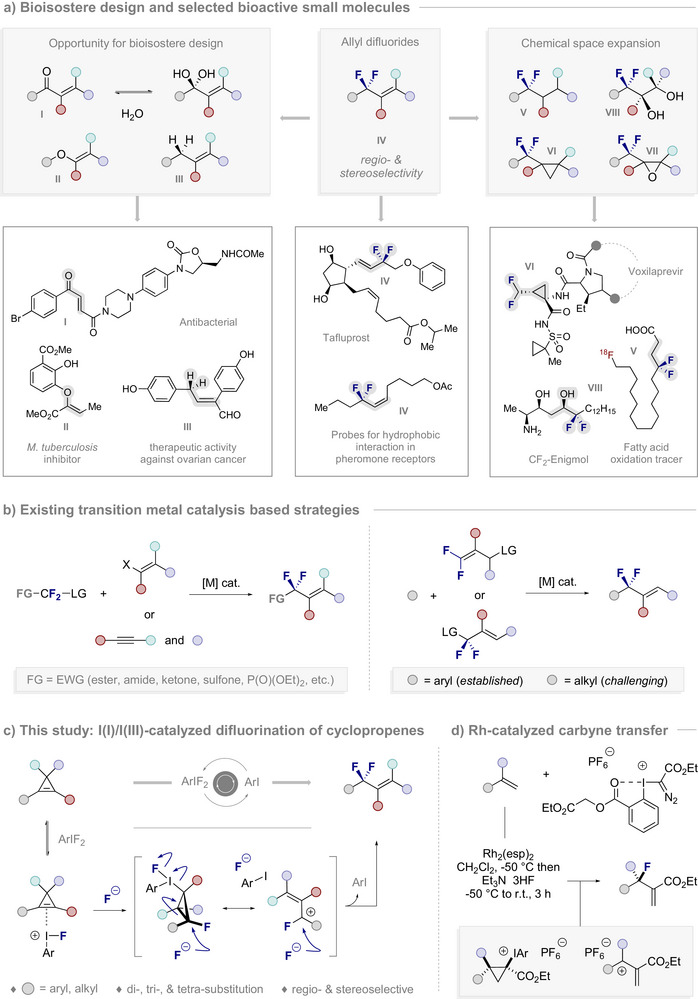
Motivation and conceptual framework for the study. a) Bioisostere design and selected examples of bioactive small molecules. b) Synthesis routes to allyl difluorides based on transition metal catalysis. c) Reaction blueprint to enable the regio‐ and stereoselective difluorinative rearrangement of cyclopropenes based on I(I)/I(III) catalysis. d) The Rh‐catalyzed carbyne transfer approach to allyl fluorides by Suero and co‐workers.

Molecular editing with fluorine is a core objective in contemporary synthetic research, and an endeavour that continues to be highly disruptive in the quest for innovative functional small molecules with societal relevance.^[^ ^1^, ^2^, ^3^, ^4^, ^5^, ^6^, ^7^ ^]^ Born out of necessity, the contemporary fluorination arsenal reflects an ever‐expanding reliance on fluorinated pharmaceuticals^[^ ^8^, ^9^, ^10^, ^11^, ^12^ ^]^ and agrochemicals,^[^ ^13^, ^14^, ^15^ ^]^ juxtaposed with the paucity of naturally occurring fluorinated building blocks to expedite synthesis.^[^ ^16^ ^]^ The field remains an exemplar of how chemical intuition has been brought to bear in the creation of small molecules with conformational and physicochemical profiles that can be predicated *a priori*,^[^ ^17^, ^18^ ^]^ and which have no biological blueprints. Of the plenum of innovative fluorinated motifs in existing chemical space, allyl difluorides have become ubiquitous in medicinal chemistry and chemical biology on account of the subtle changes associated with CH_2_ to CF_2_ isosteric replacement (Figure 1a).^[^ ^19^, ^20^, ^21^, ^22^ ^]^ Pertinent structural features include a expanded internal FCF angle (cf. HCH) on account of fluorine–fluorine electrostatic repulsion,^[^ ^23^, ^24^, ^25^, ^26^ ^]^ and well‐defined exit vectors that allow for subsequent derivatisation. In addition, CH_2_ to CF_2_ substitution is accompanied by localized partial charge inversion,^[^ ^27^ ^]^ rendering allyl difluorides powerful (bio)isosteres of a range of common chemotypes that include α,β‐unsaturated carbonyl compounds, their hydrates, enol ethers, and alkenes (Figure 1a **, left**).^[^ ^28^, ^29^ ^]^ Consequently, efforts to facilitate synthesis have been intensively pursued. Despite this, the regio‐ and stereo‐controlled synthesis of allyl difluorides remains challenging, with sterically congested systems proving to be particularly intractable.^[^ ^30^ ^]^ Of the plenum of existing methods to enable the synthesis of allyl difluoride motifs, coupling reactions of alkenes or alkynes with difluoromethyene (CF_2_)‐containing reagents are frequently leveraged. Predicated on C(sp^3^)–C(sp^2^) bond formation, these approaches are valuable entry points to generate fluorinated products with electron‐withdrawing groups attached to the fluorine‐bearing carbon (Figure 1b).^[^ ^31^, ^32^, ^33^, ^34^, ^35^, ^36^, ^37^ ^]^ Similarly, the generation of *gem*‐difluoroallylated arenes by *gem*‐difluoroallylation again allow carbon–carbon bond forming events to be harnessed.^[^ ^38^, ^39^, ^40^, ^41^, ^42^, ^43^ ^]^ However, the synthesis of the corresponding *gem*‐difluoroallylated alkanes remains challenging: the coupling of conventional reagents (e.g., 3‐bromo‐3,3‐difluoropropene) with aliphatic nucleophiles is complicated by formation of regioisomeric *gem*‐difluoroalkenes.^[^ ^44^, ^45^ ^]^ Innovations in reagent design have partially alleviate this challenge, but success has been limited to the generation of terminal alkenes products.^[^ ^46^, ^47^, ^48^ ^]^ To the best of our knowledge, only a single case in which the formation of a *gem*‐difluoroallylated alkane containing a di‐substituted internal alkene as a *Z*/*E* mixture has been reported using an alkyl‐substituted sulfonium salt.^[^ ^48^ ^]^ Importantly, sulfide‐catalyzed fluorinative Meyer–Schuster‐like rearrangements of propargylic fluorides and rhodium(III)‐catalyzed hydroarylation of α,α‐difluoromethylene alkynes have proven effective in the synthesis of tri‐substituted *gem*‐difluoroallylated alkanes.^[^ ^49^, ^50^ ^]^ Nevertheless, general platforms to expedite the synthesis of *gem*‐difluoroallylated arenes and alkanes with high stereoselectivity remains conspicuously underrepresented.

To augment the existing method portfolio, and enable the construction of highly substituted allyl difluorides, a conceptually orthogonal approach was envisaged in which the substituents would be pre‐installed in an easily accessible cyclopropene fragment (Figure 1c).

It was envisaged that a main group I(I)/I(III) catalysis fluorination platform^[^ ^51^, ^52^, ^53^, ^54^, ^55^, ^56^, ^57^, ^58^, ^59^, ^60^, ^61^, ^62^, ^63^, ^64^, ^65^, ^66^, ^67^, ^68^, ^69^, ^70^, ^71^, ^72^, ^73^, ^74^, ^75^, ^76^, ^77^, ^78^ ^]^ would be ideally suited to activate the π‐bond toward an initial nucleophilic fluorination through in situ generation of an ArIF_2_ species.^[^ ^79^, ^80^, ^81^ ^]^ The constraints imposed by the rigid, highly substituted cyclopropene would enforce a 1,2‐*anti* relationship of the fluorine and hypervalent iodine substituents following the initial addition. Moreover, the regio‐selectivity of the process, which could lead to two conceivable isomeric *anti*‐adducts, would be dictated by the disparity of the alkene positions and stabilizing interactions (e.g., ArI^+^F cation‐π)^[^ ^1^, ^2^ ^]^ imparted by the substituents at the C(sp^3^) position of the cyclopropene. Addition of the second fluoride nucleophile to the cyclopropyl intermediate (**I**) is anticipated to occur at the more electrophilic fluorine‐bearing carbon, thereby delivering the *geminal* difluoro motif and liberating the catalyst. Confidence in this approach via intermediate **I** stemmed from the seminal work by Fleming and Thomas on the stereochemical course of halocyclopropane ring‐opening.^[^ ^82^, ^83^, ^84^, ^85^, ^86^ ^]^ In addition to this asynchronous, concerted model, the leaving group ability of I(III) renders the involvement of an allyl cation intermediate (**II**) plausible. At this end of the mechanistic spectrum, the ability of fluorine to act as a cation stabilizing auxiliary^[^ ^87^ ^]^ enables the the regioselectivity of the second C(sp^3^)–F bond forming event to be rationalized. Importantly, Suero and co‐workers have reported an elegant Rh‐catalyzed carbyne transfer approach to allyl fluorides in which intermediates **III** and **IV** are implicated (Figure 1d).^[^ ^88^, ^89^ ^]^ This approach leverages a diazo‐containing iodonium reagent to process 1,1‐disubstituted alkenes to the branched products through the action of a Rh_2_(esp)_2._ Although caution must be exercised when drawing mechanistic comparisons on account of the very different conditions involved, the studies by Fleming and Thomas^[^ ^82^, ^83^, ^84^, ^85^, ^86^ ^]^ and Suero and colleagues^[^ ^88^, ^89^ ^]^ are instructive when contemplating the mechanistic continuum. Collectively, the blueprint shown in Figure 1c shifts stereoselective allyl difluoride synthesis away from (often fluorinated) linear precursors and enables key events to be regulated in a highly‐preorganized environment.

## Results and Discussion

**Figure 2:**
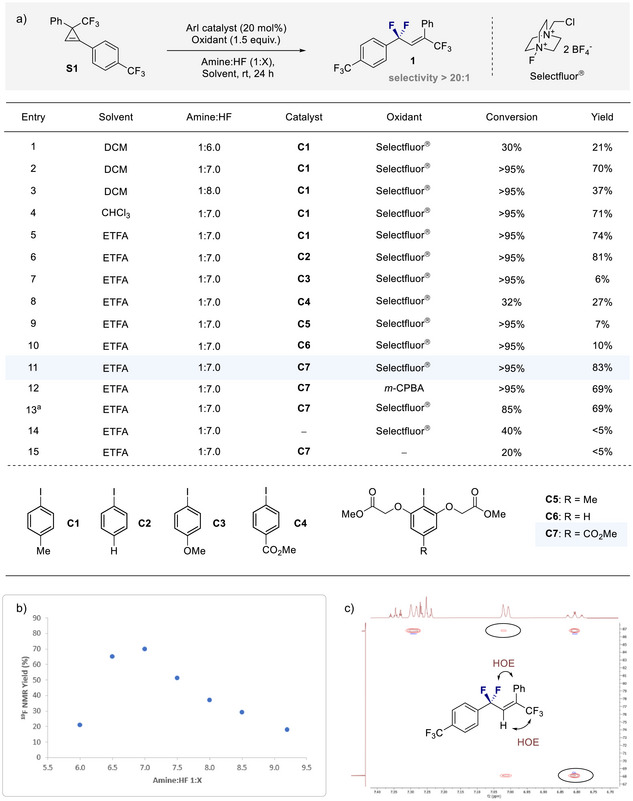
Reaction development. a) Optimization of reaction conditions. Standard reaction conditions: cyclopropene **S1** (0.10 mmol), catalyst (20 mol%), amine•HF (0.25 mL), solvent (0.25 mL), and oxidant (0.15 mmol). Ethyl fluoroacetate was used as internal standard. Conversions and yields were determined by ^19^F NMR. The stereoselectivity was >20:1 in all cases. b) Influence of the amine:HF ratio on the transformation. Standard reaction conditions: cyclopropene **S1** (0.10 mmol), **C1** (20 mol%), amine•HF (0.25 mL), DCM (0.25 mL), and Selectfluor (0.15 mmol). c) ^19^F‐^1^H HOE analysis of the product ***E*‐1**. ^a)^10 mol% of catalyst was used.

To explore the feasibility of an I(I)/I(III) catalysis platform, the trisubstituted cyclopropene **S1** was selected as a model substrate for reaction development. Initial reactions were performed with *p*‐TolI (20 mol%), Selectfluor (1.5 equiv.) as an oxidant, and methylene chloride (DCM) as the reaction medium. Variation of the amine:HF ratio (entries 1–3) revealed 1:7 to be optimal and this served as the choice for the reminder of the investigation (Figure 2). A solvent screen (entries 4 and 5) identified ethyl trifluoroacetate (ETFA) as being the most effective reaction medium for the title transformation. Comparison of aryl‐iodide organocatalysts **C1**–**C7** (entries 6–11) confirmed **C7** to be the most efficient catalyst, furnishing the desired allyl difluoride **1** in 83% yield. It is important to note that the reaction proved to be highly stereoselective (>20:1), and no competing *vicinal* difluorination was observed.^[^ ^69^, ^70^, ^71^, ^72^, ^73^, ^74^, ^75^, ^76^, ^77^, ^78^ ^]^ Replacing Selectfluor with *m*‐CPBA proved to be detrimental to efficiency (entry 12), and reducing the catalyst loading led to a slight decrease in yield (entry 13). Control experiments without the catalyst (entry 14) and the terminal oxidant (entry 15) inhibited the reaction, which support the involvement of an I(I)/I(III) catalysis cycle (Figure 2a).

To correlate the Brønsted acidity with reaction efficiency,^[^ ^71^, ^90^, ^91^ ^]^ the amine:HF ratio was plotted against yield as determined by ^19^F NMR spectroscopy.

The plateau observed with the 1:7 amine:HF ratio is evident from Figure 2b: this sensitivity is consistent with early observations on the activation of iodobenzene dichloride catalysed by trifluoroacetic acid.^[^ ^92^, ^93^, ^94^ ^]^ In addition to securing the configuration of product ***E*‐1** by HOE analysis (Figure 2c), it was possible to unequivocally establish the molecular connectivity by single crystal X‐ray diffraction analysis (*vide infra*).

**Figure 3:**
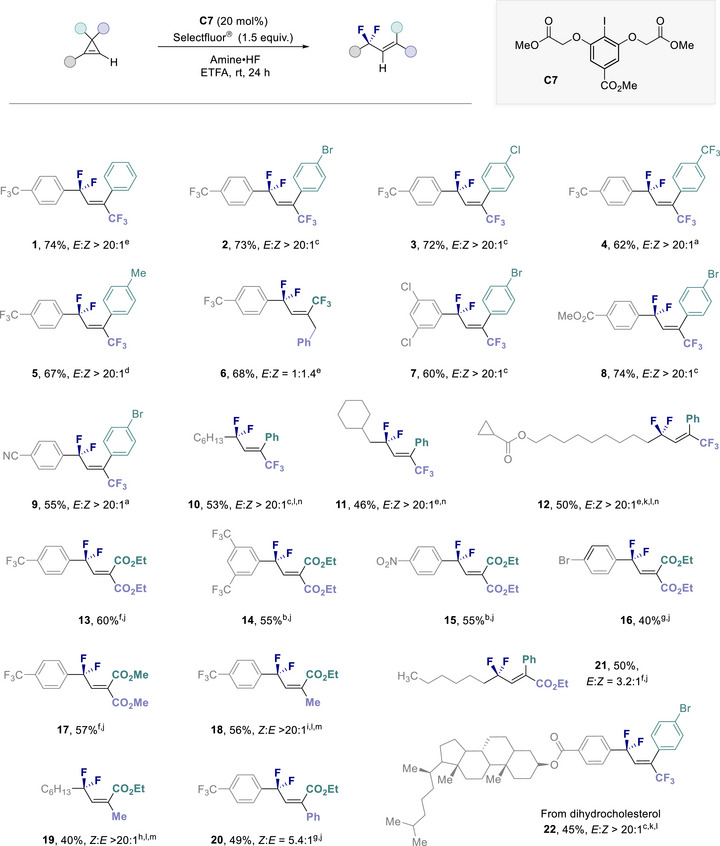
Scope for the stereoselective difluorination to access trisubstituted alkenes. Reaction conditions: cyclopropene (0.10 mmol), catalyst (20 mol%), amine•HF (0.25 mL), ETFA (0.25 mL), and Selectfluor (0.15 mmol). Isolated yields are given. The stereoselectivity was determined by ^19^F NMR from the crude reaction mixture. ^a)^Amine:HF = 1:9.2. ^b)^Amine:HF = 1:8.5. ^c)^Amine:HF = 1:8.0. ^d)^Amine:HF = 1:7.5. ^e)^Amine:HF = 1:7.0. ^f)^Amine:HF = 1:6.5. ^g)^Amine:HF = 1:5.5. ^h)^Amine:HF = 1:5.0.^i)^Amine:HF = 1:4.5. ^j)^Reaction performed on 0.20 mmol scale. ^k)^Reaction performed on 0.05 mmol scale. ^l)^DCM was used as solvent. ^m)^ **C1** was used as catalyst. ^n)^ **C2** was used as catalyst.

With optimized conditions for the difluorinative rearrangement of cyclopropenes having been identified, the scope and limitations of the transformation were explored (Figure 3). As previously noted, subtle variations in the amine:HF ratio were found to fine‐tune reaction performance in individual cases. Initially, the impact of modifying the C(sp^3^) position was investigated (Figure 3, highlighted in green).

The introduction of halogens, electron‐withdrawing groups and short alkyl fragments on the aryl ring proved unproblematic, and the target trisubstituted allylic difluorides were forged with high levels of stereoselectivity (**1**–**5**, >20:1). Replacing the aryl substituent with a benzyl group was also tolerated (**6**), albeit with a reduction in selectivity: this supports the notion that the aryl substituent biases alkene geometry, potentially through a stabilizing interaction in the initial *anti*‐addition step (e.g., ArI^+^F cation‐π).^[^ ^1^, ^2^ ^]^ Regrettably, efforts to replace the CF_3_ unit with small alkyl groups was unsuccessful. To further establish generality of substrate scope, subsequent modifications were performed at the alkene unit of the cyclopropene (shown in gray). To that end, a range of cyclopropenes with aryl substituents were initially subjected to the general catalysis conditions and this led to the formation of the chlorine derivative (**7**), ester (**8**) and nitrile (**9**). Simple alkyl and cycloalkyl derivatives were also compatible with the reaction conditions (**10** and **11**), and the transformation proved to be highly chemoselective for the cyclopropene motif: competing 1,3‐difluorination of the cyclopropane was not observed (**12**). Gratifyingly, the trifluoromethyl derivatives **13** and **14**, the nitro derivative **15** and bromide **16** were generated and which contain Michael acceptor motifs. Variation of the ester moiety (**17**) and replacement with a methyl group, to furnish **18** and **19** respectively, occurred in a highly stereoselective fashion (>20:1).

An interesting observation resulted from the replacement of the ester group with a phenyl substituent (**13** versus **20** and **21**). Interestingly, this led to the predominant formation of the *Z*‐isomer in the case of **20**. It is likely that the directing influence of the ester^[^ ^95^ ^]^ complicates with the model described in Figure 1. Interestingly, exploring the influence of aryl to alkyl exchange at the neighbouring position (in gray) results in preferential formation of the *E*‐isomer **21**. (N.B. In the starting cyclopropene, the only variation is the aryl / alkyl substitution **S20** and **S21** in the Supporting Information). Finally, the compatibility of the method with a dihydrocholesterol derivative was validated and enabled compound **22** to be generated stereoselectively (>20:1).

**Figure 4:**
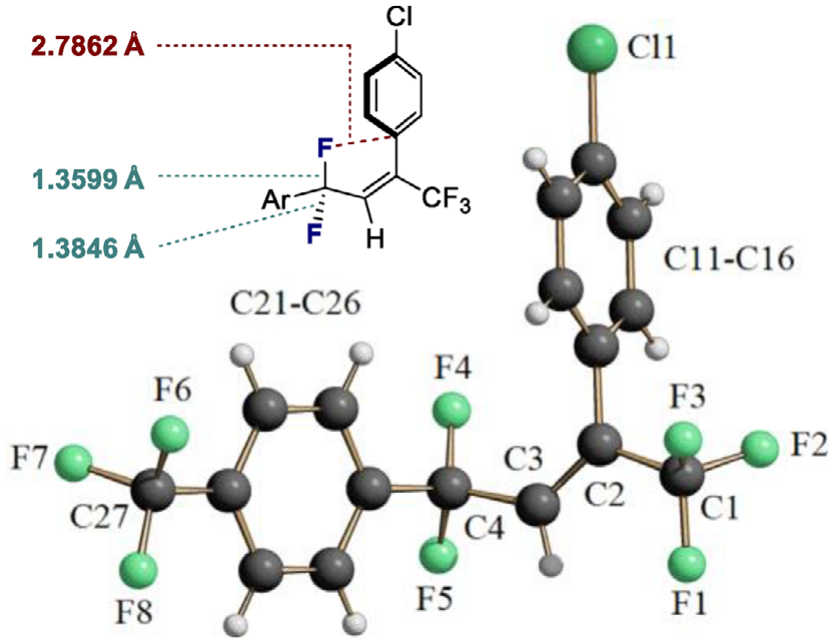
Crystal structure analysis of allylic difluoride **3**. Thermal ellipsoids are shown at 50% probability.

The allylic difluoride **3** was crystalline and it was possible to grow crystals that were suitable for X‐ray analysis (Figure 4).^[^ ^96^ ^]^ It is interesting to note that a small difference in bond lengths was observed between the two allylic C─F bonds (1.3599 Å versus 1.3846 Å): this can be rationalized by invoking a stabilizing hyperconjugative π→σ*_C─F_ interaction between the alkene π‐bond and the C(sp^3^)─F antibonding orbital which is coplanar.^[^ ^1^, ^97^ ^]^ A second intriguing feature of this analysis proved to be the short distance between one of the F atoms (F4) and the C(sp^2^) center of the neighbouring *ipso*‐carbon of the aryl ring. The distance F4^…^C11 is 2.7862 Å, which is less than the sum of the van der Waals distance (3.17 Å).^[^ ^98^, ^99^ ^]^ It is interesting to note that the F4 fluorine atom is slightly bent out from the plane of π system (torsion angle C2─C3─C4─F4 is 23.2°), thereby enabling this proximity to the *ipso*‐C atom (C11).

**Figure 5:**
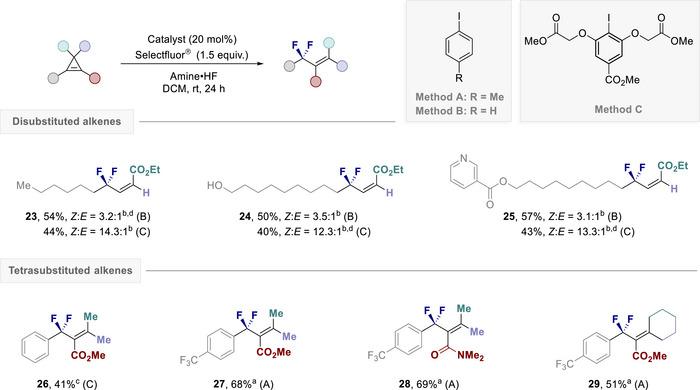
Scope for the stereoselective difluorination to access di‐ and tetra‐substituted alkenes. Reaction conditions: cyclopropene (0.10 mmol), catalyst (20 mol%), amine•HF (0.25 mL), DCM (0.25 mL), and Selectfluor (0.15 mmol). Isolated yields were given. The stereoselectivity was determined by ^19^F NMR from the crude reaction mixture. ^a)^Amine:HF = 1:6.0. ^b)^Amine:HF = 1:5.5. ^c)^Amine:HF = 1:4.5. ^d)19^F NMR yield using ethyl fluoroacetate as internal standard.

Having established a stereoselective difluorination platform that enables the operationally simple generation of trisubstituted alkenes, attention was then turned to expanding the scope of the process to encompass di‐, and tetrasubstitution (Figure 5). This was possible but required a reassessment of the reaction conditions, such that dichloromethane was used as the solvent instead of ethyl trifluoroacetate and a short process of catalyst refinement was conducted. For the construction of disubstituted alkenes, a general trend was noted: whilst catalyst PhI (**C2**) is a highly competent catalyst, the resorcinol derivative (**C7**) proved to be more stereoselective. Exposing simple alkyl derivatives to the catalysis conditions delivered the expected disubstituted alkene **23** with good level of stereoselectivity (up to 14:1 *Z*:*E*). Gratifyingly, unprotected alcohols (**24**) and medicinally relevant pyridines (**25**) were also compatible with the reaction conditions.

For the construction of the more challenging tetrasubstituted alkenes, the revised conditions once again enabled the target allyl difluoride **26** to be generated. The positive impact of the electron‐withdrawing aryl substituent was fully leveraged by installing a trifluoromethyl handle, thereby facilitating access to **27**. Amide groups were tolerated (**28**). Notably, this approach also enabled the cyclic alkene **29** to be prepared with synthetically useful levels of efficiency. Collectively, examples **23**–**25** illustrate the value of this approach in accessing rare, *Z*‐configured products (up to *Z*:*E* 14:1), whereas compounds **26**–**29** illustrate the insensitivity of the method to sterically demanding substrates (up to 69% yield).

**Figure 6:**
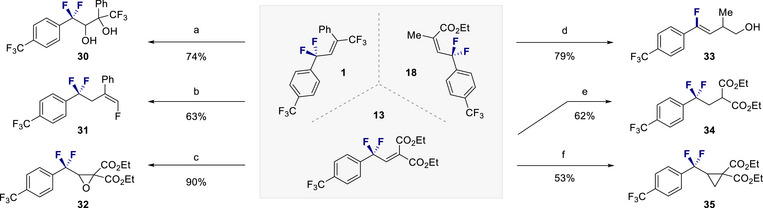
Product derivatisation. Conditions: a) OsO_4_, NMO, THF, H_2_O, 50 °C−65 °C. b) LiAlH_4_, THF, rt. c) *m*‐CPBA, KOH, DCM, rt. d) DIBAL‐H, THF, ‐78 °C ‐ rt. e) Pd/C, H_2_, MeOH, rt. f) trimethylsulfoxonium iodide, NaH, DMSO, rt.

To further demonstrate the synthetic utility of this difluorinative rearrangement of cyclopropenes, a series of product derivatization reactions were conducted (Figure 6). Initially, alkene **1** was converted to the *vicinal* diol **30** through treatment with OsO_4_ / NMO. Under hydrodefluorination conditions, the trifluoromethylated alkene **1** could be smoothly processed to the terminal monofluoroalkene **31**.^[^ ^100^ ^]^ Epoxidation of alkene **13** proved facile in the presence of *m*‐CPBA, furnishing **32** in 90% yield.^[^ ^101^ ^]^ Moreover, the homoallyl alcohol **33** was generated via reduction of **18** and subsequent hydrodefluorination of the intermediate allyl alcohol. Hydrogenation of alkene **13** enabled alkane **34** to be prepared, and cyclopropanation of alkene **13** allowed the α,α‐difluorocyclopropane **35** to be synthesized.

**Figure 7:**
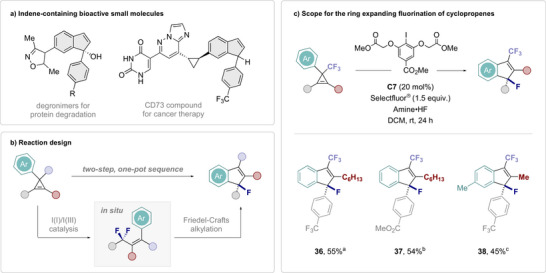
Ring expanding fluorination of cyclopropenes. a) Selected examples of bioactive small molecules containing the indene unit. b) Integrating an I(I)/I(III) catalysis event in a one‐pot approach for the synthesis of fluorinated indenes from cyclopropenes. c) Scope of the title transformation. Standard reaction conditions: cyclopropene (0.10 mmol), **C7** (20 mol%), amine•HF (0.25 mL), DCM (0.25 mL), and Selectfluor (0.15 mmol). Isolated yields were given. ^a)^Amine:HF = 1:9.2. ^b)^Amine:HF = 1:8.5. ^c)^Amine:HF = 1:7.0.

Leveraging the intrinsic acidity of (HF‐based) catalysis conditions in multi‐step processes has attracted attention in recent years.^[^ ^66^, ^102^ ^]^ Motivated by the prominence of indenes in pharmaceuticals (Figure 7a),^[^ ^103^, ^104^ ^]^ it was envisaged that a one‐pot sequence could provide enabling access to fluorinated isosteres: this approach would combine the title transformation with a Brønsted acid activation event to enable facile conversion of cyclopropenes to fluorinated indenes (Figure 7b). Specifically, the transformation would be reliant on the in situ generation of an allyl difluoride under the auspices of I(I)/I(III) catalysis. Inspired by the work of Paquin^[^ ^105^ ^]^ and previous work from this laboratory^[^ ^66^ ^]^ on the activation of benzylic C─F bonds, it was conceived that a Friedel–Crafts‐type cyclization would liberate a fluorinated indene under the Brønsted acidic reaction conditions. To validate the working hypothesis, fully substituted cyclopropene **S36** was prepared and exposed to catalysis condition using aryl iodide **C7**, Selectfluor and HF source. Gratifyingly, this enabled the desired fluorinated indene **36** to be generated in 55% yield (Figure 7c). Variations of the allyl and alkenyl substituents were also tolerated (**37** and **38**), providing further validation of the synthetic versatility of products generated by this I(I)/I(III)‐catalysis‐based difluorinative rearrangement of cyclopropenes.

## Conclusions

Motivated by the prominence and versatility of allyl difluorides, and the conspicuous dearth of general methods to facilitate their construction, a highly regio‐ and stereoselective difluorinative rearrangement of cyclopropenes has been developed leveraging a metal‐free (I)/I(III) catalysis platform. Based on a stereochemical model that exploits the transient iodine (III) substituent as a traceless steering element, the method enables the formation of di‐, tri‐, and tetrasubstituted *gem*‐difluoroallylated arenes and alkanes with broad functional group tolerance. High levels of stereocontrol are observed (up to 20:1), and X‐ray analysis reveals the subtle impact of the π‐system on proximal C─F bond lengths. The synthetic utility of the transformation is showcased through facile product derivatization. Moreover, given the importance of indenes in bioactive small molecules, integrating this process in a reaction cascade is validated, thereby enabling the synthesis of fluorinated indenes from cyclopropenes in a single operation. It is envisaged that this platform to enable the stereocontrolled synthesis of heavily substituted allyl difluorides will expedite organofluorine chemical space exploration.^[^ ^106^ ^]^

## Conflict of Interests

The authors declare no conflict of interest.

## Supporting information



Supporting Information

## Data Availability

The data that support the findings of this study are available from the corresponding author upon reasonable request.
