# Supplementary material for: Difluorinative Cyclopropene Rearrangement by I(I)/I(III) Catalysis: Regio‐ and Stereoselective Synthesis of Allyl Difluorides
Source: Angew Chem Int Ed Engl. 2025 Oct 11;64(50):e202518520. doi: 10.1002/anie.202518520 (PMC12684307; doi:10.1002/anie.202518520)
Supplement: Supplementary file 1 — Supporting Information [file ANIE-64-e202518520-s001.pdf]

Supplementary Information

Difluorinative Cyclopropene Rearrangement by I(I)/I(III) Catalysis: Regio- and Stereoselective Synthesis of Allyl Difluorides

Zi-Xuan Wang, Nele A. Heckmann, Constantin G. Daniliuc, Ryan Gilmour*

Institute for Organic Chemistry, University of Münster, Corrensstraße 36, 48149 Münster (Germany)

*E-mail: ryan.gilmour@uni-muenster.de

Table of Contents

1. Supplementary Methods 3
1.1 General information 3
1.2 Preparation and characterisation of starting materials 5
1.3 Preparation and characterisation of catalysts 38
1.4 Preparation and characterisation of allyl difluorides 38
1.5 Product derivatisation 66
1.6 Ring expanding fluorination of cyclopropenes 72
1.7 X-ray crystallographic data 79
1.8 NMR-Spectra of key compounds 81
2. Supplementary References 214

1. Supplementary Methods

1.1 General information

All commercially available reagents were purchased as reagent grade from *Sigma Aldrich*, *Merck*, *Alfa Aesar*, *TCI*, *Fluorochem* or *abcr* and were used without further purification unless otherwise stated. Solvents for extractions or chromatographic purifications were bought as technical grade and distilled on a rotary evaporator prior to use. All reactions with HF were run in Teflon® vials. For analytical thin layer chromatography, glass plates coated with SiO_2-60 F254 were used from *Merck*. They were visualized with UV-light (254 nm) or with $KMnO_4$ or CAM solution. Column chromatography was performed using silica gel (40-63 µm, *VWR Chemicals*). For preparative thin layer chromatography, glass plates coated with SiO_2-60 F254 and 2 mm thickness were used from *Merck.* The obtained products are often volatile and care must be taken in the isolation. The NMR measurements were performed on a *Bruker AV300*, *AV400*, *Agilent DD2 500* or an *Agilent DD2 600* by the NMR service department of the Organisch-Chemisches Institut, University of Münster. The chemical shifts were referenced to the residual solvent peak as the internal standard (7.26 ppm for $CDCl_3$, 2.50 ppm for DMSO-d_6 for ^{1}H-NMR, 77.16 ppm for $CDCl_3$, 39.52 ppm for DMSO-d_6 for ^{13}C-NMR). The multiplicity is abbreviated as follows: s (singlet), d (doublet), t (triplet), q (quartet), p (pentet), h (heptet), m (multiplet) and br (broad). The given assignments are supported by additional 1D and 2D NMR experiments. The melting points were determined on a *Büchi* B-545 melting point apparatus with open glass capillaries. The IR measurements were performed on a *Perkin-Elmer 100 FT-IR* spectrometer and the intensities of the bands are assigned as follows: w (weak), m (medium), s (strong). High resolution mass spectrometry was performed by the MS service of the Organisch-Chemisches Institut, University of Münster on a Bruker Daltonics MicroTof (HRMS-ESI), a Triplequad TSQ 7000 (MS-EI), Triplequad Quattro Micro GC (GC-EI-MS), a Qp5050 Single Quad (GC-EI-MS) or a LTQ Orbitap LTQ XL (HRMS-APCI).

Preparation of various amine·HF mixtures

Amine·HF sources:

$NEt_3 \cdot 3HF$; Supplier: abcr; (MW: 161.21g/mol, ρ = 0.990 g/mL)

Olah's Reagent (70wt% HF: Py·9.23HF); Supplier: Sigma Aldrich; (MW: 263.79 g/mol, ρ = 1.1 g/mL)

Procedure for calculating compositions of amine·HF mixtures:

The amine·HF mixtures based on $NEt_3 \cdot 3HF$ and Olah´s reagent were prepared following the procedure previously described by this group.[1]

1.2 Preparation and characterisation of starting materials

General Procedure A

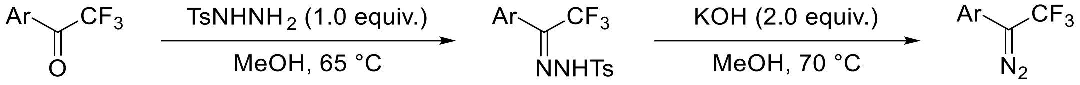

According to a modified literature procedure,[2] $TsNHNH_2$ (1.0 equiv.) was stirred in MeOH (1.5 M) at reflux until complete dissolution. Subsequently the reaction was cooled to rt and trifluoroacetophenone (1.0 equiv.) was added in one portion under Ar atmosphere. The resulting reaction mixture was then stirred at 65 °C for 12 h, after which it was cooled to -20 °C, leading to the precipitation of the desired products (precipitation can be induced by addition of pentane). The precipitate was collected by vacuum filtration and washed with pentane.

Tosyl hydrazone (1.0 equiv.) was added to a solution of KOH (2.0 equiv.) in MeOH (0.4 M) and the resulting suspension was heated to reflux for 1-2 h under Ar atmosphere. The reaction was cooled to room temperature and diluted with distilled water. It was then extracted three times with DCM and washed with a saturated aqueous solution of $NaHCO_3$ and brine. The combined organic layers were dried over anhydrous Na_2SO_4, filtered, and concentrated under reduced pressure. The crude product was purified by column chromatography (SiO_2, specified combination of solvents).

General Procedure B

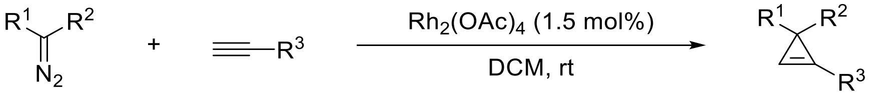

According to a modified literature procedure,[2] the diazo compound (1.0 equiv.) was dissolved in DCM (~ 0.6 M) and the resulting solution was added via syringe pump to a suspension of $Rh_2(OAc)_4$ (1.5 mol%) and the corresponding alkyne (2.5 equiv.) in DCM (~ 1.0 M) at room temperature under Ar atmosphere. After the addition was complete, the reaction mixture was allowed to stir for another 10 h. The reaction mixture was then filtered through a small pad of silica eluting with DCM and the filtrate was concentrated under reduced pressure. The crude residue was purified by column chromatography (SiO_2, specified combination of solvents).

General Procedure C

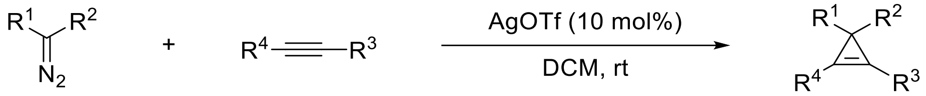

According to a modified literature procedure,[3] a mixture of alkyne (2.0 equiv.) and AgOTf (10 mol%) was added to a three-necked flask covered with aluminum foil to exclude light. The mixture was dissolved with DCM (~ 1.0 M) and stirred at room temperature under Ar atmosphere. The diazo compound (1.0 equiv.) in DCM (~ 0.2 M) was then added to former solution via syringe pump. After addition, the mixture was stirred for additional 10 h then concentrated in vacuo. The crude residue was purified by column chromatography (SiO_2, specified combination of solvents).

General Procedure D

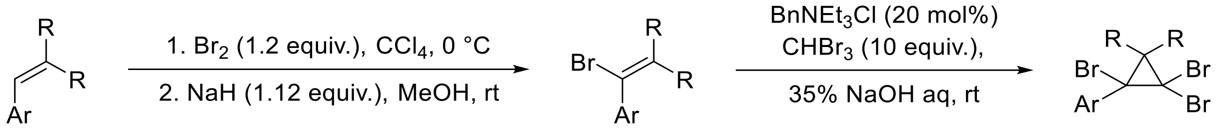

According to a modified literature procedure,[4] to a stirred solution of alkene (1.0 equiv.) in CCl_4 (0.5 M) was added bromine (1.2 equiv.) at 0 °C under Ar atmosphere, and stirring was continued for 4 h at 0 °C. To the reaction mixture was added 10% aqueous $Na_2S_2O_3$ and aqueous saturated $NaHCO_3$ successively. The mixture was extracted with DCM three times, and the combined organic layers were washed with brine, dried over anhydrous Na_2SO_4, filtered, and concentrated under reduced pressure. The crude residue was passed through a silica plug to afford the corresponding 1,2-dibromide (specified combination of solvents).

To a solution of 1,2-dibromide (1.0 equiv.) in MeOH (0.3 M) was added sodium hydride (1.12 equiv.) slowly at 0 °C under Ar atmosphere, and the resulting mixture was stirred at rt overnight. After adding water, the mixture was extracted with Et_2O three times. The combined organic layers were washed with brine, dried over anhydrous Na_2SO_4, filtered, and concentrated under reduced pressure. The crude residue was passed through a silica plug to afford the corresponding bromoalkene (specified combination of solvents).

A mixture of bromoalkene (1.0 equiv.), bromoform (10 equiv.), 35% NaOH aqueous solution (0.5 M) and benzyltriethylammonium chloride (20 mol%) was stirred at rt under Ar atmosphere for 40 h. The mixture was diluted with water and extracted with DCM three times. The combined organic layers were dried over anhydrous Na_2SO_4, filtered, and concentrated under reduced pressure. The

crude residue was purified by column chromatography (SiO_2, specified combination of solvents).

General Procedure E

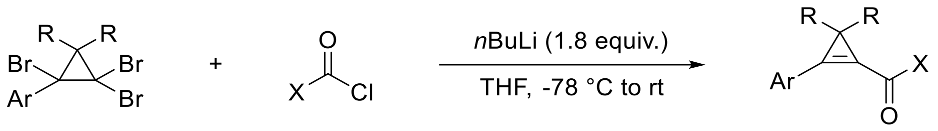

According to a modified literature procedure,[4] to a solution of tribromocyclopropane (1.0 equiv.) in THF (0.2 M) at -78 °C, was added dropwise a solution of *n*BuLi (1.8 equiv.). The reaction mixture was stirred at -78 °C and after 1.5 h, chloroformate or carbamoyl chloride (3.0 equiv.) was added. The reaction mixture was warmed to rt, stirred for 1 h and then quenched by the addition of a saturated aqueous solution of NH_4Cl. The layers were separated and the aqueous phase was extracted three times with Et_2O. The combined organic layers were washed with brine, dried over anhydrous Na_2SO_4, filtered and concentrated under reduced pressure. The crude residue was purified by column chromatography (SiO_2, specified combination of solvents).

(1-Diazo-2,2,2-trifluoroethyl)benzene (Int 1)

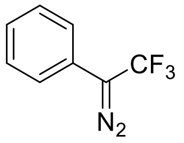

According to General Procedure **A**, the appropriate tosyl hydrazone was first prepared using 2,2,2-trifluoro-1-phenylethan-1-one (6.96 g, 40.00 mmol, 1.0 eq.). Then, the title compound **Int 1** was synthesized using the corresponding tosyl hydrazone (4.11 g, 12.00 mmol, 1.0 eq.). The crude residue was purified by column chromatography (*n*-pentane) to yield the title compound as a red oil (0.78 g, 4.17 mmol, 35%).

$\mathbf{R}_f$ = 0.85 (*n*-pentane).

^{1}H NMR (400 MHz, $CDCl_3$) δ 7.45 – 7.36 (m, 2H), 7.23 – 7.16 (m, 1H), 7.10 (d, *J* = 8.5 Hz, 2H).

^{19}F{^{1}H} NMR (377 MHz, $CDCl_3$) δ -57.36 (s, 3F).

ESI-MS: (*m/z*) requires: $[(C_8H_4N_2F_3)^-]$ = 185.0332, (*m/z*) found: $[(C_8H_4N_2F_3)^-]$ = 185.0302.

Analytical data is in agreement with literature values.[5]

1-(3-Phenyl-3-(trifluoromethyl)cycloprop-1-en-1-yl)-4-(trifluoromethyl)benzene (S1)

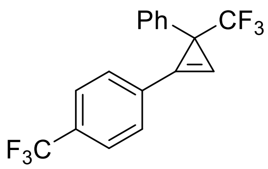

Compound **S1** was prepared according to General Procedure **B** using (1-diazo-2,2,2-trifluoroethyl)benzene (**Int 1**) (700.0 mg, 3.76 mmol, 1.0 eq.). The crude residue was purified by column chromatography (*n*-pentane) to yield the title compound as a white solid (702.2 mg, 2.14 mmol, 57%).

$\mathbf{R}_f$ = 0.50 (*n*-pentane).

^{1}**H NMR** (400 MHz, $CDCl_3$) δ 7.76 (d, *J* = 8.2 Hz, 2H), 7.71 (d, *J* = 8.2 Hz, 2H), 7.40 (d, *J* = 7.8 Hz, 2H), 7.37 – 7.27 (m, 4H).

^{19}**F{^{1}H} NMR** (377 MHz, $CDCl_3$) δ -62.97 (s, 3F), -64.11 (s, 3F).

GC-EI-MS: (*m/z*) requires: [$(C_{17}H_{10}F_6)^+$] = 328.0681, (*m/z*) found: [$(C_{17}H_{10}F_6)^+$] = 328.0677.

Analytical data is in agreement with literature values.[6]

1-Bromo-4-(1-diazo-2,2,2-trifluoroethyl)benzene (Int 2)

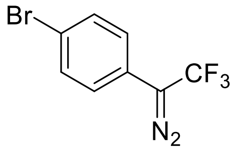

According to General Procedure **A**, the appropriate tosyl hydrazone was first prepared using 1-(4-bromophenyl)-2,2,2-trifluoroethan-1-one (10.12 g, 40.00 mmol, 1.0 eq.). Then, the title compound **Int 2** was synthesized using the corresponding tosyl hydrazone (5.05 g, 12.00 mmol, 1.0 eq.). The crude residue was purified by column chromatography (*n*-pentane) to yield the title compound as a red oil (1.04 g, 3.93 mmol, 33%).

$\mathbf{R}_f$ = 0.85 (*n*-pentane).

^{1}**H NMR** (400 MHz, $CDCl_3$) δ 7.60 – 7.46 (m, 2H), 6.96 (d, *J* = 8.3 Hz, 2H).

^{19}**F{^{1}H} NMR** (377 MHz, $CDCl_3$) δ -57.42 (s, 3F).

ESI-MS: (*m/z*) requires: [$(C_8H_5N_2BrF_3)^-$] = 264.9594, (*m/z*) found: [$(C_8H_5N_2BrF_3)^-$] = 264.9592.
Analytical data is in agreement with literature values.[5]

1-Bromo-4-(1-(trifluoromethyl)-2-(4-(trifluoromethyl)phenyl)cycloprop-2-en-1-yl)benzene (S2)

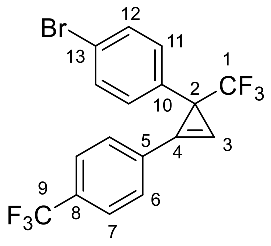

Compound **S2** was prepared according to General Procedure **B** using 1-bromo-4-(1-diazo-2,2,2-trifluoroethyl)benzene (**Int 2**) (530.1 mg, 2.00 mmol, 1.0 eq.). The crude residue was purified by column chromatography (*n*-pentane) to yield the title compound as a white solid (529.6 mg, 1.30 mmol, 65%).

$\mathbf{R}_f$ = 0.35 (*n*-pentane).

^{1}H NMR (400 MHz, $CDCl_3$) δ 7.73 (m, 4H, H-C6~C7), 7.45 (d, $^3J_{HH}$ = 8.6 Hz, 2H, H-C12), 7.33 (q, $^4J_{HF}$ = 1.5 Hz, 1H, H-C3), 7.30 – 7.23 (m, 2H, H-C11).

^{13}C NMR (126 MHz, $CDCl_3$) δ 136.67 (C13), 132.45 (q, $^2J_{CF}$ = 32.9 Hz, C8), 131.85 (C12), 130.31 (C6), 129.39 (q, $^4J_{CF}$ = 1.6 Hz, C11), 128.24 (C5), 126.26 (q, $^1J_{CF}$ = 277.7 Hz, C1), 126.26 (q, $^3J_{CF}$ = 3.7 Hz, C7), 123.77 (q, $^1J_{CF}$ = 272.7 Hz, C9), 121.76 (C10), 116.55 (q, $^3J_{CF}$ = 1.9 Hz, C4), 101.72 (q, $^3J_{CF}$ = 2.8 Hz, C3), 32.30 (q, $^2J_{CF}$ = 36.2 Hz, C2).

^{19}F NMR (470 MHz, $CDCl_3$) δ -63.04 (s, 3F, F-C9), -64.19 (s, 3F, F-C1).

^{19}F{^{1}H} NMR (377 MHz, $CDCl_3$) δ -63.04 (s, 3F, F-C9), -64.19 (s, 3F, F-C1).

GC-EI-MS: (*m/z*) requires: [$(C_{17}H_9BrF_6)^+$] = 405.9786, (*m/z*) found: [$(C_{17}H_9BrF_6)^+$] = 405.9783.

FT-IR ($\tilde{\nu}$ = cm^{-1}): 1492 (w), 1412 (w), 1320 (s), 1228 (w), 1165 (s), 1108 (s), 1062 (s), 1010 (m), 970 (w), 918 (m), 844 (m), 752 (w), 729 (m), 712 (s), 580 (w).

Melting Point: 55-56 ˚C.

1-Chloro-4-(1-(trifluoromethyl)-2-(4-(trifluoromethyl)phenyl)cycloprop-2-en-1-yl)benzene (S3)

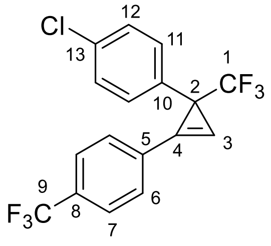

1-Chloro-4-(1-diazo-2,2,2-trifluoroethyl)benzene was first synthesized according to General Procedure **A** using 1-(4-chlorophenyl)-2,2,2-trifluoroethan-1-one (4.17 g, 20.00 mmol, 1.0 eq.). Compound **S3** was prepared according to General Procedure **B** using 1-chloro-4-(1-diazo-2,2,2-trifluoroethyl)benzene (371.7 mg, 1.69 mmol, 1.0 eq.). The crude residue was purified by column chromatography (*n*-pentane) to yield the title compound as a white

solid (239.8 mg, 0.66 mmol, 39%).

R$_f$ = 0.35 (*n*-pentane).

^{1}H NMR (400 MHz, $CDCl_3$) δ 7.79 – 7.67 (m, 4H, H-C6~C7), 7.36 – 7.27 (m, 5H, H-C3, H-C11~C12).

^{13}C NMR (126 MHz, $CDCl_3$) δ 136.15 (C13), 133.67 (C10), 132.44 (q, $^2J_{CF}$ = 32.9 Hz, C8), 130.31 (C6), 129.08 (q, $^4J_{CF}$ = 1.5 Hz, C11), 128.89 (C12), 128.28 (C5), 126.31 (q, $^1J_{CF}$ = 277.6 Hz, C1), 126.25 (q, $^3J_{CF}$ = 3.8 Hz, C7), 123.78 (q, $^1J_{CF}$ = 272.6 Hz, C9), 116.65 (C4), 101.83 (q, $^3J_{CF}$ = 2.9 Hz, C3), 32.23 (q, $^2J_{CF}$ = 36.0 Hz, C2).

^{19}F NMR (470 MHz, $CDCl_3$) δ -63.04 (s, 3F, F-C9), -64.21 – -64.26 (m, 3F, F-C1).

^{19}F{^{1}H} NMR (377 MHz, $CDCl_3$) δ -63.04 (s, 3F, F-C9), -64.23 (s, 3F, F-C1).

GC-EI-MS: (*m/z*) requires: [$(C_{17}H_9ClF_6)^+$] = 362.0292, (*m/z*) found: [$(C_{17}H_9ClF_6)^+$] = 362.0289.

FT-IR ($\tilde{\nu}$ = cm^{-1}): 1492 (w), 1412 (w), 1320 (s), 1303 (m), 1246 (w), 1165 (s), 1119 (s), 1062 (s), 1016 (m), 976 (w), 918 (m), 844 (m), 752 (m), 729 (m), 712 (m), 585 (w), 522 (w).

Melting Point: 33-35 ˚C.

1-(1-Diazo-2,2,2-trifluoroethyl)-4-(trifluoromethyl)benzene (Int 3)

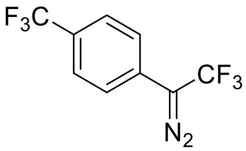

According to General Procedure **A**, the appropriate tosyl hydrazone was first prepared using 2,2,2-trifluoro-1-(4-(trifluoromethyl)phenyl)ethan-1-one (4.84 g, 20.00 mmol, 1.0 eq.). The solvent was changed to toluene and the reaction was performed at 90 °C. Then, the title compound **Int 3** was synthesized using the corresponding tosyl hydrazone (2.46 g, 6.00 mmol, 1.0 eq.). The crude residue was purified by column chromatography (*n*-pentane) to yield the title compound as an orange oil (0.76 g, 2.98 mmol, 50%).

R$_f$ = 0.85 (*n*-pentane).

^{1}H NMR (400 MHz, $CDCl_3$) δ 7.64 (d, *J* = 8.3 Hz, 2H), 7.18 (d, *J* = 8.3 Hz, 2H).

^{19}F{^{1}H} NMR (377 MHz, $CDCl_3$) δ -57.40 (s, 3F), -62.57 (s, 3F).

Analysis by mass spectrometry was inconclusive.

Analytical data is in agreement with literature values.[7]

4,4'-(1-(Trifluoromethyl)cycloprop-2-ene-1,2-diyl)bis((trifluoromethyl)benzene) (S4)

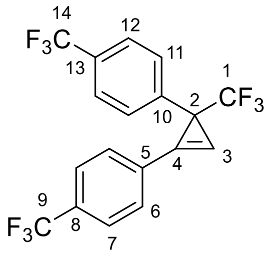

Compound **S4** was prepared according to General Procedure **B** using 1-(1-diazo-2,2,2-trifluoroethyl)-4-(trifluoromethyl)benzene (**Int 3**) (508.3 mg, 2.00 mmol, 1.0 eq.). The crude residue was purified by column chromatography (*n*-pentane) to yield the title compound as a colorless oil (650.9 mg, 1.64 mmol, 82%).

R*f* = 0.40 (*n*-pentane).

^{1}H NMR (400 MHz, $CDCl_3$) δ 7.74 (m, 4H, H-C6~C7), 7.58 (d, $^3J_{HH}$ = 8.4 Hz, 2H, H-C12), 7.51 (d, $^3J_{HH}$ = 8.2 Hz, 2H, H-C11), 7.34 (q, $^4J_{HF}$ = 1.6 Hz, 1H, H-C3).

^{13}C NMR (151 MHz, $CDCl_3$) δ 141.66 (C10), 132.62 (q, $^2J_{CF}$ = 33.1 Hz, C8), 130.39 (C6), 129.88 (q, $^2J_{CF}$ = 32.5 Hz, C13), 127.99 (C5), 127.94 (q, $^4J_{CF}$ = 1.7 Hz, C11), 126.32 (q, $^3J_{CF}$ = 3.8 Hz, C7), 126.20 (q, $^1J_{CF}$ = 277.3 Hz, C1), 125.68 (q, $^3J_{CF}$ = 3.8 Hz, C12), 124.13 (q, $^1J_{CF}$ = 272.6 Hz, C14), 123.74 (q, $^1J_{CF}$ = 272.6 Hz, C9), 116.08 (C4), 101.29 (q, $^3J_{CF}$ = 2.9 Hz, C3), 32.54 (q, $^2J_{CF}$ = 36.2 Hz, C2).

^{19}F NMR (564 MHz, $CDCl_3$) δ -62.73 (t, $^4J_{HF}$ = 1.1 Hz, 3F, CF_3), -63.09 (s, 3F, CF_3), -63.97 (dt, $^4J_{HF}$ = 1.5, $^5J_{HF}$ = 0.8 Hz, 3F, F-C1).

^{19}F{^{1}H} NMR (377 MHz, $CDCl_3$) δ -62.73 (s, 3F, CF_3), -63.09 (s, 3F, CF_3), -63.97 (s, 3F, F-C1).

GC-EI-MS: (*m/z*) requires: [$(C_{18}H_9F_9)^+$] = 396.0555, (*m/z*) found: [$(C_{18}H_9F_9)^+$] = 396.0553.

FT-IR ($\tilde{\nu}$ = cm^{-1}): 1619 (w), 1412 (w), 1320 (s), 1251 (w), 1165 (s), 1108 (s), 1062 (s), 1016 (m), 976 (w), 924 (m), 844 (s), 752 (m), 712 (m), 672 (w), 603 (m), 517 (w).

1-(1-Diazo-2,2,2-trifluoroethyl)-4-methylbenzene (Int 4)

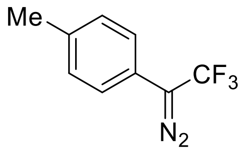

According to General Procedure **A**, the appropriate tosyl hydrazone was first prepared using 2,2,2-trifluoro-1-(p-tolyl)ethan-1-one (3.76 g, 20.00 mmol, 1.0 eq.). Then, the title compound **Int 4** was synthesized using the corresponding tosyl hydrazone (1.78 g, 5.00 mmol, 1.0 eq.). The crude residue was purified by column chromatography (*n*-pentane) to yield the title compound as a red oil (0.25 g, 1.23 mmol, 25%).

R*f* = 0.85 (*n*-pentane).

^{1}**H NMR** (400 MHz, $CDCl_3$) δ 7.21 (d, *J* = 8.0 Hz, 2H), 6.99 (d, *J* = 8.3 Hz, 2H), 2.35 (s, 3H).

^{19}**F{^{1}H} NMR** (377 MHz, $CDCl_3$) δ -57.40 (s, 3F).

Analysis by mass spectrometry was inconclusive.
Analytical data is in agreement with literature values.[5]

1-Methyl-4-(1-(trifluoromethyl)-2-(4-(trifluoromethyl)phenyl)cycloprop-2-en-1-yl)benzene (S5)

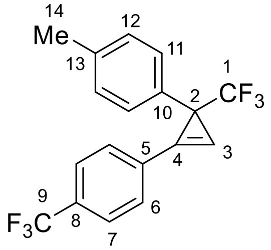

Compound **S5** was prepared according to General Procedure **B** using 1-(1-diazo-2,2,2-trifluoroethyl)-4-methylbenzene (**Int 4**) (270.3 mg, 1.35 mmol, 1.0 eq.). The crude residue was purified by column chromatography (*n*-pentane) to yield the title compound as a white wax (113.1 mg, 0.33 mmol, 24%).

$\mathbf{R}_f$ = 0.25 (*n*-pentane).

^{1}**H NMR** (400 MHz, $CDCl_3$) δ 7.76 (d, $^3J_{HH}$ = 8.4 Hz, 2H, H-C6), 7.71 (d, $^3J_{HH}$ = 8.6 Hz, 2H, H-C7), 7.35 (q, $^4J_{HF}$ = 1.7 Hz, 1H, H-C3), 7.29 (d, $^3J_{HH}$ = 8.1 Hz, 2H, H-C11), 7.13 (d, $^3J_{HH}$ = 8.2 Hz, 2H, H-C12), 2.32 (s, 3H, H-C14).

^{13}**C NMR** (126 MHz, $CDCl_3$) δ 137.45 (C13), 134.60 (C10), 132.14 (q, $^2J_{CF}$ = 32.7 Hz, C8), 130.27 (C6), 129.41 (C12), 128.76 (C5), 127.64 (q, $^4J_{CF}$ = 1.5 Hz, C11), 126.59 (q, $^1J_{CF}$ = 277.2 Hz, C1), 126.14 (q, $^3J_{CF}$ = 3.8 Hz, C7), 123.85 (q, $^1J_{CF}$ = 272.8 Hz, C9), 117.11 (C4), 102.43 (q, $^3J_{CF}$ = 3.2 Hz, C3), 32.43 (q, $^2J_{CF}$ = 35.7 Hz, C2), 21.17 (C14).

^{19}**F NMR** (470 MHz, $CDCl_3$) δ -62.99 (s, 3F, F-C9), -64.28 – -68.31 (m, 3F, F-C1).

^{19}**F{^{1}H} NMR** (377 MHz, $CDCl_3$) δ -62.99 (s, 3F, F-C9), -64.30 (s, 3F, F-C1).

GC-EI-MS: (*m/z*) requires: $[(C_{18}H_{12}F_6)^+]$ = 342.0838, (*m/z*) found: $[(C_{18}H_{12}F_6)^+]$ = 342.0837.

FT-IR ($\tilde{\nu}$ = cm^{-1}): 1619 (w), 1515 (w), 1412 (w), 1320 (s), 1303 (s), 1251 (m), 1159 (s), 1119 (s), 1062 (s), 1016 (m), 976 (w), 918 (m), 844 (s), 821 (m), 729 (m), 706 (m), 666 (w), 585 (m), 505 (w).

(2-Diazo-3,3,3-trifluoropropyl)benzene (Int 5)

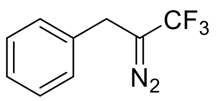

An oven-dried round bottom flask was charged with a stir bar. 1,1,1-Trifluoro-3-phenylpropan-2-one (1.88 g, 10.00 mmol, 1.0 eq.), EtOH (10.0 mL) and hydrazine hydrate (hydrazine 64%) (1.5 mL, 30.00 mmol, 3.0 eq.) were added to the reaction flask and the resulting mixture was stirred at 80 °C overnight. The reaction mixture was cooled to room temperature and 30 mL water was added. The layers were separated and the aqueous phase was extracted with Et_2O three times. The combined organic layers were washed with brine, dried over anhydrous Na_2SO_4, filtered and concentrated under reduced pressure. The crude hydrazone derivative was directly used in next step without further purification.

Oxalyl chloride (451 µL, 5.25 mmol, 1.05 eq.) was added in a dropwise manner to a stirred solution of DMSO (389 µL, 5.50 mmol, 1.1 eq.) in THF (20 mL) at -55 °C under Ar atmosphere. The reaction was maintained at -55 °C until gas evolution ceased (~20 min). Then, the reaction mixture was cooled further to -78 °C and a solution of hydrazone (1.01 g, 5.00 mmol, 1.0 eq.) and NEt_3 (1.5 mL, 10.5 mmol, 2.1 eq.) in THF (5 mL) was added dropwise to provide an orange solution containing a white precipitate. The reaction mixture was stirred at -78 °C for 2 h. The reaction mixture was warmed to rt and filtered. Then the filtrate was diluted with pentane, washed with water and brine, dried over anhydrous Na_2SO_4, filtered and concentrated under reduced pressure. The crude residue was passed through a short silica plug (*n*-pentane) to yield the title compound as a yellow oil (0.75 g, 3.76 mmol, 75%).

$\mathbf{R}_{\boldsymbol{f}}$ = 0.90 (*n*-pentane).

^{1}H NMR (400 MHz, $CDCl_3$) δ 7.38 – 7.33 (m, 2H), 7.32 – 7.28 (m, 1H), 7.26 – 7.22 (m, 1H), 3.54 (s, 2H).

^{19}F{^{1}H} NMR (377 MHz, $CDCl_3$) δ -58.63 (s, 3F).

Analysis by mass spectrometry was inconclusive.
Analytical data is in agreement with literature values.[8]

1-(3-Benzyl-3-(trifluoromethyl)cycloprop-1-en-1-yl)-4-(trifluoromethyl)benzene (S6)

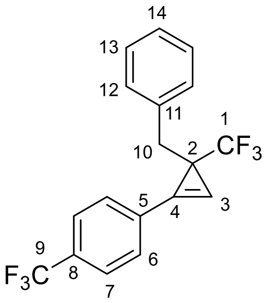

Compound **S6** was prepared according to General Procedure **B** using (2-diazo-3,3,3-trifluoropropyl)benzene (**Int 5**) (376.2 mg, 1.88 mmol, 1.0 eq.), 1-ethynyl-4-(trifluoromethyl)benzene (959.5 mg, 5.64 mmol, 3.0 eq.) and $Rh_2(esp)_2$ (14.3 mg, 0.02 mmol, 1 mol%). The reaction was performed at -78 °C. The crude residue was purified by column chromatography (*n*-pentane) to yield the title compound as a white solid (137.4 mg, 0.40 mmol, 21%).

R*f* = 0.30 (*n*-pentane).

^{1}H NMR (400 MHz, $CDCl_3$) δ 7.64 (d, $^3J_{HH}$ = 8.1 Hz, 2H, H-C7), 7.54 (d, $^3J_{HH}$ = 8.1 Hz, 2H, H-C6), 7.20 – 7.11 (m, 3H, H-C13~C14), 6.98 – 6.93 (m, 2H, H-C12), 6.89 (s, 1H, H-C3), 3.29 (d, $^2J_{HH}$ = 14.2 Hz, 1H, H-C10), 3.22 (d, $^2J_{HH}$ = 14.3 Hz, 1H, H-C10).

^{13}C NMR (126 MHz, $CDCl_3$) δ 137.25 (C11), 131.80 (q, $^2J_{CF}$ = 32.5 Hz, C8), 129.95 (*C*H), 129.92 (*C*H), 129.44 (C5), 128.49 (C13), 126.99 (C1), 126.62 (C14), 125.89 (q, $^3J_{CF}$ = 3.7 Hz, C7), 123.91 (q, $^1J_{CF}$ = 272.6 Hz, C9), 116.43 (C4), 102.84 (q, $^3J_{CF}$ = 3.1 Hz, C3), 35.35 (C10), 30.79 (q, $^2J_{CF}$ = 34.6 Hz, C2).

^{19}F NMR (470 MHz, $CDCl_3$) δ -62.93 (s, 3F, F-C9), -68.08 – -68.45 (m, 3F, F-C1).

^{19}F{^{1}H} NMR (377 MHz, $CDCl_3$) δ -62.93 (s, 3F, F-C9), -68.32 (s, 3F, F-C1).

GC-EI-MS: (*m/z*) requires: [$(C_{18}H_{12}F_6)^+$] = 342.0838, (*m/z*) found: [$(C_{18}H_{12}F_6)^+$] = 342.0837.

FT-IR ($\tilde{\nu}$ = cm^{-1}): 1613 (w), 1452 (w), 1412 (w), 1320 (s), 1280 (m), 1211 (w), 1119 (s), 1062 (s), 1016 (m), 993 (m), 844 (m), 746 (w), 700 (s), 591 (w), 517 (w).

Melting Point: 38-40 ˚C.

1-(3-(4-Bromophenyl)-3-(trifluoromethyl)cycloprop-1-en-1-yl)-3,5-dichlorobenzene (S7)

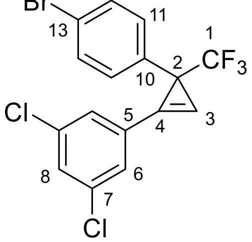

Compound **S7** was prepared according to General Procedure **B** using 1-bromo-4-(1-diazo-2,2,2-trifluoroethyl)benzene (**Int 2**) (530.1 mg, 2.00 mmol, 1.0 eq.). The crude residue was purified by column chromatography (*n*-pentane) to yield the title compound as a white wax (599.4 mg, 1.47 mmol, 73%).

R*f* = 0.25 (*n*-pentane).

^{1}H NMR (400 MHz, $CDCl_3$) δ 7.47 (d, $^4J_{HH}$ = 1.9 Hz, 2H, H-C6), 7.47 – 7.44 (m, 2H, H-C12), 7.43 (t, $^4J_{HH}$ = 1.9 Hz, 1H, H-C8), 7.30 (q, $^4J_{HF}$ = 1.5 Hz, 1H, H-C3), 7.24 (d, $^3J_{HH}$ = 8.0 Hz, 2H, H-C11).

^{13}C NMR (126 MHz, $CDCl_3$) δ 136.33 (C13), 136.00 (C7), 131.91 (C12), 130.78 (C8), 129.32 (q, $^4J_{CF}$ = 1.6 Hz, C11), 128.07 (C6), 127.65 (C5), 126.09 (q, $^1J_{CF}$ = 277.7 Hz, C1), 121.88 (C10), 115.81 (q, $^3J_{CF}$ = 2.6 Hz, C4), 102.09 (q, $^3J_{CF}$ = 2.9 Hz, C3), 32.58 (q, $^2J_{CF}$ = 35.8 Hz, C2).

^{19}F NMR (470 MHz, $CDCl_3$) δ -64.20 (s, 3F, F-C1).

^{19}F{^{1}H} NMR (377 MHz, $CDCl_3$) δ -64.20 (s, 3F, F-C1).

GC-EI-MS: (*m/z*) requires: [$(C_{16}H_8BrCl_2F_3)^+$] = 405.9133, (*m/z*) found: [$(C_{16}H_8BrCl_2F_3)^+$] = 405.9131.

FT-IR ($\tilde{\nu}$ = cm^{-1}): 2359 (w), 1561 (m), 1492 (w), 1418 (w), 1297 (s), 1165 (s), 1131 (s), 1073 (m), 1010 (w), 959 (w), 918 (s), 861 (m), 838 (m), 804 (m), 723 (s), 666 (w), 585 (w), 517 (w).

Methyl 4-(3-(4-bromophenyl)-3-(trifluoromethyl)cycloprop-1-en-1-yl)benzoate (S8)

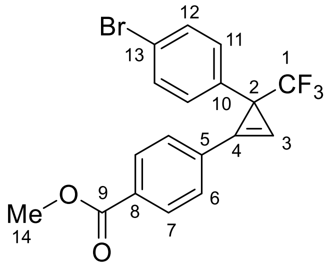

Compound **S8** was prepared according to General Procedure **B** using 1-bromo-4-(1-diazo-2,2,2-trifluoroethyl)benzene (**Int 2**) (1.06 g, 4.00 mmol, 1.0 eq.). The crude residue was purified by column chromatography (*n*-pentane:Et_2O 30:1-17.5:1) to yield the title compound as a pale yellow solid (0.38 g, 0.96 mmol, 24%).

R*f* = 0.25 (*n*-pentane:Et_2O 20:1).

^{1}H NMR (400 MHz, $CDCl_3$) δ 8.17 – 8.06 (m, 2H, H-C7), 7.73 – 7.64 (m, 2H, H-C6), 7.47 – 7.40 (m, 2H, H-C12), 7.31 (s, 1H), 7.29 – 7.25 (m, 2H, H-C11), 3.94 (s, 3H, H-C14).

^{13}C NMR (126 MHz, $CDCl_3$) δ 166.33 (C9), 136.84 (C13), 131.96 (C5), 131.80 (C12), 130.39 (C7), 129.96 (C6), 129.45 (q, $^4J_{CF}$ = 1.5 Hz, C11), 128.88 (C8), 126.31 (q, $^1J_{CF}$ = 277.3 Hz, C1), 121.68 (C10), 116.88 (C4), 101.62 (q, $^3J_{CF}$ = 3.0 Hz, C3), 52.57 (C14), 32.28 (q, $^2J_{CF}$ = 35.9 Hz, C2).

^{19}F NMR (470 MHz, $CDCl_3$) δ -64.18 (s, 3F, F-C1).

^{19}F{^{1}H} NMR (377 MHz, $CDCl_3$) δ -64.18 (s, 3F, F-C1).

GC-EI-MS: (*m/z*) requires: [$(C_{18}H_{12}O_2BrF_3)^+$] = 395.9967, (*m/z*) found: [$(C_{17}H_9BrF_6)^+$] = 395.9965.

FT-IR ($\tilde{\nu}$ = cm^{-1}): 1710 (s), 1492 (w), 1435 (w), 1274 (s), 1240 (m), 1154 (s), 1113 (s), 1073 (s), 1010 (m), 964 (w), 918 (s), 867 (m), 832 (m), 775 (m), 758 (m), 717 (s), 660 (w), 580 (w), 528 (w).

Melting Point: 107-109 ˚C.

4-(3-(4-Bromophenyl)-3-(trifluoromethyl)cycloprop-1-en-1-yl)benzonitrile (S9)

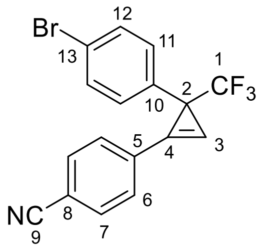

Compound **S9** was prepared according to General Procedure **B** using 1-bromo-4-(1-diazo-2,2,2-trifluoroethyl)benzene (**Int 2**) (530.1 mg, 2.00 mmol, 1.0 eq.). The crude residue was purified by column chromatography (*n*-pentane:Et_2O 7.5:1-5:1) to yield the title compound as a pale yellow solid (179.9 mg, 0.49 mmol, 25%).

R*f* = 0.25 (*n*-pentane:Et_2O 5:1).

^{1}H NMR (400 MHz, $CDCl_3$) δ 7.78 – 7.73 (m, 2H, H-C7), 7.73 – 7.69 (m, 2H, H-C6), 7.48 – 7.42 (m, 2H, H-C12), 7.40 (q, $^4J_{HF}$ = 1.5 Hz, 1H, H-C3), 7.24 (d, $^3J_{HH}$ = 8.4 Hz, 2H, H-C11).

^{13}C NMR (126 MHz, $CDCl_3$) δ 136.32 (C13), 132.97 (C7), 131.91 (C12), 130.45 (C6), 129.33 (q, $^4J_{CF}$ = 1.5 Hz, C11), 129.05 (C8), 126.10 (q, $^1J_{CF}$ = 277.6 Hz, C1), 121.91 (C10), 118.17 (C9), 116.43 (q, $^3J_{CF}$ = 2.5 Hz, C4), 114.21 (C5), 103.17 (q, $^3J_{CF}$ = 2.8 Hz, C3), 32.47 (q, $^2J_{CF}$ = 35.9 Hz, C2).

^{19}F NMR (470 MHz, $CDCl_3$) δ -64.20 (dt, $^4J_{HF}$ = 1.5, $^5J_{HF}$ = 0.7 Hz, 3F, F-C1).

^{19}F{^{1}H} NMR (377 MHz, $CDCl_3$) δ -64.20 (s, 3F, F-C1).

GC-EI-MS: (*m/z*) requires: [$(C_{17}H_9NBrF_3)^+$] = 362.9865, (*m/z*) found: [$(C_{17}H_9NBrF_3)^+$] = 362.9864.

FT-IR ($\tilde{\nu}$ = cm^{-1}): 2233 (w), 1492 (m), 1406 (w), 1303 (s), 1223 (w), 1165 (s), 1108 (s), 1073 (m), 1010 (m), 970 (w), 918 (s), 861 (m), 838 (s), 740 (m), 706 (m), 672 (m), 585 (w), 562 (m), 540 (m), 511 (m).

Melting Point: 83-85 ˚C.

(2-Hexyl-1-(trifluoromethyl)cycloprop-2-en-1-yl)benzene (S10)

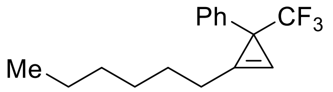

Compound **S10** was prepared according to General Procedure **B** using (1-diazo-2,2,2-trifluoroethyl)benzene (**Int 1**) (372.3 mg, 2.00 mmol, 1.0 eq.) and 1-octyne (661.2 mg, 6.00 mmol, 3.0 eq.). The crude residue was purified by column chromatography (*n*-pentane) to yield the title compound as a colorless oil (387.3 mg, 1.44 mmol, 72%).

R*f* = 0.65 (*n*-pentane).

^{1}H NMR (400 MHz, $CDCl_3$) δ 7.36 – 7.29 (m, 4H), 7.28 – 7.23 (m, 1H), 6.69 (q, *J* = 1.7 Hz, 1H), 2.54 (t, *J* = 7.3 Hz, 2H), 1.67 – 1.56 (m, 2H), 1.40 – 1.20 (m, 6H), 0.87 (t, *J* = 6.8 Hz, 3H).

^{19}F{^{1}H} NMR (377 MHz, $CDCl_3$) δ -64.48 (s, 3F).

GC-EI-MS: (*m/z*) requires: $[(C_{16}H_{19}F_3)^+]$ = 268.1433, (*m/z*) found: $[(C_{16}H_{19}F_3)^+]$ = 268.1440.

Analytical data is in agreement with literature values.[9]

(2-(Cyclohexylmethyl)-1-(trifluoromethyl)cycloprop-2-en-1-yl)benzene (S11)

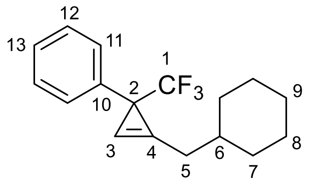

Compound **S11** was prepared according to General Procedure **B** using (1-diazo-2,2,2-trifluoroethyl)benzene (**Int 1**) (372.3 mg, 2.00 mmol, 1.0 eq.). The crude residue was purified by column chromatography (*n*-pentane) to yield the title compound as a colorless oil (396.5 mg, 1.41 mmol, 71%).

R*f* = 0.50 (*n*-pentane).

^{1}H NMR (400 MHz, $CDCl_3$) δ 7.36 – 7.28 (m, 4H, H-C11~C12), 7.26 – 7.22 (m, 1H. H-C13), 6.73 – 6.69 (m, 1H. H-C3), 2.52 – 2.34 (m, 2H, H-C5), 1.83 – 1.57 (m, 6H, H-C6~C9), 1.32 – 1.06 (m, 3H, H-C8~C9), 1.04 – 0.90 (m, 2H, H-C7).

^{13}C NMR (126 MHz, $CDCl_3$) δ 139.15 (C10), 128.43 (C12), 127.76 (q, $^4J_{CF}$ = 1.4 Hz, C11), 127.01 (q, $^1J_{CF}$ = 277.5 Hz, C1), 127.00 (C13), 120.01 (q, $^3J_{CF}$ = 2.4 Hz, C4), 97.71 (q, $^3J_{CF}$ = 3.2 Hz, C3), 36.24 (C6), 33.24 (C7), 33.13 (C7), 32.03 (C5), 31.29 (q, $^2J_{CF}$ = 35.1 Hz, C2), 26.37 (C9), 26.20 (C8), 26.18 (C8).

^{19}F NMR (470 MHz, $CDCl_3$) δ -64.45 (d, $^4J_{HF}$ = 1.9 Hz, 3F, F-C1).

^{19}F{^{1}H} NMR (377 MHz, $CDCl_3$) δ -64.45 (s, 3F, F-C1).

GC-EI-MS: (*m/z*) requires: [$(C_{17}H_{19}F_3)^+$] = 280.1433, (*m/z*) found: [$(C_{17}H_{19}F_3)^+$] = 280.1436.

FT-IR ($\tilde{\nu}$ = cm^{-1}): 2927 (m), 2853 (w), 2359 (w), 1446 (w), 1303 (m), 1251 (m), 1159 (s), 1119 (s), 976 (w), 924 (m), 907 (m), 700 (s), 649 (w), 580 (w).

Undec-10-yn-1-yl cyclopropanecarboxylate (Int 6)

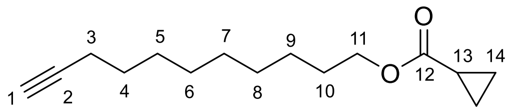

To a solution of cyclopropanecarboxylic acid (516.5 mg, 6.00 mmol, 1.5 eq.) in DCM (8 mL) was added 1-ethyl-3-(3-(dimethylamino)propyl)-carbodiimide hydrochloride (EDCI) (1.15 g, 6.00 mmol, 1.5 eq.), N,N-dimethylaminopyridine (48.9 mg, 0.40 mmol, 10 mol %) and then 10-undecyn-1-ol (673.1 mg, 4.00 mmol, 1.0 eq.). The reaction mixture was stirred at room temperature for 12 h, before DCM was added. The mixture was washed with brine and dried over Na_2SO_4. After removal of the solvent, the residue was purified by column chromatography (*n*-pentane:Et_2O 30:1) to yield the title compound as a colorless oil (890.3 mg, 3.77 mmol, 94%).

R_f = 0.35 (*n*-pentane:Et_2O 30:1).

^{1}H NMR (400 MHz, $CDCl_3$) δ 4.05 (t, $^3J_{HH}$ = 6.7 Hz, 2H, H-C11), 2.18 (td, $^3J_{HH}$ = 7.1 Hz, $^4J_{HH}$ = 2.6 Hz, 2H, H-C3), 1.93 (t, $^4J_{HH}$ = 2.7 Hz, 1H, H-C1), 1.68 – 1.57 (m, 3H, H-C10, H-C13), 1.55 – 1.47 (m, 2H, H-C4), 1.45 – 1.20 (m, 10H), 1.01 – 0.95 (m, 2H, H-C14), 0.87 – 0.80 (m, 2H, H-C14).

^{13}C NMR (151 MHz, $CDCl_3$) δ 175.12 (C12), 84.89 (C2), 68.21 (C1), 64.74 (C11), 29.48 (CH$_2$), 29.32 (CH$_2$), 29.14 (CH$_2$), 28.84 (CH$_2$), 28.81 (C10), 28.60 (C4), 26.03 (CH$_2$), 18.53 (C3), 13.05 (C13), 8.42 (C14).

ESI-MS: (*m/z*) requires: [$(C_{15}H_{24}O_2Na)^+$] = 259.1669, (*m/z*) found: [$(C_{15}H_{24}O_2Na)^+$] = 259.1668.

FT-IR ($\tilde{\nu}$ = cm^{-1}): 2927 (m), 2858 (m), 1728 (s), 1458 (w), 1400 (m), 1372 (m), 1268 (m), 1171 (s), 1073 (m), 1033 (w), 901 (w), 849 (w), 827 (w), 626 (s).

9-(3-Phenyl-3-(trifluoromethyl)cycloprop-1-en-1-yl)nonyl cyclopropanecarboxylate (S12)

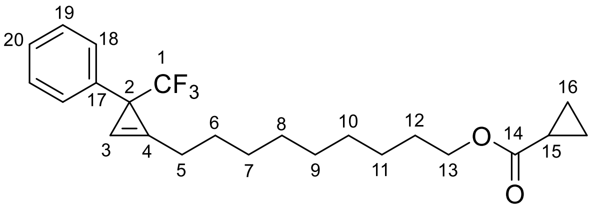

Compound **S12** was prepared according to General Procedure **B** using (1-diazo-2,2,2-trifluoroethyl)benzene (**Int 1**) (279.2 mg, 1.50 mmol, 1.0 eq.) and undec-10-yn-1-yl cyclopropanecarboxylate (**Int 6**) (797.7 mg, 3.38 mmol, 2.3 eq.). The crude residue was purified by column chromatography (*n*-pentane:Et_2O 30:1) to yield the title compound as a colorless oil (81.6 mg, 0.21 mmol, 14%).

$\mathbf{R_f}$ = 0.20 (*n*-pentane:Et_2O 30:1).

$^{\mathbf{1}}$**H NMR** (400 MHz, $CDCl_3$) δ 7.35 – 7.28 (m, 4H, H-C18~C19), 7.26 – 7.22 (m, 1H, H-20), 6.72 – 6.67 (m, 1H. H-C3), 4.05 (t, $^3J_{HH}$ = 6.8 Hz, 2H, H-C13), 2.60 – 2.47 (m, 2H, H-C5), 1.65 – 1.56 (m, 5H, H-C6, H-C12, H-C15), 1.38 – 1.21 (m, 10H, H-C7~C11), 1.02 – 0.95 (m, 2H, H-C16), 0.88 – 0.81 (m, 2H, H-C16).

$^{\mathbf{13}}$**C NMR** (126 MHz, $CDCl_3$) δ 175.14 (C14), 139.16 (C17), 128.44 (C19), 127.76 (q, $^4J_{CF}$ = 1.5 Hz, C18), 127.02 (C20), 126.98 (q, $^1J_{CF}$ = 277.2 Hz, C1), 120.94 (q, $^3J_{CF}$ = 2.2 Hz, C4), 97.29 (q, $^3J_{CF}$ = 3.1 Hz, C3), 64.73 (C13), 31.61 (q, $^2J_{CF}$ = 35.0 Hz, C2), 29.44 (CH_2), 29.29 (CH_2), 29.23 (CH_2), 29.20 (CH_2), 28.80 (C12), 26.80 (C6), 26.00 (C11), 24.39 (C5), 13.05 (C15), 8.42 (C16).

$^{\mathbf{19}}$**F NMR** (470 MHz, $CDCl_3$) δ -64.52 (d, $^4J_{HF}$ = 1.9 Hz, 3F, F-C1).

$^{\mathbf{19}}$**F{$^{\mathbf{1}}$H} NMR** (377 MHz, $CDCl_3$) δ -64.52 (s, 3F, F-C1).

GC-EI-MS: (*m/z*) requires: [$(C_{22}H_{29}O_2)^+$] = 325.2162, (*m/z*) found: [$(C_{22}H_{29}O_2)^+$] = 325.2162.

FT-IR ($\tilde{\nu}$ = cm^{-1}): 2933 (m), 2858 (w), 2359 (w), 1722 (s), 1498 (w), 1458 (w), 1406 (m), 1372 (w), 1303 (m), 1257 (m), 1159 (s), 1125 (s), 1079 (m), 1033 (w), 907 (m), 735 (s), 700 (s), 649 (m), 580 (w).

Diethyl 2-diazomalonate (Int 7)

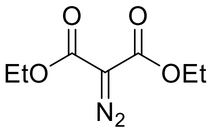

Triethylamine (3.3 mL, 24.00 mmol, 2.4 eq.) and diethyl malonate (1.60 g, 10.00 mmol, 1.0 eq.) were added to a solution of *p*-acetamidobenzenesulfonyl azide (*p*ABSA) (3.60 g, 15.00 mmol, 1.5 eq.) in CH_3CN (40 mL) at 0 °C and the resulting mixture was stirred for 18 h at room temperature. Thereafter the mixture was filtered and

the solvent was evaporated. The crude residue was purified by column chromatography (*n*-pentane:EA 10:1) to yield the title compound as a yellow oil (1.52 g, 8.18 mmol, 82%).

R*f* = 0.26 (*n*-pentane:EA 10:1).

^{1}H NMR (400 MHz, $CDCl_3$) δ 4.30 (q, *J* = 7.1 Hz, 4H), 1.32 (t, *J* = 7.1 Hz, 6H).

ESI-MS: (*m/z*) requires: $[(C_7H_{10}N_2O_4Na)^+]$ = 209.0533, (*m/z*) found: $[(C_7H_{10}N_2O_4Na)^+]$ = 209.0532.

Analytical data is in agreement with literature values.[10]

Diethyl 2-(4-(trifluoromethyl)phenyl)cycloprop-2-ene-1,1-dicarboxylate (S13)

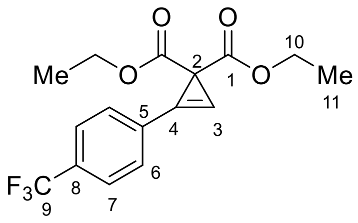

Compound **S13** was prepared according to General Procedure **B** using diethyl 2-diazomalonate (**Int 7**) (908.8 mg, 4.88 mmol, 1.0 eq.) and 1-ethynyl-4-(trifluoromethyl)benzene (2.49 g, 14.64 mmol, 3.0 eq.). The crude residue was purified by column chromatography (*n*-pentane:Et_2O 3:1) to yield the title compound as a yellow oil (1.12 g, 3.41 mmol, 70%).

R*f* = 0.30 (*n*-pentane:Et_2O 3:1).

^{1}H NMR (400 MHz, $CDCl_3$) δ 7.76 (d, $^3J_{HH}$ = 8.1 Hz, 2H, H-C6), 7.70 (d, $^3J_{HH}$ = 8.3 Hz, 2H, H-C7), 7.07 (s, 1H, H-C3), 4.21 (q, $^3J_{HH}$ J = 7.1 Hz, 4H, H-C10), 1.26 (t, $^3J_{HH}$ = 7.1 Hz, 6H, H-C11).

^{13}C NMR (126 MHz, $CDCl_3$) δ 170.52 (C1), 132.25 (q, $^2J_{CF}$ = 32.8 Hz, C8), 130.66 (C6), 127.83 (C5), 126.00 (q, $^3J_{CF}$ = 3.9 Hz, C7), 123.84 (q, $^1J_{CF}$ = 272.7 Hz, C9), 111.94 (C4), 98.65 (C3), 61.57 (C10), 33.59 (C2), 14.29 (C11).

^{19}F NMR (470 MHz, $CDCl_3$) δ -62.99 (s, 3F, F-C9).

^{19}F{^{1}H} NMR (377 MHz, $CDCl_3$) δ -62.99 (s, 3F, F-C9).

ESI-MS: (*m/z*) requires: $[(C_{16}H_{15}O_4F_3Na)^+]$ = 351.0815, (*m/z*) found: $[(C_{16}H_{15}O_4F_3Na)^+]$ = 351.0814.

FT-IR ($\tilde{\nu}$ = cm^{-1}): 2985 (w), 2359 (w), 1733 (s), 1412 (w), 1372 (w), 1326 (s), 1246 (m), 1171 (m), 1131 (s), 1068 (m), 1016 (m), 849 (w).

Diethyl 2-(3,5-bis(trifluoromethyl)phenyl)cycloprop-2-ene-1,1-dicarboxylate (S14)

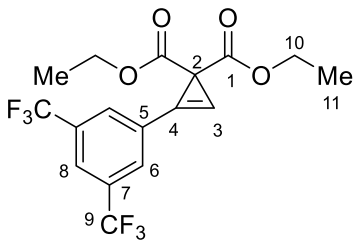

Compound **S14** was prepared according to General Procedure **B** using diethyl 2-diazomalonate (**Int 7**) (1.12 g, 6.00 mmol, 1.0 eq.) and 1-ethynyl-3,5-bis(trifluoromethyl)benzene (4.29 g, 18.00 mmol, 3.0 eq.). The crude residue was purified by column chromatography (*n*-pentane:Et_2O 4:1) to yield the title compound as a yellow solid (1.31 g, 3.31 mmol, 55%).

R*f* = 0.35 (*n*-pentane:Et_2O 4:1).

^{1}H NMR (500 MHz, $CDCl_3$) δ 8.08 (s, 2H, H-C6), 7.93 (s, 1H, H-C8), 7.20 (s, 1H, H-C3), 4.28 – 4.18 (m, 4H. H-C10), 1.27 (t, $^3J_{HH}$ = 7.1 Hz, 6H, H-C11).

^{13}C NMR (126 MHz, $CDCl_3$) δ 170.07 (C1), 132.75 (q, $^2J_{CF}$ = 33.9 Hz, C7), 130.13 (m, C6), 126.83 (C5), 124.01 (m, C8), 122.97 (q, $^1J_{CF}$ = 272.9 Hz, C9), 111.17 (C4), 100.56 (C3), 61.83 (C10), 33.90 (C2), 14.23 (C11).

^{19}F NMR (470 MHz, $CDCl_3$) δ -63.12 (s, 3F, F-C9).

^{19}F{^{1}H} NMR (377 MHz, $CDCl_3$) δ -63.12 (s, 3F, F-C9).

ESI-MS: (*m/z*) requires: [$(C_{17}H_{14}O_4F_6Na)^+$] = 419.0689, (*m/z*) found: [$(C_{17}H_{14}O_4F_6Na)^+$] = 419.0689.

FT-IR ($\tilde{\nu}$ = cm^{-1}): 3151 (w), 2985 (w), 1722 (s), 1458 (w), 1372 (m), 1280 (s), 1165 (s), 1125 (s), 1068 (s), 1027 (m), 907 (m), 878 (w), 838 (m), 752 (m), 700 (m), 683 (m), 620 (w), 562 (w).
Melting Point: 58-59 ˚C.

Diethyl 2-(4-nitrophenyl)cycloprop-2-ene-1,1-dicarboxylate (S15)

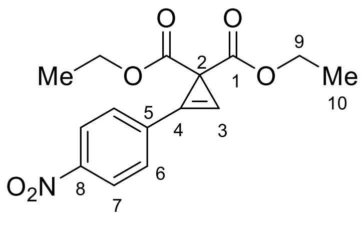

Compound **S15** was prepared according to General Procedure **B** using diethyl 2-diazomalonate (**Int 7**) (0.75 g, 4.00 mmol, 1.0 eq.). The crude residue was purified by column chromatography (*n*-pentane:Et_2O 3:1-2.5:1) to yield the title compound as a yellow solid (0.19 g, 0.62 mmol, 16%).

R*f* = 0.19 (*n*-pentane:Et_2O 2.5:1).

^{1}H NMR (400 MHz, $CDCl_3$) δ 8.34 – 8.27 (m, 2H, H-C7), 7.85 – 7.78 (m, 2H, H-C6), 7.20 (s, 1H, H-C3), 4.22 (q, $^{3}J_{HH}$ = 7.1 Hz, 4H, H-C9), 1.27 (t, $^{3}J_{HH}$ = 7.1 Hz, 6H, H-C10).

^{13}C NMR (151 MHz, $CDCl_3$) δ 170.20 (C1), 148.77 (C5), 131.17 (C6), 130.46 (C8), 124.28 (C7), 111.59 (C4), 100.90 (C3), 61.73 (C9), 33.81 (C2), 14.29 (C10).

ESI-MS: (*m/z*) requires: $[(C_{15}H_{15}NO_6Na)^+]$ = 328.0803, (*m/z*) found: $[(C_{15}H_{15}NO_6Na)^+]$ = 328.0789.

FT-IR ($\tilde{\nu}$ = cm^{-1}): 3140 (w), 2990 (w), 1705 (s), 1596 (w), 1515 (m), 1475 (w), 1446 (w), 1343 (m), 1251 (s), 1108 (m), 1068 (s), 1016 (m), 918 (w), 849 (m), 792 (w), 729 (s).

Melting Point: 72-74 ˚C.

Diethyl 2-(4-bromophenyl)cycloprop-2-ene-1,1-dicarboxylate (S16)

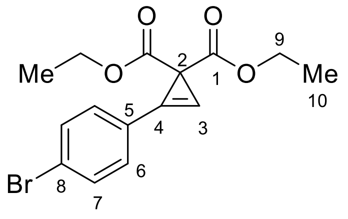

Compound **S16** was prepared according to General Procedure **B** using diethyl 2-diazomalonate (**Int 7**) (0.75 g, 4.00 mmol, 1.0 eq.). The crude residue was purified by column chromatography (*n*-pentane:Et_2O 3:1) to yield the title compound as a pale yellow solid (0.36 g, 1.06 mmol, 26%).

R*f* = 0.19 (*n*-pentane:Et_2O 3:1).

^{1}H NMR (400 MHz, $CDCl_3$) δ 7.62 – 7.55 (m, 2H, H-C7), 7.54 – 7.47 (m, 2H, H-C6), 6.94 (s, 1H, H-C3), 4.20 (q, $^{3}J_{HH}$ = 7.1 Hz, 4H, H-C9), 1.25 (t, $^{3}J_{HH}$ = 7.1 Hz, 6H, H-C10).

^{13}C NMR (126 MHz, $CDCl_3$) δ 170.72 (C1), 132.35 (C7), 131.83 (C6), 125.19 (C5), 123.35 (C8), 112.00 (C4), 96.56 (C3), 61.46 (C9), 33.46 (C2), 14.31 (C10).

ESI-MS: (*m/z*) requires: $[(C_{15}H_{15}O_4BrH)^+]$ = 339.0227, (*m/z*) found: $[(C_{15}H_{15}O_4BrH)^+]$ = 339.0226.

FT-IR ($\tilde{\nu}$ = cm^{-1}): 3145 (w), 2985 (w), 1710 (s), 1584 (w), 1475 (w), 1395 (w), 1366 (w), 1280 (m), 1240 (s), 1148 (m), 1056 (s), 1010 (m), 918 (m), 832 (m), 792 (w), 735 (m), 706 (m), 631 (w).

Melting Point: 43-45 ˚C.

Dimethyl 2-diazomalonate (Int 8)

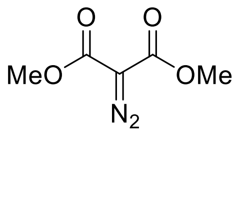

Triethylamine (6.7 mL, 48.00 mmol, 2.4 eq.) and dimethyl malonate (2.64 g, 20.00 mmol, 1.0 eq.) were added to a solution of *p*-acetamidobenzenesulfonyl azide (*p*ABSA) (7.21 g, 30.00 mmol, 1.5 eq.) in CH_3CN (80 mL) at 0 °C and

the resulting mixture was stirred for 18 h at room temperature. Thereafter the mixture was filtered and the solvent was evaporated. The crude residue was purified by column chromatography (*n*-pentane:EA 5:1) to yield the title compound as a yellow oil (2.64 g, 16.70 mmol, 83%).

R*f* = 0.31 (*n*-pentane:EA 5:1).

^{1}H NMR (400 MHz, $CDCl_3$) δ 3.84 (s, 6H).

ESI-MS: (*m/z*) requires: $[(C_5H_6N_2O_4Na)^+]$ = 181.0220, (*m/z*) found: $[(C_5H_6N_2O_4Na)^+]$ = 181.0219.

Analytical data is in agreement with literature values.[11]

Dimethyl 2-(4-(trifluoromethyl)phenyl)cycloprop-2-ene-1,1-dicarboxylate (S17)

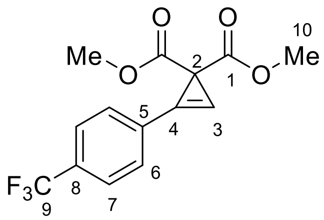

Compound **S17** was prepared according to General Procedure **B** using dimethyl 2-diazomalonate (**Int 8**) (0.63 g, 4.00 mmol, 1.0 eq.). The crude residue was purified by column chromatography (*n*-pentane:Et_2O 3:1-2.5:1) to yield the title compound as a yellow solid (0.73 g, 2.43 mmol, 61%).

R*f* = 0.30 (*n*-pentane:Et_2O 2.5:1).

^{1}H NMR (400 MHz, $CDCl_3$) δ 7.75 (d, $^{3}J_{HH}$ = 8.4 Hz, 2H, H-C6), 7.71 (d, $^{3}J_{HH}$ = 8.4 Hz, 2H, H-C7), 7.06 (s, 1H, H-C3), 3.75 (s, 6H, H-C10).

^{13}C NMR (151 MHz, $CDCl_3$) δ 170.83 (C1), 132.40 (q, $^{2}J_{CF}$ = 32.9 Hz, C8), 130.71 (C6), 127.59 (C5), 126.07 (q, $^{3}J_{CF}$ = 3.8 Hz, C7), 123.81 (q, $^{1}J_{CF}$ = 272.5 Hz, C9), 111.68 (C4), 98.46 (C3), 52.72 (C10), 33.12 (C2).

^{19}F NMR (564 MHz, $CDCl_3$) δ -63.03 (s, 3F, F-C9).

^{19}F{^{1}H} NMR (377 MHz, $CDCl_3$) δ -63.03 (s, 3F, F-C9).

ESI-MS: (*m/z*) requires: $[(C_{14}H_{11}O_4F_3Na)^+]$ = 323.0502, (*m/z*) found: $[(C_{14}H_{11}O_4F_3Na)^+]$ = 323.0499.

FT-IR ($\tilde{\nu}$ = cm^{-1}): 2956 (w), 1733 (s), 1435 (w), 1412 (w), 1326 (s), 1280 (m), 1251 (m), 1171 (m), 1131 (m), 1062 (s), 1016 (w), 849 (w), 746 (w), 695 (w).

Melting Point: 40-42 ˚C.

Ethyl 2-diazopropanoate (Int 9)

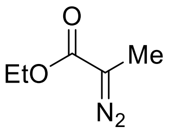

DBU (13.5 mL, 90.00 mmol, 3.0 eq.) was added slowly to a stirred solution of ethyl 2-methyl-3-oxobutanoate (4.33 g, 30.00 mmol, 1.0 eq.) and *p*-acetamidobenzenesulfonyl azide (*p*ABSA) (10.81 g, 45.00 mmol, 1.5 eq.) in CH_3CN (60 mL) at 0 °C. The reaction mixture was then allowed to warm to room temperature. After stirring for 12 h, the reaction mixture was quenched with a saturated aqueous solution of NH_4Cl. The layers were separated and the aqueous phase was extracted three times with Et_2O. The combined organic layers were dried over anhydrous Na_2SO_4, filtered and concentrated under reduced pressure. The crude residue was purified by column chromatography (*n*-pentane:Et_2O 20:1) to yield the title compound as a yellow oil (2.29 g, 17.88 mmol, 60%).

R_f = 0.25 (*n*-pentane:Et_2O 20:1).

^{1}H NMR (400 MHz, $CDCl_3$) δ 4.22 (q, *J* = 7.1 Hz, 2H), 1.96 (s, 3H), 1.27 (t, *J* = 7.1 Hz, 3H).

ESI-MS: (*m/z*) requires: $[(C_5H_8N_2O_2Na)^+]$ = 151.0478, (*m/z*) found: $[(C_5H_8N_2O_2Na)^+]$ = 151.0479.

Analytical data is in agreement with literature values.[12]

Ethyl 1-methyl-2-(4-(trifluoromethyl)phenyl)cycloprop-2-ene-1-carboxylate (S18)

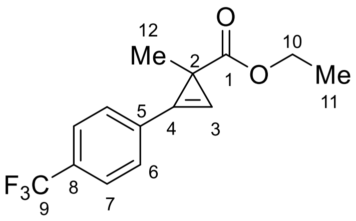

Compound **S18** was prepared according to General Procedure **B** using ethyl 2-diazopropanoate (**Int 9**) (640.7 mg, 5.00 mmol, 1.0 eq.). The crude residue was purified by column chromatography (*n*-pentane:DCM 2:1-1.5:1) to yield the title compound as a yellow oil (328.5 mg, 1.22 mmol, 24%).

R_f = 0.30 (*n*-pentane:DCM 1:1).

^{1}H NMR (400 MHz, $CDCl_3$) δ 7.67 (d, $^3J_{HH}$ = 8.0 Hz, 2H, H-C7), 7.61 (d, $^3J_{HH}$ = 8.7 Hz, 2H, H-C6), 7.15 (s, 1H, H-C3), 4.12 (q, $^3J_{HH}$ = 7.1 Hz, 2H, H-C10), 1.54 (s, 3H, H-C12), 1.20 (t, $^3J_{HH}$ = 7.1 Hz, 3H, H-C11).

^{13}C NMR (126 MHz, $CDCl_3$) δ 176.20 (C1), 131.37 (app. q, $^2J_{CF}$ = 32.6 Hz, C8), 129.88 (C6), 129.83 (C5), 125.91 (q, $^3J_{CF}$ = 3.8 Hz, C7), 124.00 (q, $^1J_{CF}$ = 272.7 Hz, C9), 118.48 (C4), 106.12 (C3), 60.89 (C10), 25.37 (C2), 20.07 (C12), 14.47 (C11).

^{19}F NMR (470 MHz, $CDCl_3$) δ -62.86 (s, 3F, F-C9).

$^{19}F\{^1H\}$ NMR (377 MHz, $CDCl_3$) δ -62.86 (s, 3F, F-C9).

GC-EI-MS: (*m/z*) requires: $[(C_{14}H_{13}O_2F_3)^+]$ = 270.0862, (*m/z*) found: $[(C_{14}H_{13}O_2F_3)^+]$ = 270.0863.

FT-IR ($\tilde{\nu}$ = cm^{-1}): 2985 (w), 1768 (m), 1716 (m), 1619 (w), 1446 (w), 1412 (w), 1320 (s), 1263 (m), 1165 (s), 1119 (s), 1068 (s), 1016 (s), 959 (m), 913 (m), 844 (m), 735 (m), 695 (w), 597 (w).

Ethyl 2-hexyl-1-methylcycloprop-2-ene-1-carboxylate (S19)

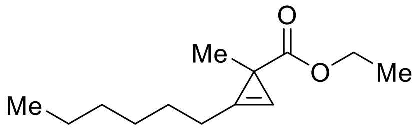

Compound **S19** was prepared according to General Procedure **B** using ethyl 2-diazopropanoate (**Int 9**) (640.7 mg, 5.00 mmol, 1.0 eq.). The crude residue was purified by column chromatography (*n*-pentane:DCM 2.5:1-1.5:1) to yield the title compound as a colorless oil (284.6 mg, 1.35 mmol, 27%).

R*f* = 0.20 (*n*-pentane:DCM 2:1).

^1H NMR (400 MHz, $CDCl_3$) δ 6.40 (s, 1H), 4.18 – 3.98 (m, 2H), 2.45 (td, *J* = 7.3, 1.3 Hz, 2H), 1.60 – 1.49 (m, 2H), 1.41 – 1.25 (m, 9H), 1.21 (t, *J* = 7.1 Hz, 3H), 0.88 (t, *J* = 6.7 Hz, 3H).

ESI-MS: (*m/z*) requires: $[(C_{13}H_{22}O_2Na)^+]$ = 233.1512, (*m/z*) found: $[(C_{13}H_{22}O_2Na)^+]$ = 233.1512. Analytical data is in agreement with literature values.[13]

Ethyl 2-diazo-2-phenylacetate (Int 10)

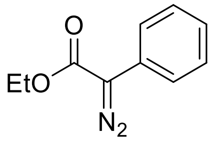

DBU (4.8 mL, 32.00 mmol, 1.6 eq.) was added slowly to a stirred solution of ethyl phenylacetate (3.28 g, 20.00 mmol, 1.0 eq.) and *p*-acetamidobenzenesulfonyl azide (*p*ABSA) (7.21 g, 30.00 mmol, 1.5 eq.) in CH_3CN (80 mL) at 0 °C. The reaction mixture was then allowed to warm to room temperature. After stirring for 12 h, the reaction mixture was quenched with a saturated aqueous solution of NH_4Cl. The layers were separated and the aqueous phase was extracted three times with Et_2O. The combined organic layers were dried over anhydrous Na_2SO_4, filtered and concentrated under reduced pressure. The crude residue was purified by column chromatography (*n*-pentane:EA 40:1) to yield the title compound as an orange oil (2.89 g, 15.19 mmol, 76%).

R*f* = 0.28 (*n*-pentane:EA 40:1).

^{1}H NMR (400 MHz, $CDCl_3$) δ 7.53 – 7.43 (m, 2H), 7.42 – 7.35 (m, 2H), 7.18 (tt, *J* = 7.4, 1.2 Hz, 1H), 4.34 (q, *J* = 7.1 Hz, 2H), 1.34 (t, *J* = 7.1 Hz, 3H).

ESI-MS: (*m/z*) requires: $[(C_{10}H_{10}N_2O_2Na)^+]$ = 213.0635, (*m/z*) found: $[(C_{10}H_{10}N_2O_2Na)^+]$ = 213.0635.

Analytical data is in agreement with literature values.[14]

Ethyl 1-phenyl-2-(4-(trifluoromethyl)phenyl)cycloprop-2-ene-1-carboxylate (S20)

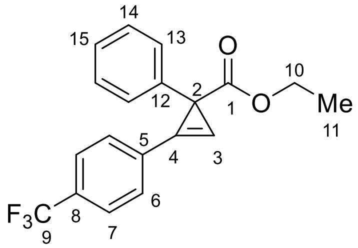

Compound **S20** was prepared according to General Procedure **B** using ethyl 2-diazo-2-phenylacetate (**Int 10**) (1.14 g, 6.00 mmol, 1.0 eq.). The crude residue was purified by column chromatography (*n*-pentane:Et_2O 50:1) to yield the title compound as a pale yellow wax (1.29 g, 3.88 mmol, 65%).

R_f = 0.31 (*n*-pentane:Et_2O 50:1).

^{1}H NMR (400 MHz, $CDCl_3$) δ 7.73 (d, $^3J_{HH}$ = 8.2 Hz, 2H, H-C6), 7.68 (d, $^3J_{HH}$ = 8.2 Hz, 2H, H-C7), 7.39 (s, 1H, H-C3), 7.36 (dd, $^3J_{HH}$ = 8.3 Hz, $^4J_{HH}$ = 1.4 Hz, 2H, H-C13), 7.33 – 7.27 (m, 2H, H-C14), 7.25 – 7.20 (m, 1H, H-C15), 4.21 (q, $^3J_{HH}$ = 7.1 Hz, 2H, H-C10), 1.23 (t, $^3J_{HH}$ = 7.1 Hz, 3H, H-C11).

^{13}C NMR (126 MHz, $CDCl_3$) δ 174.08 (C1), 140.53 (C12), 131.72 (app. q, $^2J_{CF}$ = 32.9 Hz, C8), 130.16 (C6), 129.22 (app. q, $^5J_{CF}$ = 1.4 Hz, C5), 128.31 (C14), 128.17 (C13), 126.83 (C15), 126.01 (q, $^3J_{CF}$ = 3.8 Hz, C7), 123.92 (q, $^1J_{CF}$ = 272.4 Hz, C9), 116.73 (C4), 103.72 (C3), 61.25 (C10), 34.16 (C2), 14.44 (C11).

^{19}F NMR (470 MHz, $CDCl_3$) δ -62.90 (s, 3F, F-C9).

^{19}F{^{1}H} NMR (377 MHz, $CDCl_3$) δ -62.90 (s, 3F, F-C9).

ESI-MS: (*m/z*) requires: $[(C_{19}H_{15}O_2F_3Na)^+]$ = 355.0916, (*m/z*) found: $[(C_{19}H_{15}O_2F_3Na)^+]$ = 355.0916.

FT-IR ($\tilde{\nu}$ = cm^{-1}): 2985 (w), 1716 (m), 1613 (w), 1492 (w), 1446 (w), 1406 (w), 1320 (s), 1211 (m), 1165 (s), 1125 (s), 1062 (s), 1016 (m), 970 (w), 913 (m), 844 (m), 735 (m), 695 (s).

Ethyl 2-hexyl-1-phenylcycloprop-2-ene-1-carboxylate (S21)

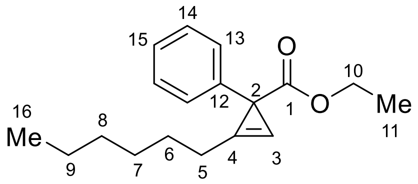

Compound **S21** was prepared according to General Procedure **B** using ethyl 2-diazo-2-phenylacetate (**Int 10**) (380.4 mg, 2.00 mmol, 1.0 eq.). The crude residue was purified by column chromatography (*n*-pentane:Et_2O 20:1-15:1) to yield the title compound as a yellow oil (160.0 mg, 0.59 mmol, 29%).

$\mathbf{R_f}$ = 0.25 (*n*-pentane:Et_2O 20:1).

^{1}H NMR (400 MHz, $CDCl_3$) δ 7.32 – 7.26 (m, 4H, H-C13~C14), 7.23 – 7.15 (m, 1H, H-C15), 6.66 (s, 1H, H-C3), 4.15 (q, $^3J_{HH}$ = 7.1 Hz, 2H, H-C10), 2.55 (t, $^3J_{HH}$ = 7.6 Hz, 2H, H-C5), 1.62 – 1.54 (m, 2H, H-C6), 1.37 – 1.18 (m, 9H, H-C7~C9, H-C11), 0.86 (t, $^3J_{HH}$ = 6.7 Hz, 3H, H-C16).

^{13}C NMR (151 MHz, $CDCl_3$) δ 175.40 (C1), 141.92 (C12), 128.18 (*C*H), 127.87 (*C*H), 126.02 (C15), 120.87 (C4), 96.85 (C3), 60.56 (C10), 33.09 (C2), 31.42 (*C*H_2), 28.80 (*C*H_2), 26.71 (C6), 24.44 (C5), 22.47 (*C*H_2), 14.31 (C11), 13.99 (C16).

ESI-MS: (*m/z*) requires: $[(C_{18}H_{24}O_2Na)^+]$ = 295.1669, (*m/z*) found: $[(C_{18}H_{24}O_2Na)^+]$ = 295.1663.

FT-IR ($\tilde{\nu}$ = cm^{-1}): 2933 (m), 2858 (w), 1716 (s), 1492 (w), 1446 (w), 1366 (w), 1280 (m), 1205 (s), 1096 (m), 1039 (m), 964 (w), 913 (w), 821 (w), 758 (m), 729 (m), 695 (s).

(3R,8R,10S,13R)-10,13-Dimethyl-17-((R)-6-methylheptan-2-yl)hexadecahydro-1H-cyclopenta[a]phenanthren-3-yl 4-(3-(4-bromophenyl)-3-(trifluoromethyl)cycloprop-1-en-1-yl)benzoate (S22)

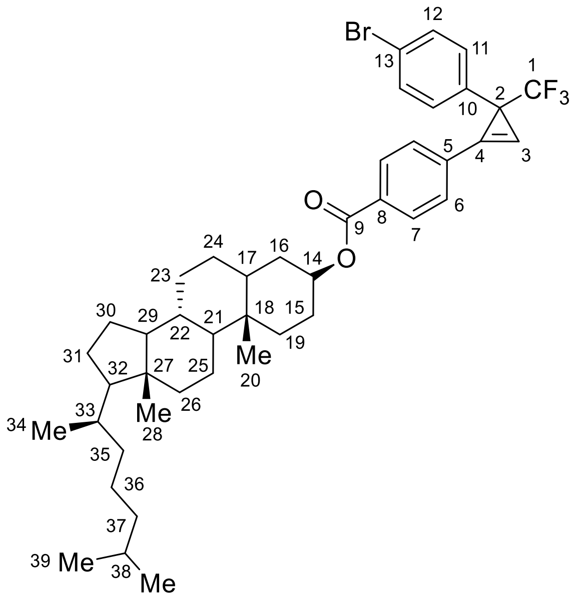

A mixture of methyl 4-(3-(4-bromophenyl)-3-(trifluoromethyl)cycloprop-1-en-1-yl)benzoate (**S8**) (198.6 mg, 0.50 mmol, 1.0 eq.) and LiOH (71.8 mg, 3.00 mmol, 6.0 eq.) in THF (1.5 mL) and H_2O (1.5 mL) was vigorously stirred at rt for 8 h. After the reaction was quenched with saturated aqueous NH_4Cl, the aqueous layer was acidified with concentrated HCl (pH = 3) and extracted with DCM. The organic layer was dried with Na_2SO_4, concentrated and the crude 4-(3-(4-bromophenyl)-3-(trifluoromethyl)cycloprop-1-en-1-yl)benzoic acid was directly used in next step without further purification.

To a solution of crude 4-(3-(4-bromophenyl)-3-(trifluoromethyl)cycloprop-1-en-1-yl)benzoic acid (187.0 mg, 0.49 mmol, 1.0 eq.) in DCM (1 mL) were added 1-ethyl-3-(3-(dimethylamino)propyl)-carbodiimide hydrochloride (EDCI) (140.9 mg, 0.74 mmol, 1.5 eq.), N,N-dimethylaminopyridine (6.0 mg, 0.05 mmol, 10 mol %) and then dihydrocholesterol (209.5 mg, 0.54 mmol, 1.1 eq.). The reaction mixture was stirred at room temperature for 12 h, before DCM was added. The mixture was washed with brine and dried over Na_2SO_4. After removal of the solvent, the residue was purified by column chromatography (*n*-pentane:Et_2O 20:1) to yield the title compound as a white solid (297.6 mg, 0.39 mmol, 81%).

$\mathbf{R_f}$ = 0.35 (*n*-pentane: Et_2O 20:1).

^{1}H NMR (400 MHz, $CDCl_3$) δ 8.19 – 8.06 (m, 2H, H-C7), 7.71 – 7.62 (m, 2H, H-C6), 7.46 – 7.39 (m, 2H, H-C12), 7.29 (q, $^4J_{HF}$ = 1.5 Hz, 1H, H-C3), 7.28 – 7.25 (m, 2H, H-C11), 5.04 – 4.88 (m, 1H, H-C14), 2.03 – 1.90 (m, 2H), 1.87 – 1.76 (m, 2H), 1.75 – 1.61 (m, 3H), 1.57 – 1.44 (m, 4H), 1.41 – 1.20 (m, 9H), 1.18 – 0.97 (m, 9H), 0.95 – 0.89 (m, 4H), 0.89 – 0.86 (m, 6H), 0.86 – 0.84 (m, 3H), 0.74 – 0.63 (m, 4H).

^{13}C NMR (126 MHz, $CDCl_3$) δ 165.35 (C9), 136.91 (C13), 132.81 (C5), 131.78 (C12), 130.34 (C7), 129.88 (C6), 129.46 (q, $^4J_{CF}$ = 1.6 Hz, C11), 128.61 (C8), 126.33 (q, $^1J_{CF}$ = 277.4 Hz, C1),

121.65 (C10), 116.89 (C4), 101.42 (C3), 75.07 (C14), 56.58 (*C*H), 56.44 (*C*H), 54.40 (*C*H), 44.87 (*C*H), 42.76 (*C*), 40.15 (*C*H_2), 39.67 (*C*H_2), 36.93 (*C*H_2), 36.33 (*C*H_2), 35.96 (*C*H), 35.67 (*C*H), 35.66 (*C*), 34.24 (*C*H_2), 32.23 (q, $^2J_{CF}$ = 35.9 Hz, C2), 32.16 (*C*H_2), 28.79 (*C*H_2), 28.40 (*C*H_2), 28.17 (*C*H), 27.71 (*C*H_2), 24.37 (*C*H_2), 24.00 (*C*H_2), 22.97(*C*H_3), 22.71 (*C*H_3), 21.39 (*C*H_2), 18.83 (*C*H_3), 12.45 (*C*H_3), 12.23 (*C*H_3).

^{19}F NMR (470 MHz, $CDCl_3$) δ -64.19 (d, $^4J_{HF}$ = 1.4 Hz, 3F, F-C1).

^{19}F{^{1}H} NMR (377 MHz, $CDCl_3$) δ -64.19 (s, 3F, F-C1).

ESI-MS: (*m/z*) requires: $[(C_{44}H_{55}O_2BrF_3)^-]$ = 751.3343, (*m/z*) found: $[(C_{44}H_{55}O_2BrF_3)^-]$ = 751.3339.

FT-IR ($\tilde{\nu}$ = cm^{-1}): 3335 (m), 2973 (m), 2927 (m), 2359 (w), 1716 (w), 1383 (w), 1274 (m), 1165 (w), 1085 (s), 1045 (s), 918 (w), 878 (s).
Melting Point: 99-100 ˚C.

Ethyl 2-hexylcycloprop-2-ene-1-carboxylate (S23)

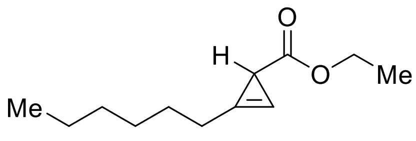

Compound **S23** was prepared according to General Procedure **B** using ethyl 2-diazoacetate (456.4 mg, 4.00 mmol, 1.0 eq.) and 1-octyne (1.32 g, 12.00 mmol, 3.0 eq.). The crude residue was purified by column chromatography (cyclohexane:DCM 1:1) to yield the title compound as a colorless oil (394.8 mg, 2.01 mmol, 50%).

R*f* = 0.10 (cyclohexane:DCM 1:1).

^{1}H NMR (400 MHz, $CDCl_3$) δ 6.32 (q, *J* = 1.4 Hz, 1H), 4.21 – 4.04 (m, 2H), 2.49 (td, *J* = 7.3, 1.4 Hz, 2H), 2.12 (d, *J* = 1.6 Hz, 1H), 1.62 – 1.55 (m, 2H), 1.40 – 1.20 (m, 9H), 0.95 – 0.82 (m, 3H).

ESI-MS: (*m/z*) requires: $[(C_{12}H_{20}O_2Na)^+]$ = 219.1356, (*m/z*) found: $[(C_{12}H_{20}O_2Na)^+]$ = 219.1356.

Analytical data is in agreement with literature values.[15]

Tert-butyldimethyl(undec-10-yn-1-yloxy)silane (Int 11)

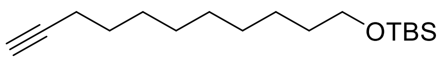

To a solution of 10-undecyn-1-ol (1.01 g, 6.00 mmol, 1.0 eq.) in DCM (15 mL) was added imidazole (612.7 mg, 9.00 mmol, 1.5 eq.) and DMAP (73.3 mg, 0.60 mmol, 10 mol%). TBSCl (1.36 g, 9.00 mmol, 1.5 eq.) was

added in one portion and the reaction was stirred at room temperature for 1 h. The reaction was quenched with a saturated aqueous solution of $NaHCO_3$. The layers were separated and the aqueous layer was extracted two times with DCM. The combined organic layers were dried over Na_2SO_4, filtered, and concentrated under reduced pressure. The crude residue was purified by column chromatography (*n*-pentane:Et_2O 80:1-70:1) to yield the title compound as a colorless oil (2.89 g, 15.19 mmol, 76%).

R*f* = 0.35 (*n*-pentane:Et_2O 80:1).

^{1}H NMR (400 MHz, $CDCl_3$) δ 3.60 (t, *J* = 6.6 Hz, 2H), 2.18 (td, *J* = 7.1, 2.6 Hz, 2H), 1.93 (t, *J* = 2.7 Hz, 1H), 1.53 – 1.45 (m, 4H), 1.44 – 1.35 (m, 2H), 1.34 – 1.25 (m, 8H), 0.89 (s, 9H), 0.05 (s, 6H).

GC-EI-MS: (*m/z*) requires: $[(C_{13}H_{25}OSi)^+]$ = 225.1669, (*m/z*) found: $[(C_{13}H_{25}OSi)^+]$ = 225.1673.

Analytical data is in agreement with literature values.[16]

Ethyl 2-(9-hydroxynonyl)cycloprop-2-ene-1-carboxylate (S24)

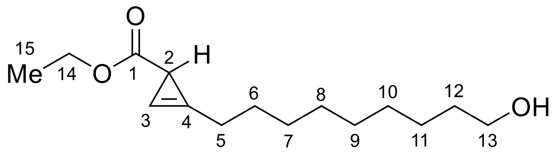

The cyclopropene precursor was prepared according to General Procedure **B** using ethyl 2-diazoacetate (456.4 mg, 4.00 mmol, 1.0 eq.) and tert-butyldimethyl(undec-10-yn-1-yloxy)silane (**Int 11**) (2.83 g, 10.00 mmol, 2.5 eq.). The crude residue was passed through a short silica plug (*n*-pentane:Et_2O 15:1) to afford ethyl 2-(9-((tert-butyldimethylsilyl)oxy)nonyl)cycloprop-2-ene-1-carboxylate, which was used without any further purification for the next step.

To a solution of ethyl 2-(9-((tert-butyldimethylsilyl)oxy)nonyl)cycloprop-2-ene-1-carboxylate (916.0 mg, 2.50 mmol, 1.0 eq.) in 10.0 mL of THF was added TBAF (3.00 mmol, 1.0 M in THF, 1.2 eq.). The solution was stirred at room temperature for 2 h. Then, solvent was removed under reduced pressure and the crude product was purified by column chromatography (*n*-pentane:Et_2O 1:1) to yield the title compound **S24** as a colorless oil (576.9 mg, 2.27 mmol, 57% over two steps).

R*f* = 0.20 (*n*-pentane:Et_2O 1:1).

^{1}H NMR (400 MHz, $CDCl_3$) δ 6.32 (q, $^3J_{HH}$ = 1.4 Hz, $^4J_{HH}$ = 1.4 Hz, 1H, H-C3), 4.21 – 4.06 (m, 2H, H-C14), 3.70 – 3.59 (m, 2H, H-C13), 2.49 (td, $^3J_{HH}$ = 7.2 Hz, $^4J_{HH}$ =1.4 Hz, 2H, H-C5), 2.12

(d, $^3J_{HH}$ = 1.5 Hz, 1H, H-C2), 1.63 – 1.54 (m, 4H, H-C6, H-C12), 1.42 – 1.18 (m, 13H, H-C7~C11, H-C15).

^{13}C NMR (151 MHz, $CDCl_3$) δ 176.80 (C1), 115.75 (C4), 94.08 (C3), 63.17 (C13), 60.29 (C14), 32.91 (C12), 29.55 (CH_2), 29.46 (CH_2), 29.30 (CH_2), 29.20 (CH_2), 26.77 (C6), 25.82 (CH_2), 25.08 (C5), 19.87 (C2), 14.52 (C15).

ESI-MS: (*m/z*) requires: $[(C_{15}H_{26}O_3Na)^+]$ = 277.1774, (*m/z*) found: $[(C_{15}H_{26}O_3Na)^+]$ = 277.1782.

FT-IR ($\tilde{\nu}$ = cm^{-1}): 2927 (m), 2858 (m), 1710 (s), 1464 (w), 1372(w), 1337 (m), 1251 (m), 1182 (s), 1033 (s), 959 (w), 918 (w), 804 (w), 729 (s).

9-(3-(Ethoxycarbonyl)cycloprop-1-en-1-yl)nonyl nicotinate (S25)

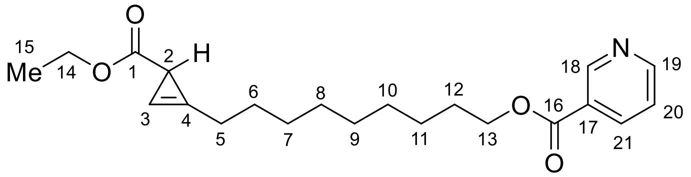

Oxalyl chloride (228.5 mg, 1.80 mmol, 1.8 eq.) was added slowly to a stirred suspension of nicotinic acid (147.7 mg, 1.20 mmol, 1.2 eq.) in dry DCM, (2.5 mL) containing 5 μL of DMF at room temperature under Ar atmosphere. The mixture was allowed to stir overnight at room temperature and then the volatile substances were removed under vacuum. The residue was dissolved in DCM (2.5 mL) followed by successive addition of ethyl 2-(9-hydroxynonyl)cycloprop-2-ene-1-carboxylate (**S24**) (254.4 mg, 1.00 mmol, 1.0 eq.) and triethylamine (182.1 mg, 1.80 mmol, 1.8 eq.). The mixture was stirred overnight at room temperature and then the volatile substances were removed under vacuum. The residue was purified by column chromatography (*n*-pentane:Et_2O 1:1) to yield the title compound as a yellow oil (327.2 mg, 0.91 mmol, 91%).

R_f = 0.18 (*n*-pentane:Et_2O 1:1).

^{1}H NMR (400 MHz, $CDCl_3$) δ 9.22 (s, 1H, H-C18), 8.77 (dt, $^3J_{HH}$ = 4.9 Hz, $^4J_{HH}$ = 1.6 Hz, 1H, H-C19), 8.29 (dt, $^3J_{HH}$ = 8.0 Hz, $^4J_{HH}$ = 1.9 Hz, 1H, H-C21), 7.39 (dd, $^3J_{HH}$ = 7.9, 4.8 Hz, 1H, H-C20), 6.32 (q, $^3J_{HH}$ = 1.4 Hz, 1H, H-C3), 4.35 (t, $^3J_{HH}$ = 6.7 Hz, 2H, H-C13), 4.21 – 4.03 (m, 2H, H-C14), 2.48 (t, $^3J_{HH}$ = 7.3 Hz, 2H, H-C5), 2.12 (d, $^3J_{HH}$ = 1.5 Hz, 1H, H-C2), 1.83 – 1.73 (m, 2H, H-C12), 1.62 – 1.52 (m, 2H, H-C6), 1.48 – 1.40 (m, 2H, H-C11), 1.39 – 1.28 (m, 8H, H-C7~C10), 1.24 (t, $^3J_{HH}$ = 7.1 Hz, 3H, H-C15).

^{13}**C NMR** (151 MHz, $CDCl_3$) δ 176.76 (C1), 165.48 (C16), 153.48 (C19), 151.06 (C18), 137.14 (C21), 126.50 (C17), 123.39 (C20), 115.74 (C4), 94.12 (C3), 65.70 (C13), 60.27 (C14), 29.50 (CH_2), 29.33 (CH_2), 29.33 (CH_2), 29.21 (CH_2), 28.77 (C12), 26.77 (C6), 26.10 (C11), 25.09 (C5), 19.86 (C2), 14.52 (C15).

ESI-MS: (*m/z*) requires: $[(C_{21}H_{29}NO_4Na)^+]$ = 382.1989, (*m/z*) found: $[(C_{21}H_{29}NO_4Na)^+]$ = 382.1995.

FT-IR ($\tilde{\nu}$ = cm^{-1}): 2927 (m), 2858 (w), 1722 (s), 1590 (w), 1464 (w), 1423 (w), 1366 (w), 1332 (w), 1280 (s), 1182 (s), 1113 (m), 1022 (m), 959 (w), 918 (w), 804 (w), 740 (s), 700 (m), 620 (w).

(1,2,2-Tribromo-3,3-dimethylcyclopropyl)benzene (Int 12)

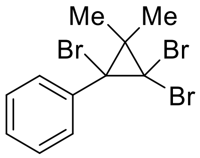

According to General Procedure **D**, the appropriate bromoalkene was first prepared using (2-methylprop-1-en-1-yl)benzene (1.98 g, 15.00 mmol, 1.0 eq.). Then, the title compound **Int 12** was synthesized using the corresponding bromoalkene (1.27 g, 6.00 mmol, 1.0 eq.). The crude residue was purified by column chromatography (*n*-pentane) to yield the title compound as a white solid (1.47 g, 3.83 mmol, 64%)

$\mathbf{R_f}$ = 0.70 (*n*-pentane).

^{1}**H NMR** (400 MHz, $CDCl_3$) δ 7.48 – 7.43 (m, 2H), 7.40 – 7.34 (m, 2H), 7.32 – 7.26 (m, 1H), 1.74 (s, 3H), 1.41 (s, 3H).

GC-EI-MS: (*m/z*) requires: $[(C_{11}H_{10}Br)^+]$ = 220.9960, (*m/z*) found: $[(C_{11}H_{10}Br)^+]$ = 220.9962.

Analytical data is in agreement with literature values.[4]

Methyl 3,3-dimethyl-2-phenylcycloprop-1-ene-1-carboxylate (S26)

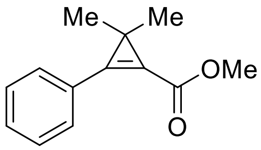

Compound **S26** was prepared according to General Procedure **E** using (1,2,2-tribromo-3,3-dimethylcyclopropyl)benzene (**Int 12**) (765.8 mg, 2.00 mmol, 1.0 eq.). The crude residue was purified by column chromatography (*n*-pentane:Et_2O 20:1-15:1) to yield the title compound as a yellow oil (111.2 mg, 0.55 mmol, 27%).

$\mathbf{R_f}$ = 0.20 (*n*-pentane:Et_2O 50:1).

^{1}H NMR (400 MHz, $CDCl_3$) δ 7.77 – 7.69 (m, 2H), 7.49 – 7.42 (m, 3H), 3.87 (s, 3H), 1.56 (s, 3H), 1.44 (s, 6H).

ESI-MS: (*m/z*) requires: $[(C_{13}H_{14}O_2Na)^+]$ = 225.0886, (*m/z*) found: $[(C_{13}H_{14}O_2Na)^+]$ = 225.0885.

Analytical data is in agreement with literature values.[4]

1-(2-Methylprop-1-en-1-yl)-4-(trifluoromethyl)benzene (Int 13)

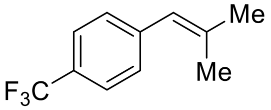

A three-neck flask was charged with isopropyltriphenylphosphonium iodide (10.81 g, 25.00 mmol, 1.0 eq.) and THF (80.0 mL) under Ar atmosphere. Potassium tert-butoxide (2.81 g, 25.00 mmol, 1.0 eq.) was added and the mixture was stirred vigorously at room temperature. After 1 h, 4-(trifluoromethyl)benzaldehyde (4.35 g, 25.00 mmol, 1.0 eq.) was added dropwise. The reaction was stirred at room temperature for 16 h and then quenched with a saturated aqueous solution of NH_4Cl. The layers were separated and the aqueous layer was extracted three times with Et_2O. The combined organic layers were washed with brine, dried over anhydrous Na_2SO_4, filtered, and concentrated under reduced pressure. The crude residue was purified by column chromatography (*n*-pentane) to yield the title compound as a colorless oil (2.98 g, 14.91 mmol, 60%).

$\mathbf{R}_f$ = 0.85 (*n*-pentane).

^{1}H NMR (400 MHz, $CDCl_3$) δ 7.55 (d, *J* = 8.1 Hz, 2H), 7.31 (d, *J* = 8.0 Hz, 2H), 6.28 (s, 1H), 1.93 (d, *J* = 1.5 Hz, 3H), 1.87 (d, *J* = 1.4 Hz, 3H).

^{19}F{^{1}H} NMR (377 MHz, $CDCl_3$) δ -62.34 (s, 3F).

GC-EI-MS: (*m/z*) requires: $[(C_{11}H_{11}F_3)^+]$ = 200.0807, (*m/z*) found: $[(C_{11}H_{11}F_3)^+]$ = 200.0803.

Analytical data is in agreement with literature values.[17]

1-(1,2,2-Tribromo-3,3-dimethylcyclopropyl)-4-(trifluoromethyl)benzene (Int 14)

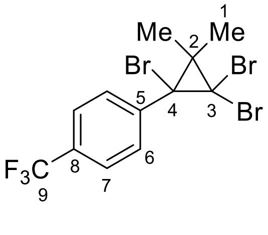

According to General Procedure **D**, the appropriate bromoalkene was first prepared using 1-(2-methylprop-1-en-1-yl)-4-(trifluoromethyl)benzene (**Int 13**) (2.90 g, 14.50 mmol, 1.0 eq.). Then, the title compound **Int 14** was synthesized using the corresponding bromoalkene (1.67 g, 6.00 mmol, 1.0 eq.). The crude residue was purified by column chromatography (*n*-pentane)

to yield the title compound as a white solid (0.88 g, 1.94 mmol, 32%)

R*f* = 0.70 (*n*-pentane).

^{1}H NMR (400 MHz, $CDCl_3$) δ 7.64 (d, $^{3}J_{HH}$ = 8.2 Hz, 2H, H-C7), 7.56 (d, $^{3}J_{HH}$ = 8.2 Hz, 2H, H-C6), 1.74 (s, 3H, H-C1), 1.40 (s, 3H, H-C1).

^{13}C NMR (151 MHz, $CDCl_3$) δ 143.14 (C5), 130.96 (C6), 130.33 (q, $^{2}J_{CF}$ = 32.8 Hz, C8), 125.54 (q, $^{3}J_{CF}$ = 3.8 Hz, C7), 123.97 (q, $^{1}J_{CF}$ = 272.5 Hz, C9), 52.00 (C4), 48.22 (*C*), 34.81 (*C*), 26.97(C1), 24.80 (C1).

^{19}F NMR (564 MHz, $CDCl_3$) δ -62.77 (s, 3F, F-C9).

^{19}F{^{1}H} NMR (377 MHz, $CDCl_3$) δ -62.77 (s, 3F, F-C9).

GC-EI-MS: (*m/z*) requires: $[(C_{12}H_{10}Br_2F_3)^+]$ = 370.9076, (*m/z*) found: $[(C_{12}H_{10}Br_2F_3)^+]$ = 370.9075.

FT-IR ($\tilde{\nu}$ = cm^{-1}): 2967 (w), 1619 (w), 1412 (w), 1320 (s), 1154 (s), 1108 (s), 1062 (s), 1022 (m), 861 (w), 815 (m), 786 (m), 763 (m), 740 (m), 672 (m), 608 (m), 522 (w).

Melting Point: 95-97 ˚C.

Methyl 3,3-dimethyl-2-(4-(trifluoromethyl)phenyl)cycloprop-1-ene-1-carboxylate (S27)

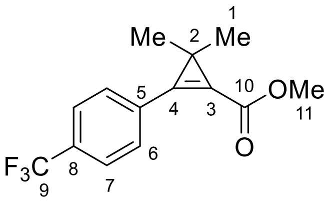

Compound **S27** was prepared according to General Procedure **E** using 1-(1,2,2-Tribromo-3,3-dimethylcyclopropyl)-4-(trifluoromethyl)benzene (**Int 14**) (811.7 mg, 1.80 mmol, 1.0 eq.) and methyl chloroformate (510.3 mg, 5.40 mmol, 3.0 eq.). The crude residue was purified by column chromatography (*n*-pentane:DCM 10:1-3:1) to yield the title compound as a yellow oil (55.6 mg, 0.21 mmol, 11%).

R*f* = 0.10 (*n*-pentane:DCM 10:1).

^{1}H NMR (400 MHz, $CDCl_3$) δ 7.82 (d, $^{3}J_{HH}$ = 8.0 Hz, 2H, H-C6), 7.70 (d, $^{3}J_{HH}$ = 8.0 Hz, 2H, H-C7), 3.89 (s, 3H, H-C11), 1.45 (s, 6H, H-C1).

^{13}C NMR (126 MHz, $CDCl_3$) δ 161.96 (C10), 141.09 (C4), 132.23 (app. q, $^{2}J_{CF}$ = 32.6 Hz, C8), 131.22 (C6), 130.87 (C5), 125.96 (q, $^{3}J_{CF}$ = 3.8 Hz, C7), 123.93 (q, $^{1}J_{CF}$ = 272.2 Hz, C9), 120.47 (C3), 52.24 (C11), 25.89 (C2), 25.28 (C1).

^{19}F NMR (470 MHz, $CDCl_3$) δ -62.96 (s, 3F, F-C9).

^{19}F{^{1}H} NMR (377 MHz, $CDCl_3$) δ -62.96 (s, 3F, F-C9).

ESI-MS: (*m/z*) requires: [($C_{14}H_{13}O_2F_3Na$)$^+$] = 293.0760, (*m/z*) found: [($C_{14}H_{13}O_2F_3Na$)$^+$] = 293.0759.

FT-IR ($\tilde{\nu}$ = cm^{-1}): 2950 (w), 2365 (w), 1819 (w), 1705 (m), 1613 (w), 1435 (w), 1412(w), 1320 (s), 1286 (m), 1200 (m), 1165 (s), 1125 (s), 1062 (s), 1016 (m), 849 (m), 763 (m), 735 (w), 717 (w), 608 (w).

N,N,3,3-tetramethyl-2-(4-(trifluoromethyl)phenyl)cycloprop-1-ene-1-carboxamide (S28)

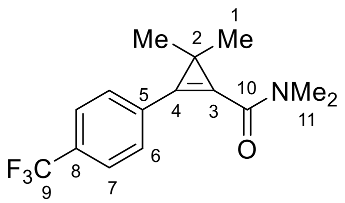

Compound **S28** was prepared according to General Procedure **E** using 1-(1,2,2-Tribromo-3,3-dimethylcyclopropyl)-4-(trifluoromethyl)benzene (**Int 14**) and dimethylcarbamoyl chloride. The crude residue was purified by column chromatography to yield the title compound as a yellow solid.

R*f* = 0.43 (*n*-pentane: Et_2O 1:1).

^{1}H NMR (500 MHz, $CDCl_3$) δ 7.99 – 7.89 (m, 2H, H-C6), 7.71 – 7.62 (m, 2H, H-C7), 3.21 – 3.19 (m, 3H, H-C11), 3.09 – 3.07 (m, 3H, H-C11), 1.46 – 1.43 (m, 6H, H-C1).

^{13}C NMR (126 MHz, $CDCl_3$) δ 161.60 (C10), 137.67 (C4), 131.58 (q, $^{2}J_{CF}$ = 32.6 Hz, C8), 131.17 (C6), 125.76 (q, $^{3}J_{CF}$ = 3.9 Hz, C7), 124.06 (q, $^{1}J_{CF}$ = 272.4 Hz, C9), 122.43 (C3), 37.06 (C11), 34.94 (C11), 27.16 (C2), 26.39 (C1).

^{19}F NMR (470 MHz, $CDCl_3$) δ -62.83 (m, 3F, F-C9).

^{19}F{^{1}H} NMR (470 MHz, $CDCl_3$) δ -62.83 (m, 3F, F-C9).

ESI-MS: (*m/z*) requires: [($C_{15}H_{16}NOF_3Na$)$^+$] = 306.1076, (*m/z*) found: [($C_{15}H_{16}NOF_3Na$)$^+$] = 306.1073.

FT-IR ($\tilde{\nu}$ = cm^{-1}): 2933 (w), 2864 (w), 2365 (w), 2336 (w), 1802 (w), 1619 (s), 1510 (w), 1487 (w), 1458 (m), 1395 (m), 1320 (s), 1274 (m), 1159 (m), 1108 (s), 1056 (s), 1016 (m), 918 (w), 867 (m), 838 (m), 821 (m), 758 (m), 729 (m), 672 (m), 608 (m), 574 (w).

Melting Point: 49-51 ˚C.

1-(Cyclohexylidenemethyl)-4-(trifluoromethyl)benzene (Int 15)

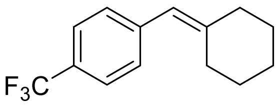

4-Trifluoromethylbenzylbromide (5.98 g, 25.00 mmol, 1.0 eq.) and triethyl phosphite (4.98 g, 30.00 mmol, 1.2 eq.) were added to a pressure tube under Ar atmosphere and heated to 100 °C for 12 h. After completion of the reaction, the mixture was passed through a silica plug (*n*-pentane:EA 1:2) to afford crude diethyl 4-(trifluoromethyl)benzylphosphonate, which was used without any further purification for the next step.

To a solution of NaH (60% in paraffin liquid, 1.28 g, 32.00 mmol, 1.6 eq.) in THF (50.0 mL) was added diethyl 4-(trifluoromethyl)benzylphosphonate (5.92 g, 20.00 mmol, 1.0 eq.) at 0 °C under Ar atmosphere. The resulting mixture was stirred at room temperature for 0.5 h. Cyclohexanone (1.96 g, 20.00 mmol, 1.0 eq.) was added at 0 °C. After stirring at room temperature overnight, the reaction was quenched with water, and then extracted three times with Et_2O. The combined organic layers were washed with brine, dried over anhydrous Na_2SO_4, filtered, and concentrated under reduced pressure. The crude residue was purified by column chromatography (*n*-pentane) to yield the title compound as a colorless oil (4.16 g, 17.31 mmol, 87%).

R*f* = 0.85 (*n*-pentane).

^{1}H NMR (400 MHz, $CDCl_3$) δ 7.54 (d, *J* = 8.1 Hz, 2H), 7.31 – 7.27 (m, 2H), 6.23 (s, 1H), 2.39 – 2.31 (m, 2H), 2.31 – 2.22 (m, 2H), 1.70 – 1.55 (m, 6H).

^{19}F{^{1}H} NMR (377 MHz, $CDCl_3$) δ -62.29 (s, 3F).

GC-EI-MS: (*m/z*) requires: $[(C_{14}H_{15}F_3)^+]$ = 240.1120, (*m/z*) found: $[(C_{14}H_{15}F_3)^+]$ = 240.1121. Analytical data is in agreement with literature values.[18]

1,1,2-Tribromo-2-(4-(trifluoromethyl)phenyl)spiro[2.5]octane (Int 16)

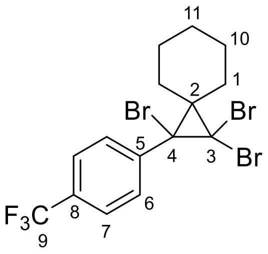

According to General Procedure **D**, the appropriate bromoalkene was first prepared using 1-(Cyclohexylidenemethyl)-4-(trifluoromethyl)benzene (**Int 15**) (3.60 g, 15.00 mmol, 1.0 eq.). Then, the title compound **Int 16** was synthesized using the corresponding bromoalkene (1.92 g, 6.00 mmol, 1.0 eq.). The crude residue was purified by column chromatography (*n*-pentane) to yield the title compound as a white solid (1.28 g, 2.60 mmol, 43%)

R*f* = 0.65 (*n*-pentane).

^{1}**H NMR** (599 MHz, $CDCl_3$) δ 7.63 (d, $^{3}J_{HH}$ = 8.1 Hz, 2H, H-C7), 7.57 (d, $^{3}J_{HH}$ = 8.1 Hz, 2H, H-C6), 2.18 – 2.10 (m, 1H, H-C10), 1.95 – 1.87 (m, 1H, H-C10), 1.87 – 1.78 (m, 3H, H-C1, H-C11), 1.75 – 1.65 (m, 2H, H-C1, H-C10), 1.61 – 1.53 (m, 1H, H-C10), 1.52 – 1.46 (m, 1H, H-C11), 1.45 – 1.36 (m, 1H, H-C1).

^{13}**C NMR** (151 MHz, $CDCl_3$) δ 142.90 (C5), 130.95 (C6), 130.29 (q, $^{2}J_{CF}$ = 32.7 Hz, C8), 125.49 (q, $^{3}J_{CF}$ = 3.8 Hz, C7), 123.98 (q, $^{1}J_{CF}$ = 272.4 Hz, C9), 53.07 (C4), 47.35 (C3), 38.56 (C2), 36.74 (C10), 35.20 (C11), 25.25 (C1), 24.81 (C1), 24.27 (C10).

^{19}**F NMR** (564 MHz, $CDCl_3$) δ -62.76 (s, 3F, F-C9).

^{19}**F{^{1}H} NMR** (377 MHz, $CDCl_3$) δ -62.76 (s, 3F, F-C9).

GC-EI-MS: (*m/z*) requires: $[(C_{15}H_{14}Br_2F_3)^+]$ = 410.9389, (*m/z*) found: $[(C_{15}H_{14}Br_2F_3)^+]$ = 410.9389.

FT-IR ($\tilde{\nu}$ = cm^{-1}): 2933 (w), 2858 (w), 1446 (w), 1412 (w), 1320 (s), 1154 (s), 1108 (s), 1068 (s), 1022 (m), 821 (m), 786 (m), 758 (m), 666 (w), 603 (m), 557 (w).

Melting Point: 124-126 ˚C.

Methyl 2-(4-(trifluoromethyl)phenyl)spiro[2.5]oct-1-ene-1-carboxylate (S29)

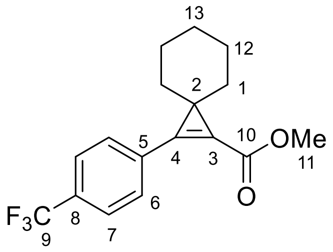

Compound **S29** was prepared according to General Procedure **E** using 1,1,2-Tribromo-2-(4-(trifluoromethyl)phenyl)spiro[2.5]octane (**Int 16**) (982.0 mg, 2.00 mmol, 1.0 eq.) and methyl chloroformate (567.0 mg, 6.00 mmol, 3.0 eq.). The crude residue was purified by column chromatography (*n*-pentane:DCM 5:1-4:1) to yield the title compound as a yellow wax (176.3 mg, 0.57 mmol, 28%).

$\mathbf{R_{\mathit{f}}}$ = 0.30 (*n*-pentane:DCM 4:1).

^{1}**H NMR** (500 MHz, $CDCl_3$) δ 7.88 (d, $^{3}J_{HH}$ = 7.8 Hz, 2H, H-C6), 7.70 (d, $^{3}J_{HH}$ = 7.7 Hz, 2H, H-C7), 3.89 (s, 3H, H-C11), 1.83 – 1.57 (m, 10H, H-C1, H-C12~C13).

^{13}**C NMR** (126 MHz, $CDCl_3$) δ 162.05 (C10), 142.22 (C4), 132.13 (app. q, $^{2}J_{CF}$ = 32.7 Hz, C8), 131.41 (C6), 131.15 (app. q, $^{5}J_{CF}$ = 1.5 Hz, C5), 125.92 (q, $^{3}J_{CF}$ = 3.8 Hz, C7), 123.94 (q, $^{1}J_{CF}$ = 272.7 Hz, C9), 121.12 (C3), 52.26 (C11), 36.96 ($\mathit{C}H_2$), 33.64 (C2), 27.41 ($\mathit{C}H_2$), 26.56 (C13).

^{19}F NMR (470 MHz, $CDCl_3$) δ -62.95 (s, 3F, F-C9).

^{19}F{^{1}H} NMR (470 MHz, $CDCl_3$) δ -62.95 (s, 3F, F-C9).

ESI-MS: (*m/z*) requires: [($C_{17}H_{17}O_2F_3Na)^+$] = 333.1084, (*m/z*) found: [($C_{17}H_{17}O_2F_3Na)^+$] = 333.1072.

FT-IR ($\tilde{\nu}$ = cm^{-1}): 2927 (m), 2853 (w), 2365 (w), 1808 (m), 1705 (m), 1613 (w), 1435 (w), 1412 (w), 1320 (s), 1280 (m), 1205 (m), 1165 (s), 1125 (s), 1062 (s), 1016 (m), 907 (w), 844 (m), 763 (m), 735 (m), 608 (w).

1.3 Preparation and characterisation of catalysts

Dimethyl 2,2'-((2-iodo-5-methyl-1,3-phenylene)bis(oxy))diacetate (C5)

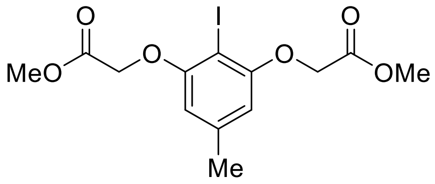

This compound was prepared according to a previous report from our research group.[19]

Dimethyl 2,2'-((2-iodo-1,3-phenylene)bis(oxy))diacetate (C6)

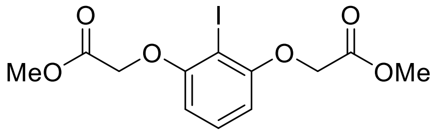

This compound was prepared according to a previous report from our research group.[19]

Dimethyl 2,2'-((2-iodo-5-(methoxycarbonyl)-1,3-phenylene)bis(oxy))diacetate (C7)

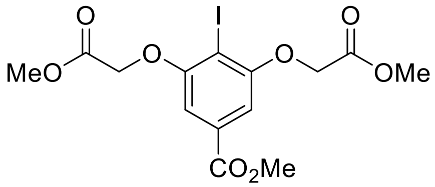

This compound was prepared according to a previous report from our research group.[19]

1.4 Preparation and characterisation of allyl difluorides

General Procedure F

Unless otherwise stated, a Teflon® vial was equipped with a 1 cm stirring bar followed by the addition of cyclopropene (0.10 mmol, 1.0 eq.), aryl iodide catalyst (0.02 mmol, 20 mol%) and ethyl

trifluoroacetate (0.25 mL). The stated amine:HF mixture was added (0.25 mL) via syringe. After stirring for 1 min, Selectfluor® (53.1 mg, 0.15 mmol, 1.5 eq.) was added in one portion. The reaction vessel was then sealed with a Teflon® screw cap. After stirring (350 rpm) at ambient temperature for 24 h, the reaction mixture was poured into 100 mL of a saturated solution of $NaHCO_3$ (CAUTION, generation of CO_2!). The Teflon® vial was rinsed with DCM and dropped into another flask of saturated aqueous solution of $NaHCO_3$ to guarantee the removal of excess HF. The organics were extracted with DCM (3x 30 mL), the combined organic layers were dried over Na_2SO_4, filtered and the solvent was carefully removed under reduced pressure. An internal standard (ethyl fluoroacetate) was added to the crude residue. The NMR yield and stereoselectivity ratio were analysed by ^{19}F NMR spectroscopy against the internal standard. The NMR sample was recombined with the crude residue and purification by column chromatography or preparative thin layer chromatography yielded the desired product.

(*E*)-1-(1,1,4,4,4-Pentafluoro-3-phenylbut-2-en-1-yl)-4-(trifluoromethyl)benzene (1)

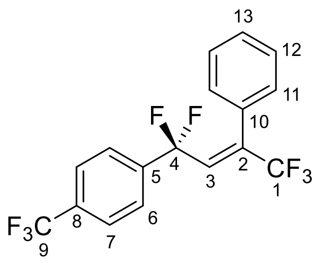

Compound **1** was prepared according to the General Procedure **F** with an amine:HF ratio of 1:7.0 using 1-(3-phenyl-3-(trifluoromethyl)cycloprop-1-en-1-yl)-4-(trifluoromethyl)benzene (**S1**) (32.8 mg, 0.10 mmol, 1.0 eq.) and aryl iodide **C7** (8.8 mg, 0.02 mmol, 20 mol%). After workup, the crude mixture was analysed by ^{19}F NMR (*E*:*Z* > 20:1). Purification by column chromatography (*n*-pentane) yielded the title compound **1** as a colorless oil (26.9 mg, 0.074 mmol, 74%).

$\mathbf{R}_f$ = 0.65 (*n*-pentane).

^{1}H NMR (500 MHz, $CDCl_3$) δ 7.51 (d, $^3J_{HH}$ = 8.3 Hz, 2H, H-C7), 7.36 – 7.31 (m, 1H, H-C13), 7.28 (d, $^3J_{HH}$ = 8.1 Hz, 2H, H-C6), 7.26 – 7.22 (m, 2H, H-C12), 7.00 (d, $^3J_{HH}$ = 7.2 Hz, 2H, H-C11), 6.80 (tq, $^3J_{HF}$ = 10.6 Hz, $^4J_{HF}$ = 1.5 Hz, 1H, H-C3).

^{13}C NMR (126 MHz, $CDCl_3$) δ 139.66 (app. t, $^2J_{CF}$ = 26.7 Hz, C5), 138.31 (C2), 132.31 (app. q, $^2J_{CF}$ = 32.9 Hz, C8), 130.65 (tq, $^2J_{CF}$ = 32.9 Hz, $^3J_{CF}$ = 5.6 Hz, C3), 129.65 (C10), 129.38 (t, $^5J_{CF}$ = 1.8 Hz, C11), 129.34 (C13), 128.12 (C12), 125.78 (t, $^3J_{CF}$ = 5.7 Hz, C6), 125.47 (q, $^3J_{CF}$ = 3.8 Hz, C7), 123.68 (q, $^1J_{CF}$ = 272.3 Hz, C9), 122.39 (q, $^1J_{CF}$ = 275.2 Hz, C1), 117.67 (t, $^1J_{CF}$ = 241.6 Hz, C4).

^{19}F NMR (470 MHz, $CDCl_3$) δ -63.11 (s, 3F, F-C9), -68.09 – -68.13 (m, 3F, F-C1), -86.72 (d, $^3J_{HF}$ = 11.1 Hz, 2F, F-C4).

^{19}F{^{1}H} NMR (377 MHz, $CDCl_3$) δ -63.11 (s, 3F, F-C9), -68.16 (s, 3F, F-C1), -86.79 (s, 2F, F-C4).

GC-EI-MS: (*m/z*) requires: [$(C_{17}H_{10}F_8)^+$] = 366.0649, (*m/z*) found: [$(C_{17}H_{10}F_8)^+$] = 366.0640.

FT-IR ($\tilde{\nu}$ = cm^{-1}): 1412 (w), 1326 (s), 1286 (m), 1263 (m), 1171 (s), 1125 (s), 1068 (s), 1010 (m), 844, 763 (w), 706 (m), 654 (m), 614 (m), 522 (w).

(*E*)-1-Bromo-4-(1,1,1,4,4-Pentafluoro-4-(4-(trifluoromethyl)phenyl)but-2-en-2-yl)benzene (2)

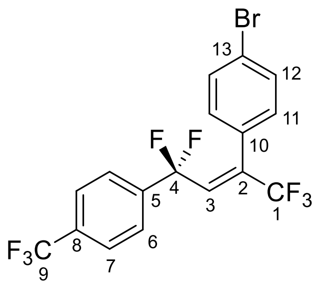

Compound **2** was prepared according to the General Procedure **F** with an amine:HF ratio of 1:8.0 using 1-bromo-4-(1-(trifluoromethyl)-2-(4-(trifluoromethyl)phenyl)cycloprop-2-en-1-yl)benzene (**S2**) (40.7 mg, 0.10 mmol, 1.0 eq.) and aryl iodide **C7** (8.8 mg, 0.02 mmol, 20 mol%). After workup, the crude mixture was analysed by ^{19}F NMR (*E*:*Z* > 20:1). Purification by column chromatography (*n*-pentane) yielded the title compound **2** as a white solid (32.6 mg, 0.073 mmol, 73%).

R_f = 0.55 (*n*-pentane).

^{1}H NMR (400 MHz, $CDCl_3$) δ 7.58 (d, $^3J_{HH}$ = 8.0 Hz, 2H, H-C7), 7.46 – 7.38 (m, 2H, H-C12), 7.32 (d, $^3J_{HH}$ = 7.7 Hz, 2H, H-C6), 6.90 (d, $^3J_{HH}$ = 8.3 Hz, 2H, H-C11), 6.79 (tq, $^3J_{HF}$ = 10.8 Hz, $^4J_{HF}$ = 1.5 Hz, 1H, H-C3).

^{13}C NMR (126 MHz, $CDCl_3$) δ 139.48 (C5), 137.03 (C2), 132.63 (C8), 131.48 (C12), 131.09 (tq, $^2J_{CF}$ = 32.7 Hz, $^3J_{CF}$ = 5.3 Hz, C3), 130.97 (t, $^5J_{CF}$ = 1.9 Hz, C11), 128.57 (C10), 125.76 (t, $^3J_{CF}$ = 5.7 Hz, C6), 125.68 (q, $^3J_{CF}$ = 3.8 Hz, C7), 124.05 (C13), 123.62 (q, $^1J_{CF}$ = 272.4 Hz, C9), 122.10 (q, $^1J_{CF}$ = 274.6 Hz, C1), 117.50 (t, $^1J_{CF}$ = 242.3 Hz, C4).

^{19}F NMR (470 MHz, $CDCl_3$) δ -63.08 (s, 3F, F-C9), -68.06 – -68.09 (m, 3F, F-C1), -87.30 (d, $^3J_{HF}$ = 10.2 Hz, 2F, F-C4).

^{19}F{^{1}H} NMR (377 MHz, $CDCl_3$) δ -63.08 (s, 3F, F-C9), -68.06 – -68.20 (m, 3F, F-C1), -87.39 (s, 2F, F-C4).

GC-EI-MS: (*m/z*) requires: [$(C_{17}H_9BrF_8)^+$] = 443.9754, (*m/z*) found: [$(C_{17}H_9BrF_8)^+$] = 443.9753.

FT-IR ($\tilde{\nu}$ = cm^{-1}): 1492 (w), 1412 (w), 1320 (m), 1286 (m), 1257 (m), 1211 (m), 1165 (s), 1136 (s), 1113 (s), 1062 (s), 1004 (s), 959 (w), 936 (w), 890 (m), 838 (m), 804 (m), 763 (m), 729 (m), 660 (m), 614 (m), 580 (w), 534 (w), 511 (w).

Melting Point: 64-66 ˚C.

(*E*)-1-Chloro-4-(1,1,1,4,4-pentafluoro-4-(4-(trifluoromethyl)phenyl)but-2-en-2-yl)benzene (3)

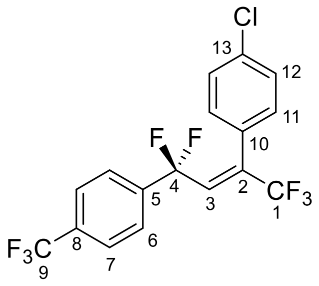

Compound **3** was prepared according to the General Procedure **F** with an amine:HF ratio of 1:8.0 using 1-chloro-4-(1-(trifluoromethyl)-2-(4-(trifluoromethyl)phenyl)cycloprop-2-en-1-yl)benzene (**S3**) (36.3 mg, 0.10 mmol, 1.0 eq.) and aryl iodide **C7** (8.8 mg, 0.02 mmol, 20 mol%). After workup, the crude mixture was analysed by ^{19}F NMR (*E*:*Z* > 20:1). Purification by column chromatography (*n*-pentane) yielded the title compound **3** as a white solid (28.8 mg, 0.072 mmol, 72%).

R*f* = 0.60 (*n*-pentane).

^{1}H NMR (400 MHz, $CDCl_3$) δ 7.59 (d, $^3J_{HH}$ = 8.2 Hz, 2H, H-C7), 7.33 (d, $^3J_{HH}$ = 8.1 Hz, 2H, H-C6), 7.29 – 7.23 (m, 2H, H-C12), 6.98 (d, $^3J_{HH}$ = 8.5 Hz, 2H, H-C11), 6.80 (tq, $^3J_{HF}$ = 10.7 Hz, $^4J_{HF}$ = 1.5 Hz, 1H, H-C3).

^{13}C NMR (126 MHz, $CDCl_3$) δ 139.50 (C5), 137.04 (C2), 135.87 (C13), 132.63 (C8), 131.13 (tq, $^2J_{CF}$ = 32.6 Hz, $^3J_{CF}$ = 5.4 Hz, C3), 130.74 (t, $^5J_{CF}$ = 1.9 Hz, C11), 128.51 (C12), 128.07 (C10), 125.76 (t, $^3J_{CF}$ = 5.7 Hz, C6), 125.67 (q, $^3J_{CF}$ = 3.8 Hz, C7), 123.62 (q, $^1J_{CF}$ = 272.7 Hz, C9), 122.18 (q, $^1J_{CF}$ = 275.0 Hz, C1), 117.50 (t, $^1J_{CF}$ = 242.7 Hz, C4).

^{19}F NMR (470 MHz, $CDCl_3$) δ -63.10 (s, 3F, F-C9), -68.06 – -68.13 (m, 3F, F-C1), -87.26 (d, $^3J_{HF}$ = 10.6 Hz, 2F, F-C4).

^{19}F{^{1}H} NMR (377 MHz, $CDCl_3$) δ -63.10 (s, 3F, F-C9), -68.12 – -68.18 (m, 3F, F-C1), -87.36 (s, 2F, F-C4).

GC-EI-MS: (*m/z*) requires: [$(C_{17}H_9ClF_8)^+$] = 400.0260, (*m/z*) found: [$(C_{17}H_9ClF_8)^+$] = 400.0258.

FT-IR ($\tilde{\nu}$ = cm^{-1}): 1492 (w), 1412 (w), 1320 (m), 1286 (m), 1257 (m), 1211 (m), 1165 (m), 1119 (s), 1062 (s), 1004 (m), 890 (w), 838 (m), 804 (w), 769 (m), 729 (w), 695 (w), 660 (m), 614 (m), 580 (w), 545 (w), 511 (w).

Melting Point: 59-60 ˚C.

(*E*)-4,4'-(1,1,4,4,4-Pentafluorobut-2-ene-1,3-diyl)bis((trifluoromethyl)benzene) (4)

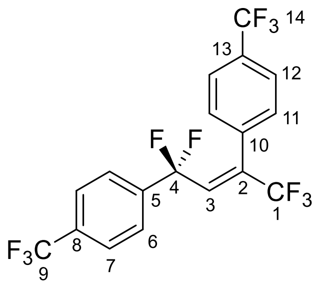

Compound **4** was prepared according to the General Procedure **F** with an amine:HF ratio of 1:9.2 using 4,4'-(1-(trifluoromethyl)cycloprop-2-ene-1,2-diyl)bis((trifluoromethyl)benzene) (**S4**) (39.6 mg, 0.10 mmol, 1.0 eq.) and aryl iodide **C7** (8.8 mg, 0.02 mmol, 20 mol%). After workup, the crude mixture was analysed by ^{19}F NMR (*E*:*Z* > 20:1). Purification by column chromatography (*n*-pentane) yielded the title compound **4** as a colorless oil (26.9 mg, 0.062 mmol, 62%).

R*f* = 0.50 (*n*-pentane).

^{1}H NMR (400 MHz, $CDCl_3$) δ 7.54 (m, 4H, H-C7, H-C12), 7.30 (d, $^{3}J_{HH}$ = 8.1 Hz, 2H, H-C6), 7.16 (d, $^{3}J_{HH}$ = 8.0 Hz, 2H, H-C11), 6.84 (tq, $^{3}J_{HF}$ = 10.6 Hz, $^{4}J_{HF}$ = 1.6 Hz, 1H, H-C3).

^{13}C NMR (151 MHz, $CDCl_3$) δ 139.26 (C5), 136.85 (C2), 133.42 (C10), 132.74 (C8), 131.73 (q, $^{2}J_{CF}$ = 32.8 Hz, C13), 131.49 (tq, $^{2}J_{CF}$ = 32.5 Hz, $^{3}J_{CF}$ = 5.4 Hz, C3), 129.92 (t, $^{5}J_{CF}$ = 1.8 Hz, C11), 125.74 (t, $^{3}J_{CF}$ = 5.7 Hz, C6), 125.70 (q, $^{3}J_{CF}$ = 3.8 Hz, C7), 125.15 (q, $^{3}J_{CF}$ = 3.7 Hz, C12), 123.77 (q, $^{1}J_{CF}$ = 272.3 Hz, C14), 123.54 (q, $^{1}J_{CF}$ = 272.9 Hz, C9), 122.08 (q, $^{1}J_{CF}$ = 275.0 Hz, C1), 117.39 (t, $^{1}J_{CF}$ = 242.9 Hz, C4).

^{19}F NMR (564 MHz, $CDCl_3$) δ -63.14 (s, 3F, F-C14), -63.23 (s, 3F, F-C9), -67.87 – -67.95 (m, 3F, F-C1), -87.32 (d, $^{3}J_{HF}$ = 10.4 Hz, 2F, F-C4).

^{19}F{^{1}H} NMR (377 MHz, $CDCl_3$) δ -63.14 (s, 3F, F-C14), -63.23 (s, 3F, F-C9), -67.92 – -68.02 (m, 3F, F-C1), -87.41 (s, 2F, F-C4).

ESI-MS: (*m/z*) requires: [$(C_{18}H_8F_{11})^+$] = 433.0456, (*m/z*) found: [$(C_{18}H_8F_{11})^+$] = 433.0451.

FT-IR ($\tilde{\nu}$ = cm^{-1}): 1412 (w), 1326 (s), 1286 (m), 1263 (m), 1171 (s), 1125 (s), 1068 (s), 1016 (m), 844 (s), 798 (w), 758 (w), 723 (w), 700 (w), 660 (m), 620 (m).

(*E*)-1-Methyl-4-(1,1,1,4,4-pentafluoro-4-(4-(trifluoromethyl)phenyl)but-2-en-2-yl)benzene (5)

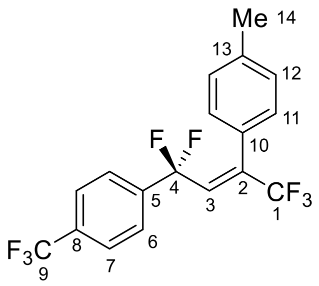

Compound **5** was prepared according to the General Procedure **F** with an amine:HF ratio of 1:7.5 using 1-methyl-4-(1-(trifluoromethyl)-2-(4-(trifluoromethyl)phenyl)cycloprop-2-en-1-yl)benzene (**S5**) (34.2 mg, 0.10 mmol, 1.0 eq.) and aryl iodide **C7** (8.8 mg, 0.02 mmol, 20 mol%). After workup, the crude mixture was analysed by ^{19}F NMR (*E*:*Z* > 20:1). Purification by column chromatography (*n*-pentane) yielded the title compound **5** as a colorless oil (25.4 mg, 0.067 mmol, 67%).

R*f* = 0.50 (*n*-pentane).

^{1}H NMR (400 MHz, $CDCl_3$) δ 7.51 (d, $^{3}J_{HH}$ = 8.1 Hz, 2H, H-C7), 7.29 (d, $^{3}J_{HH}$ = 8.1 Hz, 2H, H-C6), 7.04 (d, $^{3}J_{HH}$ = 8.0 Hz, 2H, H-C12), 6.88 (d, $^{3}J_{HH}$ = 8.1 Hz, 2H, H-C11), 6.76 (tq, $^{3}J_{HF}$ = 10.6 Hz, $^{4}J_{HF}$ = 1.6 Hz, 1H, H-C3), 2.33 (s, 3H, H-C14).

^{13}C NMR (126 MHz, $CDCl_3$) δ 139.71 (C5), 139.42 (C13), 138.37 (C2), 132.23 (C8), 130.43 (tq, $^{2}J_{CF}$ = 32.9 Hz, $^{3}J_{CF}$ = 5.4 Hz, C3), 129.26 (t, $^{5}J_{CF}$ = 1.9 Hz, C11), 128.80 (C12), 126.67 (C10), 125.86 (t, $^{3}J_{CF}$ = 5.7 Hz, C6), 125.38 (q, $^{3}J_{CF}$ = 3.7 Hz, C7), 123.72 (q, $^{1}J_{CF}$ = 272.5 Hz, C9), 122.47 (q, $^{1}J_{CF}$ = 274.7 Hz, C1), 117.74 (t, $^{1}J_{CF}$ = 241.9 Hz, C4), 21.34 (C12).

^{19}F NMR (470 MHz, $CDCl_3$) δ -63.10 (s, 3F, F-C9), -68.12 – -68.20 (m, 3F, F-C1), -86.62 (d, $^{3}J_{HF}$ = 10.8 Hz, 2F, F-C4).

^{19}F{^{1}H} NMR (377 MHz, $CDCl_3$) δ -63.10 (s, 3F, F-C9), -68.16 – -68.26 (m, 3F, F-C1), -86.70 (s, 2F, F-C4).

GC-EI-MS: (*m/z*) requires: [$(C_{18}H_{12}F_8)^+$] = 380.0806, (*m/z*) found: [$(C_{18}H_{12}F_8)^+$] = 380.0803.

FT-IR ($\tilde{\nu}$ = cm^{-1}): 2365 (w), 1515 (w), 1412 (w), 1326 (s), 1286 (m), 1263 (m), 1171 (s), 1125 (s), 1068 (s), 1010 (m), 844 (m), 804 (w), 729 (w), 712 (w), 643 (m), 614 (m), 568 (w), 511 (w).

(*E*)-1-(3-Benzyl-1,1,4,4,4-pentafluorobut-2-en-1-yl)-4-(trifluoromethyl)benzene (*E*-6)

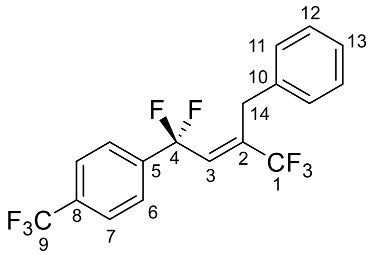

Compound ***E*-6** was prepared according to the General Procedure **F** with an amine:HF ratio of 1:7.0 using 1-(3-benzyl-3-(trifluoromethyl)cycloprop-1-en-1-yl)-4-(trifluoromethyl)benzene (**S6**) (34.2 mg, 0.10 mmol, 1.0 eq.) and aryl iodide **C7** (8.8 mg, 0.02 mmol, 20 mol%). After workup, the crude mixture was analysed by ^{19}F NMR (*E*:*Z* = 1:1.4). Purification by column chromatography (*n*-pentane) yielded the title compound ***E*-6** as a colorless oil (10.9 mg, 0.029 mmol, 29%).

R*f* = 0.35 (*n*-pentane).

^{1}H NMR (400 MHz, $CDCl_3$) δ 7.68 (d, $^{3}J_{HH}$ = 8.2 Hz, 2H, H-C7), 7.55 (d, $^{3}J_{HH}$ = 8.1 Hz, 2H, H-C6), 7.29 – 7.22 (m, 3H, H-C12, H-C13), 7.11 (d, $^{3}J_{HH}$ = 6.2 Hz, 2H, H-C11), 6.59 (t, $^{3}J_{HF}$ = 13.3 Hz, 1H, H-C3), 3.77 (s, 2H, H-C14).

^{13}C NMR (126 MHz, $CDCl_3$) δ 139.65 (C5), 136.16 (C2), 135.97 (C10), 132.92 (C8), 129.34 (tq, $^{2}J_{CF}$ = 30.7 Hz, $^{3}J_{CF}$ = 5.9 Hz, C3), 128.67 (C12), 128.57 (C11), 127.02 (C13), 126.08 (q, $^{3}J_{CF}$ = 3.8 Hz, C7), 125.80 (t, $^{3}J_{CF}$ = 5.6 Hz, C6), 123.66 (q, $^{1}J_{CF}$ = 272.8 Hz, C9), 123.32 (q, $^{1}J_{CF}$ = 275.8 Hz, C1), 118.26 (t, $^{1}J_{CF}$ = 242.7 Hz, C4), 32.19 (C14).

^{19}F NMR (470 MHz, $CDCl_3$) δ -63.06 (s, 3F, F-C9), -66.94 (q, $^{4}J_{HF}$ = 1.9 Hz, $^{5}J_{FF}$ = 1.9 Hz, 3F, F-C1), -88.78 (d, $^{3}J_{HF}$ = 13.1 Hz, 2F, F-C4).

^{19}F{^{1}H} NMR (377 MHz, $CDCl_3$) δ -63.06 (s, 3F, F-C9), -66.99 (t, $^{5}J_{FF}$ = 1.9 Hz, 3F, F-C1), -88.86 (s, 2F, F-C4).

GC-EI-MS: (*m/z*) requires: [$(C_{18}H_{12}F_8)^+$] = 380.0806, (*m/z*) found: [$(C_{18}H_{12}F_8)^+$] = 380.0804.

FT-IR ($\tilde{\nu}$ = cm^{-1}): 1412 (w), 1326 (s), 1263 (m), 1165 (s), 1131 (s), 1068 (s), 1004 (m), 941 (w), 844 (m), 763 (w), 735 (w), 700 (m), 614 (w), 568 (w).

(*Z*)-1-(3-benzyl-1,1,4,4,4-pentafluorobut-2-en-1-yl)-4-(trifluoromethyl)benzene (*Z*-6)

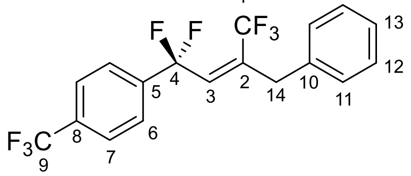

Compound **S1** was prepared according to the General Procedure **F** with an amine:HF ratio of 1:7.0 using 1-(3-benzyl-3-(trifluoromethyl)cycloprop-1-en-1-yl)-4-(trifluoromethyl)benzene (**S6**) (34.2 mg, 0.10 mmol, 1.0 eq.) and

aryl iodide **C7** (8.8 mg, 0.02 mmol, 20 mol%). After workup, the crude mixture was analysed by ^{19}F NMR (*E*:*Z* = 1:1.4). Purification by column chromatography (*n*-pentane) yielded the title compound ***Z*-6** as a colorless oil (14.8 mg, 0.039 mmol, 39%).

$\mathbf{R_f}$ = 0.25 (*n*-pentane).

^{1}H NMR (400 MHz, $CDCl_3$) δ 7.69 (d, $^3J_{HH}$ = 8.2 Hz, 2H, H-C7), 7.59 (d, $^3J_{HH}$ = 8.1 Hz, 2H, H-C6), 7.38 (t, $^3J_{HH}$ = 7.1 Hz, 2H, H-C12), 7.31 (t, $^3J_{HH}$ = 7.2 Hz, 1H, H-C13), 7.20 (d, $^3J_{HH}$ = 6.8 Hz, 2H, H-C11), 5.92 (t, $^3J_{HF}$ = 14.2 Hz, 1H, H-C3), 3.63 (q, $^4J_{HF}$ = 2.2 Hz, 2H, H-C14).

^{13}C NMR (126 MHz, $CDCl_3$) δ 140.12 (t, $^2J_{CF}$ = 28.4 Hz, C5), 137.43 (qt, $^2J_{CF}$ = 31.8 Hz, $^3J_{CF}$ = 6.7 Hz, C2), 135.46 (C10), 132.59 (q, $^2J_{CF}$ = 32.9 Hz, C8), 131.75 (tq, $^2J_{CF}$ = 34.0 Hz, $^3J_{CF}$ = 3.1 Hz, C3), 129.38 (C11), 129.20 (C12), 127.67 (C13), 125.89 (t, $^3J_{CF}$ = 5.3 Hz, C6), 125.81 (q, $^3J_{CF}$ = 3.7 Hz, C7), 123.76 (q, $^1J_{CF}$ = 272.8 Hz, C9), 122.31 (q, $^1J_{CF}$ = 276.7 Hz, C1), 117.41 (t, $^1J_{CF}$ = 240.9 Hz, C4), 38.46 (q, $^3J_{CF}$ = 2.8 Hz, C14).

^{19}F NMR (470 MHz, $CDCl_3$) δ -60.86 (t, $^5J_{FF}$ = 13.1 Hz, 3F, F-C1), -62.99 (s, 3F, F-C9), -85.53 (p, $^5J_{FF}$ = 13.2 Hz, $^3J_{HF}$ = 13.2 Hz, 2F, F-C4).

^{19}F{^{1}H} NMR (470 MHz, $CDCl_3$) δ -60.86 (t, $^5J_{FF}$ = 13.1 Hz, 3F, F-C1), -62.99 (t, $^7J_{FF}$ = 1.2 Hz, 3F, F-C9), -85.53 (qq, $^5J_{FF}$ = 13.1 Hz, $^7J_{FF}$ = 1.2 Hz, 2F, F-C4).

GC-EI-MS: (*m/z*) requires: [$(C_{18}H_{12}F_8)^+$] = 380.0806, (*m/z*) found: [$(C_{18}H_{12}F_8)^+$] = 380.0806.

FT-IR ($\tilde{\nu}$ = cm^{-1}): 1412 (w), 1389 (w), 1326 (s), 1240 (m), 1165 (s), 1131 (s), 1068 (s), 1033 (m), 1010 (m), 941 (w), 844 (m), 758 (w), 706 (m), 608 (w), 568 (w), 511 (w).

(*E*)-1-(3-(4-Bromophenyl)-1,1,4,4,4-pentafluorobut-2-en-1-yl)-3,5-dichlorobenzene (7)

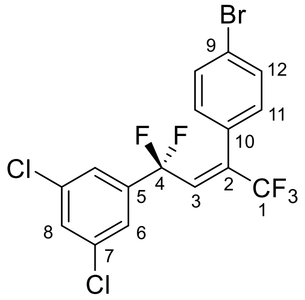

Compound **7** was prepared according to the General Procedure **F** with an amine:HF ratio of 1:8.0 using 1-(3-(4-bromophenyl)-3-(trifluoromethyl)cycloprop-1-en-1-yl)-3,5-dichlorobenzene (**S7**) (40.8 mg, 0.10 mmol, 1.0 eq.) and aryl iodide **C7** (8.8 mg, 0.02 mmol, 20 mol%). After workup, the crude mixture was analysed by ^{19}F NMR (*E*:*Z* > 20:1). Purification by column chromatography (*n*-pentane) yielded the title compound **7** as a white wax (26.6 mg, 0.060 mmol, 60%).

$\mathbf{R_f}$ = 0.75 (*n*-pentane).

^{1}H NMR (400 MHz, $CDCl_3$) δ 7.49 – 7.41 (m, 2H, H-C12), 7.35 (t, $^{4}J_{HH}$ = 1.9 Hz, 1H, H-C8), 6.99 (d, $^{4}J_{HH}$ = 1.9 Hz, 2H, H-C6), 6.90 (d, $^{3}J_{HH}$ = 8.4 Hz, 2H, H-C11), 6.75 (tq, $^{3}J_{HF}$ = 10.4 Hz, $^{4}J_{HF}$ = 1.5 Hz, 1H, H-C3).

^{13}C NMR (126 MHz, $CDCl_3$) δ 138.72 (t, $^{2}J_{CF}$ = 28.0 Hz, C5), 137.60 (C2), 135.56 (C7), 131.56 (C12), 130.95 (t, $^{5}J_{CF}$ = 1.7 Hz, C11), 130.92 (tq, $^{2}J_{CF}$ = 33.1 Hz, $^{3}J_{CF}$ = 5.4 Hz, C3), 130.45 (t, $^{5}J_{CF}$ = 1.5 Hz, C8), 128.23 (C10), 124.40 (C9), 124.01 (t, $^{3}J_{CF}$ = 5.8 Hz, C6), 122.02 (q, $^{1}J_{CF}$ = 274.9 Hz, C1), 116.77 (t, $^{1}J_{CF}$ = 243.4 Hz, C4).

^{19}F NMR (470 MHz, $CDCl_3$) δ -68.14 – -68.18 (m, 3F, F-C1), -86.53 (d, $^{3}J_{HF}$ = 10.4 Hz, 2F, F-C4).

^{19}F{^{1}H} NMR (470 MHz, $CDCl_3$) δ -68.16 (t, $^{5}J_{FF}$ = 2.1 Hz, 3F, F-C1), -86.53 (q, $^{5}J_{FF}$ = 2.1 Hz, 2F, F-C4).

GC-EI-MS: (*m/z*) requires: [($C_{16}H_8BrCl_2F_5)^+$] = 443.9101, (*m/z*) found: [($C_{16}H_8BrCl_2F_5)^+$] = 443.9097.

FT-IR ($\tilde{\nu}$ = cm^{-1}): 1573 (w), 1487 (w), 1423 (w), 1389 (w), 1257 (s), 1182 (s), 1125 (s), 1102 (s), 1073 (s), 1056 (s), 1010 (m), 953 (w), 930 (w), 884 (m), 867 (m), 821 (m), 798 (s), 763 (m), 712 (m), 677 (m), 660 (m), 626 (w), 505 (m).

Methyl (*E*)-4-(3-(4-bromophenyl)-1,1,4,4,4-pentafluorobut-2-en-1-yl)benzoate (8)

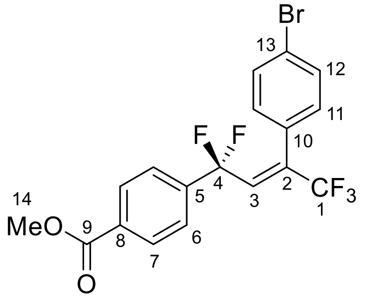

Compound **8** was prepared according to the General Procedure **F** with an amine:HF ratio of 1:8.0 using methyl 4-(3-(4-bromophenyl)-3-(trifluoromethyl)cycloprop-1-en-1-yl)benzoate (**S8**) (39.7 mg, 0.10 mmol, 1.0 eq.) and aryl iodide **C7** (8.8 mg, 0.02 mmol, 20 mol%). After workup, the crude mixture was analysed by ^{19}F NMR (*E*:*Z* > 20:1). Purification by column chromatography (*n*-pentane:Et_2O 30:1) yielded the title compound **8** as a white solid (32.1 mg, 0.074 mmol, 74%).

R*f* = 0.30 (*n*-pentane:Et_2O 30:1).

^{1}H NMR (400 MHz, $CDCl_3$) δ 7.99 (d, $^{3}J_{HH}$ = 8.0 Hz, 2H, H-C7), 7.47 – 7.38 (m, 2H, H-C12), 7.28 (d, $^{3}J_{HH}$ = 7.9 Hz, 2H, H-C6), 6.93 (d, $^{3}J_{HH}$ = 7.4 Hz, 2H, H-C11), 6.83 – 6.70 (m, 1H, H-C3), 3.96 – 3.92 (m, 3H, H-C14).

^{13}**C NMR** (126 MHz, $CDCl_3$) δ 166.19 (C9), 140.13 (t, $^2J_{CF}$ = 27.1 Hz, C5), 136.62 (C2), 132.13 (t, $^5J_{CF}$ = 1.7 Hz, C8), 131.45 (C12), 131.11 (tq, $^2J_{CF}$ = 32.4 Hz, $^3J_{CF}$ = 5.2 Hz, C3), 131.00 (t, $^5J_{CF}$ = 1.8 Hz, C11), 129.91 (C7), 128.67 (C10), 125.27 (t, $^3J_{CF}$ = 5.6 Hz, C6), 123.99 (C13), 122.16 (q, $^1J_{CF}$ = 275.0 Hz, C1), 117.71 (t, $^1J_{CF}$ = 242.7 Hz, C4), 52.58 (C14).

^{19}**F NMR** (470 MHz, $CDCl_3$) δ -67.96 – -68.02 (m, 3F, F-C1), -87.48 (d, $^3J_{HF}$ = 11.1 Hz, 2F, F-C4).

^{19}**F{^{1}H} NMR** (377 MHz, $CDCl_3$) δ -67.99 (s, 3F, F-C1), -87.51 (s, 2F, F-C4).

GC-EI-MS: (*m/z*) requires: [$(C_{18}H_{12}O_2BrF_5)^+$] = 433.9935, (*m/z*) found: [$(C_{18}H_{12}O_2BrF_5)^+$] = 433.9932.

FT-IR ($\tilde{\nu}$ = cm^{-1}): 1716 (m), 1584 (w), 1492 (w), 1458 (w), 1435(w), 1412 (w), 1274 (s), 1205 (m), 1177 (s), 1131 (s), 1108 (s), 1062 (s), 1010 (s), 970 (m), 861 (m), 827 (m), 763 (m), 729 (w), 706 (m), 666 (m), 597 (m), 534 (w).

Melting Point: 56-58 ˚C.

(*E*)-4-(3-(4-Bromophenyl)-1,1,4,4,4-pentafluorobut-2-en-1-yl)benzonitrile (9)

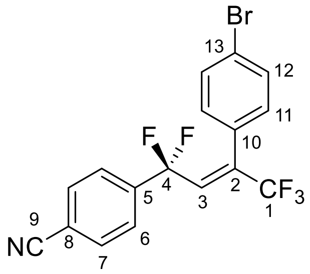

Compound **9** was prepared according to the General Procedure **F** with an amine:HF ratio of 1:9.2 using 4-(3-(4-bromophenyl)-3-(trifluoromethyl)cycloprop-1-en-1-yl)benzonitrile (**S9**) (36.4 mg, 0.10 mmol, 1.0 eq.) and aryl iodide **C7** (8.8 mg, 0.02 mmol, 20 mol%). After workup, the crude mixture was analysed by ^{19}F NMR (*E*:*Z* > 20:1). Purification by column chromatography (*n*-pentane:Et_2O 25:1-20:1) yielded the title compound **9** as a pale yellow solid (22.1 mg, 0.055 mmol, 55%).

R*f* = 0.25 (*n*-pentane:Et_2O 20:1).

^{1}**H NMR** (400 MHz, $CDCl_3$) δ 7.62 (d, $^3J_{HH}$ = 8.8 Hz, 2H, H-C7), 7.47 – 7.40 (m, 2H, H-C12), 7.30 (d, $^3J_{HH}$ = 8.9 Hz, 2H, H-C6), 6.91 (d, $^3J_{HH}$ = 8.4 Hz, 2H, H-C11), 6.77 (tq, $^3J_{HF}$ = 10.8 Hz, $^4J_{HF}$ = 1.5 Hz, 1H, H-C3).

^{13}**C NMR** (126 MHz, $CDCl_3$) δ 140.29 (t, $^2J_{CF}$ = 27.6 Hz, C5), 137.48 (C2), 132.45 (C7), 131.55 (C12), 130.94 (t, $^5J_{CF}$ = 1.8 Hz, C11), 130.75 (tq, $^2J_{CF}$ = 32.4 Hz, $^3J_{CF}$ = 5.3 Hz, C3), 128.41 (C10),

126.03 (t, $^3J_{CF}$ = 5.7 Hz, C6), 124.23 (C13), 122.01 (q, $^1J_{CF}$ = 274.9 Hz, C1), 117.81 (C9), 117.23 (t, $^1J_{CF}$ = 243.4 Hz, C4), 114.58 (t, $^5J_{CF}$ = 1.7 Hz, C8).

^{19}F NMR (470 MHz, $CDCl_3$) δ -68.02 – -68.13 (m, 3F, F-C1), -87.42 (d, $^3J_{HF}$ = 10.8 Hz, 2F, F-C4).

^{19}F{^{1}H} NMR (377 MHz, $CDCl_3$) δ -68.08 (t, $^5J_{FF}$ = 2.1 Hz, 3F, F-C1), -87.45 (q, $^5J_{FF}$ = 2.1 Hz, 2F, F-C4).

GC-EI-MS: (*m/z*) requires: [$(C_{17}H_9NBrF_5)^+$] = 400.9833, (*m/z*) found: [$(C_{17}H_9NBrF_5)^+$] = 400.9825.

FT-IR ($\tilde{\nu}$ = cm^{-1}): 2238 (w), 1487 (w), 1400 (w), 1286 (m), 1257 (m), 1182 (s), 1131 (s), 1073 (s), 1010 (s), 907 (w), 838 (m), 804 (w), 763 (m), 723 (m), 672 (m), 643 (w), 603 (m), 545 (w), 511 (w).

Melting Point: 83-85 ˚C.

(*E*)-(1,1,1,4,4-Pentafluorodec-2-en-2-yl)benzene (10)

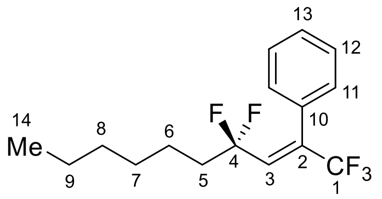

Compound **10** was prepared according to the General Procedure **F** with an amine:HF ratio of 1:8.0 using (2-hexyl-1-(trifluoromethyl)cycloprop-2-en-1-yl)benzene (**S10**) (26.8 mg, 0.10 mmol, 1.0 eq.) and iodobenzene **C2** (4.1 mg, 0.02 mmol, 20 mol%). The solvent was changed to DCM. After workup, the crude mixture was analysed by ^{19}F NMR (*E*:*Z* > 20:1). Purification by column chromatography (*n*-pentane) yielded the title compound **10** as a colorless oil (16.2 mg, 0.053 mmol, 53%).

R*f* = 0.80 (*n*-pentane).

^{1}H NMR (400 MHz, $CDCl_3$) δ 7.48 – 7.35 (m, 3H, H-C12, H-C13), 7.30 – 7.25 (m, 2H, H-C11), 6.45 (tq, $^3J_{HF}$ = 11.6 Hz, $^4J_{HF}$ = 1.7 Hz, 1H, H-C3), 1.78 – 1.60 (m, 2H, H-C5), 1.41 – 1.30 (m, 2H, H-C6), 1.29 – 1.13 (m, 6H, H-C7~C9), 0.86 (t, $^3J_{HH}$ = 7.1 Hz, 3H, H-C14).

^{13}C NMR (126 MHz, $CDCl_3$) δ 136.73 (C2), 130.41 (C10), 130.31 (tq, $^2J_{CF}$ = 30.0 Hz, $^3J_{CF}$ = 5.4 Hz, C3), 129.43 (t, $^5J_{CF}$ = 2.0 Hz, C11), 129.33 (C13), 128.21 (C12), 122.69 (q, $^1J_{CF}$ = 274.7 Hz, C1), 120.93 (t, $^1J_{CF}$ = 240.4 Hz, C4), 37.47 (t, $^2J_{CF}$ = 25.3 Hz, C5), 31.53 (CH_2), 28.89 (CH_2), 22.55 (CH_2), 22.04 (t, $^3J_{CF}$ = 3.9 Hz, C6), 14.12 (C14).

^{19}F NMR (470 MHz, $CDCl_3$) δ -67.74 – -67.79 (m, 3F, F-C1), -89.12 – -89.32 (m, 2F, F-C4).

^{19}F{^{1}H} NMR (470 MHz, $CDCl_3$) δ -67.76 (t, $^5J_{FF}$ = 2.3 Hz, 3F, F-C1), -89.21 (q, $^5J_{FF}$ = 2.3 Hz, 2F, F-C4).

GC-EI-MS: (*m/z*) requires: [$(C_{16}H_{19}F_5)^+$] = 306.1401, (*m/z*) found: [$(C_{16}H_{19}F_5)^+$] = 306.1399.

FT-IR ($\tilde{\nu}$ = cm^{-1}): 2962 (m), 2933 (m), 2359 (s), 2158 (w), 1653 (w), 1510 (m), 1286 (m), 1188 (s), 1045 (s), 666 (w).

(*E*)-(5-Cyclohexyl-1,1,1,4,4-pentafluoropent-2-en-2-yl)benzene (11)

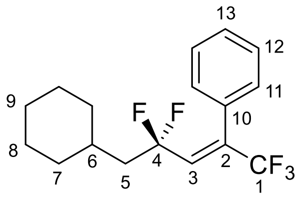

Compound **11** was prepared according to the General Procedure **F** with an amine:HF ratio of 1:7.0 using (2-(cyclohexylmethyl)-1-(trifluoromethyl)cycloprop-2-en-1-yl)benzene (**S11**) (28.3 mg, 0.10 mmol, 1.0 eq.) and iodobenzene **C2** (4.1 mg, 0.02 mmol, 20 mol%). After workup, the crude mixture was analysed by ^{19}F NMR (*E*:*Z* > 20:1). Purification by column chromatography (*n*-pentane) yielded the title compound **11** as a colorless oil (14.8 mg, 0.046 mmol, 46%).

R_f = 0.60 (*n*-pentane).

^{1}H NMR (400 MHz, $CDCl_3$) δ 7.46 – 7.36 (m, 3H, H-C12, H-C13), 7.29 – 7.25 (m, 2H, H-C11), 6.54 – 6.36 (m, 1H, H-C3), 1.72 – 1.57 (m, 7H), 1.54 – 1.45 (m, 1H, H-C6), 1.28 – 1.02 (m, 3H), 0.94 – 0.80 (m, 2H).

^{13}C NMR (126 MHz, $CDCl_3$) δ 136.48 (C2), 130.78 (tq, $^2J_{CF}$ = 30.0 Hz, $^3J_{CF}$ = 5.3 Hz, C3), 130.40 (C10), 129.49 (t, $^5J_{CF}$ = 2.0 Hz, C11), 129.30 (C13), 128.16 (C12), 122.72 (q, $^1J_{CF}$ = 275.0 Hz, C1), 121.15 (t, $^1J_{CF}$ = 241.3 Hz, C4), 44.69 (t, $^2J_{CF}$ = 24.2 Hz, C5), 33.89 (CH_2), 32.35 (t, $^3J_{CF}$ = 2.6 Hz, C6), 26.15 (CH_2), 26.13 (CH_2).

^{19}F NMR (470 MHz, $CDCl_3$) δ -67.69 – -67.75 (m, 3F, F-C1), -86.28 – -86.49 (m, 2F, F-C4).

^{19}F{^{1}H} NMR (470 MHz, $CDCl_3$) δ -67.72 (t, $^5J_{FF}$ = 2.3 Hz, 3F, F-C1), -86.38 (q, $^5J_{FF}$ = 2.3 Hz, 2F, F-C4).

GC-EI-MS: (*m/z*) requires: [$(C_{17}H_{19}F_5)^+$] = 318.1401, (*m/z*) found: [$(C_{17}H_{19}F_5)^+$] = 318.1401.

FT-IR ($\tilde{\nu}$ = cm^{-1}): 2927 (m), 2853 (w), 2365 (w), 1452 (w), 1286 (m), 1177 (s), 1131 (s), 1079 (m, 1056 (m), 1016 (s), 821 (w), 775 (w), 706 (s), 654 (m), 620 (w), 517 (w).

(*E*)-10,10,13,13,13-Pentafluoro-12-phenyltridec-11-en-1-yl cyclopropanecarboxylate (12)

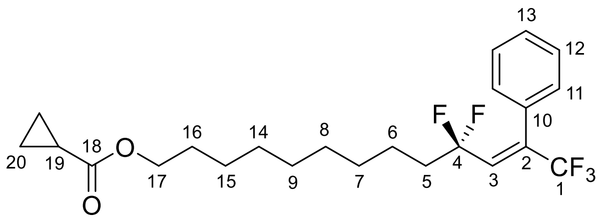

Compound **12** was prepared according to the General Procedure **F** with an amine:HF ratio of 1:7.0 using 9-(3-phenyl-3-(trifluoromethyl)cycloprop-1-en-1-yl)nonyl cyclopropanecarboxylate (**S12**) (19.7 mg, 0.05 mmol, 1.0 eq.) and iodobenzene **C2** (2.0 mg, 0.01 mmol, 20 mol%). The solvent was changed to DCM. After workup, the crude mixture was analysed by ^{19}F NMR (*E*:*Z* > 20:1). Purification by column chromatography (*n*-pentane:Et_2O 30:1) yielded the title compound **12** as a colorless oil (10.9 mg, 0.025 mmol, 50%).

R*f* = 0.20 (*n*-pentane:Et_2O 30:1).

^{1}H NMR (400 MHz, $CDCl_3$) δ 7.45 – 7.36 (m, 3H, H-C12, H-C13), 7.30 – 7.25 (m, 2H, H-C11), 6.45 (tq, $^3J_{HF}$ = 11.6 Hz, $^4J_{HF}$ = 1.6 Hz, 1H, H-C3), 4.05 (t, $^3J_{HH}$ = 6.7 Hz, 2H, H-C17), 1.76 – 1.57 (m, 5H, H-C5, H-C16, H-C19), 1.40 – 1.15 (m, 12H, H-C6~C9, H-C14~C15), 1.01 – 0.95 (m, 2H, H-C20), 0.88 – 0.81 (m, 2H, H-C20).

^{13}C NMR (126 MHz, $CDCl_3$) δ 175.13 (C18), 136.75 (C2), 130.40 (C10), 130.29 (tq, $^2J_{CF}$ = 29.8 Hz, $^3J_{CF}$ = 5.4 Hz, C3), 129.42 (t, $^5J_{CF}$ = 1.9 Hz, C11), 129.33 (C13), 128.21 (C12), 122.68 (q, $^1J_{CF}$ = 274.9 Hz, C1), 120.90 (t, $^1J_{CF}$ = 240.5 Hz, C4), 64.71 (C17), 37.45 (t, $^2J_{CF}$ = 25.3 Hz, C5), 29.38 (CH_2), 29.29 (CH_2), 29.24 (CH_2), 29.18 (CH_2), 28.80 (C16), 26.00 (CH_2), 22.05 (t, $^3J_{CF}$ = 4.0 Hz, C6), 13.05 (C19), 8.42 (C20).

^{19}F NMR (470 MHz, $CDCl_3$) δ -67.70 – -67.81 (m, 3F, F-C1), -89.13 – -89.35 (m, 2F, F-C4).

^{19}F{^{1}H} NMR (470 MHz, $CDCl_3$) δ -67.76 (t, $^5J_{FF}$ = 2.3 Hz, 3F, F-C1), -89.26 (q, $^5J_{FF}$ = 2.3 Hz, 2F, F-C4).

ESI-MS: (*m/z*) requires: [($C_{23}H_{29}O_2F_5Na$)$^+$] = 455.1980, (*m/z*) found: [($C_{23}H_{29}O_2F_5Na$)$^+$] = 455.1972.

FT-IR ($\tilde{\nu}$ = cm^{-1}): 2933 (m), 2858 (w), 1728 (s), 1458 (w), 1400 (m), 1372 (m), 1286 (m), 1171 (s), 1131 (s), 1073 (m), 1027 (m), 941 (w), 895 (w), 827 (m), 775 (w), 746 (w), 706 (s), 649 (w), 620 (w), 562 (w), 522 (w).

Diethyl 2-(2,2-difluoro-2-(4-(trifluoromethyl)phenyl)ethylidene)malonate (13)

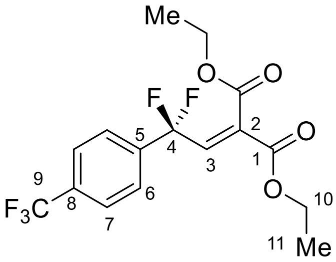

Compound **13** was prepared according to the General Procedure **F** with an amine:HF ratio of 1:6.5 using diethyl 2-(4-(trifluoromethyl)phenyl)cycloprop-2-ene-1,1-dicarboxylate (**S13**) (65.7 mg, 0.20 mmol, 1.0 eq.) and aryl iodide **C7** (17.5 mg, 0.04 mmol, 20 mol%). The crude product was purified by column chromatography (*n*-pentane:Et_2O 15:1) yielded the title compound **13** as a colorless oil (43.6 mg, 0.12 mmol, 60%).

R*f* = 0.40 (*n*-pentane:Et_2O 15:1).

^{1}H NMR (400 MHz, $CDCl_3$) δ 7.72 (s, 4H, H-C6, H-C7), 6.87 (t, $^{3}J_{HF}$ = 13.2 Hz, 1H, H-C3), 4.35 (q, $^{3}J_{HH}$ = 7.1 Hz, 2H, H-C10), 4.28 (q, $^{3}J_{HH}$ = 7.2 Hz, 2H, H-C10), 1.44 – 1.22 (m, 6H, H-C11).

^{13}C NMR (126 MHz, $CDCl_3$) δ 164.20 (C1), 162.39 (C1), 138.28 (t, $^{2}J_{CF}$ = 27.5 Hz, C5), 135.29 (t, $^{2}J_{CF}$ = 30.6 Hz, C3), 133.05 (q, $^{2}J_{CF}$ = 32.5 Hz, C8), 131.45 (t, $^{3}J_{CF}$ = 5.3 Hz, C2), 126.20 (t, $^{3}J_{CF}$ = 5.6 Hz, C6), 126.01 (q, $^{3}J_{CF}$ = 3.7 Hz, C7), 123.69 (q, $^{1}J_{CF}$ = 272.8 Hz, C9), 117.72 (t, $^{1}J_{CF}$ = 243.2 Hz, C4), 62.71 (C10), 62.35 (C10), 14.09 (C11), 14.04 (C11).

^{19}F NMR (470 MHz, $CDCl_3$) δ -63.08 (s, 3F, F-C9), -94.58 (d, $^{3}J_{HF}$ = 11.9 Hz, 2F, F-C4).

^{19}F{^{1}H} NMR (377 MHz, $CDCl_3$) δ -63.08 (s, 3F, F-C9), -94.74 (s, 2F, F-C4).

ESI-MS: (*m/z*) requires: $[(C_{16}H_{15}O_4F_5Na)^+]$ = 389.0783, (*m/z*) found: $[(C_{16}H_{15}O_4F_5Na)^+]$ = 389.0778.

FT-IR ($\tilde{\nu}$ = cm^{-1}): 2990 (w), 1733 (m), 1412 (w), 1372 (w), 1326 (s), 1246 (s), 1223 (s), 1171 (s), 1131 (s), 1085 (s), 1068 (s), 1045 (m), 999 (s), 844 (m), 763 (w), 672 (w), 614 (m), 568 (w).

Diethyl 2-(2-(3,5-bis(trifluoromethyl)phenyl)-2,2-difluoroethylidene)malonate (14)

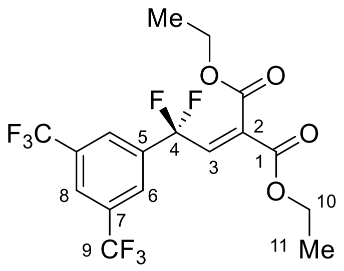

Compound **14** was prepared according to the General Procedure **F** with an amine:HF ratio of 1:8.5 using diethyl 2-(3,5-bis(trifluoromethyl)phenyl)cycloprop-2-ene-1,1-dicarboxylate (**S14**) (79.3 mg, 0.20 mmol, 1.0 eq.) and aryl iodide **C7** (17.5 mg, 0.04 mmol, 20 mol%). The crude product was purified by column chromatography (*n*-pentane:Et_2O 20:1) yielded the title compound **14** as a colorless oil (47.4 mg, 0.11 mmol, 55%).

R_f = 0.40 (*n*-pentane:Et_2O 20:1).

^{1}H NMR (400 MHz, $CDCl_3$) δ 8.07 (s, 2H, H-C6), 8.01 (s, 1H, H-C8), 6.87 (t, $^3J_{HF}$ = 13.2 Hz, 1H, H-C3), 4.36 (q, $^3J_{HH}$ = 7.1 Hz, 2H, H-C10), 4.30 (q, $^3J_{HH}$ = 7.1 Hz, 2H, H-C10), 1.38 – 1.33 (m, 3H, H-C11), 1.33 – 1.28 (m, 3H, H-C11).

^{13}C NMR (126 MHz, $CDCl_3$) δ 163.86 (C1), 162.15 (C1), 137.31 (t, $^2J_{CF}$ = 28.7 Hz, C5), 134.36 (t, $^2J_{CF}$ = 30.4 Hz, C3), 132.77 (q, $^2J_{CF}$ = 34.2 Hz, C7), 132.32 (t, $^3J_{CF}$ = 5.4 Hz, C2), 126.20 (C6), 124.93 (C8), 122.89 (q, $^1J_{CF}$ = 273.4 Hz, C9), 117.10 (t, $^1J_{CF}$ = 244.3 Hz, C4), 62.91 (C10), 62.56 (C10), 14.08 (C11), 13.96 (C11).

^{19}F NMR (470 MHz, $CDCl_3$) δ -63.02 (s, 6F, F-C9), -94.14 (d, $^3J_{HF}$ = 13.2 Hz, 2F, F-C4).

^{19}F{^{1}H} NMR (377 MHz, $CDCl_3$) δ -63.02 (s, 6F, F-C9), -94.28 (s, 2F, F-C4).

ESI-MS: (*m/z*) requires: [$(C_{17}H_{14}O_4F_8Na)^+$] = 457.0657, (*m/z*) found: [$(C_{17}H_{14}O_4F_8Na)^+$] = 457.0656.

FT-IR ($\tilde{\nu}$ = cm^{-1}): 2990 (w), 1733 (m), 1469 (w), 1378 (w), 1343 (w), 1280 (s), 1251 (s), 1223 (s), 1177 (s), 1131 (s), 1091 (s), 1056 (m), 1039 (m), 1022 (m), 907 (m), 867 (w), 844 (w), 706 (m), 677 (m), 631 (w), 562 (w).

Diethyl 2-(2,2-difluoro-2-(4-nitrophenyl)ethylidene)malonate (15)

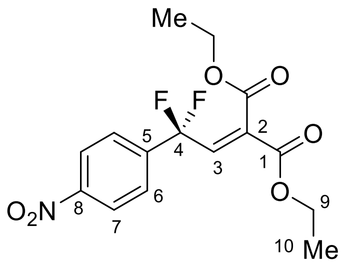

Compound **15** was prepared according to the General Procedure **F** with an amine:HF ratio of 1:8.5 using diethyl 2-(4-nitrophenyl)cycloprop-2-ene-1,1-dicarboxylate (**S15**) (61.1 mg, 0.20 mmol, 1.0 eq.) and aryl iodide **C7** (17.5 mg, 0.04 mmol, 20 mol%). The crude product was purified by column chromatography (*n*-pentane:Et_2O 7:1) yielded the title compound **15** as a colorless oil (37.9 mg, 0.11 mmol, 55%).

$\mathbf{R_f}$ = 0.30 (*n*-pentane:Et_2O 7:1).

^{1}H NMR (400 MHz, $CDCl_3$) δ 8.31 (d, $^3J_{HH}$ = 8.6 Hz, 2H, H-C7), 7.79 (d, $^3J_{HH}$ = 8.7 Hz, 2H, H-C6), 6.86 (t, $^3J_{HF}$ = 13.2 Hz, 1H, H-C3), 4.36 (q, $^3J_{HH}$ = 7.1 Hz, 2H, H-C9), 4.28 (q, $^3J_{HH}$ = 7.1 Hz, 2H, H-C9), 1.35 (t, $^3J_{HH}$ = 7.1 Hz, 3H, H-C10), 1.30 (t, $^3J_{HH}$ = 7.1 Hz, 3H, H-C10).

^{13}C NMR (126 MHz, $CDCl_3$) δ 164.10 (C1), 162.22 (C1), 149.47 (C8), 140.70 (t, $^2J_{CF}$ = 27.8 Hz, C5), 134.69 (t, $^2J_{CF}$ = 30.1 Hz, C3), 131.92 (t, $^3J_{CF}$ = 5.3 Hz, C2), 127.00 (t, $^3J_{CF}$ = 5.7 Hz, C6), 124.18 (C7), 117.47 (t, $^1J_{CF}$ = 243.7 Hz, C4), 62.81 (C9), 62.46 (C9), 14.08 (C10), 14.06 (C10).

^{19}F NMR (470 MHz, $CDCl_3$) δ -94.62 (d, $^3J_{HF}$ = 13.2 Hz, 2F, F-C4).

^{19}F{^{1}H} NMR (377 MHz, $CDCl_3$) δ -94.62 (s, 2F, F-C4).

ESI-MS: (*m/z*) requires: [$(C_{15}H_{15}NO_6F_2Na)^+$] = 366.0760, (*m/z*) found: [$(C_{15}H_{15}NO_6F_2Na)^+$] = 366.0759.

FT-IR ($\tilde{\nu}$ = cm^{-1}): 2985 (w), 2365 (w), 1733 (s), 1532 (m), 1349 (m), 1251 (s), 1223 (s), 1085 (s), 1045 (m), 1004 (m), 855 (m), 752 (w), 695 (w), 585 (w).

Diethyl 2-(2-(4-bromophenyl)-2,2-difluoroethylidene)malonate (16)

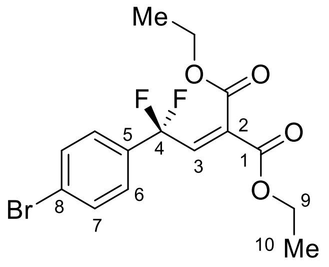

Compound **16** was was prepared according to the General Procedure **F** with an amine:HF ratio of 1:5.5 using diethyl 2-(4-bromophenyl)cycloprop-2-ene-1,1-dicarboxylate (**S16**) (67.8 mg, 0.20 mmol, 1.0 eq.) and aryl iodide **C7** (17.5 mg, 0.04 mmol, 20 mol%). The crude product was purified by column chromatography (*n*-pentane:Et_2O 10:1) yielded the title compound **16** as a colorless oil (29.8 mg, 0.08 mmol, 40%).

$\mathbf{R_f}$ = 0.35 (*n*-pentane:Et_2O 10:1).

^{1}H NMR (400 MHz, $CDCl_3$) δ 7.59 (d, $^{3}J_{HH}$ = 8.9 Hz, 2H, H-C7), 7.45 (d, $^{3}J_{HH}$ = 8.9 Hz, 2H, H-C6), 6.85 (t, $^{3}J_{HF}$ = 13.1 Hz, 1H, H-C3), 4.33 (q, $^{3}J_{HH}$ = 7.2 Hz, 2H, H-C9), 4.27 (q, $^{3}J_{HH}$ = 7.1 Hz, 2H, H-C9), 1.37 – 1.31 (m, 3H, H-C10), 1.31 – 1.27 (m, 3H, H-C10).

^{13}C NMR (126 MHz, $CDCl_3$) δ 164.27 (C1), 162.50 (C1), 135.64 (t, $^{2}J_{CF}$ = 30.9 Hz, C3), 133.70 (t, $^{2}J_{CF}$ = 27.6 Hz, C5), 132.19 (C7), 130.98 (t, $^{3}J_{CF}$ = 5.3 Hz, C2), 127.28 (t, $^{3}J_{CF}$ = 5.6 Hz, C6), 125.51 (C8), 117.98 (t, $^{1}J_{CF}$ = 242.8 Hz, C4), 62.63 (C9), 62.29 (C9), 14.10 (C10), 14.05 (C10).

^{19}F NMR (470 MHz, $CDCl_3$) δ -93.97 (d, $^{3}J_{HF}$ = 13.0 Hz, 2F, F-C4).

^{19}F{^{1}H} NMR (470 MHz, $CDCl_3$) δ -93.97 (s, 2F, F-C4).

ESI-MS: (*m/z*) requires: [$(C_{15}H_{15}O_4BrF_2Na)^+$] = 399.0014, (*m/z*) found: [$(C_{15}H_{15}O_4BrF_2Na)^+$] = 399.0015.

FT-IR ($\tilde{\nu}$ = cm^{-1}): 2985 (w), 1733 (s), 1670 (w), 1596 (w), 1395 (w), 1372 (m), 1343 (m), 1246 (s), 1217 (s), 1188 (s), 1079 (s), 1039 (m), 999 (s), 867 (m), 827 (m), 712 (w), 677 (w), 585 (m).

Dimethyl 2-(2,2-difluoro-2-(4-(trifluoromethyl)phenyl)ethylidene)malonate (17)

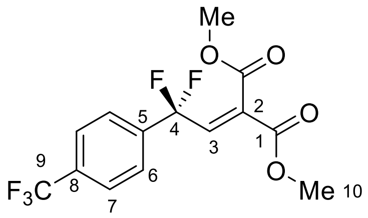

Compound **17** was was prepared according to the General Procedure **F** with an amine:HF ratio of 1:6.5 using dimethyl 2-(4-(trifluoromethyl)phenyl)cycloprop-2-ene-1,1-dicarboxylate (**S17**) (60.1 mg, 0.20 mmol, 1.0 eq.) and aryl iodide **C7** (17.5 mg, 0.04 mmol, 20 mol%). The crude product was purified by column chromatography (*n*-pentane:Et_2O 8:1) yielded the title compound **17** as a white solid (38.6 mg, 0.11 mmol, 57%).

R*f* = 0.40 (*n*-pentane:Et_2O 8:1).

^{1}H NMR (400 MHz, $CDCl_3$) δ 7.80 – 7.63 (m, 4H, H-C6, H-C7), 6.91 (t, $^{3}J_{HF}$ = 12.9 Hz, 1H, H-C3), 3.87 (s, 3H, H-C10), 3.83 (s, 3H, H-C10).

^{13}C NMR (126 MHz, $CDCl_3$) δ 164.61 (C1), 162.77 (C1), 138.12 (t, $^{2}J_{CF}$ = 27.8 Hz, C5), 136.03 (t, $^{2}J_{CF}$ = 30.5 Hz, C3), 133.11 (q, $^{2}J_{CF}$ = 33.0 Hz, C8), 130.72 (t, $^{3}J_{CF}$ = 5.4 Hz, C2), 126.16 (t, $^{3}J_{CF}$ = 5.7 Hz, C6), 126.06 (q, $^{3}J_{CF}$ = 3.7 Hz, C7), 123.65 (q, $^{1}J_{CF}$ = 272.7 Hz, C9), 117.57 (t, $^{1}J_{CF}$ = 243.7 Hz, C4), 53.44 (C10), 53.12 (C10).

^{19}F NMR (470 MHz, $CDCl_3$) δ -63.09 (s, 3F, F-C9), -95.06 (d, $^3J_{HF}$ = 13.0 Hz, 2F, F-C4).

^{19}F{^{1}H} NMR (377 MHz, $CDCl_3$) δ -63.09 (s, 3F, F-C9), -95.18 (s, 2F, F-C4).

ESI-MS: (*m/z*) requires: $[(C_{14}H_{11}O_4F_5Na)^+]$ = 361.0470, (*m/z*) found: $[(C_{14}H_{11}O_4F_5Na)^+]$ = 361.0469.

FT-IR ($\tilde{\nu}$ = cm^{-1}): 1751 (m), 1728 (s), 1441 (w), 1418 (w), 1355 (w), 1320 (s), 1251 (s), 1223 (s), 1200 (m), 1165 (s), 1113 (s), 1085 (s), 1062 (s), 1045 (s), 1004 (m), 970 (m), 930 (m), 844 (m), 763 (m), 712 (m), 672 (w), 631 (w), 603 (m), 568 (w).

Melting Point: 63-65 ˚C.

Ethyl (*Z*)-4,4-difluoro-2-methyl-4-(4-(trifluoromethyl)phenyl)but-2-enoate (18)

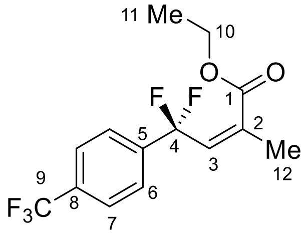

Compound **18** was prepared according to the General Procedure **F** with an amine:HF ratio of 1:4.5 using ethyl 1-methyl-2-(4-(trifluoromethyl)phenyl)cycloprop-2-ene-1-carboxylate (**S18**) (27.3 mg, 0.10 mmol, 1.0 eq.) and *p*-iodotoluene **C1** (4.4 mg, 0.02 mmol, 20 mol%). The solvent was changed to DCM. After workup, the crude mixture was analysed by ^{19}F NMR (*Z*:*E* > 20:1). Purification by column chromatography (*n*-pentane:Et_2O 30:1) yielded the title compound **18** as a colorless oil (17.2 mg, 0.056 mmol, 56%).

R_f = 0.30 (*n*-pentane:Et_2O 30:1).

^{1}H NMR (400 MHz, $CDCl_3$) δ 7.75 – 7.65 (m, 4H, H-C6, H-C7), 5.79 (tq, $^3J_{HF}$ = 13.1 Hz, $^4J_{HH}$ = 1.7 Hz, 1H, H-C3), 4.15 (q, $^3J_{HH}$ = 7.2 Hz, 2H, H-C10), 2.08 – 2.01 (m, 3H, C12), 1.22 (t, $^3J_{HH}$ = 7.1 Hz, 3H, H-C11).

^{13}C NMR (126 MHz, $CDCl_3$) δ 168.32 (C1), 140.29 (C5), 136.62 (t, $^3J_{CF}$ = 6.9 Hz, C2), 132.32 (C8), 126.24 (t, $^3J_{CF}$ = 5.5 Hz, C6), 125.61 (C3, C7), 123.86 (q, $^1J_{CF}$ = 272.1 Hz, C9), 118.04 (t, $^1J_{CF}$ = 240.9 Hz, C4), 61.48 (C10), 21.14 (C12), 13.99 (C11).

^{19}F NMR (470 MHz, $CDCl_3$) δ -62.95 (s, 3F, F-C9), -88.65 (d, $^3J_{HF}$ = 13.1 Hz, 2F, F-C4).

^{19}F{^{1}H} NMR (377 MHz, $CDCl_3$) δ -62.95 (s, 3F, F-C9), -88.74 (s, 2F, F-C4).

GC-EI-MS: (*m/z*) requires: $[(C_{14}H_{13}O_2F_5)^+]$ = 308.0830, (*m/z*) found: $[(C_{14}H_{13}O_2F_5)^+]$ = 308.0829.

FT-IR ($\tilde{\nu}$ = cm^{-1}): 2990 (w), 1733 (m), 1676 (w), 1452 (w), 1412 (w), 1378 (w), 1326 (s), 1228 (m), 1171 (m), 1125 (s), 1068 (s), 1010 (s), 987 (m), 844 (m), 809 (m), 781 (w), 740 (w), 683 (w), 603 (w).

Ethyl (*Z*)-4,4-difluoro-2-methyldec-2-enoate (19)

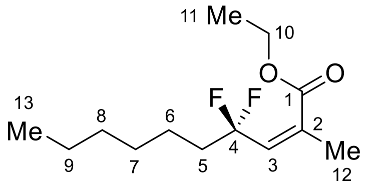

Compound **19** was prepared according to the General Procedure **F** with an amine:HF ratio of 1:5.5 using ethyl 2-hexyl-1-methylcycloprop-2-ene-1-carboxylate (**S19**) (21.0 mg, 0.10 mmol, 1.0 eq.) and *p*-iodotoluene **C1** (4.4 mg, 0.02 mmol, 20 mol%). The solvent was changed to DCM. After workup, the crude mixture was analysed by ^{19}F NMR (*Z*:*E* > 20:1). Purification by column chromatography (*n*-pentane:Et_2O 50:1) yielded the title compound **19** as a colorless oil (9.9 mg, 0.040 mmol, 40%).

$\mathbf{R_f}$ = 0.30 (*n*-pentane:Et_2O 50:1).

^{1}**H NMR** (400 MHz, $CDCl_3$) δ 5.58 (tq, $^3J_{HF}$ = 13.5 Hz, $^4J_{HH}$ = 1.6 Hz, 1H, H-C3), 4.23 (q, $^3J_{HH}$ = 7.1 Hz, 2H, H-C10), 2.14 – 1.86 (m, 5H, H-C5, H-C12), 1.52 – 1.41 (m, 2H, H-C6), 1.38 – 1.21 (m, 9H, H-C7~C9, H-C11), 0.88 (t, $^3J_{HH}$ = 6.6 Hz, 3H, H-C13).

^{13}**C NMR** (126 MHz, $CDCl_3$) δ 168.84 (C1), 135.36 (t, $^3J_{CF}$ = 7.2 Hz, C2), 126.70 (t, $^2J_{CF}$ = 28.8 Hz, C3), 121.39 (t, $^1J_{CF}$ = 239.7 Hz, C4), 61.26 (C10), 37.96 (t, $^2J_{CF}$ = 26.3 Hz, C5), 31.71 (*C*H_2), 29.13 (*C*H_2), 22.63 (*C*H_2), 22.21 (t, $^3J_{CF}$ = 3.9 Hz, C6), 21.00 (C12), 14.17 (*C*H_3), 14.10 (*C*H_3).

^{19}**F NMR** (470 MHz, $CDCl_3$) δ -93.10 – -93.27 (m, 2F, F-C4).

^{19}**F{^{1}H} NMR** (377 MHz, $CDCl_3$) δ -93.19 (s, 2F, F-C4).

GC-EI-MS: (*m/z*) requires: [$(C_{13}H_{22}O_2F_2)^+$] = 248.1582, (*m/z*) found: [$(C_{13}H_{22}O_2F_2)^+$] = 248.1581.

FT-IR ($\tilde{\nu}$ = cm^{-1}): 2956 (m), 2933 (m), 2864 (w), 1733 (s), 1676 (w), 1458 (m), 1372 (m), 1349 (m), 1234 (s), 1177 (s), 1142 (s), 1113 (s), 1022 (s), 964 (m), 861 (w), 775 (w).

Ethyl (*Z*)-4,4-difluoro-2-phenyl-4-(4-(trifluoromethyl)phenyl)but-2-enoate (*Z*-20)

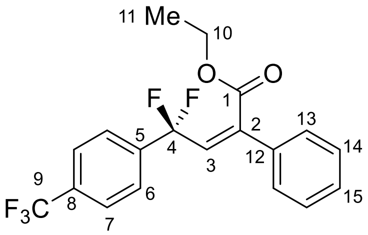

Compound **20** was prepared according to the General Procedure **F** with an amine:HF ratio of 1:5.5 using ethyl 1-phenyl-2-(4-(trifluoromethyl)phenyl)cycloprop-2-ene-1-carboxylate (**S20**) (66.5 mg, 0.20 mmol, 1.0 eq.) and aryl iodide **C7** (17.5 mg, 0.04 mmol, 20 mol%). After workup, the crude mixture was analysed by ^{19}F NMR (*Z*:*E* = 5.4:1). Purification by column chromatography (*n*-pentane:Et_2O 50:1) yielded the title compound **20** (*Z*: colorless oil, 21.4 mg, 0.058 mmol, 29%; *Z*/*E* mixture: colorless oil, 14.6 mg, 0.039 mmol, 20%).

$\mathbf{R_{\mathit{f}}}$ = 0.35 (*n*-pentane:Et_2O 30:1).

^{1}H NMR (400 MHz, $CDCl_3$) δ 7.78 (d, $^3J_{HH}$ = 8.2 Hz, 2H, H-C6), 7.72 (d, $^3J_{HH}$ = 8.2 Hz, 2H, H-C7), 7.46 – 7.34 (m, 5H, H-C13~C15), 6.12 (t, $^3J_{HF}$ = 13.3 Hz, 1H, H-C3), 4.32 (q, $^3J_{HH}$ = 7.2 Hz, 2H, H-C10), 1.31 (t, $^3J_{HH}$ = 7.1 Hz, 3H, H-C11).

^{13}C NMR (126 MHz, $CDCl_3$) δ 167.49 (C1), 139.95 (t, $^2J_{CF}$ = 28.6 Hz, C5), 139.81 (t, $^3J_{CF}$ = 6.3 Hz, C2), 134.31 (C12), 132.54 (q, $^2J_{CF}$ = 32.5 Hz, C8), 129.84 (C15), 129.08 (C14), 126.62 (C13), 126.26 (t, $^3J_{CF}$ = 5.5 Hz, C6), 125.80 (q, $^3J_{CF}$ = 3.8 Hz, C7), 123.82 (q, $^1J_{CF}$ = 272.7 Hz, C9), 123.45 (t, $^2J_{CF}$ = 29.2 Hz, C3), 118.51 (t, $^1J_{CF}$ = 242.1 Hz, C4), 61.99 (C10), 14.08 (C11).

^{19}F NMR (470 MHz, $CDCl_3$) δ -62.96 (s, 3F, F-C9), -90.48 (d, $^3J_{HF}$ = 13.2 Hz, 2F, F-C4).

^{19}F{^{1}H} NMR (377 MHz, $CDCl_3$) δ -62.96 (s, 3F, F-C9), -90.60 (s, 2F, F-C4).

GC-EI-MS: (*m/z*) requires: $[(C_{19}H_{15}O_2F_5)^+]$ = 370.0987, (*m/z*) found: $[(C_{19}H_{15}O_2F_5)^+]$ = 370.0984.

FT-IR ($\tilde{\nu}$ = cm^{-1}): 2985 (w), 1733 (m), 1647 (w), 1498 (w), 1452 (w), 1412 (w), 1372 (w), 1326 (s), 1194 (s), 1171 (s), 1125 (s), 1062 (s), 1022 (m), 999 (s), 844 (m), 758 (m), 689 (m), 608 (m), 551 (m).

Ethyl 4,4-difluoro-2-phenyldec-2-enoate (21)

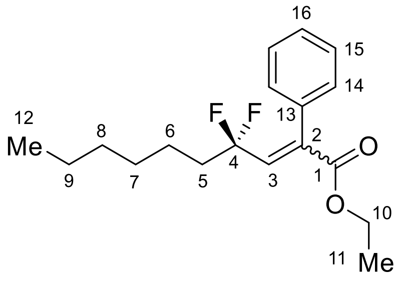

Compound **21** was prepared according to the General Procedure **F** with an amine:HF ratio of 1:6.5 using ethyl 2-hexyl-1-phenylcycloprop-2-ene-1-carboxylate (**S21**) (54.5 mg, 0.20 mmol, 1.0 eq.) and aryl iodide **C7** (17.5 mg, 0.04 mmol, 20 mol%). After workup, the crude mixture was analysed by ^{19}F NMR (*E*:*Z* = 3.2:1). Purification by column chromatography (*n*-pentane:Et_2O 30:1) yielded the title compound **21** as a yellow oil (31.2 mg, 0.10 mmol, 50%, *E*:*Z* = 3.2:1).

R*f* = 0.35 (*n*-pentane:Et_2O 30:1).

^{1}H NMR (400 MHz, $CDCl_3$) δ 7.42 – 7.33 (m, ArH), 7.21 (dd, $^3J_{HH}$ = 6.8 Hz, $^4J_{HH}$ = 2.8 Hz, 2H, H-C14, *E*), 6.94 (t, $^3J_{HF}$ = 12.3 Hz, 1H, H-C3, *E*), 5.98 (t, $^3J_{HF}$ = 13.5 Hz, 1H, H-C3, *Z*), 4.33 (q, $^3J_{HH}$ = 7.1 Hz, 2H, H-C10, *Z*), 4.23 (q, $^3J_{HH}$ = 7.1 Hz, 2H, H-C10, *E*), 2.22 – 1.90 (m, 2H, H-C5, *Z*), 1.75 – 1.60 (m, 2H, H-C5, *E*), 1.52 – 1.12 (m, 11H, H-C6~C9, H-C11), 0.94 – 0.82 (m, 3H, H-C12).

^{13}C NMR (126 MHz, $CDCl_3$) δ 166.61 (C1, *E*), 138.98 (t, $^3J_{CF}$ = 7.3 Hz, C2, *E*), 135.56 (t, $^2J_{CF}$ = 29.7 Hz, C3, *E*), 133.76 (C13, *E*), 129.28 (t, $^5J_{CF}$ = 2.1 Hz, C14, *E*), 128.33 (C16, *E*), 127.77 (C15, *E*), 121.51 (t, $^1J_{CF}$ = 239.7 Hz, C4, *E*), 61.95 (C10, *E*), 37.27 (t, $^2J_{CF}$ = 25.6 Hz, C5, *E*), 31.53 (*C*H_2, *E*), 28.92 (*C*H_2, *E*), 22.55 (*C*H_2, *E*), 22.13 (t, $^3J_{CF}$ = 3.9 Hz, C6, *E*), 14.23 (*C*H_3, *E*), 14.12 (*C*H_3, *E*).

^{19}F NMR (470 MHz, $CDCl_3$) δ -88.16 (td, $^3J_{HF}$ = 16.6, 12.4 Hz, 2F, F-C4, *E*), -93.97 (td, $^3J_{HF}$ = 16.5, 13.4 Hz, 2F, F-C4, *Z*).

^{19}F{^{1}H} NMR (377 MHz, $CDCl_3$) δ -88.16 (s, 2F, F-C4, *E*), -94.02 (s, 2F, F-C4, *Z*).

GC-EI-MS: (*m/z*) requires: [$(C_{18}H_{23}O_2F)^+$] = 290.1677, (*m/z*) found: [$(C_{18}H_{23}O_2F)^+$] = 290.1676.

FT-IR ($\tilde{\nu}$ = cm^{-1}): 2967 (s), 2365 (m), 1751 (w), 1705 (m), 1395 (m), 1246 (m), 1068 (s), 901 (w), 695 (w).

(3*R*,8*R*,10*S*,13*R*)-10,13-Dimethyl-17-((R)-6-methylheptan-2-yl)hexadecahydro-1H-cyclopenta[a]phenanthren-3-yl 4-((E)-3-(4-bromophenyl)-1,1,4,4,4-pentafluorobut-2-en-1-yl)benzoate (22)

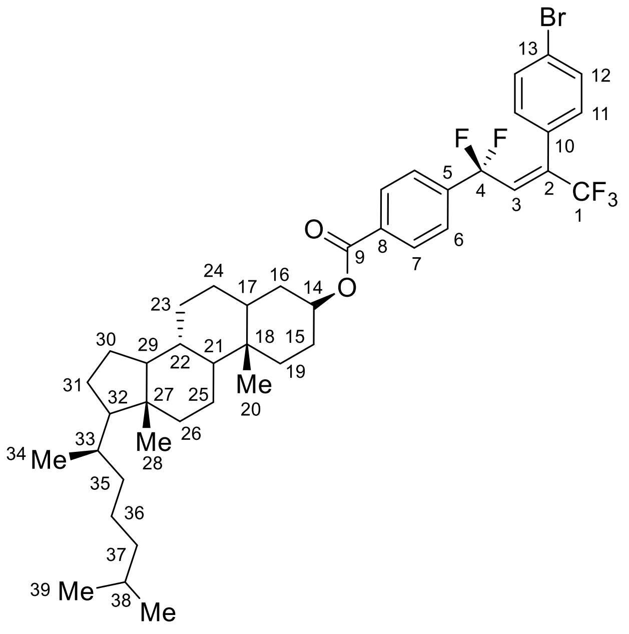

Compound **22** was prepared according to the General Procedure **F** with an amine:HF ratio of 1:8.0 using (3*R*,8*R*,10*S*,13*R*)-10,13-dimethyl-17-((*R*)-6-methylheptan-2-yl)hexadecahydro-1H-cyclopenta[a]phenanthren-3-yl 4-(3-(4-bromophenyl)-3-(trifluoromethyl)cycloprop-1-en-1-yl)benzoate (**S22**) (37.7 mg, 0.05 mmol, 1.0 eq.) and aryl iodide **C7** (4.4 mg, 0.01 mmol, 20 mol%). The solvent was changed to DCM. After workup, the crude mixture was analysed by ^{19}F NMR (*E*:*Z* > 20:1). Purification by column chromatography (*n*-pentane:DCM 5:1-4:1) yielded the title compound **22** as a white wax (17.7 mg, 0.022 mmol, 45%).

R*f* = 0.30 (*n*-pentane:DCM 4:1).

^{1}H NMR (400 MHz, $CDCl_3$) δ 7.98 (d, $^3J_{HH}$ = 8.3 Hz, 2H, H-C7), 7.45 – 7.40 (m, 2H, H-C12), 7.30 – 7.26 (m, 2H, H-C6), 6.93 (d, $^3J_{HH}$ = 8.4 Hz, 2H, H-C11), 6.76 (tq, $^3J_{HF}$ = 11.4 Hz, $^4J_{HF}$ = 1.7 Hz, 1H, H-C3), 5.01 – 4.89 (m, 1H, H-C14), 2.02 – 1.89 (m, 2H), 1.88 – 1.76 (m, 2H), 1.75 – 1.62 (m, 3H), 1.56 – 1.43 (m, 4H), 1.40 – 1.21 (m, 9H), 1.18 – 0.97 (m, 9H), 0.94 – 0.87 (m, 4H), 0.89 – 0.86 (m, 6H), 0.86 – 0.84 (m, 3H), 0.74 – 0.63 (m, 4H).

^{13}C NMR (126 MHz, $CDCl_3$) δ 165.22 (C9), 139.87 (t, $^2J_{CF}$ = 27.2 Hz, C5), 136.47 (C2), 132.97 (C8), 131.44 (C12), 131.15 (C3), 131.00 (C11), 129.85 (C7), 128.71 (C10), 125.14 (t, $^3J_{CF}$ = 5.5 Hz, C6), 123.97 (C13), 122.17 (q, $^1J_{CF}$ = 274.8 Hz, C1), 117.77 (t, $^1J_{CF}$ = 242.4 Hz, C4), 75.13 (C14), 56.59 (*C*H), 56.44 (*C*H), 54.40 (*C*H), 44.87 (*C*H), 42.77 (*C*), 40.15 (*C*H_2), 39.68 (*C*H_2), 36.94 (*C*H_2), 36.33 (*C*H_2), 35.96 (*C*H), 35.68 (*C*H), 35.66 (*C*), 34.23 (*C*H_2), 32.16 (*C*H_2), 28.80 (*C*H_2), 28.40 (*C*H_2), 28.17 (*C*H), 27.70 (*C*H_2), 24.37 (*C*H_2), 24.00 (*C*H_2), 22.97 (*C*H_3), 22.72 (*C*H_3), 21.40 (*C*H_2), 18.83 (*C*H_3), 12.46 (*C*H_3), 12.24 (*C*H_3).

^{19}F NMR (470 MHz, $CDCl_3$) δ -67.99 (d, $^4J_{HF}$ = 1.9 Hz, 3F, F-C1), -87.63 (d, $^3J_{HF}$ = 12.7 Hz, 2F, F-C4).

^{19}F{^{1}H} NMR (470 MHz, $CDCl_3$) δ -67.99 (t, $^{5}J_{FF}$ = 1.9 Hz, 3F, F-C1), -87.63 (q, $^{5}J_{FF}$ = 2.1 Hz, 2F, F-C4).

ESI-MS: (*m/z*) requires: [$(C_{44}H_{56}O_2BrF_5Na)^+$] = 813.3276, (*m/z*) found: [$(C_{44}H_{56}O_2BrF_5Na)^+$] = 813.3281.

FT-IR ($\tilde{\nu}$ = cm^{-1}): 2933 (m), 2359 (s), 2336 (s), 1716 (w), 1274 (m), 1068 (m), 672 (w).

Ethyl (*Z*)-4,4-difluorodec-2-enoate (23)

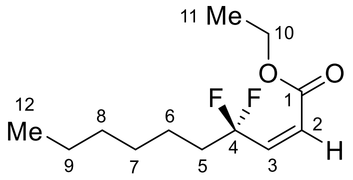

Compound **23** was prepared according to the General Procedure **F** with an amine:HF ratio of 1:5.5 using ethyl 2-hexylcycloprop-2-ene-1-carboxylate (**S23**) (19.6 mg, 0.10 mmol, 1.0 eq.) and iodobenzene **C2** (4.1 mg, 0.02 mmol, 20 mol%). The solvent was changed to DCM. After workup, the crude mixture was analysed by ^{19}F NMR (54%, *Z*:*E* = 3.2:1).

Compound **23** was prepared according to the General Procedure **F** with an amine:HF ratio of 1:5.5 using ethyl 2-hexylcycloprop-2-ene-1-carboxylate (**S23**) (19.6 mg, 0.10 mmol, 1.0 eq.) and aryl iodide **C7** (8.8 mg, 0.02 mmol, 20 mol%). The solvent was changed to DCM. After workup, the crude mixture was analysed by ^{19}F NMR (*Z*:*E* = 14.3:1). Purification by column chromatography (*n*-pentane:Et_2O 70:1) yielded the title compound ***Z*-23** as a colorless oil (10.2 mg, 0.044 mmol, 44%).

R_f = 0.30 (*n*-pentane:Et_2O 40:1).

^{1}H NMR (400 MHz, $CDCl_3$) δ 6.10 – 5.90 (m, 2H, H-C2, H-C3), 4.22 (q, $^{3}J_{HH}$ = 7.1 Hz, 2H, H-C10), 2.21 – 2.03 (m, 2H, H-C5), 1.54 – 1.44 (m, 2H, H-C6), 1.38 – 1.23 (m, 9H, H-C7~C9, H-C11), 0.94 – 0.85 (m, 3H, H-C12).

^{13}C NMR (126 MHz, $CDCl_3$) δ 165.21 (C1), 135.89 (t, $^{2}J_{CF}$ = 30.9 Hz, C3), 125.35 (t, $^{3}J_{CF}$ = 7.7 Hz, C2), 121.45 (t, $^{1}J_{CF}$ = 240.1 Hz, C4), 61.19 (C10), 37.39 (t, $^{2}J_{CF}$ = 25.3 Hz, C5), 31.69 (CH_2), 29.11 (CH_2), 22.62 (CH_2), 22.17 (t, $^{3}J_{CF}$ = 4.0 Hz, C6), 14.16 (CH_3), 14.14 (CH_3).

^{19}F NMR (470 MHz, $CDCl_3$) δ -93.20 (td, $^{3}J_{HF}$ = 16.9, 11.5 Hz, 2F, F-C4).

^{19}F{^{1}H} NMR (470 MHz, $CDCl_3$) δ -93.20 (s, 2F, F-C4).

GC-EI-MS: (*m/z*) requires: [$(C_{12}H_{19}O_2F)^+$] = 214.1364, (*m/z*) found: [$(C_{12}H_{19}O_2F)^+$] = 214.1363.

FT-IR ($\tilde{\nu}$ = cm^{-1}): 2933 (m), 2864 (w), 2359 (w), 1733 (s), 1659 (w), 1464 (w), 1406 (m), 1194 (s), 1171 (s), 1091 (m), 1027 (s), 970 (m), 878 (w), 804 (w), 746 (w), 608 (w).

Ethyl (*Z*)-4,4-difluoro-13-hydroxytridec-2-enoate (24)

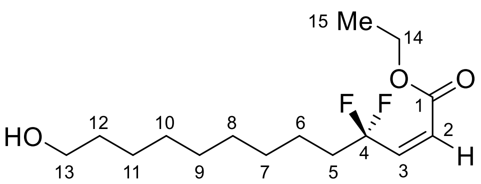

Compound **24** was prepared according to the General Procedure **F** with an amine:HF ratio of 1:5.5 using ethyl 2-(9-hydroxynonyl)cycloprop-2-ene-1-carboxylate (**S24**) (25.4 mg, 0.10 mmol, 1.0 eq.) and iodobenzene **C2** (4.1 mg, 0.02 mmol, 20 mol%). The solvent was changed to DCM. After workup, the crude mixture was analysed by ^{19}F NMR (*Z*:*E* = 3.5:1). Purification by column chromatography (*n*-pentane:Et_2O 1:1) yielded the title compound **24** (*Z*: colorless oil, 6.4 mg, 0.022 mmol, 22%; *Z*/*E* mixture: colorless oil, 8.3 mg, 0.028 mmol, 28%).

Compound **24** was prepared according to the General Procedure **F** with an amine:HF ratio of 1:5.5 using ethyl 2-(9-hydroxynonyl)cycloprop-2-ene-1-carboxylate (**S24**) (25.4 mg, 0.10 mmol, 1.0 eq.) and aryl iodide **C7** (8.8 mg, 0.02 mmol, 20 mol%). The solvent was changed to DCM. After workup, the crude mixture was analysed by ^{19}F NMR (40%, *Z*:*E* = 12.3:1).

R*f* = 0.35 (*n*-pentane:Et_2O 1:1).

^{1}H NMR (400 MHz, $CDCl_3$) δ 6.02 – 5.84 (m, 2H, H-C2, H-C3), 4.15 (q, $^{3}J_{HH}$ = 7.2 Hz, 2H, H-C14), 3.57 (t, $^{3}J_{HH}$ = 7.2 Hz, 2H, H-C13), 2.15 – 1.96 (m, 2H, H-C5), 1.54 – 1.46 (m, 2H, H-C12), 1.46 – 1.38 (m, 2H, H-C6), 1.32 – 1.20 (m, 13H, H-C7~C11, H-C15).

^{13}C NMR (126 MHz, $CDCl_3$) δ 165.21 (C1), 135.90 (t, $^{2}J_{CF}$ = 30.9 Hz, C3), 125.36 (t, $^{3}J_{CF}$ = 7.7 Hz, C2), 121.44 (t, $^{1}J_{CF}$ = 240.0 Hz, C4), 63.21 (C13), 61.20 (C14), 37.36 (t, $^{2}J_{CF}$ = 25.3 Hz, C5), 32.92 (C12), 29.51 (CH_2), 29.48 (CH_2), 29.40 (CH_2), 25.84 (CH_2), 22.18 (t, $^{3}J_{CF}$ = 4.0 Hz, C6), 14.15 (C15).

^{19}F NMR (470 MHz, $CDCl_3$) δ -93.21 (td, $^{3}J_{HF}$ = 16.9, 11.5 Hz, 2F, F-C4).

^{19}F{^{1}H} NMR (470 MHz, $CDCl_3$) δ -93.21 (s, 2F, F-C4).

ESI-MS: (*m/z*) requires: [$(C_{15}H_{26}O_3F_2Na)^+$] = 315.1742, (*m/z*) found: [$(C_{15}H_{26}O_3F_2Na)^+$] = 315.1752.

FT-IR ($\tilde{\nu}$ = cm^{-1}): 2927 (m), 2858 (m), 1733 (s), 1653 (w), 1464 (w), 1406 (m), 1177 (s), 1027 (s), 872 (w), 723 (w).

(*Z*)-13-Ethoxy-10,10-difluoro-13-oxotridec-11-en-1-yl nicotinate (25)

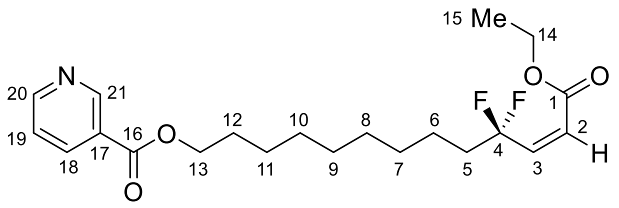

Compound **25** was prepared according to the General Procedure **F** with an amine:HF ratio of 1:5.5 using 9-(3-(ethoxycarbonyl)cycloprop-1-en-1-yl)nonyl nicotinate (**S25**) (36.0 mg, 0.10 mmol, 1.0 eq.) and iodobenzene **C2** (4.1 mg, 0.02 mmol, 20 mol%). The solvent was changed to DCM. After workup, the crude mixture was analysed by ^{19}F NMR (*Z*:*E* = 3.1:1). Purification by column chromatography (*n*-pentane:Et_2O 1:1) yielded the title compound **25** (*Z*: colorless oil, 9.7 mg, 0.025 mmol, 25%; *Z*/*E* mixture: colorless oil, 12.9 mg, 0.032 mmol, 32%).

Compound **25**was prepared according to the General Procedure **F** with an amine:HF ratio of 1:5.5 using 9-(3-(ethoxycarbonyl)cycloprop-1-en-1-yl)nonyl nicotinate (**S25**) (36.0 mg, 0.10 mmol, 1.0 eq.) and aryl iodide **C7** (8.8 mg, 0.02 mmol, 20 mol%). The solvent was changed to DCM. After workup, the crude mixture was analysed by ^{19}F NMR (43%, *Z*:*E* = 13.3:1).

R_f = 0.35 (*n*-pentane:Et_2O 1:1).

^{1}H NMR (400 MHz, $CDCl_3$) δ 9.23 (d, $^4J_{HH}$ = 1.5 Hz, 1H, H-C21), 8.77 (dd, $^3J_{HH}$ = 4.9 Hz, $^4J_{HH}$ = 1.8 Hz, 1H, H-C20), 8.29 (dt, $^3J_{HH}$ = 8.0 Hz, $^4J_{HH}$ = 2.0 Hz, 1H, H-C18), 7.39 (dd, $^3J_{HH}$ = 8.0, 4.9 Hz, 1H, H-C19), 6.11 – 5.88 (m, 2H, H-C2, H-C3), 4.35 (t, $^3J_{HH}$ = 6.7 Hz, 2H, H-C13), 4.22 (q, $^3J_{HH}$ = 7.2 Hz, 2H, H-C14), 2.21 – 2.01 (m, 2H, H-C5), 1.84 – 1.71 (m, 2H, H-C12), 1.54 – 1.40 (m, 4H, H-C6, H-C11), 1.38 – 1.26 (m, 11H, H-C7~C10, H-C15).

^{13}C NMR (126 MHz, $CDCl_3$) δ 165.50 (C16), 165.18 (C1), 153.49 (C20), 151.06 (C21), 137.15 (C18), 135.90 (t, $^2J_{CF}$ = 31.0 Hz, C3), 126.51 (C17), 125.38 (t, $^3J_{CF}$ = 7.7 Hz, C2), 123.41 (C19), 121.41 (t, $^1J_{CF}$ = 240.1 Hz, C4), 65.71 (C13), 61.18 (C14), 37.35 (t, $^2J_{CF}$ = 25.5 Hz, C5), 29.45 (CH_2), 29.41 (CH_2), 29.40 (CH_2), 29.33 (CH_2), 28.78 (C12), 26.10 (C11), 22.18 (t, $^3J_{CF}$ = 3.9 Hz, C6), 14.14 (C15).

^{19}F NMR (470 MHz, $CDCl_3$) δ -93.21 (td, $^3J_{HF}$ = 16.9, 11.5 Hz, 2F, F-C4).

^{19}F{^{1}H} NMR (470 MHz, $CDCl_3$) δ -93.21 (s, 2F, F-C4).

ESI-MS: (*m/z*) requires: [($C_{21}H_{29}NO_4F_2Na)^+$] = 420.1957, (*m/z*) found: [($C_{21}H_{29}NO_4F_2Na)^+$] = 420.1967.

FT-IR ($\tilde{\nu}$ = cm^{-1}): 2933 (m), 2858 (w), 1722 (s), 1590 (w), 1464 (w), 1412 (w), 1280 (s), 1194 (m), 1113 (s), 1022 (m), 947 (w), 832 (w), 740 (m), 700 (m).

Methyl 2-(difluoro(phenyl)methyl)-3-methylbut-2-enoate (26)

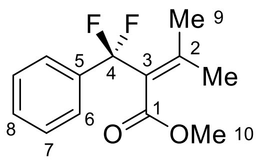

Compound **25** was prepared according to the General Procedure **F** with an amine:HF ratio of 1:4.5 using methyl 3,3-dimethyl-2-phenylcycloprop-1-ene-1-carboxylate (**S26**) (20.2 mg, 0.10 mmol, 1.0 eq.) and aryl iodide **C7** (8.8 mg, 0.02 mmol, 20 mol%). The solvent was changed to DCM. The crude product was purified by column chromatography (*n*-pentane:Et_2O 25:1) yielded the title compound **26** as a colorless oil (9.8 mg, 0.041 mmol, 41%).

R_f = 0.25 (*n*-pentane:Et_2O 20:1).

^{1}H NMR (400 MHz, $CDCl_3$) δ 7.74 – 7.60 (m, 2H, H-C6), 7.49 – 7.37 (m, 3H, H-C7, H-C8), 3.75 (s, 3H, H-C10), 1.89 (t, $^5J_{HF}$ = 2.5 Hz, 3H, H-C9), 1.69 (t, $^5J_{HF}$ = 2.2 Hz, 3H, H-C9).

^{13}C NMR (126 MHz, $CDCl_3$) δ 167.48 (t, $^3J_{CF}$ = 3.6 Hz, C1), 145.11 (t, $^3J_{CF}$ = 5.5 Hz, C2), 137.14 (t, $^2J_{CF}$ = 27.8 Hz, C5), 130.31 (t, $^4J_{CF}$ = 2.0 Hz, C7), 128.55 (C3, C8), 126.15 (t, $^3J_{CF}$ = 5.1 Hz, C6), 119.30 (t, $^1J_{CF}$ = 241.1 Hz, C4), 52.25 (C10), 23.63 (C9), 21.27 (t, $^4J_{CF}$ = 1.8 Hz, C9).

^{19}F NMR (470 MHz, $CDCl_3$) δ -81.21 – -81.28 (m, 2F, F-C4).

^{19}F{^{1}H} NMR (470 MHz, $CDCl_3$) δ -81.25 (s, 2F, F-C4).

ESI-MS: (*m/z*) requires: [($C_{13}H_{14}O_2F_2Na)^+$] = 263.0854, (*m/z*) found: [($C_{13}H_{14}O_2F_2Na)^+$] = 263.0853.

FT-IR ($\tilde{\nu}$ = cm^{-1}): 2950 (w), 2921 (w), 2359 (w), 1728 (s), 1659 (w), 1452 (m), 1378 (w), 1309 (m), 1223 (s), 1177 (m), 1108 (m), 1079 (m), 1027 (s), 981 (s), 907 (w), 884 (m), 769 (m), 700 (s), 666 (m).

Methyl 2-(difluoro(4-(trifluoromethyl)phenyl)methyl)-3-methylbut-2-enoate (27)

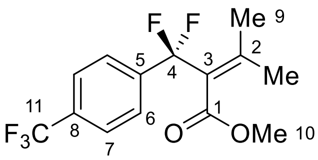

Compound **27** was prepared according to the General Procedure **F** with an amine:HF ratio of 1:6.0 using methyl 3,3-dimethyl-2-(4-(trifluoromethyl)phenyl)cycloprop-1-ene-1-carboxylate (**S27**) (27.0 mg, 0.10 mmol, 1.0 eq.) and *p*-iodotoluene **C1** (4.4 mg, 0.02 mmol, 20 mol%). The solvent was changed to DCM. The crude product was purified by column chromatography (*n*-pentane:DCM 3.5:1-2.5:1) yielded the title compound **27** as a colorless oil (21.0 mg, 0.068 mmol, 68%).

R*f* = 0.40 (*n*-pentane:DCM 2.5:1).

^{1}H NMR (400 MHz, $CDCl_3$) δ 7.80 (d, $^3J_{HH}$ = 8.2 Hz, 2H, H-C6), 7.70 (d, $^3J_{HH}$ = 8.2 Hz, 2H, H-C7), 3.76 (s, 3H, H-C10), 1.90 (t, $^5J_{HF}$ = 2.6 Hz, 3H, H-C9), 1.71 (t, $^5J_{HF}$ = 2.2 Hz, 3H, H-C9).

^{13}C NMR (126 MHz, $CDCl_3$) δ 167.12 (t, $^3J_{CF}$ = 3.8 Hz, C1), 146.02 (t, $^3J_{CF}$ = 5.6 Hz, C2), 140.75 (C5), 132.43 (C8), 127.88 (C3), 126.74 (t, $^3J_{CF}$ = 5.1 Hz, C6), 125.66 (q, $^3J_{CF}$ = 3.8 Hz, C7), 123.85 (q, $^1J_{CF}$ = 272.5 Hz, C11), 118.57 (t, $^1J_{CF}$ = 242.4 Hz, C4), 52.35 (C10), 23.71 (C9), 21.34 (t, $^4J_{CF}$ = 2.2 Hz, C9).

^{19}F NMR (470 MHz, $CDCl_3$) δ -62.95 (s, 3F, F-C11), -82.44 (s, 2F, F-C4).

^{19}F{^{1}H} NMR (377 MHz, $CDCl_3$) δ -62.95 (s, 3F, F-C11), -82.43 (s, 2F, F-C4).

ESI-MS: (*m/z*) requires: [$(C_{14}H_{13}O_2F_5Na)^+$] = 331.0728 (*m/z*) found: [$(C_{14}H_{13}O_2F_5Na)^+$] = 331.0728.

FT-IR ($\tilde{\nu}$ = cm^{-1}): 2956 (w), 1728 (m), 1659 (w), 1435 (m), 1412 (m), 1378 (w), 1320 (s), 1251 (m), 1228 (m), 1171 (s), 1125 (s), 1068 (s), 1039 (s), 981 (m), 907 (w), 884 (w), 844 (s), 781 (m), 735 (m), 677 (w), 608 (w).

2-(Difluoro(4-(trifluoromethyl)phenyl)methyl)-N,N,3-trimethylbut-2-enamide (28)

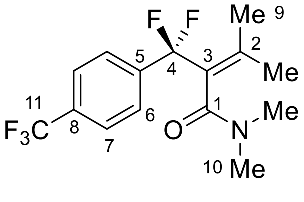

Compound **28** was prepared according to the General Procedure **F** with an amine:HF ratio of 1:6.0 using N,N,3,3-tetramethyl-2-(4-(trifluoromethyl)phenyl)cycloprop-1-ene-1-carboxamide (**S28**) (28.3 mg, 0.10 mmol, 1.0 eq.) and *p*-iodotoluene **C1** (4.4 mg, 0.02 mmol, 20 mol%). The solvent was changed to DCM. The crude product was purified by column

chromatography (n-pentane: Et_2O 1:1) yielded the title compound **28** as a colorless oil (22.3 mg, 0.069 mmol, 69%).

R$_f$ = 0.20 (n-pentane: Et_2O 1:1).

^{1}H NMR (400 MHz, $CDCl_3$) δ 7.94 (d, $^3J_{HH}$ = 8.1 Hz, 2H, H-C6), 7.68 (d, $^3J_{HH}$ = 8.0 Hz, 2H, H-C7), 3.12 (s, 3H, H-C10), 3.05 (s, 3H, H-C10), 1.78 (d, $^5J_{HF}$ = 3.5 Hz, 3H, H-C9), 1.60 (d, $^5J_{HF}$ = 3.8 Hz, 3H, H-C9).

^{13}C NMR (126 MHz, $CDCl_3$) δ 167.82 (C1), 140.82 (C5), 140.76 (dd, $^3J_{CF}$ = 9.8, 3.4 Hz, C2), 132.24 (C8), 129.67 (dd, $^2J_{CF}$ = 36.4, 22.3 Hz, C3), 127.00 (dd, $^3J_{CF}$ = 6.3, 3.8 Hz, C6), 125.56 (C7), 123.92 (q, $^1J_{CF}$ = 272.7 Hz, C11), 119.15 (dd, $^1J_{CF}$ = 248.3, 234.6 Hz, C4), 37.90 (C10), 34.43 (C10), 22.76 (t, $^4J_{CF}$ = 1.5 Hz, C9), 20.52 (d, $^4J_{CF}$ = 3.0 Hz, C9).

^{19}F NMR (470 MHz, $CDCl_3$) δ -62.90 (s, 3F, F-C11), -78.76 (d, $^2J_{FF}$ = 270.3 Hz, 1F, F-C4), -88.69 – -89.98 (m, 1F, F-C4).

^{19}F{^{1}H} NMR (377 MHz, $CDCl_3$) δ -62.90 (s, 3F, F-C11), -78.72 (d, $^2J_{FF}$ = 270.3 Hz, 1F, F-C4), -89.39 (d, $^2J_{FF}$ = 270.3 Hz, 1F, F-C4).

ESI-MS: (m/z) requires: [$(C_{15}H_{16}NOF_5Na)^+$] = 344.1044, (m/z) found: [$(C_{15}H_{16}NOF_5Na)^+$] = 344.1044.

FT-IR ($\tilde{\nu}$ = cm^{-1}): 2927 (w), 1636 (s), 1498 (w), 1452 (w), 1400 (m), 1326 (s), 1291 (m), 1240 (m), 1165 (s), 1125 (s), 1096 (m), 1068 (s), 1033 (s), 970 (m), 844 (m), 706 (w), 660 (m), 608 (w).

Methyl 2-cyclohexylidene-3,3-difluoro-3-(4-(trifluoromethyl)phenyl)propanoate (29)

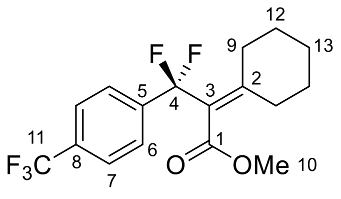

Compound **S1** was prepared according to the General Procedure **F** with an amine:HF ratio of 1:6.0 using methyl 2-(4-(trifluoromethyl)phenyl)spiro[2.5]oct-1-ene-1-carboxylate (**S29**) (31.0 mg, 0.10 mmol, 1.0 eq.) and p-iodotoluene **C1** (4.4 mg, 0.02 mmol, 20 mol%). The solvent was changed to DCM. The crude product was purified by column chromatography (n-pentane:DCM 3:1) yielded the title compound **29** as a colorless oil (17.6 mg, 0.051 mmol, 51%).

R$_f$ = 0.40 (n-pentane:DCM 3:1).

^{1}**H NMR** (599 MHz, $CDCl_3$) δ 7.83 (d, $^{3}J_{HH}$ = 7.7 Hz, 2H, H-C6), 7.70 (d, $^{3}J_{HH}$ = 8.1 Hz, 2H, H-C7), 3.78 (s, 3H, H-C10), 2.25 – 2.19 (m, 2H, H-C9), 2.09 – 2.03 (m, 2H, H-C9), 1.71 – 1.65 (m, 2H, H-C12), 1.56 – 1.50 (m, 2H, H-C13), 1.46 – 1.40 (m, 2H, H-C12).

^{13}**C NMR** (151 MHz, $CDCl_3$) δ 167.19 (t, $^{3}J_{CF}$ = 3.5 Hz, C1), 152.44 (t, $^{3}J_{CF}$ = 5.8 Hz, C2), 141.19 (C5), 132.42 (C8), 126.77 (t, $^{3}J_{CF}$ = 4.9 Hz, C6), 125.64 (q, $^{3}J_{CF}$ = 3.8 Hz, C7), 124.89 (t, $^{2}J_{CF}$ = 29.4 Hz, C3), 123.86 (q, $^{1}J_{CF}$ = 272.3 Hz, C11), 118.45 (t, $^{1}J_{CF}$ = 242.2 Hz, C4), 52.42 (C10), 33.90 (C9), 31.40 (C9), 28.21 (C12), 27.36 (C12), 25.87 (C13).

^{19}**F NMR** (564 MHz, $CDCl_3$) δ -62.93 (s, 3F, F-C11), -81.19 (s, 2F, F-C4).

^{19}**F{^{1}H} NMR** (377 MHz, $CDCl_3$) δ -62.93 (s, 3F, F-C11), -81.18 (s, 2F, F-C4).

ESI-MS: (*m/z*) requires: $[(C_{17}H_{17}O_2F_5Na)^+]$ = 371.1041, (*m/z*) found: $[(C_{17}H_{17}O_2F_5Na)^+]$ = 371.1042.

FT-IR ($\tilde{\nu}$ = cm^{-1}): 2939 (w), 1733 (m), 1326 (s), 1223 (m), 1171 (s), 1131 (s), 1068 (s), 1045 (m), 849 (w).

1.5 Product derivatisation

1,1,1,4,4-Pentafluoro-2-phenyl-4-(4-(trifluoromethyl)phenyl)butane-2,3-diol (30)

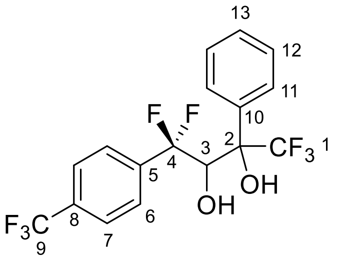

In a Schlenk tube were added (*E*)-1-(1,1,4,4,4-pentafluoro-3-phenylbut-2-en-1-yl)-4-(trifluoromethyl)benzene (**1**) (19.5 mg, 0.05 mmol, 1.0 eq.), THF (0.5 mL) and water (0.1 mL) under Ar atmosphere. Then, NMO (37.4 mg, 0.32 mmol, 6.0 eq.) and OsO_4 (2.5 wt% in *t*BuOH, 0.26 mL, 0.02 mmol, 40 mol%) were successively added. The resulting reaction mixture was stirred at 50-65 °C for 48 h. The reaction was cooled to room temperature and diluted with distilled water. The layers were separated and the aqueous phase was extracted three times with Et_2O. The combined organic layers were dried over anhydrous Na_2SO_4, filtered and concentrated under reduced pressure. The residue was purified by column chromatography (*n*-pentane:Et_2O 5:1) to yield the title compound as a white solid (15.6 mg, 0.039 mmol, 74%).

$\mathbf{R}_f$ = 0.30 (*n*-pentane:Et_2O 6:1).

^{1}H NMR (400 MHz, $CDCl_3$) δ 7.48 (d, $^{3}J_{HH}$ = 8.1 Hz, 2H, H-C7), 7.36 (d, $^{3}J_{HH}$ = 7.4 Hz, 2H, H-C11), 7.32 – 7.27 (m, 1H, H-C13), 7.26 – 7.19 (m, 4H, H-C6, H-C12), 4.93 (ddd, $^{3}J_{HF}$ = 11.2, 7.5 Hz, $^{3}J_{HH}$ = 4.9 Hz, 1H, H-C3), 3.50 (s, 1H, OH), 3.18 – 3.09 (m, 1H, OH).

^{13}C NMR (126 MHz, $CDCl_3$) δ 137.71 (t, $^{2}J_{CF}$ = 26.1 Hz, C5), 132.63 (C10), 132.18 (q, $^{2}J_{CF}$ = 33.4 Hz, C8), 129.15 (C13), 128.12 (C12), 126.69 (t, $^{3}J_{CF}$ = 6.6 Hz, C6), 126.38 (C11), 125.01 (q, $^{3}J_{CF}$ = 3.8 Hz, C7), 124.76 (q, $^{1}J_{CF}$ = 289.8 Hz, C1), 123.75 (q, $^{1}J_{CF}$ = 272.5 Hz, C9), 119.78 (t, $^{1}J_{CF}$ = 250.0 Hz, C4), 77.60 (app. q, $^{2}J_{CF}$ = 29.2 Hz, C2), 74.50 (t, $^{2}J_{CF}$ = 27.8 Hz, C3).

^{19}F NMR (470 MHz, $CDCl_3$) δ -63.12 (s, 3F, CF_3), -75.43 (s, 3F, CF_3), -97.12 (dd, $^{2}J_{FF}$ = 259.4 Hz, $^{3}J_{HF}$ = 7.7 Hz, 1F, F-C4), -101.17 (dd, $^{2}J_{FF}$ = 258.7 Hz, $^{3}J_{HF}$ = 11.6 Hz, 1F, F-C4).

^{19}F{^{1}H} NMR (377 MHz, $CDCl_3$) δ -63.11 (s, 3F, CF_3), -75.41 (s, 3F, CF_3), -97.07 (d, $^{2}J_{FF}$ = 258.8 Hz, 1F, F-C4), -101.20 (d, $^{2}J_{FF}$ = 258.8 Hz, 1F, F-C4).

ESI-MS: (*m/z*) requires: $[(C_{17}H_{12}O_2F_8Na)^+]$ = 423.0602, (*m/z*) found: $[(C_{17}H_{12}O_2F_8Na)^+]$ = 423.0600.

FT-IR ($\tilde{\nu}$ = cm^{-1}): 3444 (w), 2365 (w), 1326 (m), 1159 (s), 1131 (s), 1073 (s), 1045 (m), 999 (w), 976 (m), 930 (w), 844 (m), 809 (m), 758 (w), 712 (s), 660 (m), 614 (m).

Melting Point: 72-73 ˚C.

(*E*)-1-(1,1,4-Trifluoro-3-phenylbut-3-en-1-yl)-4-(trifluoromethyl)benzene (31)

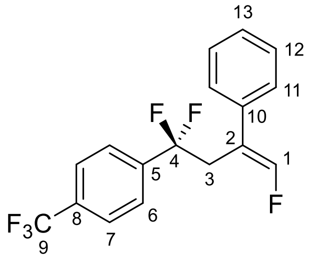

To a solution of (*E*)-1-(1,1,4,4,4-pentafluoro-3-phenylbut-2-en-1-yl)-4-(trifluoromethyl)benzene (**1**) (36.6 mg, 0.10 mmol, 1.0 eq.) in THF (0.4 mL) was slowly added a suspension of $LiAlH_4$ (7.6 mg, 0.20 mmol, 2.0 eq.) in THF (0.6 mL) at room temperature under Ar atmosphere. The reaction was stirred at room temperature for 24 h and then quenched by the addition of a 30% potassium sodium tartrate solution. The resulting mixture was stirred for 30 minutes and diluted with Et_2O. The layers were separated and the aqueous phase was extracted three times with Et_2O. The combined organic layers were washed with brine, dried over anhydrous Na_2SO_4, filtered and concentrated under reduced pressure. The residue was purified by column chromatography (*n*-pentane) to yield the title compound as a colorless oil (20.9 mg, 0.063 mmol, 63%).

R_f = 0.10 (*n*-pentane).

^{1}H NMR (400 MHz, $CDCl_3$) δ 7.59 (d, $^3J_{HH}$ = 8.2 Hz, 2H, H-C7), 7.50 (d, $^3J_{HH}$ = 8.2 Hz, 2H, H-C6), 7.33 – 7.26 (m, 3H, H-C12~C13), 7.20 – 7.13 (m, 2H, H-C11), 6.77 (d, $^2J_{HF}$ = 83.8 Hz, 1H, H-C1), 3.42 (td, $^3J_{HF}$ = 15.1 Hz, $^4J_{HF}$ = 2.7 Hz, 2H, H-C3).

^{13}C NMR (126 MHz, $CDCl_3$) δ 148.99 (d, $^1J_{CF}$ = 267.2 Hz, C1), 140.16 (*C*), 135.49 (*C*), 131.83 (*C*), 128.74 (C12), 127.97 (C13), 127.05 (d, $^4J_{CF}$ = 3.2 Hz, C11), 125.84 (t, $^3J_{CF}$ = 6.3 Hz, C6), 125.34 (q, $^3J_{CF}$ = 3.8 Hz, C7), 123.91 (app. q, $^1J_{CF}$ = 272.7 Hz, C9), 121.30 (app. t, $^1J_{CF}$ = 246.3 Hz, C4), 117.30 (*C*), 36.97 (td, $^2J_{CF}$ = 29.4 Hz, $^3J_{CF}$ = 4.0 Hz, C3).

^{19}F NMR (470 MHz, $CDCl_3$) δ -62.94 (s, 3F, F-C9), -93.25 (td, $^3J_{HF}$ = 15.2 Hz, $^5J_{FF}$ = 5.4 Hz, 2F, F-C4), -123.55 (dtt, $^2J_{HF}$ = 83.7 Hz, $^5J_{FF}$ = 5.3 Hz, $^4J_{HF}$ = 2.6 Hz, 1F, F-C1).

^{19}F{^{1}H} NMR (377 MHz, $CDCl_3$) δ -62.94 (s, 3F, F-C9), -93.24 (d, $^5J_{FF}$ = 5.3 Hz, 2F, F-C4), -123.55 (t, $^5J_{FF}$ = 5.3 Hz, 1F, F-C1).

GC-EI-MS: (*m/z*) requires: [$(C_{17}H_{12}F_6)^+$] = 330.0838, (*m/z*) found: [$(C_{17}H_{12}F_6)^+$] = 330.0842.

FT-IR ($\tilde{\nu}$ = cm^{-1}): 2985 (w), 2359 (m), 1653 (w), 1412 (w), 1320 (s), 1131 (s), 1068 (s), 1016 (m), 844 (m), 763 (m), 700 (w).

Diethyl 3-(difluoro(4-(trifluoromethyl)phenyl)methyl)oxirane-2,2-dicarboxylate (32)

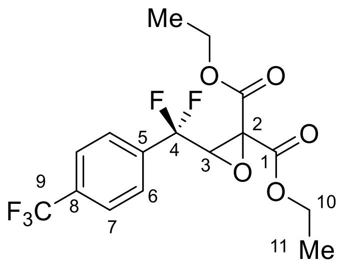

A mixture of *m*-CPBA (69.0 mg, 0.40 mmol, 4.0 eq.) and KOH (37.6 mg, 0.67 mmol, 6.7 eq.) in dry DCM (1.3 mL) was stirred under Ar atmosphere at room temperature for 1 h. A solution of diethyl 2-(2,2-difluoro-2-(4-(trifluoromethyl)phenyl)ethylidene)malonate (**13**) (36.6 mg, 0.10 mmol, 1.0 eq.) in dry DCM (1.3 mL) was added. After stirring overnight at room temperature, the mixture was filtered and the filtrate was evaporated. The residue was purified by column chromatography (*n*-pentane:Et_2O 10:1) to yield the title compound as a colorless oil (34.5 mg, 0.090 mmol, 90%).

R_f = 0.35 (*n*-pentane:Et_2O 10:1).

^{1}H NMR (400 MHz, $CDCl_3$) δ 7.84 – 7.66 (m, 4H, H-C6~C7), 4.41 – 4.20 (m, 4H, H-C10), 3.96 (dd, $^3J_{HF}$ = 10.3, 8.8 Hz, 1H, H-C3), 1.40 – 1.23 (m, 6H, H-C11).

^{13}C NMR (126 MHz, $CDCl_3$) δ 164.44 (C1), 162.77 (C1), 136.86 (t, $^2J_{CF}$ = 25.8 Hz, C5), 133.39 (app. q, $^2J_{CF}$ = 34.1 Hz, C8), 126.43 (t, $^3J_{CF}$ = 6.0 Hz, C6), 125.98 (q, $^3J_{CF}$ = 3.8 Hz, C7), 123.67 (app. q, $^1J_{CF}$ = 272.3 Hz, C9), 116.56 (dd, $^1J_{CF}$ = 247.6, 246.0 Hz, C4), 63.70 (C10), 62.76 (C10), 61.43 (dd, $^2J_{CF}$ = 38.9, 32.3 Hz, C3), 59.42 (d, $^3J_{CF}$ = 3.3 Hz, C2), 13.98 (C11), 13.95 (C11).

^{19}F NMR (470 MHz, $CDCl_3$) δ -63.12 (s, 3F, F-C9), -103.01 (dd, $^2J_{FF}$ = 264.2 Hz, $^3J_{HF}$ = 10.2 Hz, 1F, F-C4), -106.07 (dd, $^2J_{FF}$ = 264.2, $^3J_{HF}$ = 8.9 Hz, 1F, F-C4).

^{19}F{^{1}H} NMR (377 MHz, $CDCl_3$) δ -63.12 (s, 3F, F-C9), -103.07 (d, $^2J_{FF}$ = 264.0 Hz, 1F, F-C4), -106.33 (d, $^2J_{FF}$ = 264.0 Hz, 1F, F-C4).

ESI-MS: (*m/z*) requires: $[(C_{16}H_{15}O_5F_5Na)^+]$ = 405.0732, (*m/z*) found: $[(C_{16}H_{15}O_5F_5Na)^+]$ = 405.0727.

FT-IR ($\tilde{\nu}$ = cm^{-1}): 2990 (w), 1751 (s), 1412 (w), 1372 (w), 1326 (s), 1268 (m), 1240 (m), 1171 (m), 1131 (s), 1068 (s), 999 (m), 964 (m), 918 (w), 844 (m), 827 (m), 769 (m), 729 (w), 706 (w), 683 (w), 620 (w), 557 (w).

(*Z*)-4-Fluoro-2-methyl-4-(4-(trifluoromethyl)phenyl)but-3-en-1-ol (33)

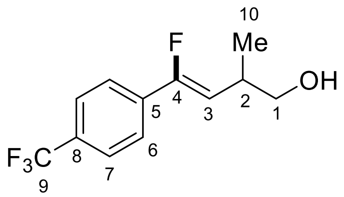

To a solution of Ethyl (*Z*)-4,4-difluoro-2-methyl-4-(4-(trifluoromethyl)phenyl)but-2-enoate (**18**) (30.8 mg, 0.10 mmol, 1.0 eq.) in THF (0.4 mL) was dropwise added DIBAL-H (1.0 M in toluene, 0.22 Ml, 2.2 eq.) at -78 °C under Ar atmosphere. After stirring at -78 °C for 2 h, the reaction was warmed to rt over 1 h and then quenched with a saturated aqueous solution of $NaHCO_3$. 5% HCl was added to remove the alumina gel. The layers were separated and the aqueous layer was extracted three times with Et_2O. The combined organic layers were dried over anhydrous Na_2SO_4, filtered, and concentrated under reduced pressure. The crude residue was purified by column chromatography (*n*-pentane:Et_2O 1.5:1) to yield the title compound as a colorless oil (19.5 mg, 0.079 mmol, 79%).

R*f* = 0.40 (*n*-pentane:Et_2O 1:1).

^{1}H NMR (400 MHz, $CDCl_3$) δ 7.61 (s, 4H, H-C6~C7), 5.43 (dd, $^3J_{HF}$ = 37.1 Hz, $^3J_{HH}$ = 9.4 Hz, 1H, H-C3), 3.74 – 3.49 (m, 2H, H-C1), 3.16 – 2.96 (m, 1H, H-C2), 1.47 (t, $^3J_{HH}$ = 5.8 Hz, 1H, OH), 1.13 (d, $^3J_{HH}$ = 6.8 Hz, 3H, H-C10).

^{13}C NMR (126 MHz, $CDCl_3$) δ 156.36 (d, $^1J_{CF}$ = 247.9 Hz, C4), 135.83 (d, $^2J_{CF}$ = 29.6 Hz, C5), 130.67 (q, $^2J_{CF}$ = 32.6 Hz, C8), 125.60 (m, C7), 124.39 (d, $^3J_{CF}$ = 7.2 Hz, C6), 124.12 (q, $^1J_{CF}$ = 272.2 Hz, C9), 110.94 (d, $^2J_{CF}$ = 16.5 Hz, C3), 67.56 (d, $^4J_{CF}$ = 1.7 Hz, C1), 32.95 (d, $^3J_{CF}$ = 3.2 Hz, C2), 17.03 (d, $^4J_{CF}$ = 1.7 Hz, C10).

^{19}F NMR (470 MHz, $CDCl_3$) δ -62.75 (s, 3F, F-C9), -119.53 (d, $^3J_{HF}$ = 37.1 Hz, 1F, F-C4).

^{19}F{^{1}H} NMR (377 MHz, $CDCl_3$) δ -62.75 (s, 3F, F-C9), -119.53 (s, 1F, F-C4).

GC-EI-MS: (*m/z*) requires: [$(C_{12}H_{12}OF_4)^+$] = 248.0819, (*m/z*) found: [$(C_{12}H_{12}OF_4)^+$] = 248.0821.

FT-IR ($\tilde{\nu}$ = cm^{-1}): 3329 (w), 2967 (w), 2933 (w), 2881 (w), 1676 (w), 1619 (w), 1412 (m), 1320 (s), 1165 (m), 1113 (s), 1068 (s), 1010 (s), 941 (w), 832 (m), 763 (w), 672 (m), 626 (m), 591 (m).

Diethyl 2-(2,2-difluoro-2-(4-(trifluoromethyl)phenyl)ethyl)malonate (34)

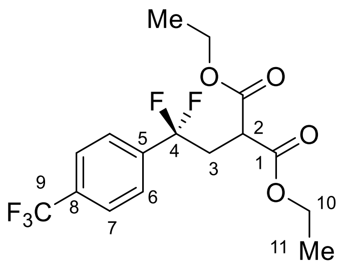

Compound **13** (36.6 mg, 0.10 mmol, 1.0 eq.) and palladium on carbon (10% w/w. 10.6 mg, 0.01 mmol, 10 mol%) were placed in a Schlenk flask. The flask was evacuated and refilled with hydrogen through a balloon. After addition of 1.0 mL of methanol, the mixture was stirred at room temperature for 24 h under balloon pressure of hydrogen. The reaction mixture was filtered through celite and washed with Et_2O. After removal of solvent, the residue was purified by column chromatography (*n*-pentane:Et_2O 15:1) to yield the title compound as a colorless oil (22.8 mg, 0.062 mmol, 62%).

R_f = 0.30 (*n*-pentane:Et_2O 15:1).

^{1}H NMR (400 MHz, $CDCl_3$) δ 7.71 (d, $^3J_{HH}$ = 8.2 Hz, 2H, H-C7), 7.62 (d, $^3J_{HH}$ = 8.4 Hz, 2H, H-C7), 4.27 – 4.10 (m, 4H, H-C10), 3.67 (t, $^3J_{HH}$ = 6.7 Hz, 1H, H-C2), 2.83 (td, $^3J_{HF}$ = 16.8 Hz, $^3J_{HH}$ = 6.7 Hz, 2H, H-C3), 1.26 (t, $^3J_{HH}$ = 7.1 Hz, 6H, H-C11).

^{13}C NMR (126 MHz, $CDCl_3$) δ 168.33 (C1), 139.93 (t, $^2J_{CF}$ = 26.3 Hz, C5), 132.55 (q, $^2J_{CF}$ = 32.6 Hz, C8), 125.81 (m, C6, C7), 123.77 (q, $^1J_{CF}$ = 272.3 Hz, C9), 121.20 (t, $^1J_{CF}$ = 244.1 Hz, C4), 62.17 (C10), 46.61 (t, $^3J_{CF}$ = 3.1 Hz, C2), 38.07 (t, $^2J_{CF}$ = 27.2 Hz, C3), 14.08 (C11).

^{19}F NMR (470 MHz, $CDCl_3$) δ -63.00 (s, 3F, F-C9), -96.84 (t, $^3J_{HF}$ = 16.8 Hz, 2F, F-C4).

^{19}F{^{1}H} NMR (377 MHz, $CDCl_3$) δ -62.87 (s, 3F, F-C9), -96.84 (s, 2F, F-C4).

GC-EI-MS: (*m/z*) requires: [$(C_{16}H_{17}O_4F_4)^+$] = 349.1058, (*m/z*) found: [$(C_{16}H_{17}O_4F_4)^+$] = 349.1056.

FT-IR ($\tilde{\nu}$ = cm^{-1}): 2985 (w), 1733 (s), 1412 (w), 1372 (w), 1326 (s), 1246 (m), 1165 (s), 1131 (s), 1068 (s), 1022 (m), 993 (m), 964 (m), 844 (m), 614 (m), 568 (w).

Diethyl 2-(difluoro(4-(trifluoromethyl)phenyl)methyl)cyclopropane-1,1-dicarboxylate (35)

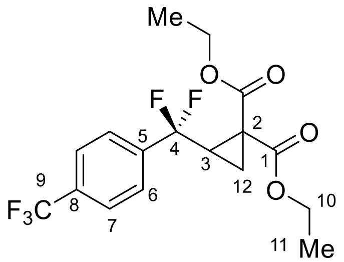

Sodium hydride (60% dispersion in paraffin liquid, 4.8 mg, 0.12 mmol, 1.2 eq.) was suspended in anhydrous DMSO (0.5 mL) under Ar atmosphere. Trimethylsulfoxonium iodide (26.4 mg, 0.12 mmol, 1.2 eq.) was added, and the solution was stirred at room temperature for 0.5 h. Compound **13** (36.6 mg, 0.10 mmol, 1.0 eq.) was added, and the reaction mixture was allowed to stir at room temperature overnight. The solution was poured onto a mixture of ice and water and extracted three times with Et_2O. The combined organic layers were dried over anhydrous Na_2SO_4, filtered, and concentrated under reduced pressure. The crude residue was purified by column chromatography (*n*-pentane:Et_2O 12.5:1) to yield the title compound as a white solid (20.2 mg, 0.053 mmol, 53%).

$\mathbf{R_f}$ = 0.20 (*n*-pentane:Et_2O 12.5:1).

^{1}H NMR (400 MHz, $CDCl_3$) δ 7.80 – 7.61 (m, 4H, H-C6~C7), 4.34 – 4.08 (m, 4H, H-C10), 2.71 – 2.52 (m, 1H, H-C3), 1.97 – 1.86 (m, 1H, H-C12), 1.56 – 1.49 (m, 1H, H-C12), 1.32 – 1.16 (m, 6H, H-C11).

^{13}C NMR (126 MHz, $CDCl_3$) δ 169.13 (C1), 166.03 (C1), 140.23 (C5), 132.62 (C8), 125.87 (t, $^3J_{CF}$ = 5.7 Hz, C6), 125.78 (q, $^3J_{CF}$ = 3.8 Hz, C7), 123.79 (app. q, $^1J_{CF}$ = 272.1 Hz, C9), 118.97 (dd, $^1J_{CF}$ = 244.5, 241.8 Hz, C4), 62.55 (C10), 62.06 (C10), 34.42 (d, $^3J_{CF}$ = 2.5 Hz, C2), 31.83 (t, $^2J_{CF}$ = 30.7 Hz, C3), 16.02 (C12), 14.10 (C11), 13.90 (C11).

^{19}F NMR (470 MHz, $CDCl_3$) δ -62.99 (s, 3F, F-C9), -93.95 (dd, $^2J_{FF}$ = 259.9 Hz, $^3J_{HF}$ = 14.2 Hz, 1F, F-C4), -98.29 (dd, $^2J_{FF}$ = 260.0 Hz, $^3J_{HF}$ = 11.8 Hz, 1F, F-C4).

^{19}F{^{1}H} NMR (377 MHz, $CDCl_3$) δ -62.99 (s, 3F, F-C9), -94.26 (d, $^2J_{FF}$ = 259.4 Hz, 1F, F-C4), -98.37 (d, $^2J_{FF}$ = 259.4 Hz, 1F, F-C4).

GC-EI-MS: (*m/z*) requires: [$(C_{17}H_{17}O_4F_5)^+$] = 380.1042, (*m/z*) found: [$(C_{17}H_{17}O_4F_5)^+$] = 380.1041.

FT-IR ($\tilde{\nu}$ = cm^{-1}): 2990 (w), 1728 (s), 1412 (w), 1372 (w), 1326 (s), 1280 (s), 1246 (m), 1211 (m), 1194 (m), 1119 (s), 1068 (s), 1045 (m), 1027 (m), 1004 (s), 941 (w), 907 (w), 844 (s), 717 (w), 614 (m), 580 (w), 534 (w), 505 (w).

Melting Point: 49-50 ˚C.

1.6 Ring expanding fluorination of cyclopropenes

1-(Oct-1-yn-1-yl)-4-(trifluoromethyl)benzene (Int 17)

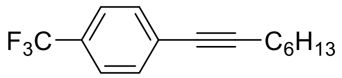

A three-necked flask charged with $Pd(PPh_3)_2Cl_2$ (105.3 mg, 0.15 mmol, 1 mol%) and CuI (85.7 mg, 0.45 mmol, 3 mol%) was evacuated and backfilled with Ar 3 times, NEt_3 (30.0 mL) was added at room temperature under argon. Then, 1-iodo-4-(trifluoromethyl)benzene (4.08 g, 15.00 mmol, 1.0 eq.) and 1-octyne (1.82 g, 16.50 mmol, 1.1 eq.) were added. The reaction mixture was stirred at room temperature overnight. After completion of the reaction, the solvent was removed under reduced pressure, a saturated NH_4Cl solution was added to the resulting residue and the aqueous layer was extracted three times with DCM. The combined organic layers were dried over anhydrous Na_2SO_4, filtered and concentrated under reduced pressure. The crude product was purified by column chromatography to yield the title compound as a pale yellow oil (3.80 g, 14.95 mmol, >99%)

R*f* = 0.80 (*n*-pentane).

^{1}H NMR (400 MHz, $CDCl_3$) δ 7.53 (d, *J* = 8.8 Hz, 2H), 7.48 (d, *J* = 8.3 Hz, 2H), 2.42 (t, *J* = 7.1 Hz, 2H), 1.68 – 1.56 (m, 2H), 1.51 – 1.40 (m, 2H), 1.36 – 1.30 (m, 4H), 0.94 – 0.89 (m, 3H).

^{19}F{^{1}H} NMR (377 MHz, $CDCl_3$) δ -62.73 (s, 3F).

GC-EI-MS: (*m/z*) requires: [$(C_{15}H_{17}F_3)^+$] = 254.1277, (*m/z*) found: [$(C_{15}H_{17}F_3)^+$] = 254.1281.

Analytical data is in agreement with literature values.[20]

1-(2-Hexyl-3-phenyl-3-(trifluoromethyl)cycloprop-1-en-1-yl)-4-(trifluoromethyl)benzene (S36)

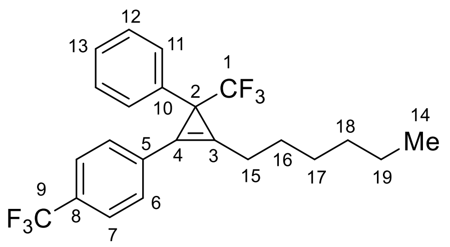

Compound **S36** was prepared according to General Procedure **C** using (1-diazo-2,2,2-trifluoroethyl)benzene (**Int 1**) (372.3 mg, 2.00 mmol, 1.0 eq.) and 1-(oct-1-yn-1-yl)-4-(trifluoromethyl)benzene (**Int 17**) (1.02 g, 4.00 mmol, 2.0 eq.). The crude residue was purified by column chromatography (*n*-pentane) to yield the title compound as a colorless oil (127.0 mg, 0.31 mmol, 15%).

R*f* = 0.65 (*n*-pentane).

^{1}H NMR (400 MHz, $CDCl_3$) δ 7.75 – 7.66 (m, 4H, H-C6~C7), 7.39 (d, $^3J_{HH}$ = 7.7 Hz, 2H, H-C11), 7.36 – 7.25 (m, 3H, H-C12~C13), 2.87 – 2.67 (m, 2H, H-C15), 1.88 – 1.72 (m, 2H, H-C16), 1.49 – 1.39 (m, 2H, H-C17), 1.37 – 1.26 (m, 4H, H-C18~C19), 0.95 – 0.86 (m, 3H, H-C14).

^{13}C NMR (151 MHz, $CDCl_3$) δ 137.94 (C10), 130.99 (q, $^2J_{CF}$ = 32.7 Hz, C8), 129.92 (C5), 129.68 (C6), 128.63 (C12), 127.57 (q, $^4J_{CF}$ = 1.6 Hz, C11), 127.21 (C13), 126.97 (q, $^1J_{CF}$ = 278.0 Hz, C1), 126.10 (q, $^3J_{CF}$ = 3.6 Hz, C7), 124.00 (q, $^1J_{CF}$ = 272.3 Hz, C9), 117.37 (C3), 107.16 (C4), 33.94 (app. q, $^2J_{CF}$ = 34.9 Hz, C2), 31.57 (*C*H_2), 29.20 (C17), 27.52 (C16), 24.84 (C15), 22.65 (*C*H_2), 14.16 (C14).

^{19}F NMR (564 MHz, $CDCl_3$) δ -62.82 (s, 3F, CF_3), -62.84 (s, 3F, CF_3).

^{19}F{^{1}H} NMR (564 MHz, $CDCl_3$) δ -62.82 (s, 3F, CF_3), -62.84 (s, 3F, CF_3).

GC-EI-MS: (*m/z*) requires: [$(C_{23}H_{22}F_6)^+$] = 412.1620, (*m/z*) found: [$(C_{23}H_{22}F_6)^+$] = 412.1618.

FT-IR ($\tilde{\nu}$ = cm^{-1}): 2933 (w), 2864 (w), 1613 (w), 1498 (w), 1464 (w), 1412 (w), 1320 (s), 1297 (m), 1246 (m), 1159 (s), 1125 (s), 1068 (s), 1016 (m), 924 (m), 843 (m), 700 (s), 649 (m), 603 (m).

Methyl 4-(oct-1-yn-1-yl)benzoate (Int 18)

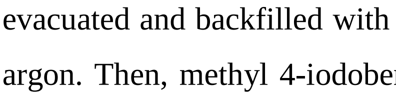

A three-necked flask charged with $Pd(PPh_3)_2Cl_2$ (93.8 mg, 0.13 mmol, 1 mol%) and CuI (76.3 mg, 0.40 mmol, 3 mol%) was evacuated and backfilled with Ar 3 times, NEt_3 (27.0 mL) was added at room temperature under argon. Then, methyl 4-iodobenzoate (3.50 g, 13.36 mmol, 1.0 eq.) and 1-octyne (1.62 g, 14.70 mmol, 1.1 eq.) were added. The reaction mixture was stirred at room temperature overnight. After

completion of the reaction, the solvent was removed under reduced pressure, a saturated NH_4Cl solution was added to the resulting residue and the aqueous layer was extracted three times with DCM. The combined organic layers were dried over anhydrous Na_2SO_4, filtered and concentrated under reduced pressure. The crude product was purified by column chromatography to yield the title compound as a pale yellow oil (2.93 g, 11.99 mmol, 90%)

R_f = 0.40 (*n*-pentane:Et_2O 30:1).

^{1}H NMR (400 MHz, $CDCl_3$) δ 7.99 – 7.91 (m, 2H), 7.44 (d, *J* = 8.3 Hz, 2H), 3.91 (s, 3H), 2.42 (t, *J* = 7.1 Hz, 2H), 1.61 (p, *J* = 7.2 Hz, 2H), 1.45 (p, *J* = 6.9 Hz, 2H), 1.37 – 1.25 (m, 4H), 0.99 – 0.85 (m, 3H).

ESI-MS: (*m/z*) requires: $[(C_{16}H_{20}O_2Na)^+]$ = 267.1356, (*m/z*) found: $[(C_{16}H_{20}O_2Na)^+]$ = 267.1356.

Analytical data is in agreement with literature values.[21]

Methyl 4-(2-hexyl-3-phenyl-3-(trifluoromethyl)cycloprop-1-en-1-yl)benzoate (S37)

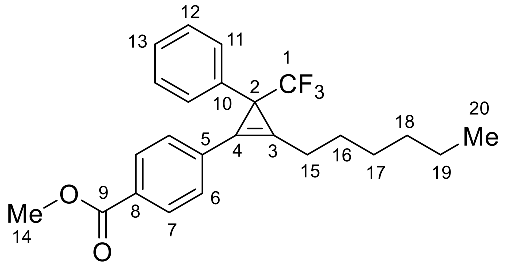

Compound **S37** was prepared according to General Procedure **C** using (1-diazo-2,2,2-trifluoroethyl)benzene (**Int 1**) (558.4 mg, 3.00 mmol, 1.0 eq.) and methyl 4-(oct-1-yn-1-yl)benzoate (**Int 18**) (1.47 g, 6.00 mmol, 2.0 eq.). The crude residue was purified by column chromatography (*n*-pentane:Et_2O 30:1) to yield the title compound as a colorless oil (293.6 mg, 0.73 mmol, 24%).

R_f = 0.35 (*n*-pentane:Et_2O 30:1).

^{1}H NMR (500 MHz, $CDCl_3$) δ 8.12 – 8.05 (m, 2H, H-C7), 7.66 – 7.59 (m, 2H, H-C6), 7.36 (d, $^3J_{HH}$ = 7.7 Hz, 2H, H-C11), 7.32 – 7.27 (m, 2H, H-C12), 7.26 – 7.22 (m, 1H, H-C13), 3.94 (s, 3H, H-C14), 2.82 – 2.67 (m, 2H, H-C15), 1.83 – 1.71 (m, 2H, H-C16), 1.47 – 1.36 (m, 2H, H-C17), 1.35 – 1.26 (m, 4H, H-C18~C19), 0.92 – 0.85 (m, 3H, H-C20).

^{13}C NMR (126 MHz, $CDCl_3$) δ 166.61 (C9), 138.08 (C10), 130.69 (C8), 130.54 (C5), 130.32 (C7), 129.37 (C6), 128.58 (C12), 127.63 (q, $^4J_{CF}$ = 1.6 Hz, C11), 127.15 (C13), 127.00 (q, $^1J_{CF}$ = 278.2 Hz, C1), 117.42 (C3), 107.51 (C4), 52.43 (C14), 33.93 (app. q, $^2J_{CF}$ = 34.7 Hz, C2), 31.57 (*C*H_2), 29.21 (C17), 27.51 (C16), 24.90 (C15), 22.65 (*C*H_2), 14.16 (C20).

^{19}F NMR (470 MHz, $CDCl_3$) δ -62.79 (s, 3F, F-C1).

^{19}F{^{1}H} NMR (470 MHz, $CDCl_3$) δ -62.79 (s, 3F, F-C1).

ESI-MS: (*m/z*) requires: [$(C_{24}H_{25}O_2F_3Na)^+$] = 425.1699, (*m/z*) found: [$(C_{24}H_{25}O_2F_3Na)^+$] = 425.1698.

FT-IR ($\tilde{\nu}$ = cm^{-1}): 2933 (w), 2858 (w), 1722 (s), 1607 (w), 1498 (w), 1435 (m), 1406 (w), 1274 (s), 1159 (s), 1108 (s), 1016 (w), 964 (w), 861 (m), 827 (w), 769 (m), 735 (m), 700 (s), 649 (m), 614 (w), 585 (w).

1-Methyl-4-(2-methyl-1-(trifluoromethyl)-3-(4-(trifluoromethyl)phenyl)cycloprop-2-en-1-yl)benzene (S38)

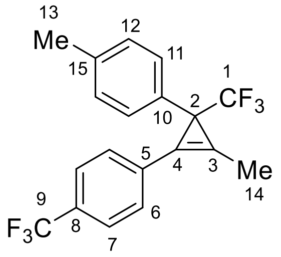

Compound **S38** was prepared according to General Procedure **C** using 1-(1-diazo-2,2,2-trifluoroethyl)-4-methylbenzene (**Int 4**) and 1-(prop-1-yn-1-yl)-4-(trifluoromethyl)benzene. The crude residue was purified by column chromatography to yield the title compound as a white wax.

R*f* = 0.43 (*n*-pentane).

^{1}H NMR (400 MHz, $CDCl_3$) δ 7.72 – 7.62 (m, 4H, H-C6~C7), 7.26 (d, $^{3}J_{HH}$ = 7.9 Hz, 2H, H-C11), 7.12 (d, $^{3}J_{HH}$ = 8.0 Hz, 2H, H-C12), 2.42 (s, 3H, H-C14), 2.32 (s, 3H, H-C13).

^{13}C NMR (126 MHz, $CDCl_3$) δ 137.12 (C10), 134.75 (C15), 130.96 (q, $^{2}J_{CF}$ = 32.5 Hz, C8), 130.01 (C5), 129.60 (C6), 129.39 (C12), 127.65 (q, $^{4}J_{CF}$ = 1.4 Hz, C11), 126.96 (q, $^{1}J_{CF}$ = 278.3 Hz, C1), 126.05 (q, $^{3}J_{CF}$ = 3.8 Hz, C7), 124.00 (q, $^{1}J_{CF}$ = 272.5 Hz, C9), 113.28 (C3), 108.28 (C4), 33.56 (app. q, $^{2}J_{CF}$ = 34.5 Hz, C2), 21.17 (C13), 9.63 (C14).

^{19}F NMR (470 MHz, $CDCl_3$) δ -62.83 (s, 3F, CF_3), -63.37 (s, 3F, CF_3).

^{19}F{^{1}H} NMR (377 MHz, $CDCl_3$) δ -62.83 (s, 3F, CF_3), -63.38 (s, 3F, CF_3).

GC-EI-MS: (*m/z*) requires: [$(C_{19}H_{14}F_6)^+$] = 356.0994, (*m/z*) found: [$(C_{19}H_{14}F_6)^+$] = 356.0994.

FT-IR ($\tilde{\nu}$ = cm^{-1}): 2927 (w), 2365 (w), 1860 (w), 1613 (w), 1515 (w), 1412 (w), 1320 (s), 1297 (m), 1246 (m), 1154 (s), 1108 (s), 1062 (s), 1016 (m), 918 (m), 838 (s), 740 (m), 729 (m), 700 (w), 683 (w), 585 (m).

1-Fluoro-2-hexyl-3-(trifluoromethyl)-1-(4-(trifluoromethyl)phenyl)-1H-indene (36)

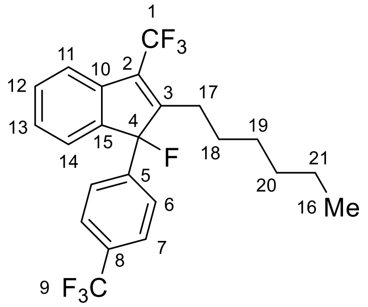

Compound **36** was prepared according to the General Procedure **F** with an amine:HF ratio of 1:9.2 using 1-(2-hexyl-3-phenyl-3-(trifluoromethyl)cycloprop-1-en-1-yl)-4-(trifluoromethyl)benzene (**S36**) (41.2 mg, 0.10 mmol, 1.0 eq.) and aryl iodide **C7** (8.8 mg, 0.02 mmol, 20 mol%). The solvent was changed to DCM. The crude product was purified by column chromatography (*n*-pentane) yielded the title compound **36** as a colorless oil (23.8 mg, 0.055 mmol, 55%).

R_f = 0.85 (*n*-pentane).

^{1}H NMR (400 MHz, $CDCl_3$) δ 7.61 (d, $^3J_{HH}$ = 8.2 Hz, 2H, H-C7), 7.43 – 7.34 (m, 4H, H-C6, H-C11~C12), 7.21 (td, $^3J_{HH}$ = 7.3 Hz, $^5J_{HF}$ = 1.6 Hz, 1H, H-C13), 7.13 (d, $^3J_{HH}$ = 7.4 Hz, 1H, H-C14), 2.52 – 2.39 (m, 1H, H-C18), 2.22 – 2.07 (m, 1H, H-C18), 1.52 – 1.39 (m, 1H, H-C17), 1.28 – 1.11 (m, 7H, H-C17, H-C19~C21), 0.83 (t, $^3J_{HH}$ = 6.9 Hz, 3H, H-C16).

^{13}C NMR (151 MHz, $CDCl_3$) δ 154.09 (C3), 143.83 (d, $^2J_{CF}$ = 18.2 Hz, C15), 141.61 (d, $^2J_{CF}$ = 27.4 Hz, C5), 138.20 (C10), 130.93 (qd, $^2J_{CF}$ = 32.6 Hz, $^5J_{CF}$ = 1.0 Hz, C8), 130.64 (C12), 130.07 (C2), 127.87 (d, $^4J_{CF}$ = 1.5 Hz, C13), 125.91 (qd, $^3J_{CF}$ = 3.8 Hz, $^4J_{CF}$ = 1.8 Hz, C7), 125.35 (d, $^3J_{CF}$ = 8.7 Hz, C6), 124.36 (C14), 124.02 (q, $^1J_{CF}$ = 272.1 Hz, C9), 123.03 (q, $^1J_{CF}$ = 272.5 Hz, C1), 121.32 (q, $^4J_{CF}$ = 1.8 Hz, C11), 104.22 (d, $^1J_{CF}$ = 188.6 Hz, C4), 31.33 (CH_2), 29.58 (CH_2), 28.98 (q, $^4J_{CF}$ = 1.6 Hz, C17), 25.90 (C18), 22.50 (CH_2), 14.09 (C16).

^{19}F NMR (564 MHz, $CDCl_3$) δ -61.35 – -61.39 (m, 3F, F-C1), -62.78 (s, 3F, F-C9), -176.61 (s, 1F, F-C4).

^{19}F{^{1}H} NMR (377 MHz, $CDCl_3$) δ -61.33 (d, $^5J_{FF}$ = 2.4 Hz, 3F, F-C1), -62.71 (s, 3F, F-C9), -176.61 (s, 1F, F-C4).

GC-EI-MS: (*m/z*) requires: [$(C_{23}H_{21}F_7)^+$] = 430.1526, (*m/z*) found: [$(C_{23}H_{21}F_7)^+$] = 430.1521.

FT-IR ($\tilde{\nu}$ = cm^{-1}): 2933 (w), 2870 (w), 1619 (w), 1464 (w), 1412 (w), 1378 (m), 1326 (s), 1165 (s), 1119 (s), 1068 (s), 1016 (m), 976 (w), 941 (m), 838 (m), 752 (m), 700 (m), 666 (w).

Methyl 4-(1-fluoro-2-hexyl-3-(trifluoromethyl)-1H-inden-1-yl)benzoate (37)

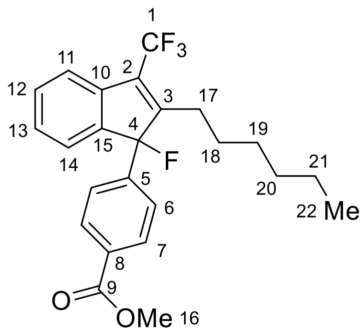

Compound **37** was prepared according to the General Procedure **F** with an amine:HF ratio of 1:8.5 using methyl 4-(2-hexyl-3-phenyl-3-(trifluoromethyl)cycloprop-1-en-1-yl)benzoate (**S37**) (40.3 mg, 0.10 mmol, 1.0 eq.) and aryl iodide **C7** (8.8 mg, 0.02 mmol, 20 mol%). The solvent was changed to DCM. The crude product was purified by column chromatography (*n*-pentane: Et_2O 40:1) yielded the title compound **37** as a colorless oil (22.7 mg, 0.054 mmol, 54%).

$\mathbf{R}_{\boldsymbol{f}}$ = 0.40 (*n*-pentane: Et_2O 30:1).

^{1}H NMR (400 MHz, $CDCl_3$) δ 8.02 (d, $^3J_{HH}$ = 8.2 Hz, 2H, H-C7), 7.46 – 7.31 (m, 4H, H-C6, H-C11~C12), 7.20 (td, $^3J_{HH}$ = 7.3 Hz, $^5J_{HF}$ = 1.4 Hz, 1H, H-C13), 7.12 (d, $^3J_{HH}$ = 7.3 Hz, 1H, H-C14), 3.92 (s, 3H, H-C16), 2.51 – 2.39 (m, 1H, H-C18), 2.22 – 2.09 (m, 1H, H-C18), 1.53 – 1.37 (m, 1H, H-C17), 1.27 – 1.10 (m, 7H, H-C17, H-C19~C21), 0.83 (t, $^3J_{HH}$ = 6.9 Hz, 3H, H-C22).

^{13}C NMR (126 MHz, $CDCl_3$) δ 166.67 (C9), 154.22 (C3), 143.95 (d, $^2J_{CF}$ = 18.3 Hz, C15), 142.48 (d, $^2J_{CF}$ = 27.1 Hz, C5), 138.23 (C10), 130.54 (C12), 130.50 (d, $^5J_{CF}$ = 0.7 Hz, C8), 130.18 (d, $^4J_{CF}$ = 1.7 Hz, C7), 129.95 (C2), 127.80 (d, $^4J_{CF}$ = 1.5 Hz, C13), 124.93 (d, $^3J_{CF}$ = 8.5 Hz, C6), 124.34 (C14), 123.06 (q, $^1J_{CF}$ = 272.3 Hz, C1), 121.26 (q, $^4J_{CF}$ = 1.7 Hz, C11), 104.37 (d, $^1J_{CF}$ = 188.6 Hz, C4), 52.37 (C16), 31.32 (CH_2), 29.61 (CH_2), 28.97 (C17), 25.91 (C18), 22.54 (CH_2), 14.10 (C22).

^{19}F NMR (470 MHz, $CDCl_3$) δ -61.29 – -61.34 (m, 3F, F-C1), -176.33 (s, 1F, F-C4).

^{19}F{^{1}H} NMR (377 MHz, $CDCl_3$) δ -61.31 (d, $^5J_{FF}$ = 2.4 Hz, 3F, F-C1), -176.32 (q, $^5J_{FF}$ = 2.4 Hz, 1F, F-C4).

ESI-MS: (*m/z*) requires: $[(C_{24}H_{24}O_2F_4Na)^+]$ = 443.1605, (*m/z*) found: $[(C_{24}H_{24}O_2F_4Na)^+]$ = 443.1602.

FT-IR ($\tilde{\nu}$ = cm^{-1}): 2956 (w), 2933 (w), 2870 (w), 1728 (s), 1613 (w), 1458 (w), 1435 (w), 1406 (w), 1378 (m), 1274 (s), 1159 (s), 1119 (s), 1050 (m), 1022 (m), 970 (m), 941 (w), 855 (m), 758 (s), 706 (m), 654 (w).

1-Fluoro-2,6-dimethyl-3-(trifluoromethyl)-1-(4-(trifluoromethyl)phenyl)-1H-indene (38)

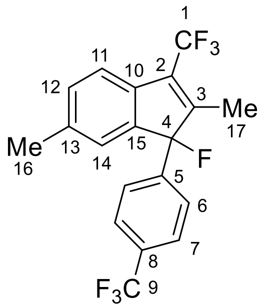

Compound **38** was prepared according to the General Procedure **F** with an amine:HF ratio of 1:7.0 using 1-methyl-4-(2-methyl-1-(trifluoromethyl)-3-(4-(trifluoromethyl)phenyl)cycloprop-2-en-1-yl)benzene (**S38**) (35.6 mg, 0.10 mmol, 1.0 eq.) and aryl iodide **C7** (8.8 mg, 0.02 mmol, 20 mol%). The solvent was changed to DCM. The crude product was purified by column chromatography (*n*-pentane) yielded the title compound **38** as a colorless oil (16.7 mg, 0.045 mmol, 45%).

$\mathbf{R}_f$ = 0.60 (*n*-pentane).

^{1}H NMR (400 MHz, $CDCl_3$) δ 7.62 (d, $^3J_{HH}$ = 8.2 Hz, 2H, H-C7), 7.38 (d, $^3J_{HH}$ = 8.2 Hz, 2H, H-C7), 7.28 (d, $^3J_{HH}$ = 7.8 Hz, 1H, H-C11), 7.17 (d, $^3J_{HH}$ = 7.8 Hz, 1H, H-C12), 6.97 (s, 1H, H-C14), 2.30 (s, 3H, H-C16), 1.93 (q, $^5J_{HF}$ = 2.9 Hz, 3H, H-C17).

^{13}C NMR (126 MHz, $CDCl_3$) δ 148.79 (C3), 143.91 (d, $^2J_{CF}$ = 18.1 Hz, C15), 141.64 (d, $^2J_{CF}$ = 27.9 Hz, C5), 137.97 (d, $^4J_{CF}$ = 1.6 Hz, C13), 135.46 (C10), 131.07 (C12), 130.88 (qd, $^2J_{CF}$ = 32.7 Hz, $^5J_{CF}$ = 1.1 Hz, C8), 129.95 (C2), 125.98 (qd, $^3J_{CF}$ = 3.8 Hz, $^4J_{CF}$ = 1.7 Hz, C7), 125.45 (C14), 125.24 (d, $^3J_{CF}$ = 8.6 Hz, C6), 124.04 (q, $^1J_{CF}$ = 272.4 Hz, C9), 123.05 (q, $^1J_{CF}$ = 272.7 Hz, C1), 120.96 (q, $^4J_{CF}$ = 1.6 Hz, C11), 103.48 (d, $^1J_{CF}$ = 188.5 Hz, C4), 21.44 (C16), 10.48 (q, $^4J_{CF}$ = 1.6 Hz, C17).

^{19}F NMR (470 MHz, $CDCl_3$) δ -61.32 – -61.37 (m, 3F, F-C1), -62.74 (s, 3F, F-C9), -176.74 (s, 1F, F-C4).

^{19}F{^{1}H} NMR (377 MHz, $CDCl_3$) δ -61.34 (d, $^5J_{FF}$ = 2.4 Hz, 3F, F-C1), -62.74 (s, 3F, F-C9), -176.73 (q, $^5J_{FF}$ = 2.4 Hz, 1F, F-C4).

GC-EI-MS: (*m/z*) requires: [$(C_{19}H_{13}F_7)^+$] = 374.0900, (*m/z*) found: [$(C_{19}H_{13}F_7)^+$] = 374.0899.

FT-IR ($\tilde{\nu}$ = cm^{-1}): 1619 (w), 1412 (w), 1372 (m), 1326 (s), 1217 (m), 1159 (s), 1113 (s), 1068 (s), 1022 (m), 890 (w), 844 (m), 821 (m), 758 (w), 706 (w), 660 (m), 603 (w).

1.7 X-ray crystallographic data

X-Ray diffraction: Data sets for compound **3** were collected with a Bruker D8 Venture Photon III Diffractometer. Programs used: data collection: *APEX6* Version 2024.9-0 [22] (Bruker AXS Inc., **2024**); cell refinement: *SAINT* Version 8.41 (Bruker AXS Inc., **2024**); data reduction: *SAINT* Version 8.41 (Bruker AXS Inc., **2024**); absorption correction, *SADABS* Version 2016/2 (Bruker AXS Inc., **2024**); structure solution *SHELXT*-Version 2018-3 [23] (G. M. Sheldrick, *Acta Cryst.*, **2015**, *A71*, 3-8); structure refinement *SHELXL*- Version 2019-2 [24] (G. M. Sheldrick, *Acta Cryst.*, **2015**, *C71*, 3-8) and graphics, *XP* [25] (Version 5.1, Bruker AXS Inc., Madison, Wisconsin, USA, **1998**). *R*-values are given for observed reflections, and wR^2 values are given for all reflections.

X-ray crystal structure analysis of 3 (gil10840): A colourless, plate shaped specimen of $C_{17}H_9ClF_8$, approximate dimensions 0.037×0.091×0.117 mm^3, was used for the X-ray crystallographic analysis. The crystal was crystallised from diethyl ether. The X-ray intensity data of **3** were measured on a Bruker D8 VENTURE KAPPA diffractometer system equipped with a microfocus sealed tube (λ = 0.71073 Å) and a multilayer mirror monochromator. The specimen was held at 100(2) K during the measurement with an Oxford Cryostream 1000 low temperature device. A total of 1672 frames were collected. The total exposure time was 3.84 hours. The frames were integrated with the SAINT V8.41 package using a narrow-frame algorithm. The integration of the data using a monoclinic unit cell yielded a total of 54695 reflections to a maximum θ angle of 27.48° (0.77 Å resolution), of which 3617 were independent (average redundancy 15.12, completeness = 99.9%, Rint = 3.86%, Rsig = 1.71%) and 3240 (89.6%) were greater than $2\sigma(F^2)$. The final cell constants of a = 14.1881(5) Å, b = 7.7695(2) Å, c = 14.3398(5) Å, volume = 1580.57(9) Å^3, are based upon the refinement of the XYZ-centroids of 9713 reflections above 20 $\sigma(I)$ with 2.84° < 2θ < 27.47°. Data were corrected for absorption effects using the Multi-Scan method in SADABS 2016/2. The calculated minimum and maximum transmission coefficients (based on crystal size) are 0.963 and 0.988. The structure was solved by SHELXT 2018/2 and refined using the SHELXL-2019/2 Software, in the space group $P2_1/c$ (14), with Z = 4 for the formula unit $C_{17}H_9ClF_8$. The final anisotropic full-matrix least-squares refinement on F^2 with 235 variables against 3617 data points converged at R_1 = 2.95%, for the observed data and wR_2 = 8.33% for all data. The goodness-of-fit on F^2 was 1.07. The largest peak in the final difference electron density synthesis was 0.34 e$^-$/Å^3 and the deepest hole was −0.29 e$^-$/Å^3 with an

RMS deviation of 0.046 e⁻/Å^3. On the basis of the final model, the calculated density was 1.68 g/cm^3 and *F*(000), 800 e⁻. CCDC Nr.: 2479575.

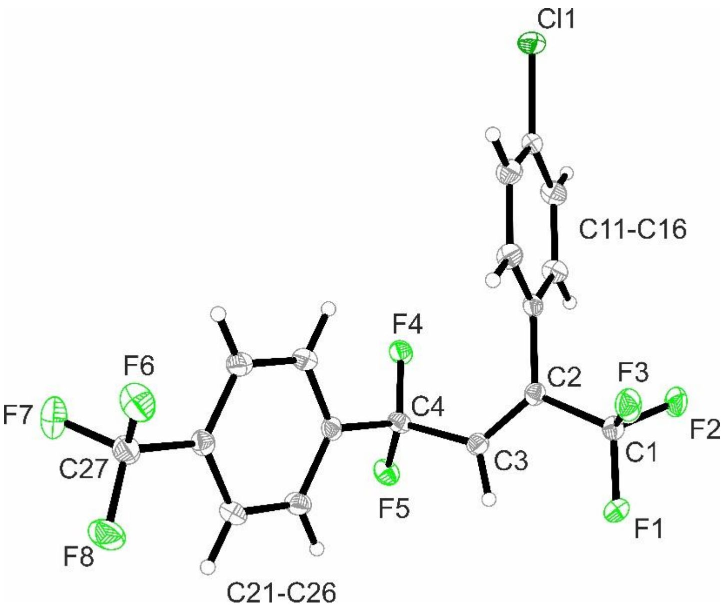

Figure S1. Crystal structure of compound **3**. Thermal ellipsoids are shown at 50% probability.

1.8 NMR-Spectra of key compounds

1-Bromo-4-(1-(trifluoromethyl)-2-(4-(trifluoromethyl)phenyl)cycloprop-2-en-1-yl)benzene (S2)

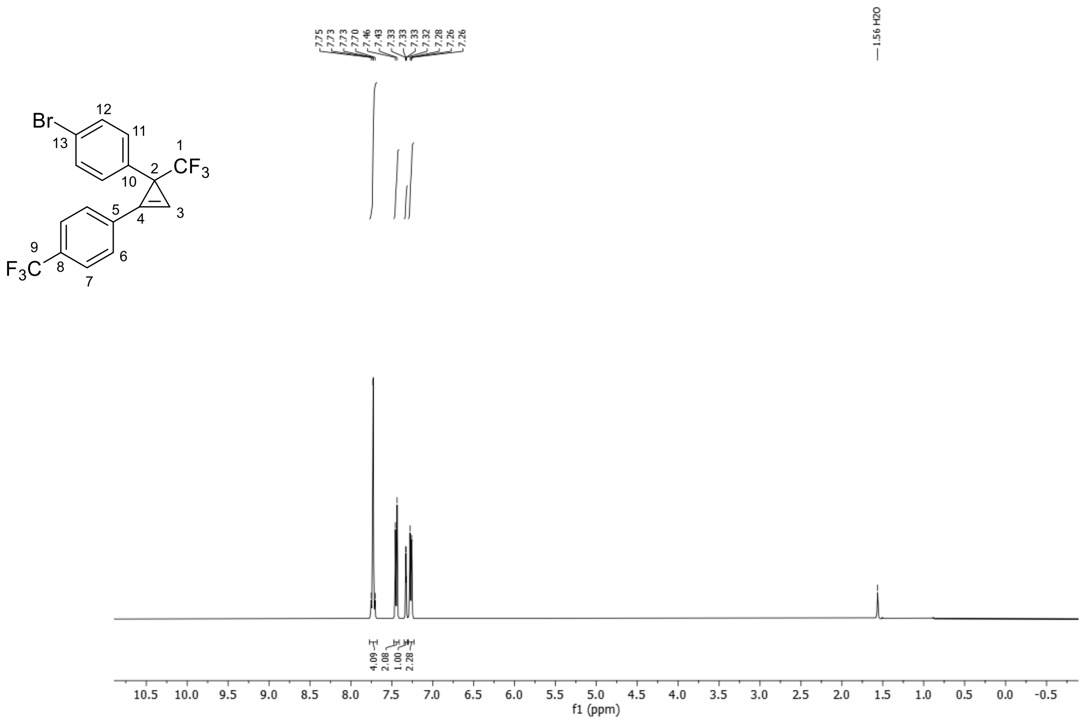

Figure S2. ^{1}H NMR of **S2** (400 MHz, 299 K, $CDCl_3$).

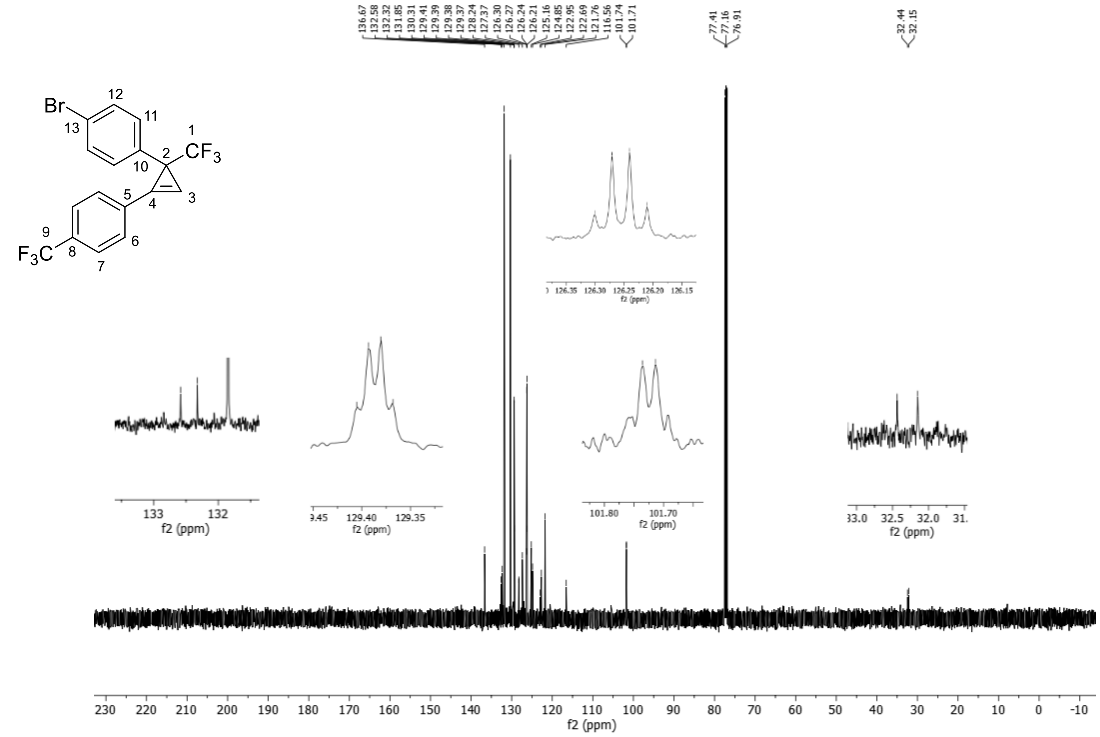

Figure S3. $^{13}C\{^{1}H\}$ NMR of **S2** (126 MHz, 299 K, $CDCl_3$).

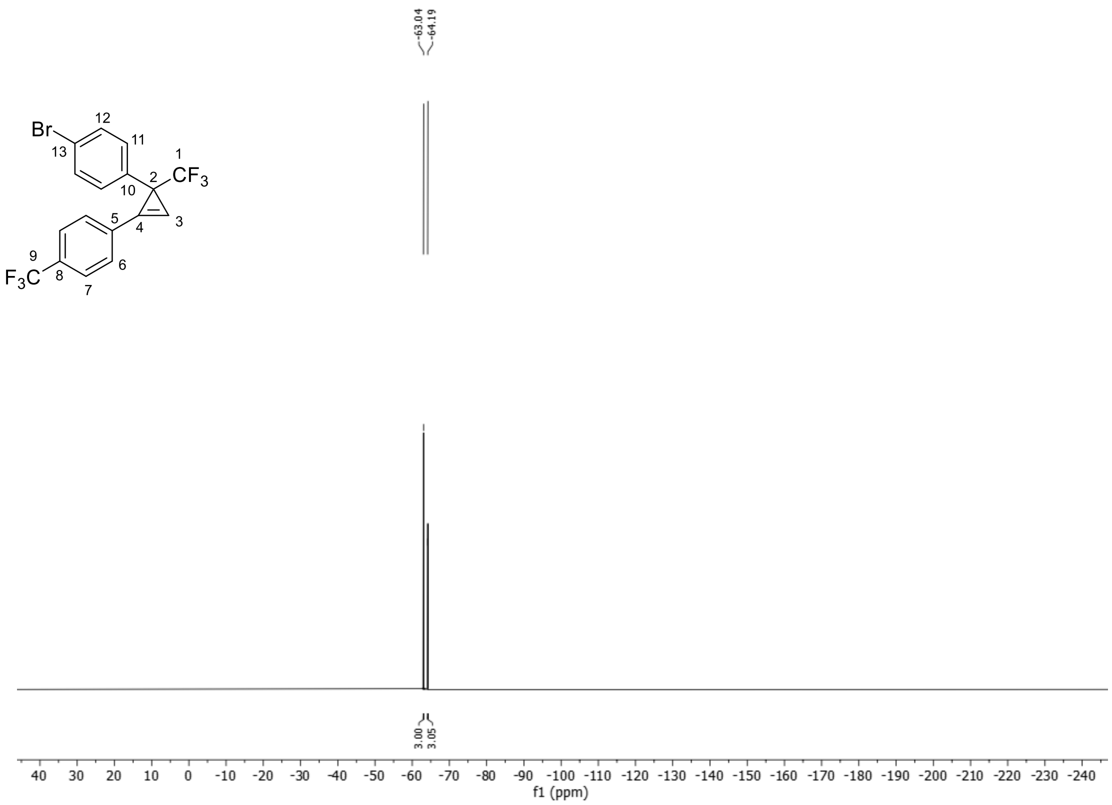

Figure S4. ^{19}F NMR of **S2** (470 MHz, 299 K, $CDCl_3$).

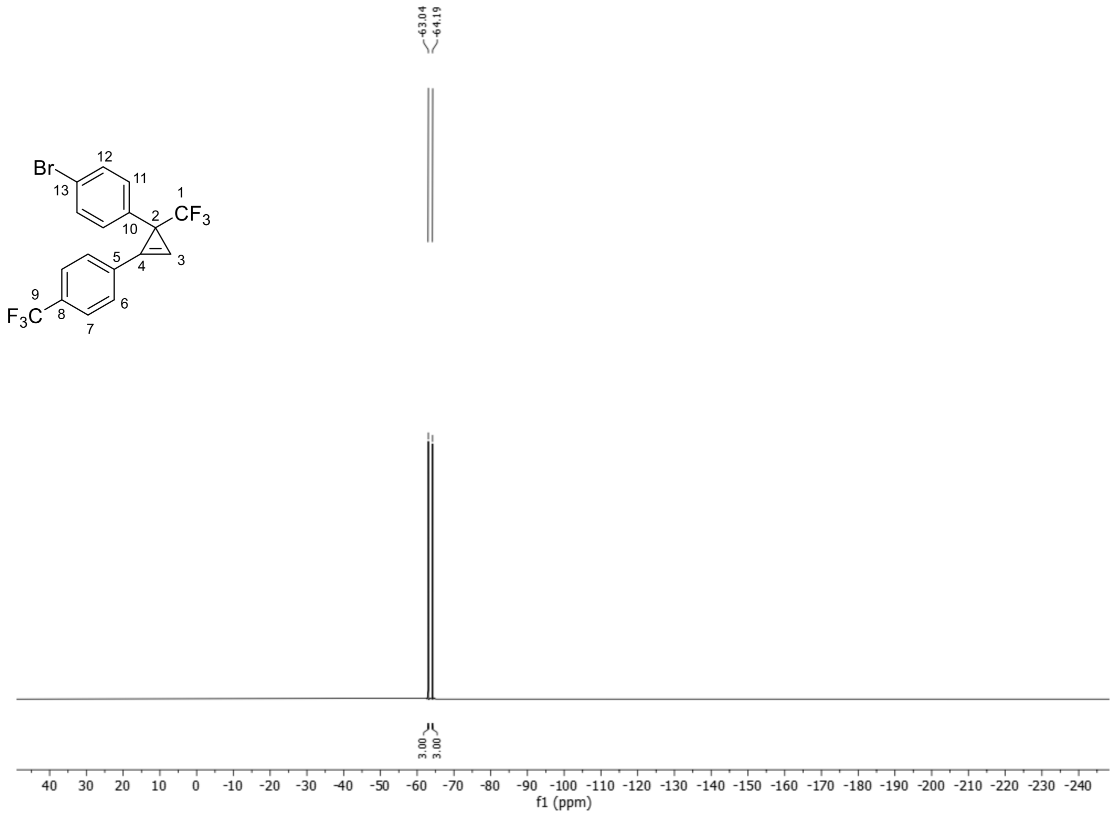

Figure S5. ^{19}F{^{1}H} NMR of **S2** (377 MHz, 299 K, $CDCl_3$).

1-Chloro-4-(1-(trifluoromethyl)-2-(4-(trifluoromethyl)phenyl)cycloprop-2-en-1-yl)benzene (S3)

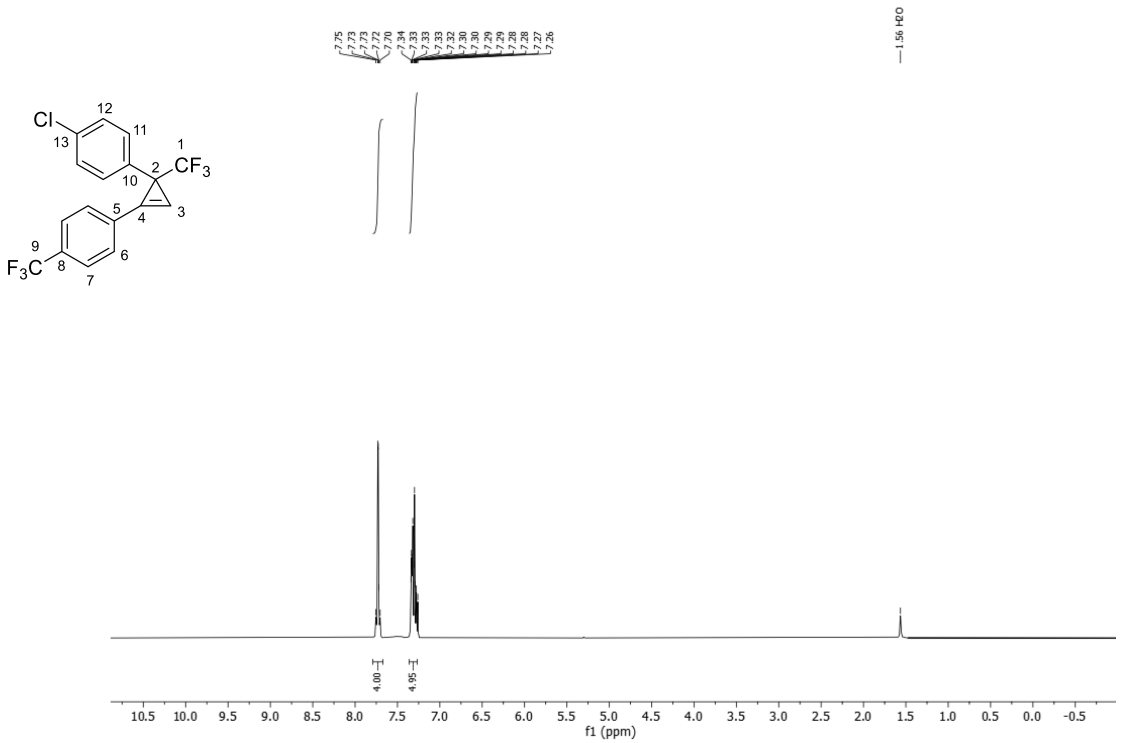

Figure S6. ^{1}H NMR of **S3** (400 MHz, 299 K, $CDCl_3$).

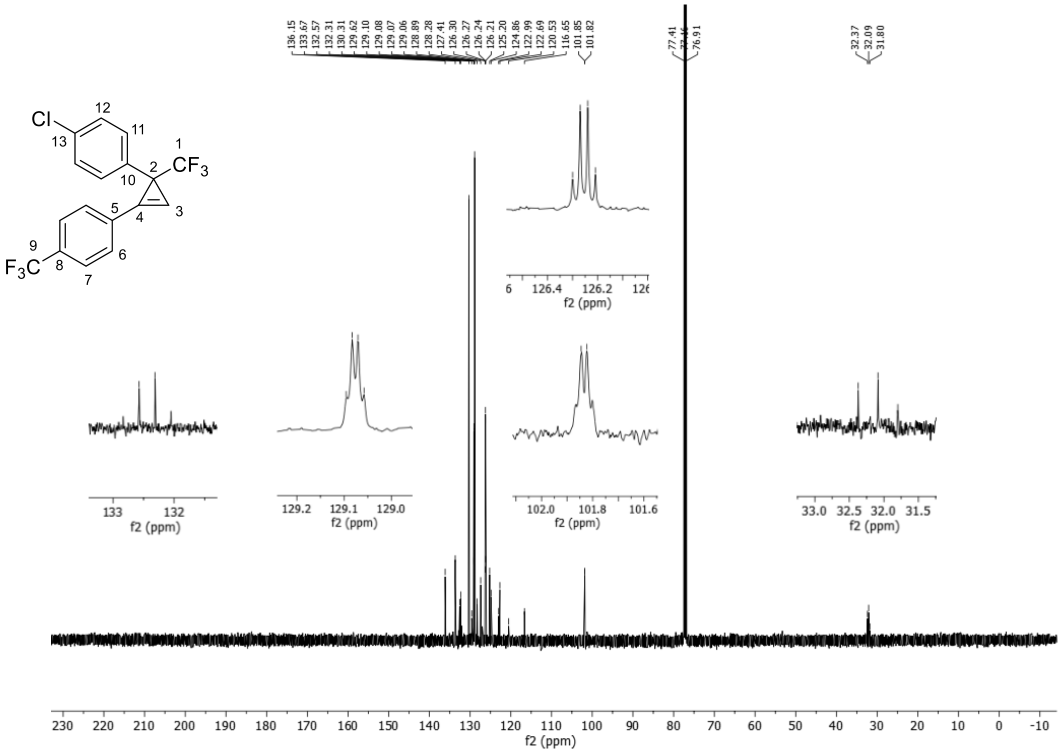

Figure S7. $^{13}C\{^{1}H\}$ NMR of **S3** (126 MHz, 299 K, $CDCl_3$).

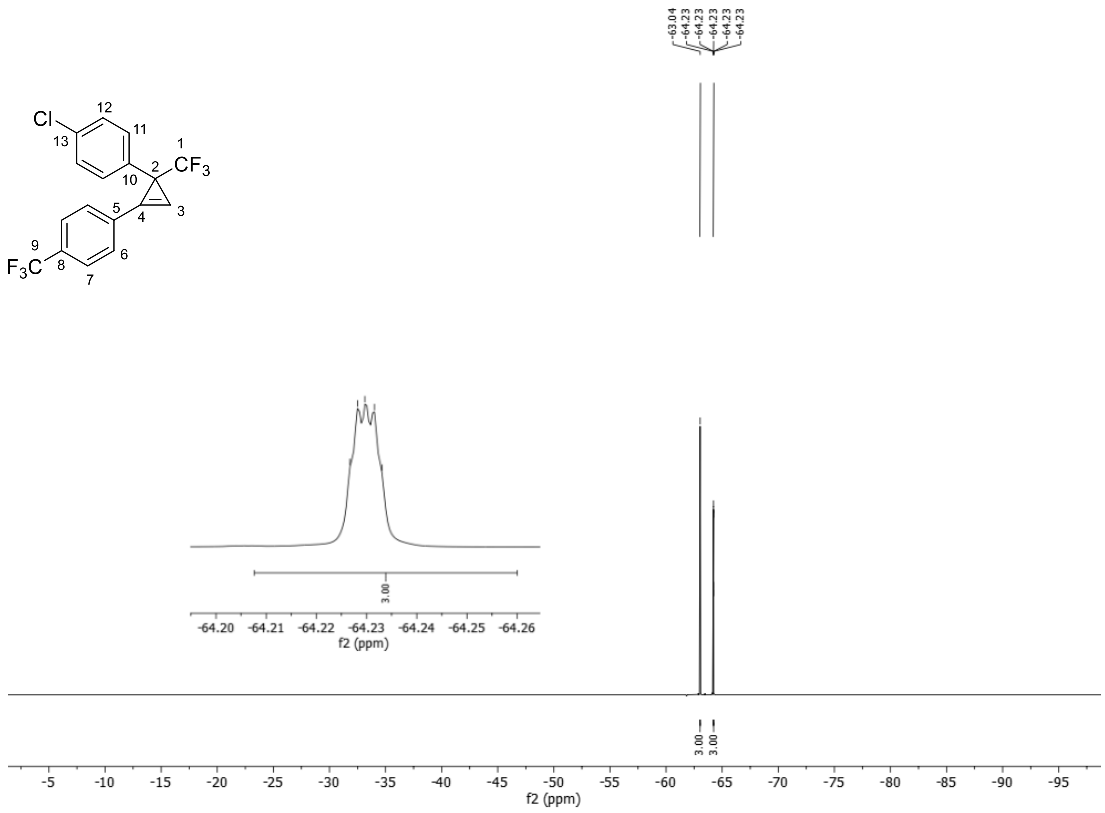

Figure S8. ^{19}F NMR of **S3** (470 MHz, 299 K, $CDCl_3$).

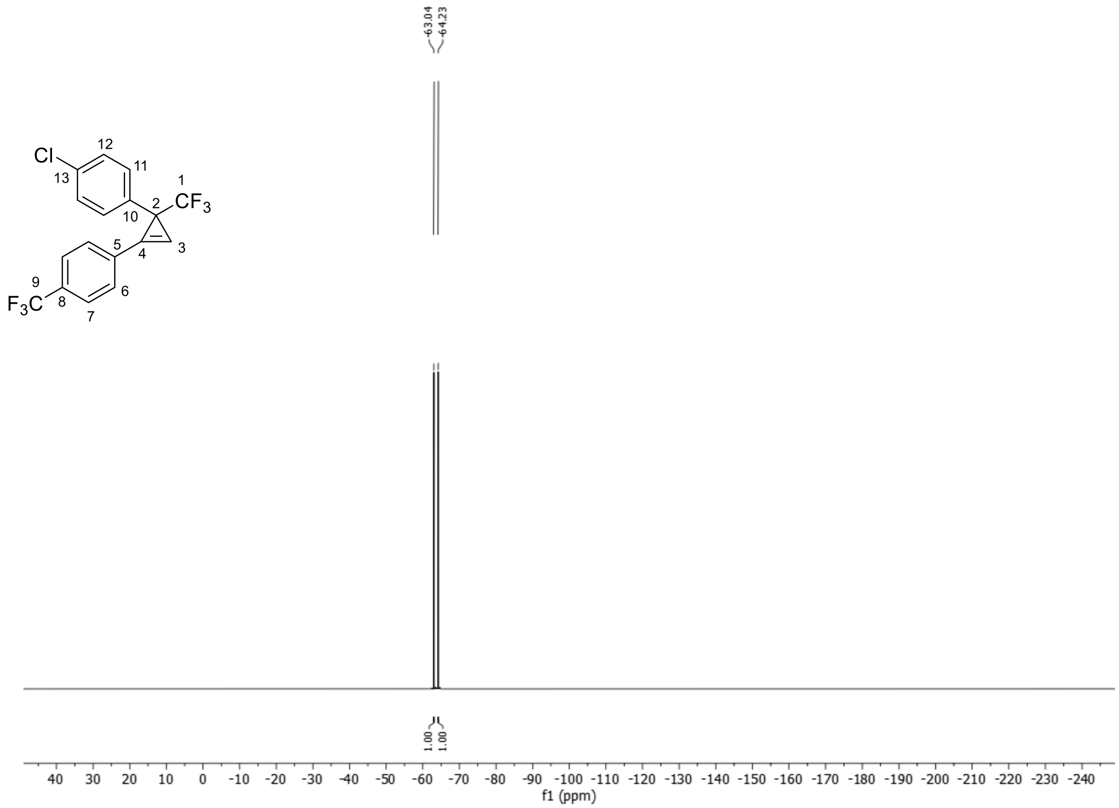

Figure S9. $^{19}F\{^1H\}$ NMR of **S3** (377 MHz, 299 K, $CDCl_3$).

4,4'-(1-(Trifluoromethyl)cycloprop-2-ene-1,2-diyl)bis((trifluoromethyl)benzene) (S4)

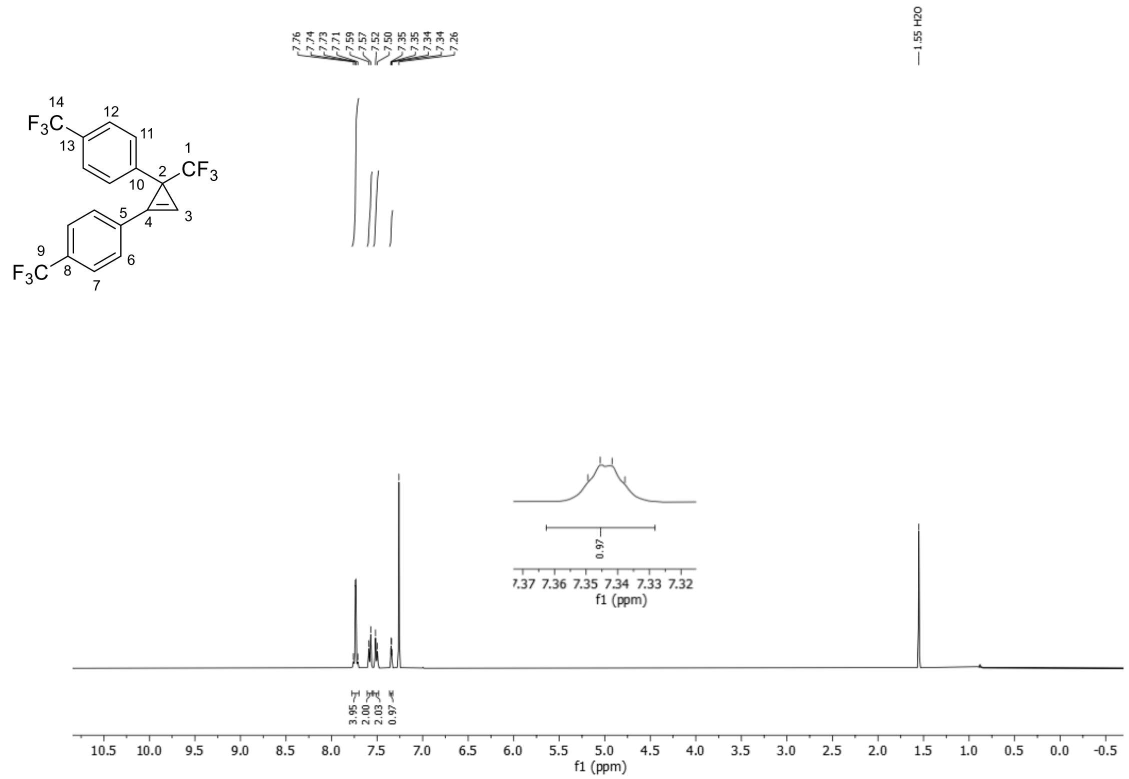

Figure S10. ^{1}H NMR of **S4** (400 MHz, 299 K, $CDCl_3$).

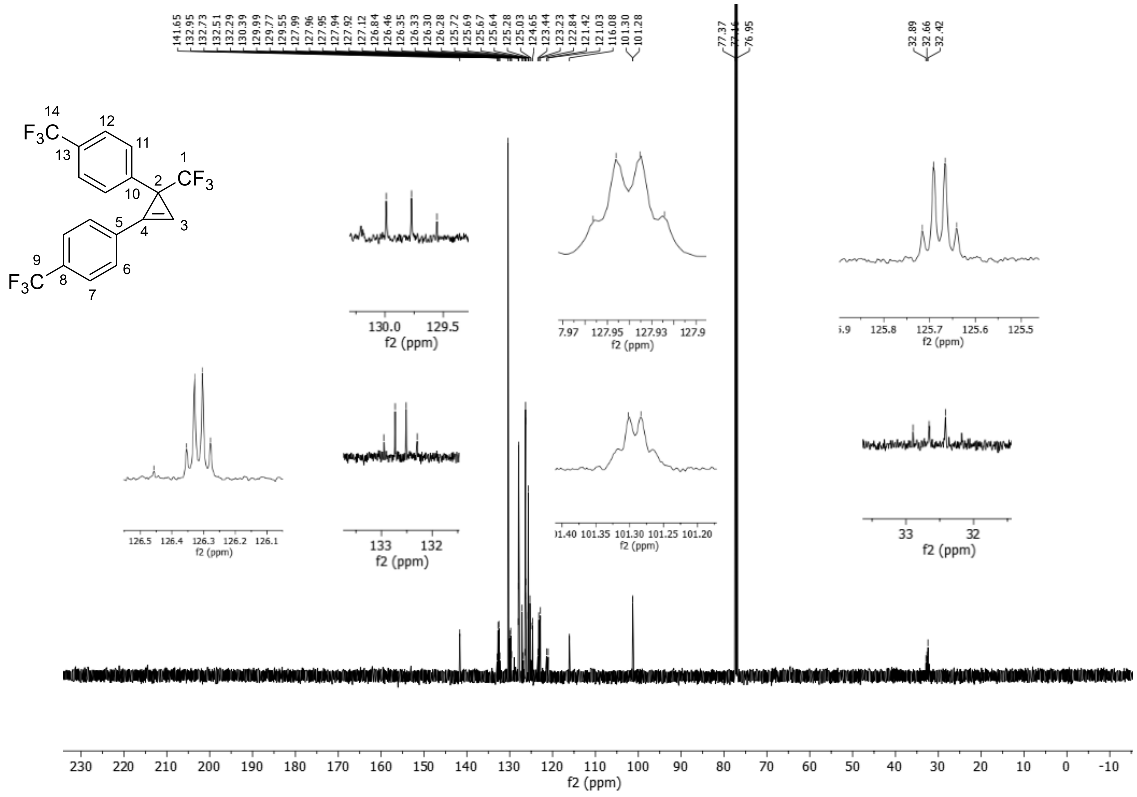

Figure S11. $^{13}C\{^{1}H\}$ NMR of **S4** (151 MHz, 299 K, $CDCl_3$).

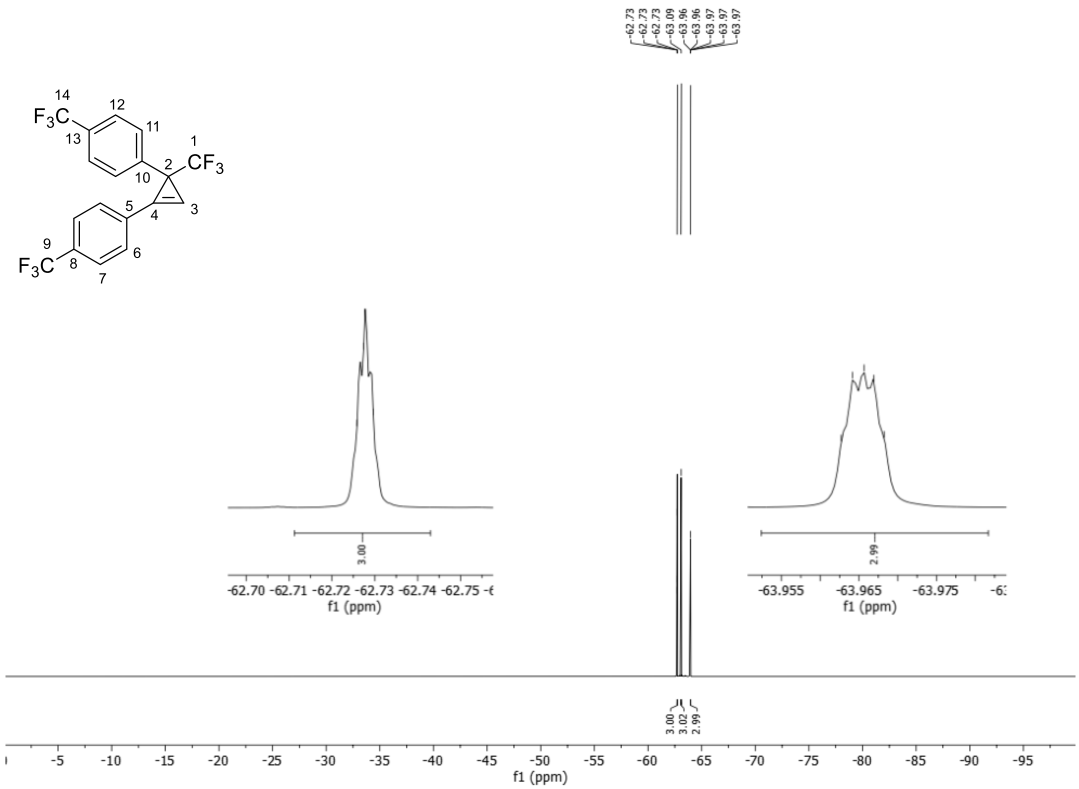

Figure S12. ^{19}F NMR of **S4** (564 MHz, 299 K, $CDCl_3$).

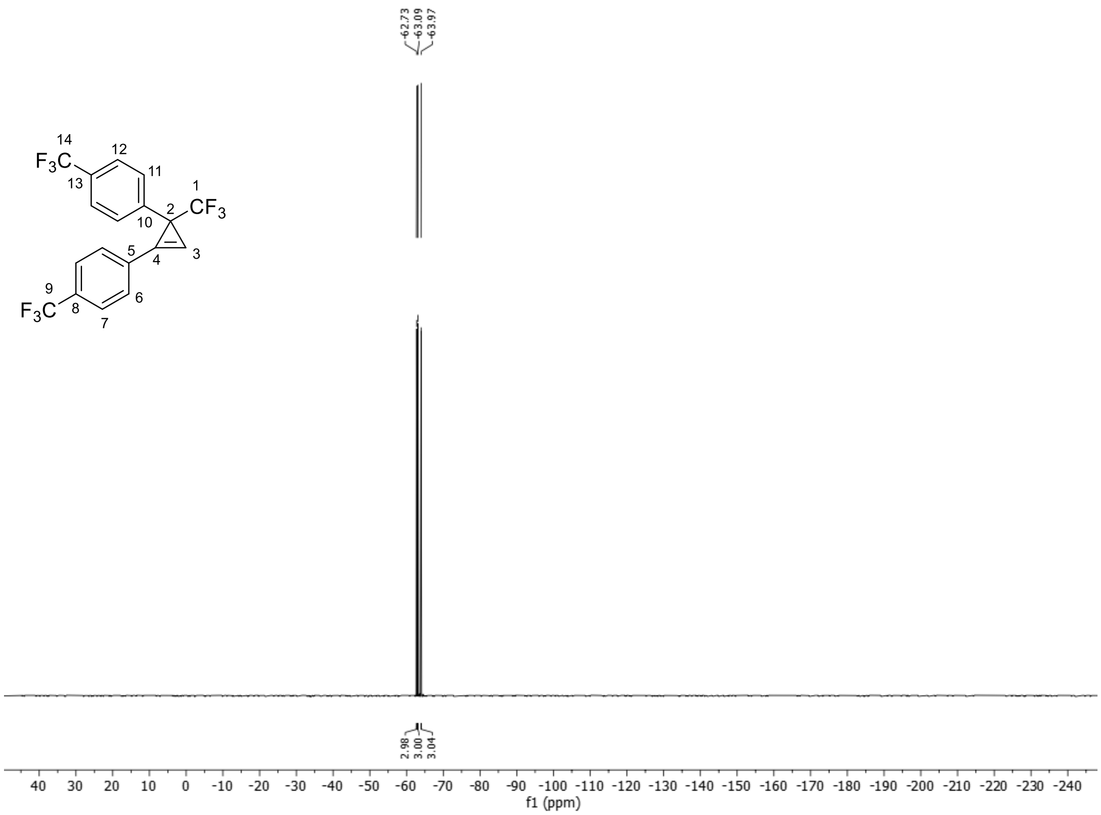

Figure S13. $^{19}F\{^1H\}$ NMR of **S4** (377 MHz, 299 K, $CDCl_3$).

1-Methyl-4-(1-(trifluoromethyl)-2-(4-(trifluoromethyl)phenyl)cycloprop-2-en-1-yl)benzene (S5)

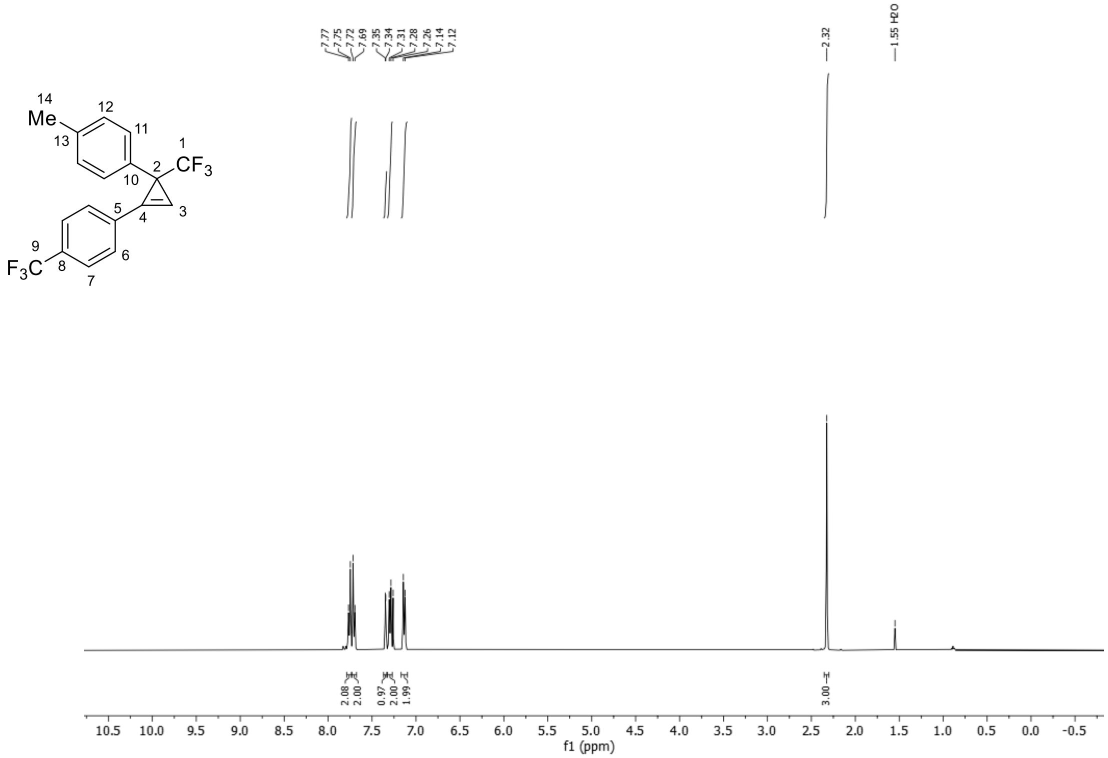

Figure S14. ^{1}H NMR of **S5** (400 MHz, 299 K, $CDCl_3$).

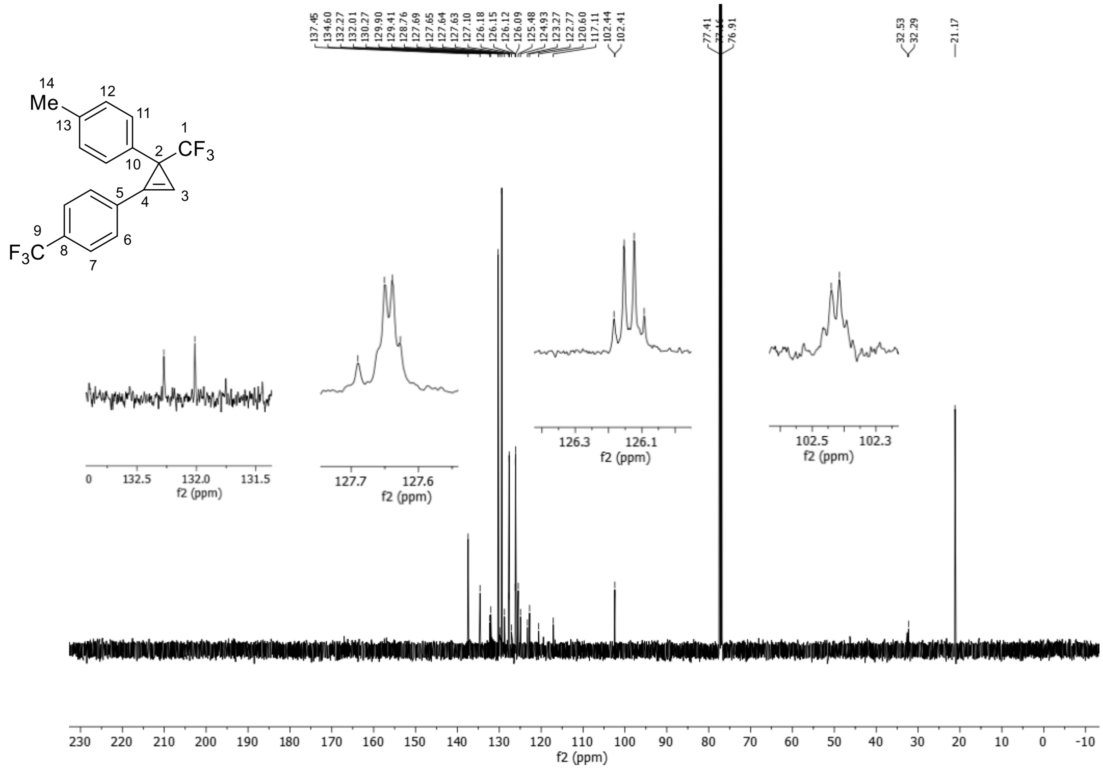

Figure S15. $^{13}C\{^{1}H\}$ NMR of **S5** (126 MHz, 299 K, $CDCl_3$).

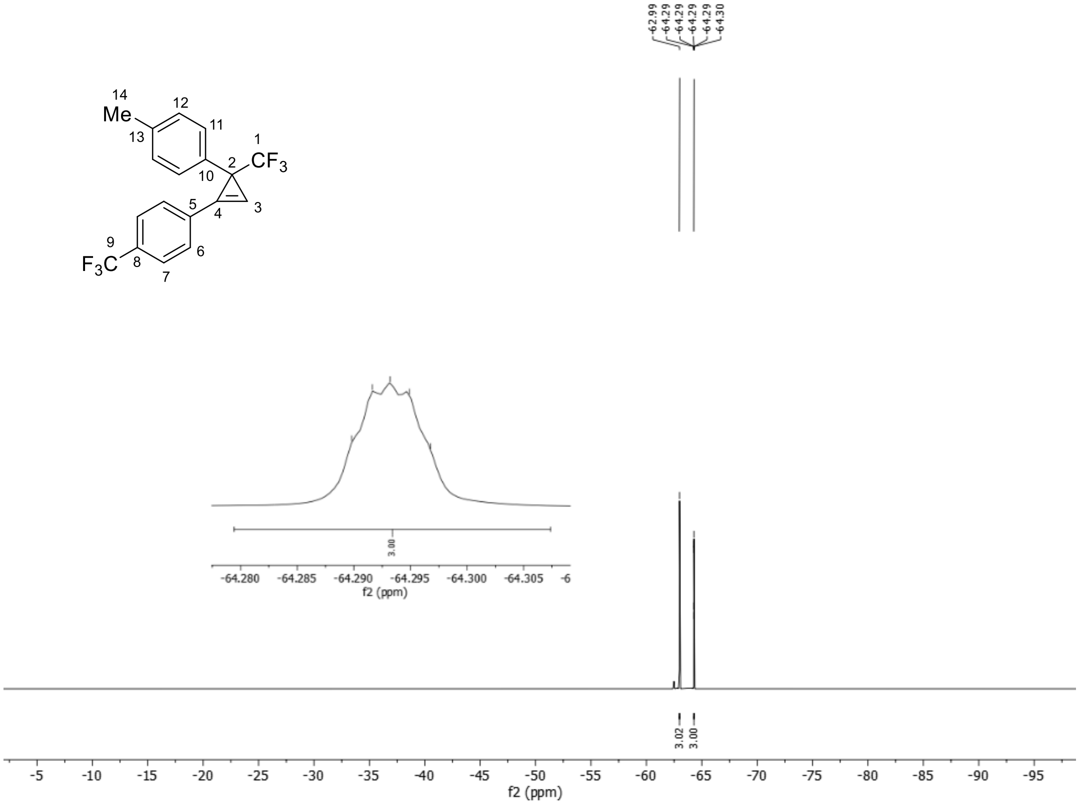

Figure S16. ^{19}F NMR of **S5** (470 MHz, 299 K, $CDCl_3$).

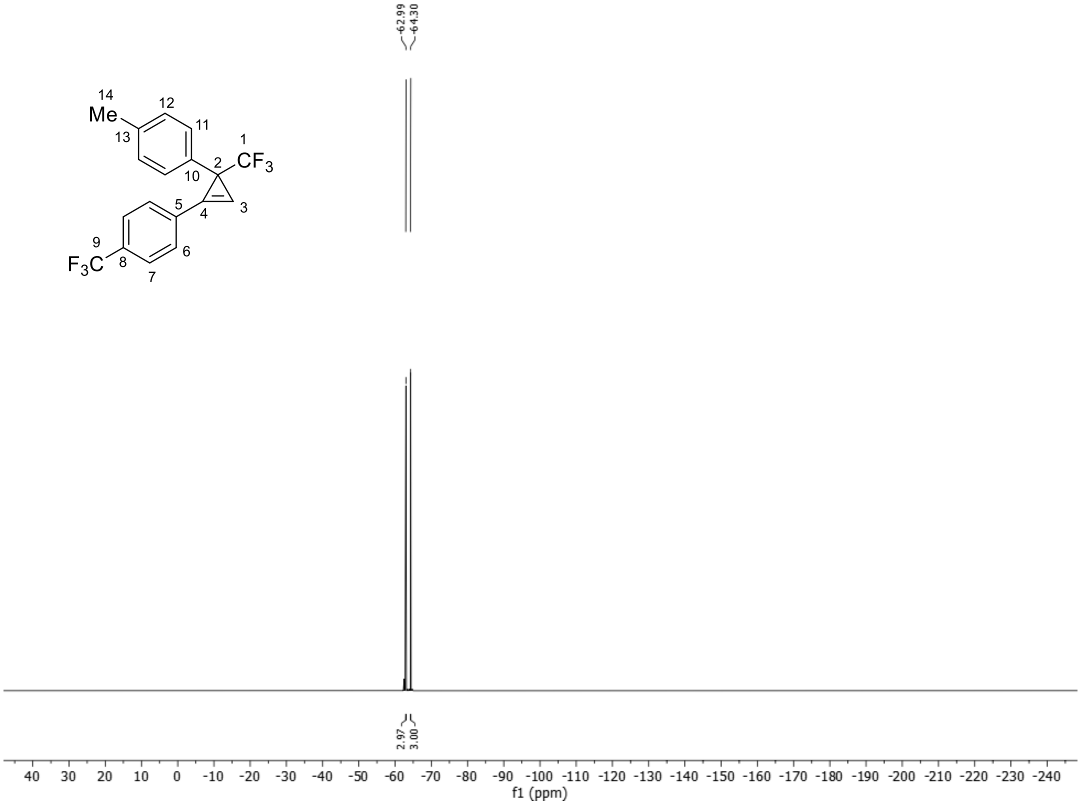

Figure S17. ^{19}F{^{1}H} NMR of **S5** (377 MHz, 299 K, $CDCl_3$).

1-(3-Benzyl-3-(trifluoromethyl)cycloprop-1-en-1-yl)-4-(trifluoromethyl)benzene (S6)

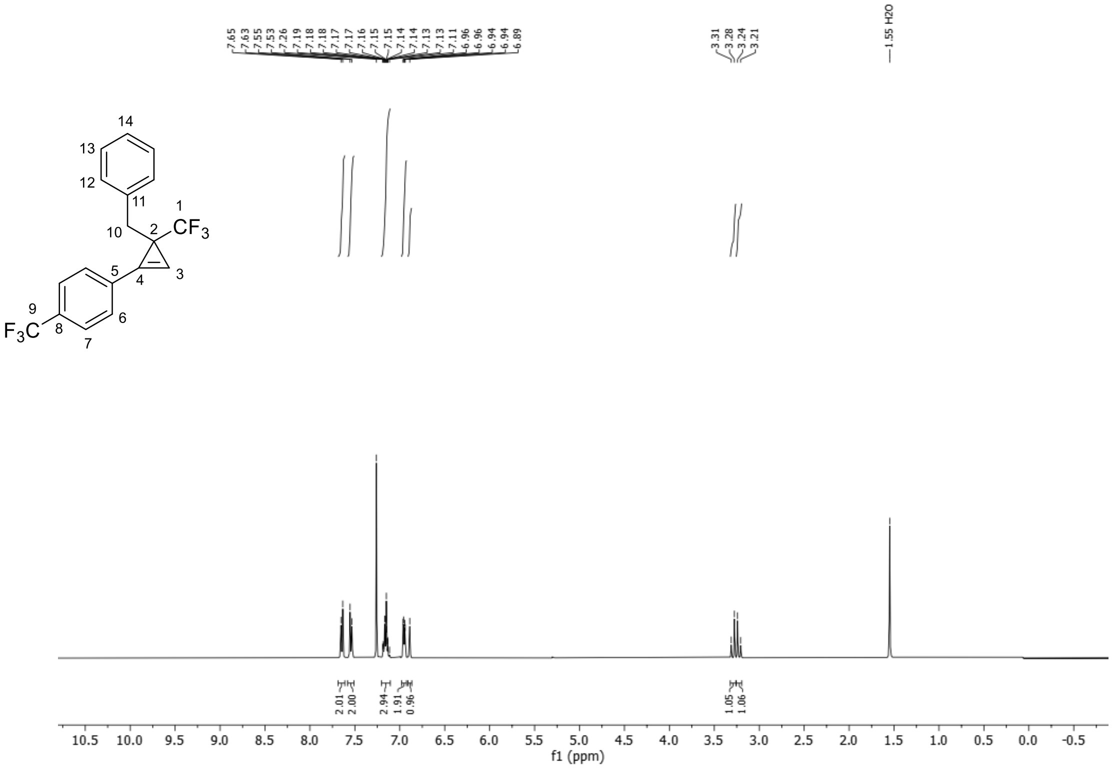

Figure S18. ^{1}H NMR of **S6** (400 MHz, 299 K, $CDCl_3$).

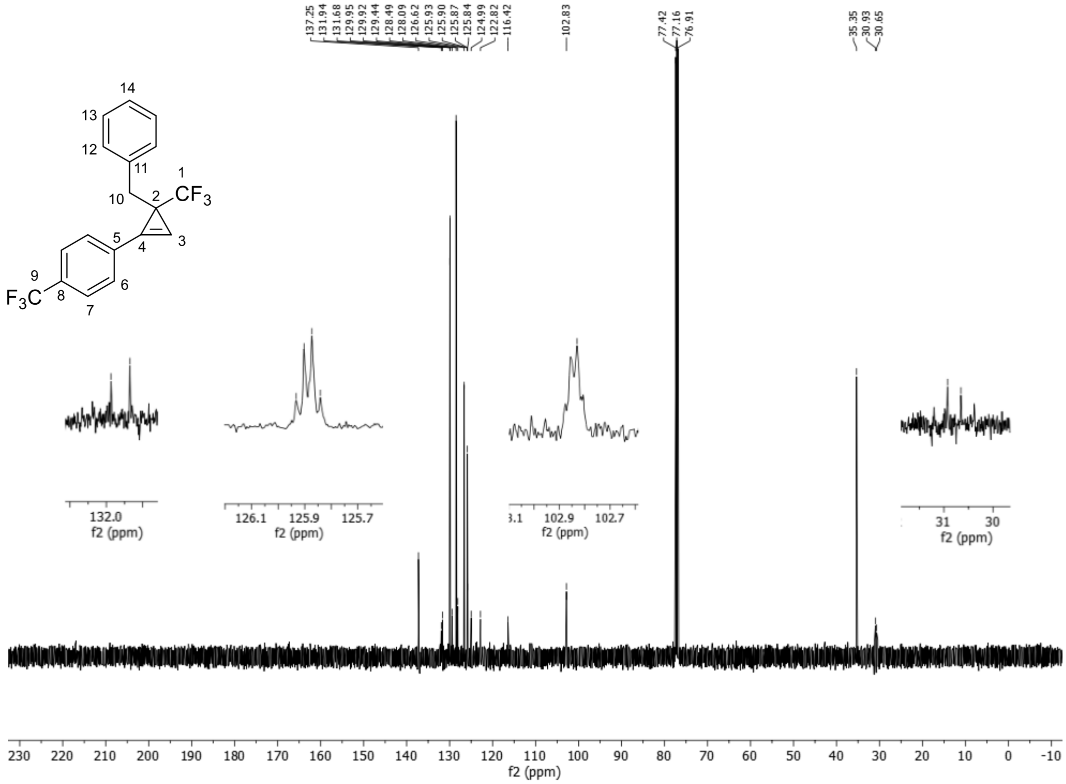

Figure S19. $^{13}C\{^{1}H\}$ NMR of **S6** (126 MHz, 299 K, $CDCl_3$).

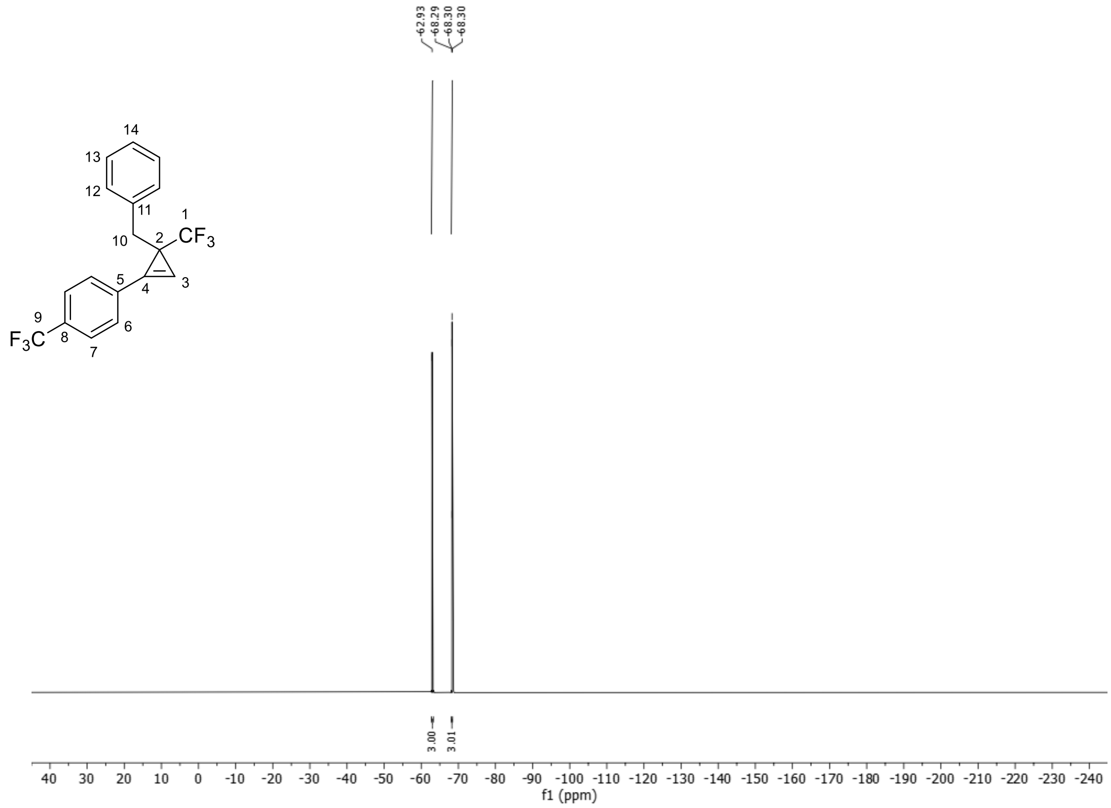

Figure S20. ^{19}F NMR of **S6** (470 MHz, 299 K, $CDCl_3$).

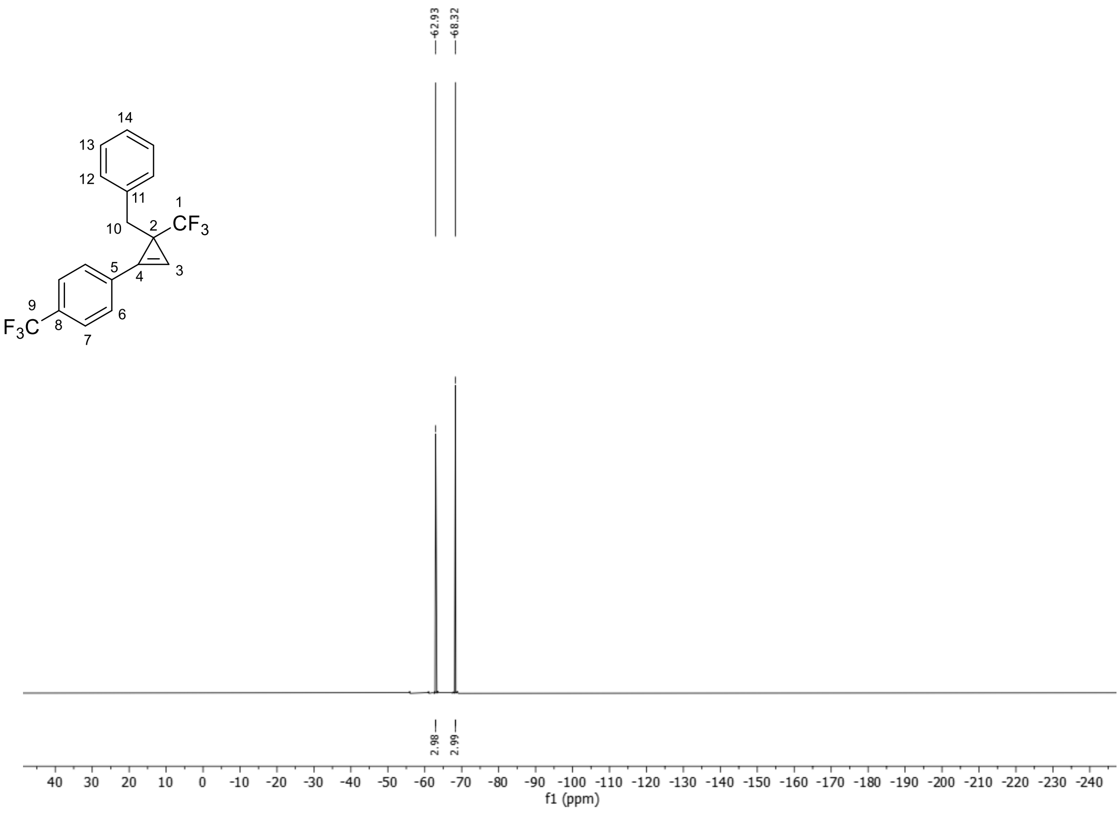

Figure S21. $^{19}F\{^1H\}$ NMR of **S6** (377 MHz, 299 K, $CDCl_3$).

1-(3-(4-Bromophenyl)-3-(trifluoromethyl)cycloprop-1-en-1-yl)-3,5-dichlorobenzene (S7)

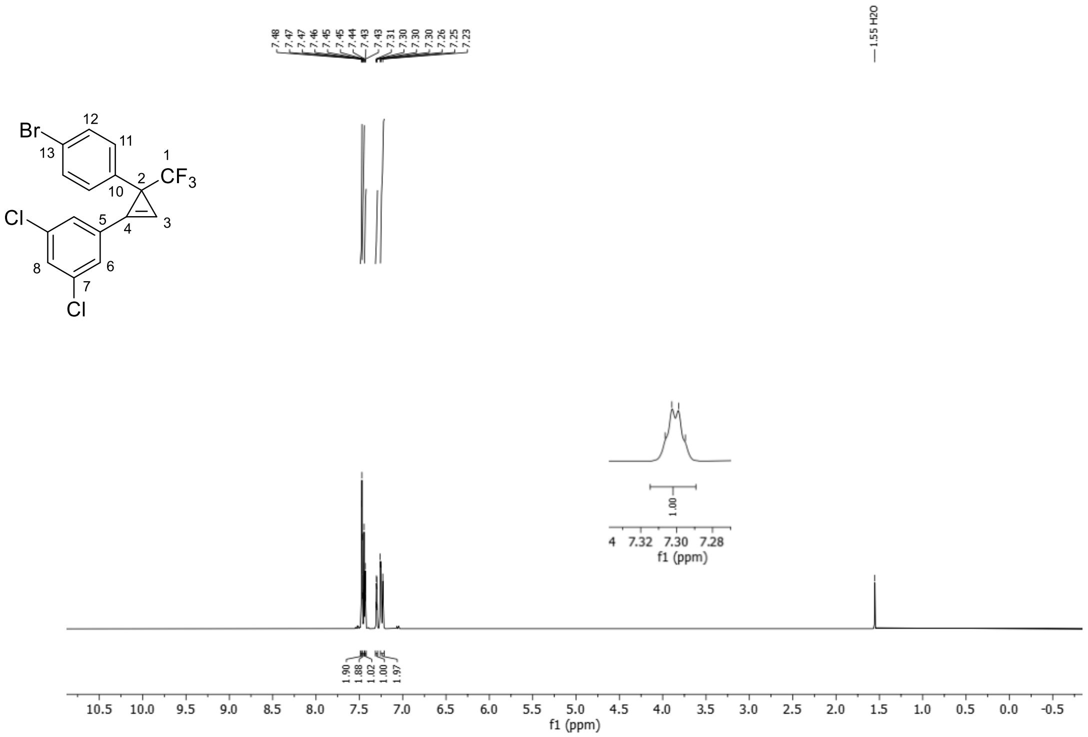

Figure S22. ^{1}H NMR of **S7** (400 MHz, 299 K, $CDCl_3$).

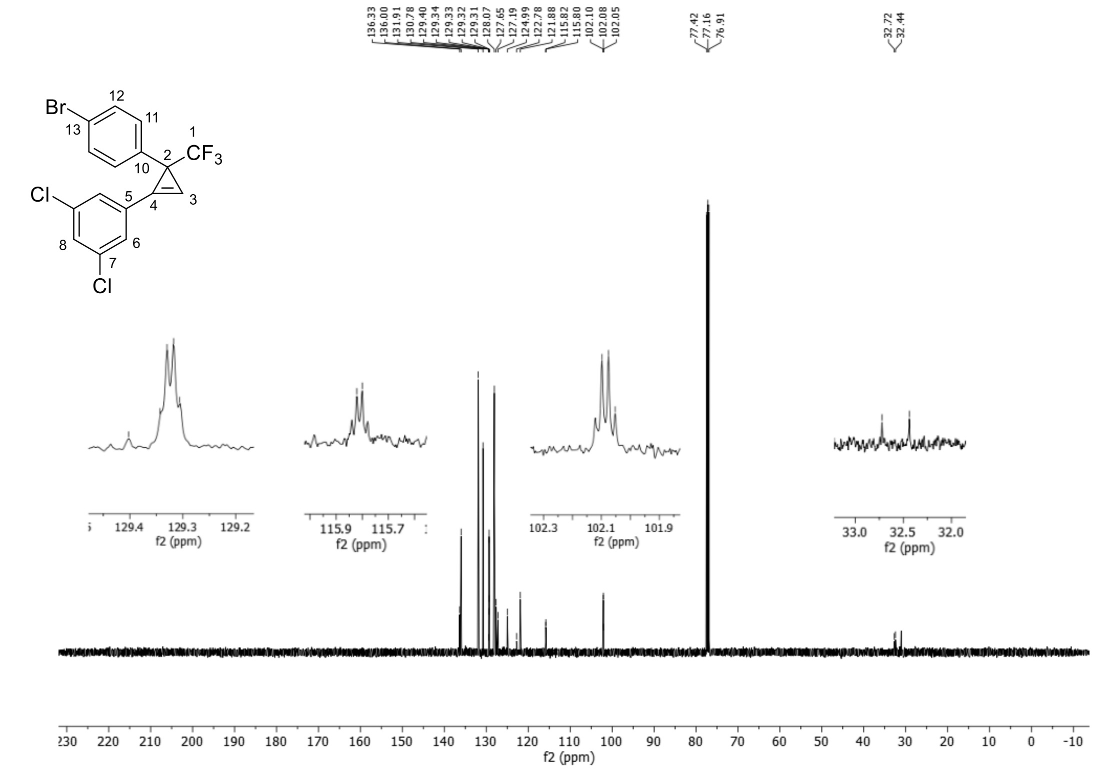

Figure S23. $^{13}C\{^{1}H\}$ NMR of **S7** (126 MHz, 299 K, $CDCl_3$).

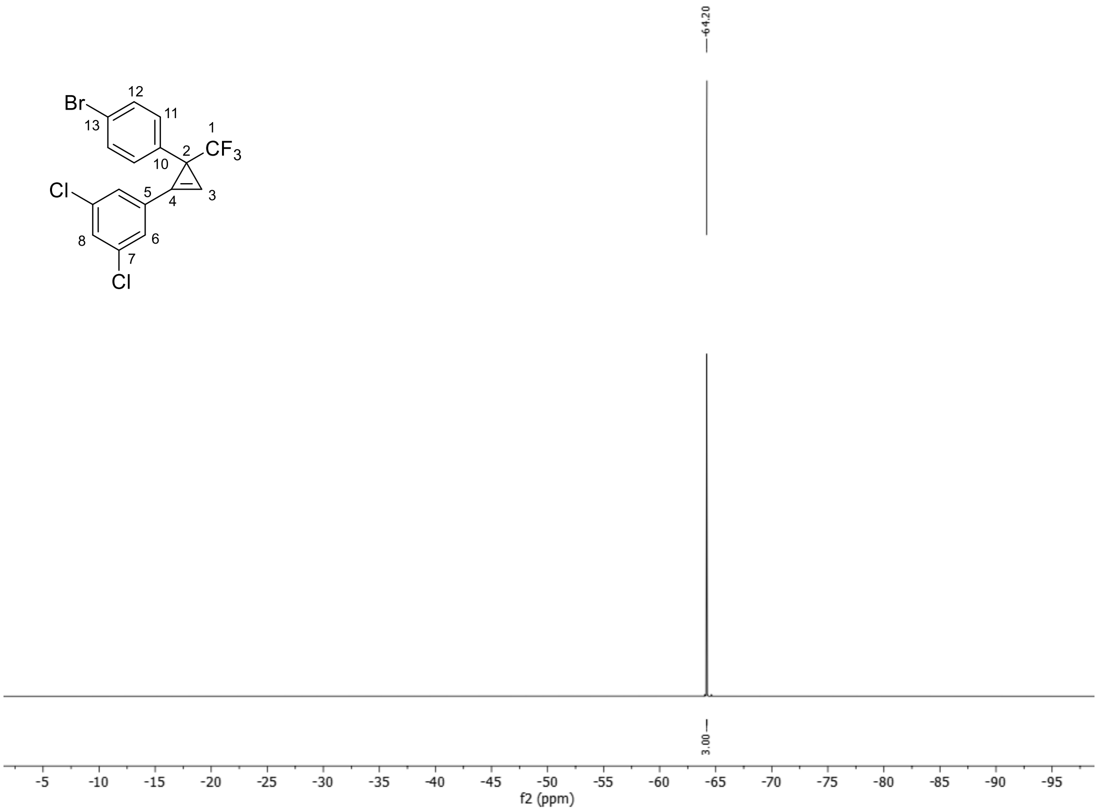

Figure S24. ^{19}F NMR of **S7** (470 MHz, 299 K, $CDCl_3$).

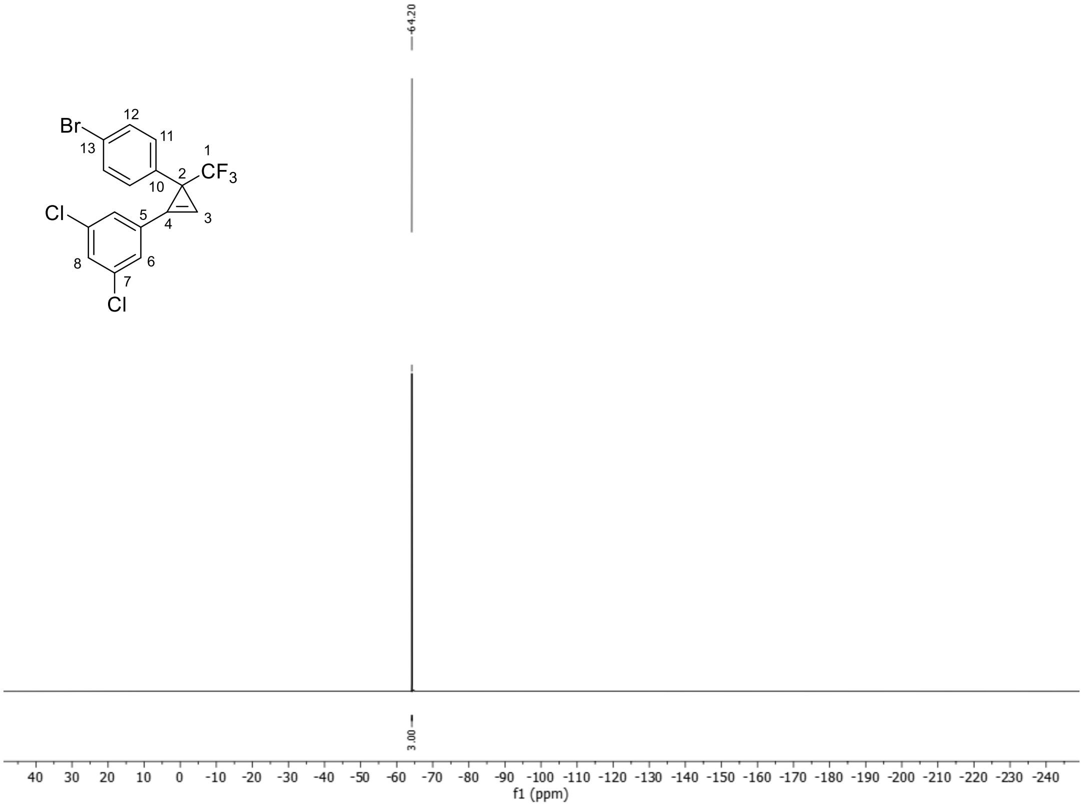

Figure S25. ^{19}F{^{1}H} NMR of **S7** (377 MHz, 299 K, $CDCl_3$).

Methyl 4-(3-(4-bromophenyl)-3-(trifluoromethyl)cycloprop-1-en-1-yl)benzoate (S8)

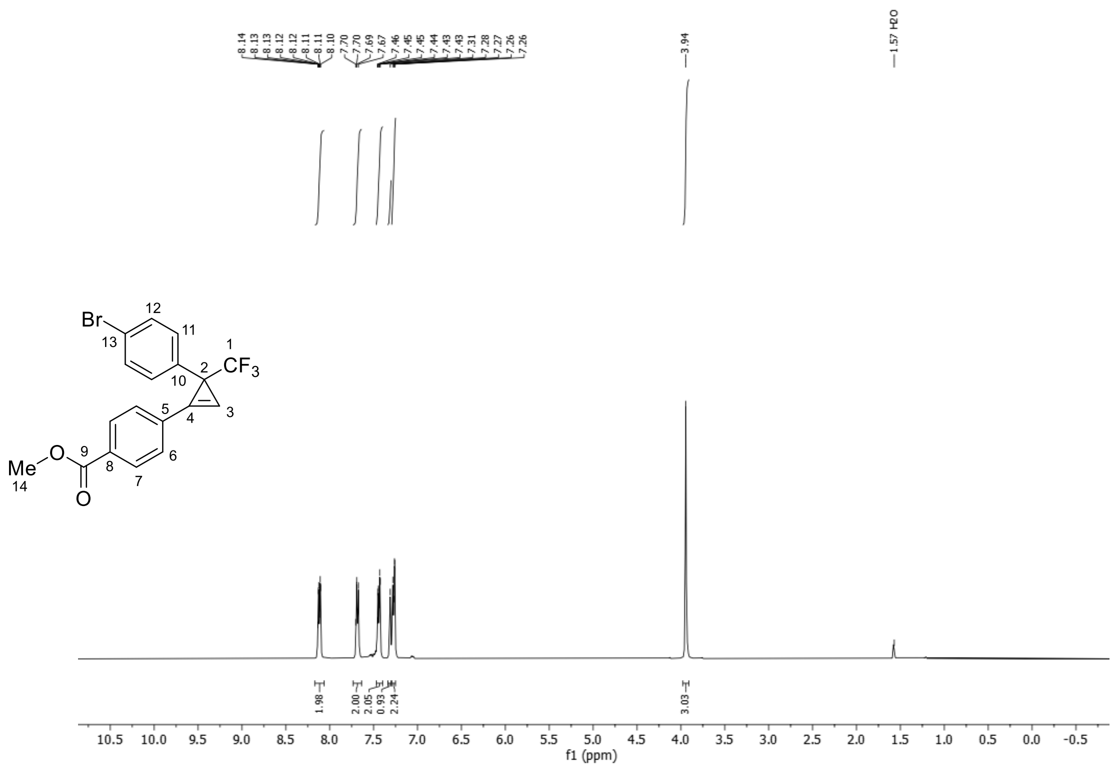

Figure S26. ^{1}H NMR of **S8** (400 MHz, 299 K, $CDCl_3$).

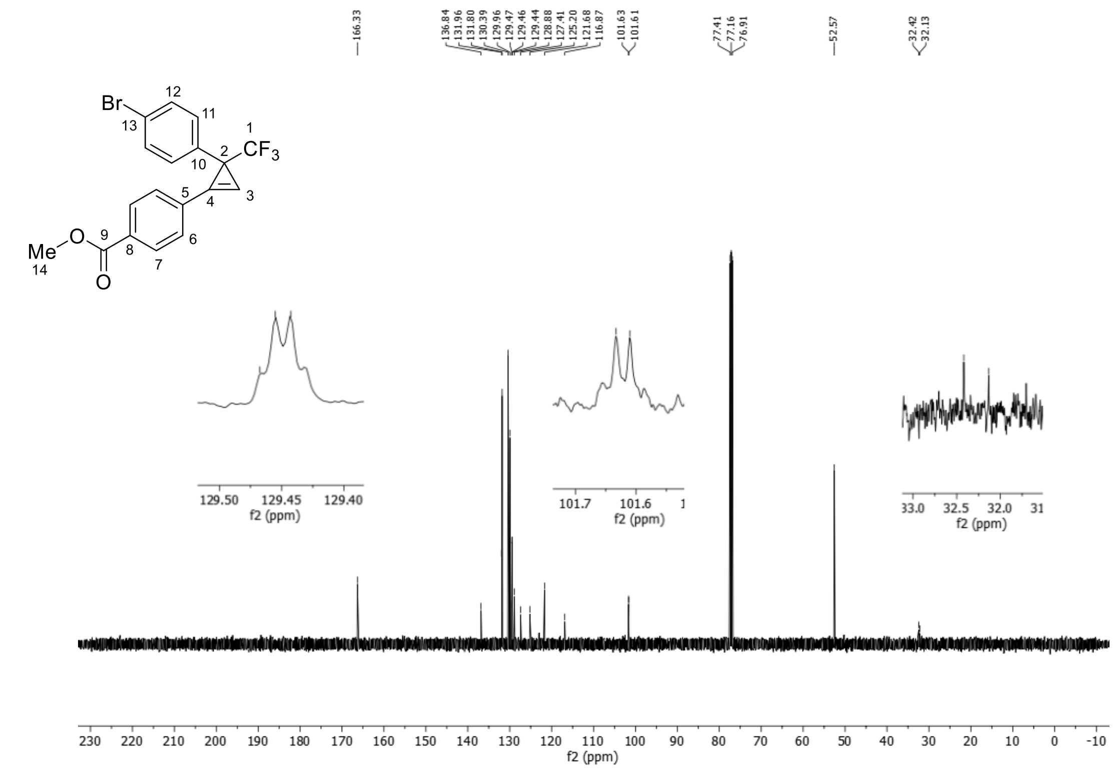

Figure S27. ^{13}C{^{1}H} NMR of **S8** (126 MHz, 299 K, $CDCl_3$).

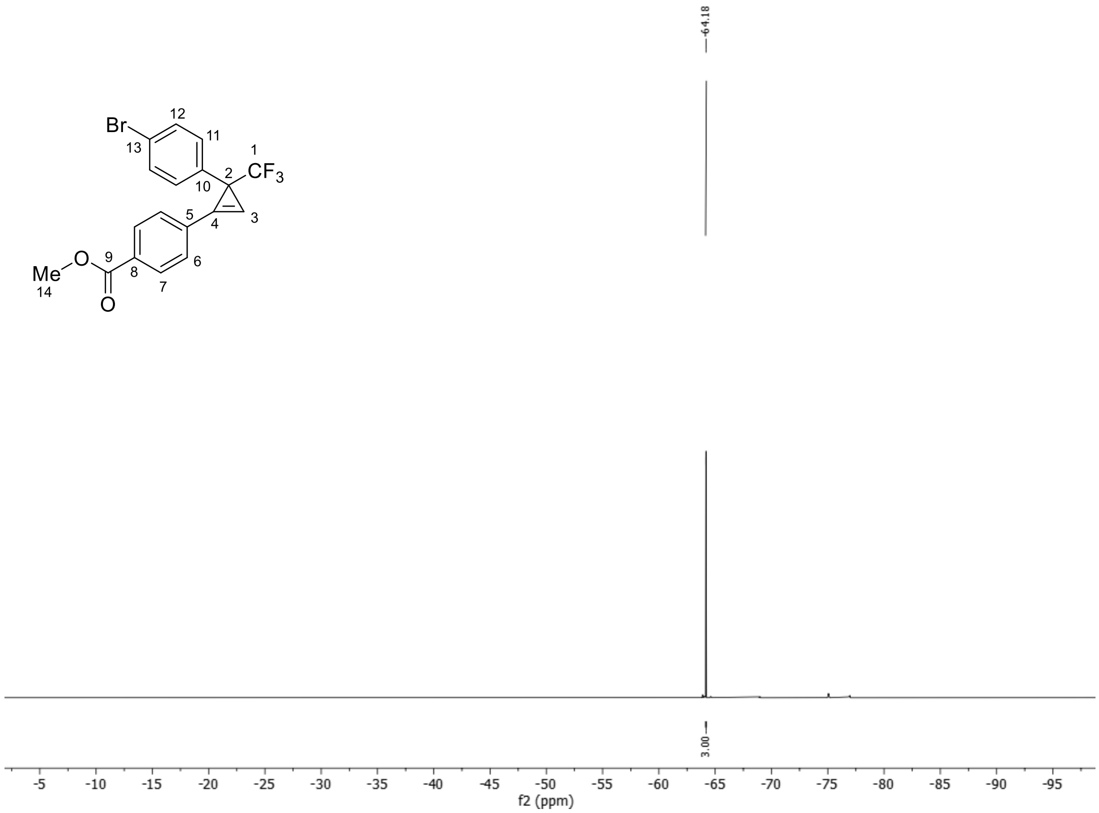

Figure S28. ^{19}F NMR of **S8** (470 MHz, 299 K, $CDCl_3$).

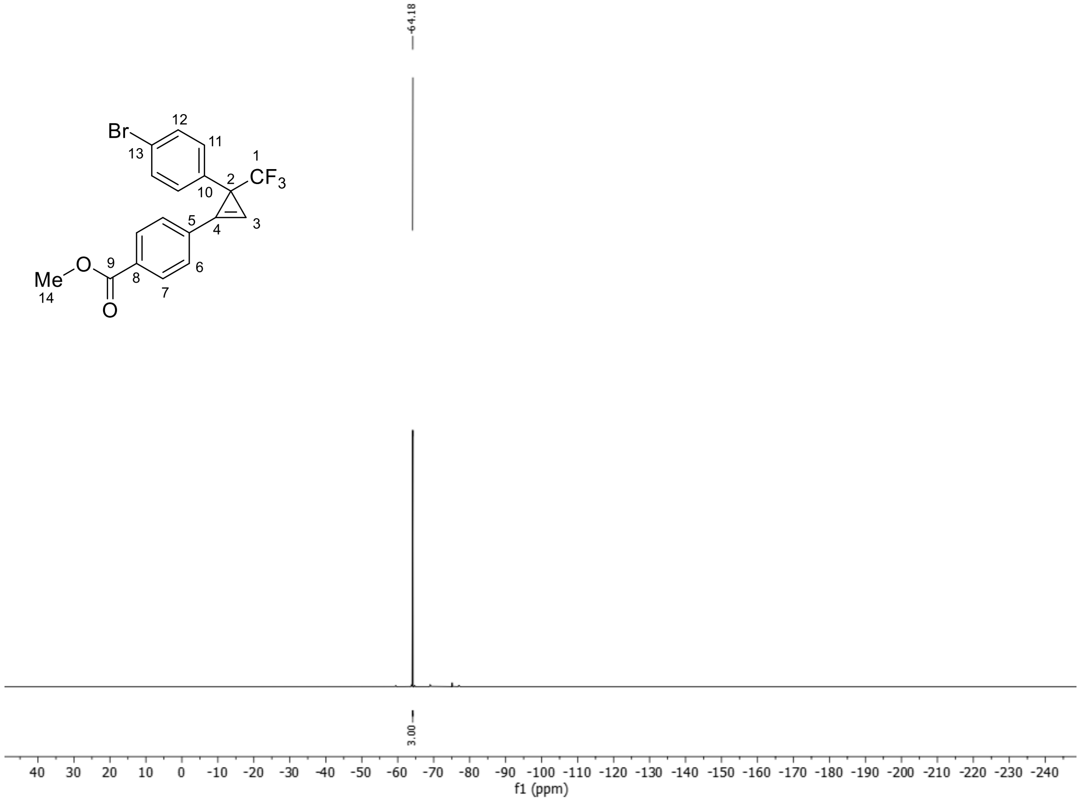

Figure S29. ^{19}F{^{1}H} NMR of **S8** (377 MHz, 299 K, $CDCl_3$).

4-(3-(4-Bromophenyl)-3-(trifluoromethyl)cycloprop-1-en-1-yl)benzonitrile (S9)

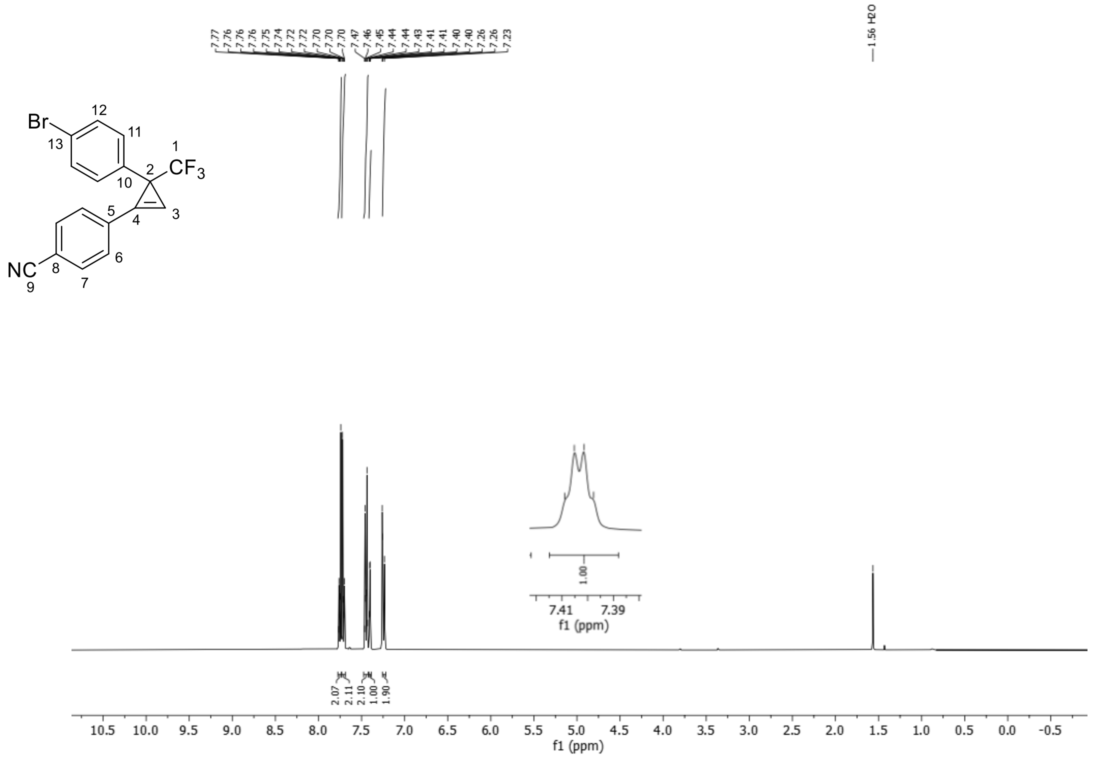

Figure S30. ^{1}H NMR of **S9** (400 MHz, 299 K, $CDCl_3$).

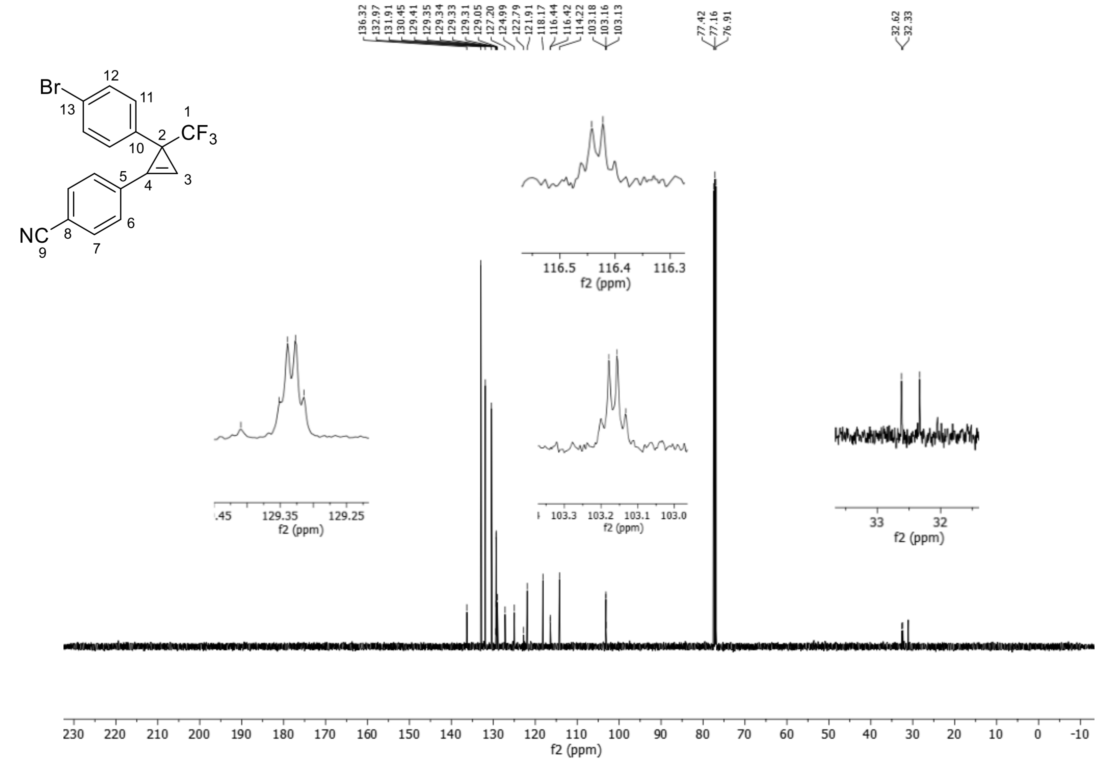

Figure S31. $^{13}C\{^1H\}$ NMR of **S9** (126 MHz, 299 K, $CDCl_3$).

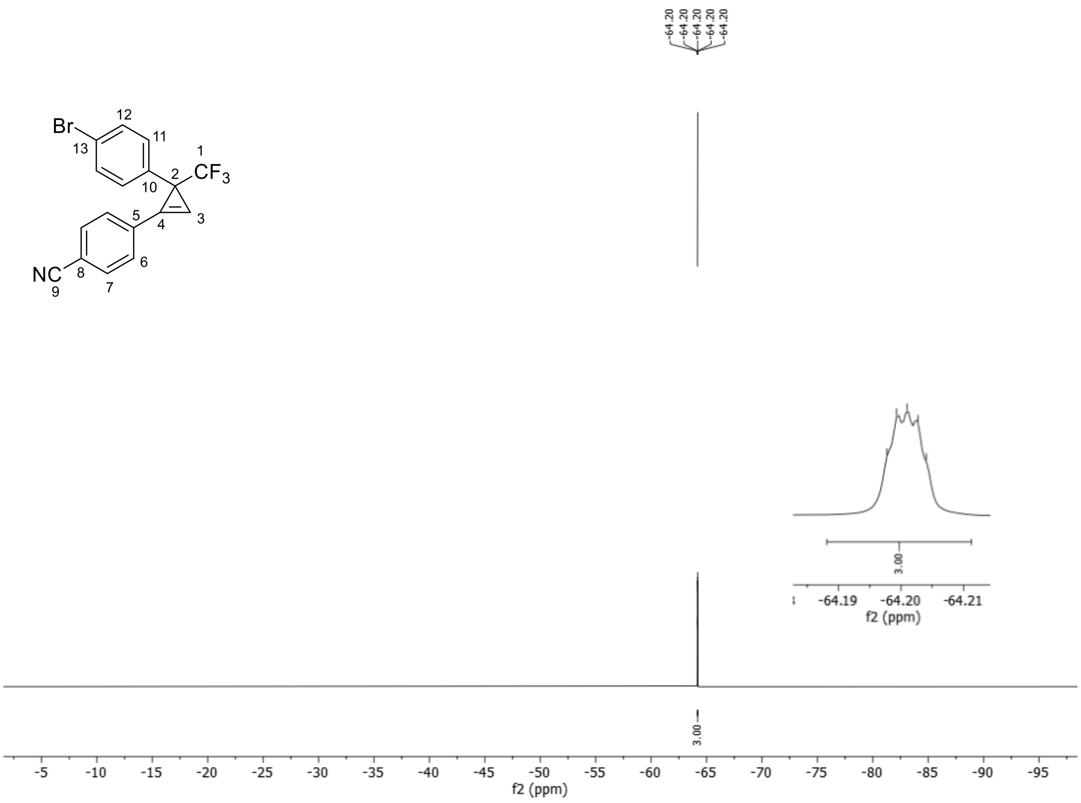

Figure S32. ^{19}F NMR of **S9** (470 MHz, 299 K, $CDCl_3$).

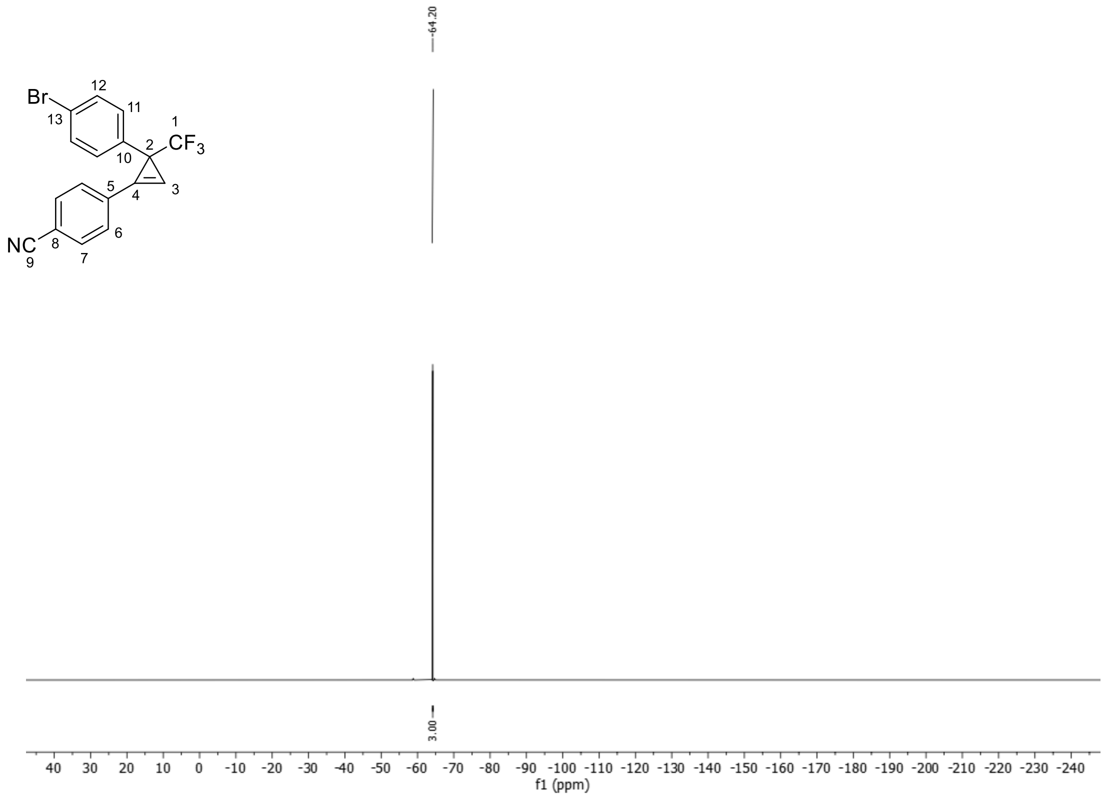

Figure S33. $^{19}F\{^1H\}$ NMR of **S9** (377 MHz, 299 K, $CDCl_3$).

(2-(Cyclohexylmethyl)-1-(trifluoromethyl)cycloprop-2-en-1-yl)benzene (S11)

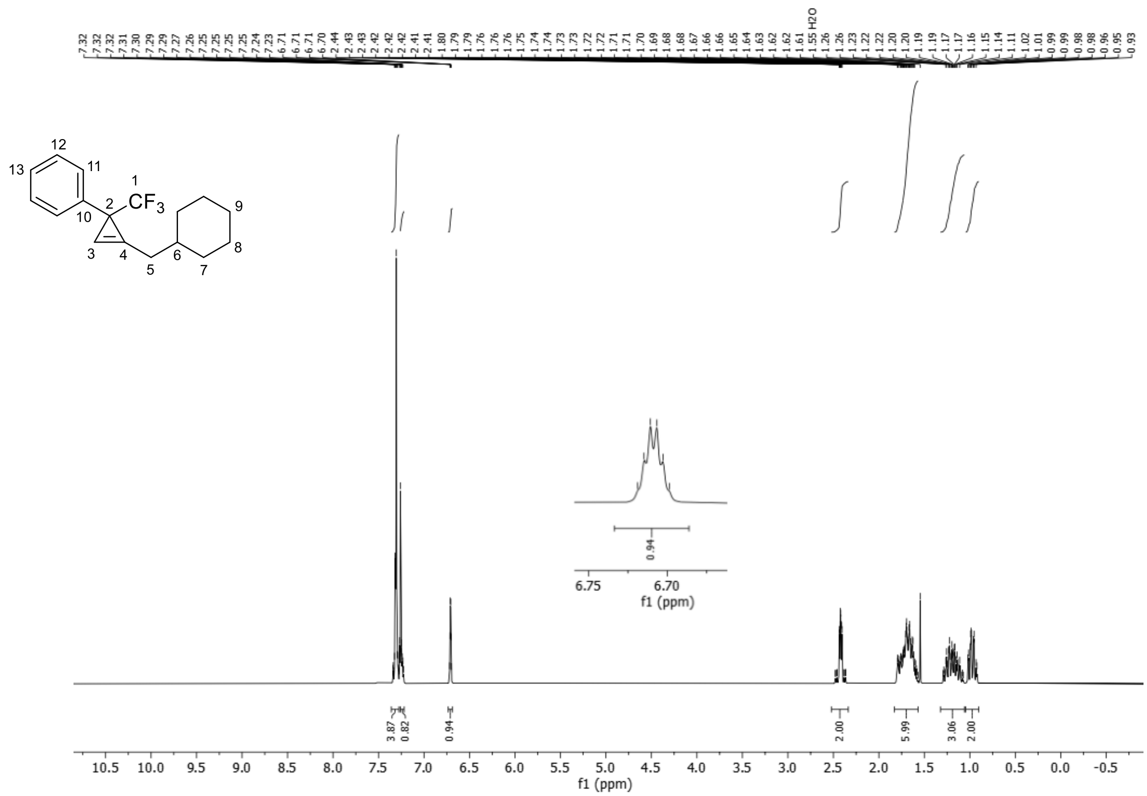

Figure S34. ^{1}H NMR of **S11** (400 MHz, 299 K, $CDCl_3$).

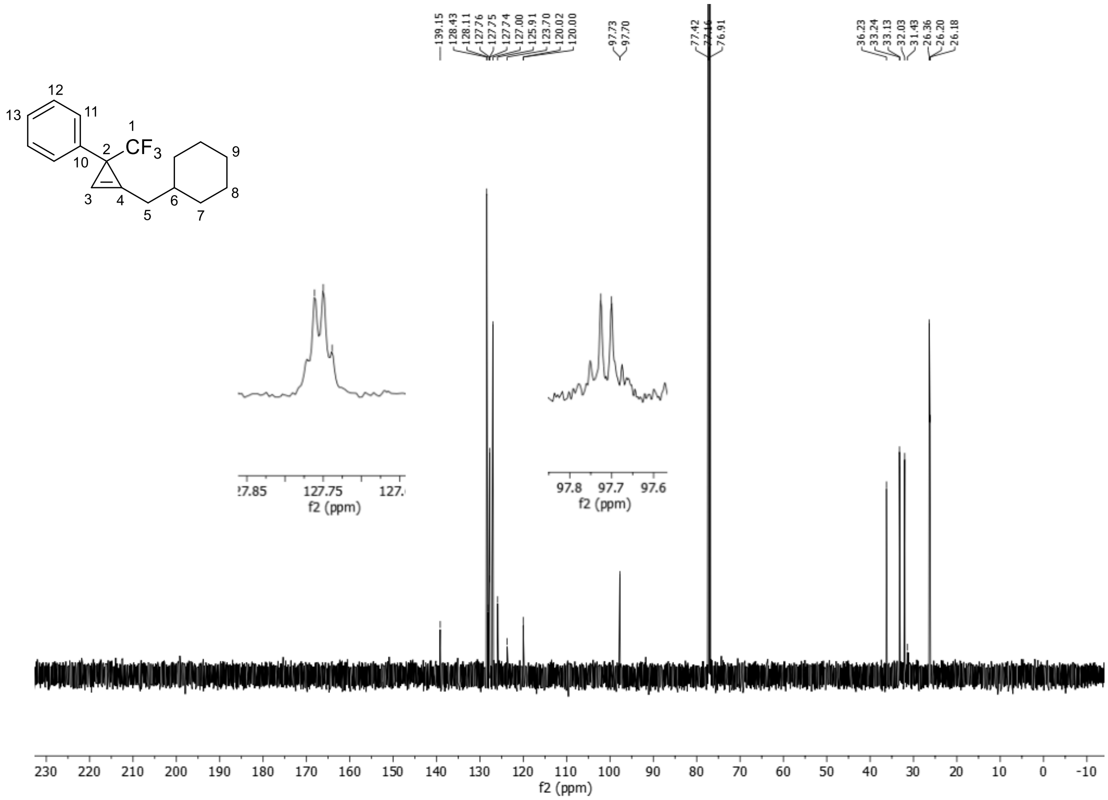

Figure S35. $^{13}C\{^{1}H\}$ NMR of **S11** (126 MHz, 299 K, $CDCl_3$).

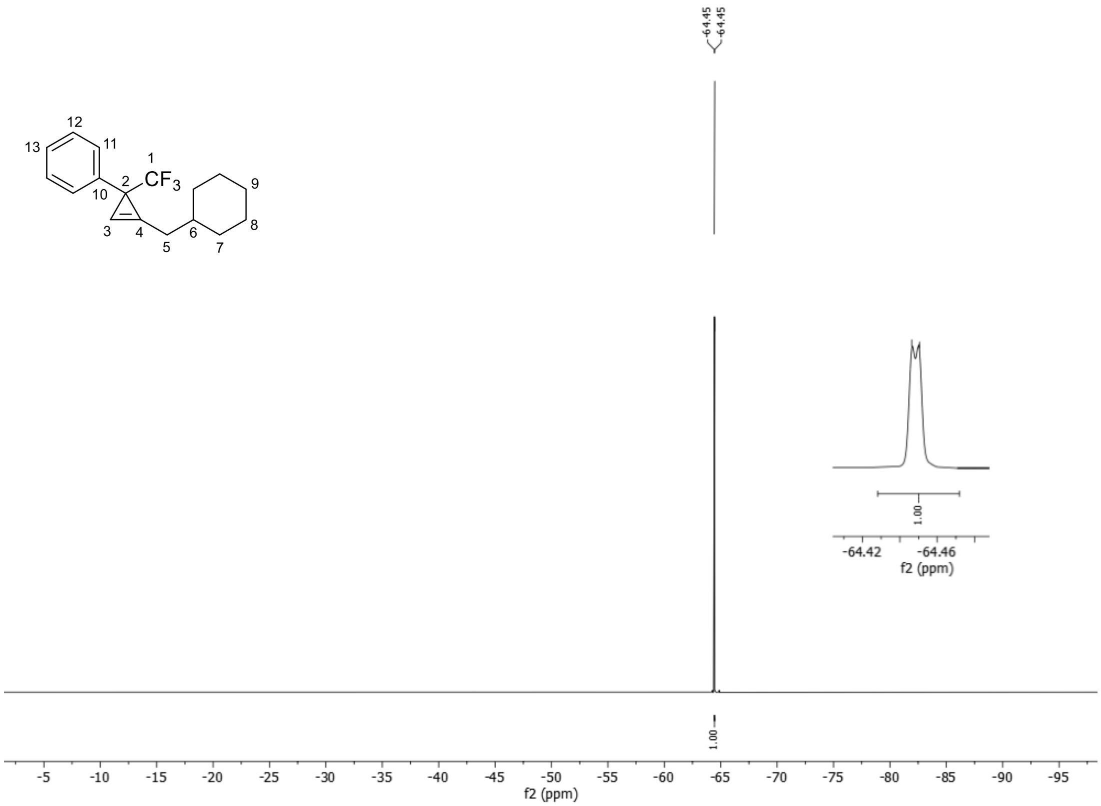

Figure S36. ^{19}F NMR of **S11** (470 MHz, 299 K, $CDCl_3$).

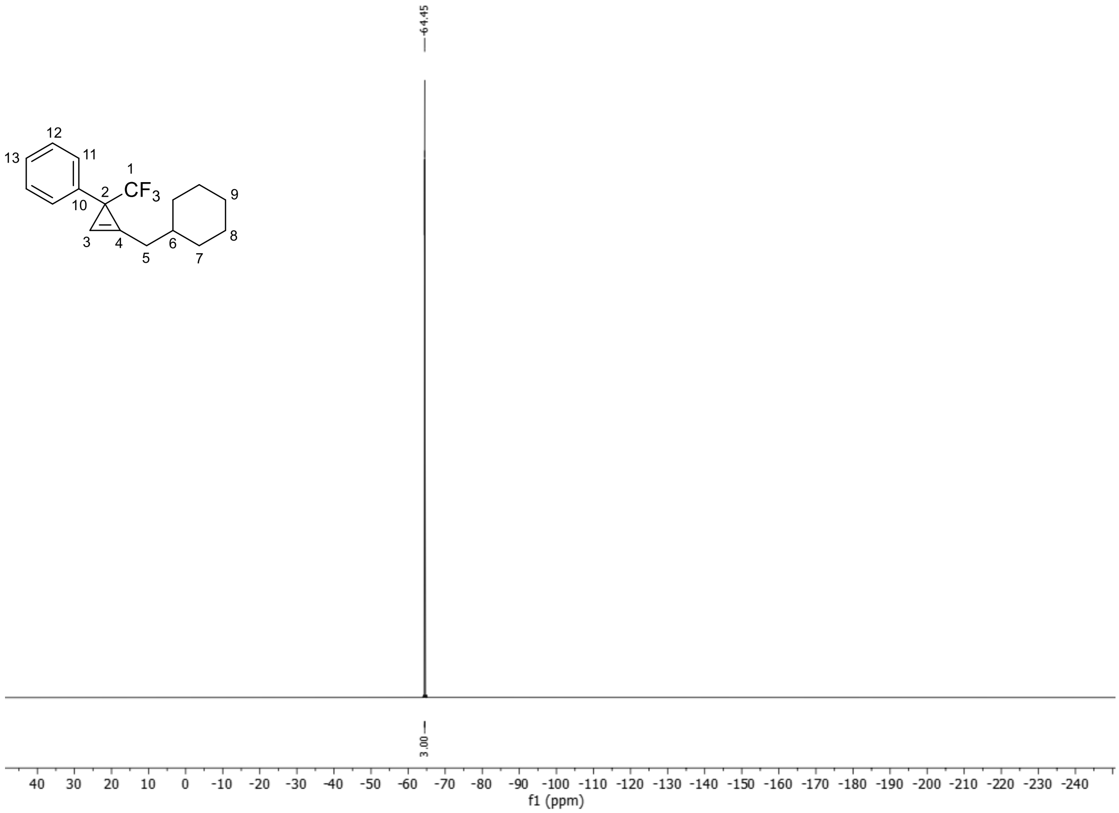

Figure S37. $^{19}F\{^1H\}$ NMR of **S11** (377 MHz, 299 K, $CDCl_3$).

Undec-10-yn-1-yl cyclopropanecarboxylate (Int 6)

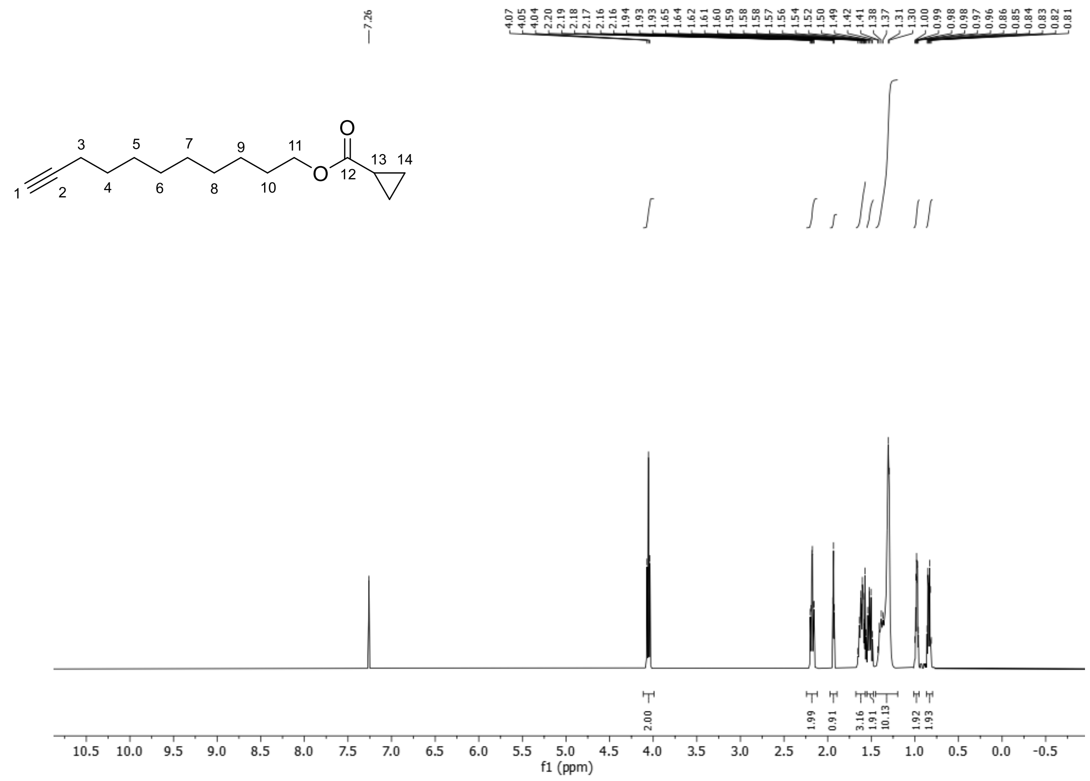

Figure S38. ^{1}H NMR of **Int 6** (400 MHz, 299 K, $CDCl_3$).

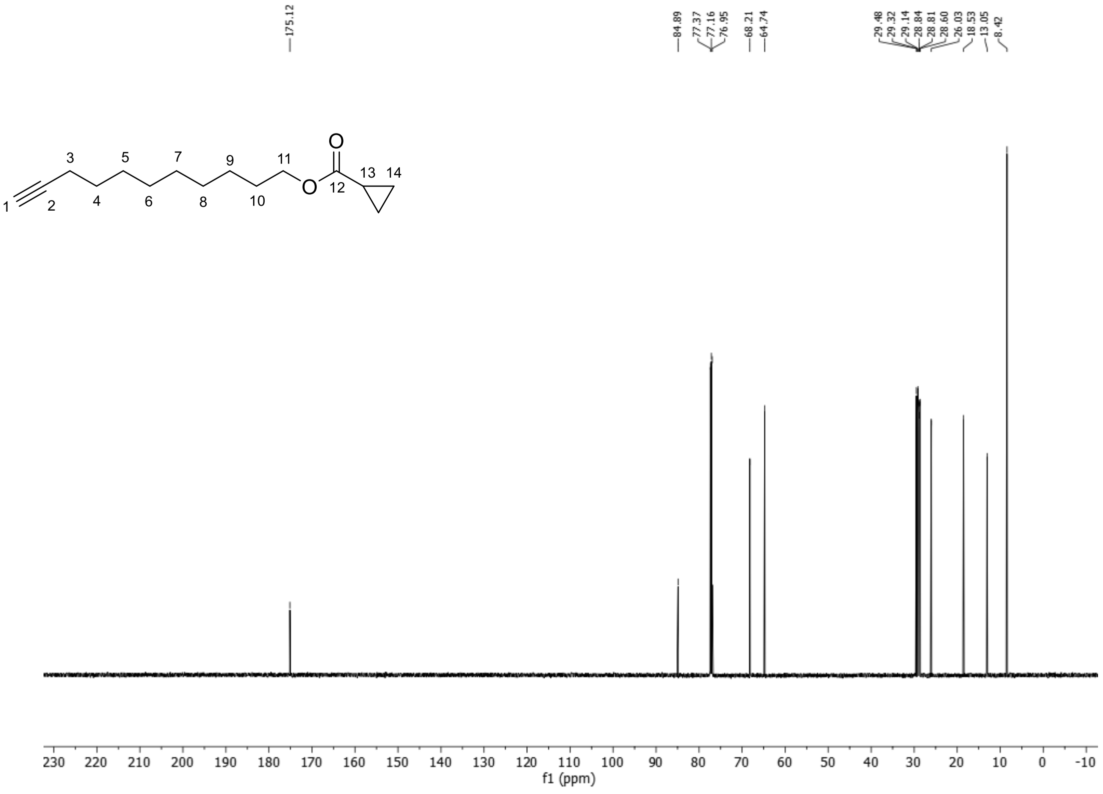

Figure S39. $^{13}C\{^{1}H\}$ NMR of **Int 6** (151 MHz, 299 K, $CDCl_3$).

9-(3-Phenyl-3-(trifluoromethyl)cycloprop-1-en-1-yl)nonyl cyclopropanecarboxylate (S12)

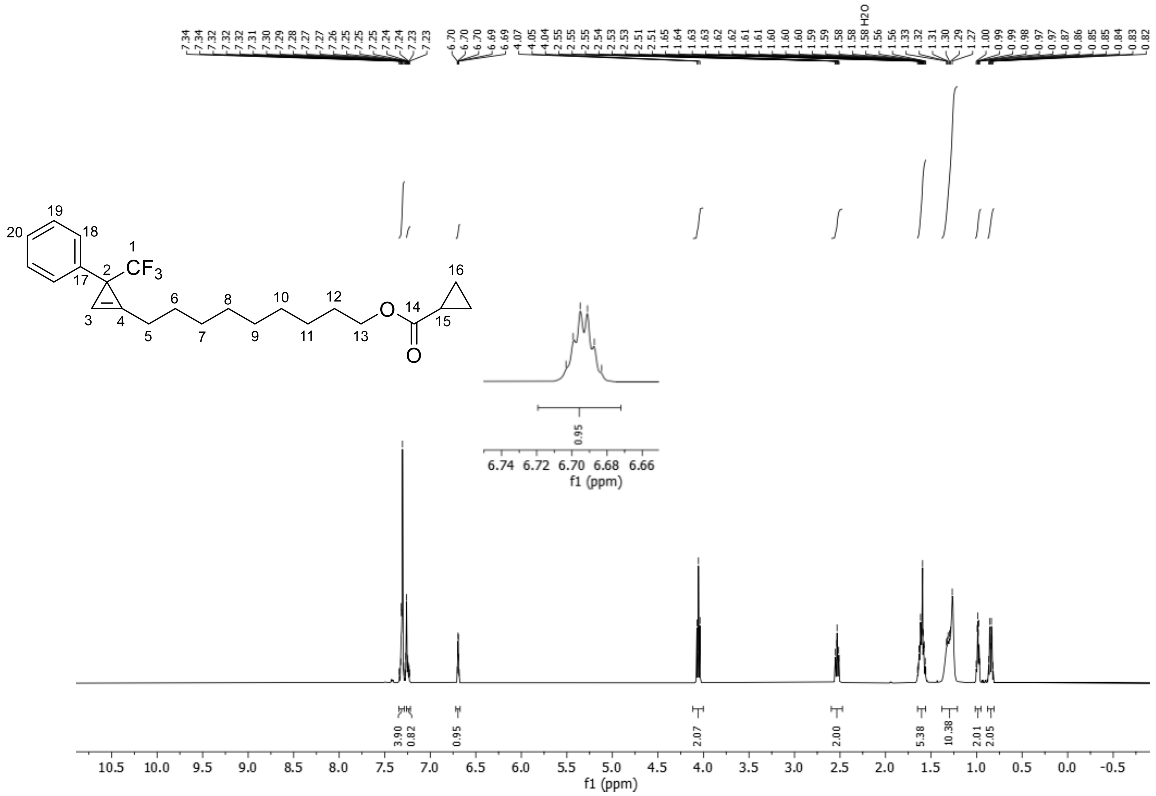

Figure S40. ^{1}H NMR of **S12** (400 MHz, 299 K, $CDCl_3$).

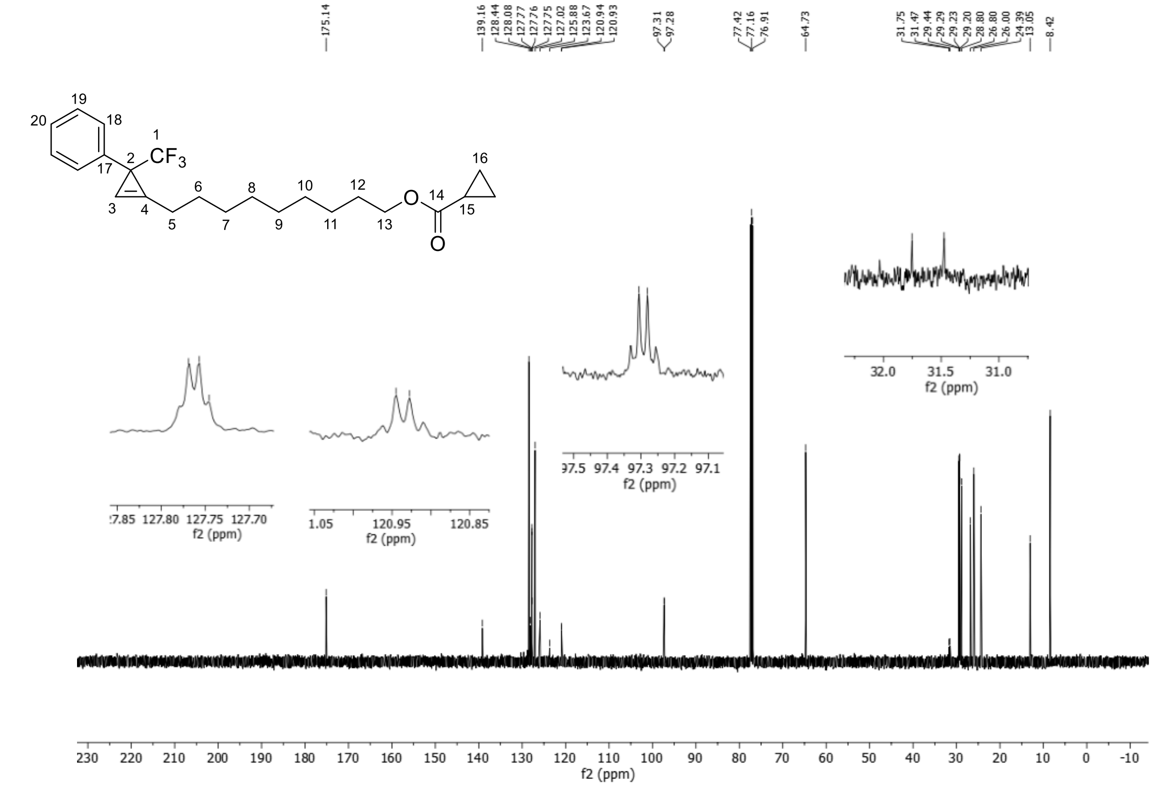

Figure S41. $^{13}C\{^{1}H\}$ NMR of **S12** (126 MHz, 299 K, $CDCl_3$).

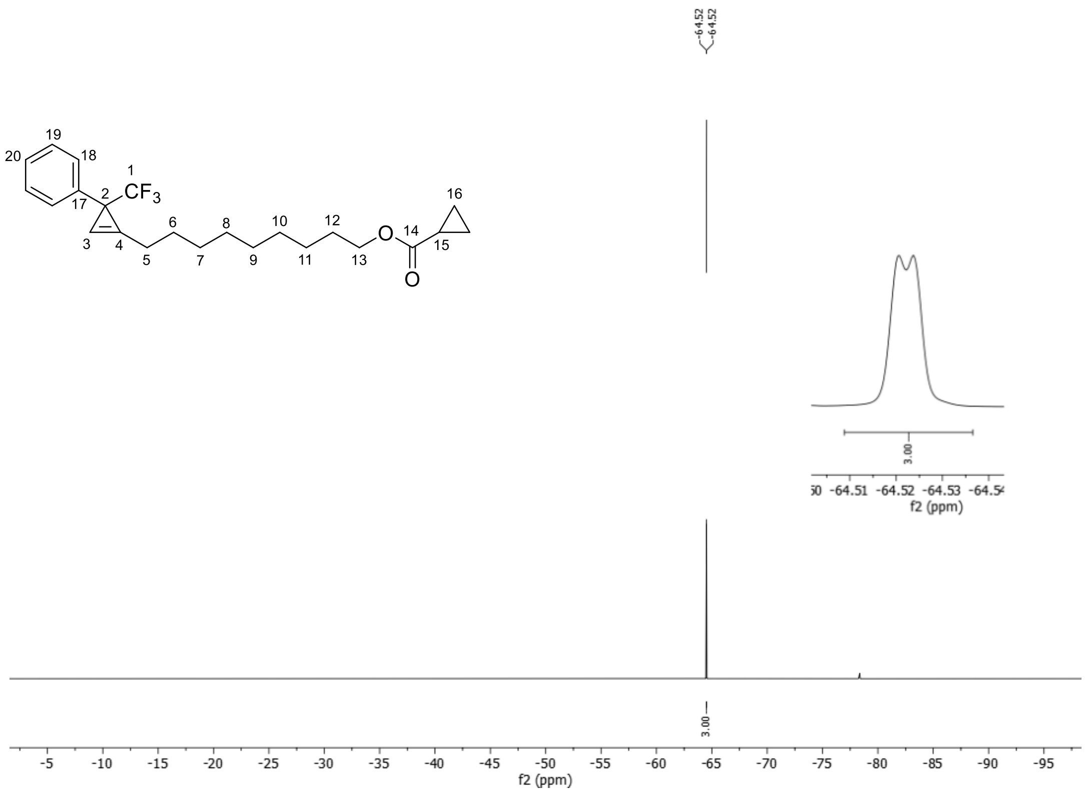

Figure S42. ^{19}F NMR of **S12** (470 MHz, 299 K, $CDCl_3$).

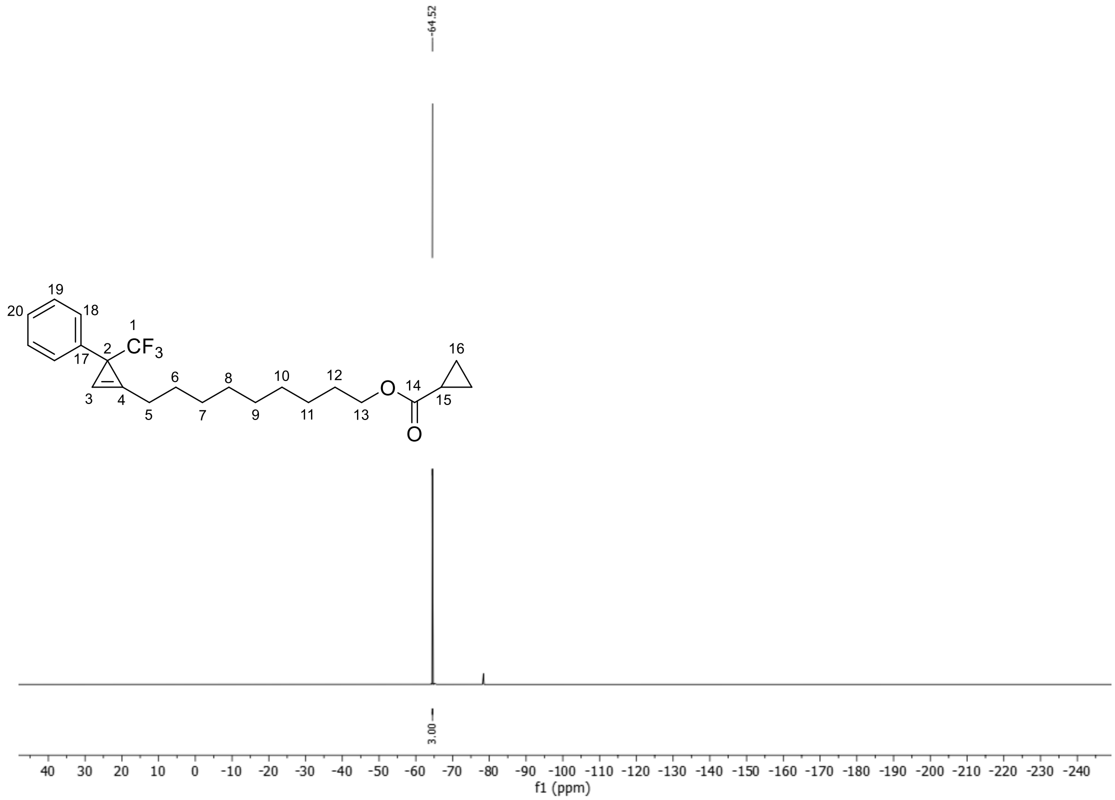

Figure S43. $^{19}F\{^1H\}$ NMR of **S12** (377 MHz, 299 K, $CDCl_3$).

Diethyl 2-(4-(trifluoromethyl)phenyl)cycloprop-2-ene-1,1-dicarboxylate (S13)

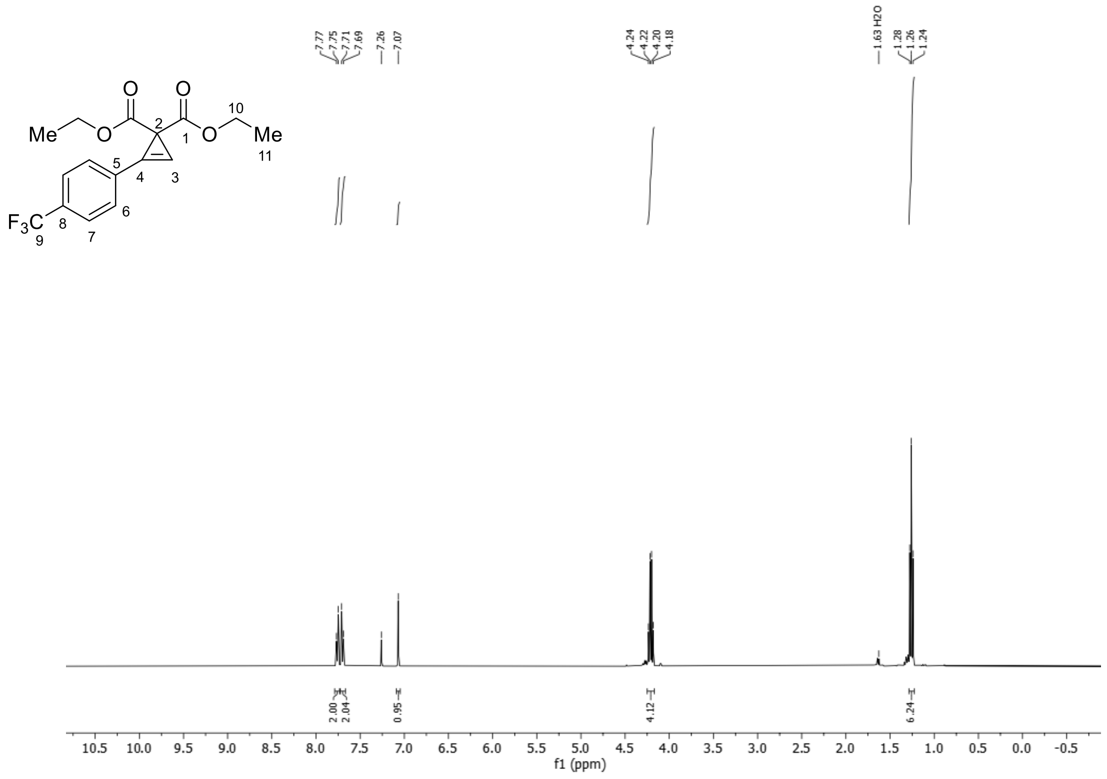

Figure S44. ^{1}H NMR of **S13** (400 MHz, 299 K, $CDCl_3$).

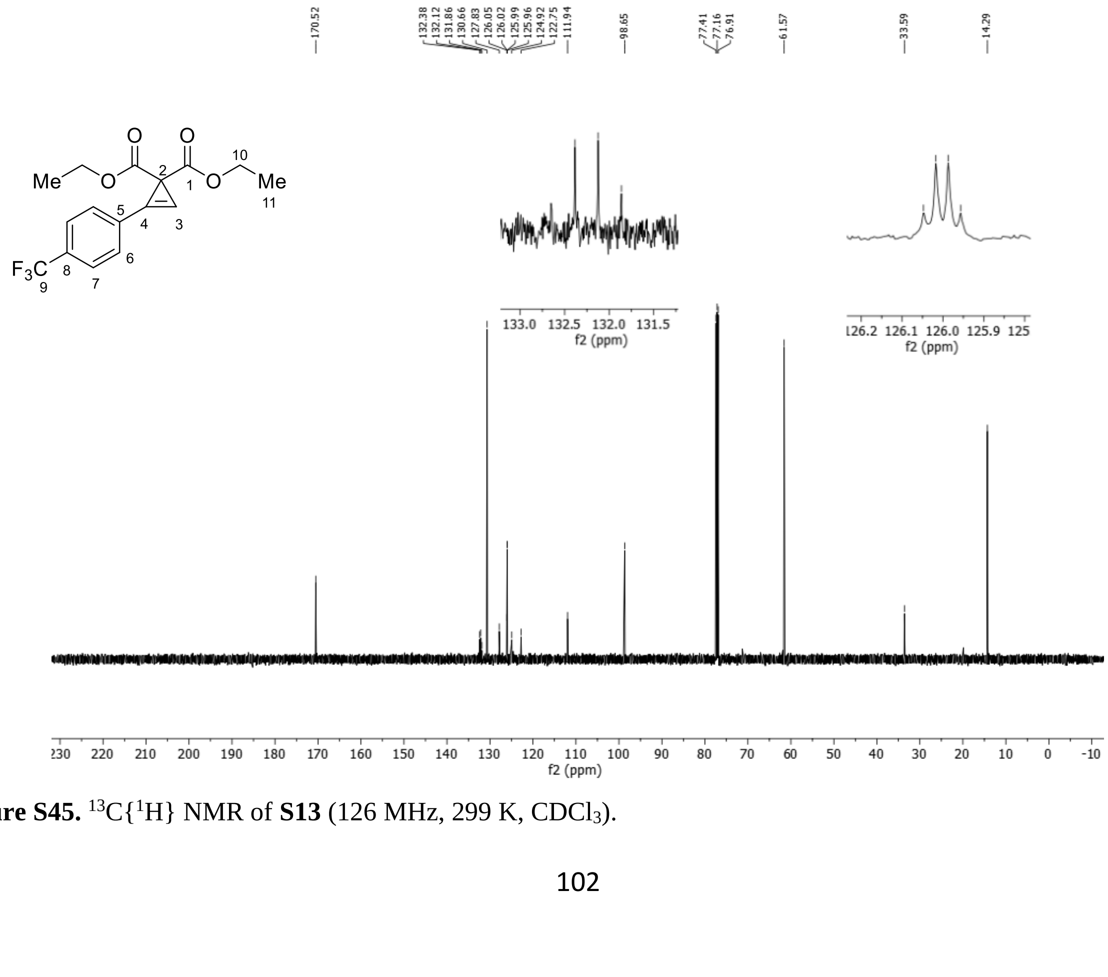

Figure S45. $^{13}C\{^{1}H\}$ NMR of **S13** (126 MHz, 299 K, $CDCl_3$).

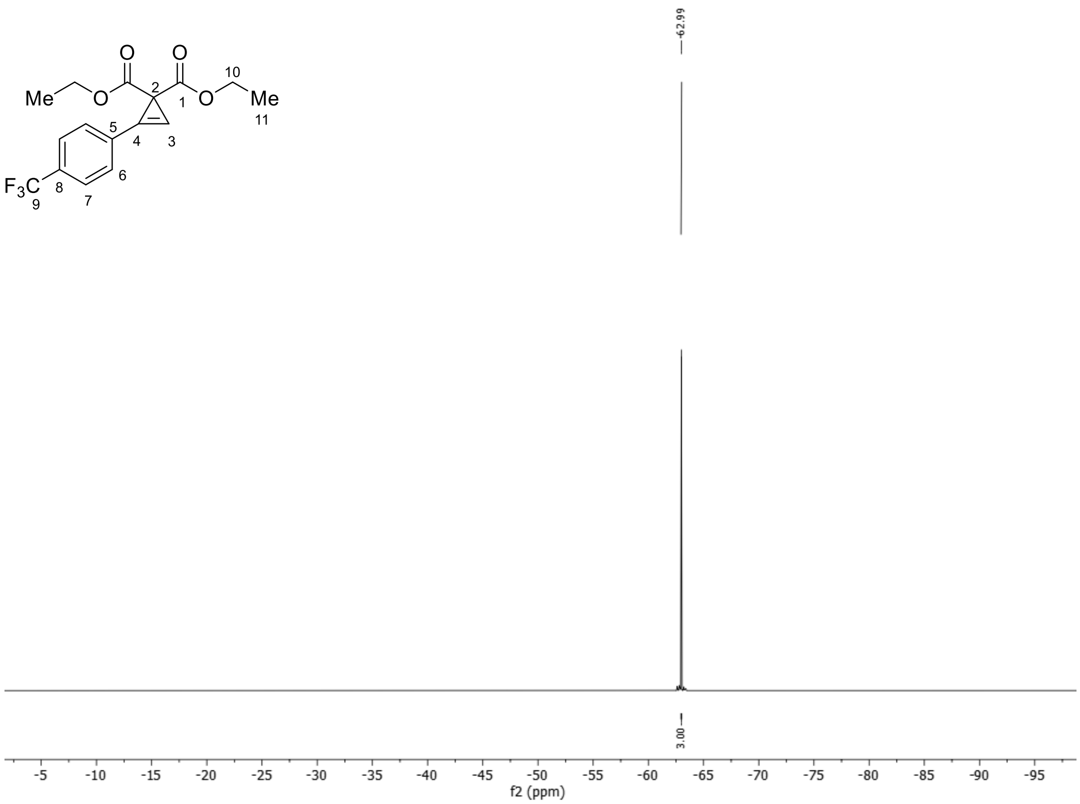

Figure S46. ^{19}F NMR of **S13** (470 MHz, 299 K, $CDCl_3$).

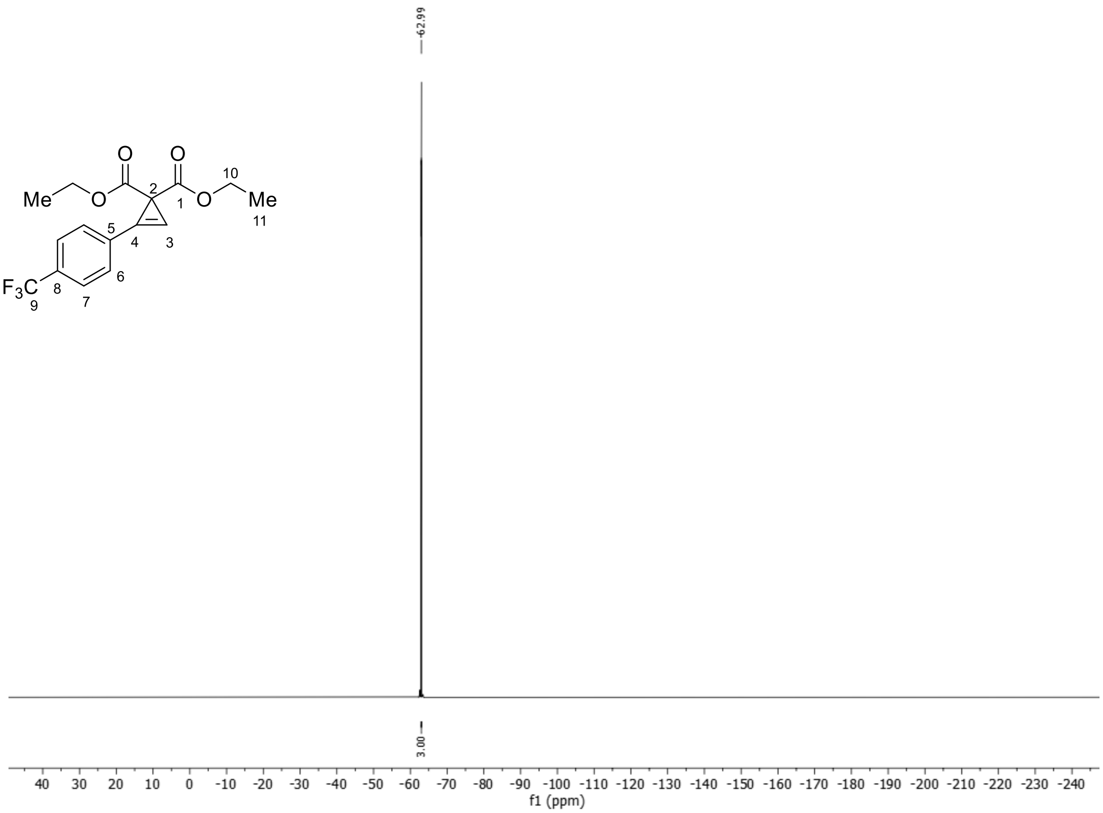

Figure S47. $^{19}F\{^1H\}$ NMR of **S13** (377 MHz, 299 K, $CDCl_3$).

Diethyl 2-(3,5-bis(trifluoromethyl)phenyl)cycloprop-2-ene-1,1-dicarboxylate (S14)

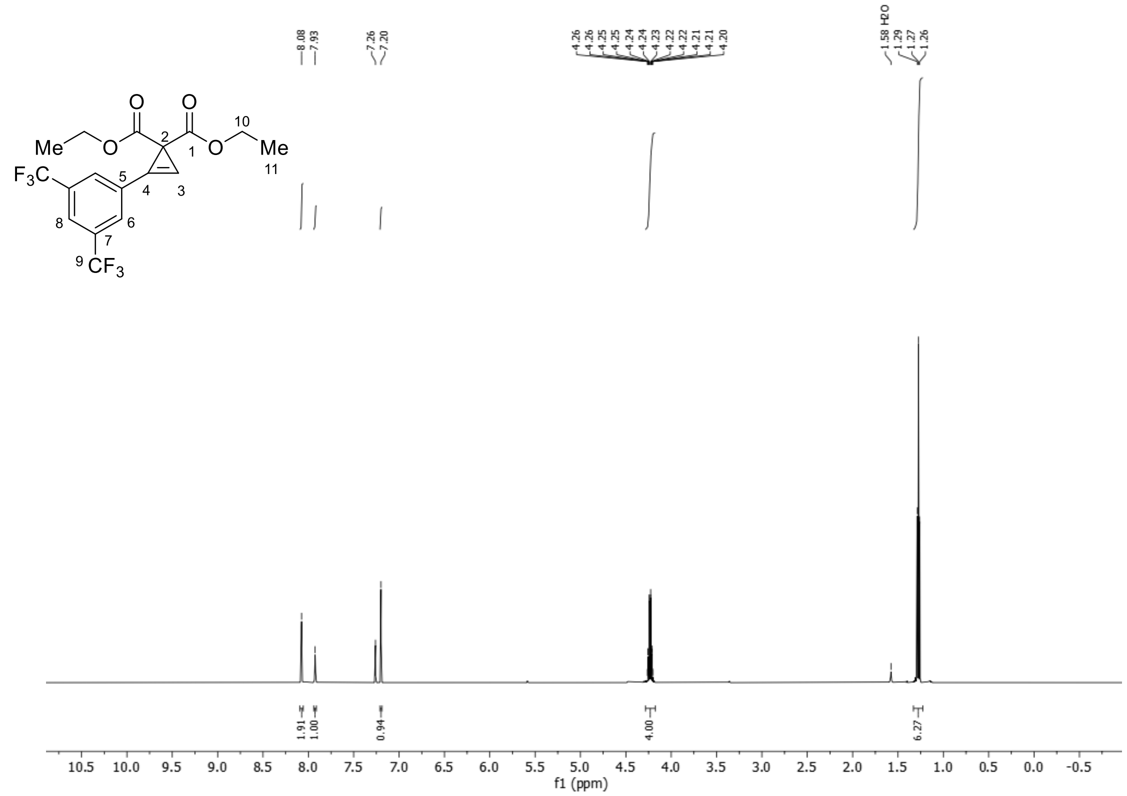

Figure S48. ^{1}H NMR of **S14** (500 MHz, 299 K, $CDCl_3$).

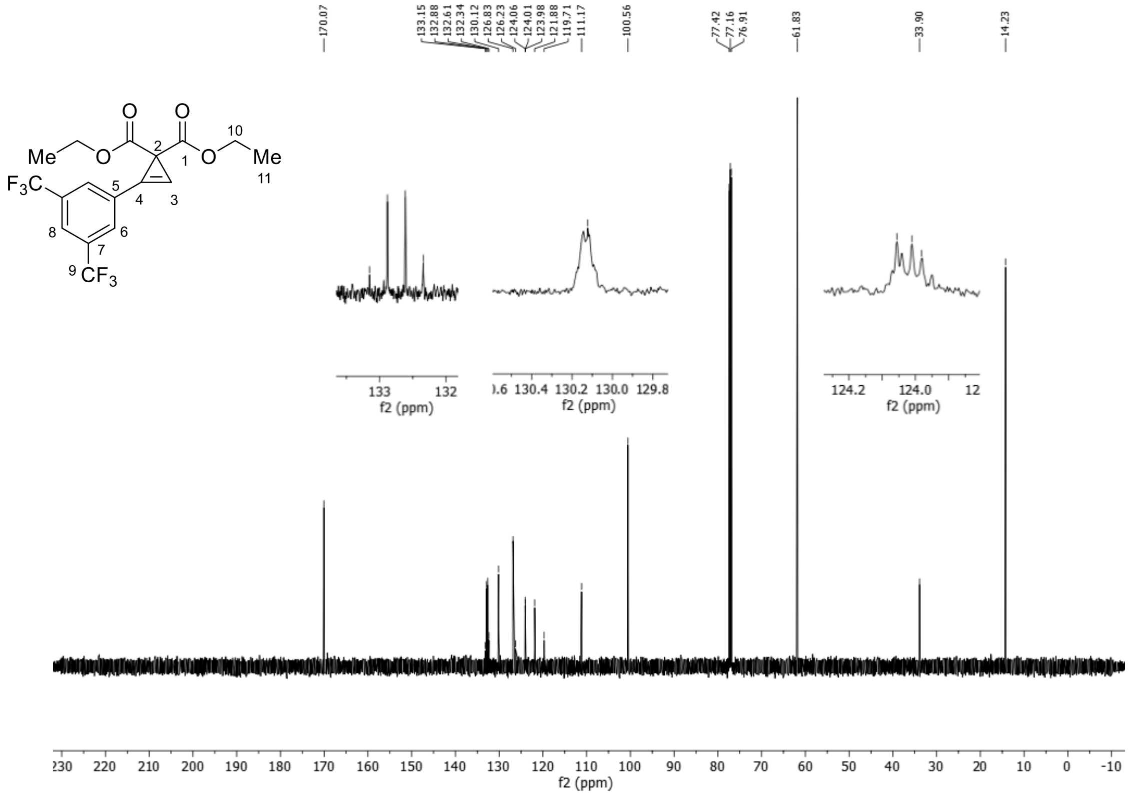

Figure S49. $^{13}C\{^{1}H\}$ NMR of **S14** (126 MHz, 299 K, $CDCl_3$).

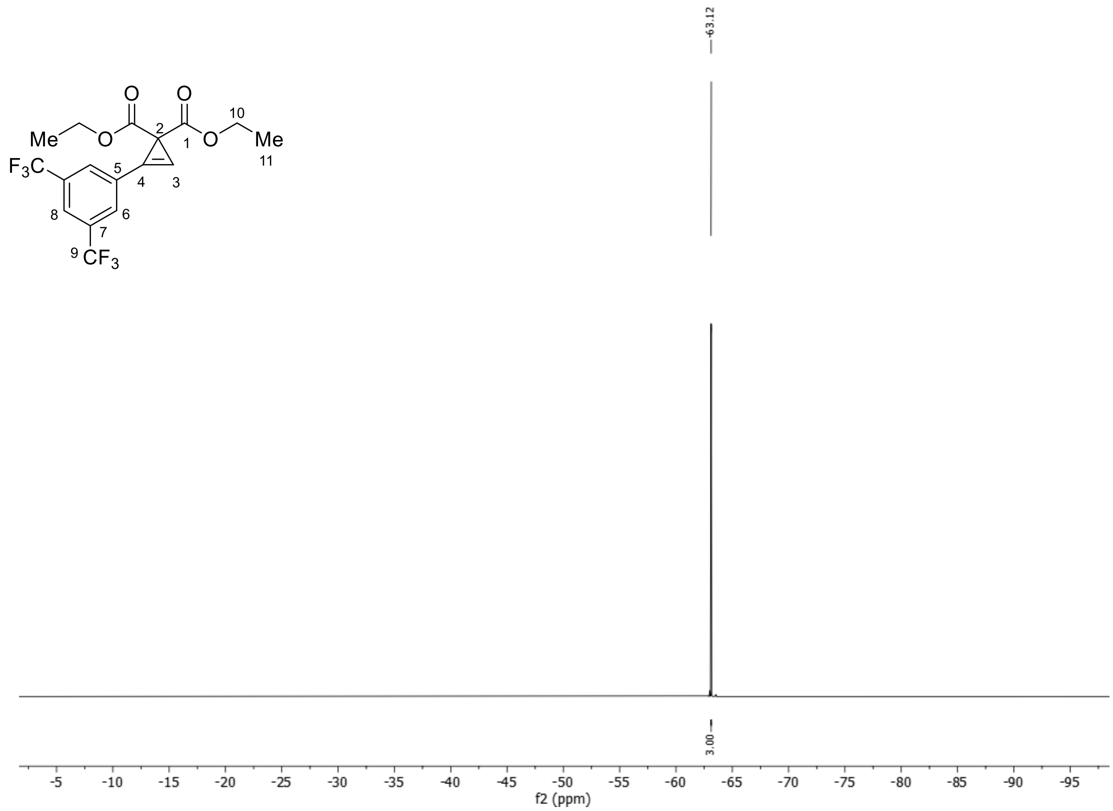

Figure S50. ^{19}F NMR of **S14** (470 MHz, 299 K, $CDCl_3$).

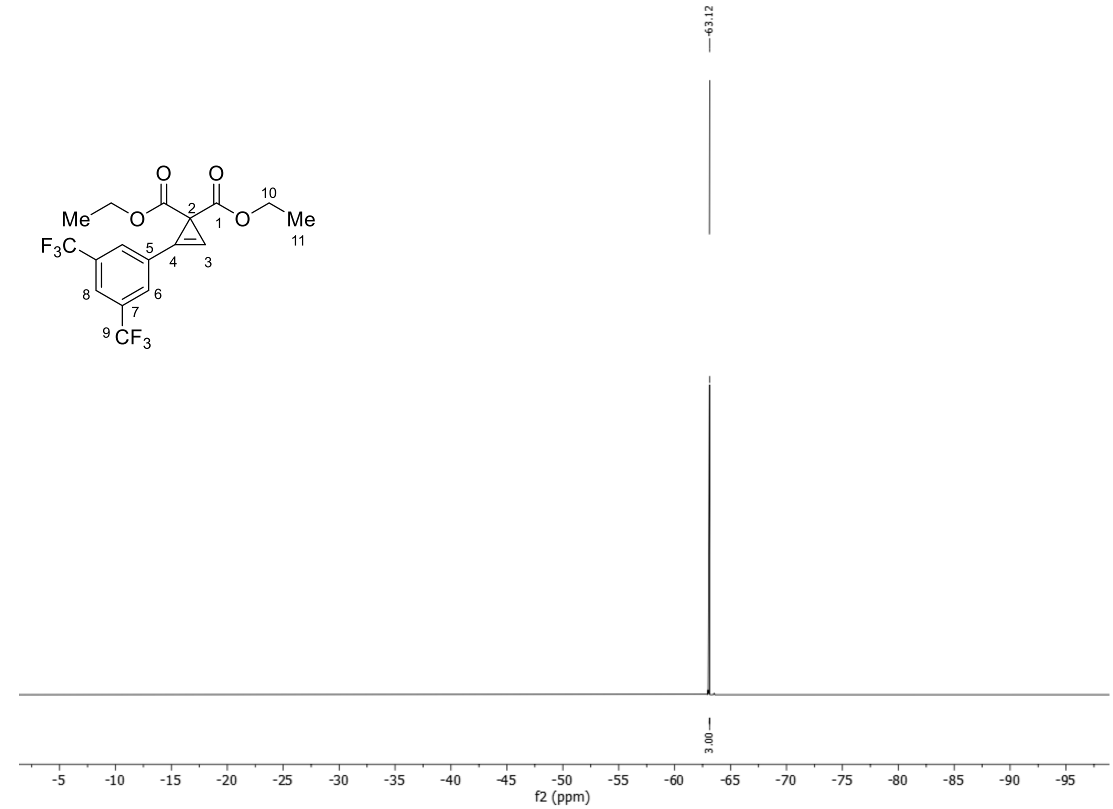

Figure S51. $^{19}F\{^1H\}$ NMR of **S14** (377 MHz, 299 K, $CDCl_3$).

Diethyl 2-(4-nitrophenyl)cycloprop-2-ene-1,1-dicarboxylate (S15)

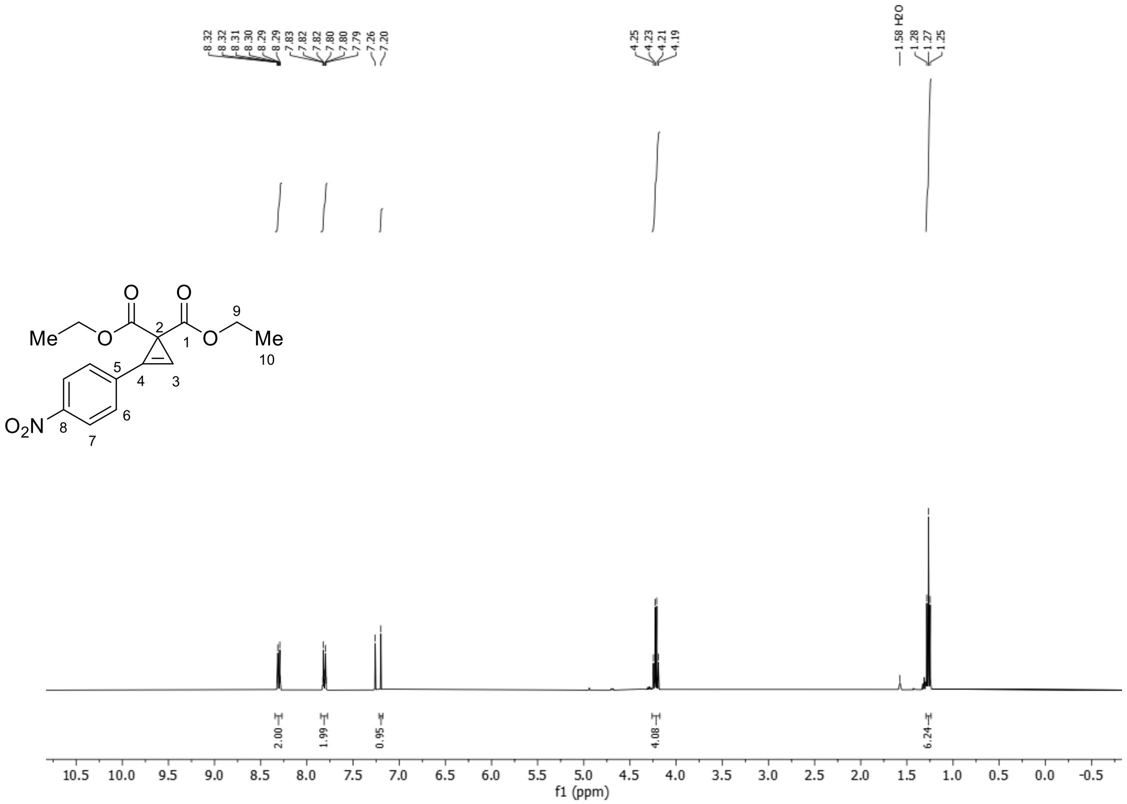

Figure S52. ^{1}H NMR of **S15** (400 MHz, 299 K, $CDCl_3$).

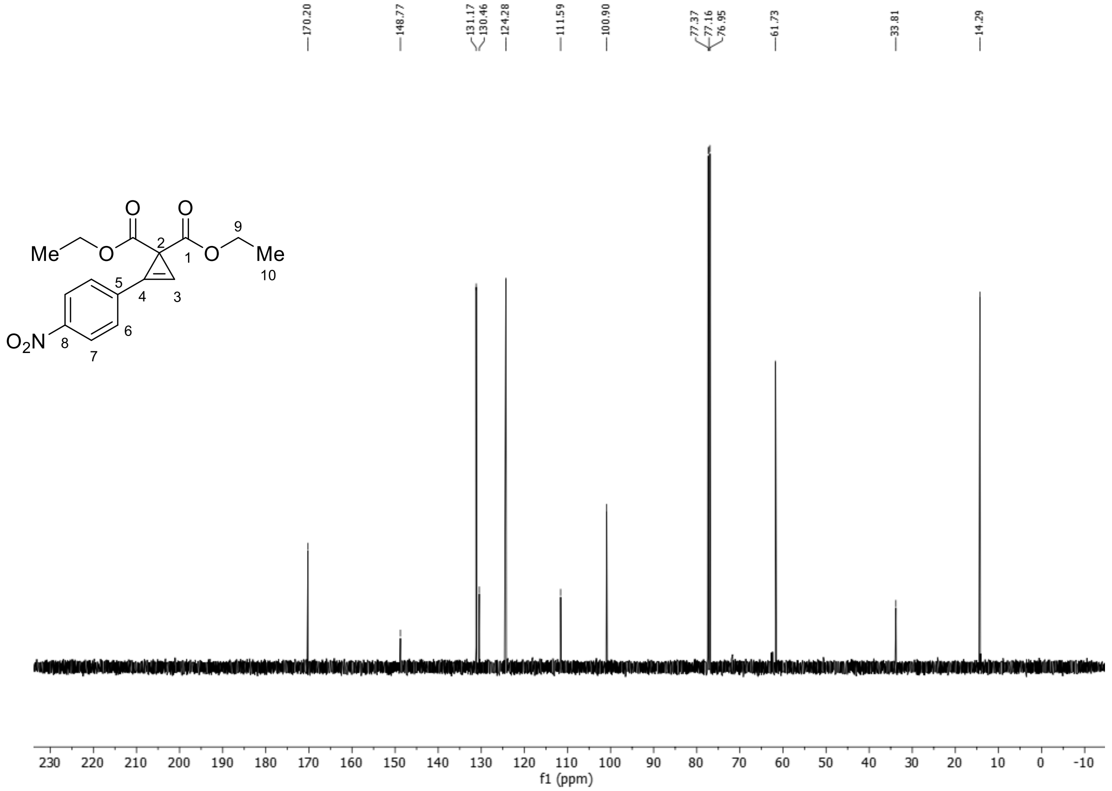

Figure S53. $^{13}C\{^{1}H\}$ NMR of **S15** (151 MHz, 299 K, $CDCl_3$).

Diethyl 2-(4-bromophenyl)cycloprop-2-ene-1,1-dicarboxylate (S16)

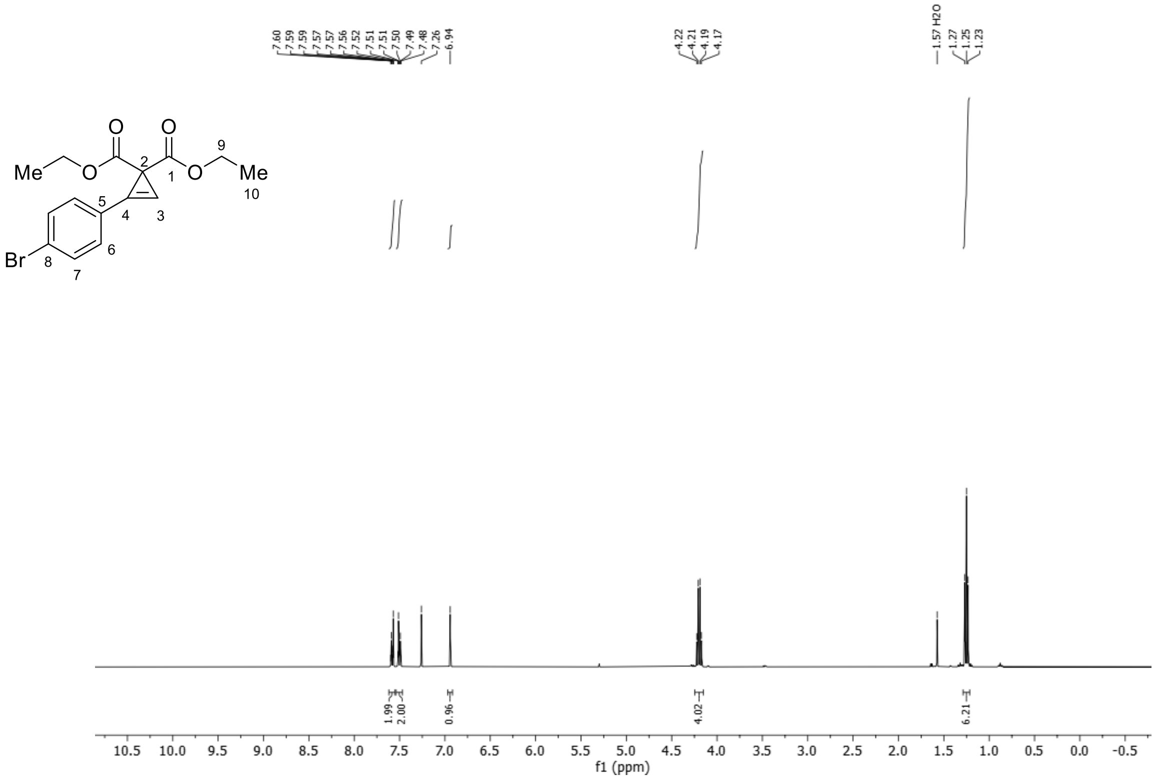

Figure S54. ^{1}H NMR of **S16** (400 MHz, 299 K, $CDCl_3$).

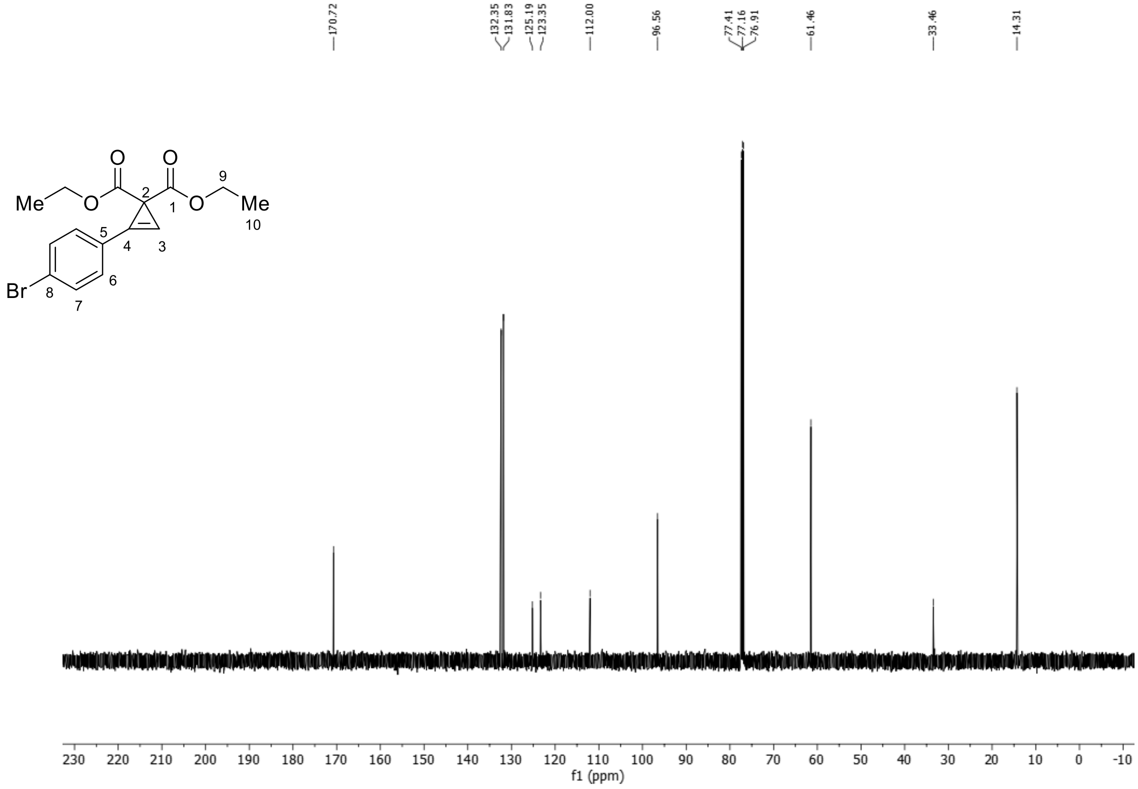

Figure S55. $^{13}C\{^{1}H\}$ NMR of **S16** (126 MHz, 299 K, $CDCl_3$).

Dimethyl 2-(4-(trifluoromethyl)phenyl)cycloprop-2-ene-1,1-dicarboxylate (S17)

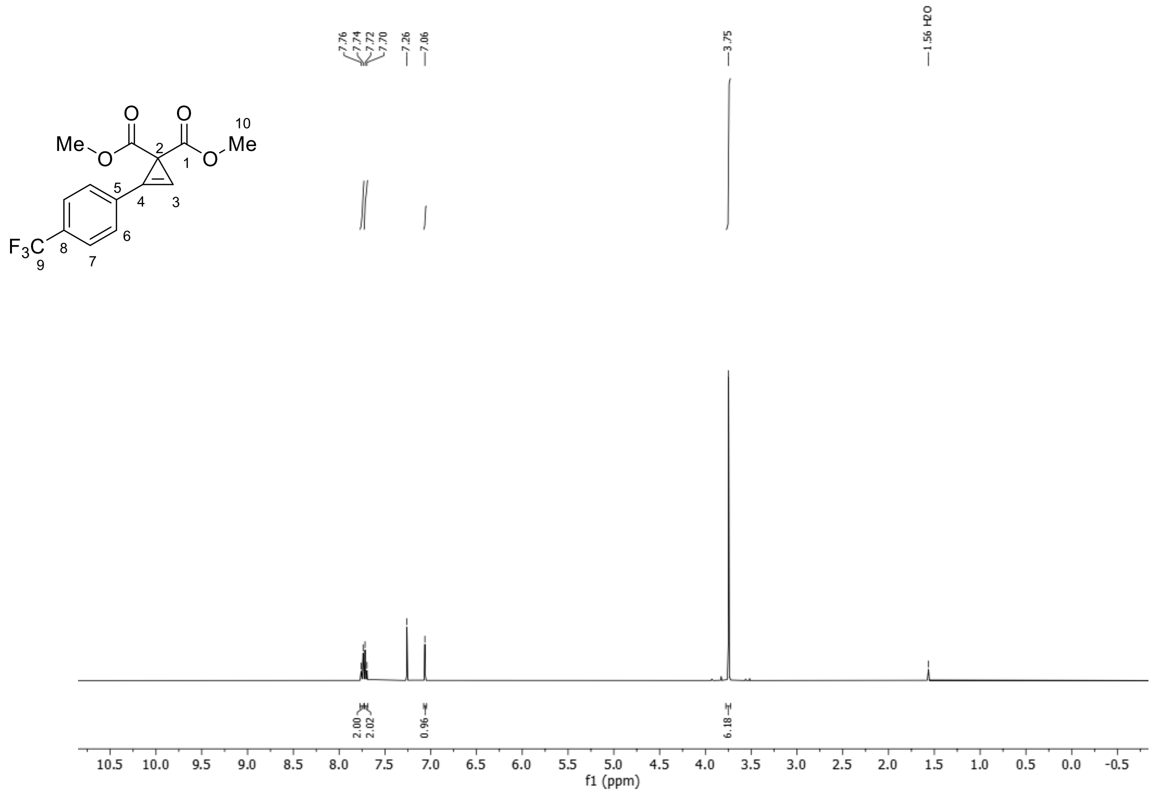

Figure S56. ^{1}H NMR of **S17** (400 MHz, 299 K, $CDCl_3$).

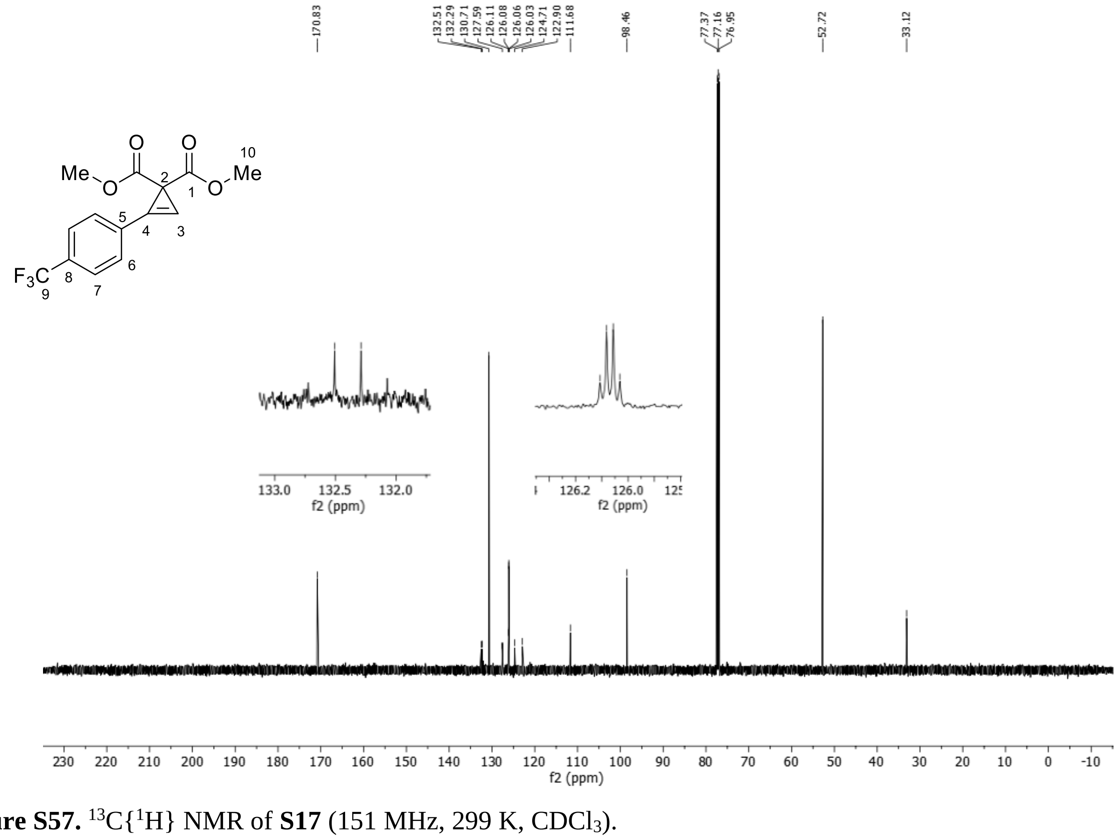

Figure S57. $^{13}C\{^{1}H\}$ NMR of **S17** (151 MHz, 299 K, $CDCl_3$).

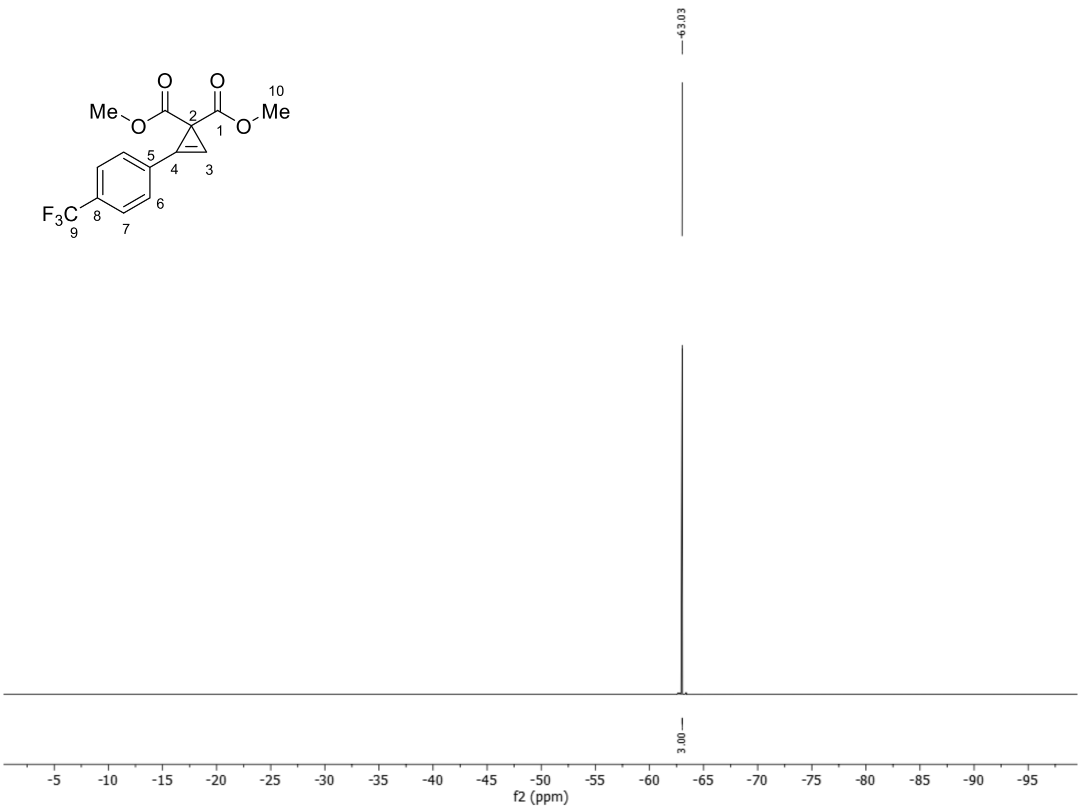

Figure S58. ^{19}F NMR of **S17** (564 MHz, 299 K, $CDCl_3$).

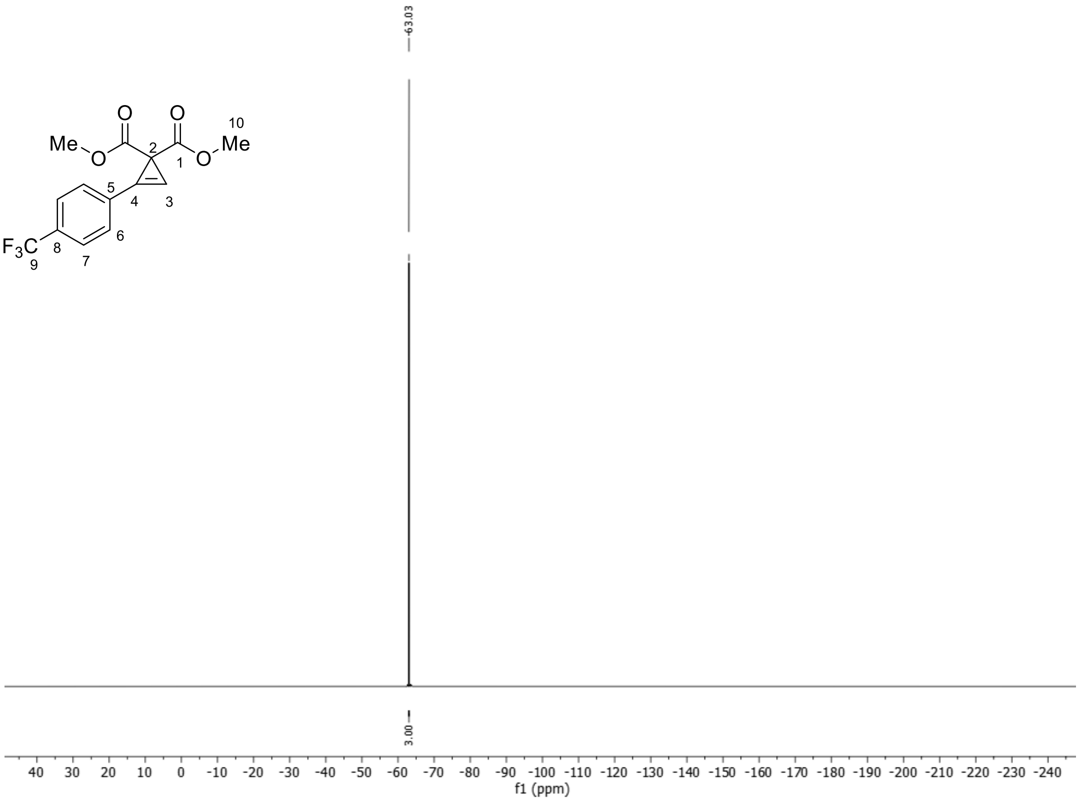

Figure S59. $^{19}F\{^{1}H\}$ NMR of **S17** (377 MHz, 299 K, $CDCl_3$).

Ethyl 1-methyl-2-(4-(trifluoromethyl)phenyl)cycloprop-2-ene-1-carboxylate (S18)

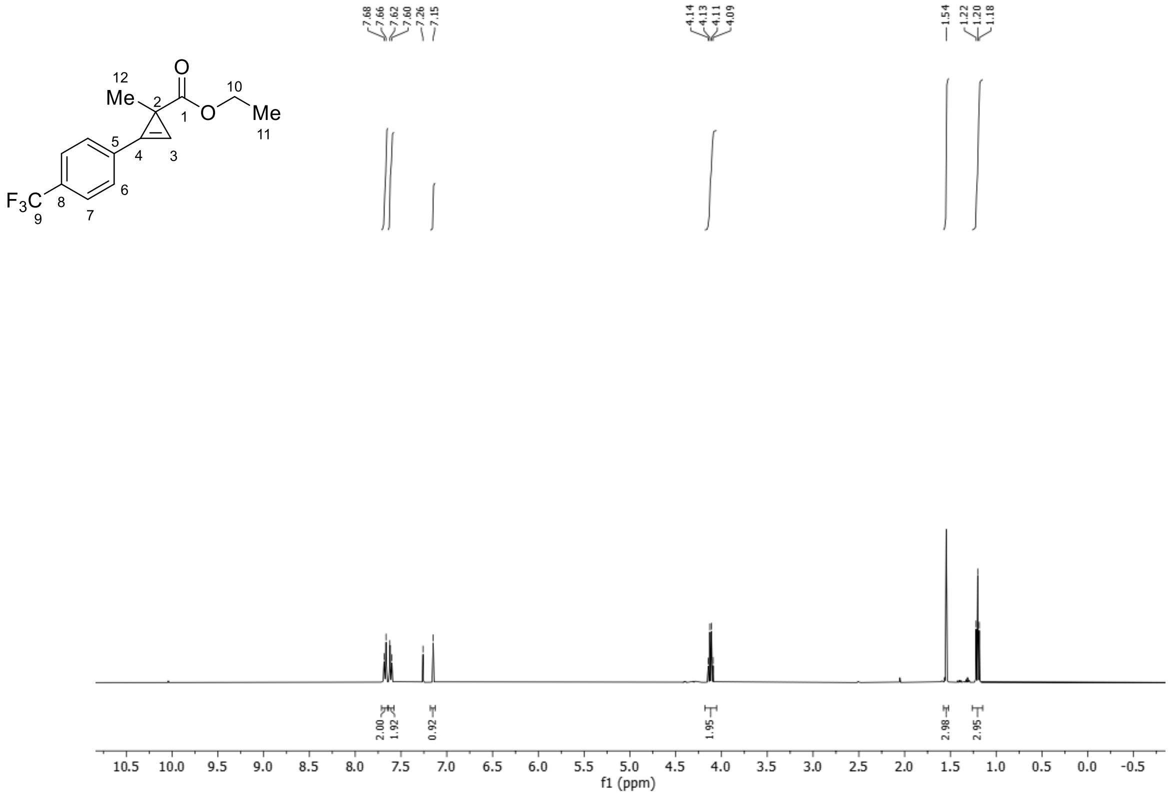

Figure S60. ^{1}H NMR of **S18** (400 MHz, 299 K, $CDCl_3$).

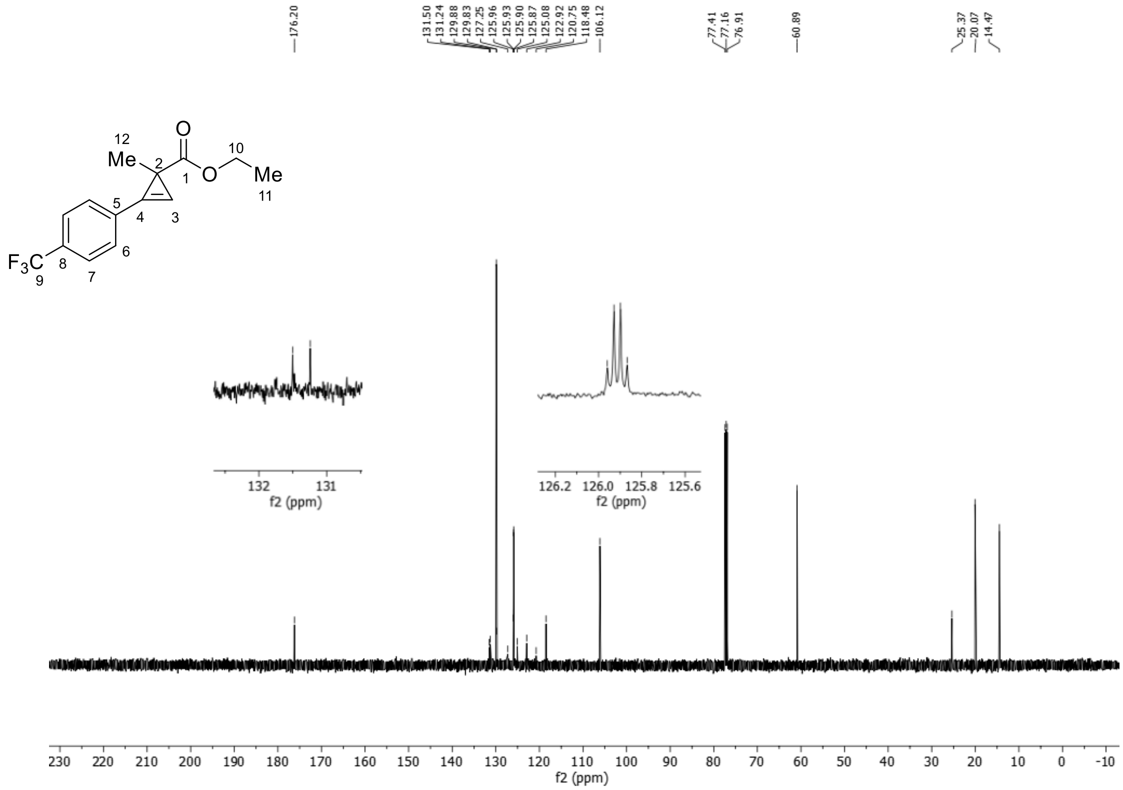

Figure S61. $^{13}C\{^{1}H\}$ NMR of **S18** (126 MHz, 299 K, $CDCl_3$).

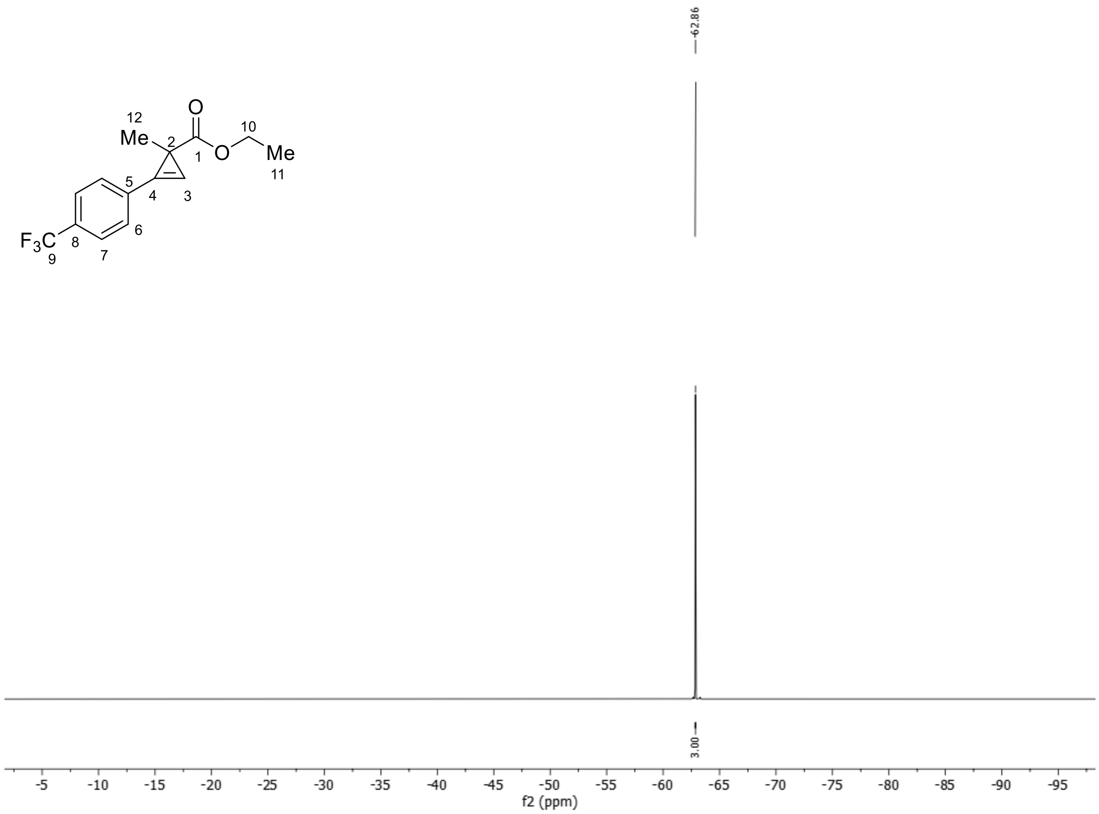

Figure S62. ^{19}F NMR of **S18** (470 MHz, 299 K, $CDCl_3$).

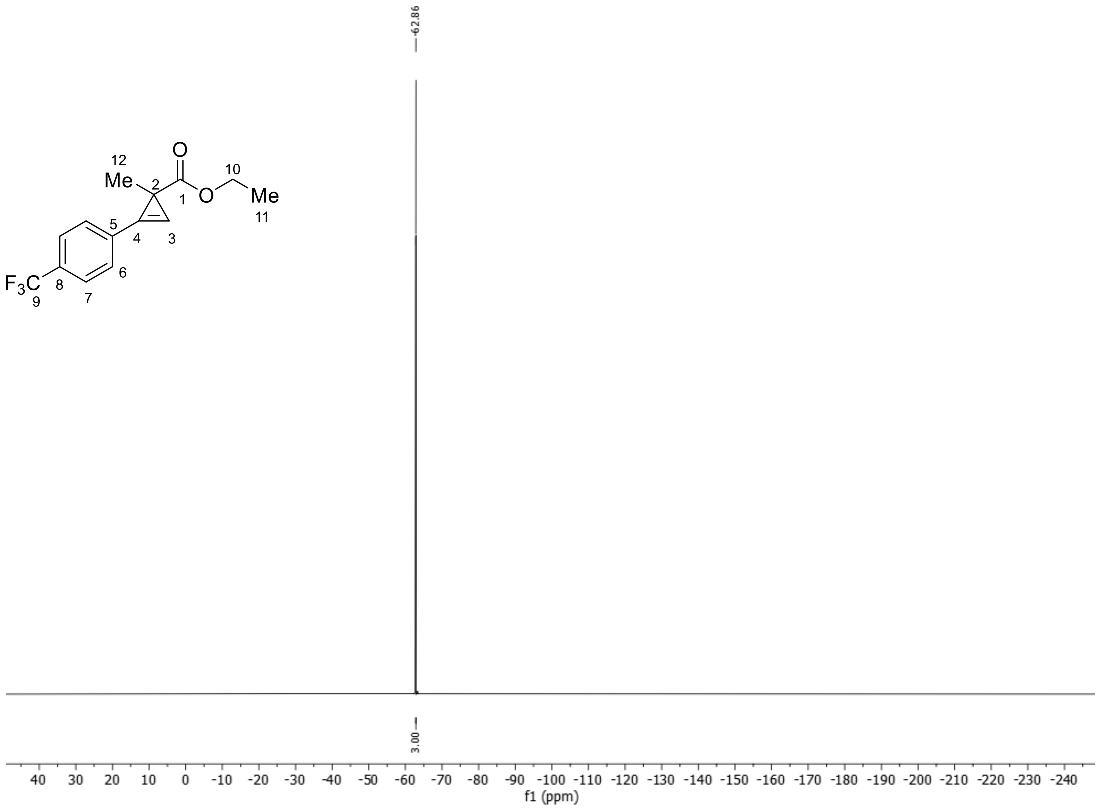

Figure S63. $^{19}F\{^1H\}$ NMR of **S18** (377 MHz, 299 K, $CDCl_3$).

Ethyl 1-phenyl-2-(4-(trifluoromethyl)phenyl)cycloprop-2-ene-1-carboxylate (S20)

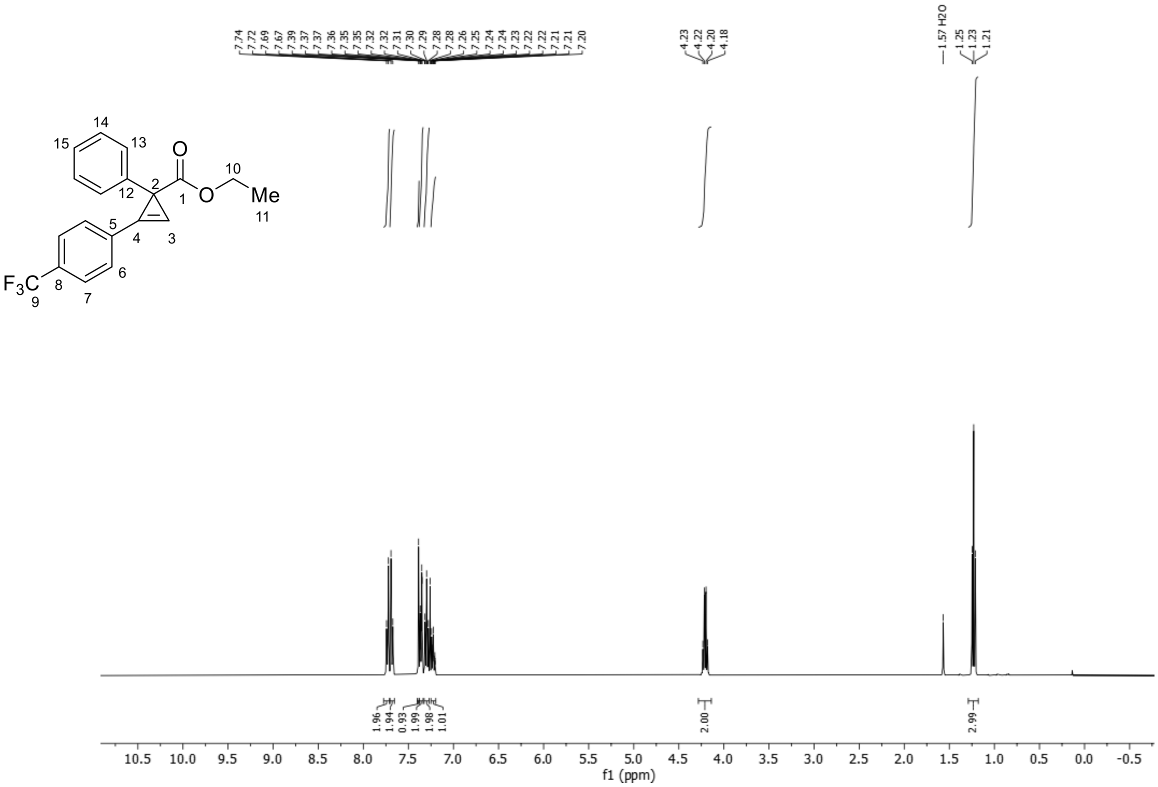

Figure S64. ^{1}H NMR of **S20** (400 MHz, 299 K, $CDCl_3$).

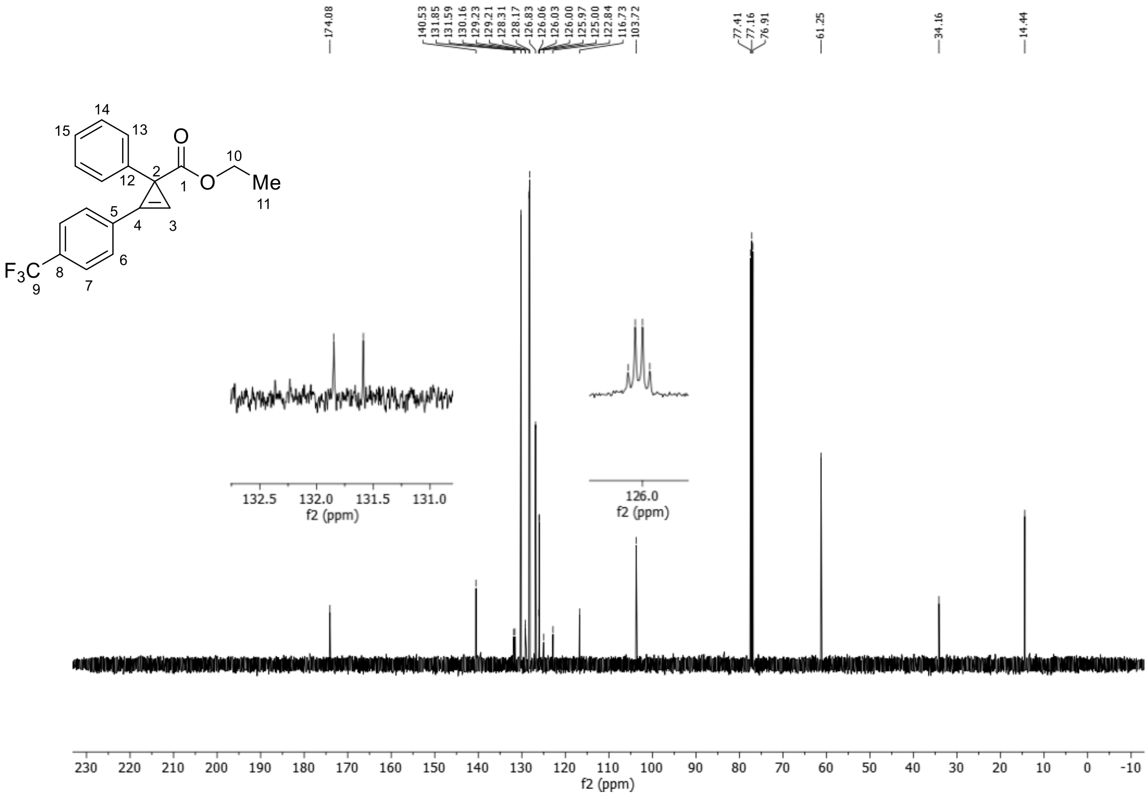

Figure S65. $^{13}C\{^1H\}$ NMR of **S20** (126 MHz, 299 K, $CDCl_3$).

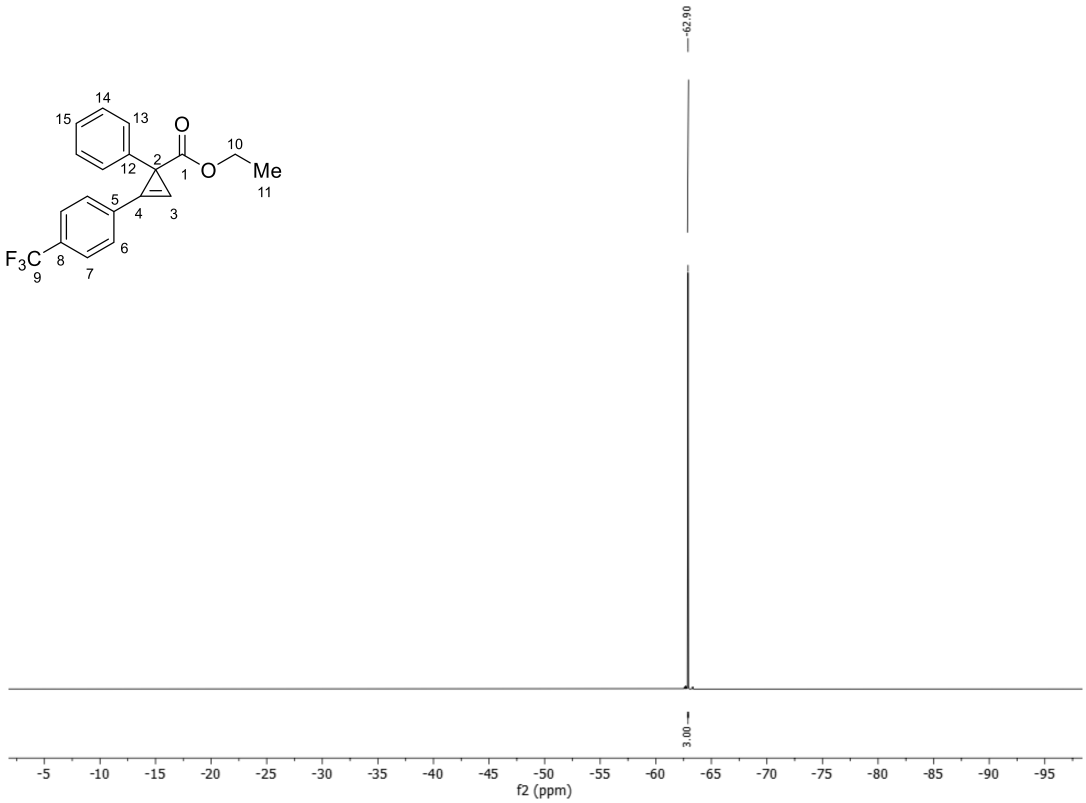

Figure S66. ^{19}F NMR of **S20** (470 MHz, 299 K, $CDCl_3$).

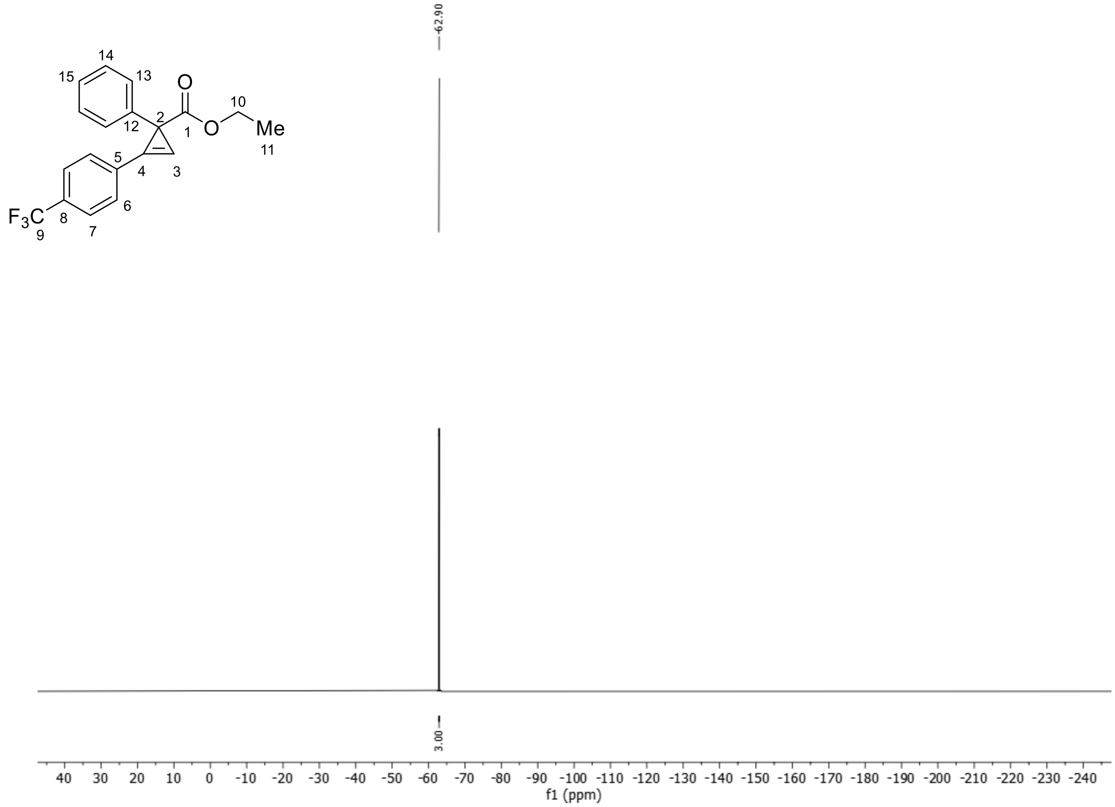

Figure S67. ^{19}F{^{1}H} NMR of **S20** (377 MHz, 299 K, $CDCl_3$).

Ethyl 2-hexyl-1-phenylcycloprop-2-ene-1-carboxylate (S21)

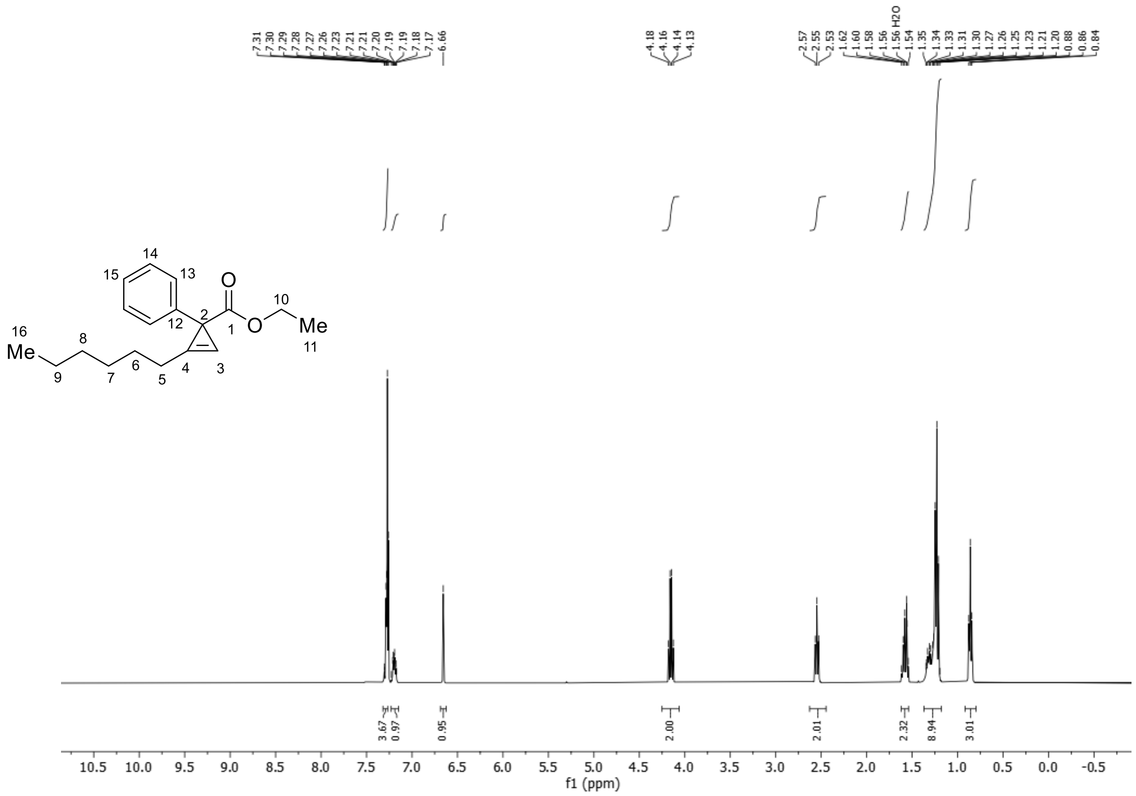

Figure S68. ^{1}H NMR of **S21** (400 MHz, 299 K, $CDCl_3$).

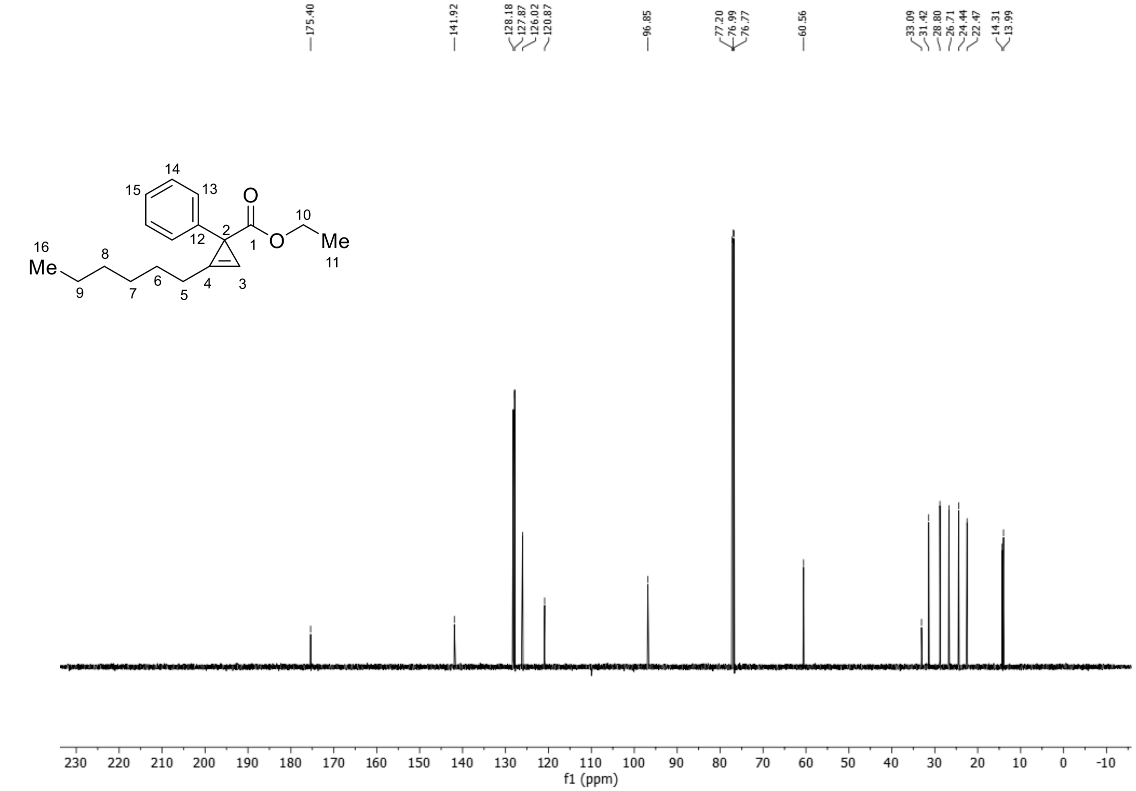

Figure S69. $^{13}C\{^{1}H\}$ NMR of **S21** (151 MHz, 299 K, $CDCl_3$).

(3R,8R,10S,13R)-10,13-Dimethyl-17-((R)-6-methylheptan-2-yl)hexadecahydro-1H-cyclopenta[a]phenanthren-3-yl 4-(3-(4-bromophenyl)-3-(trifluoromethyl)cycloprop-1-en-1-yl)benzoate (S22)

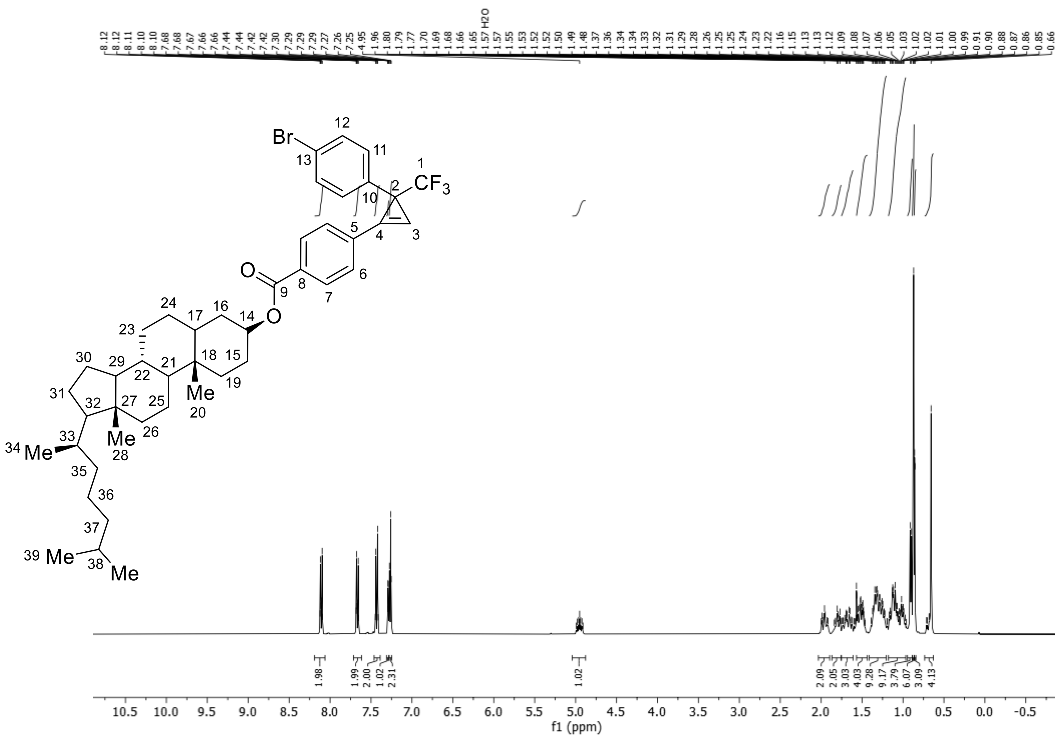

Figure S70. ^{1}H NMR of **S22** (400 MHz, 299 K, $CDCl_3$).

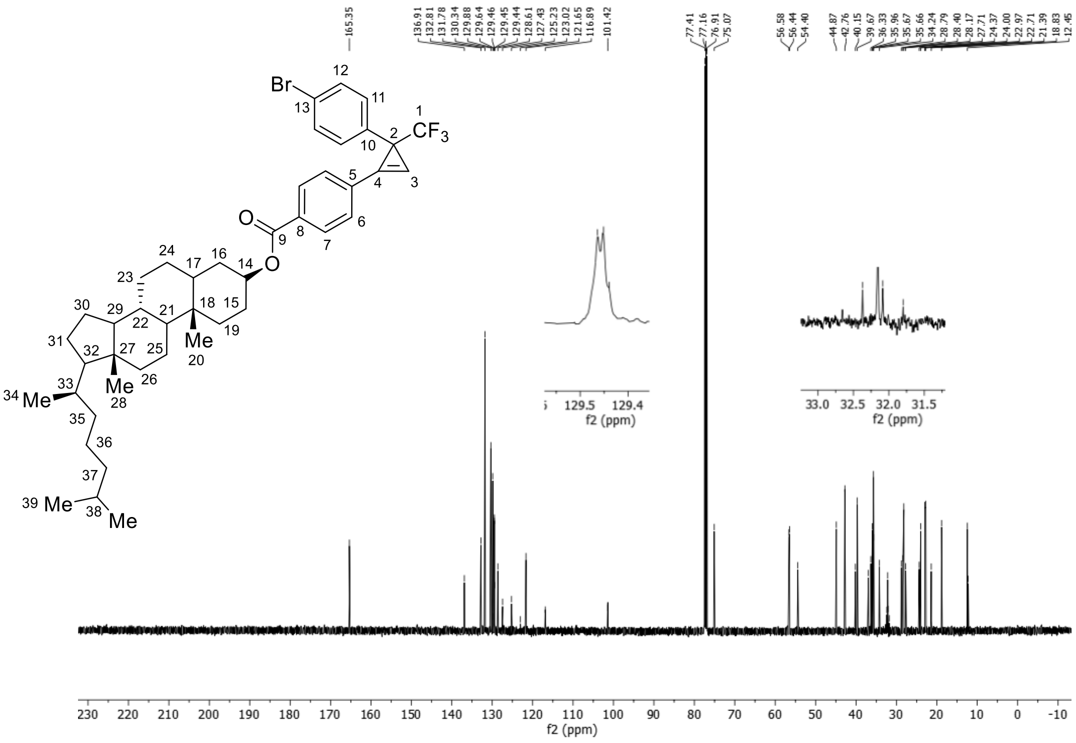

Figure S71. $^{13}C\{^{1}H\}$ NMR of **S22** (126 MHz, 299 K, $CDCl_3$).

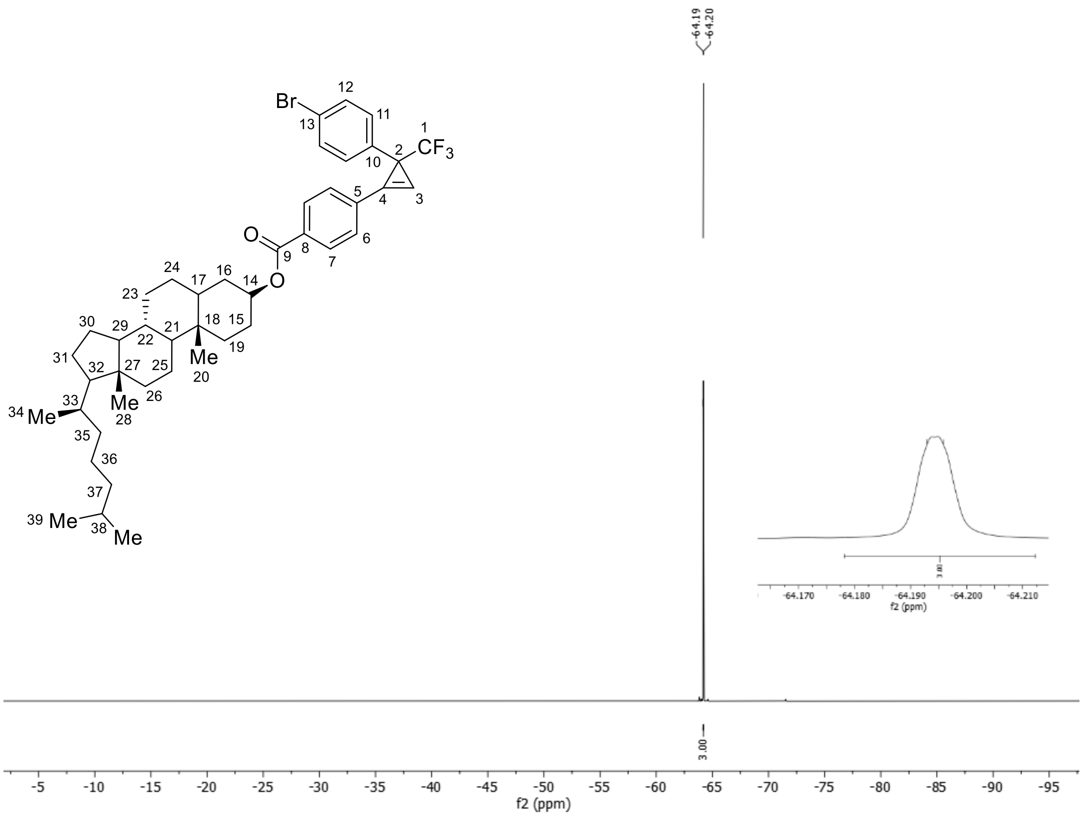

Figure S72. ^{19}F NMR of **S22** (470 MHz, 299 K, $CDCl_3$).

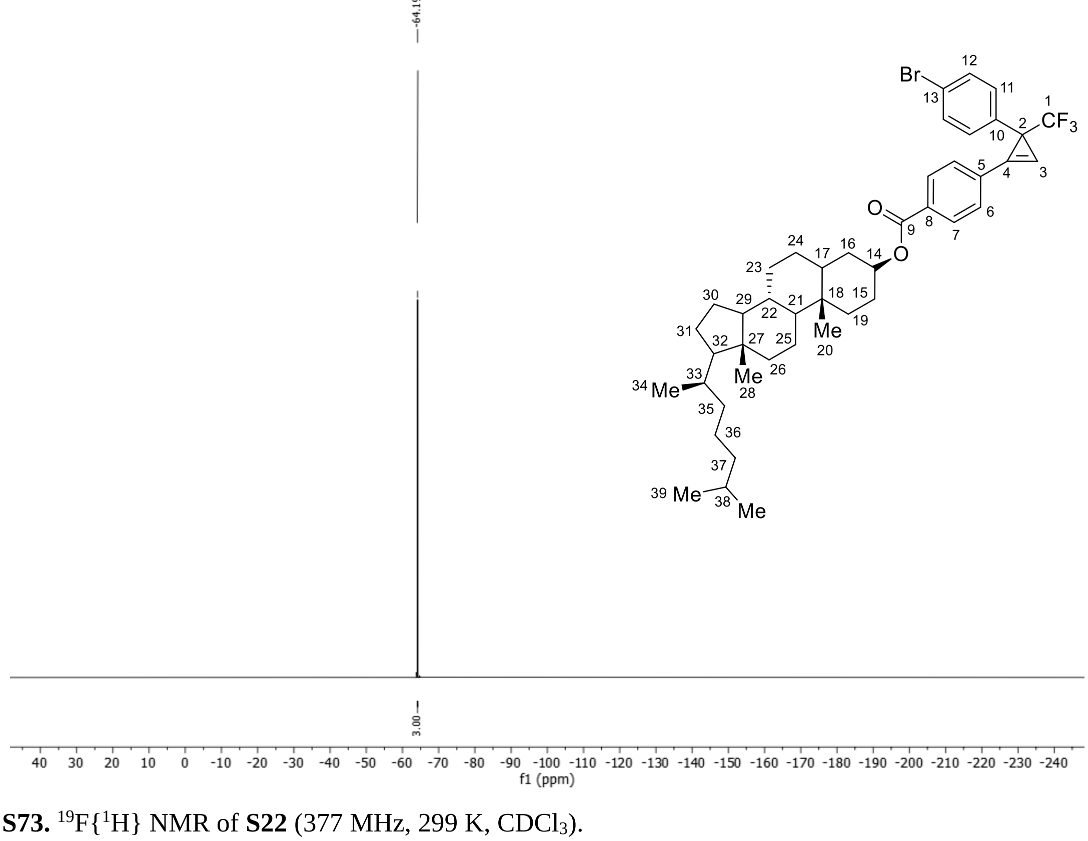

Figure S73. $^{19}F\{^1H\}$ NMR of **S22** (377 MHz, 299 K, $CDCl_3$).

Ethyl 2-(9-hydroxynonyl)cycloprop-2-ene-1-carboxylate (S24)

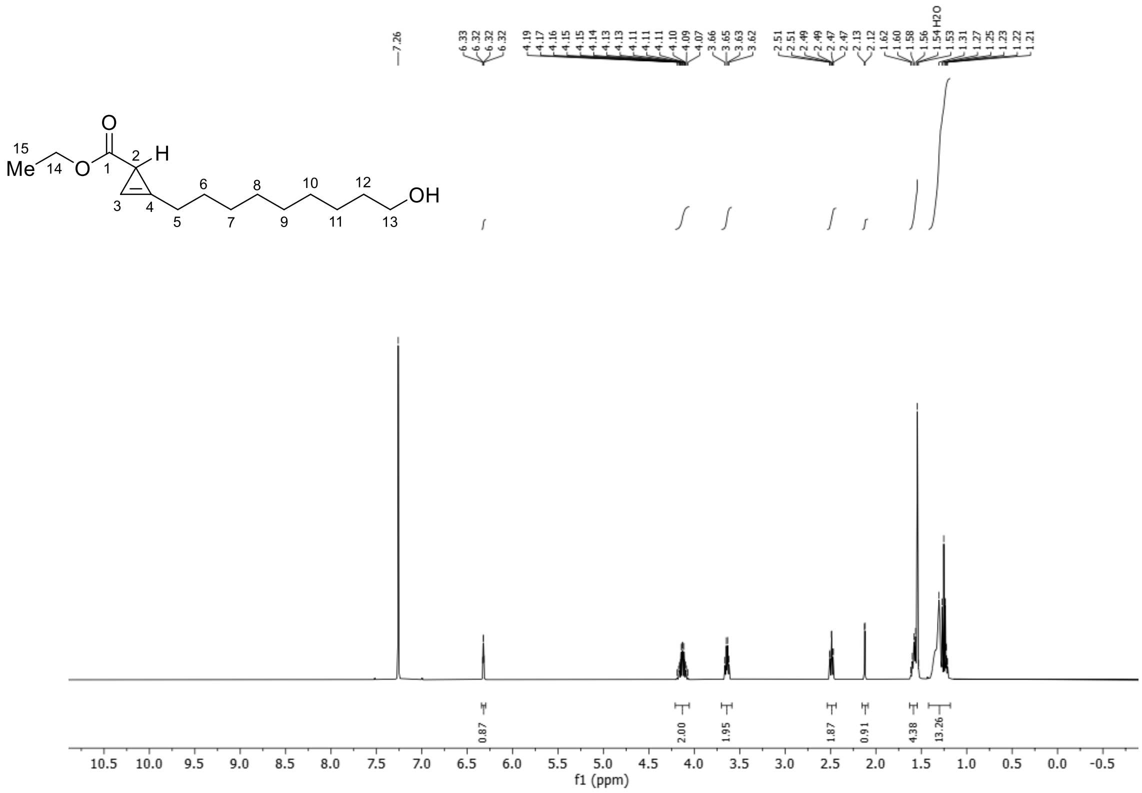

Figure S74. ^{1}H NMR of **S24** (400 MHz, 299 K, $CDCl_3$).

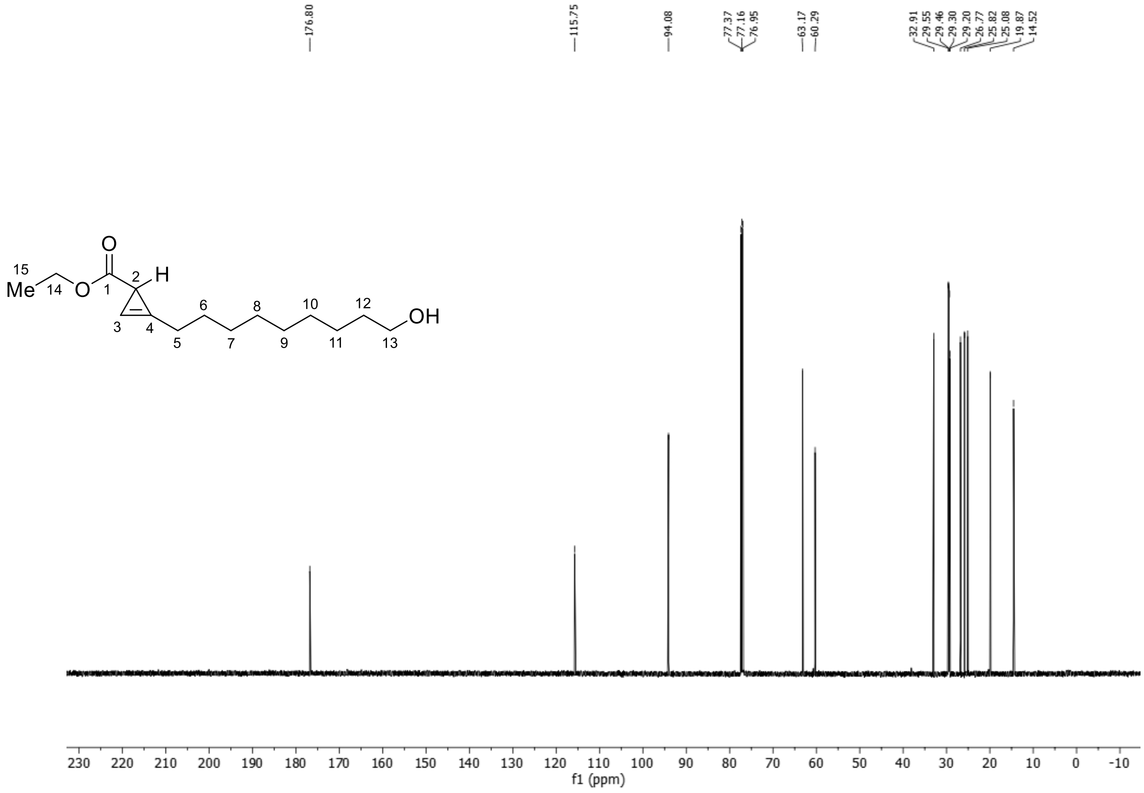

Figure S75. $^{13}C\{^{1}H\}$ NMR of **S24** (151 MHz, 299 K, $CDCl_3$).

9-(3-(Ethoxycarbonyl)cycloprop-1-en-1-yl)nonyl nicotinate (S25)

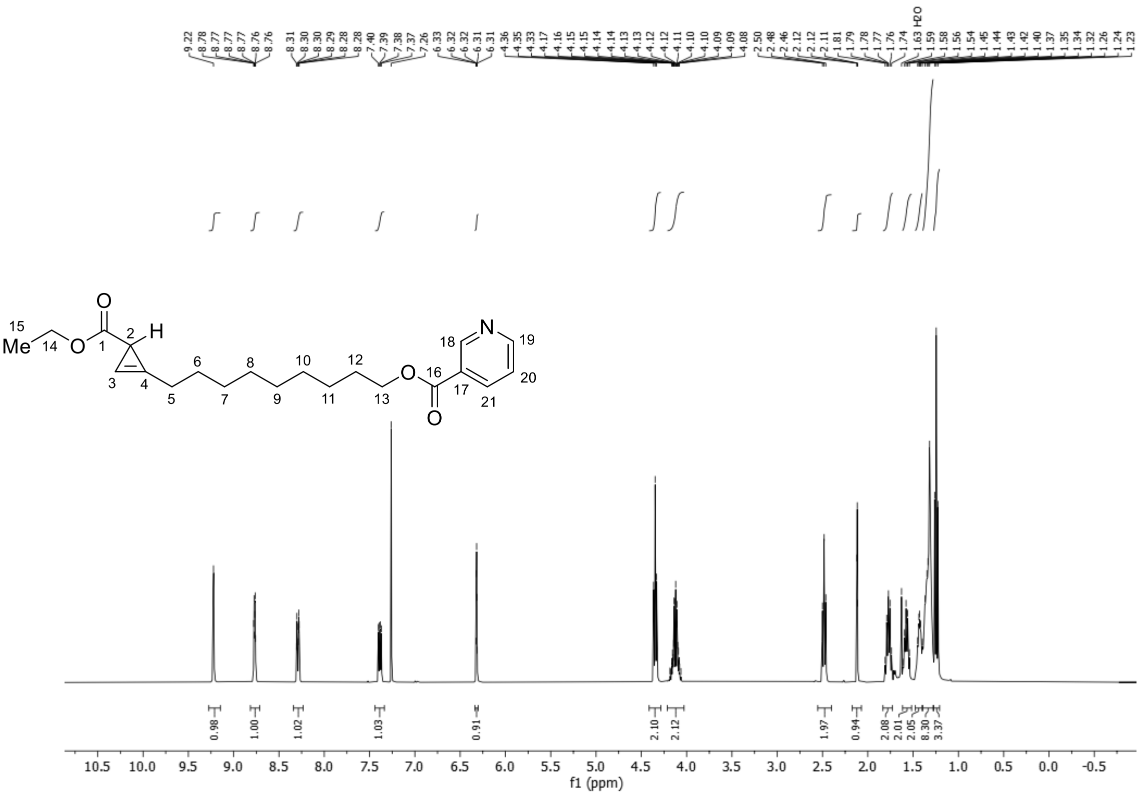

Figure S76. ^{1}H NMR of **S25** (400 MHz, 299 K, $CDCl_3$).

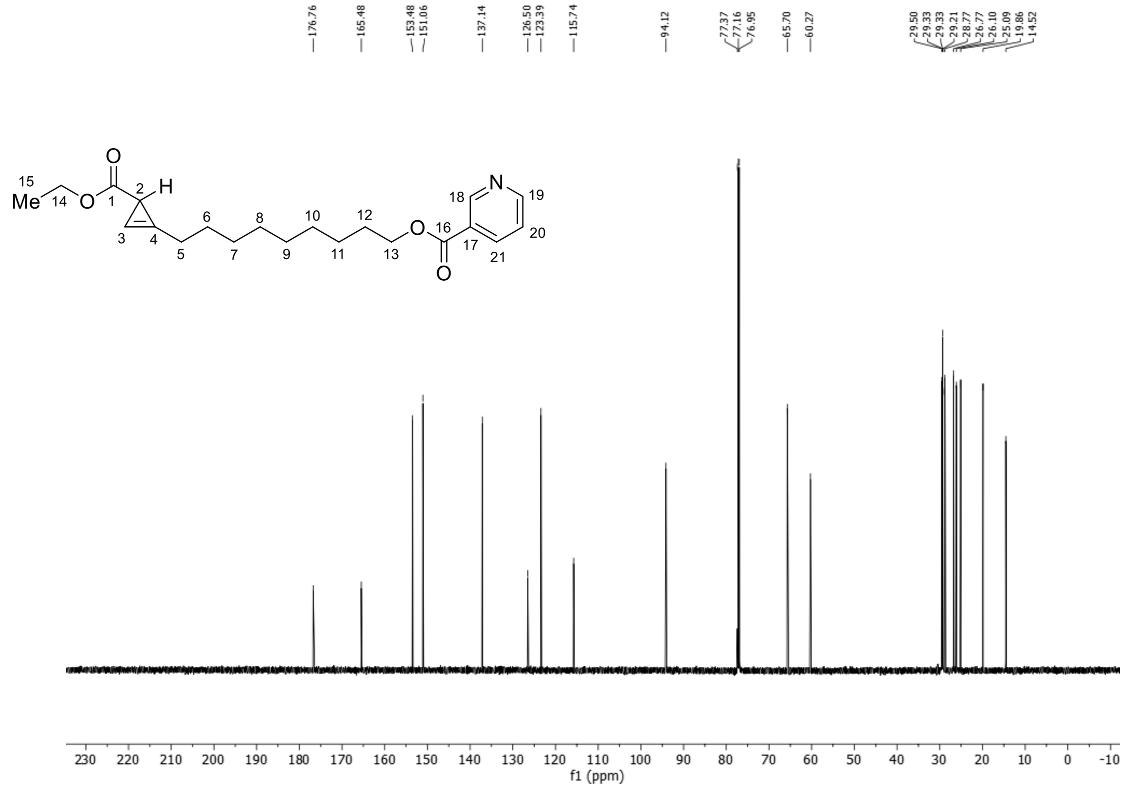

Figure S77. $^{13}C\{^{1}H\}$ NMR of **S25** (151 MHz, 299 K, $CDCl_3$).

1-(1,2,2-Tribromo-3,3-dimethylcyclopropyl)-4-(trifluoromethyl)benzene (Int 14)

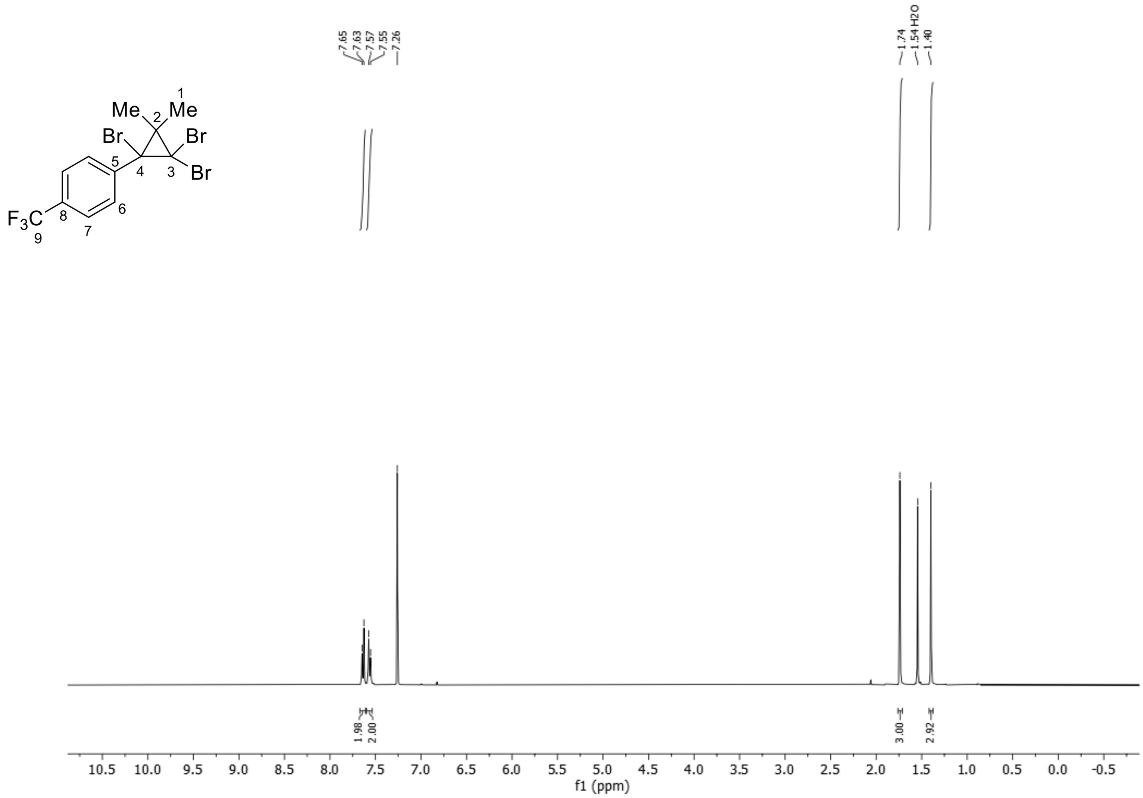

Figure S78. ^{1}H NMR of **Int 14** (400 MHz, 299 K, $CDCl_3$).

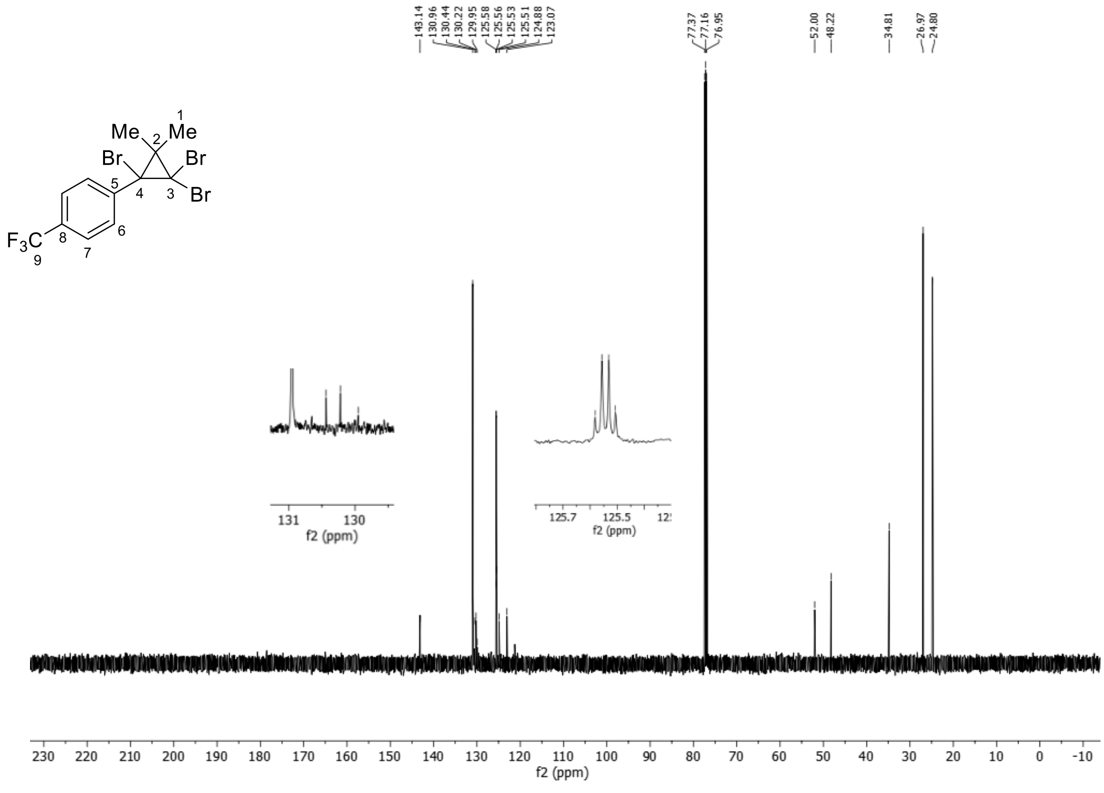

Figure S79. $^{13}C\{^{1}H\}$ NMR of **Int 14** (151 MHz, 299 K, $CDCl_3$).

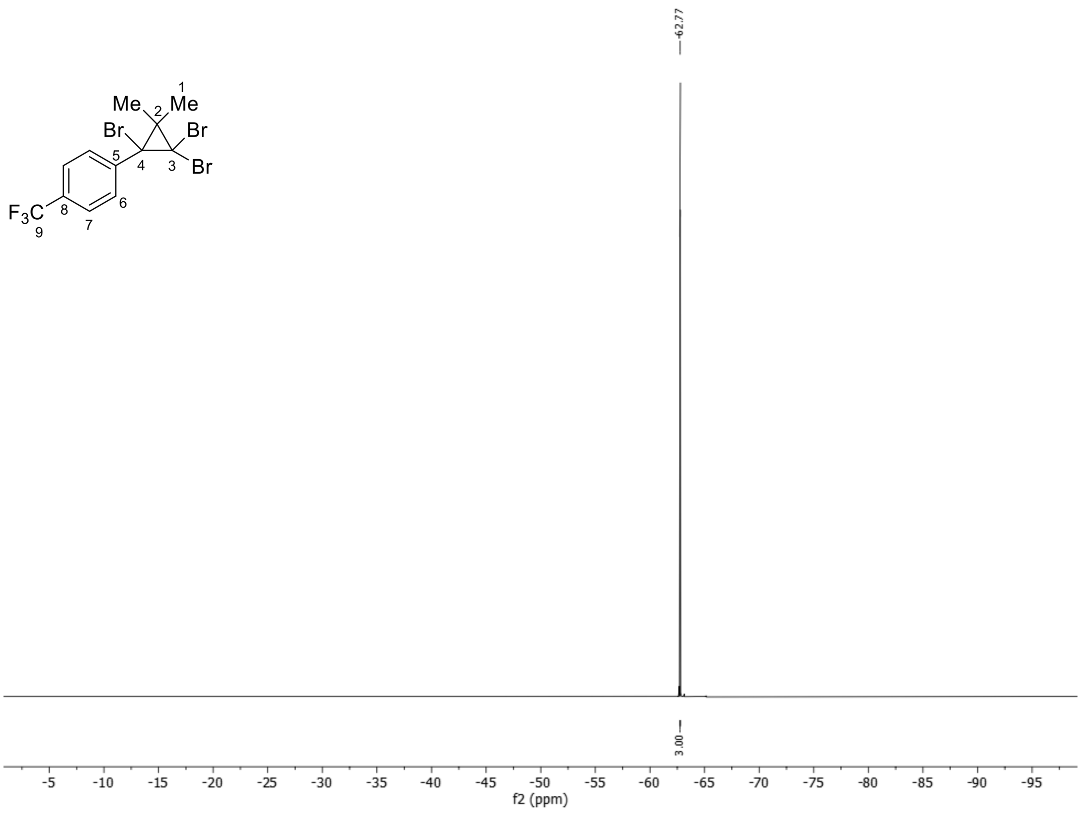

Figure S80. ^{19}F NMR of **Int 14** (564 MHz, 299 K, $CDCl_3$).

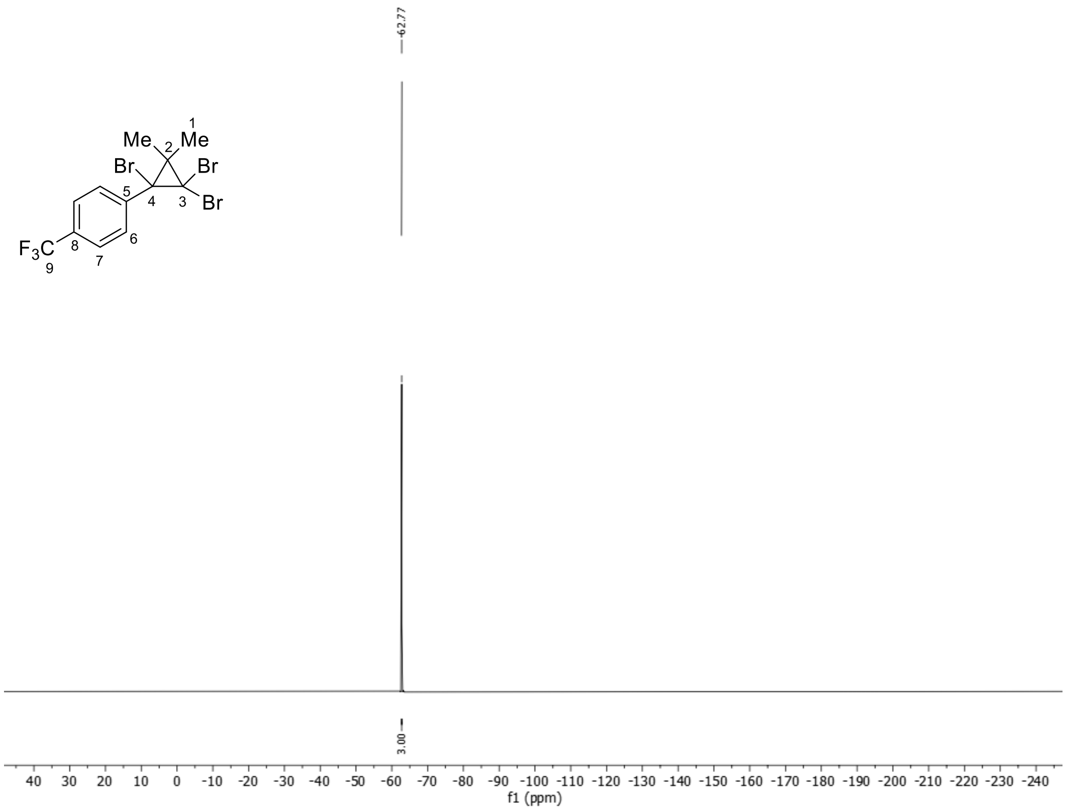

Figure S81. $^{19}F\{^1H\}$ NMR of **Int 14** (377 MHz, 299 K, $CDCl_3$).

Methyl 3,3-dimethyl-2-(4-(trifluoromethyl)phenyl)cycloprop-1-ene-1-carboxylate (S27)

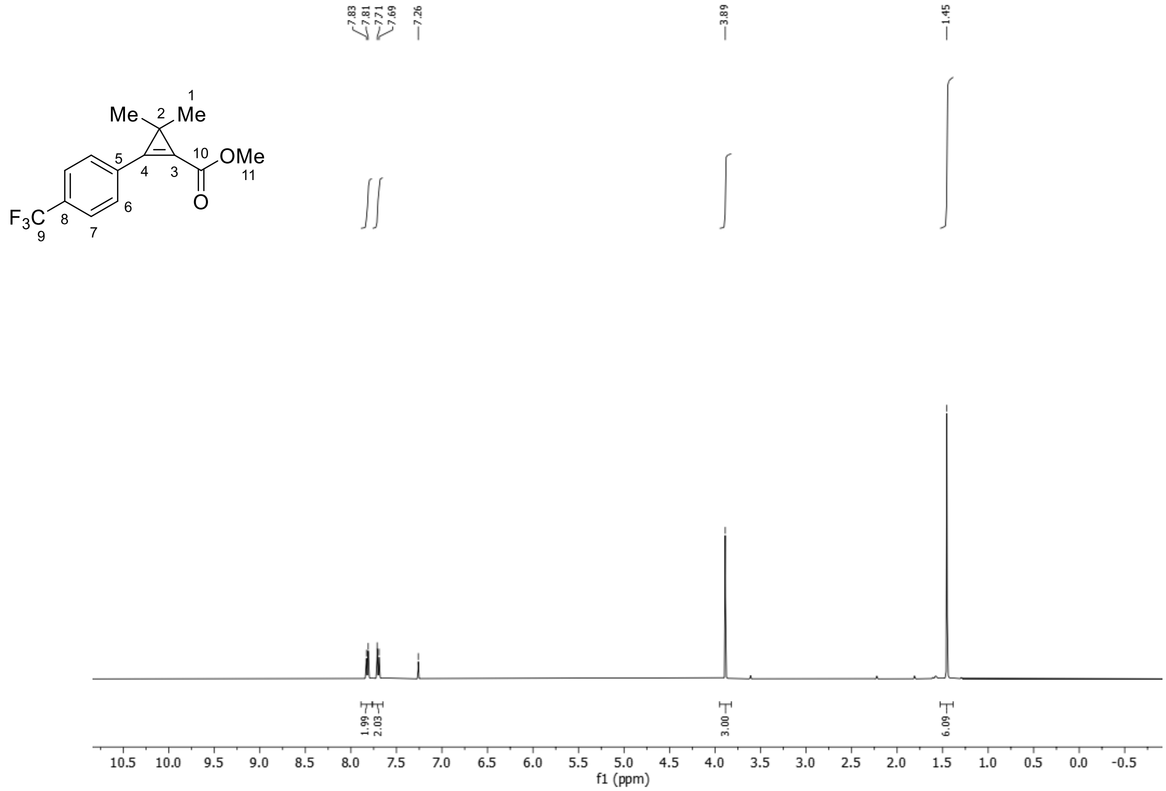

Figure S82. ^{1}H NMR of **S27** (400 MHz, 299 K, $CDCl_3$).

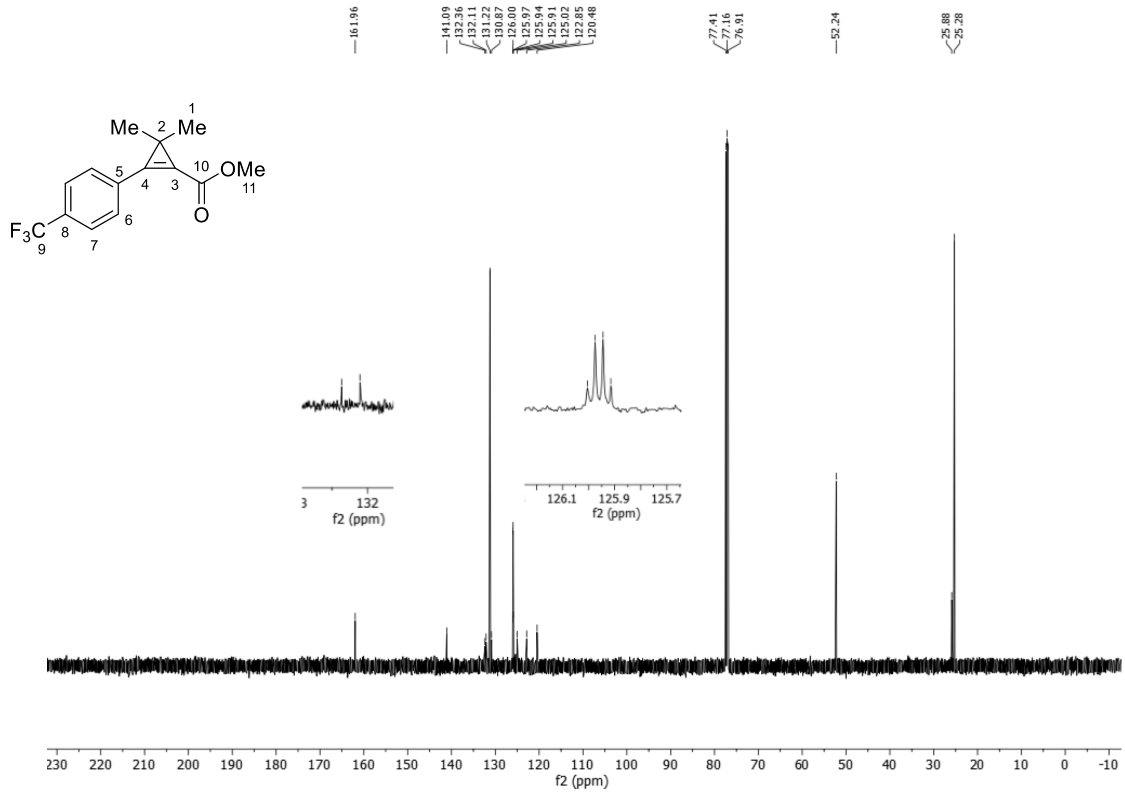

Figure S83. $^{13}C\{^{1}H\}$ NMR of **S27** (126 MHz, 299 K, $CDCl_3$).

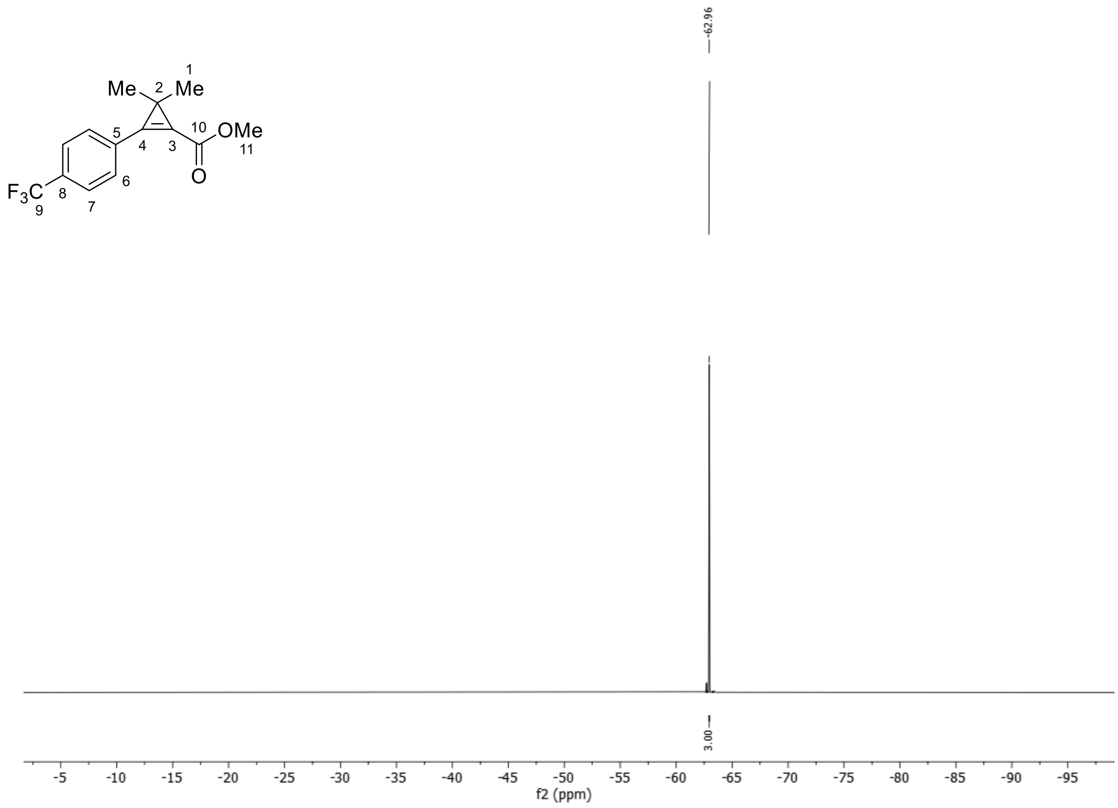

Figure S84. ^{19}F NMR of **S27** (470 MHz, 299 K, $CDCl_3$).

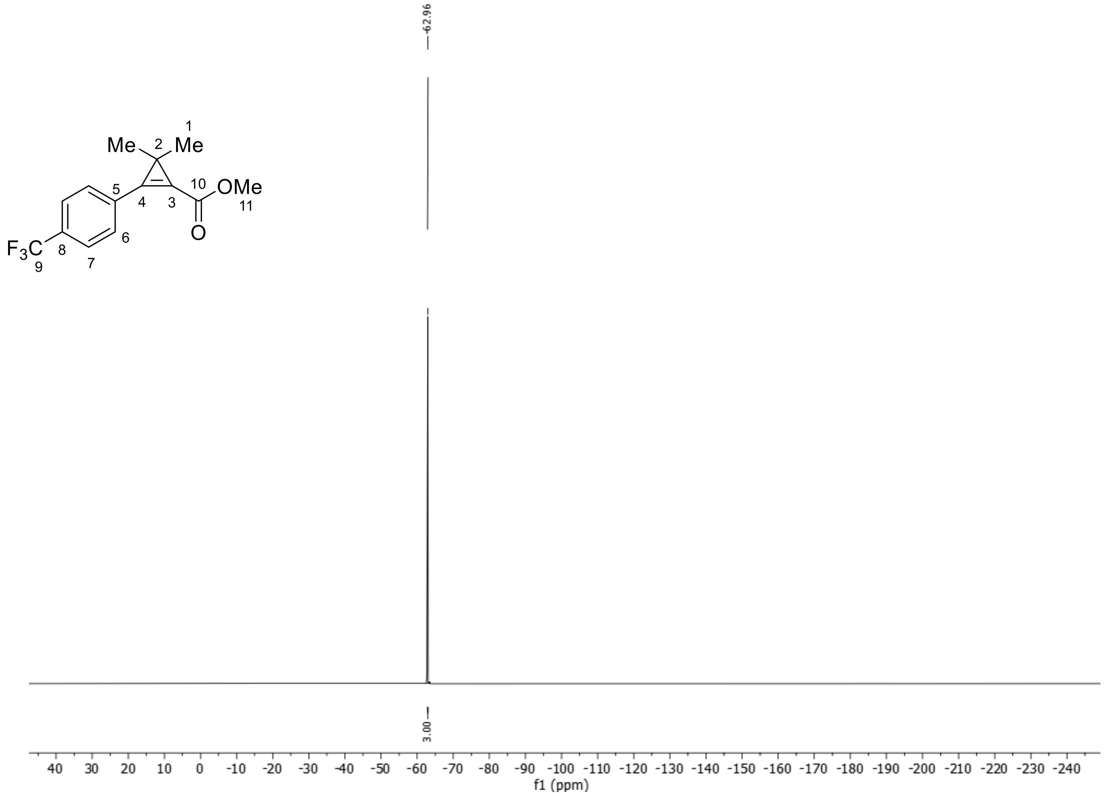

Figure S85. $^{19}F\{^1H\}$ NMR of **S27** (377 MHz, 299 K, $CDCl_3$).

N,N,3,3-tetramethyl-2-(4-(trifluoromethyl)phenyl)cycloprop-1-ene-1-carboxamide (S28)

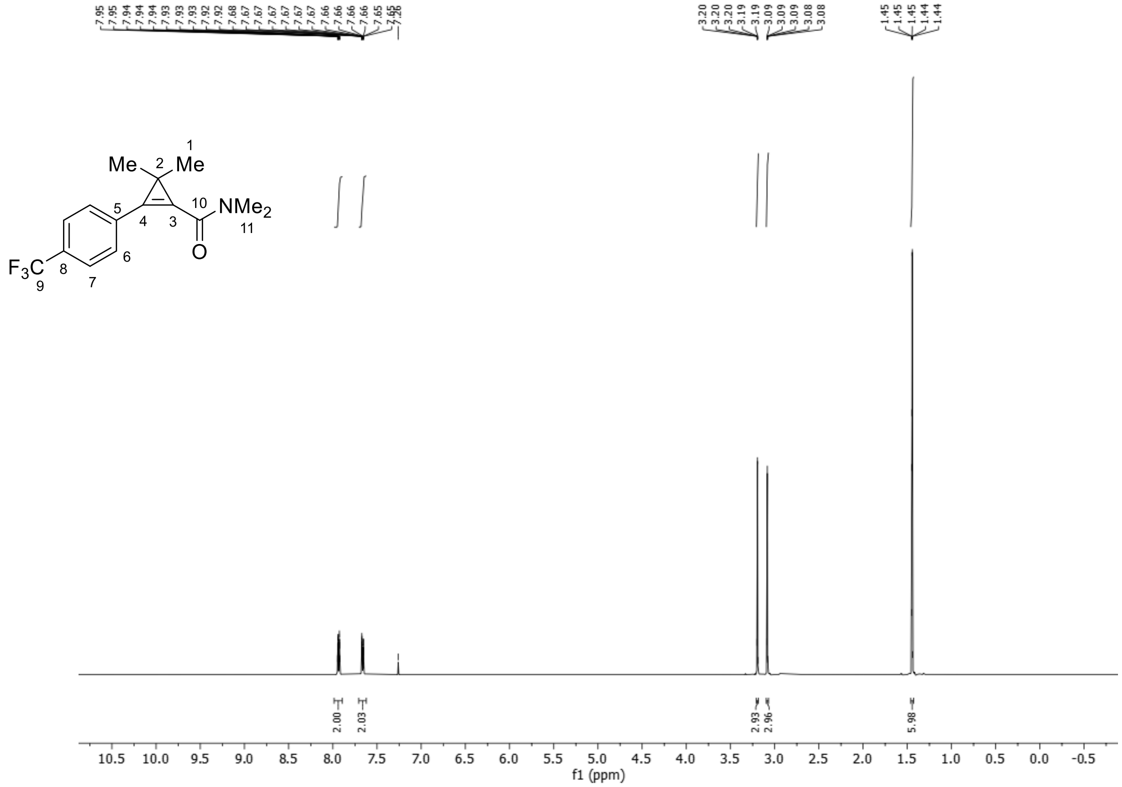

Figure S86. ^{1}H NMR of **S28** (500 MHz, 299 K, $CDCl_3$).

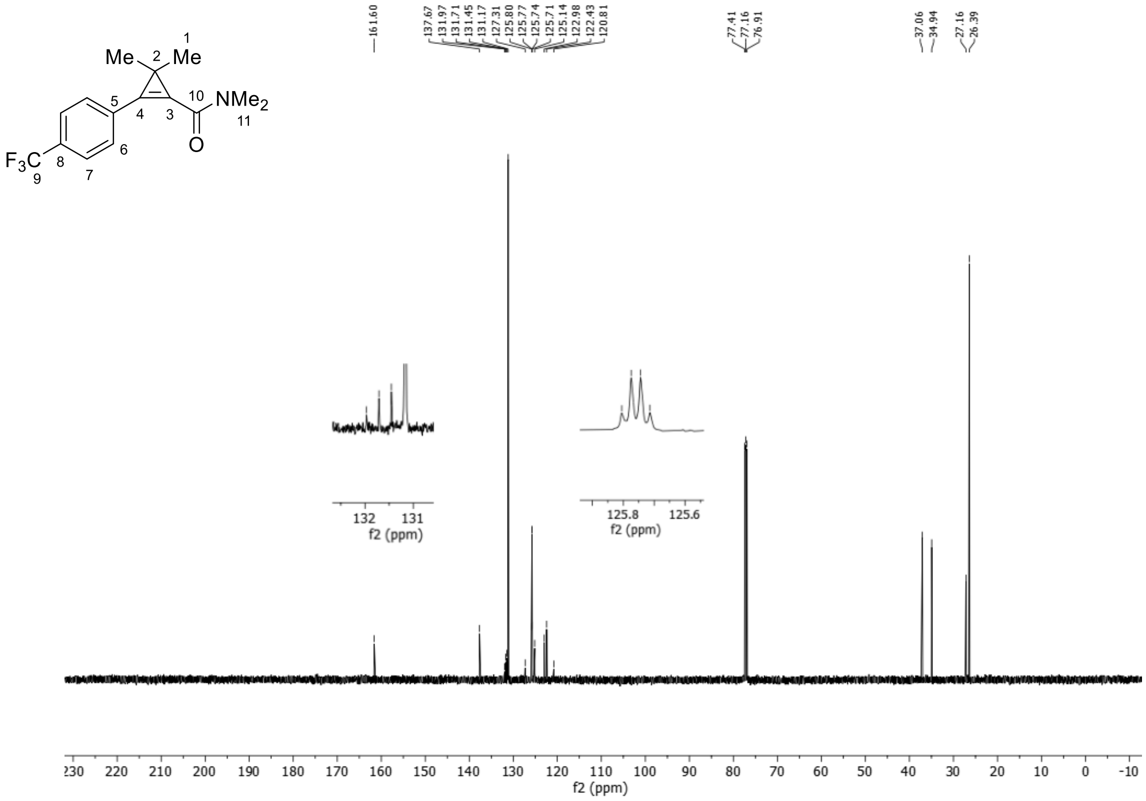

Figure S87. $^{13}C\{^{1}H\}$ NMR of **S28** (126 MHz, 299 K, $CDCl_3$).

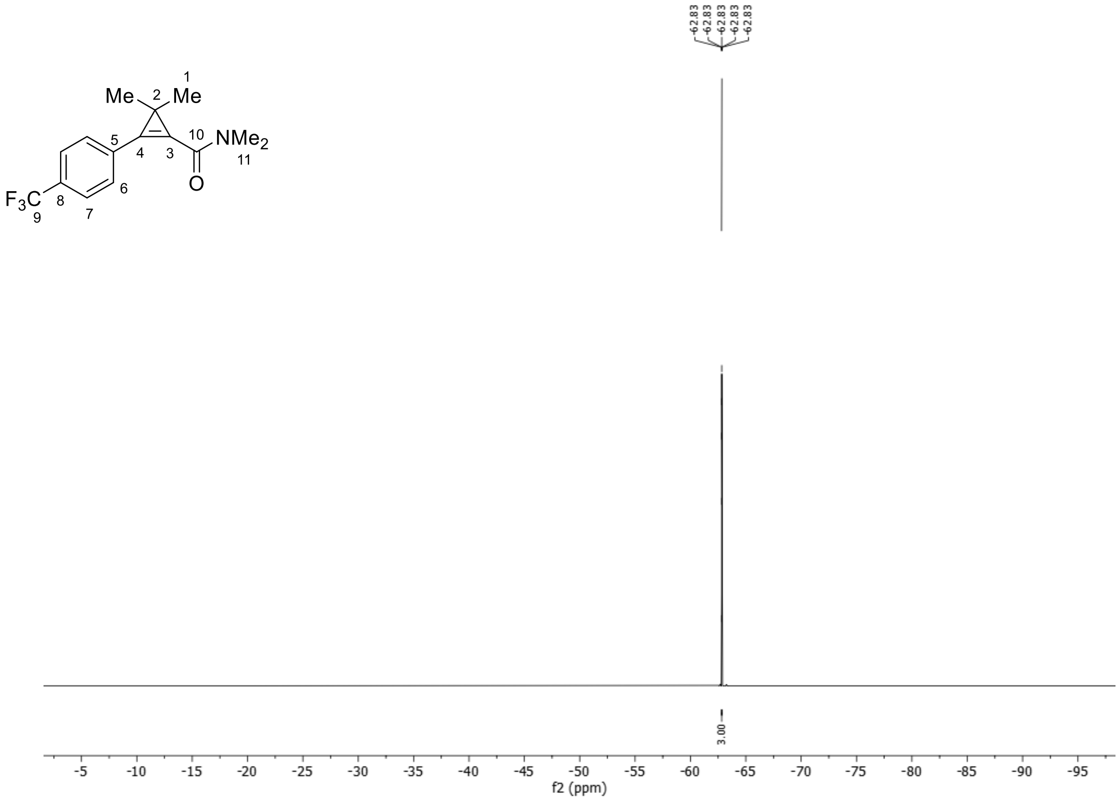

Figure S88. ^{19}F NMR of **S28** (470 MHz, 299 K, $CDCl_3$).

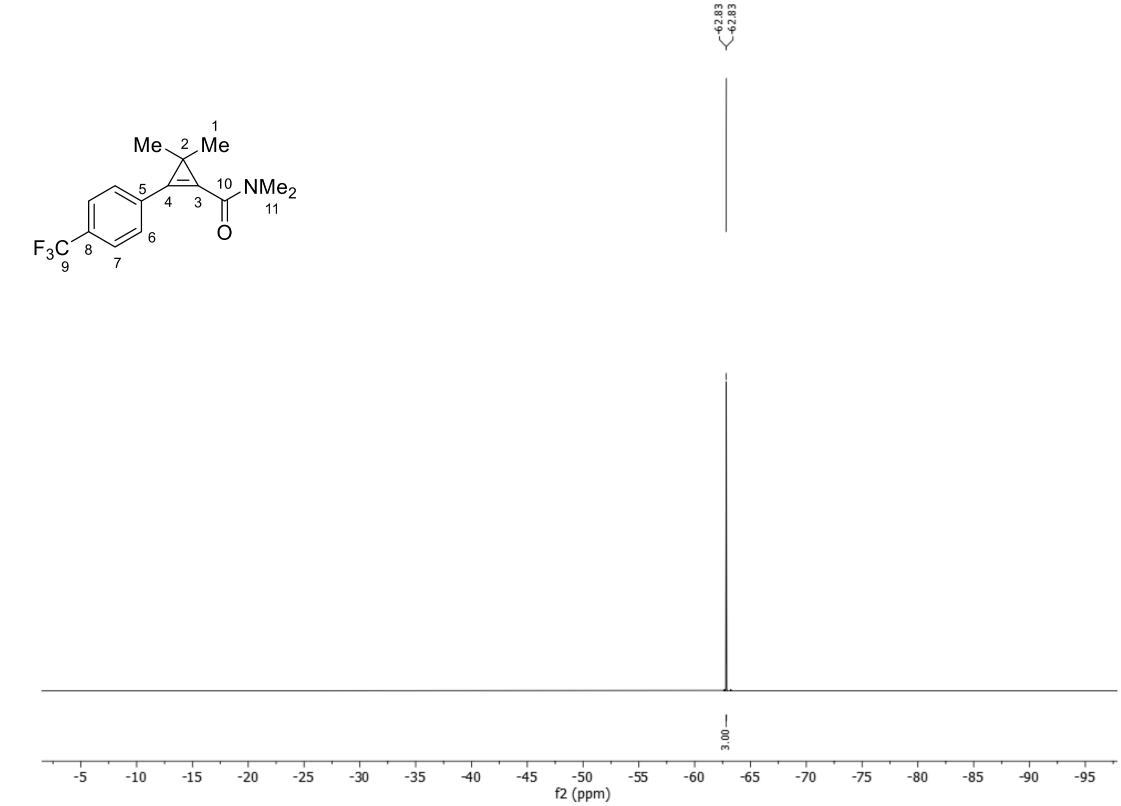

Figure S89. ^{19}F{^{1}H} NMR of **S28** (470 MHz, 299 K, $CDCl_3$).

1,1,2-Tribromo-2-(4-(trifluoromethyl)phenyl)spiro[2.5]octane (Int 16)

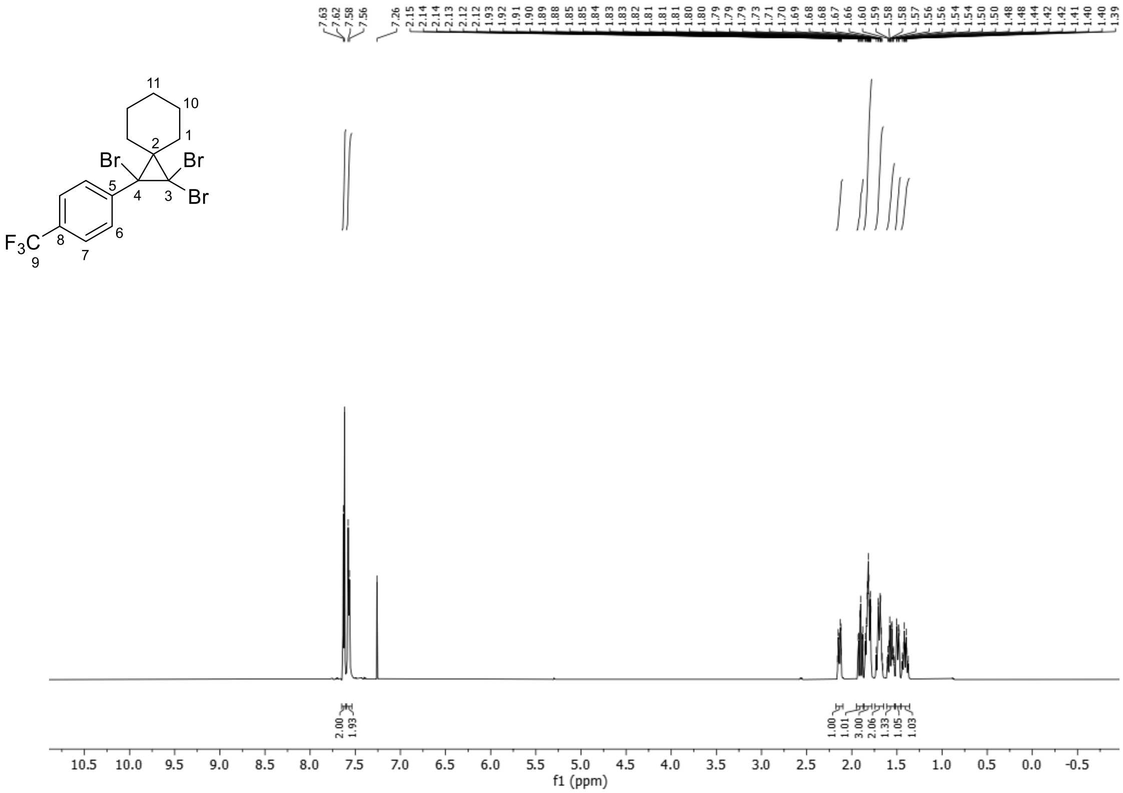

Figure S90. ^{1}H NMR of **Int 16** (599 MHz, 299 K, $CDCl_3$).

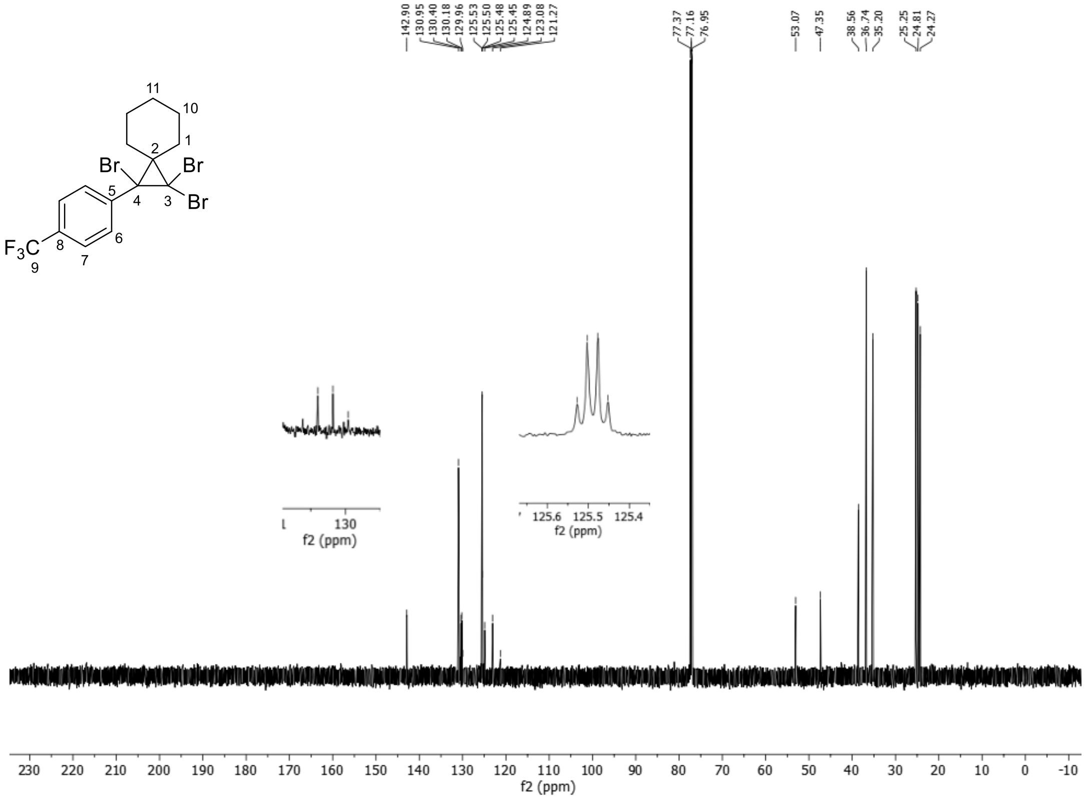

Figure S91. ^{13}C{^{1}H} NMR of **Int 16** (151 MHz, 299 K, $CDCl_3$).

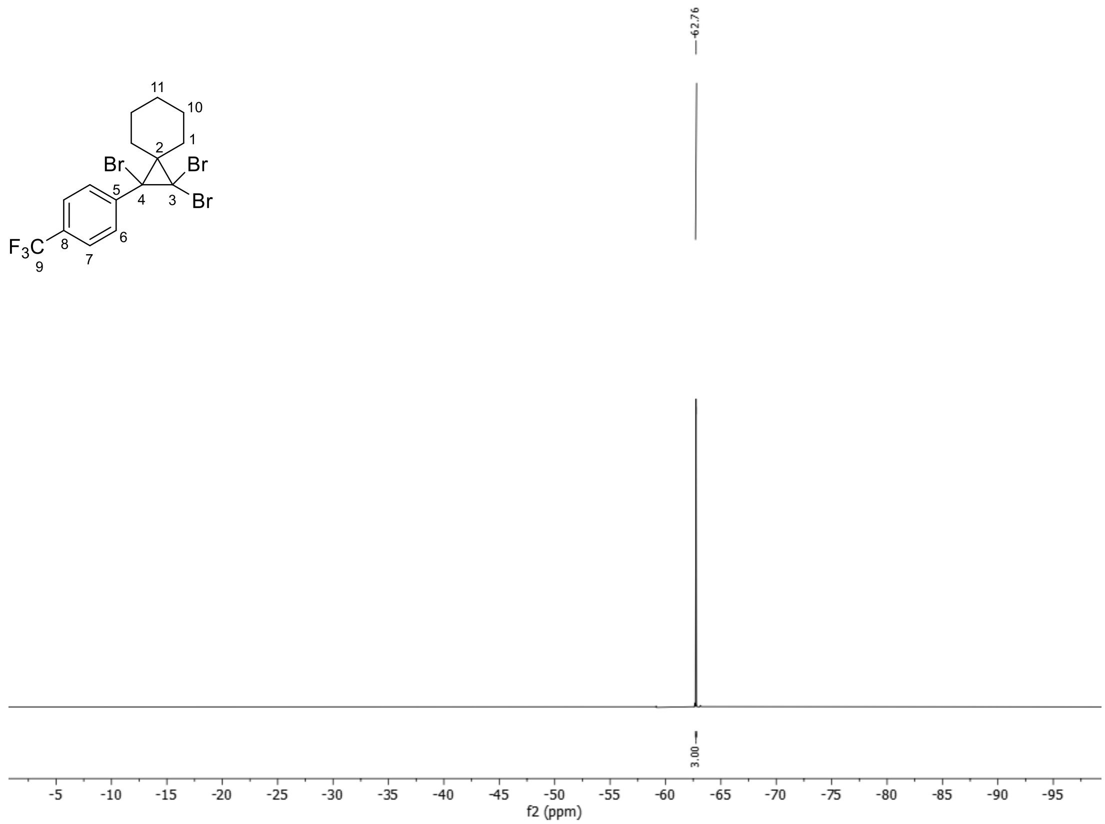

Figure S92. ^{19}F NMR of **Int 16** (564 MHz, 299 K, $CDCl_3$).

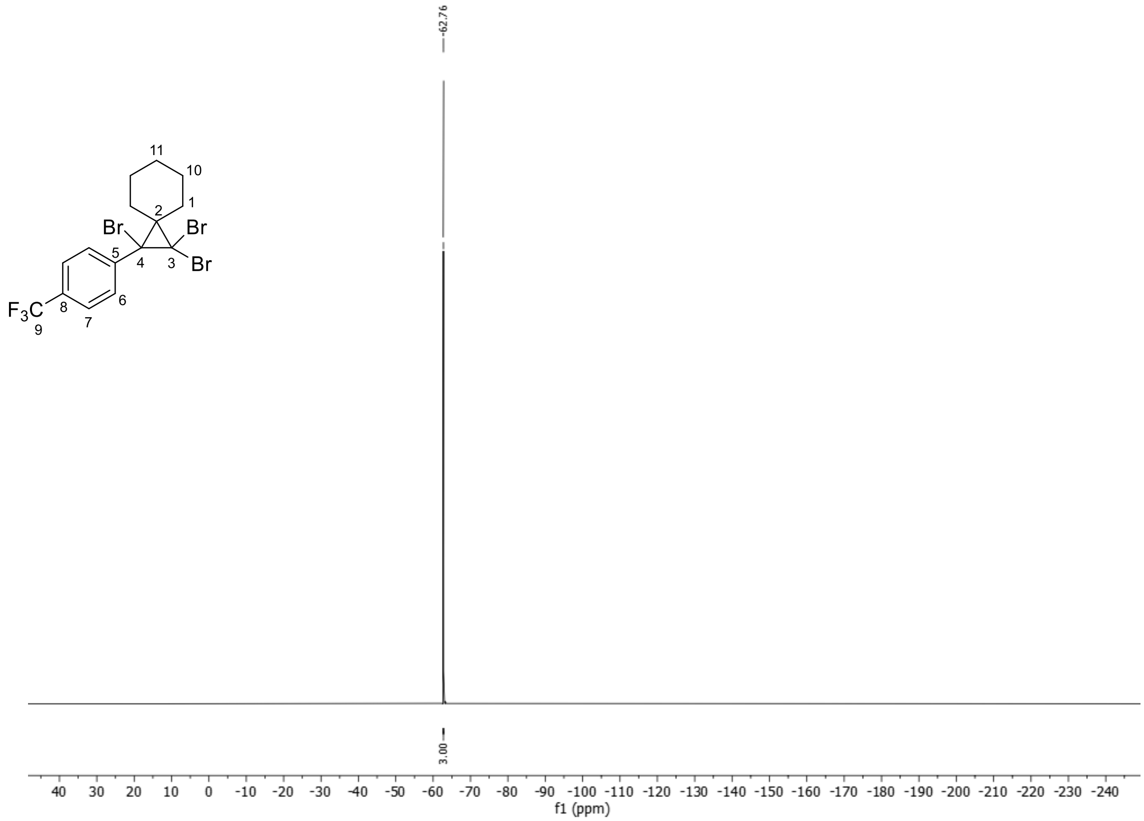

Figure S93. $^{19}F\{^{1}H\}$ NMR of **Int 16** (377 MHz, 299 K, $CDCl_3$).

Methyl 2-(4-(trifluoromethyl)phenyl)spiro[2.5]oct-1-ene-1-carboxylate (S29)

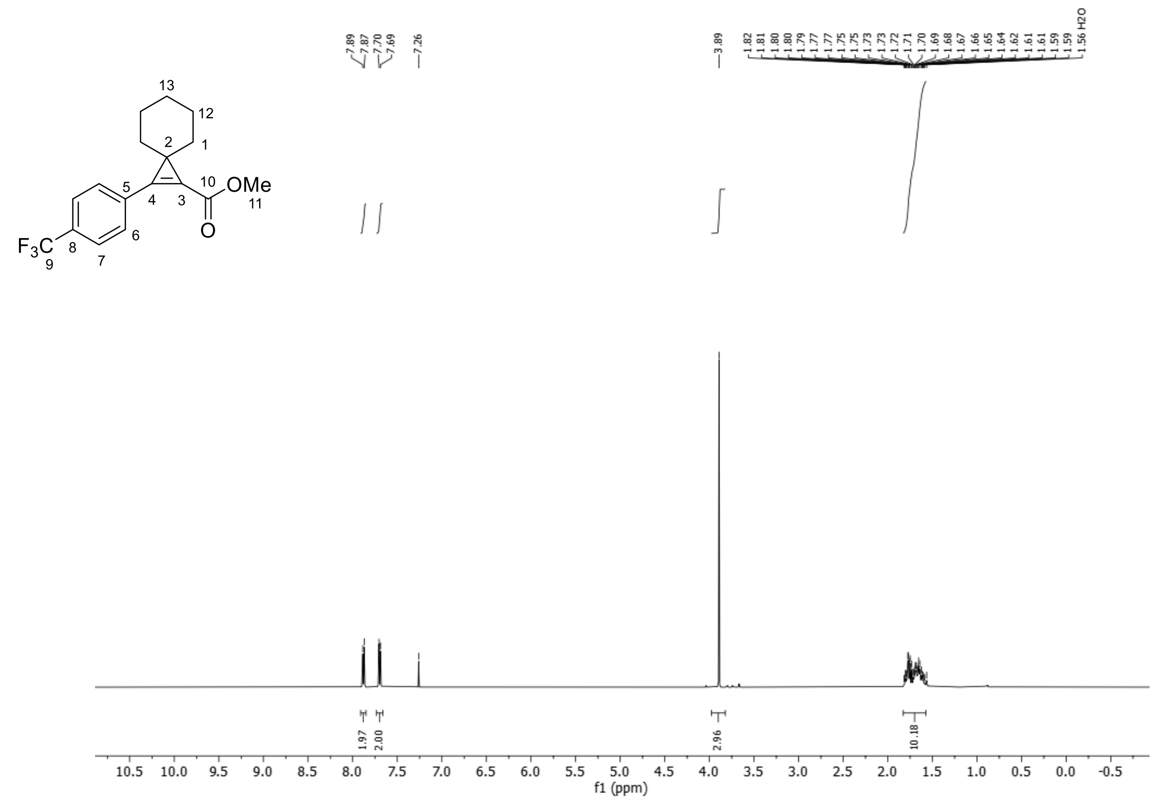

Figure S94. ^{1}H NMR of **S29** (500 MHz, 299 K, $CDCl_3$).

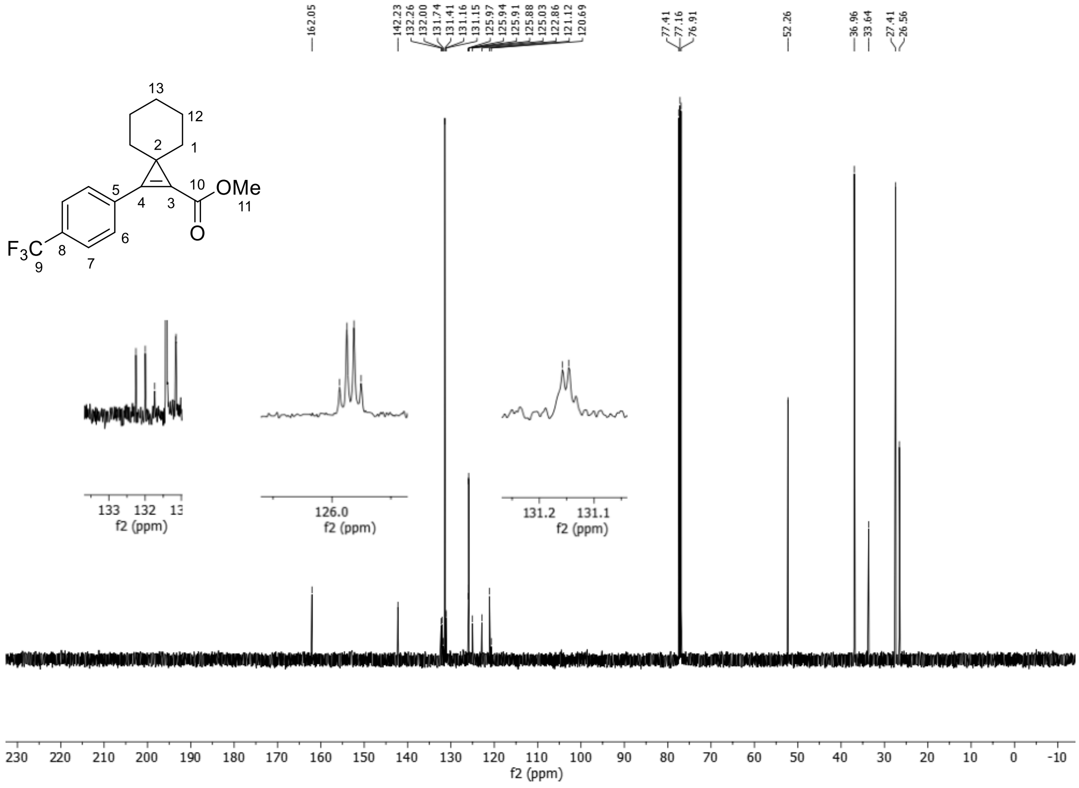

Figure S95. $^{13}C\{^{1}H\}$ NMR of **S29** (126 MHz, 299 K, $CDCl_3$).

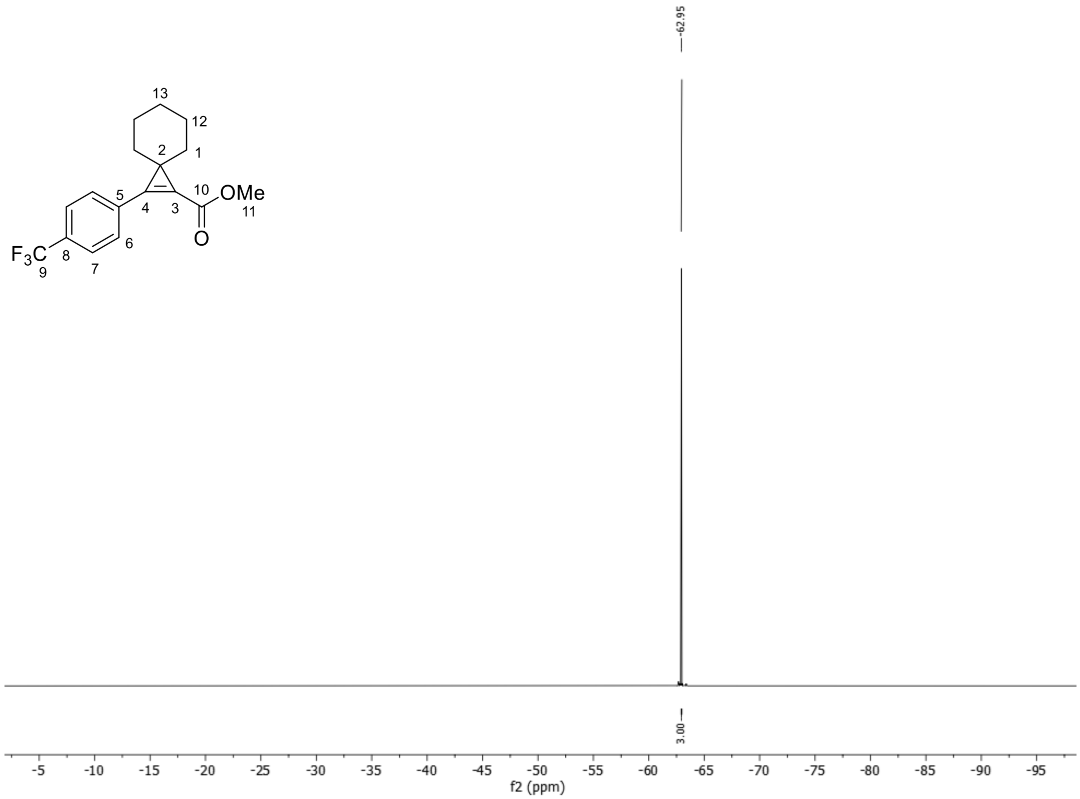

Figure S96. ^{19}F NMR of **S29** (470 MHz, 299 K, $CDCl_3$).

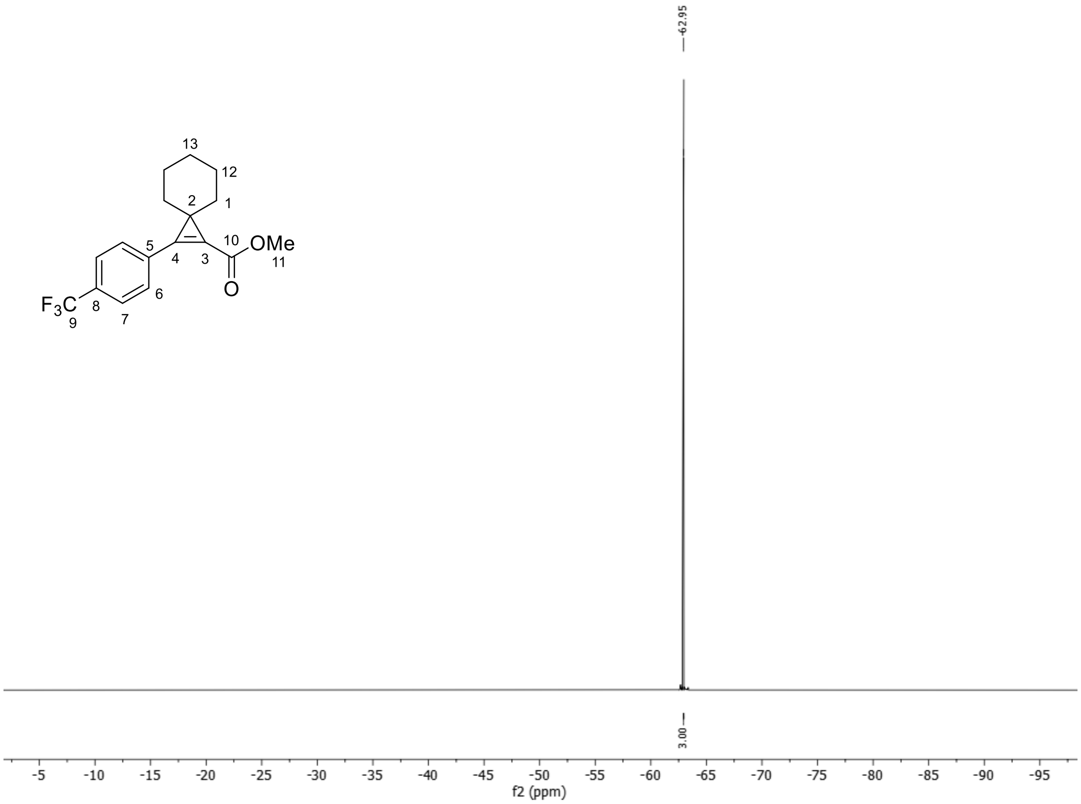

Figure S97. ^{19}F{^{1}H} NMR of **S29** (470 MHz, 299 K, $CDCl_3$).

(*E*)-1-(1,1,4,4,4-Pentafluoro-3-phenylbut-2-en-1-yl)-4-(trifluoromethyl)benzene (1)

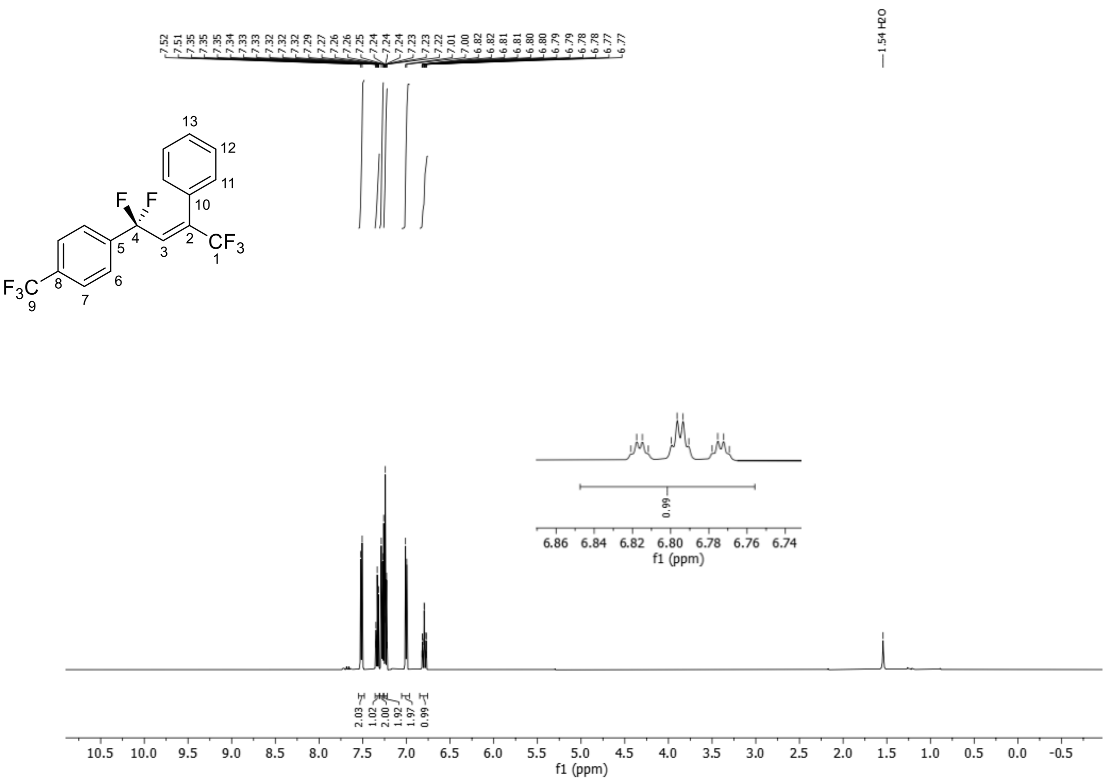

Figure S98. ^{1}H NMR of **1** (500 MHz, 299 K, $CDCl_3$).

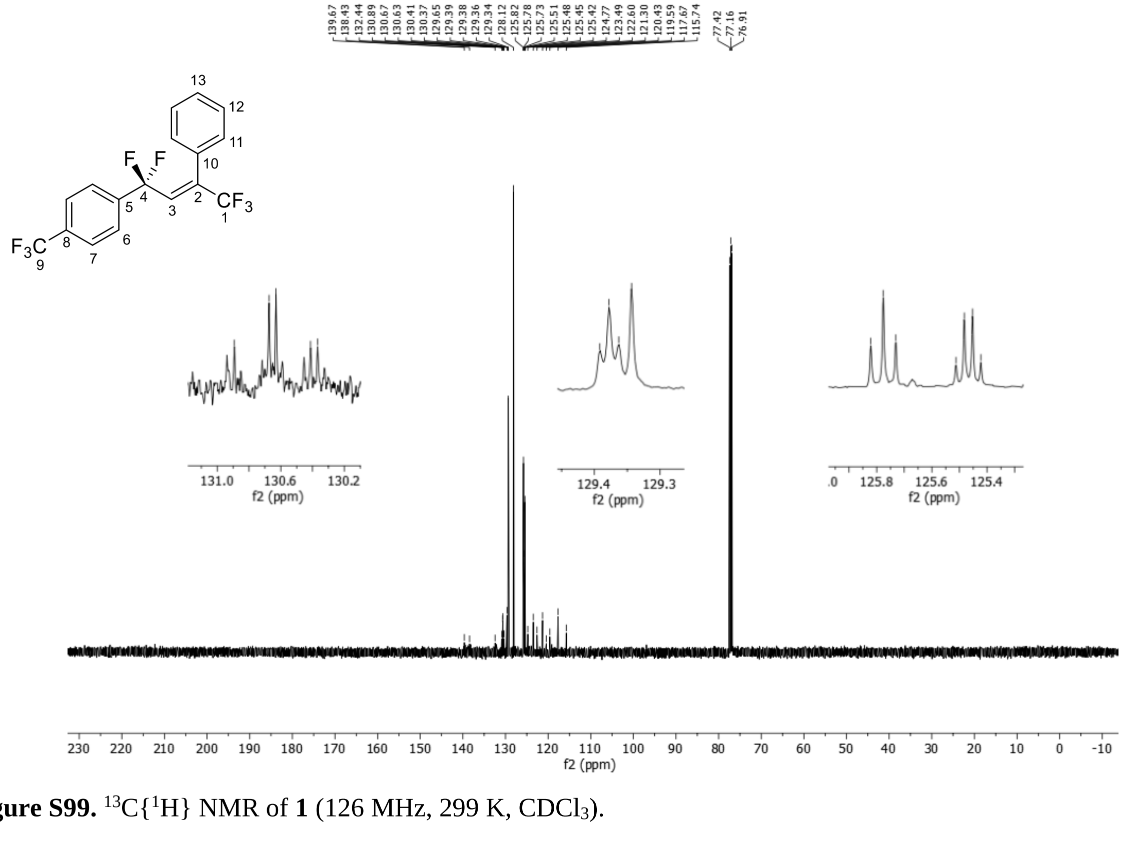

Figure S99. $^{13}C\{^{1}H\}$ NMR of **1** (126 MHz, 299 K, $CDCl_3$).

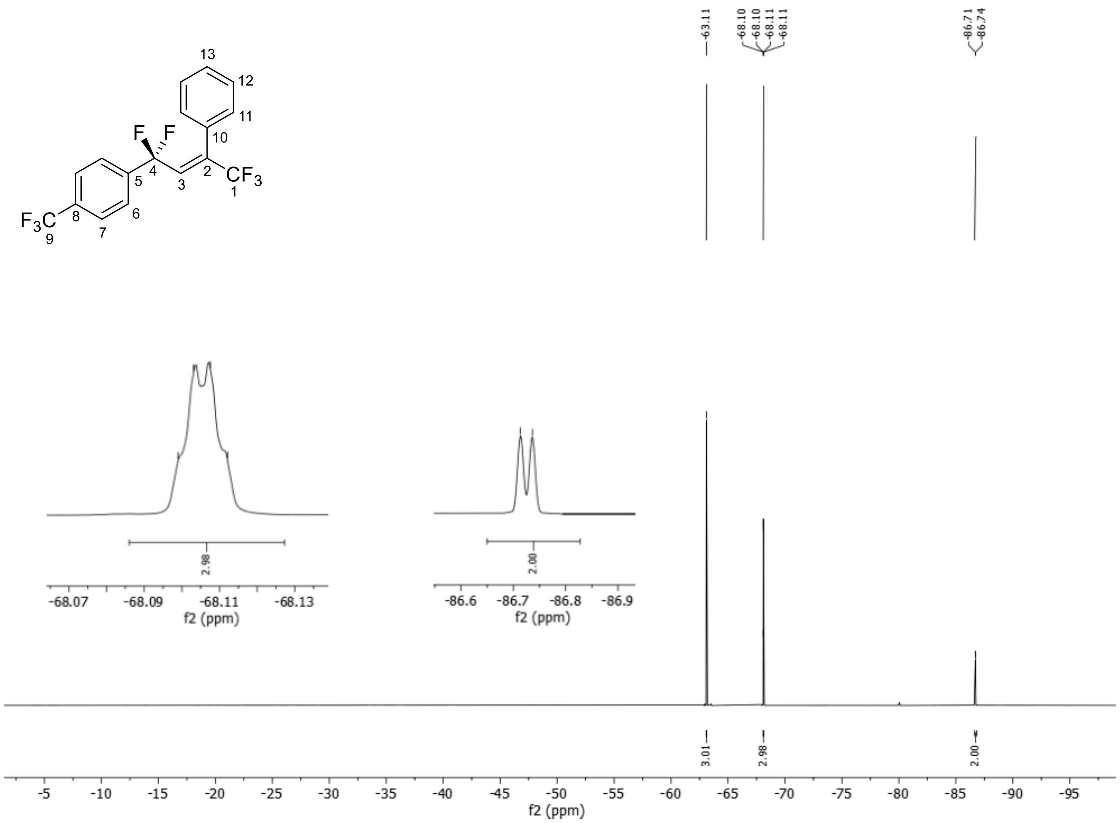

Figure S100. ^{19}F NMR of **1** (470 MHz, 299 K, $CDCl_3$).

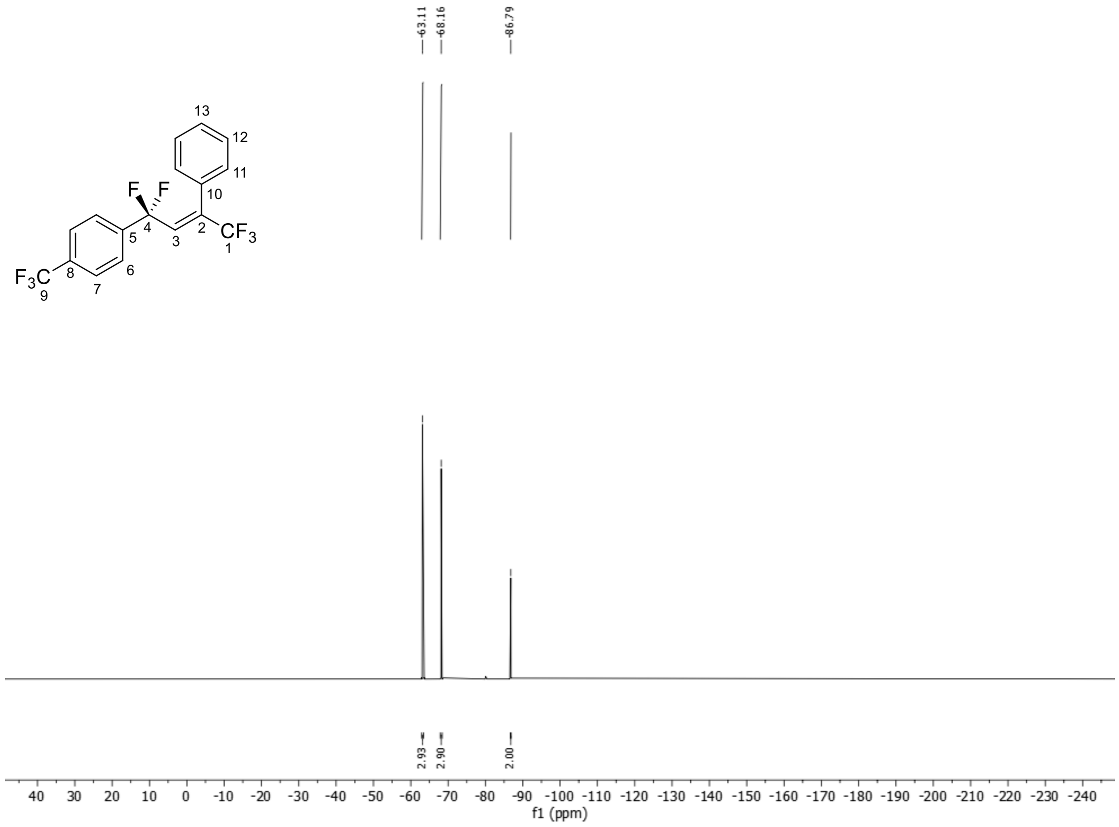

Figure S101. $^{19}F\{^{1}H\}$ NMR of **1** (377 MHz, 299 K, $CDCl_3$).

(*E*)-1-Bromo-4-(1,1,1,4,4-Pentafluoro-4-(4-(trifluoromethyl)phenyl)but-2-en-2-yl)benzene (2)

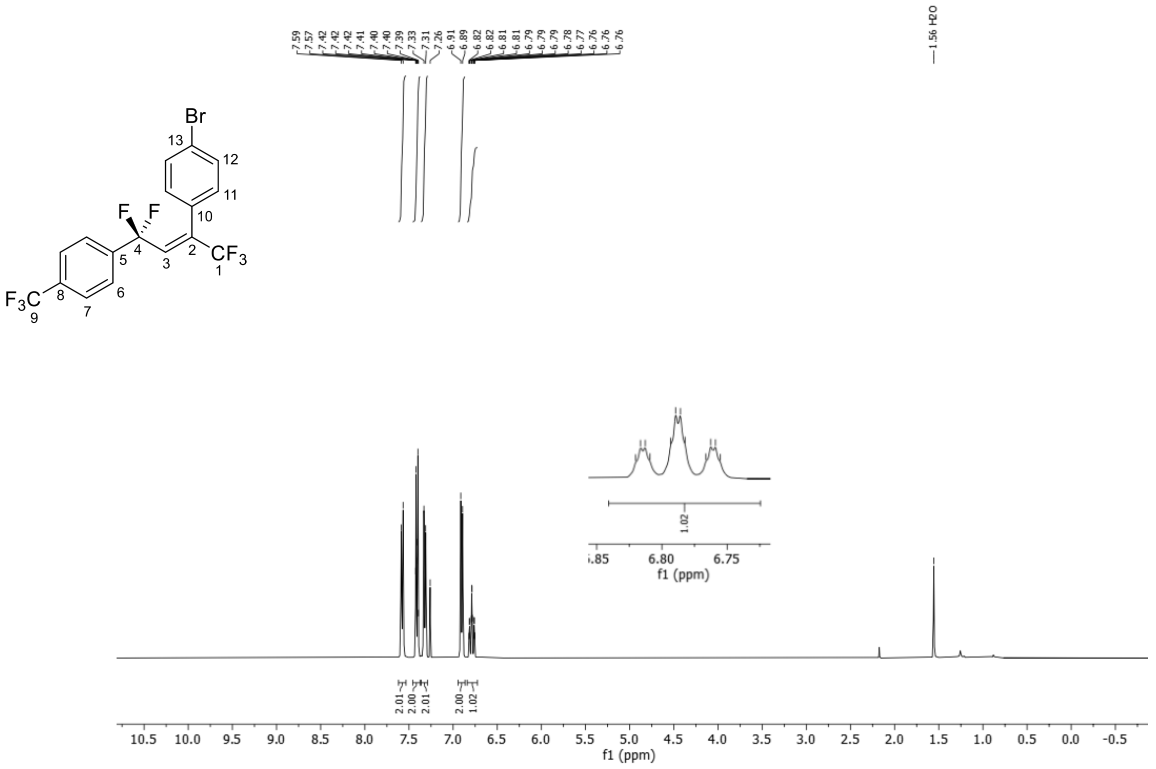

Figure S102. ^{1}H NMR of **2** (400 MHz, 299 K, $CDCl_3$).

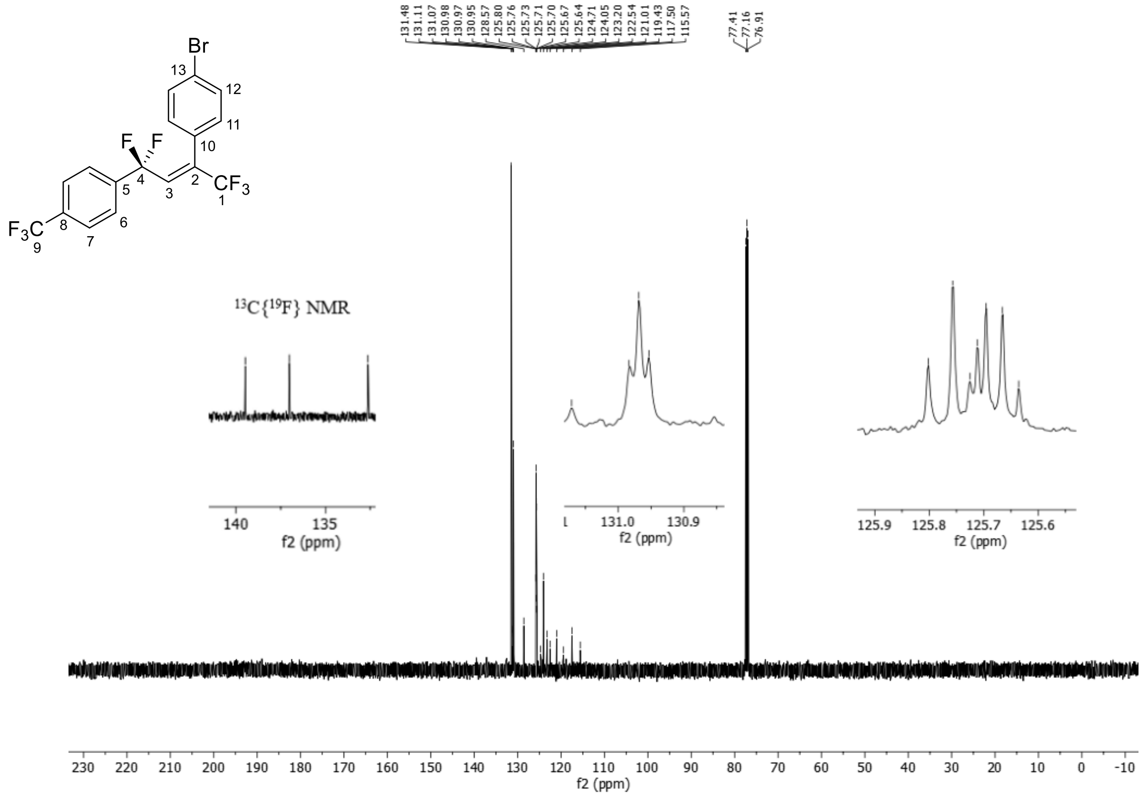

Figure S103. $^{13}C\{^{1}H\}$ NMR of **2** (126 MHz, 299 K, $CDCl_3$).

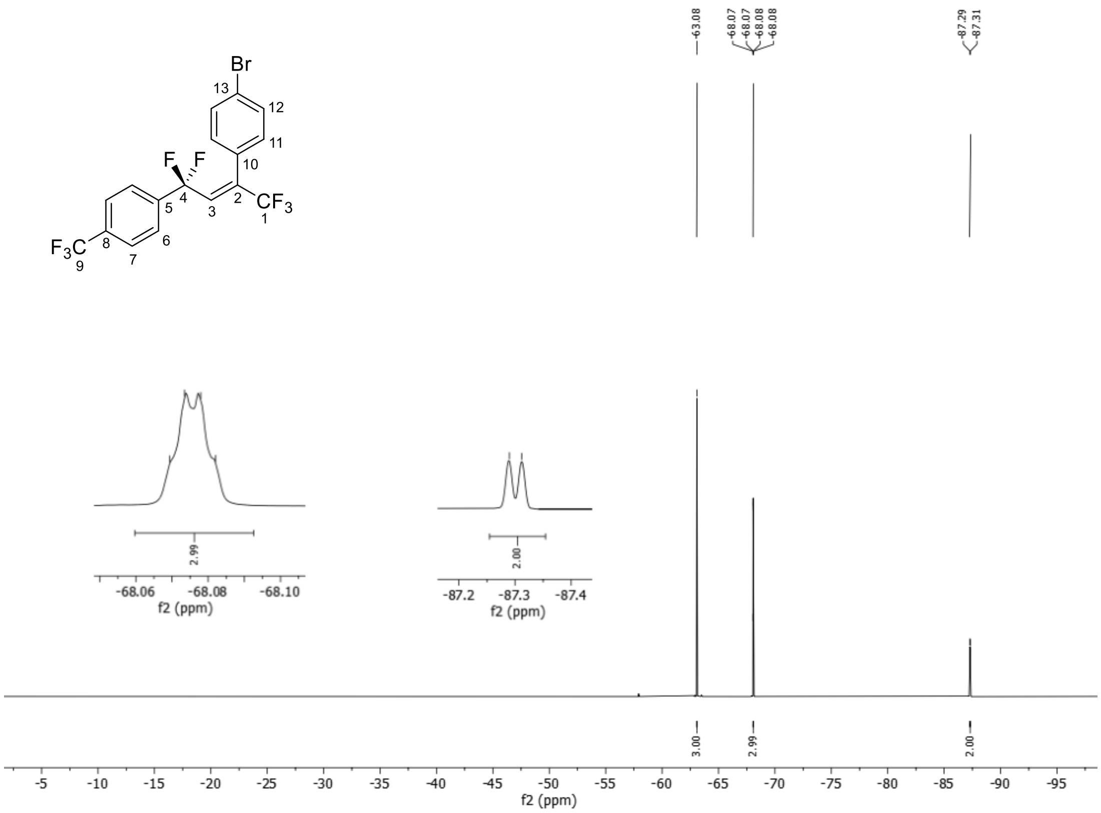

Figure S104. ^{19}F NMR of **2** (470 MHz, 299 K, $CDCl_3$).

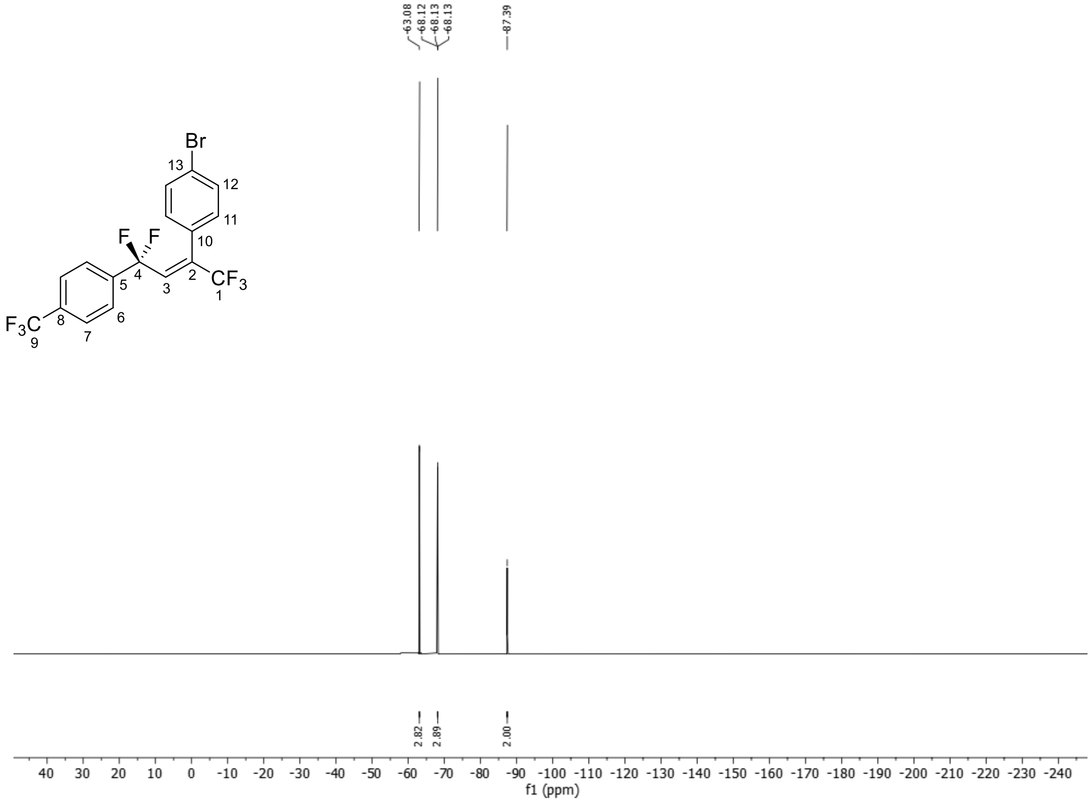

Figure S105. $^{19}F\{^1H\}$ NMR of **2** (377 MHz, 299 K, $CDCl_3$).

(*E*)-1-Chloro-4-(1,1,1,4,4-pentafluoro-4-(4-(trifluoromethyl)phenyl)but-2-en-2-yl)benzene (3)

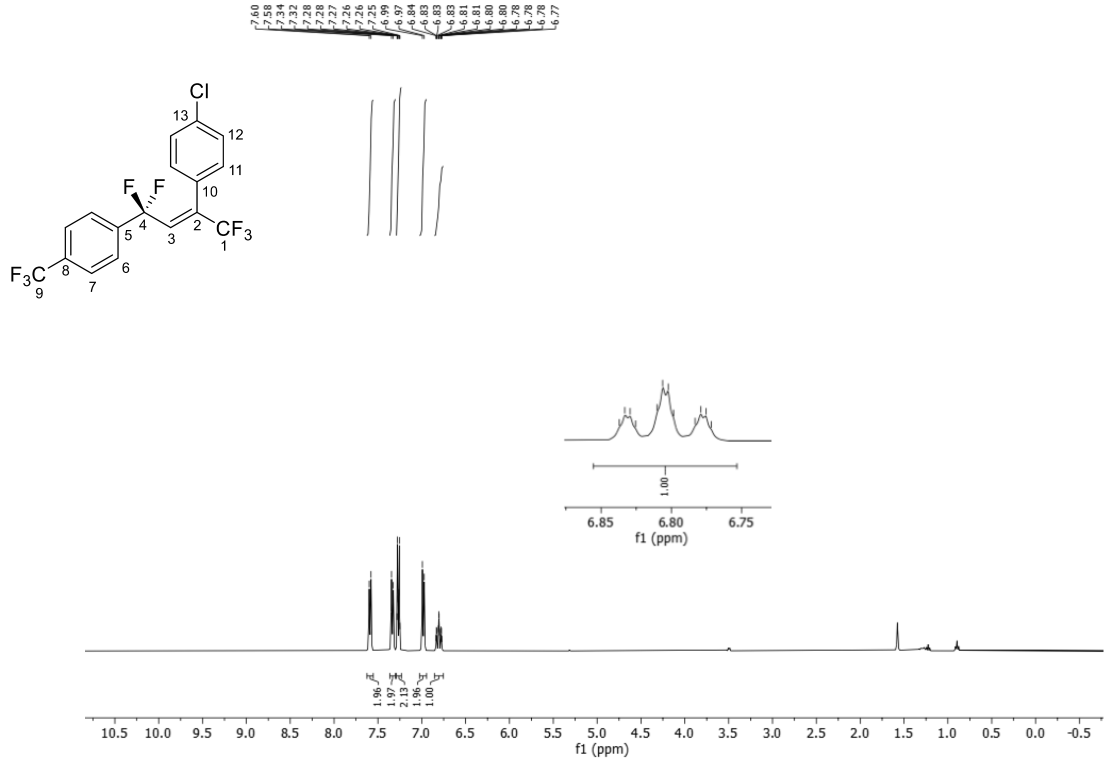

Figure S106. ^{1}H NMR of **3** (400 MHz, 299 K, $CDCl_3$).

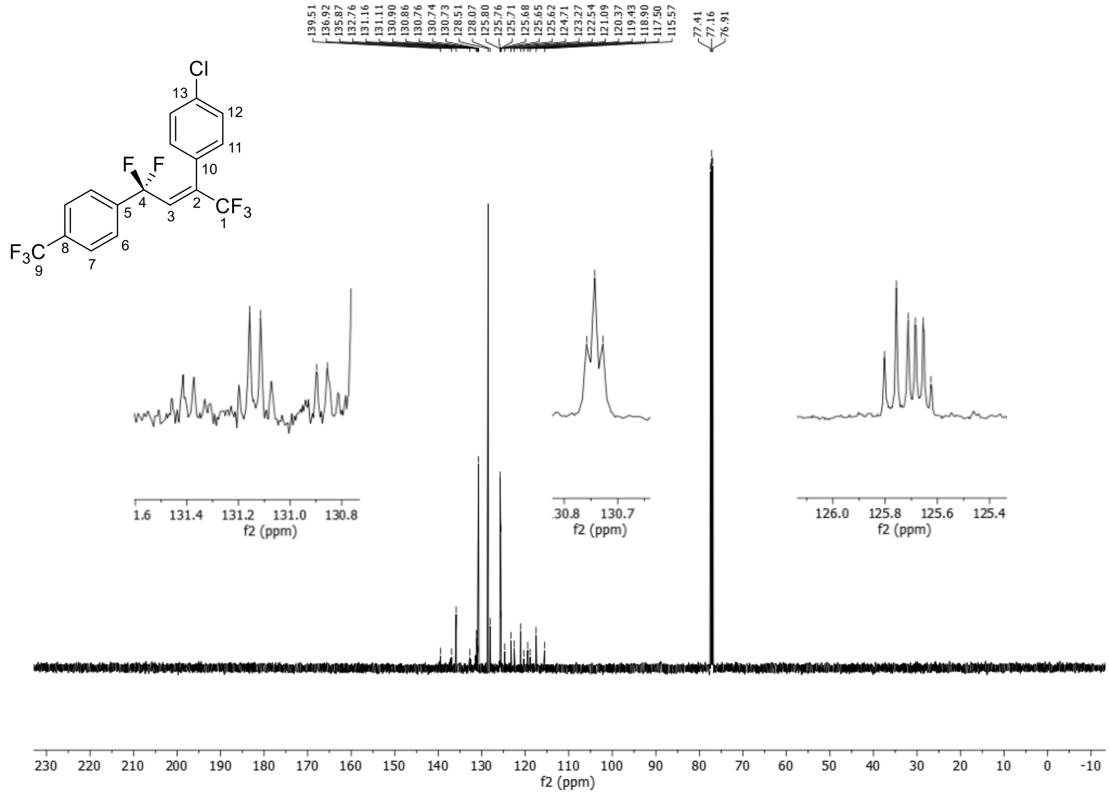

Figure S107. $^{13}C\{^{1}H\}$ NMR of **3** (126 MHz, 299 K, $CDCl_3$).

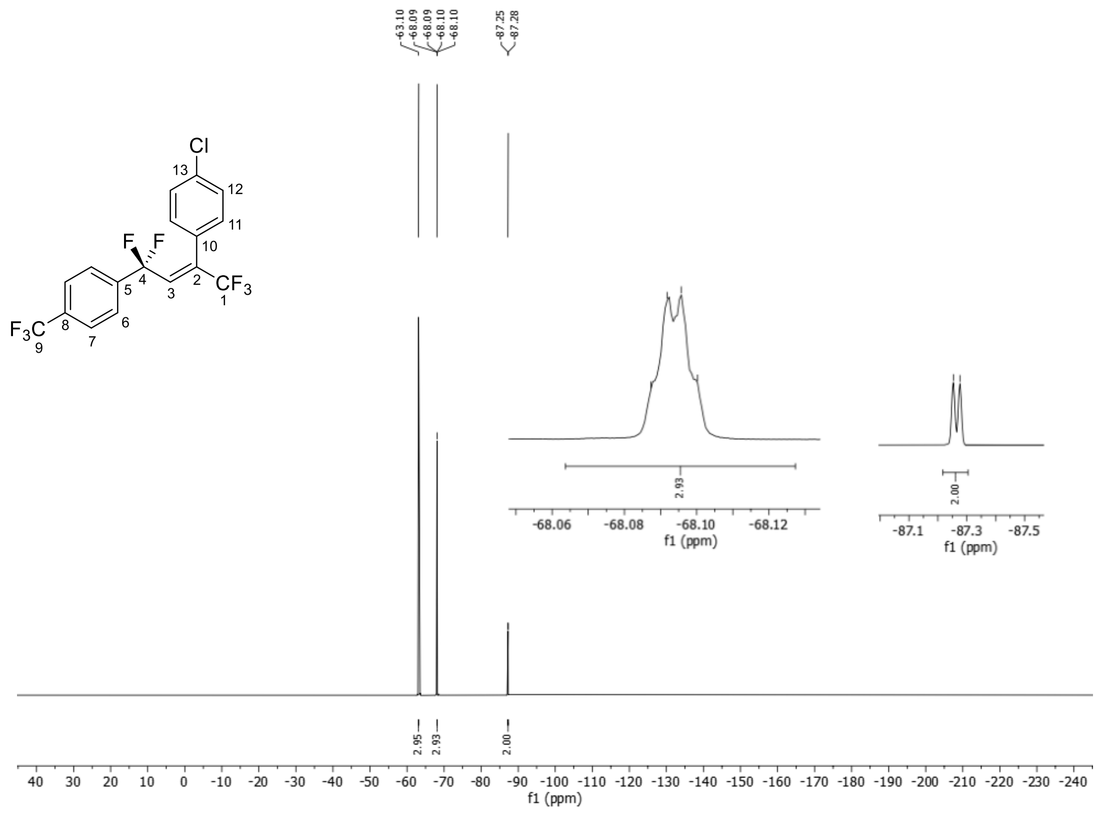

Figure S108. ^{19}F NMR of **3** (470 MHz, 299 K, $CDCl_3$).

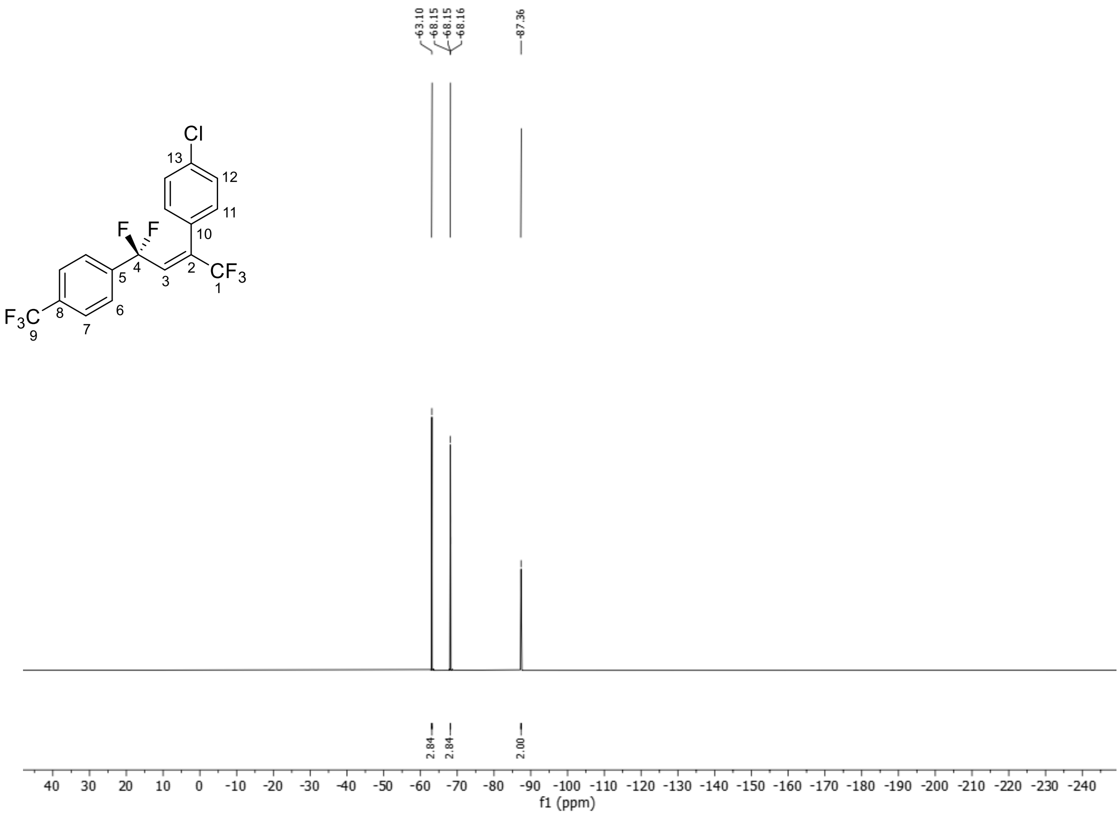

Figure S109. $^{19}F\{^1H\}$ NMR of **3** (377 MHz, 299 K, $CDCl_3$).

(*E*)-4,4'-(1,1,4,4,4-Pentafluorobut-2-ene-1,3-diyl)bis((trifluoromethyl)benzene) (4)

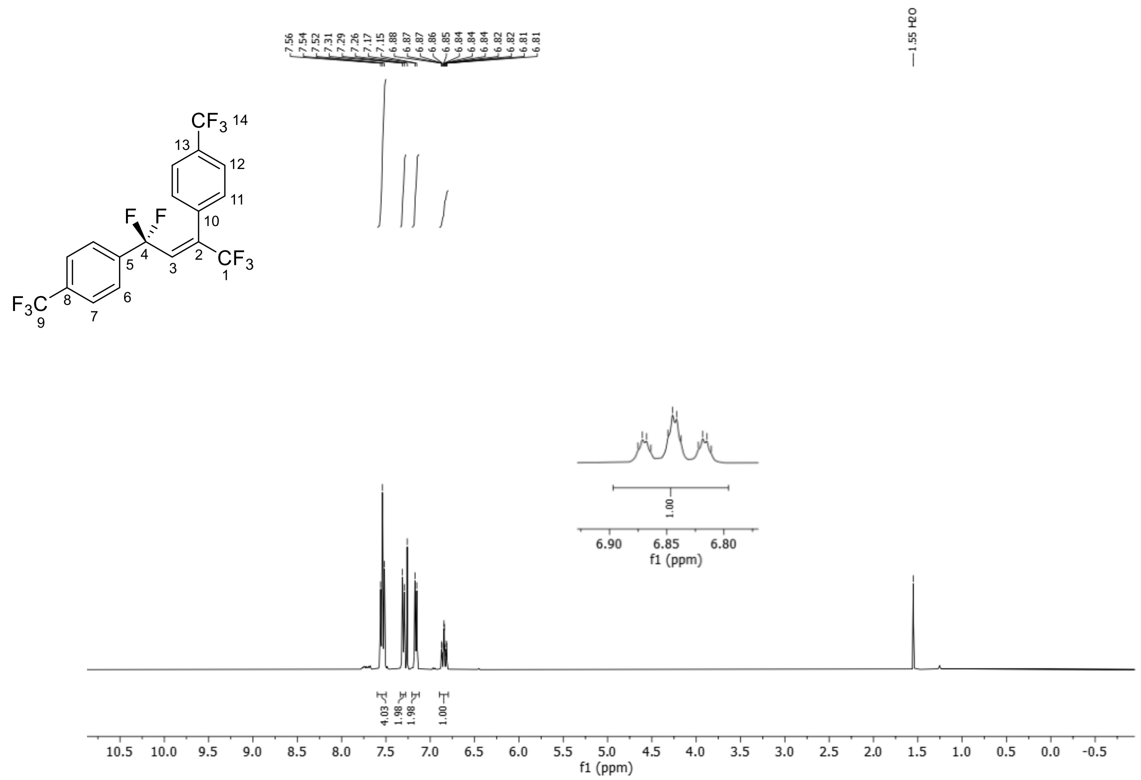

Figure S110. ^{1}H NMR of **4** (400 MHz, 299 K, $CDCl_3$).

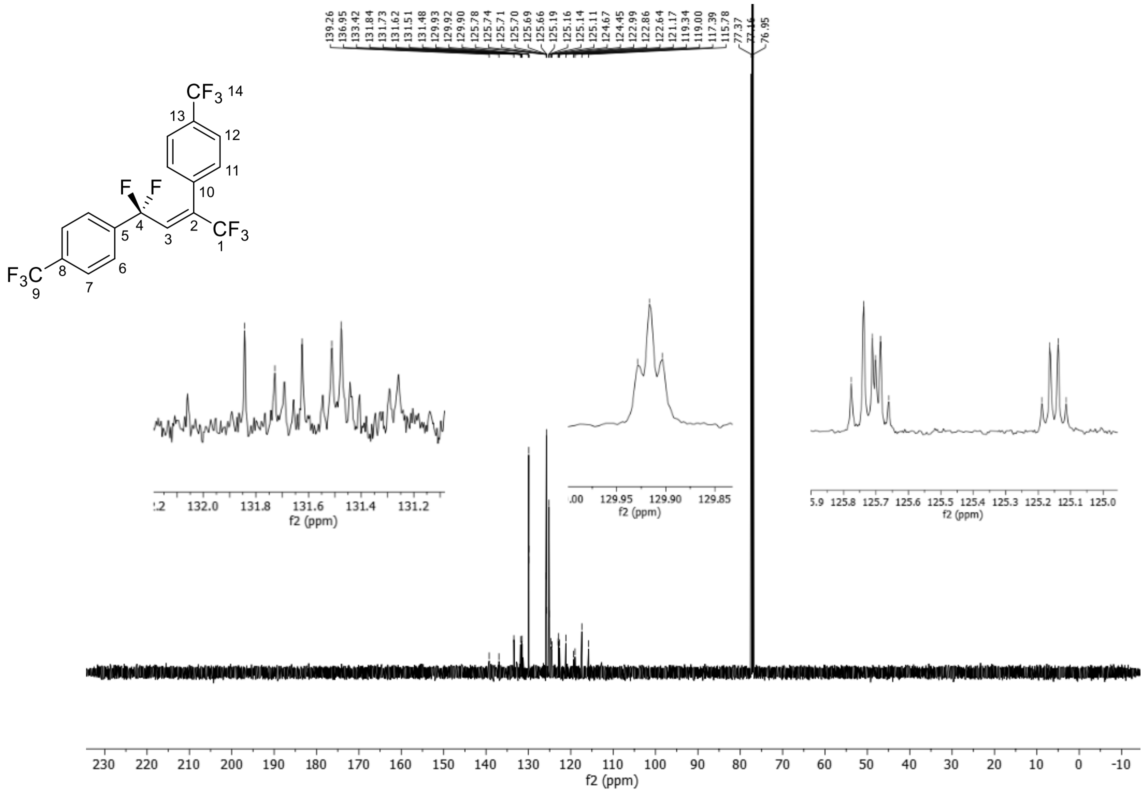

Figure S111. ^{13}C{^{1}H} NMR of **4** (151 MHz, 299 K, $CDCl_3$).

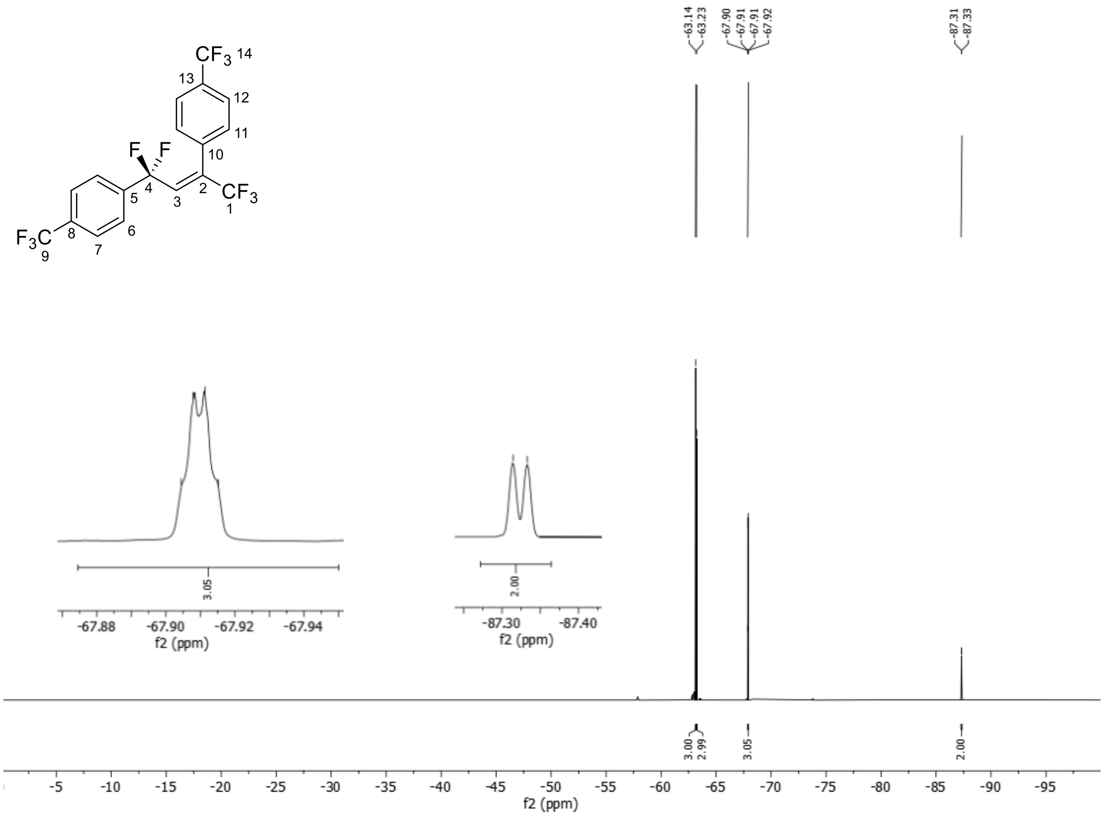

Figure S112. ^{19}F NMR of **4** (564 MHz, 299 K, $CDCl_3$).

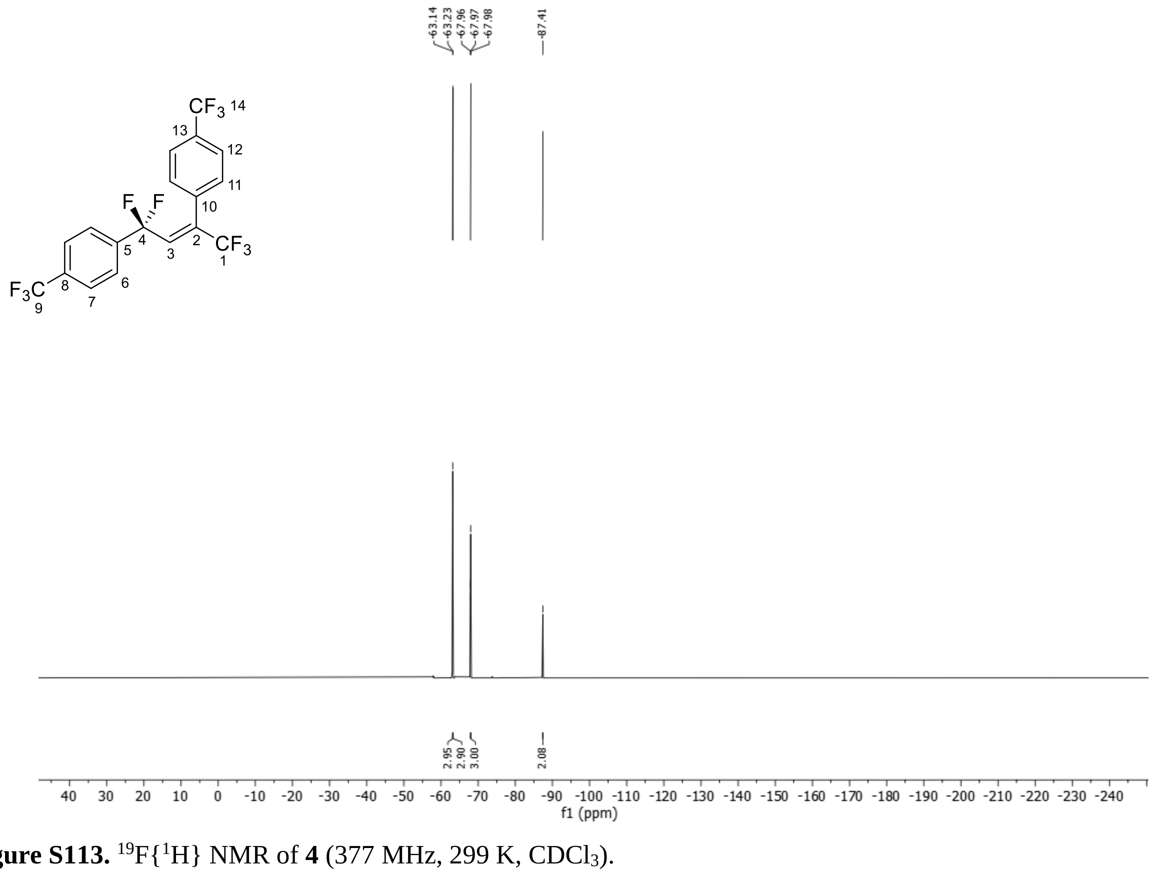

Figure S113. $^{19}F\{^1H\}$ NMR of **4** (377 MHz, 299 K, $CDCl_3$).

(*E*)-1-Methyl-4-(1,1,1,4,4-pentafluoro-4-(4-(trifluoromethyl)phenyl)but-2-en-2-yl)benzene (5)

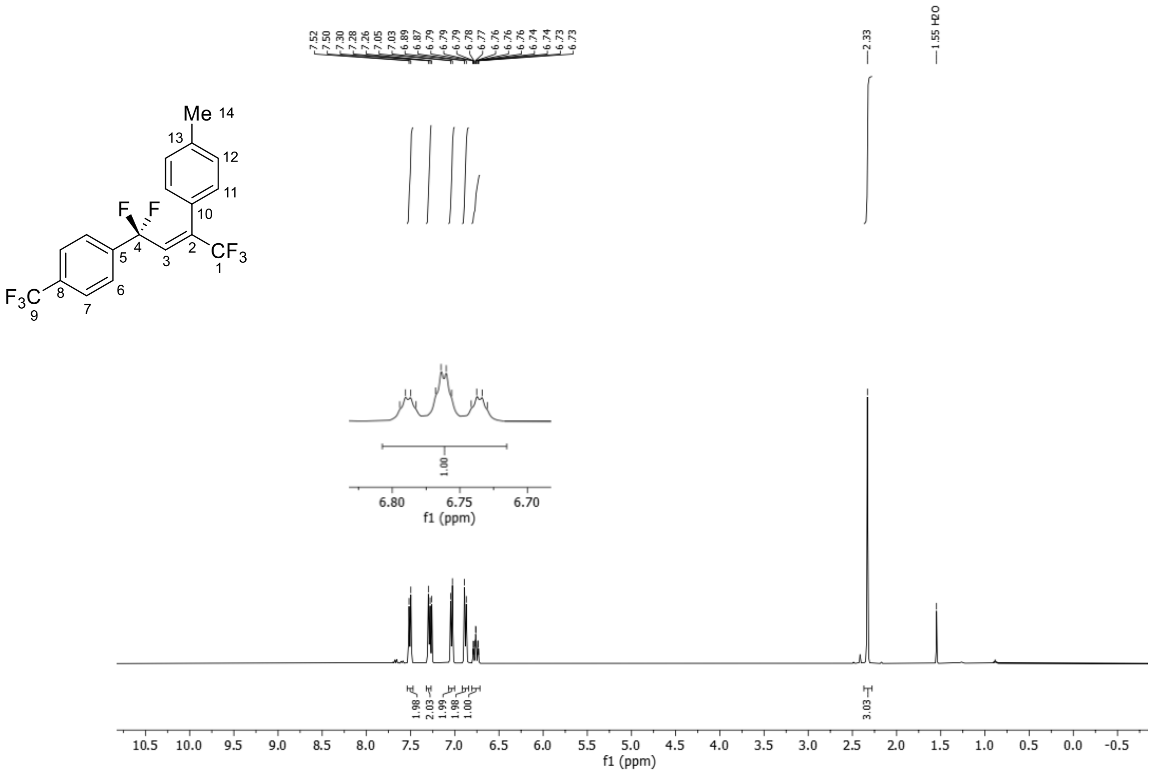

Figure S114. ^{1}H NMR of **5** (400 MHz, 299 K, $CDCl_3$).

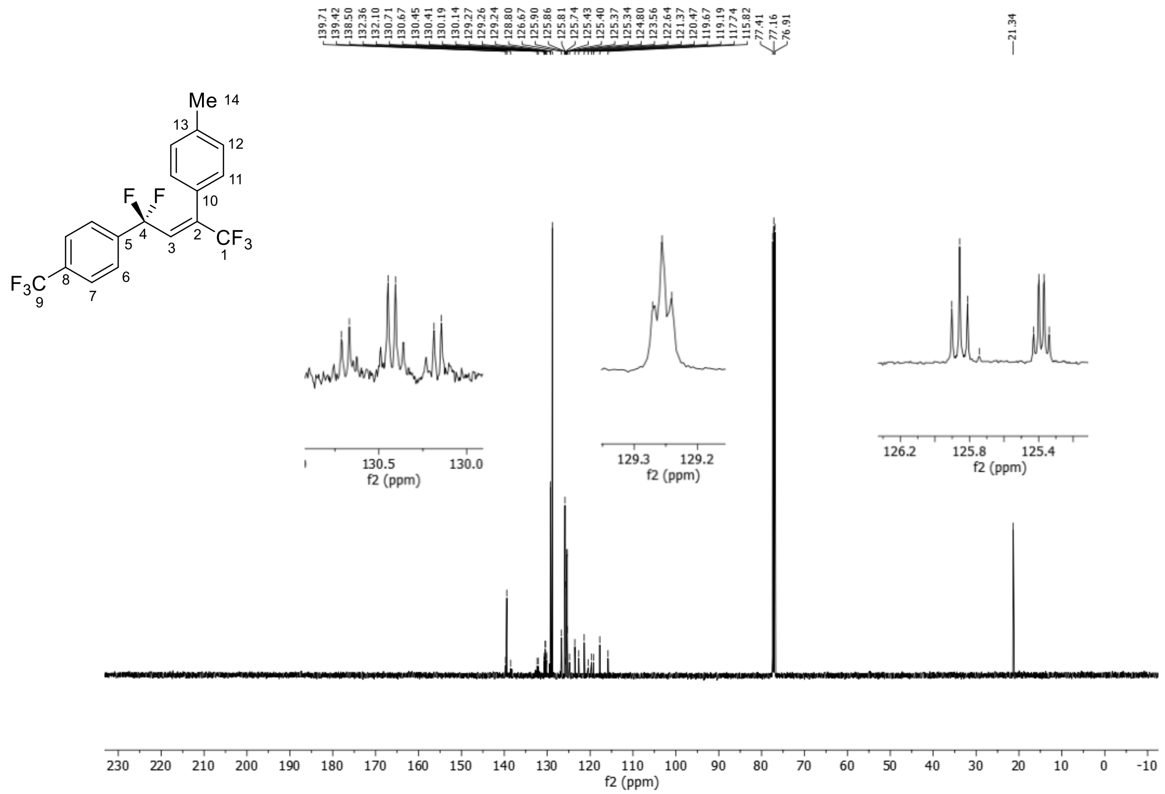

Figure S115. $^{13}C\{^{1}H\}$ NMR of **5** (126 MHz, 299 K, $CDCl_3$).

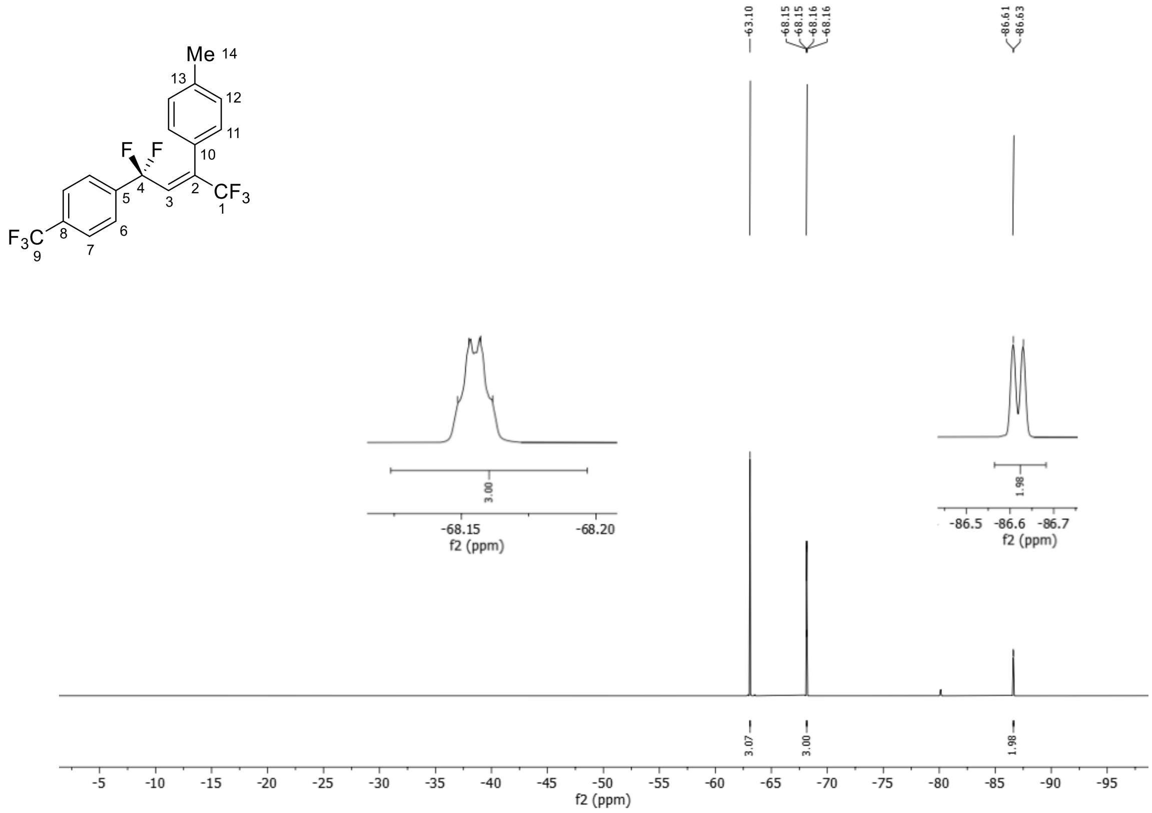

Figure S116. ^{19}F NMR of **5** (470 MHz, 299 K, $CDCl_3$).

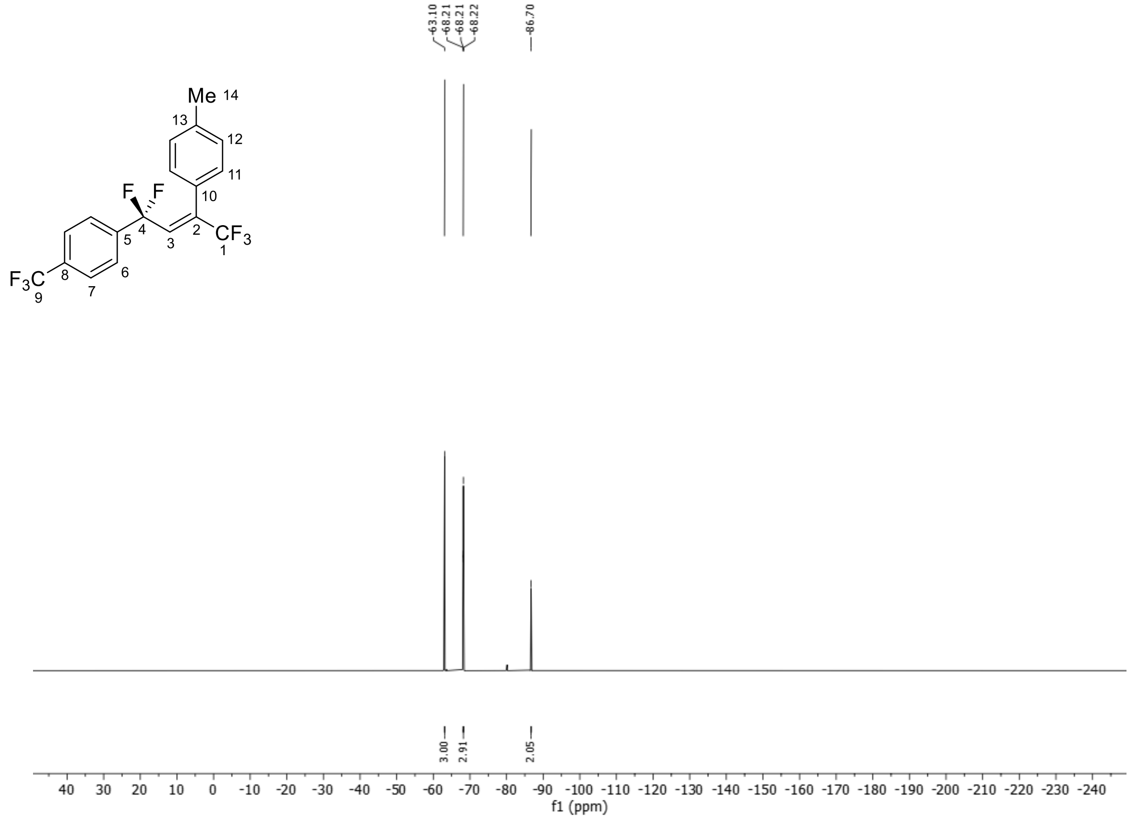

Figure S117. $^{19}F\{^1H\}$ NMR of **5** (377 MHz, 299 K, $CDCl_3$).

(*E*)-1-(3-Benzyl-1,1,4,4,4-pentafluorobut-2-en-1-yl)-4-(trifluoromethyl)benzene (*E*-6)

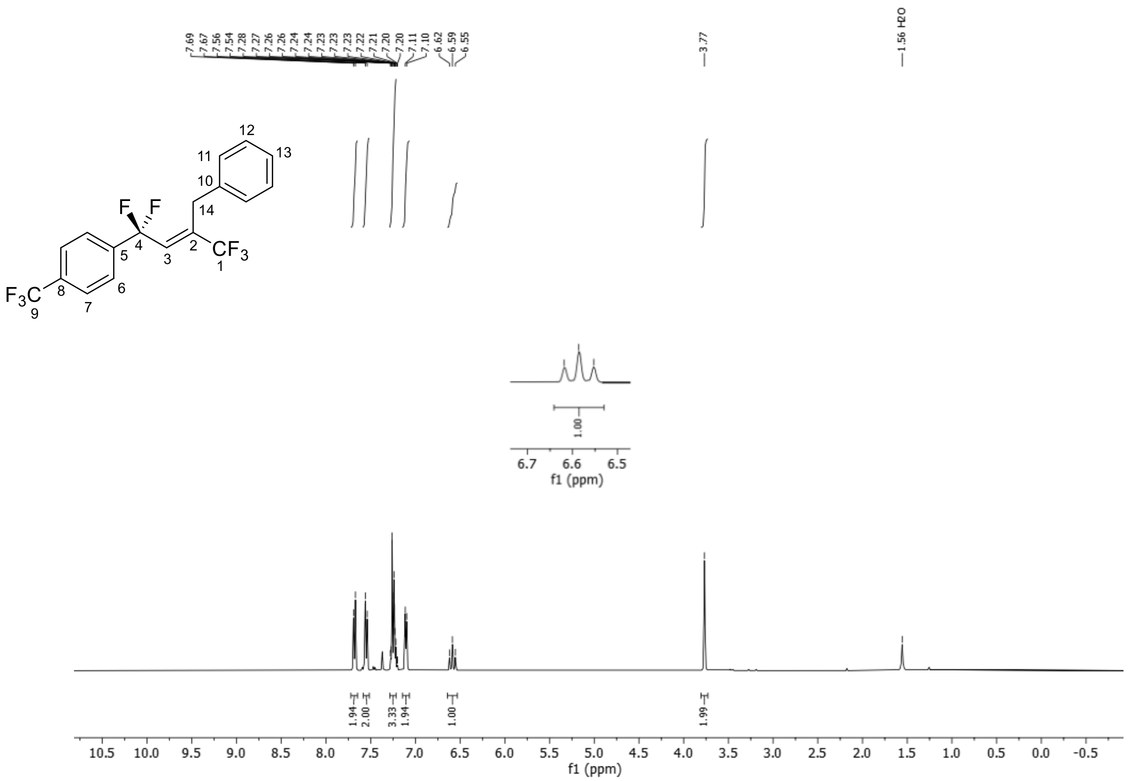

Figure S118. ^{1}H NMR of ***E*-6** (400 MHz, 299 K, $CDCl_3$).

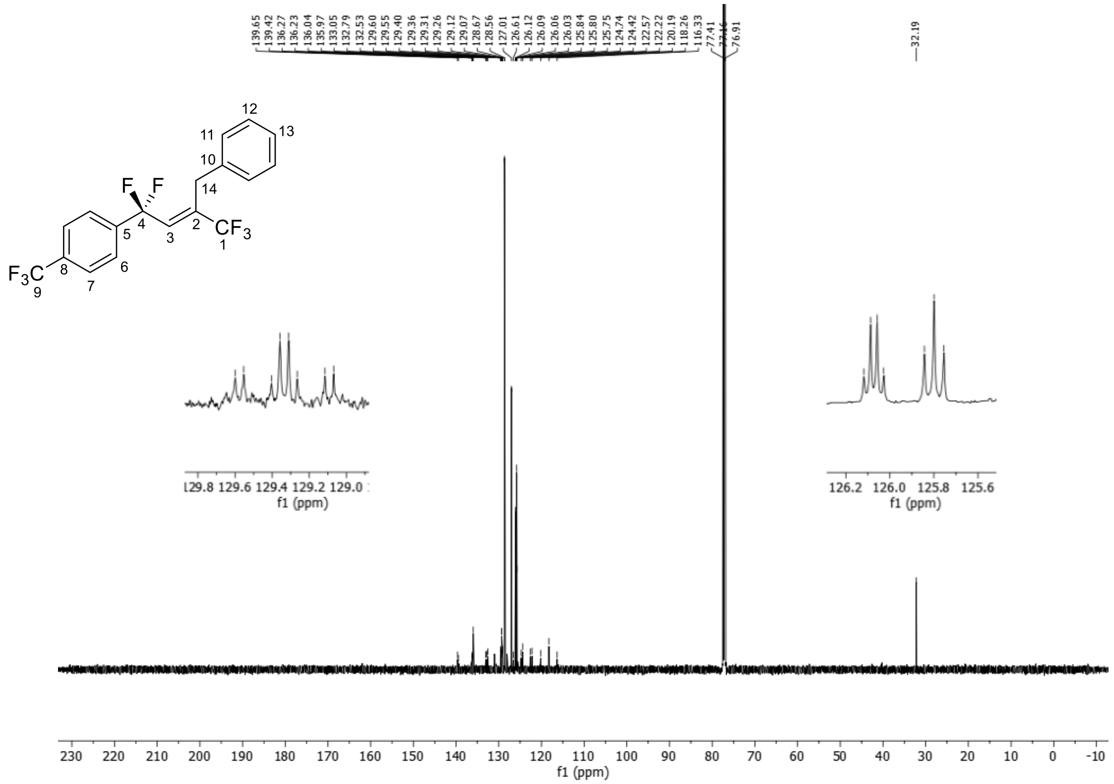

Figure S119. $^{13}C\{^{1}H\}$ NMR of ***E*-6** (126 MHz, 299 K, $CDCl_3$).

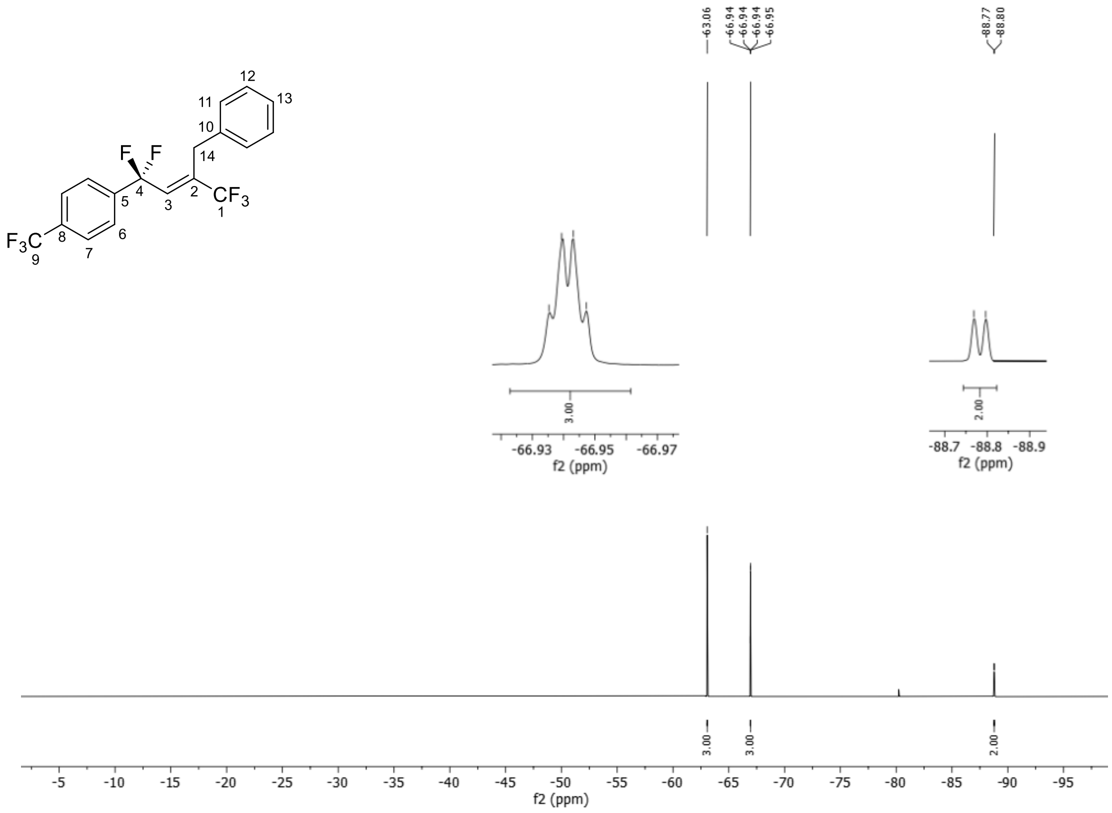

Figure S120. ^{19}F NMR of ***E*-6** (470 MHz, 299 K, $CDCl_3$).

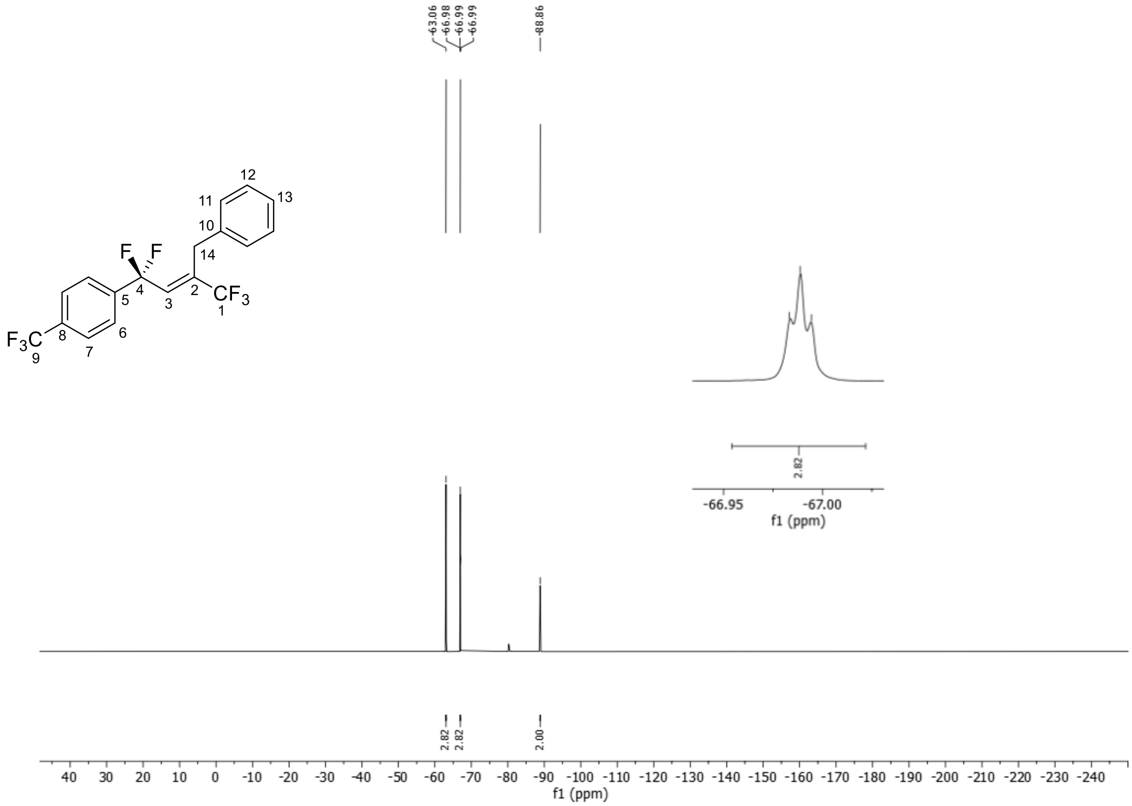

Figure S121. $^{19}F\{^1H\}$ NMR of ***E*-6** (377 MHz, 299 K, $CDCl_3$).

(*Z*)-1-(3-benzyl-1,1,4,4,4-pentafluorobut-2-en-1-yl)-4-(trifluoromethyl)benzene (*Z*-6)

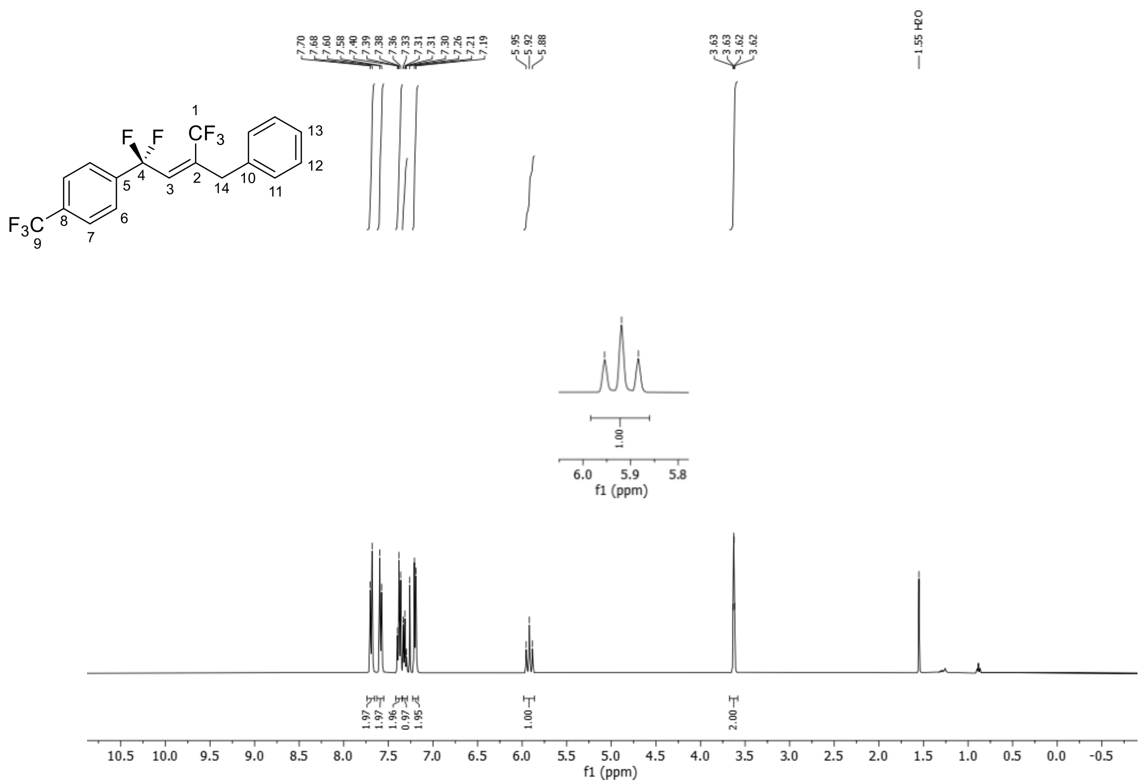

Figure S122. ^{1}H NMR of ***Z*-6** (400 MHz, 299 K, $CDCl_3$).

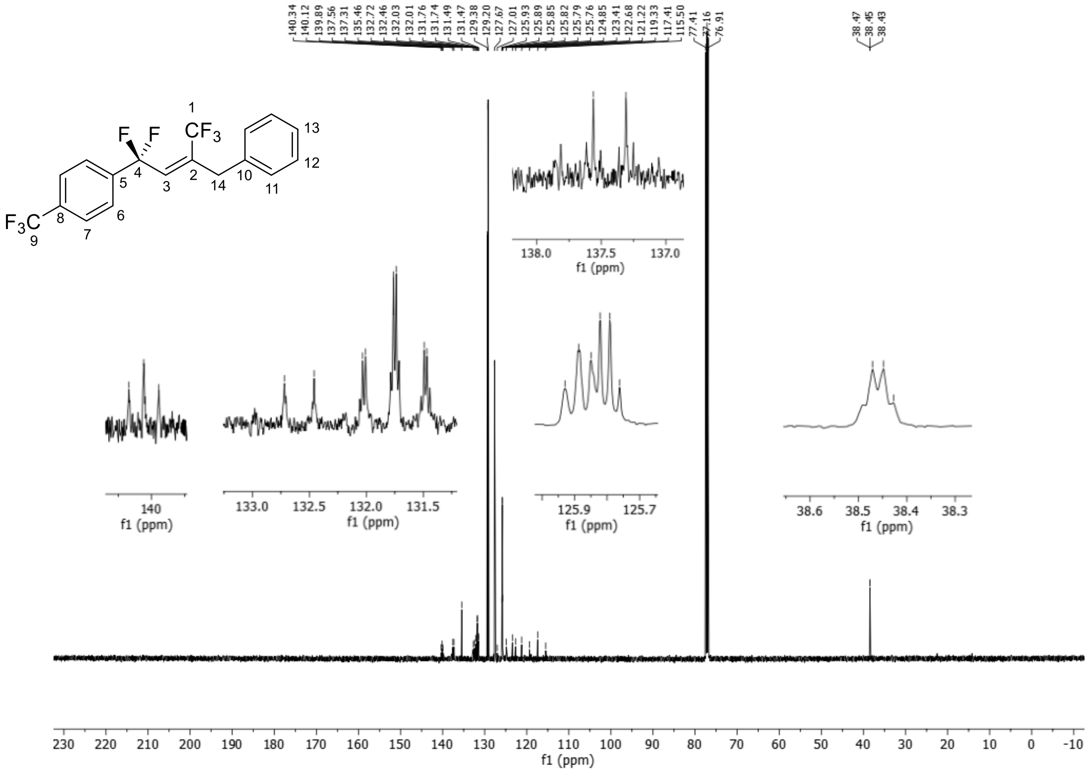

Figure S123. $^{13}C\{^{1}H\}$ NMR of ***Z*-6** (126 MHz, 299 K, $CDCl_3$).

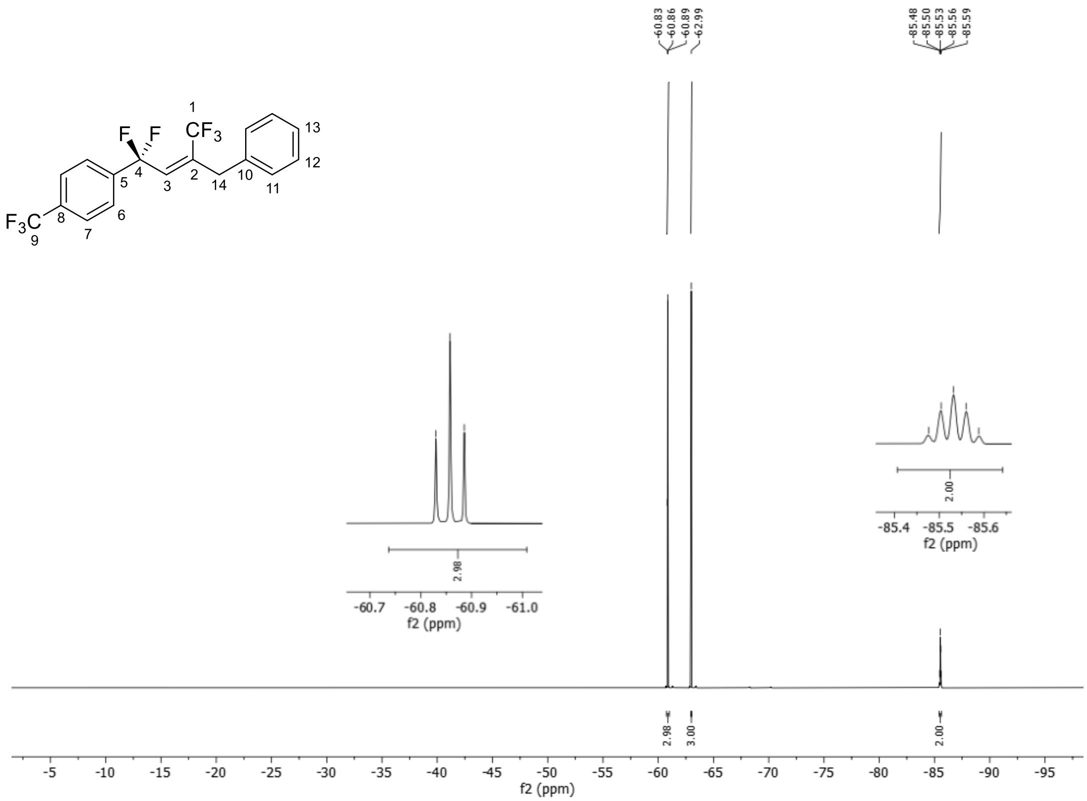

Figure S124. ^{19}F NMR of ***Z*-6** (470 MHz, 299 K, $CDCl_3$).

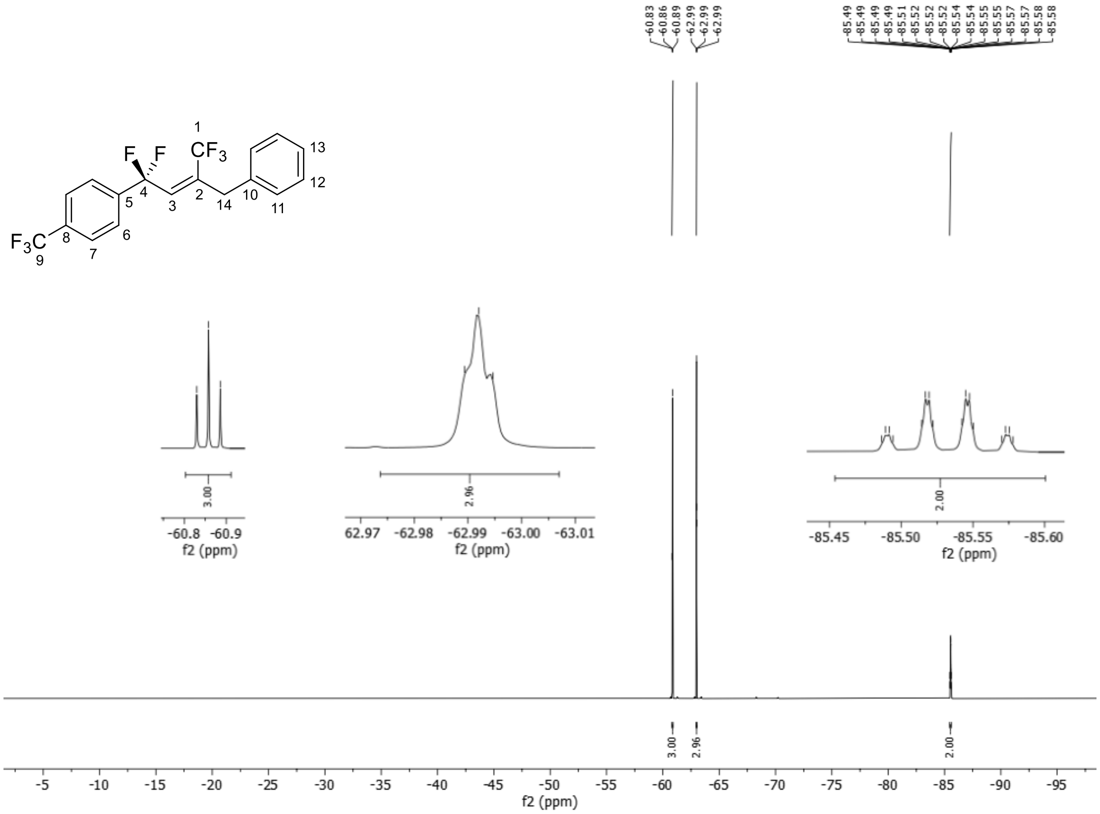

Figure S125. $^{19}F\{^1H\}$ NMR of ***Z*-6** (470 MHz, 299 K, $CDCl_3$).

(E)-1-(3-(4-Bromophenyl)-1,1,4,4,4-pentafluorobut-2-en-1-yl)-3,5-dichlorobenzene (7)

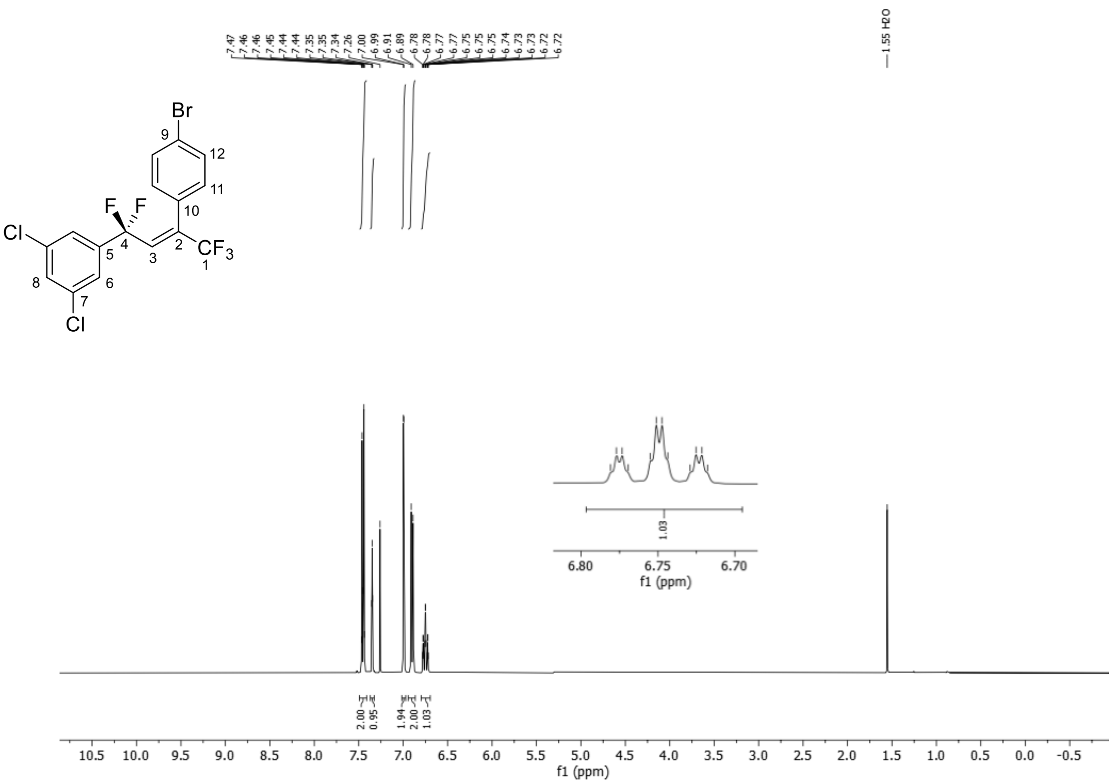

Figure S126. ^{1}H NMR of **7** (400 MHz, 299 K, $CDCl_3$).

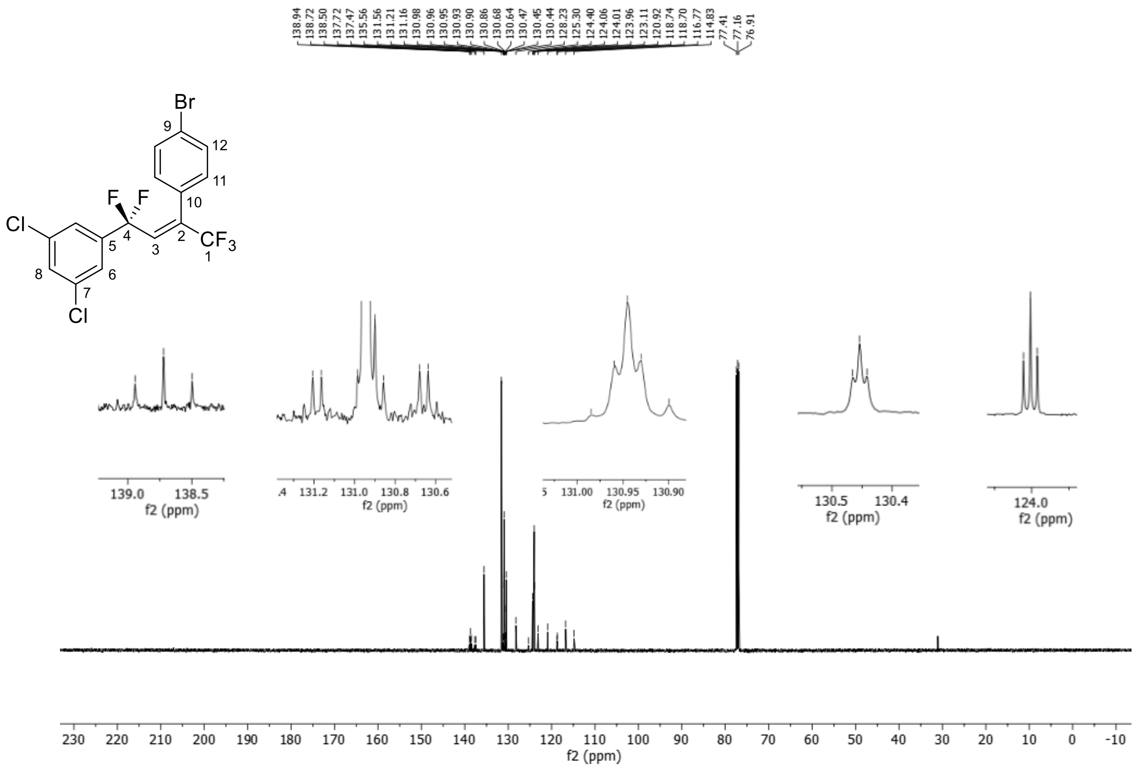

Figure S127. $^{13}C\{^{1}H\}$ NMR of **7** (126 MHz, 299 K, $CDCl_3$).

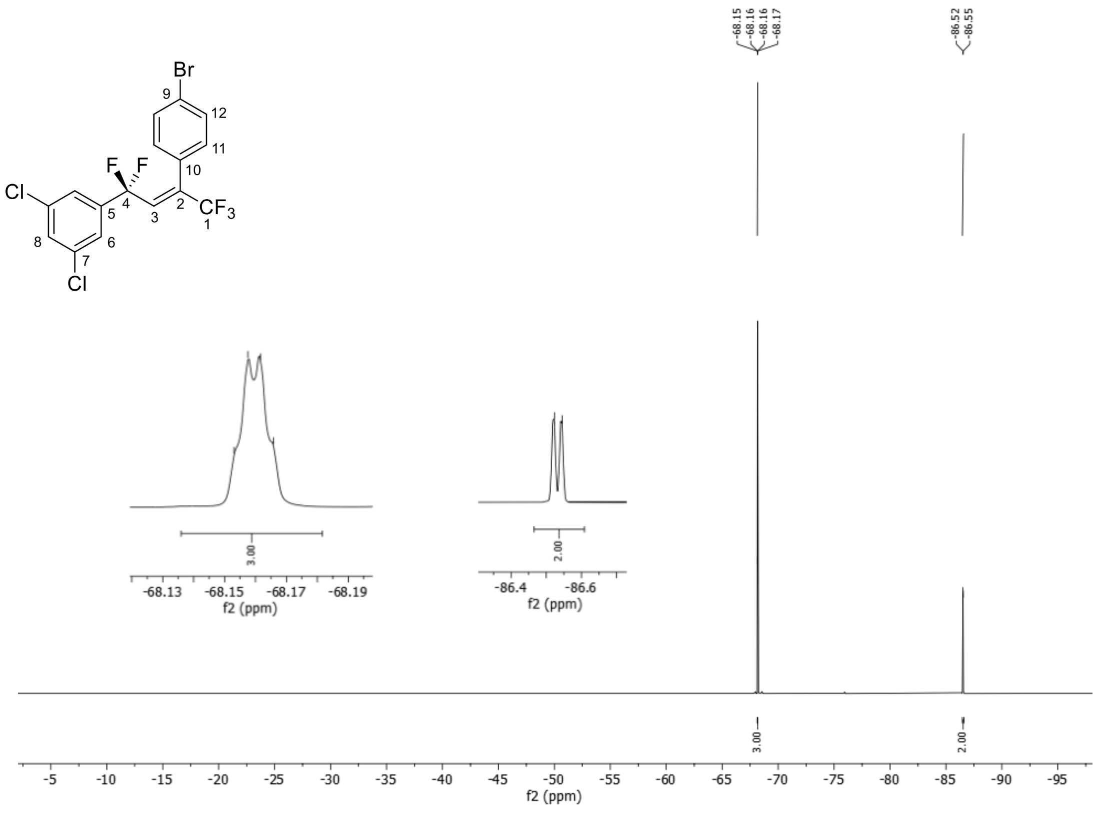

Figure S128. ^{19}F NMR of **7** (470 MHz, 299 K, $CDCl_3$).

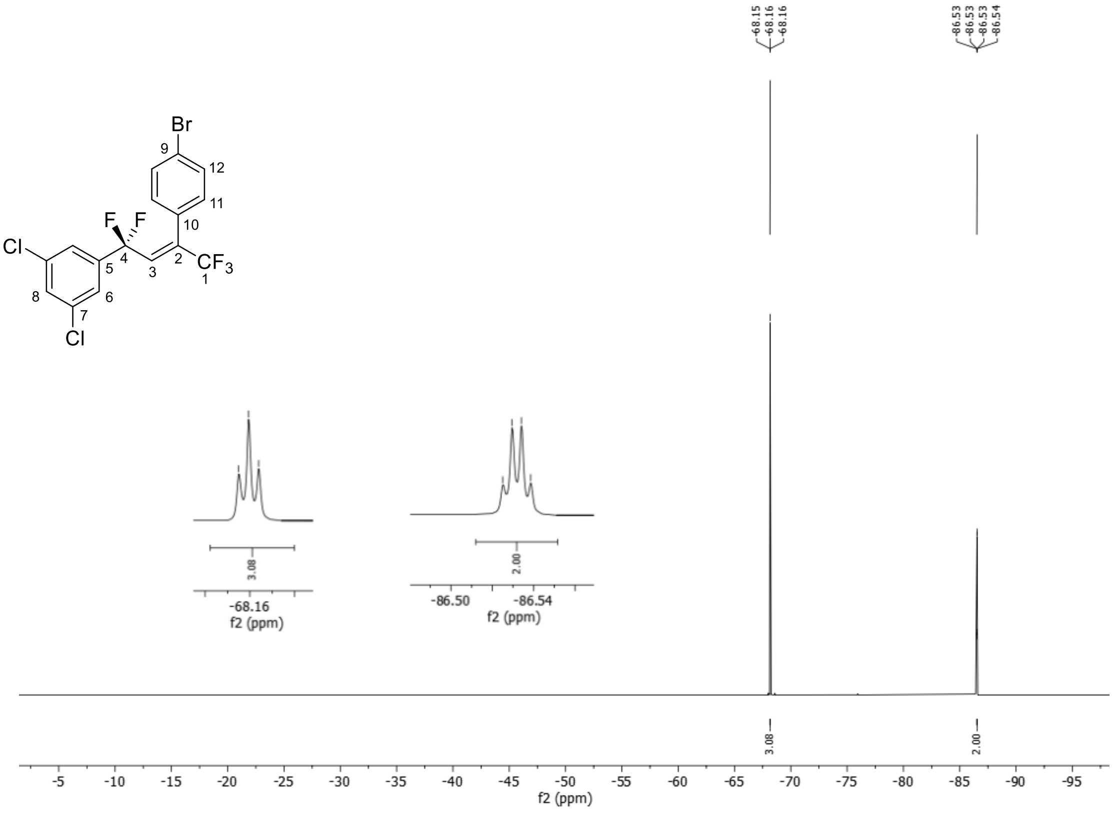

Figure S129. $^{19}F\{^1H\}$ NMR of **7** (470 MHz, 299 K, $CDCl_3$).

Methyl (*E*)-4-(3-(4-bromophenyl)-1,1,4,4,4-pentafluorobut-2-en-1-yl)benzoate (8)

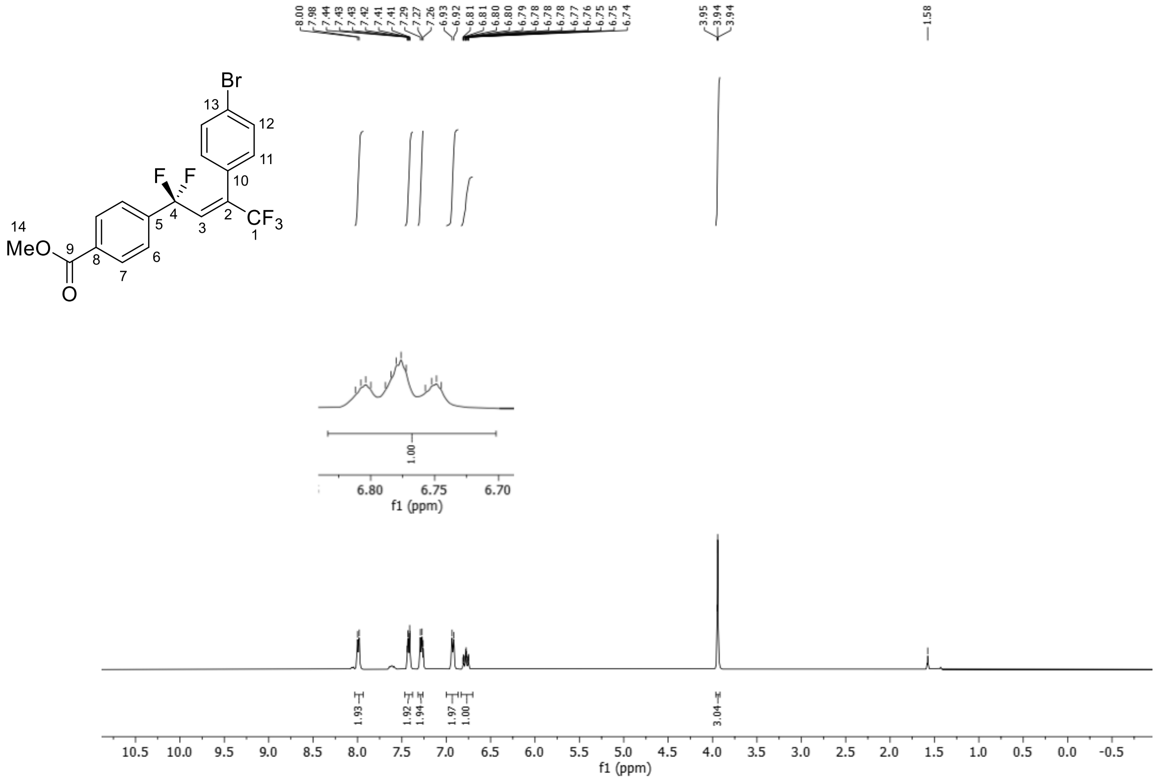

Figure S130. ^{1}H NMR of **8** (400 MHz, 299 K, $CDCl_3$).

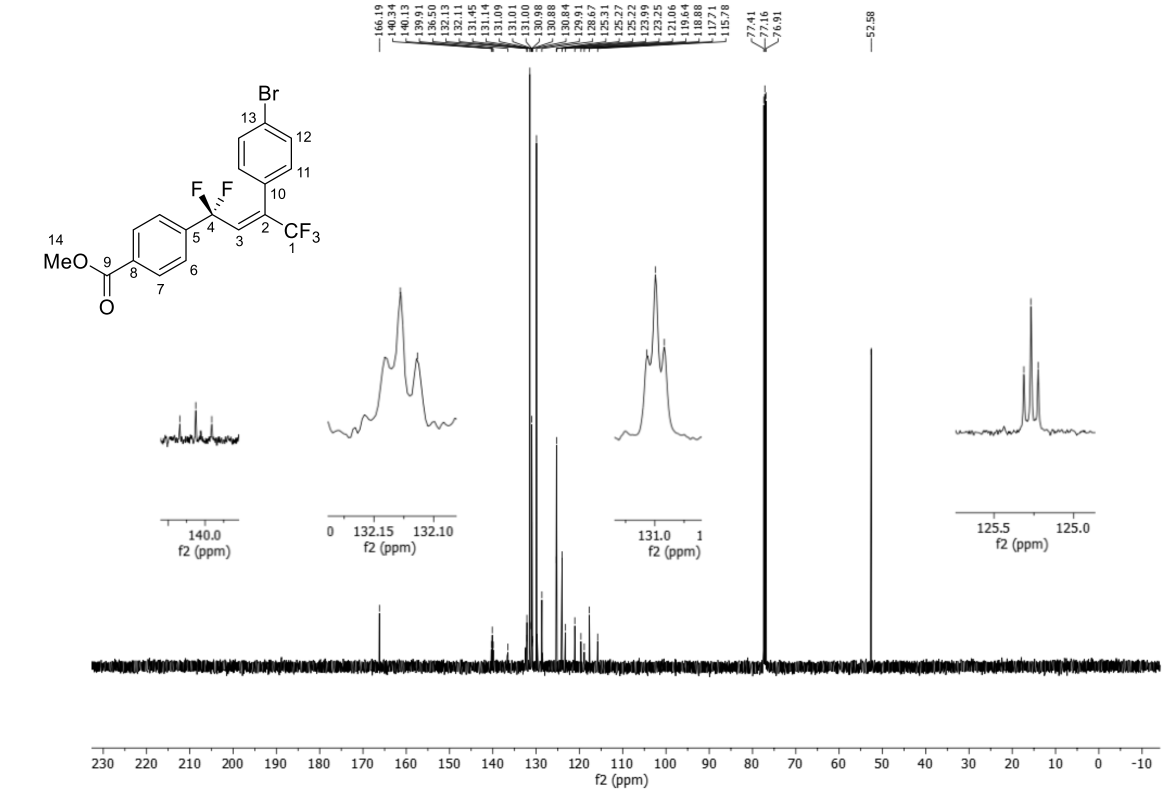

Figure S131. $^{13}C\{^{1}H\}$ NMR of **8** (126 MHz, 299 K, $CDCl_3$).

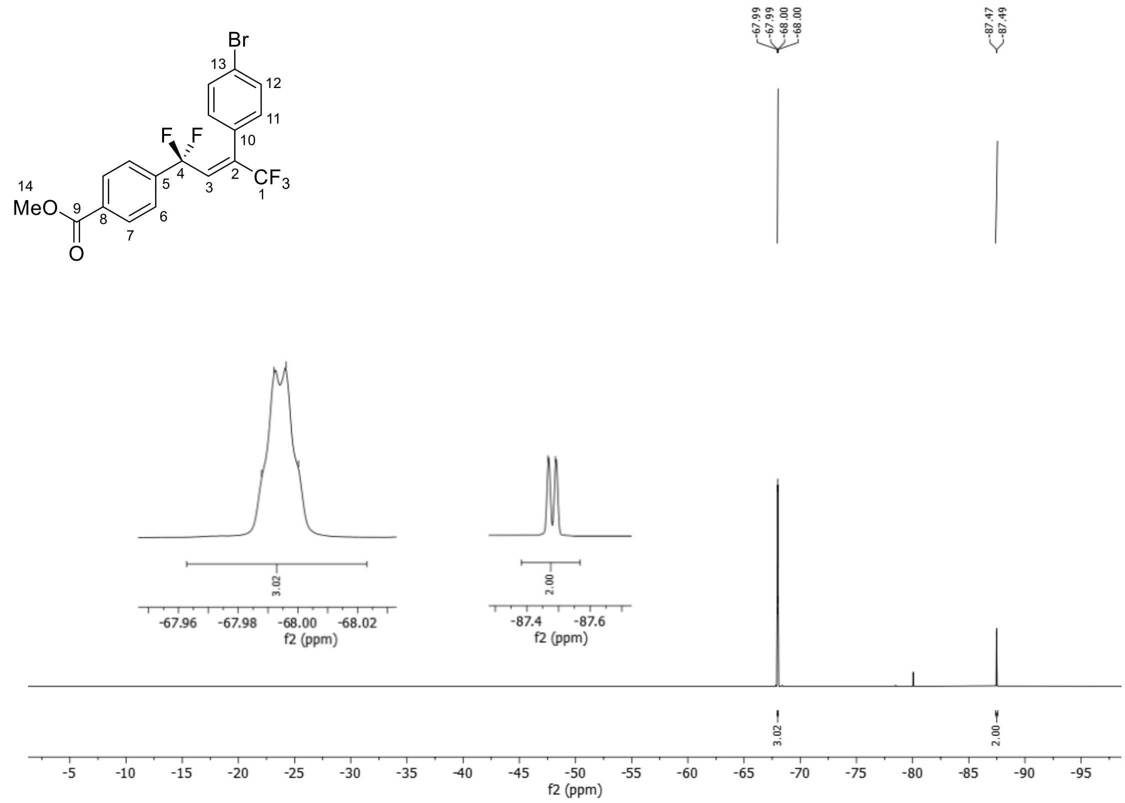

Figure S132. ^{19}F NMR of **8** (470 MHz, 299 K, $CDCl_3$).

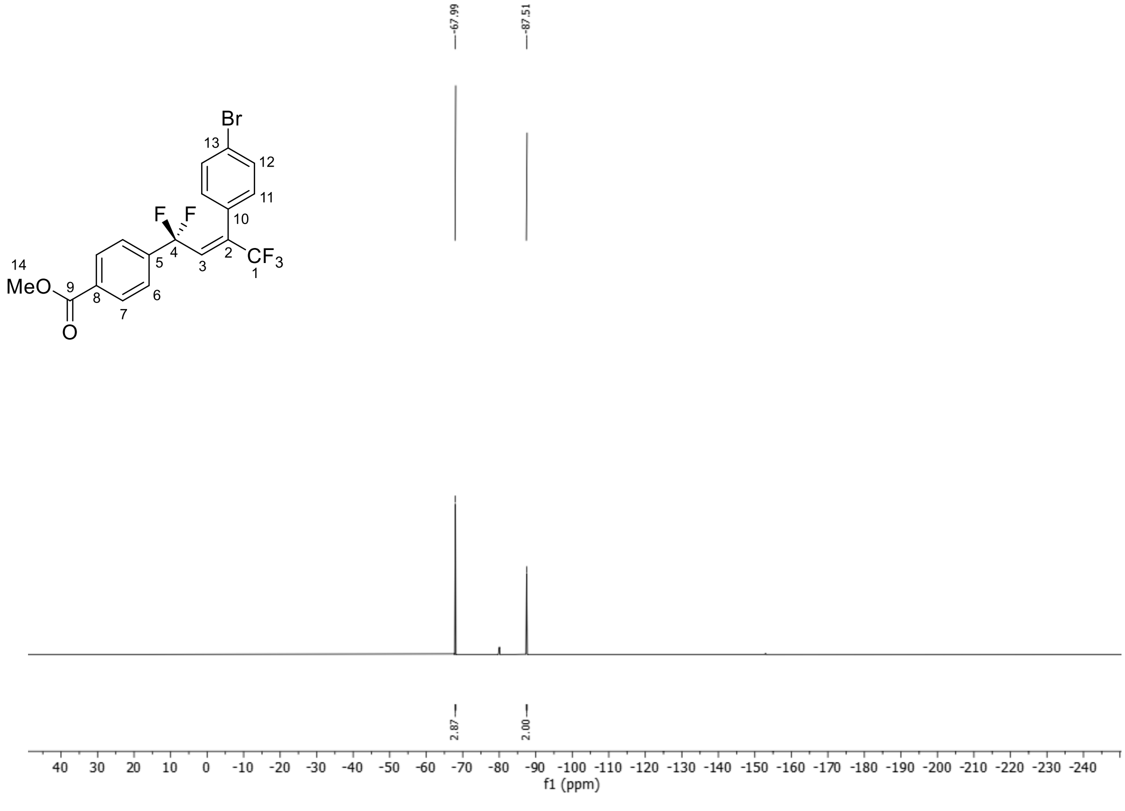

Figure S133. $^{19}F\{^1H\}$ NMR of **8** (377 MHz, 299 K, $CDCl_3$).

(*E*)-4-(3-(4-Bromophenyl)-1,1,4,4,4-pentafluorobut-2-en-1-yl)benzonitrile (9)

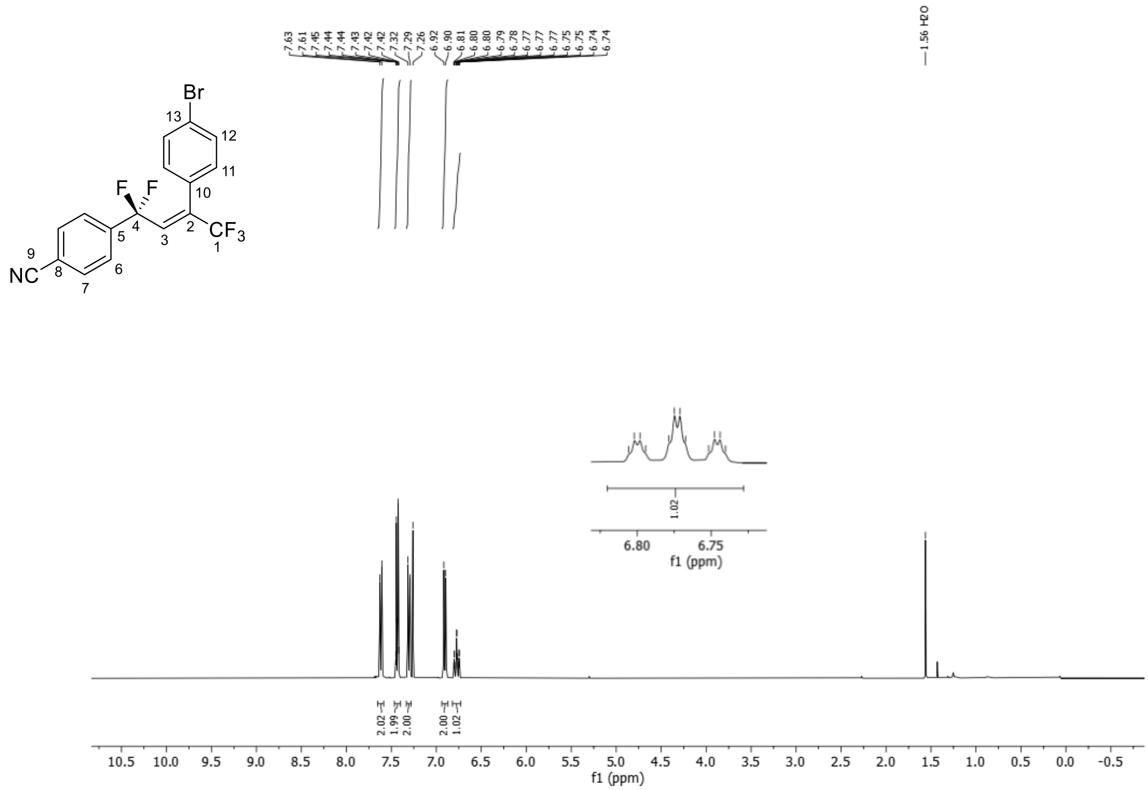

Figure S134. ^{1}H NMR of **9** (400 MHz, 299 K, $CDCl_3$).

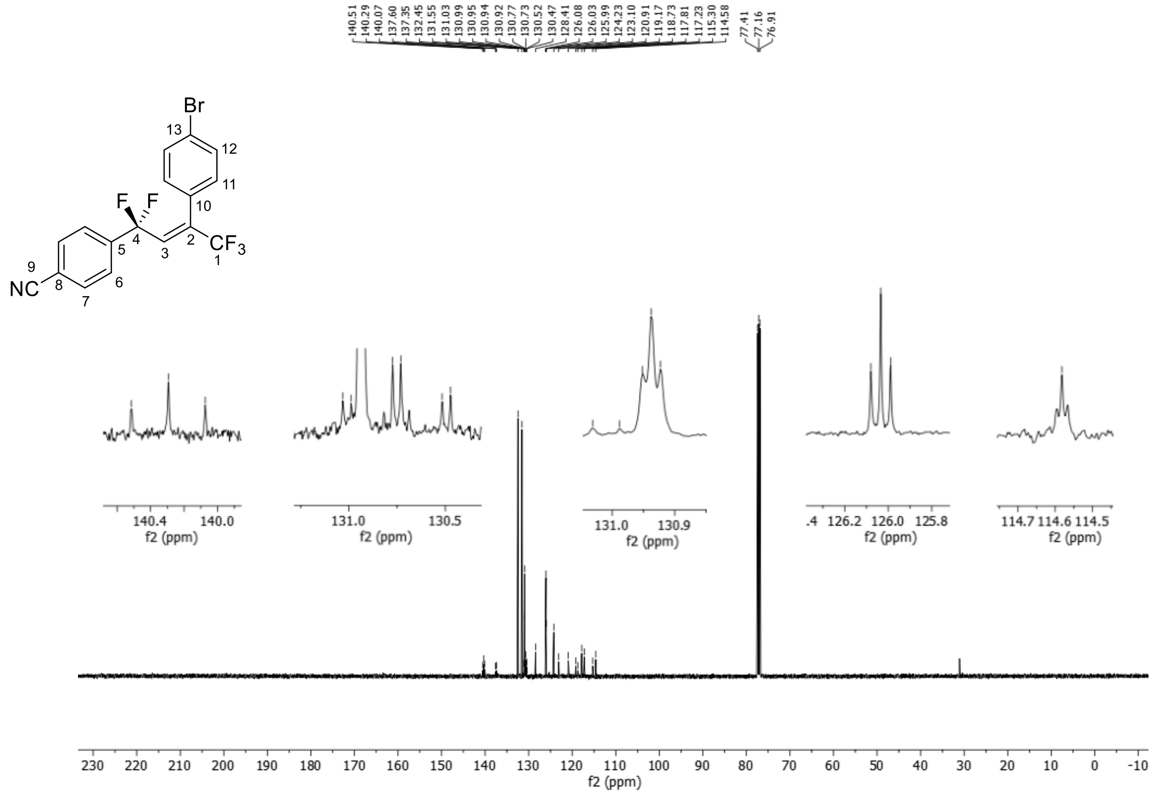

Figure S135. $^{13}C\{^{1}H\}$ NMR of **9** (126 MHz, 299 K, $CDCl_3$).

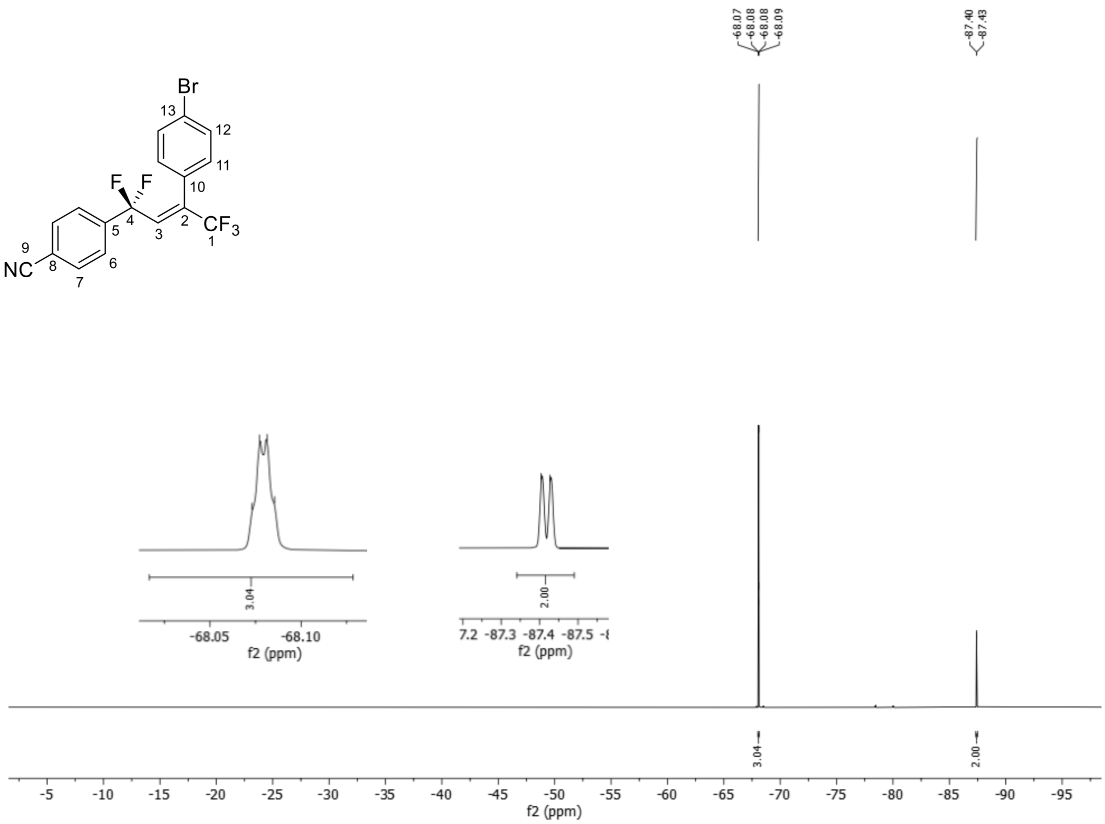

Figure S136. ^{19}F NMR of **9** (470 MHz, 299 K, $CDCl_3$).

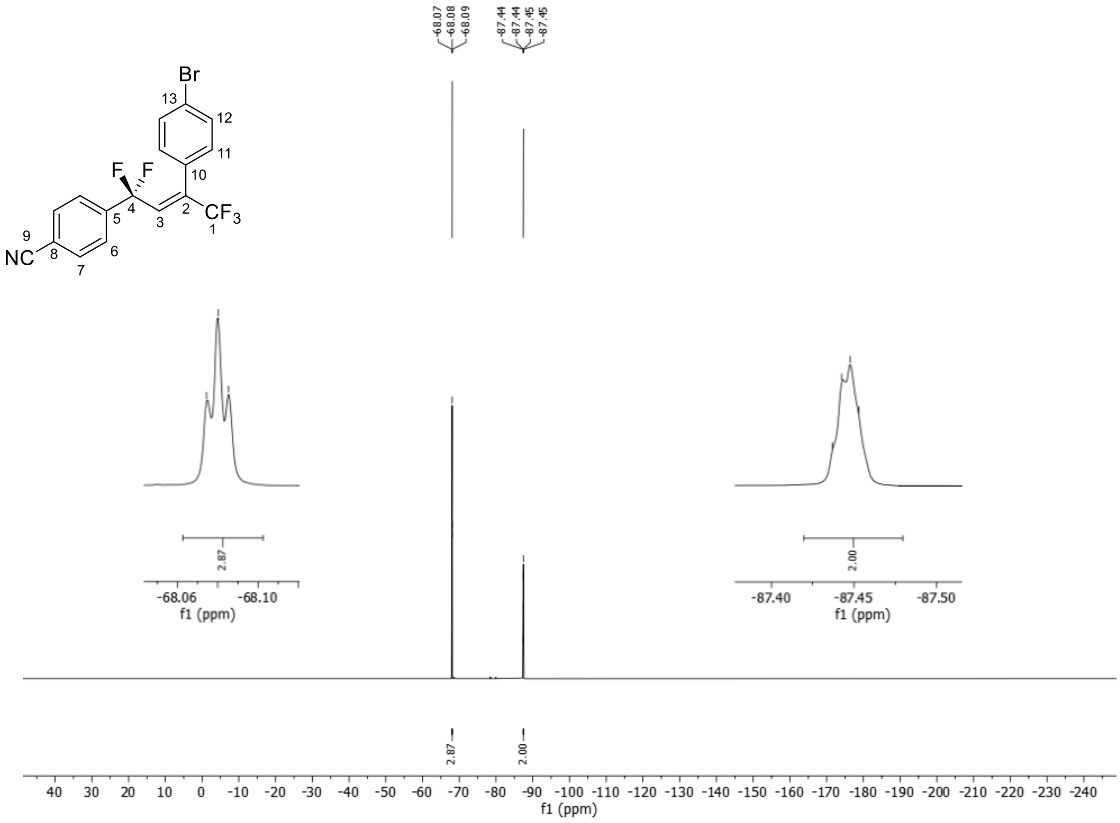

Figure S137. $^{19}F\{^1H\}$ NMR of **9** (377 MHz, 299 K, $CDCl_3$).

(*E*)-(1,1,1,4,4-Pentafluorodec-2-en-2-yl)benzene (10)

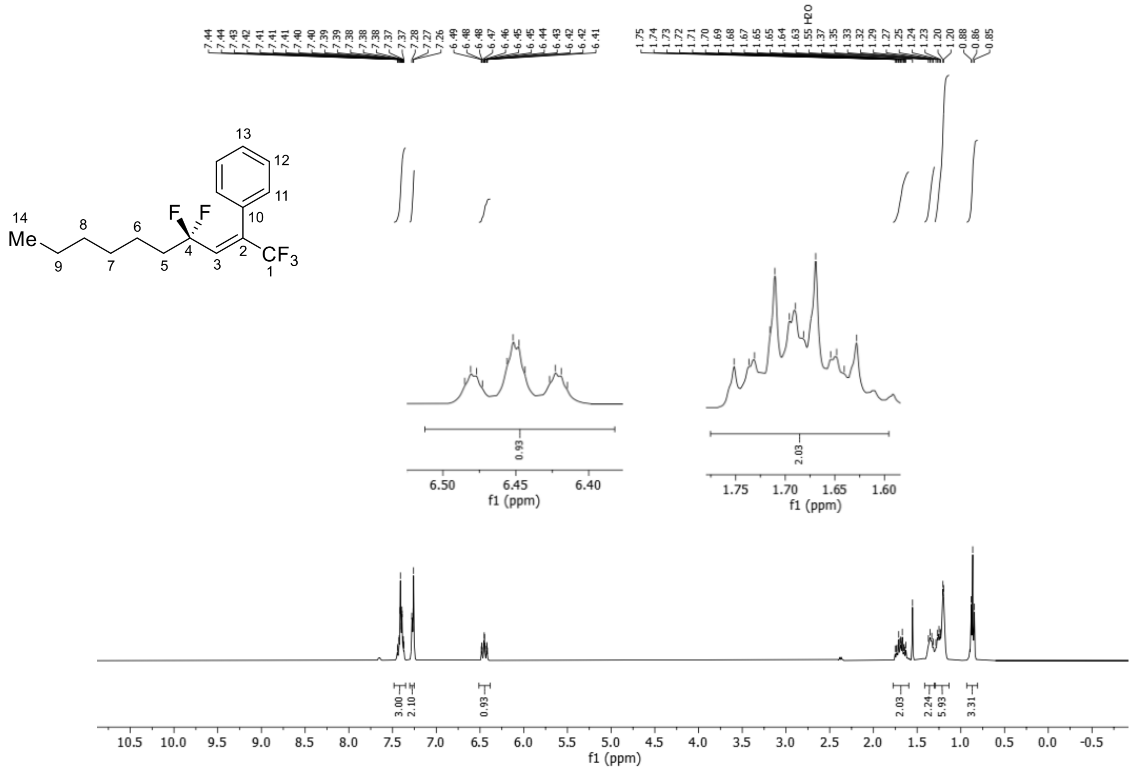

Figure S138. ^{1}H NMR of **10** (400 MHz, 299 K, $CDCl_3$).

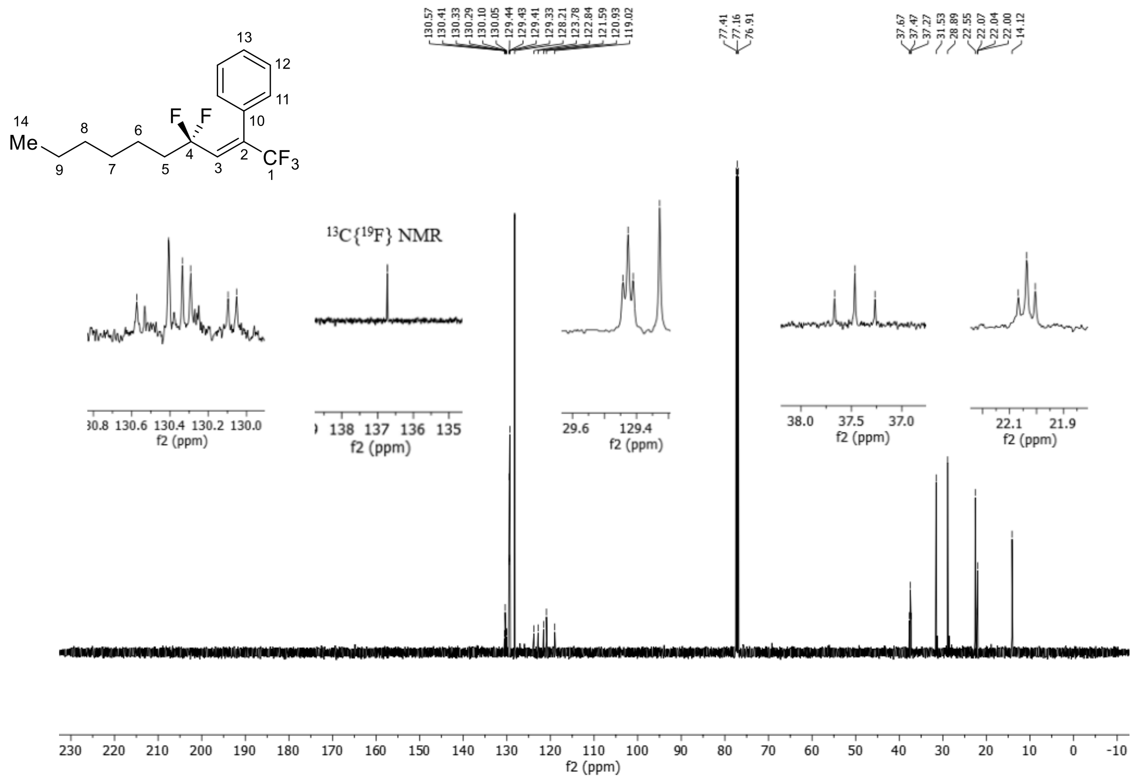

Figure S139. $^{13}C\{^{1}H\}$ NMR of **10** (126 MHz, 299 K, $CDCl_3$).

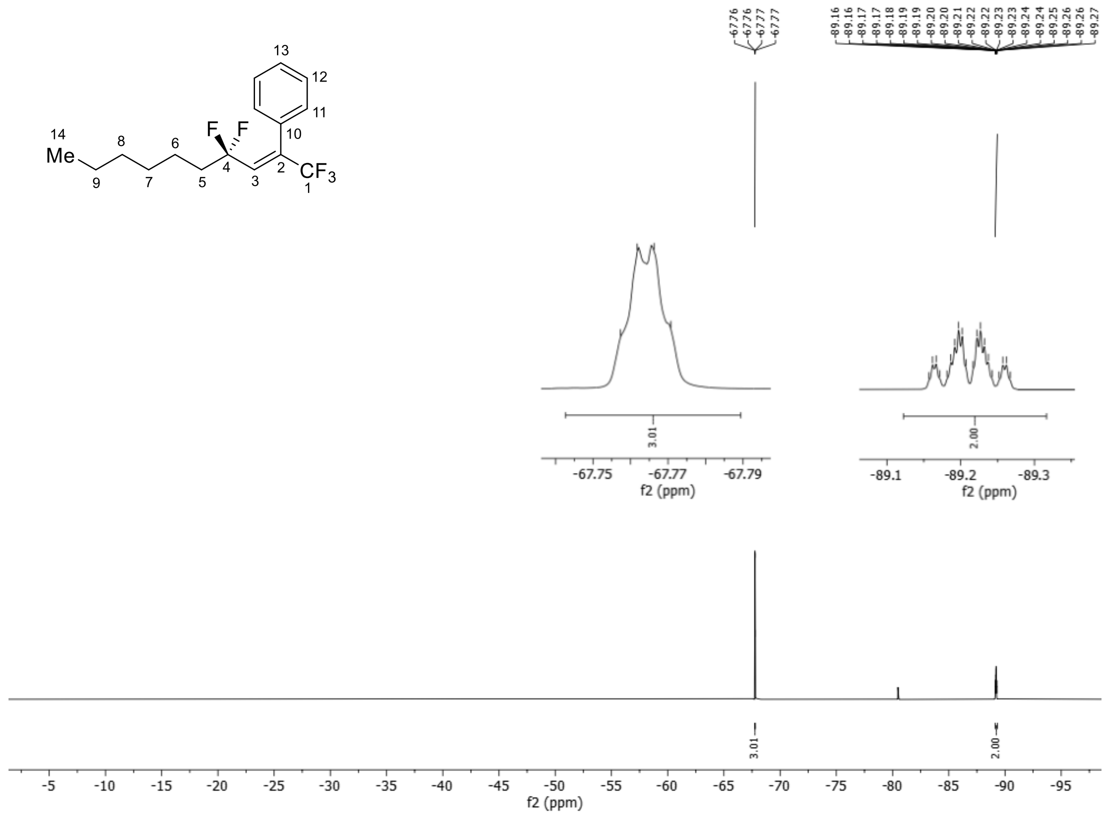

Figure S140. ^{19}F NMR of **10** (470 MHz, 299 K, $CDCl_3$).

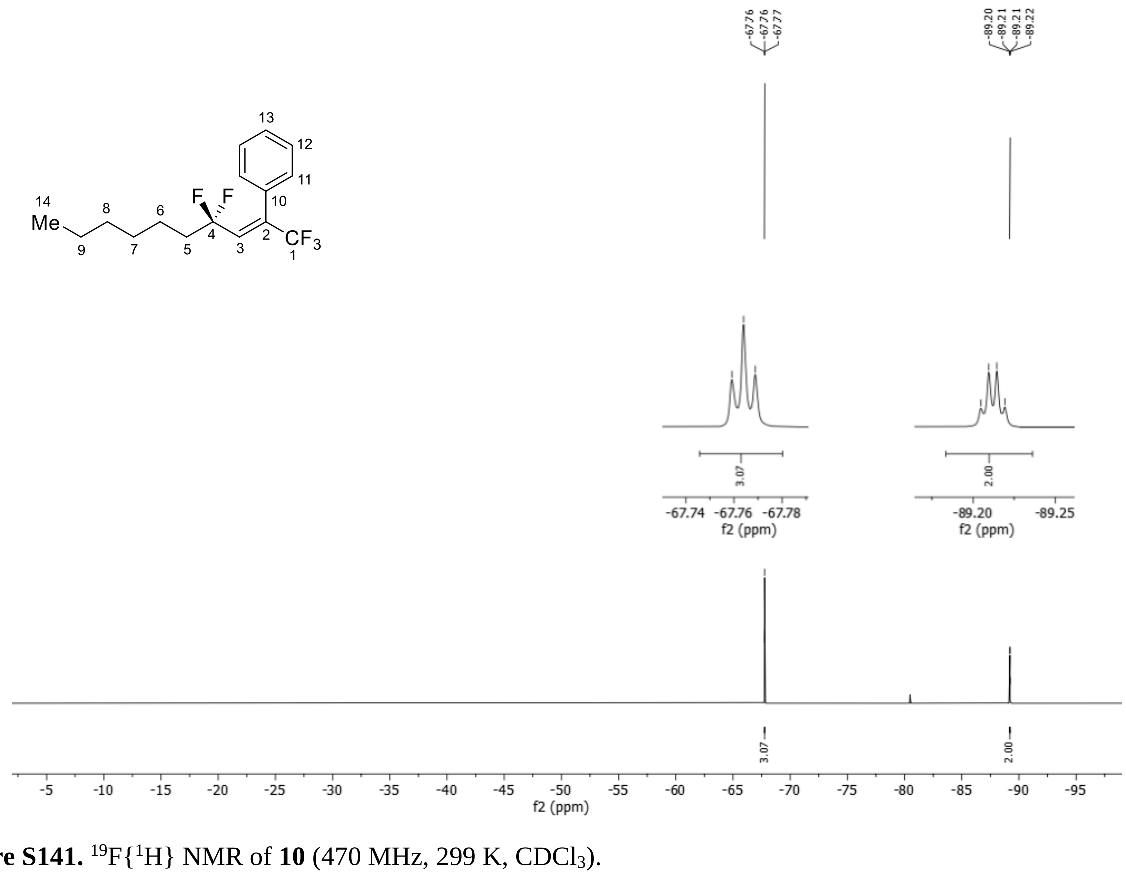

Figure S141. $^{19}F\{^1H\}$ NMR of **10** (470 MHz, 299 K, $CDCl_3$).

(*E*)-(5-Cyclohexyl-1,1,1,4,4-pentafluoropent-2-en-2-yl)benzene (11)

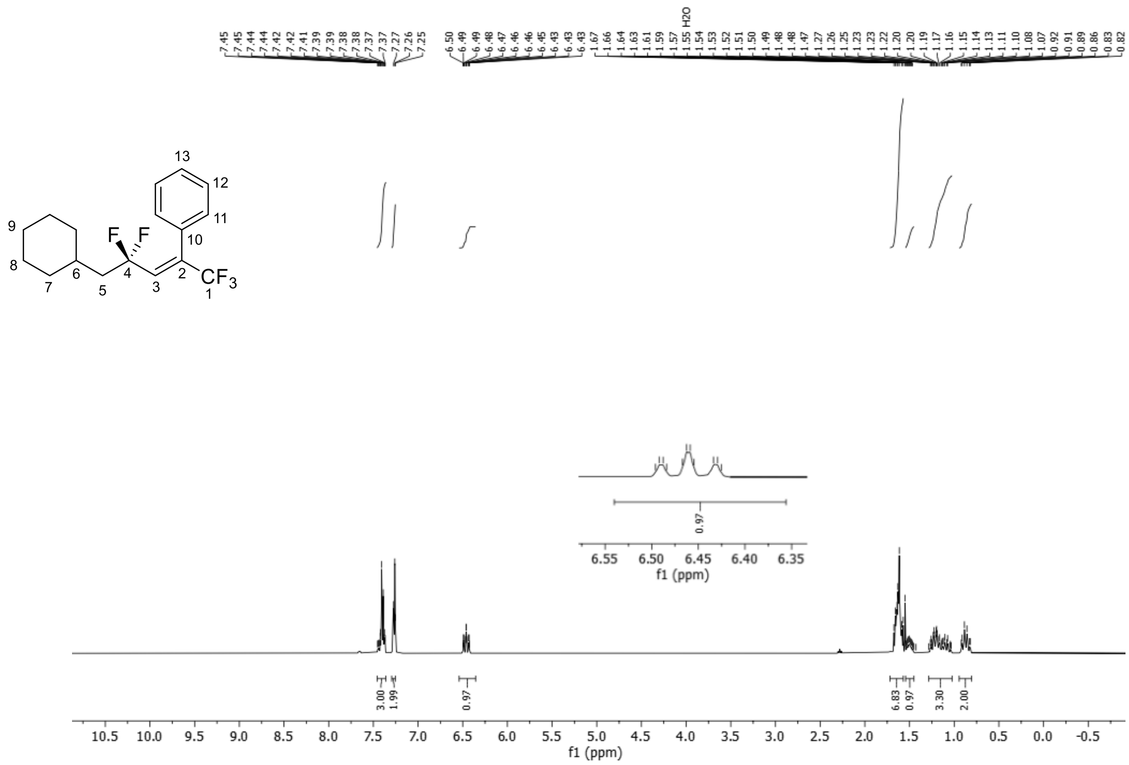

Figure S142. ^{1}H NMR of **11** (400 MHz, 299 K, $CDCl_3$).

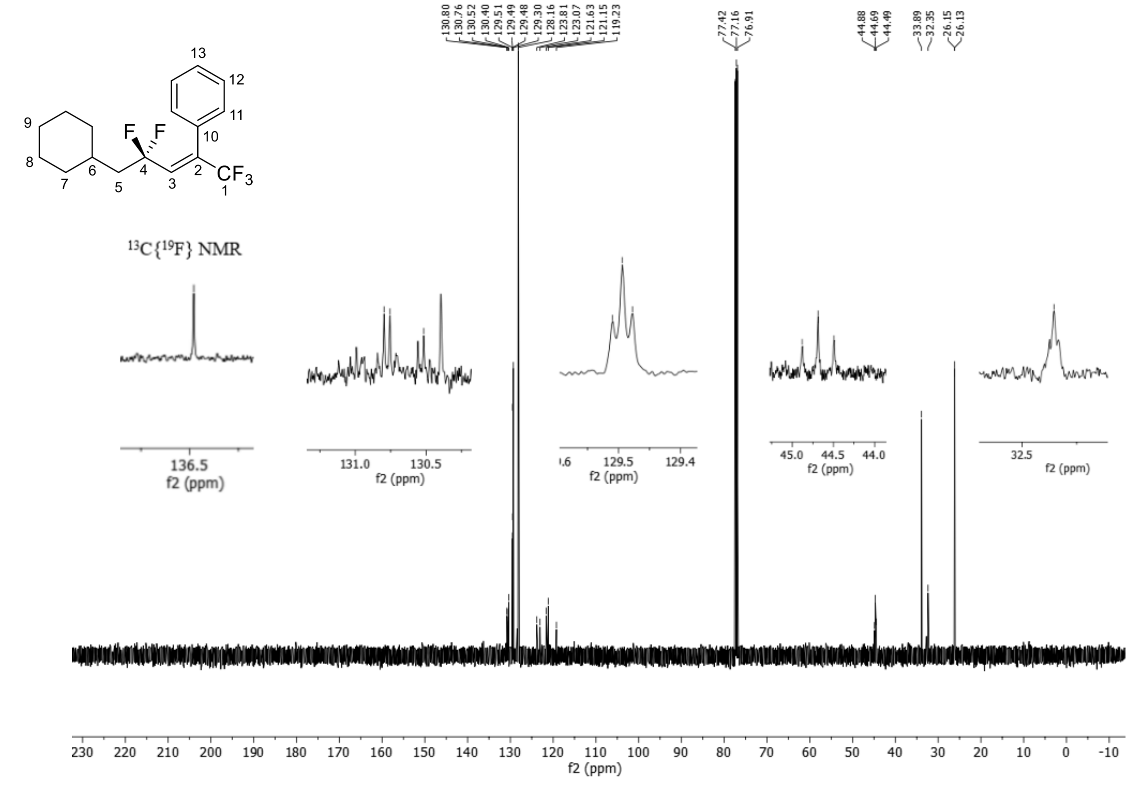

Figure S143. $^{13}C\{^{1}H\}$ NMR of **11** (126 MHz, 299 K, $CDCl_3$).

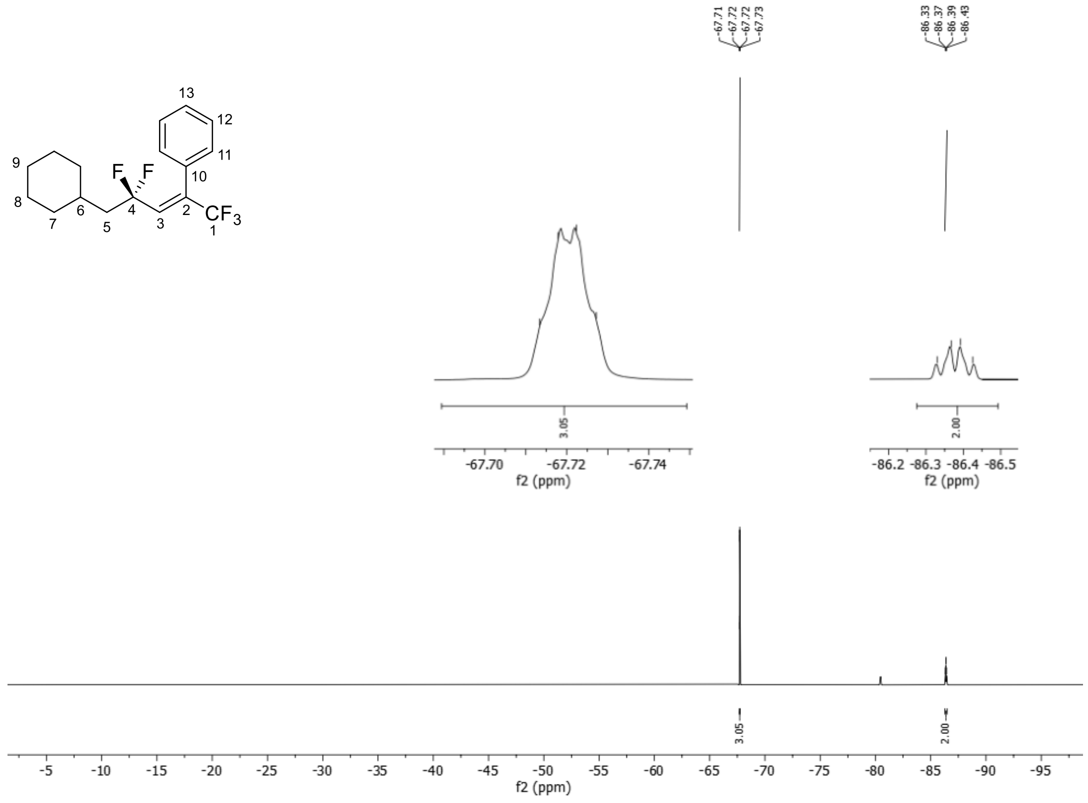

Figure S144. ^{19}F NMR of **11** (470 MHz, 299 K, $CDCl_3$).

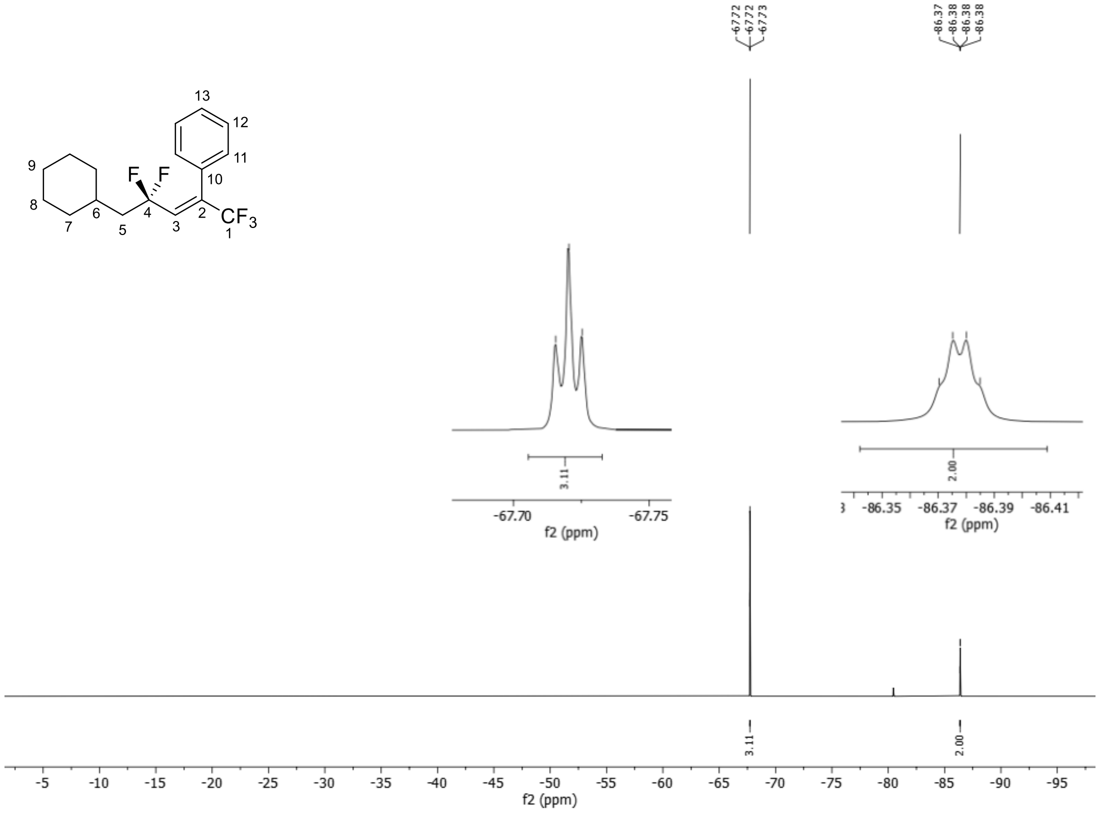

Figure S145. $^{19}F\{^1H\}$ NMR of **11** (470 MHz, 299 K, $CDCl_3$).

(*E*)-10,10,13,13,13-Pentafluoro-12-phenyltridec-11-en-1-yl cyclopropanecarboxylate (12)

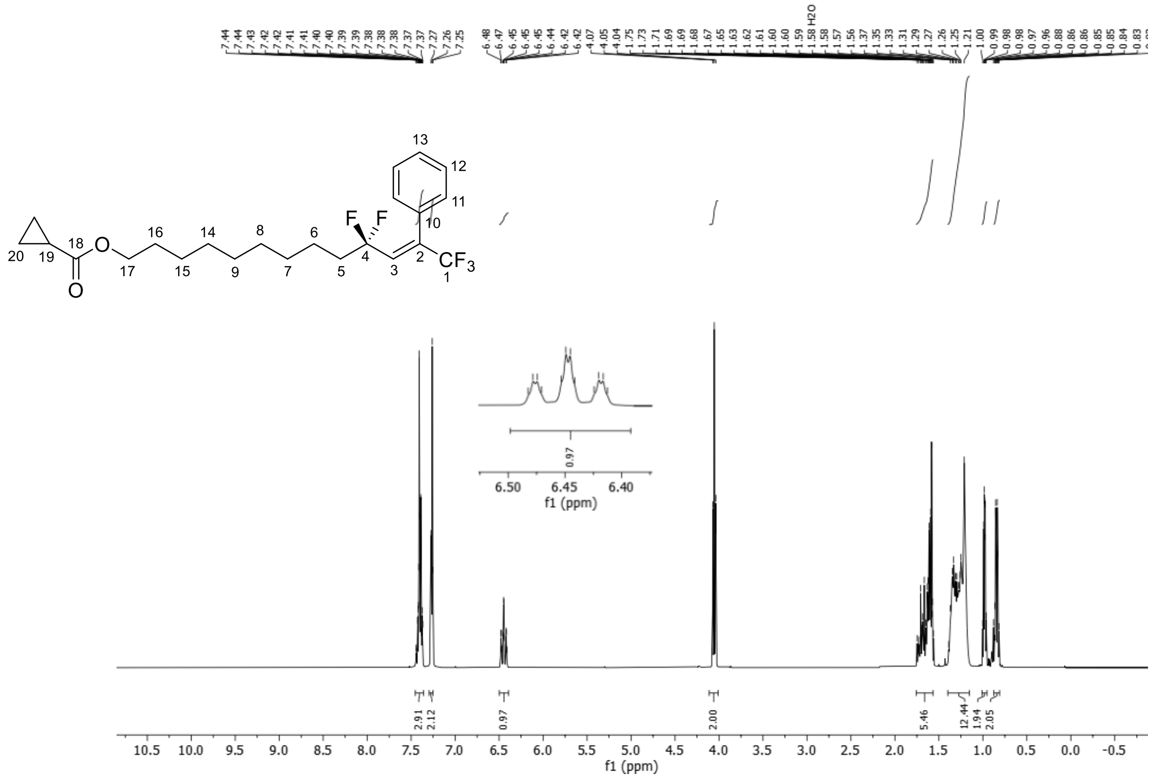

Figure S146. ^{1}H NMR of **12** (400 MHz, 299 K, $CDCl_3$).

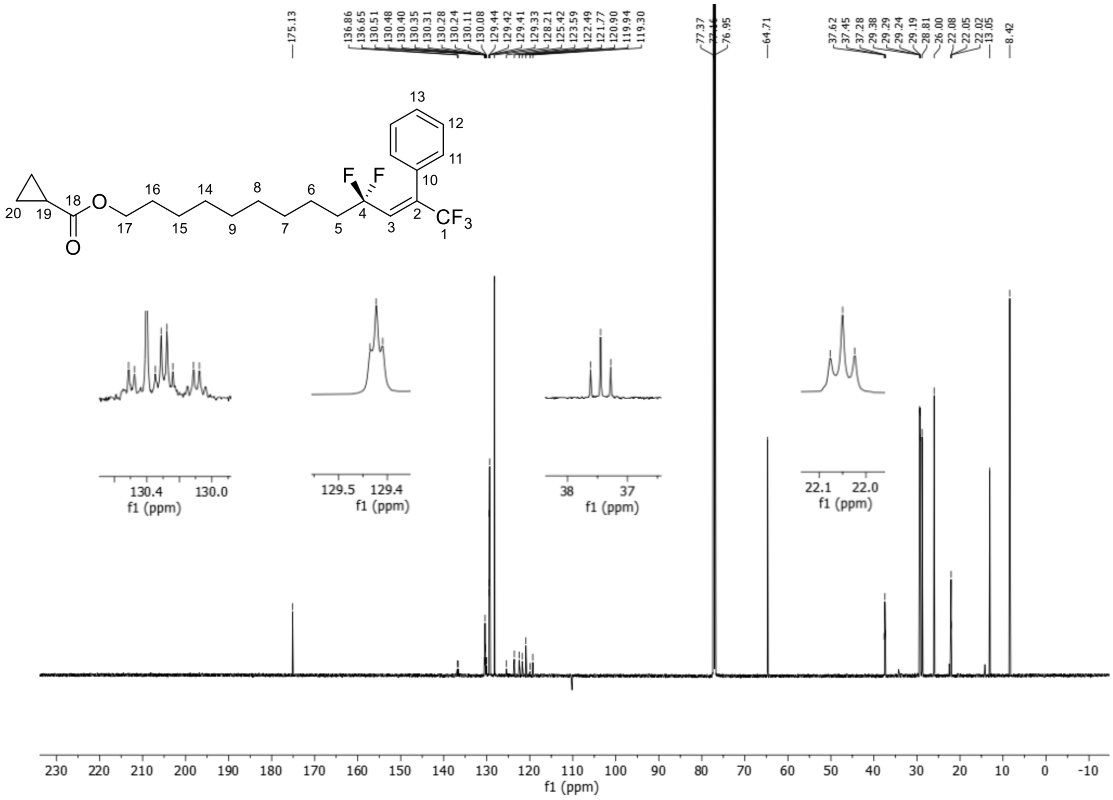

Figure S147. $^{13}C\{^{1}H\}$ NMR of **12** (126 MHz, 299 K, $CDCl_3$).

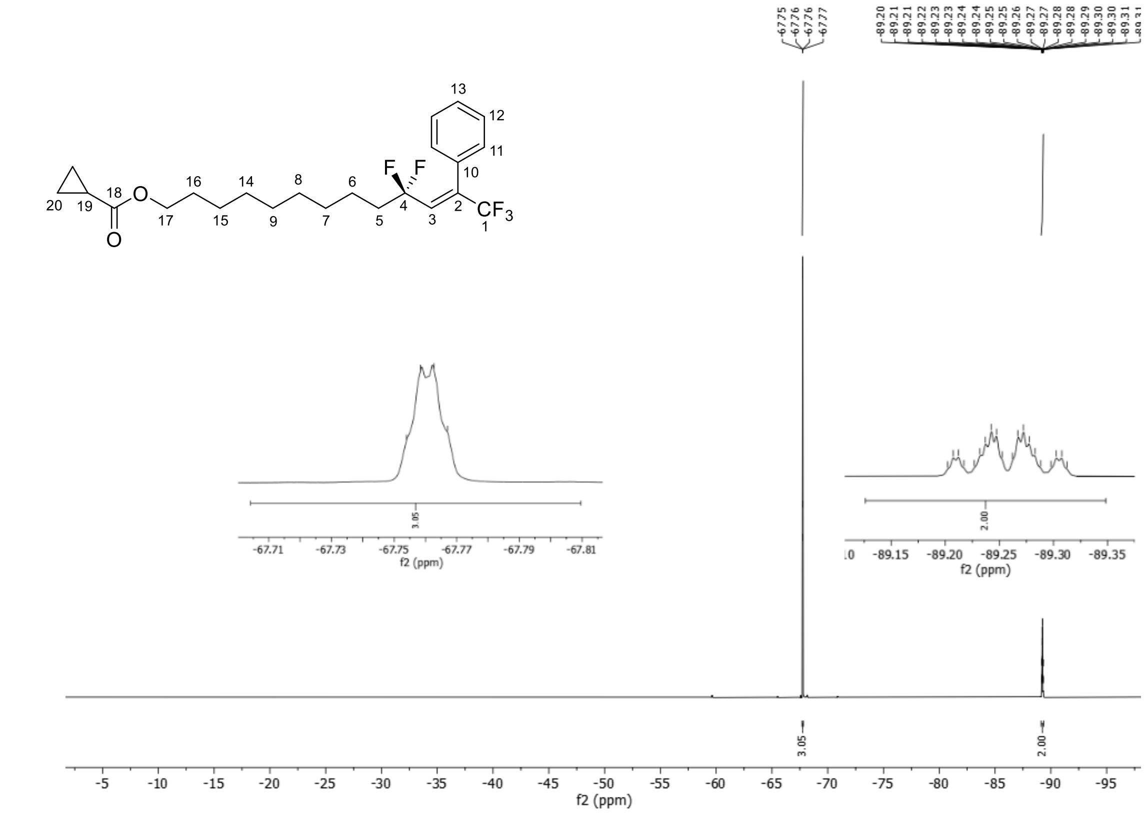

Figure S148. ^{19}F NMR of **12** (470 MHz, 299 K, $CDCl_3$).

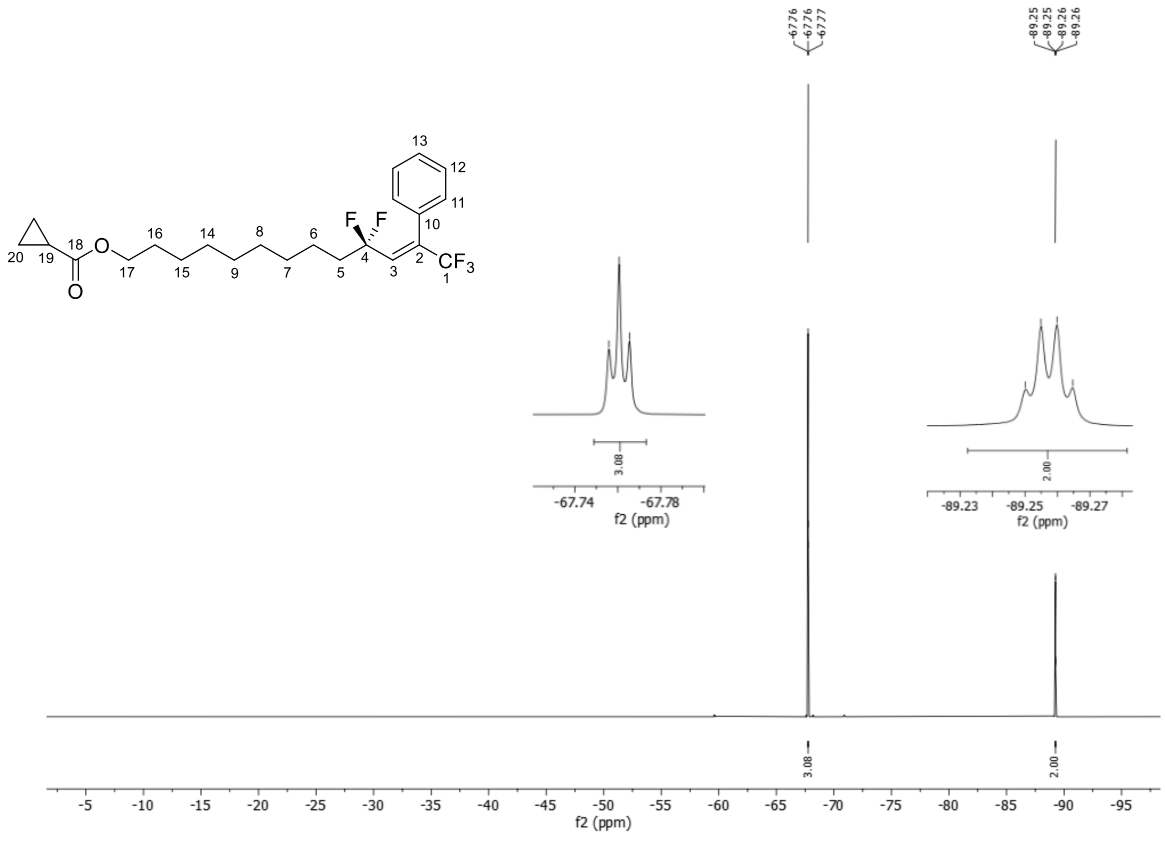

Figure S149. $^{19}F\{^1H\}$ NMR of **12** (470 MHz, 299 K, $CDCl_3$).

Diethyl 2-(2,2-difluoro-2-(4-(trifluoromethyl)phenyl)ethylidene)malonate (13)

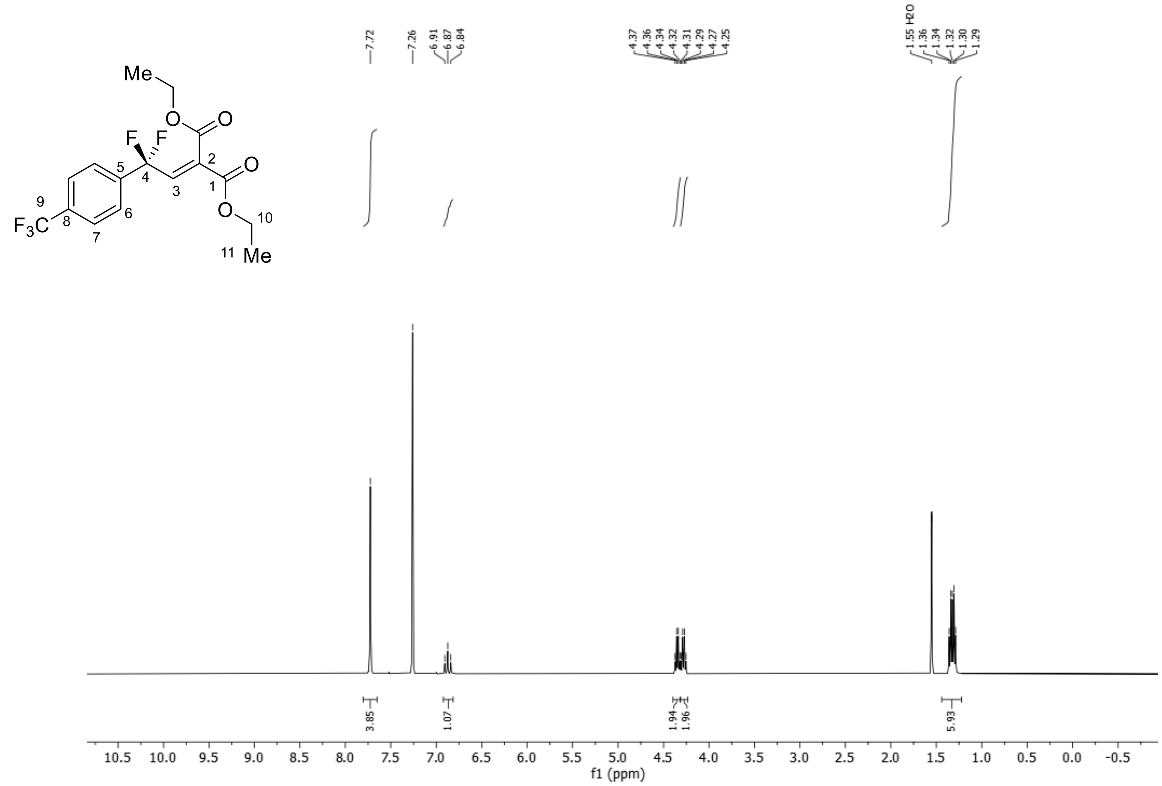

Figure S150. ^{1}H NMR of **13** (400 MHz, 299 K, $CDCl_3$).

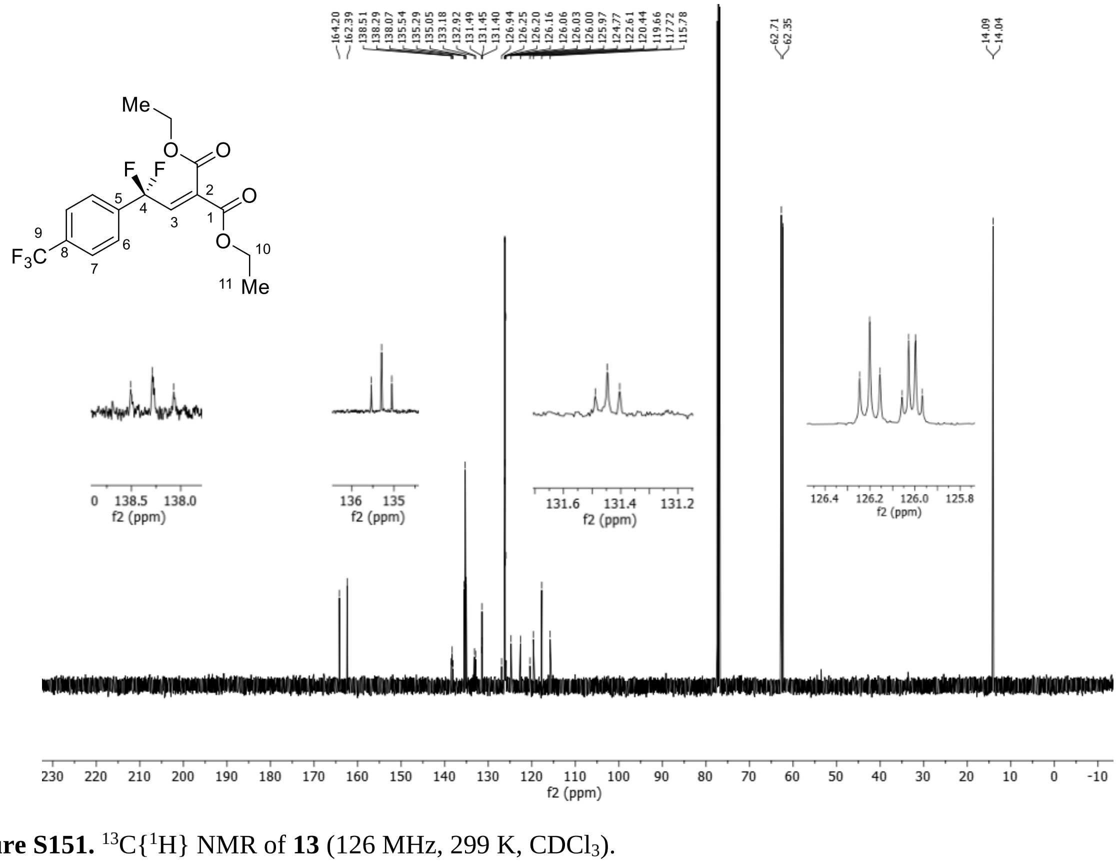

Figure S151. $^{13}C\{^{1}H\}$ NMR of **13** (126 MHz, 299 K, $CDCl_3$).

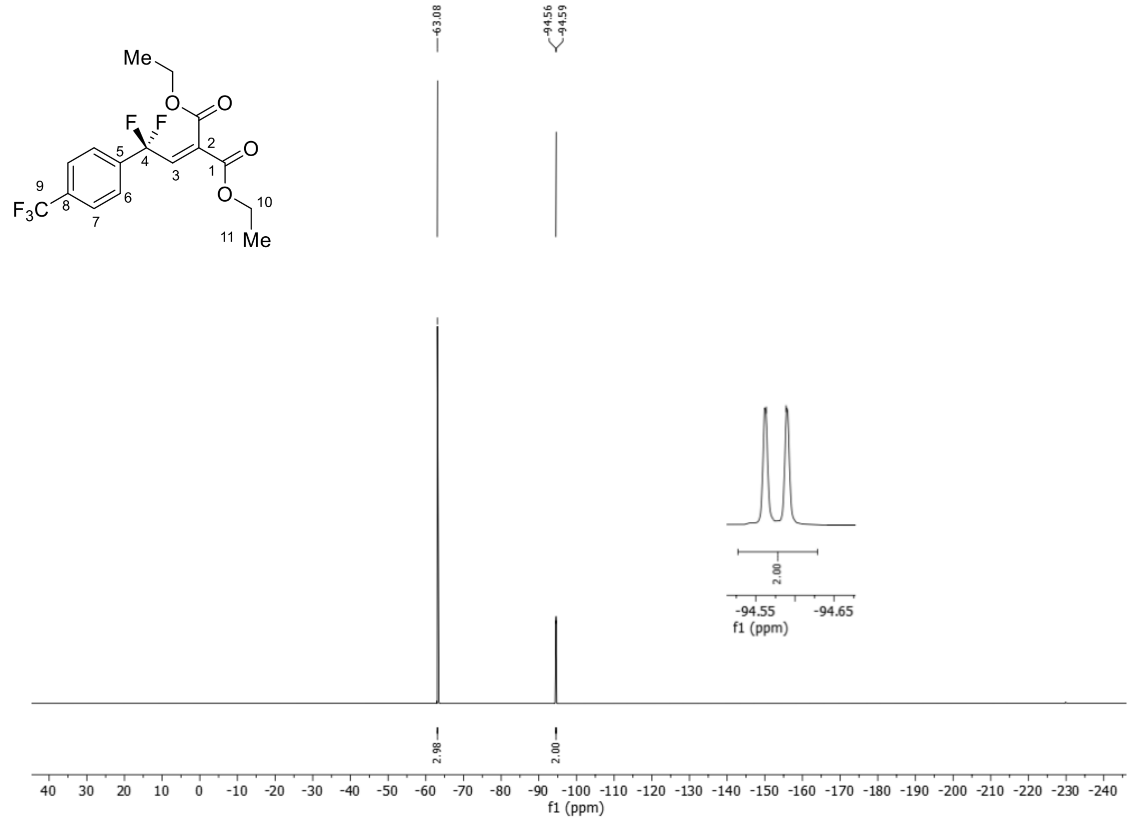

Figure S152. ^{19}F NMR of **13** (470 MHz, 299 K, $CDCl_3$).

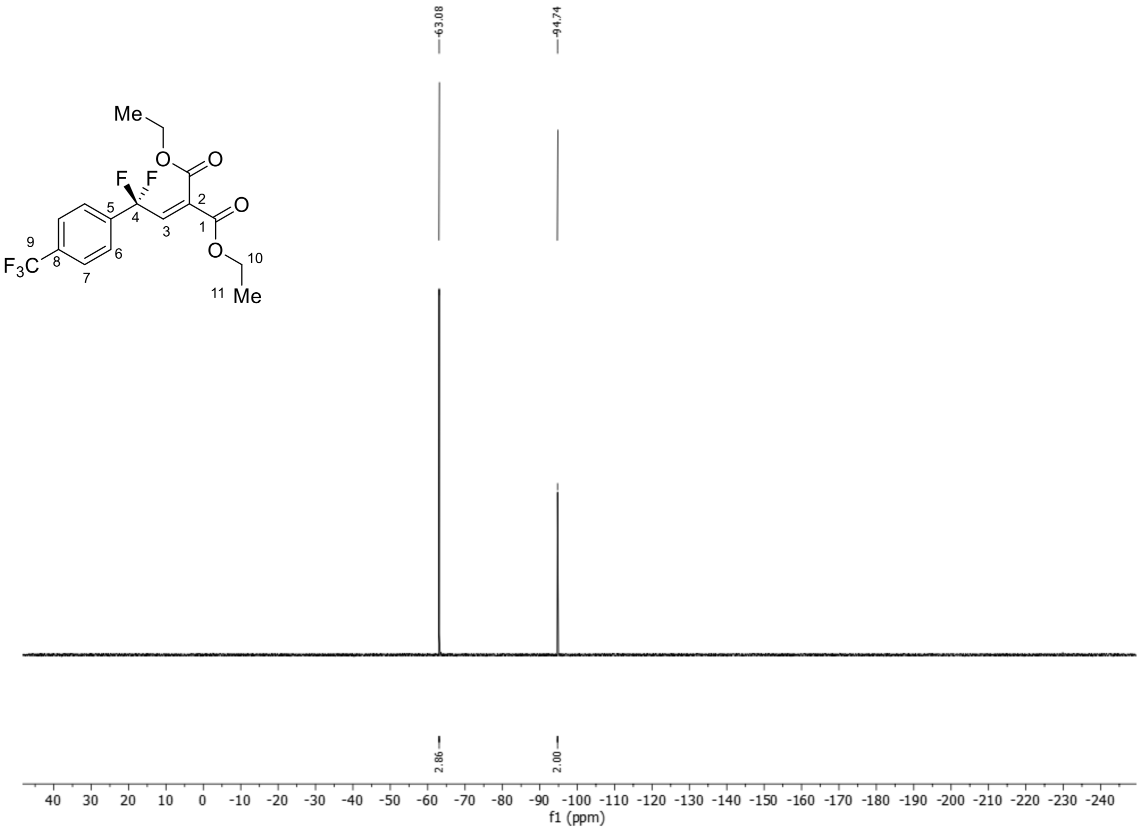

Figure S153. $^{19}F\{^1H\}$ NMR of **13** (377 MHz, 299 K, $CDCl_3$).

Diethyl 2-(2-(3,5-bis(trifluoromethyl)phenyl)-2,2-difluoroethylidene)malonate (14)

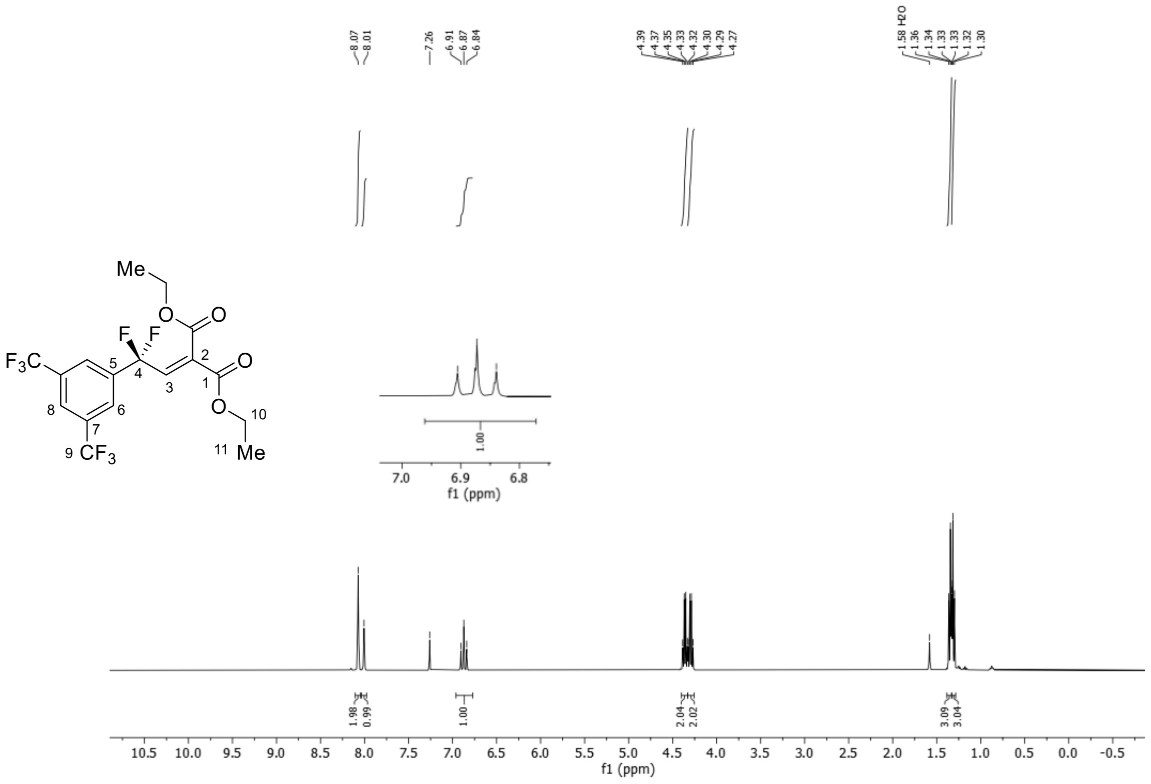

Figure S154. ^{1}H NMR of **14** (400 MHz, 299 K, $CDCl_3$).

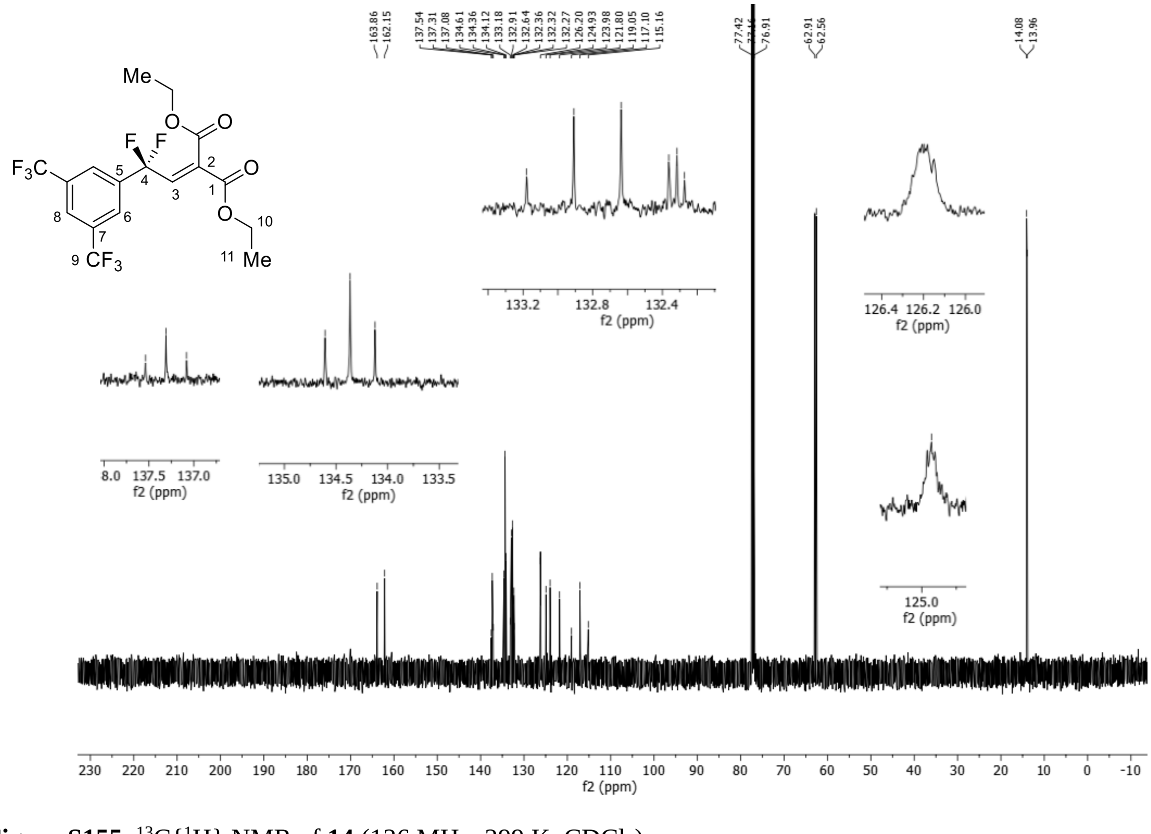

Figure S155. $^{13}C\{^{1}H\}$ NMR of **14** (126 MHz, 299 K, $CDCl_3$).

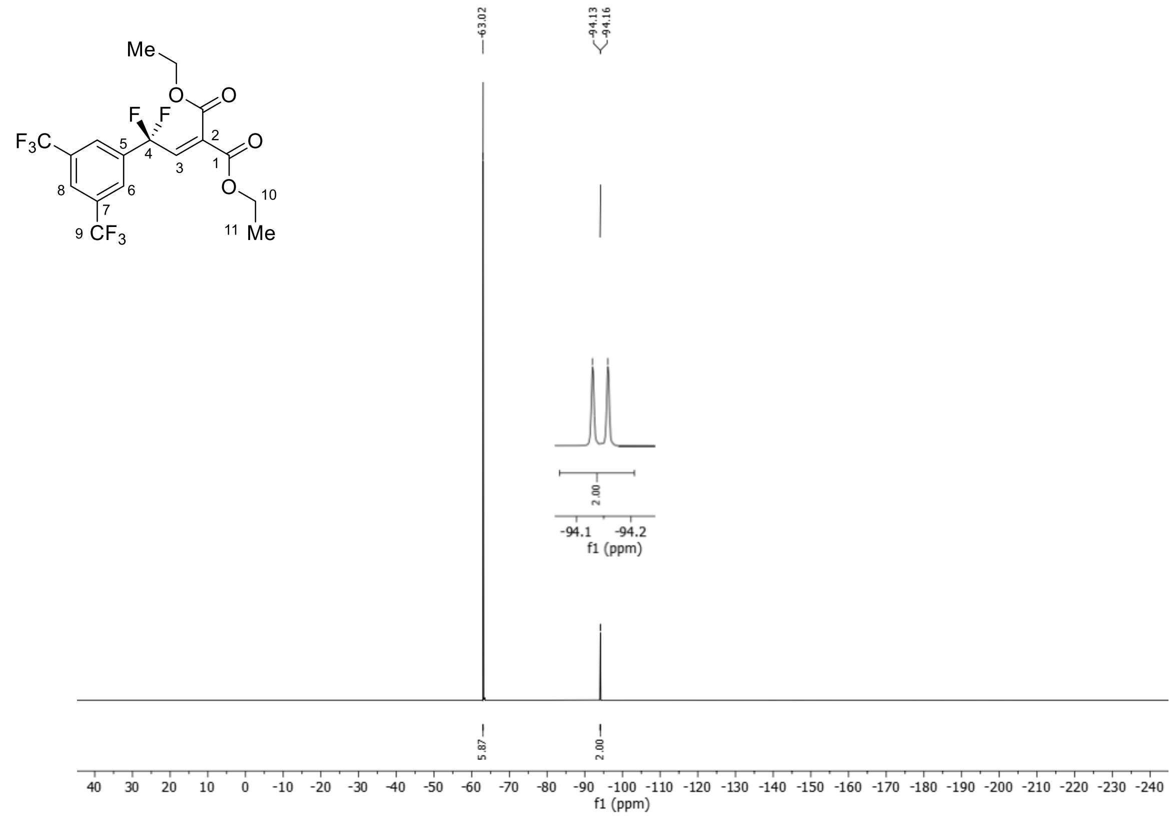

Figure S156. ^{19}F NMR of **14** (470 MHz, 299 K, $CDCl_3$).

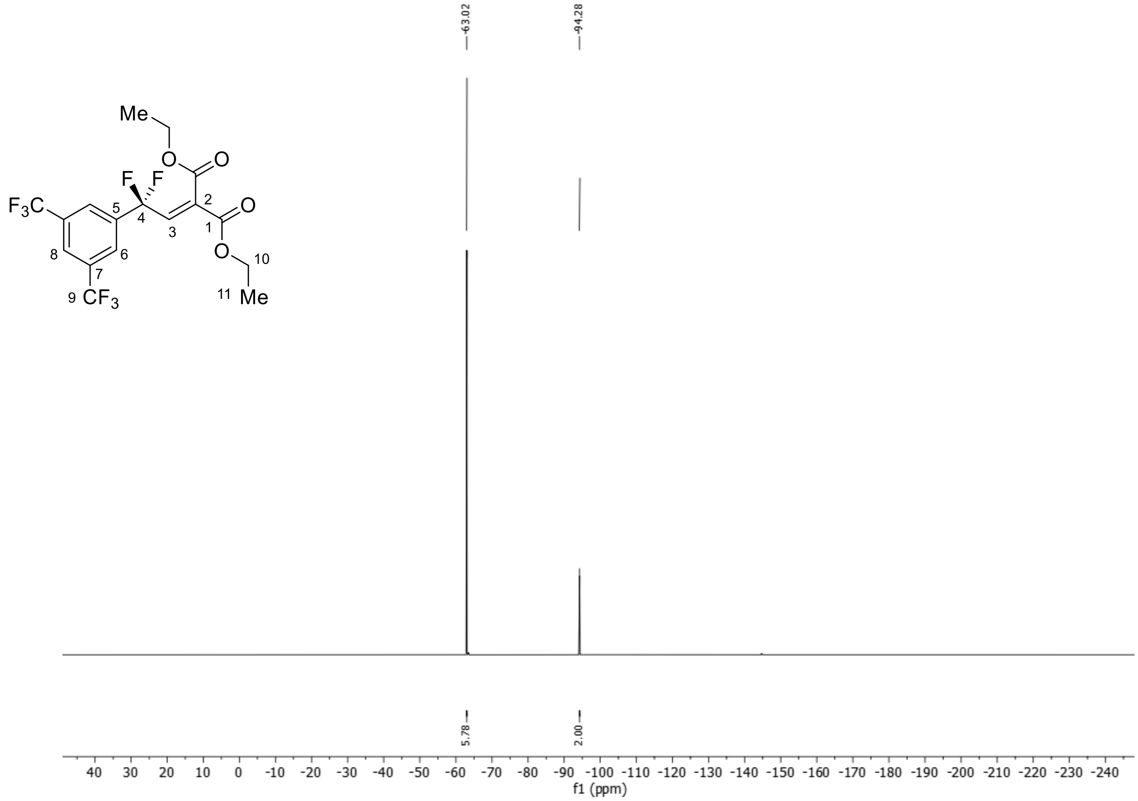

Figure S157. $^{19}F\{^1H\}$ NMR of **14** (377 MHz, 299 K, $CDCl_3$).

Diethyl 2-(2,2-difluoro-2-(4-nitrophenyl)ethylidene)malonate (15)

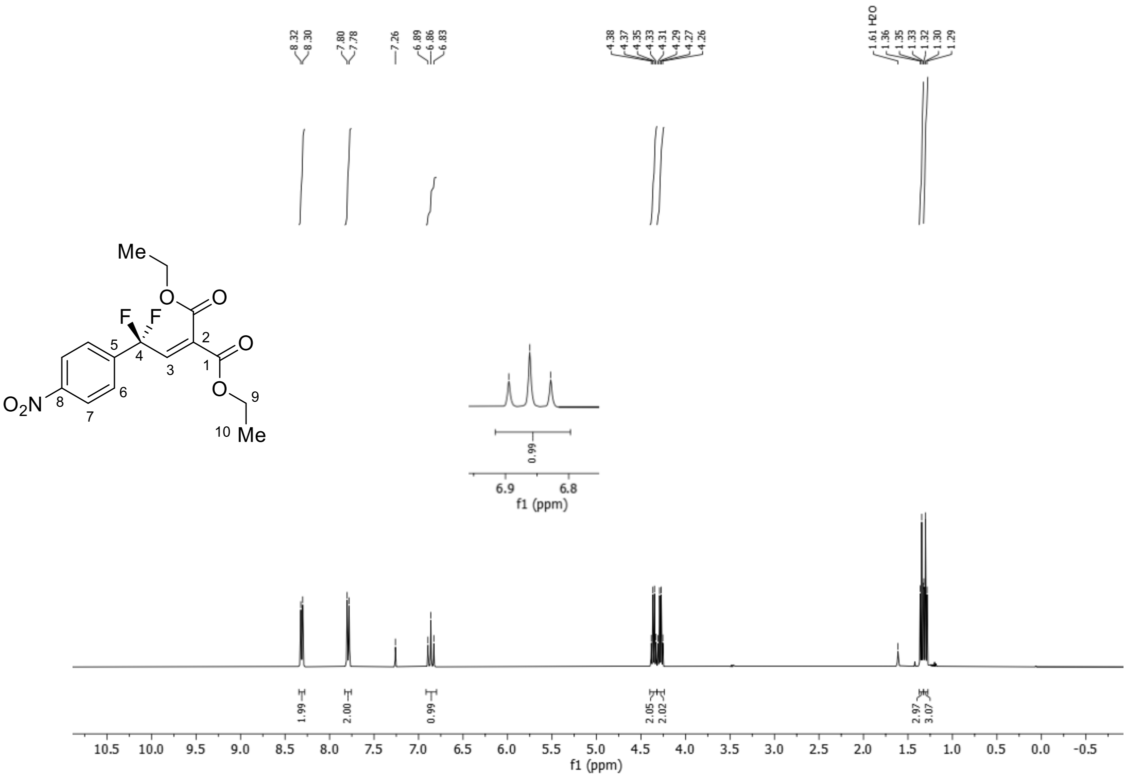

Figure S158. ^{1}H NMR of **15** (400 MHz, 299 K, $CDCl_3$).

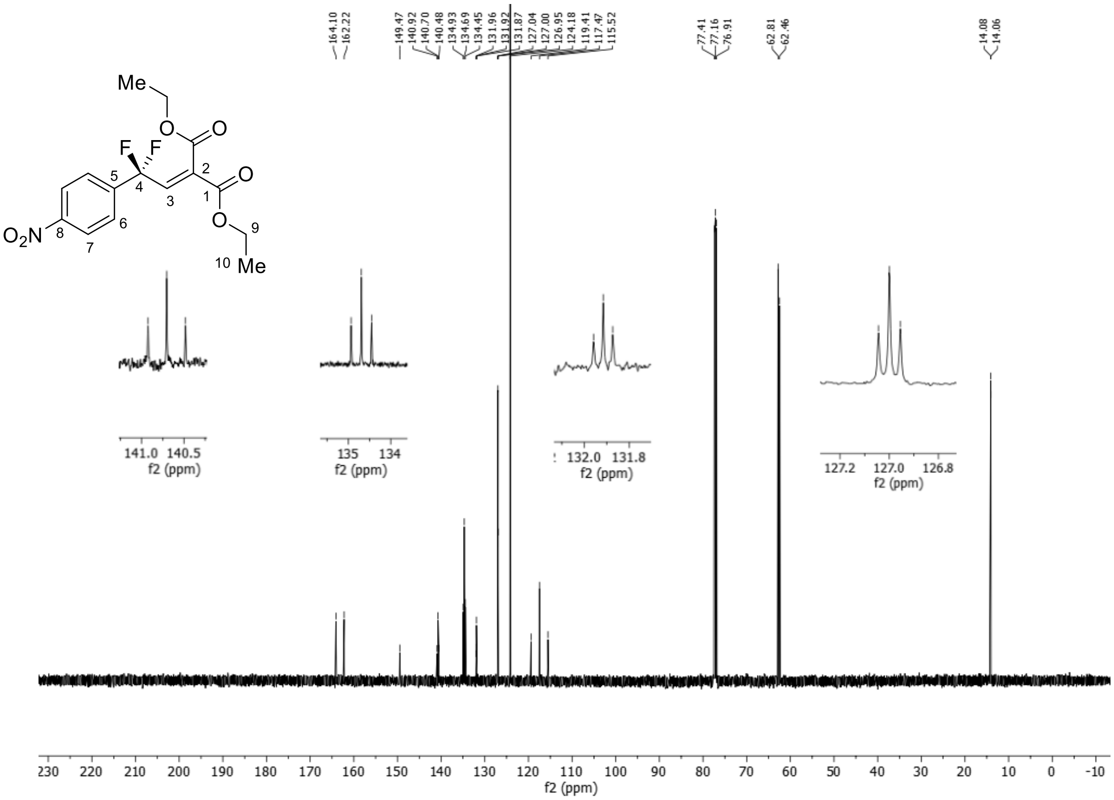

Figure S159. $^{13}C\{^{1}H\}$ NMR of **15** (126 MHz, 299 K, $CDCl_3$).

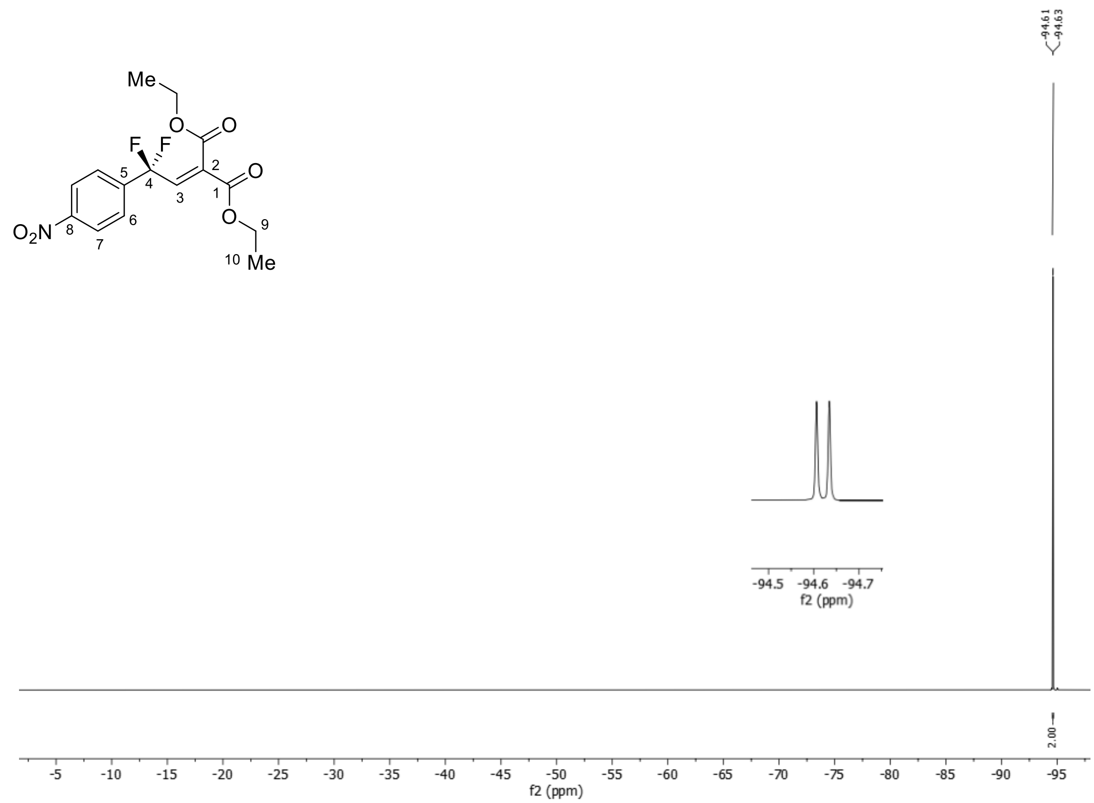

Figure S160. ^{19}F NMR of **15** (470 MHz, 299 K, $CDCl_3$).

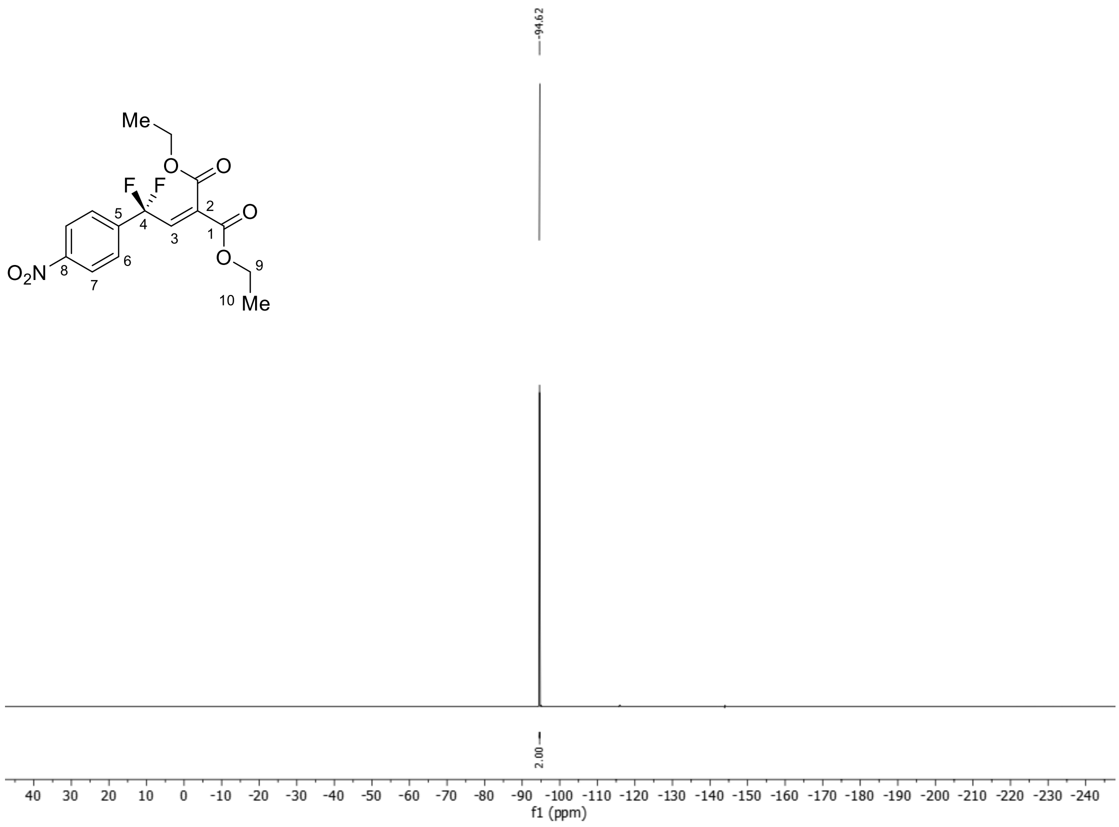

Figure S161. ^{19}F{^{1}H} NMR of **15** (377 MHz, 299 K, $CDCl_3$).

Diethyl 2-(2-(4-bromophenyl)-2,2-difluoroethylidene)malonate (16)

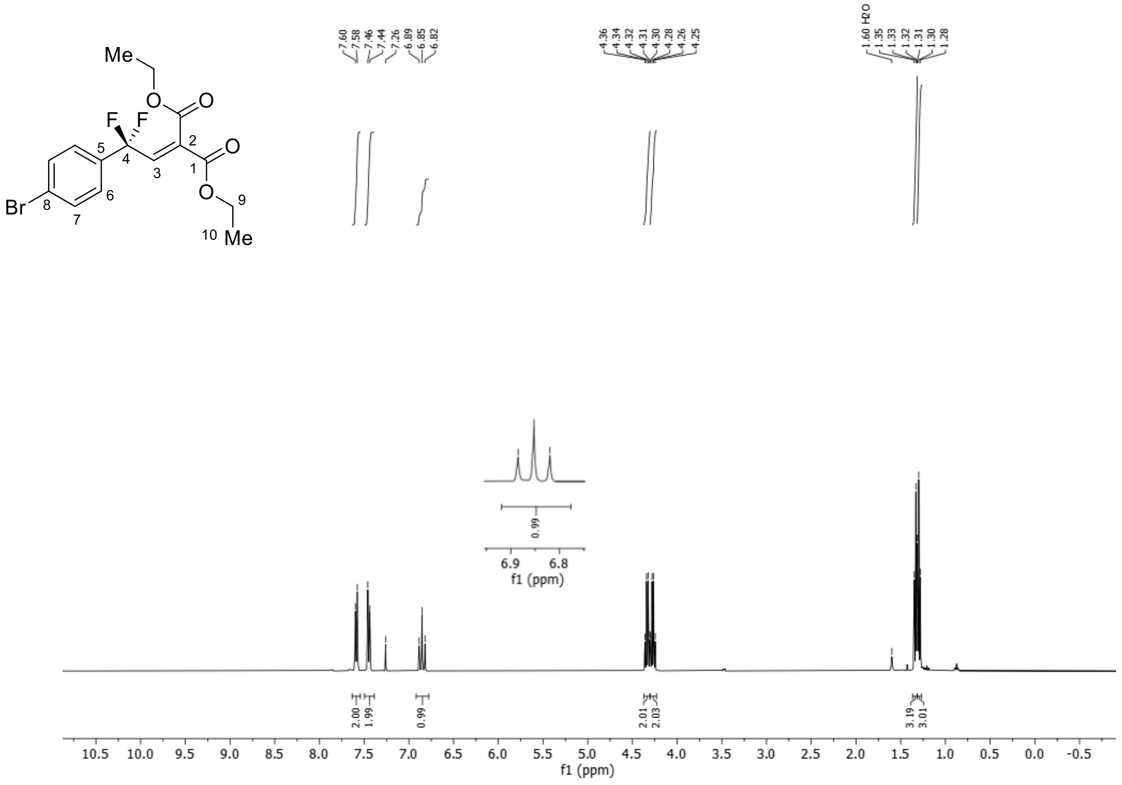

Figure S162. ^{1}H NMR of **16** (400 MHz, 299 K, $CDCl_3$).

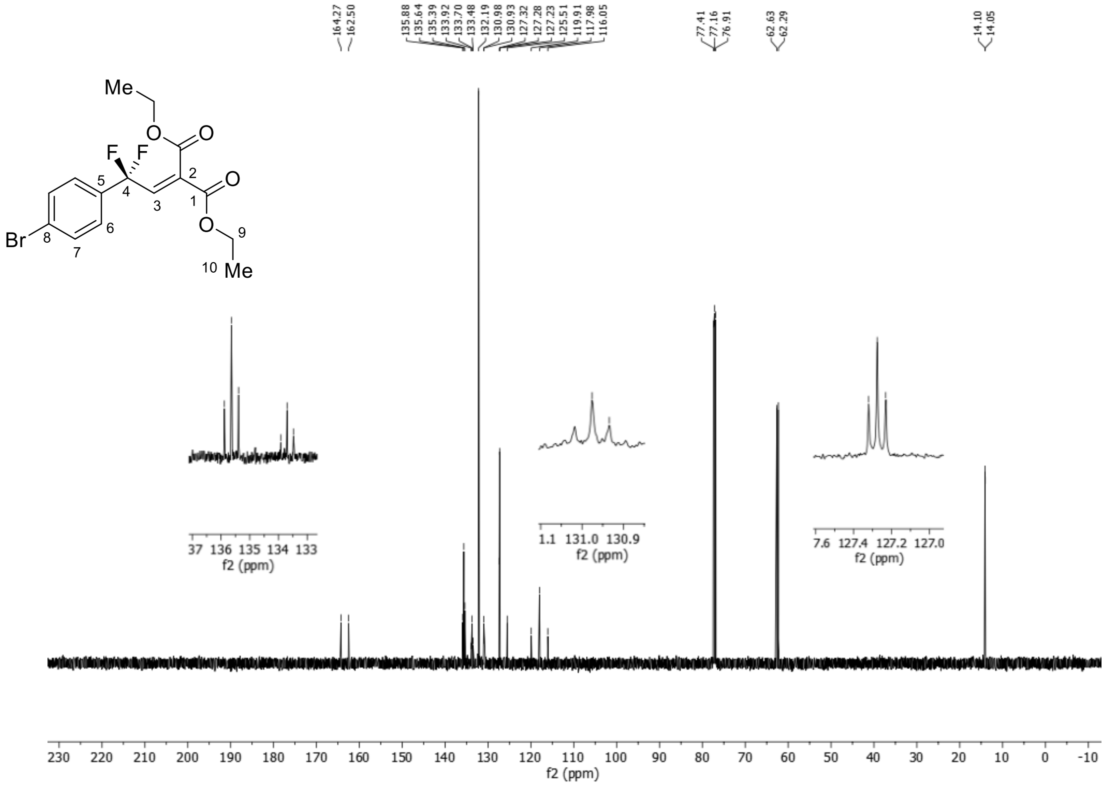

Figure S163. $^{13}C\{^{1}H\}$ NMR of **16** (126 MHz, 299 K, $CDCl_3$).

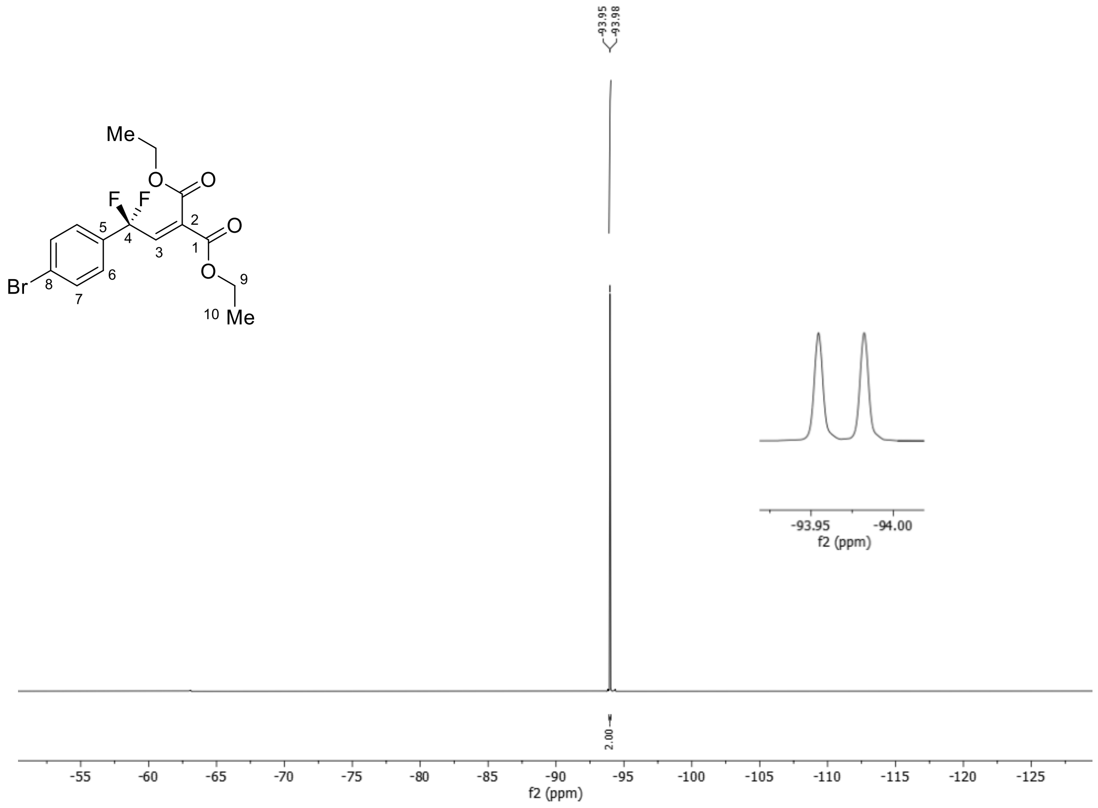

Figure S164. ^{19}F NMR of **16** (470 MHz, 299 K, $CDCl_3$).

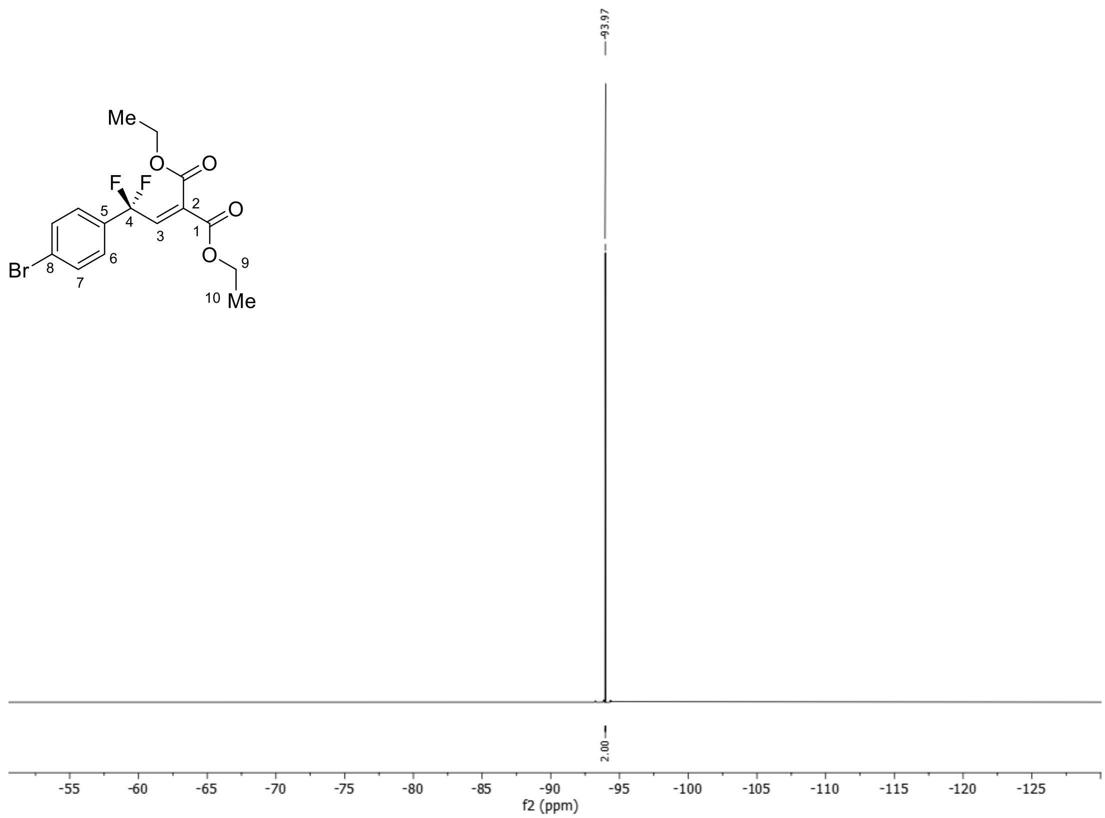

Figure S165. $^{19}F\{^{1}H\}$ NMR of **16** (470 MHz, 299 K, $CDCl_3$).

Dimethyl 2-(2,2-difluoro-2-(4-(trifluoromethyl)phenyl)ethylidene)malonate (17)

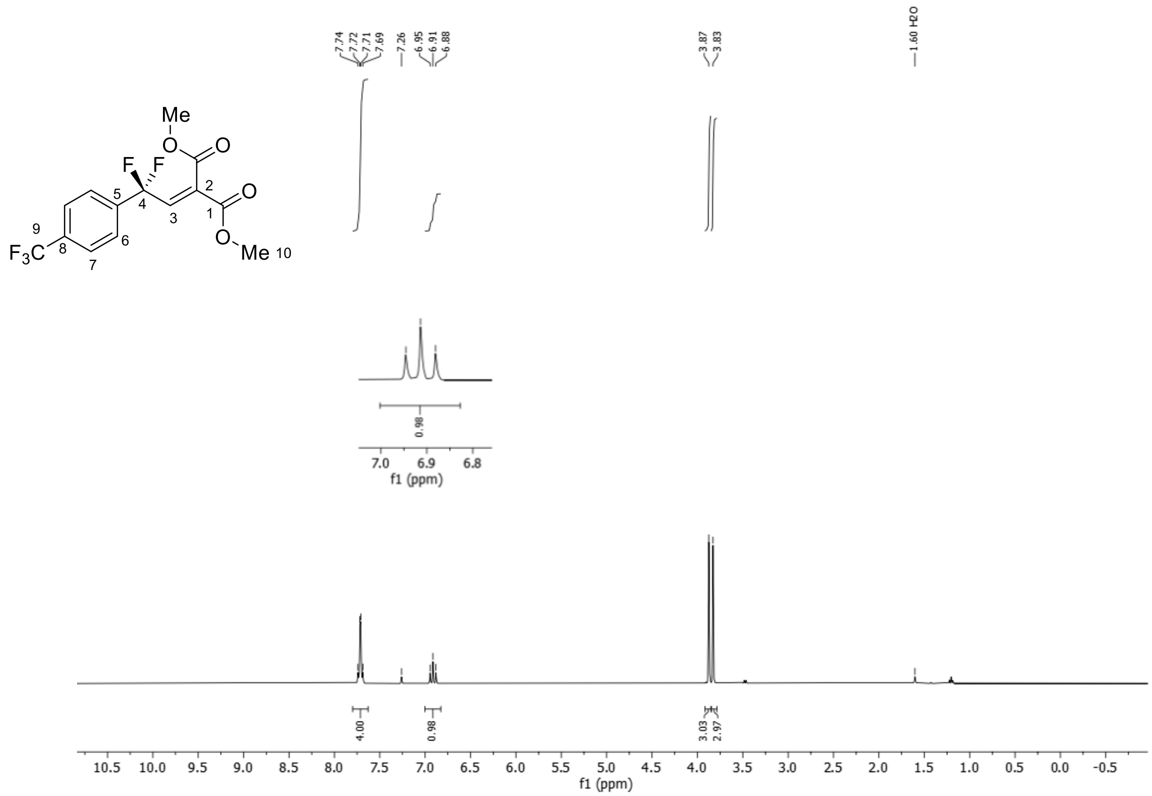

Figure S166. ^{1}H NMR of **17** (400 MHz, 299 K, $CDCl_3$).

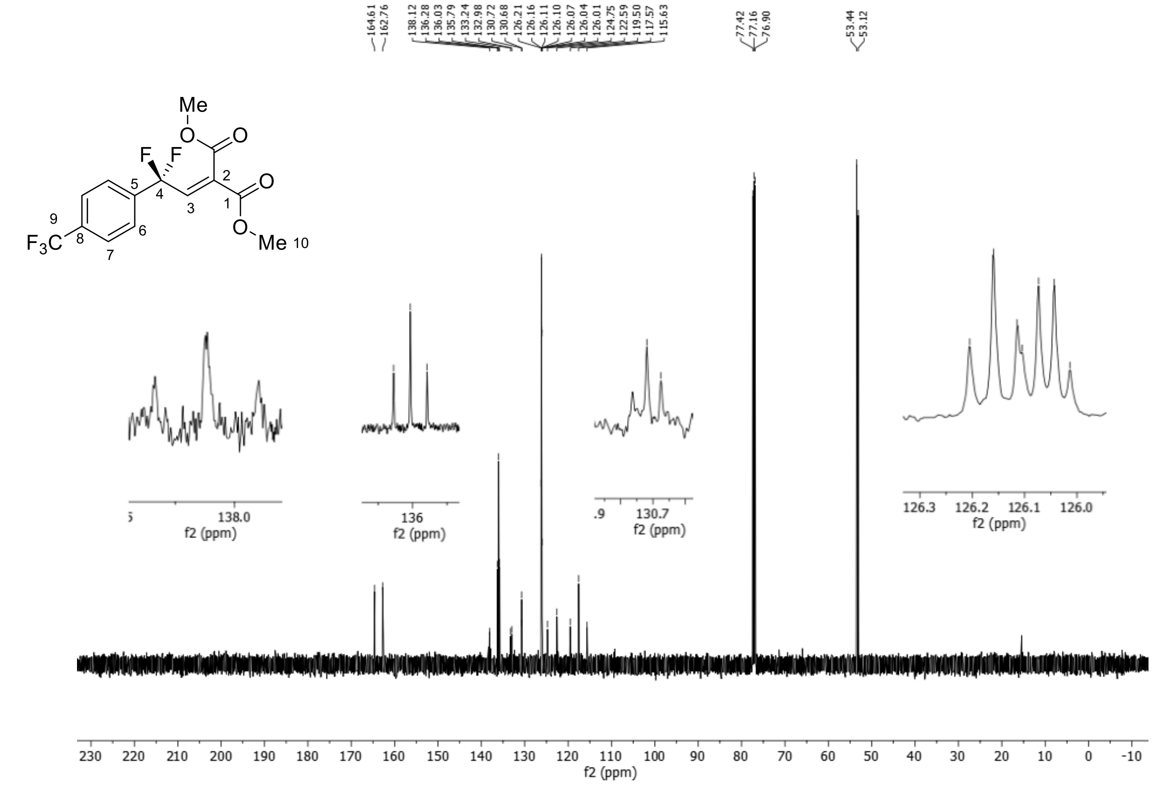

Figure S167. $^{13}C\{^{1}H\}$ NMR of **17** (126 MHz, 299 K, $CDCl_3$).

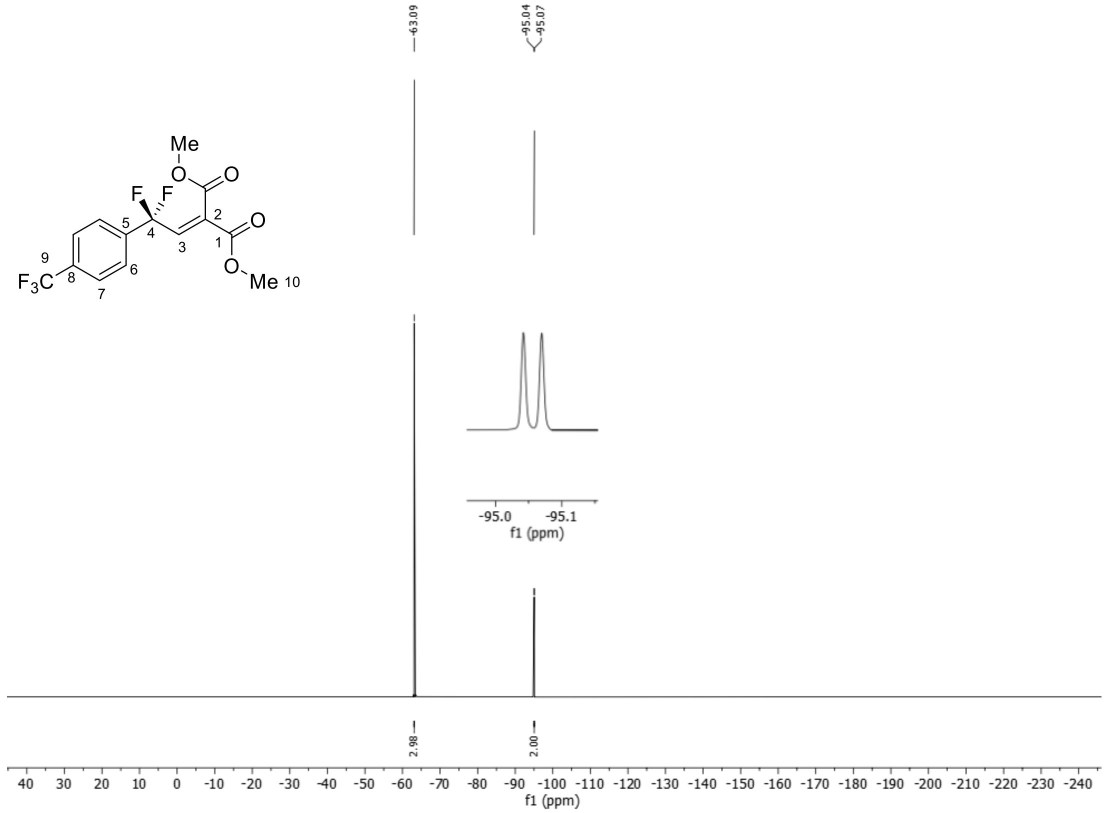

Figure S168. ^{19}F NMR of **17** (470 MHz, 299 K, $CDCl_3$).

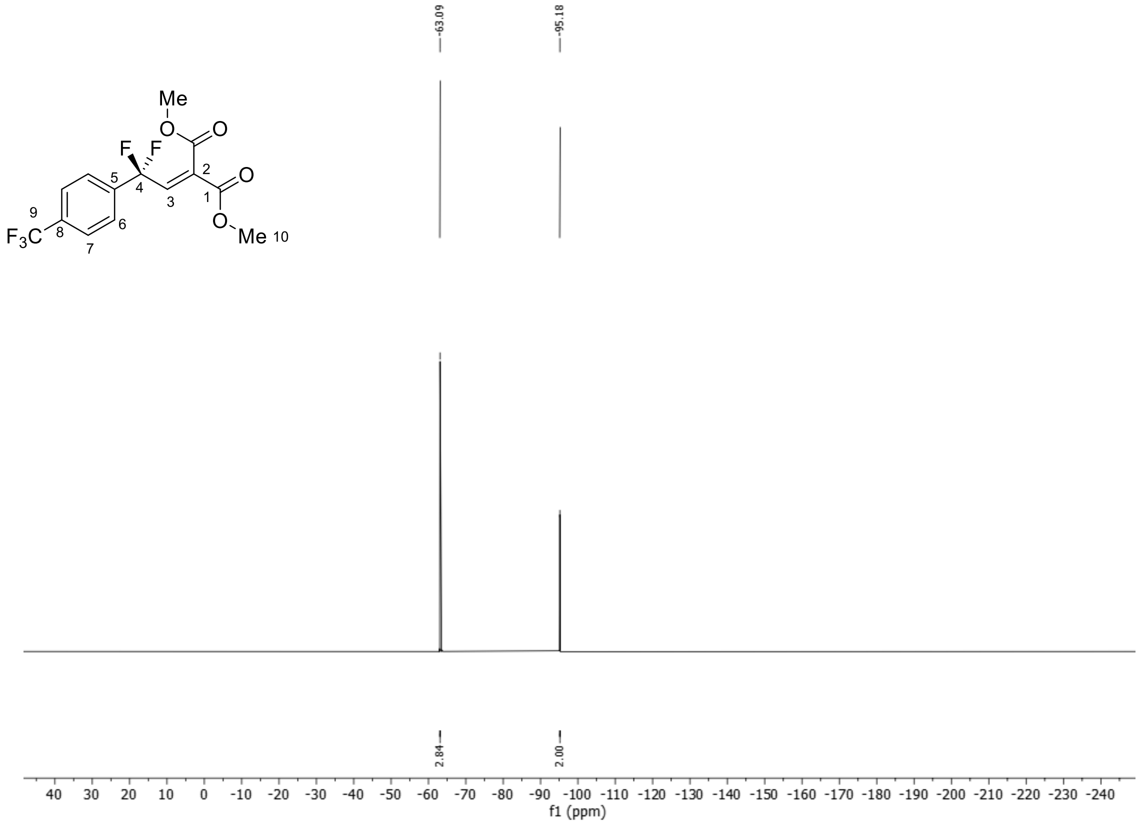

Figure S169. $^{19}F\{^{1}H\}$ NMR of **17** (377 MHz, 299 K, $CDCl_3$).

Ethyl (*Z*)-4,4-difluoro-2-methyl-4-(4-(trifluoromethyl)phenyl)but-2-enoate (18)

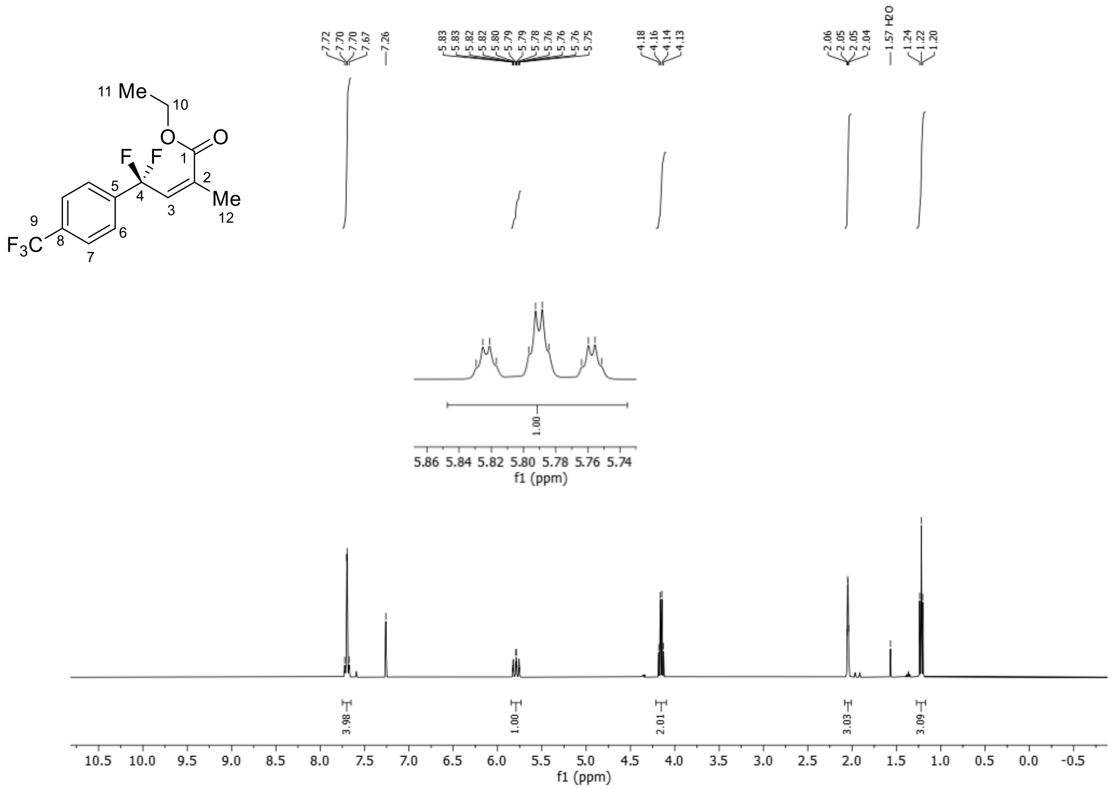

Figure S170. ^{1}H NMR of **18** (400 MHz, 299 K, $CDCl_3$).

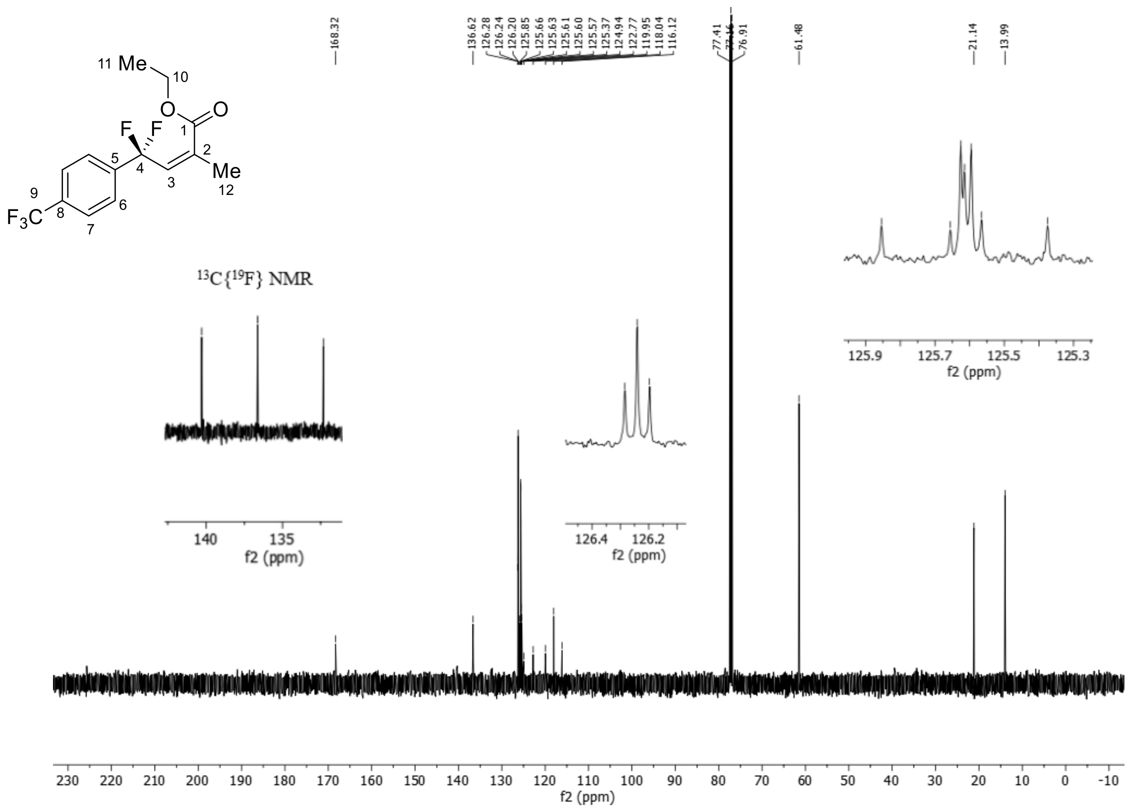

Figure S171. $^{13}C\{^{1}H\}$ NMR of **18** (126 MHz, 299 K, $CDCl_3$).

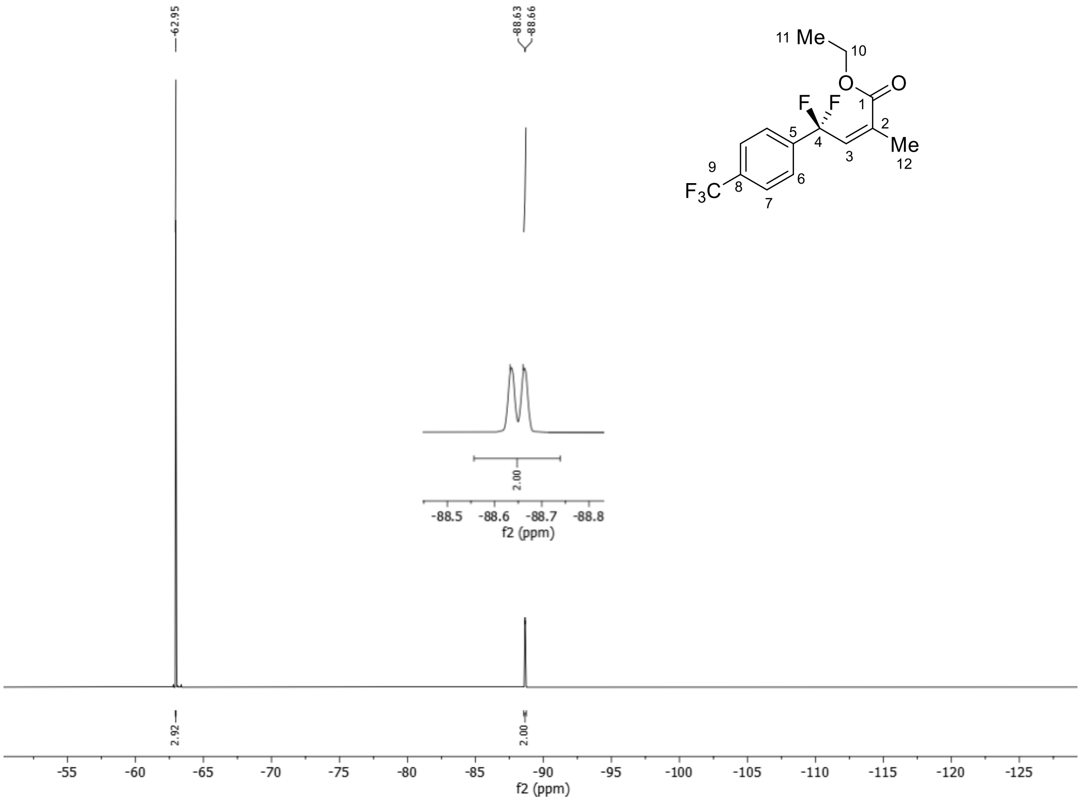

Figure S172. ^{19}F NMR of **18** (470 MHz, 299 K, $CDCl_3$).

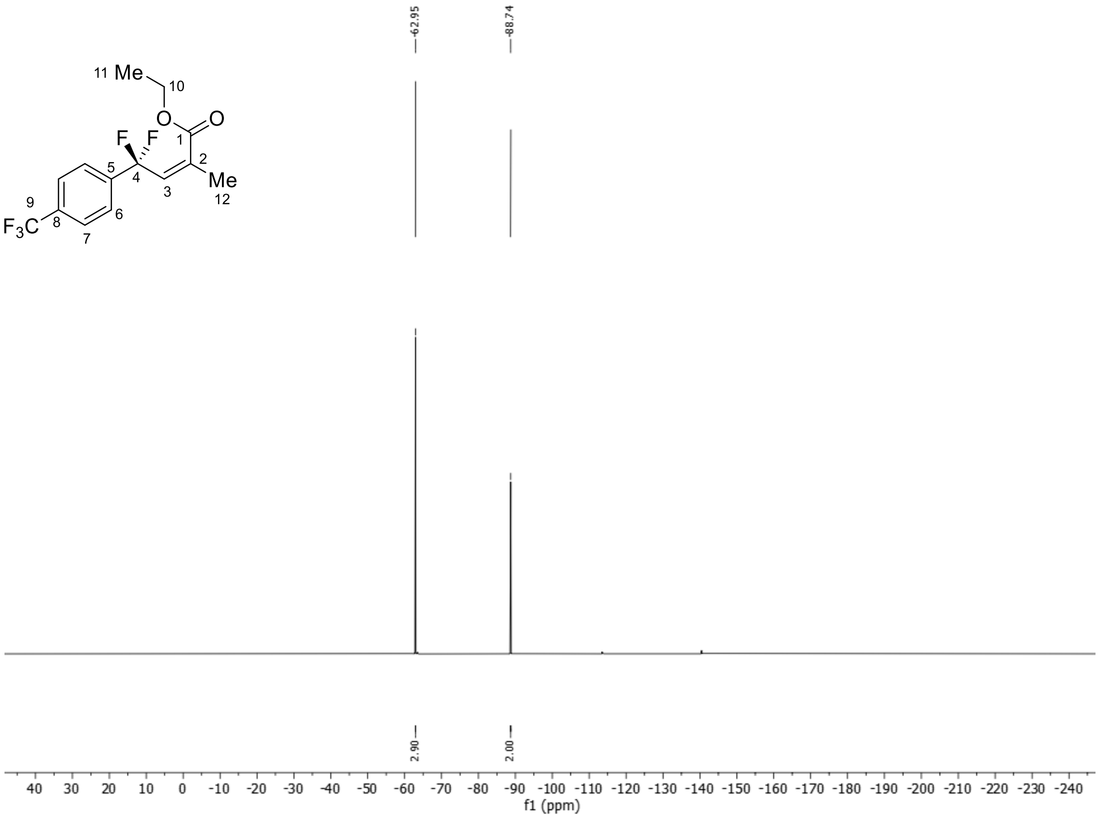

Figure S173. $^{19}F\{^1H\}$ NMR of **18** (377 MHz, 299 K, $CDCl_3$).

Ethyl (*Z*)-4,4-difluoro-2-methyldec-2-enoate (19)

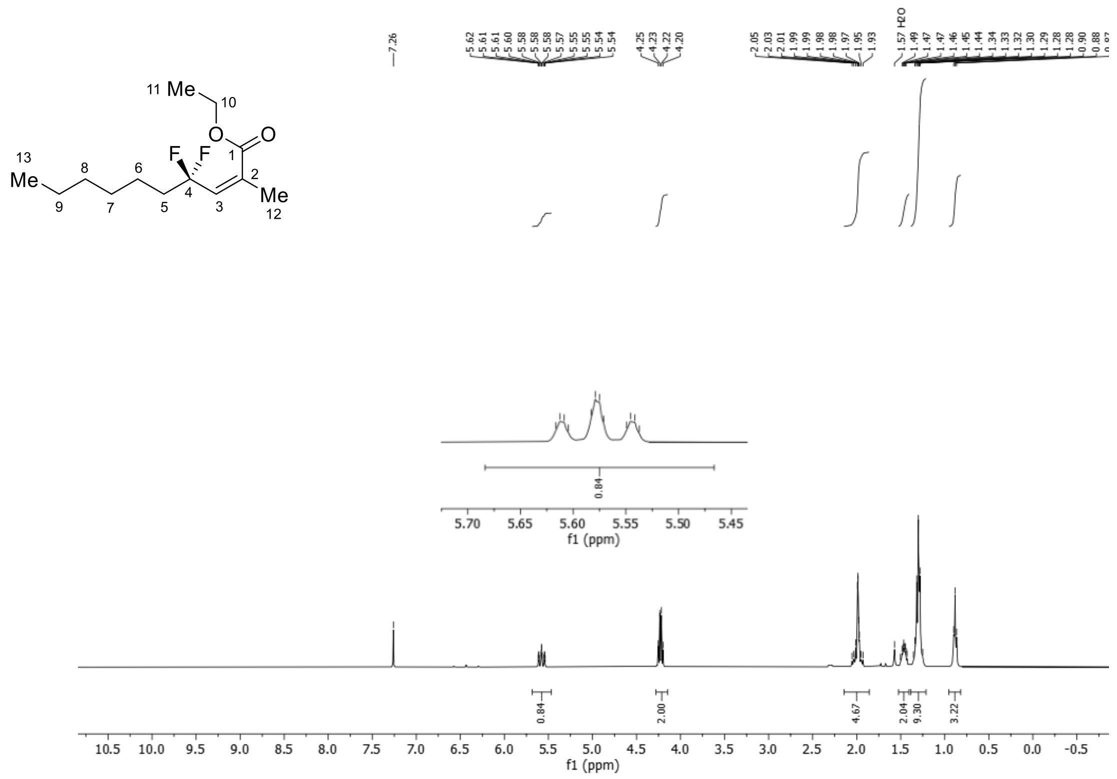

Figure S174. ^{1}H NMR of **19** (400 MHz, 299 K, $CDCl_3$).

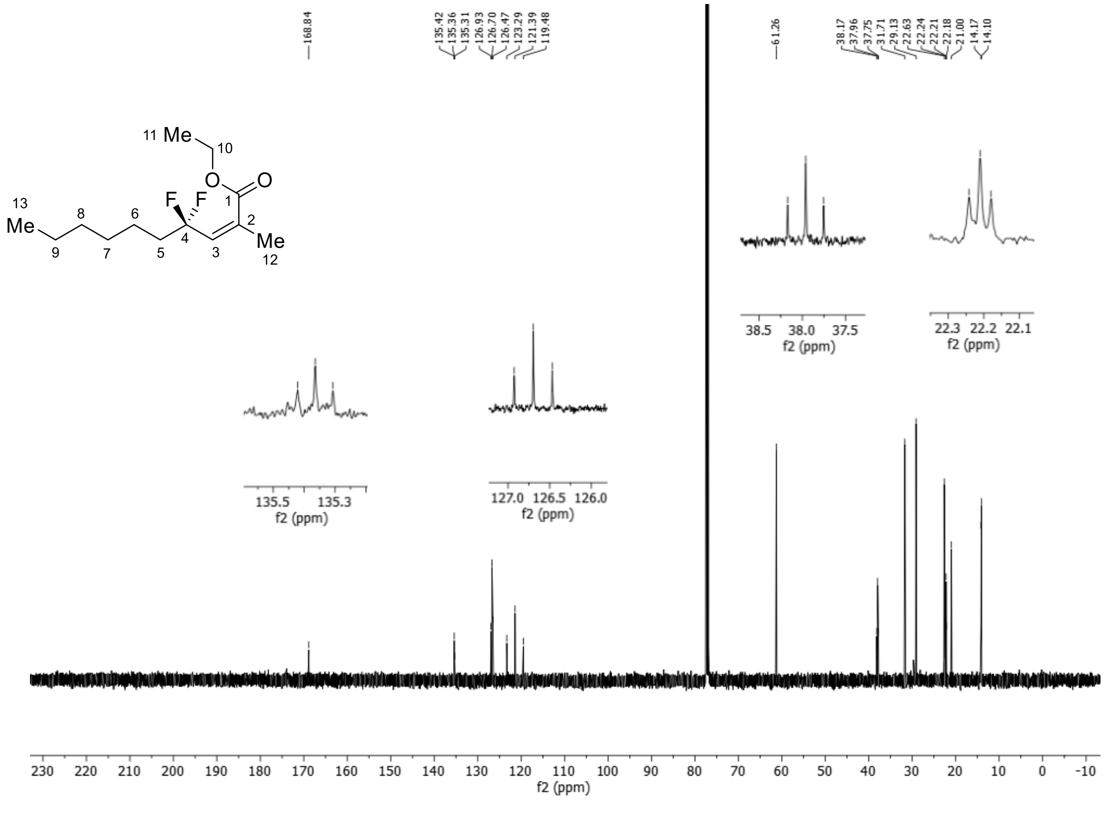

Figure S175. $^{13}C\{^{1}H\}$ NMR of **19** (126 MHz, 299 K, $CDCl_3$).

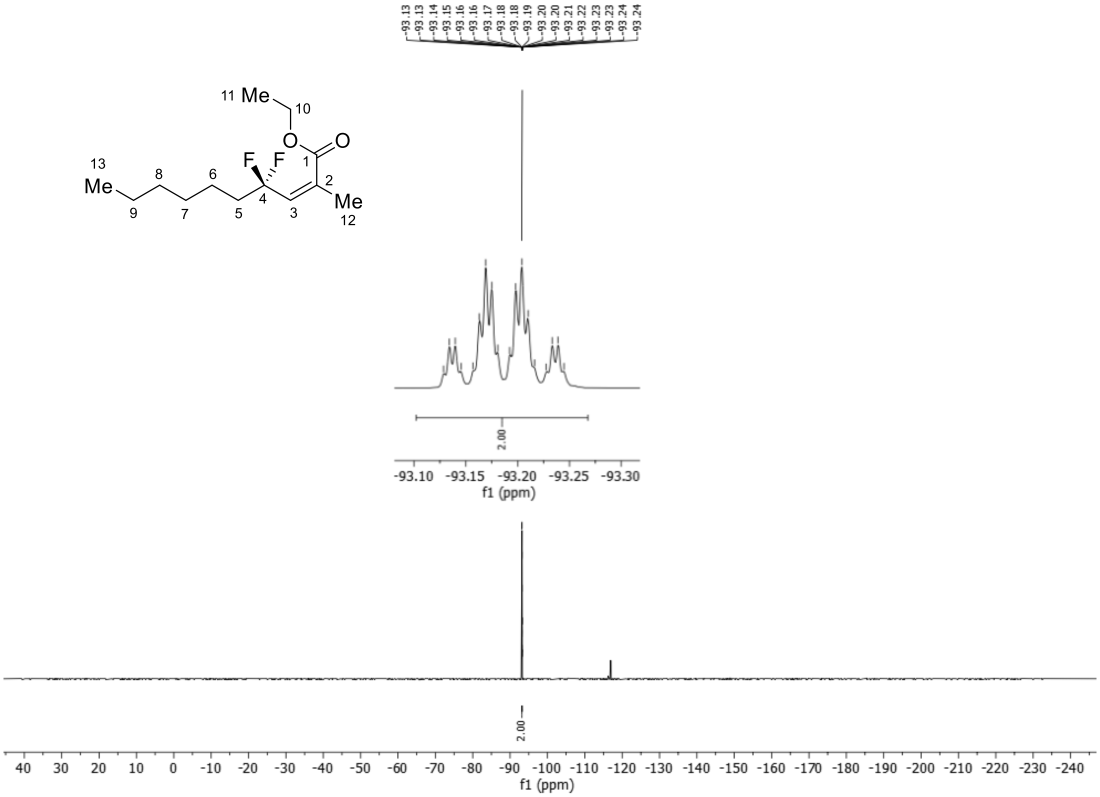

Figure S176. ^{19}F NMR of **19** (470 MHz, 299 K, $CDCl_3$).

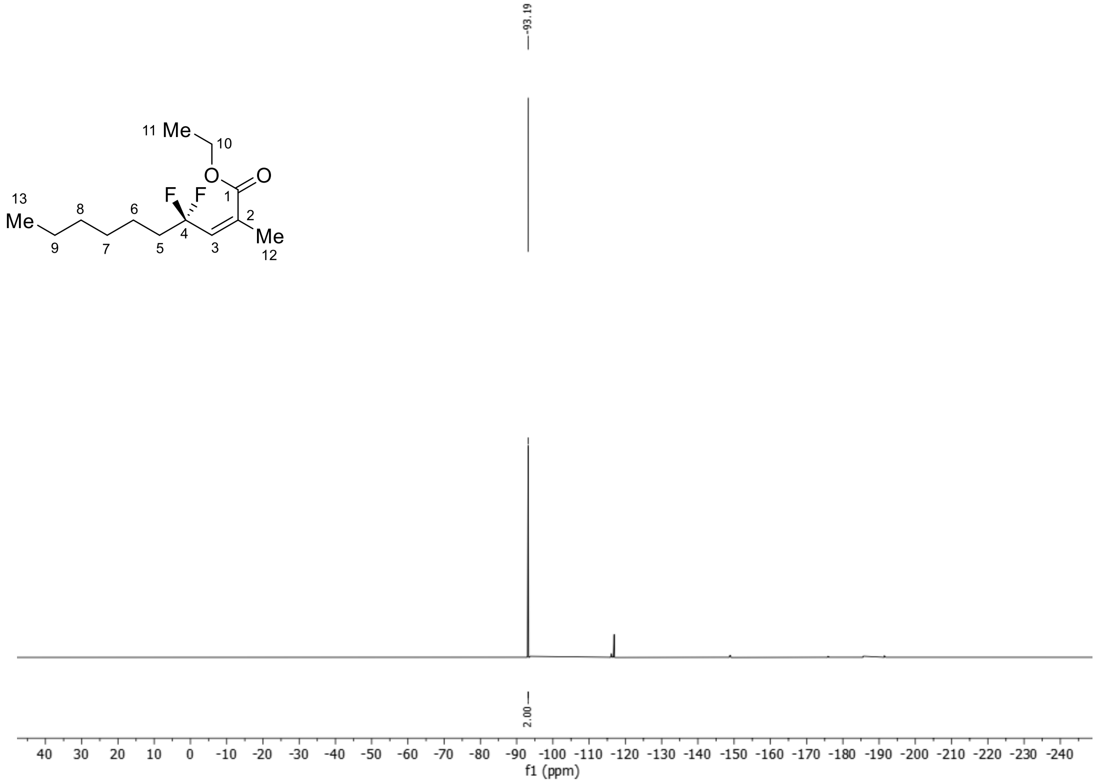

Figure S177. $^{19}F\{^1H\}$ NMR of **19** (377 MHz, 299 K, $CDCl_3$).

Ethyl (*Z*)-4,4-difluoro-2-phenyl-4-(4-(trifluoromethyl)phenyl)but-2-enoate (20)

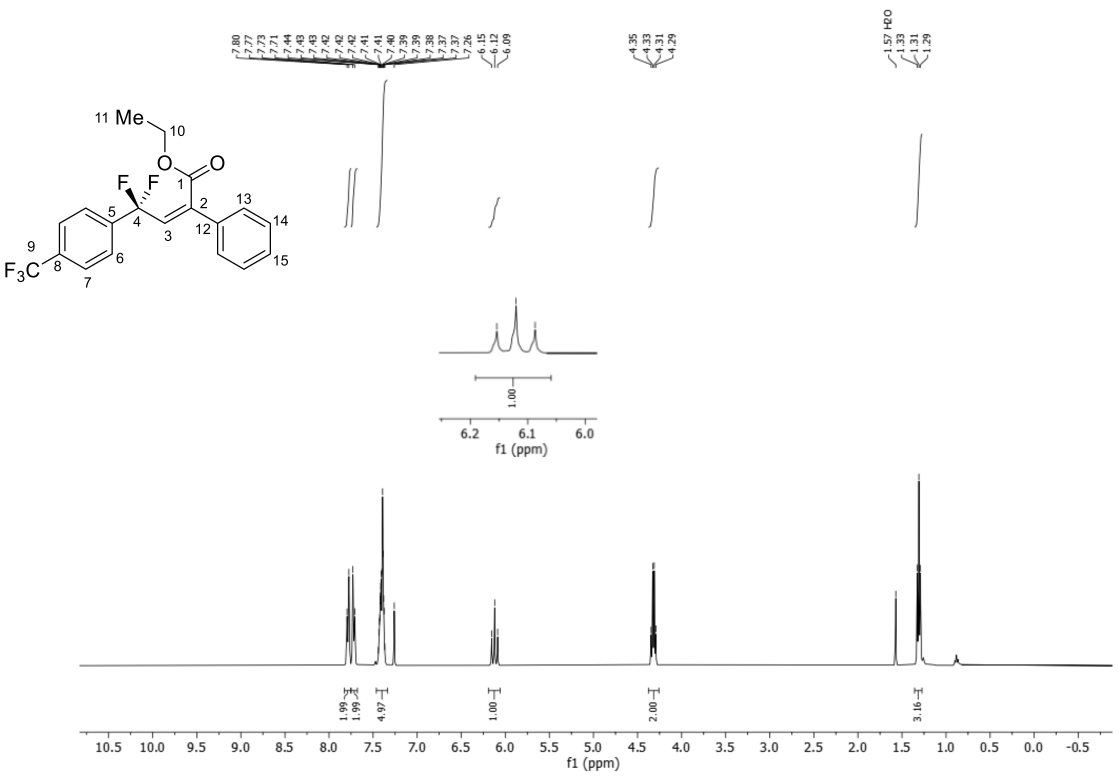

Figure S178. ^{1}H NMR of **20** (400 MHz, 299 K, $CDCl_3$).

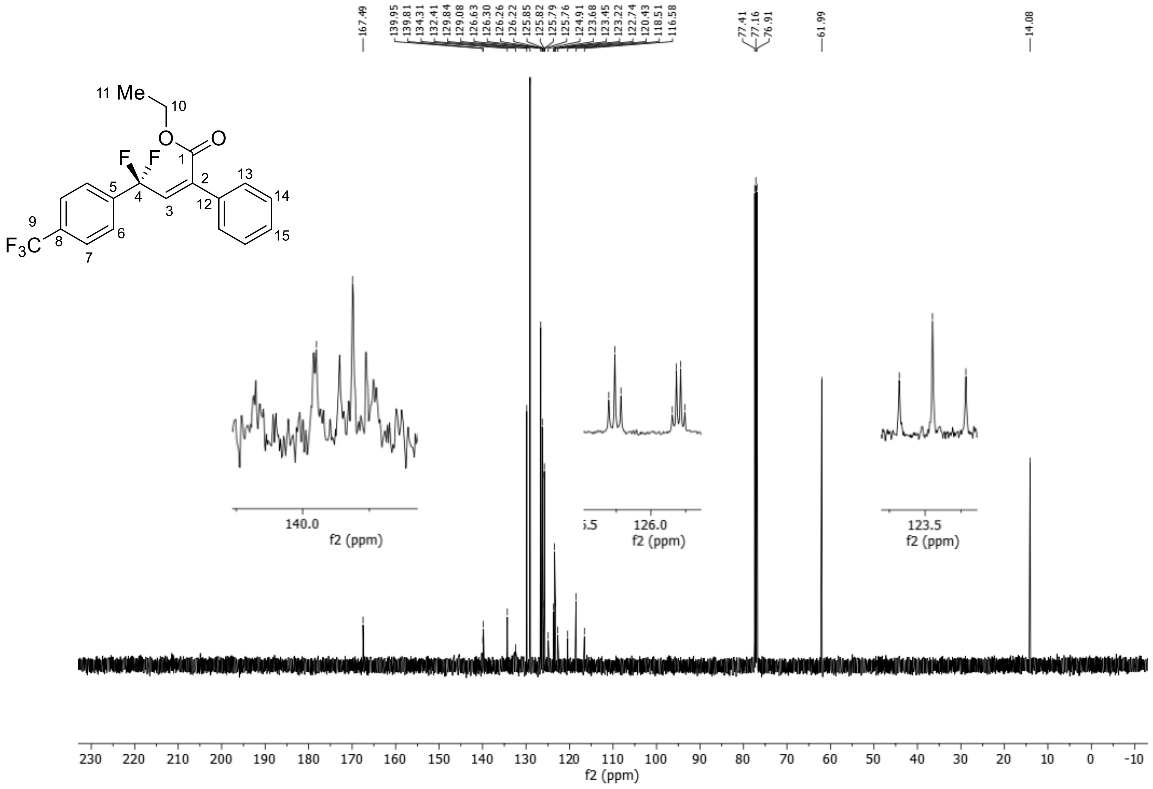

Figure S179. $^{13}C\{^{1}H\}$ NMR of **20** (126 MHz, 299 K, $CDCl_3$).

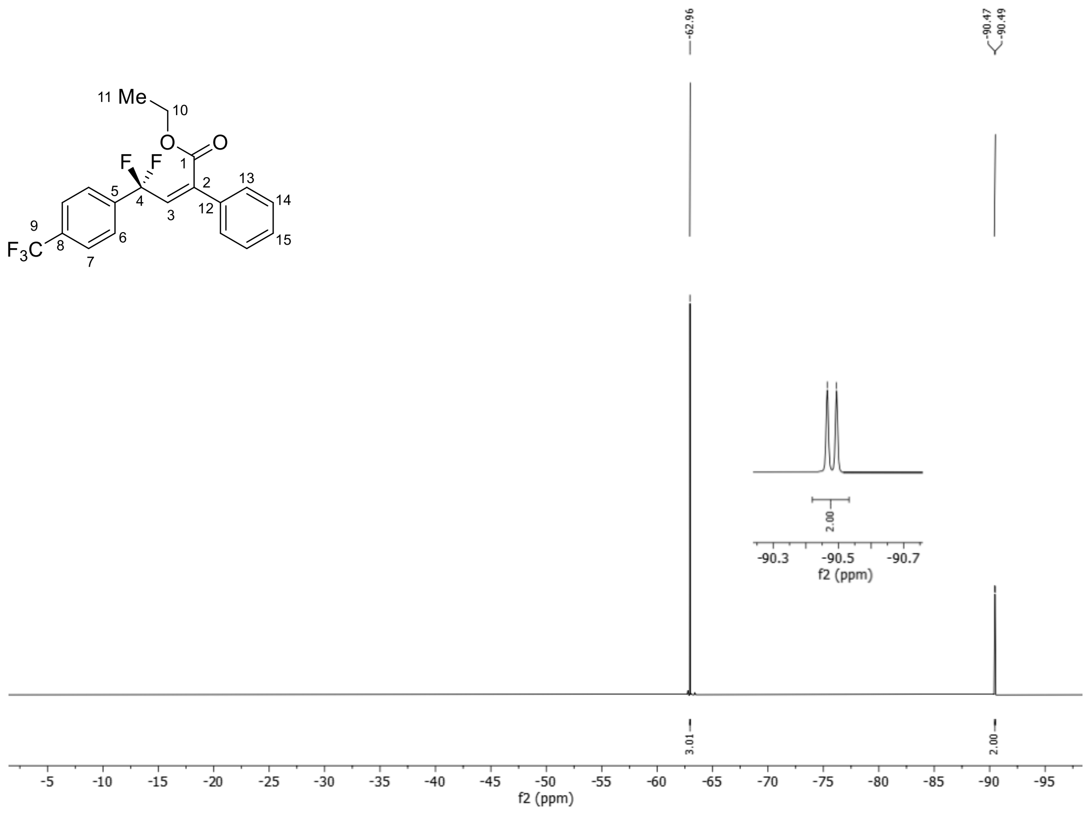

Figure S180. ^{19}F NMR of **20** (470 MHz, 299 K, $CDCl_3$).

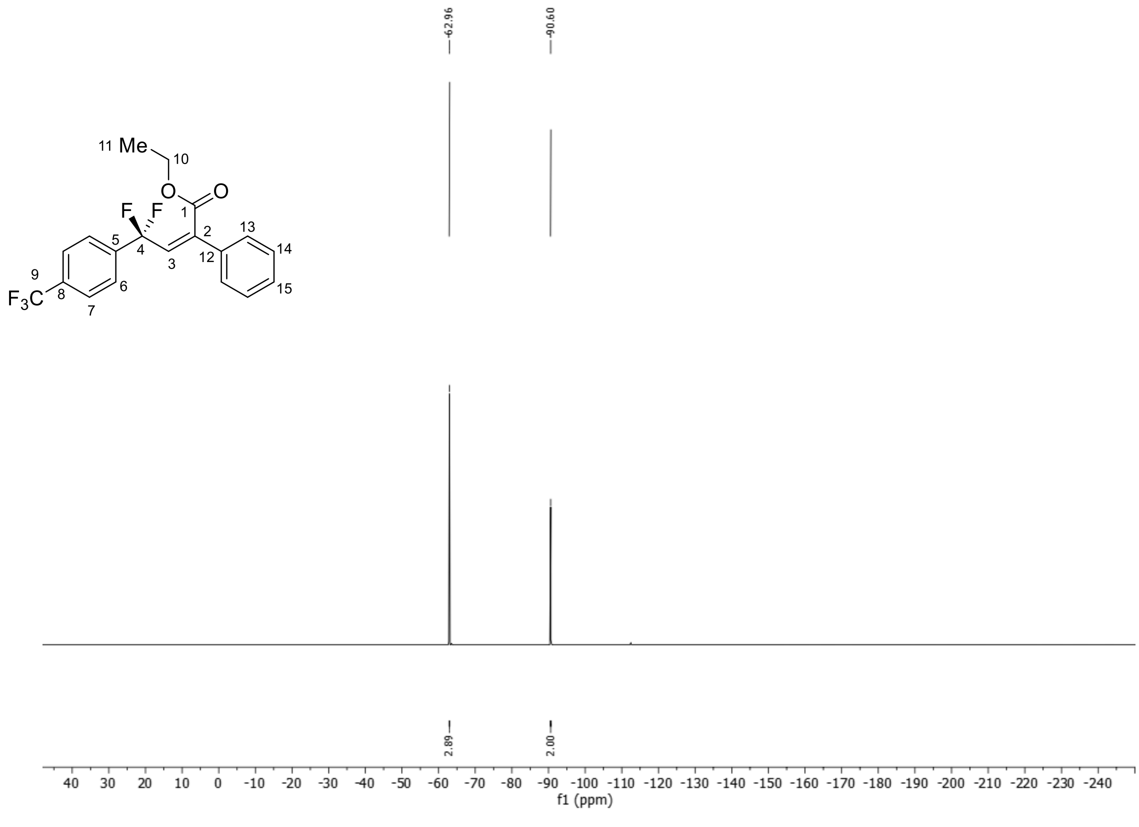

Figure S181. ^{19}F{^{1}H} NMR of **20** (377 MHz, 299 K, $CDCl_3$).

Ethyl 4,4-difluoro-2-phenyldec-2-enoate (21)

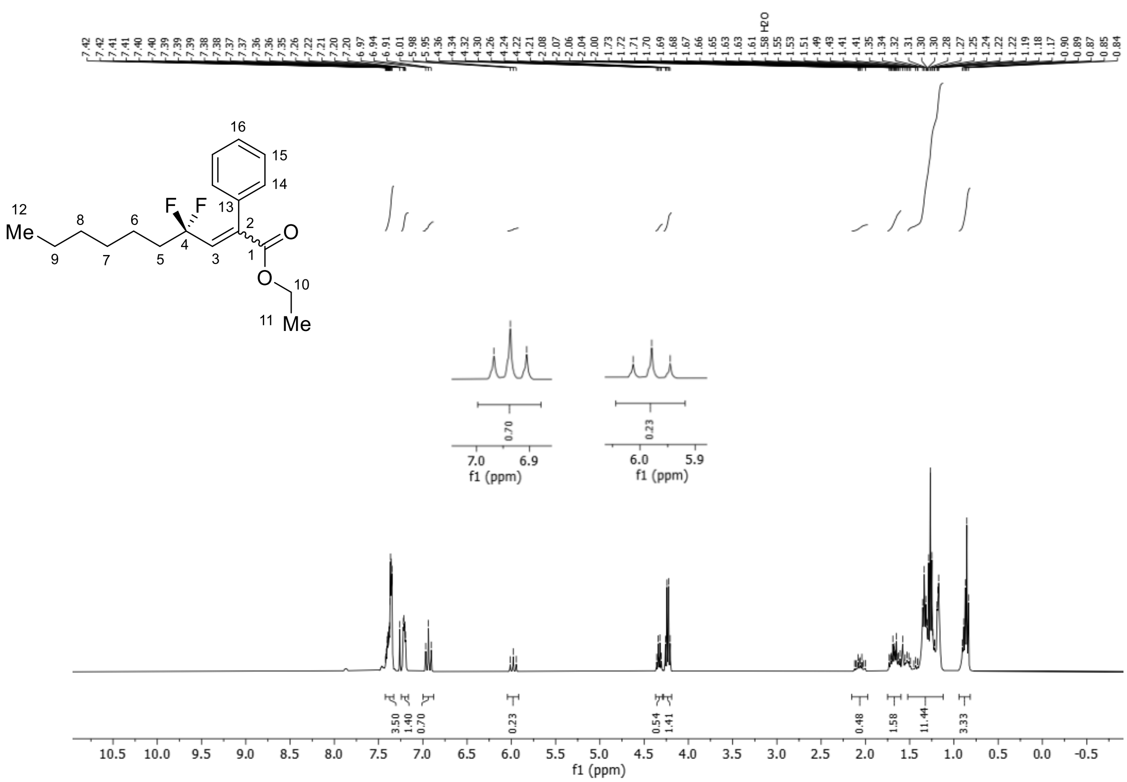

Figure S182. ^{1}H NMR of **21** (400 MHz, 299 K, $CDCl_3$).

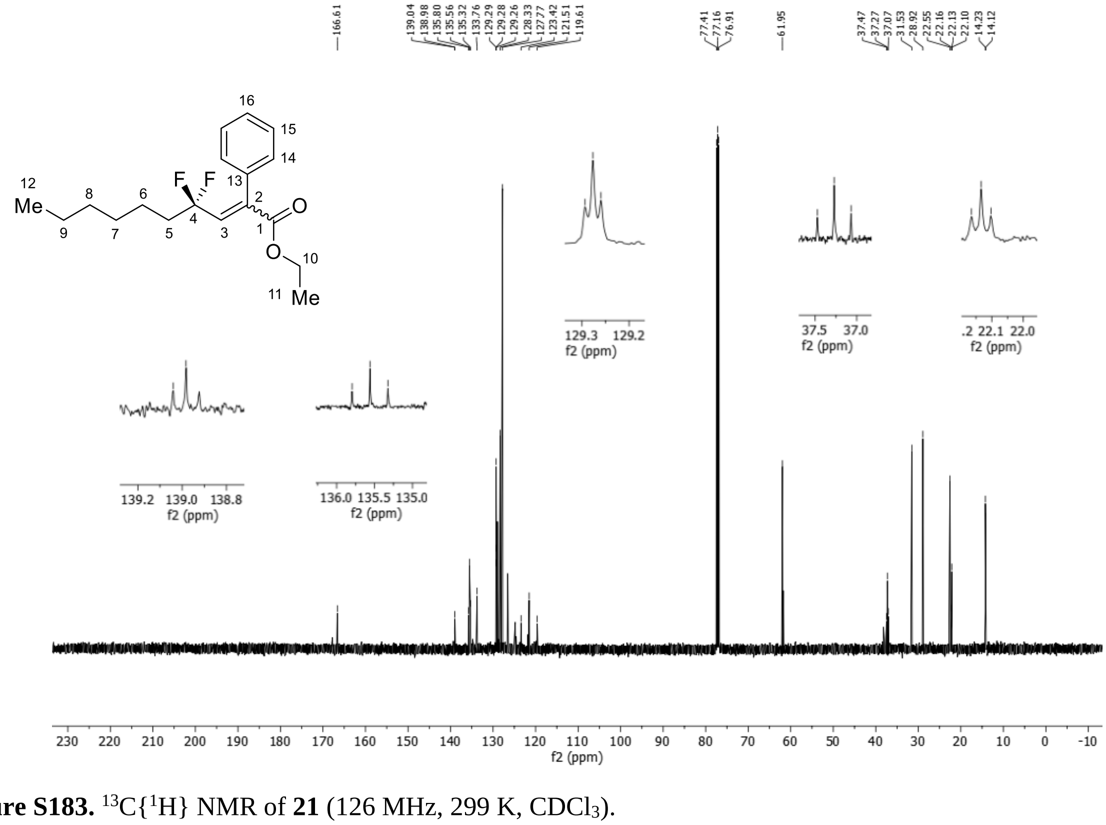

Figure S183. $^{13}C\{^{1}H\}$ NMR of **21** (126 MHz, 299 K, $CDCl_3$).

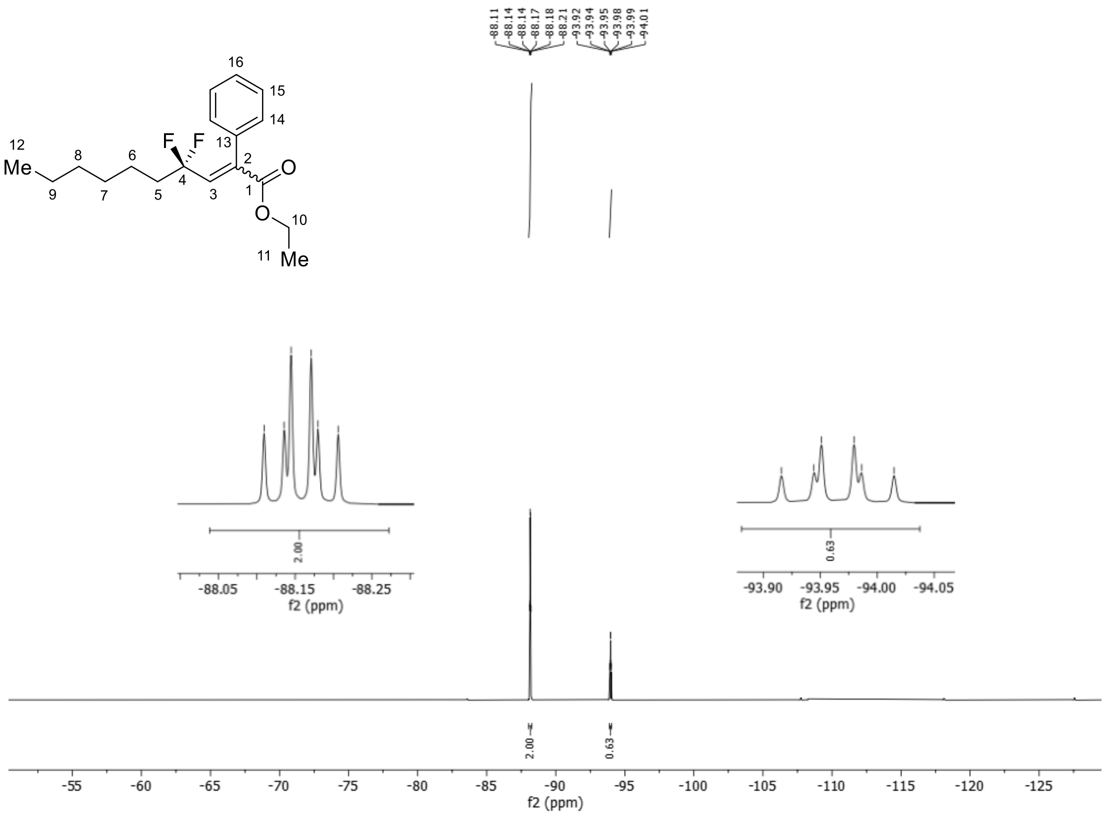

Figure S184. ^{19}F NMR of **21** (470 MHz, 299 K, $CDCl_3$).

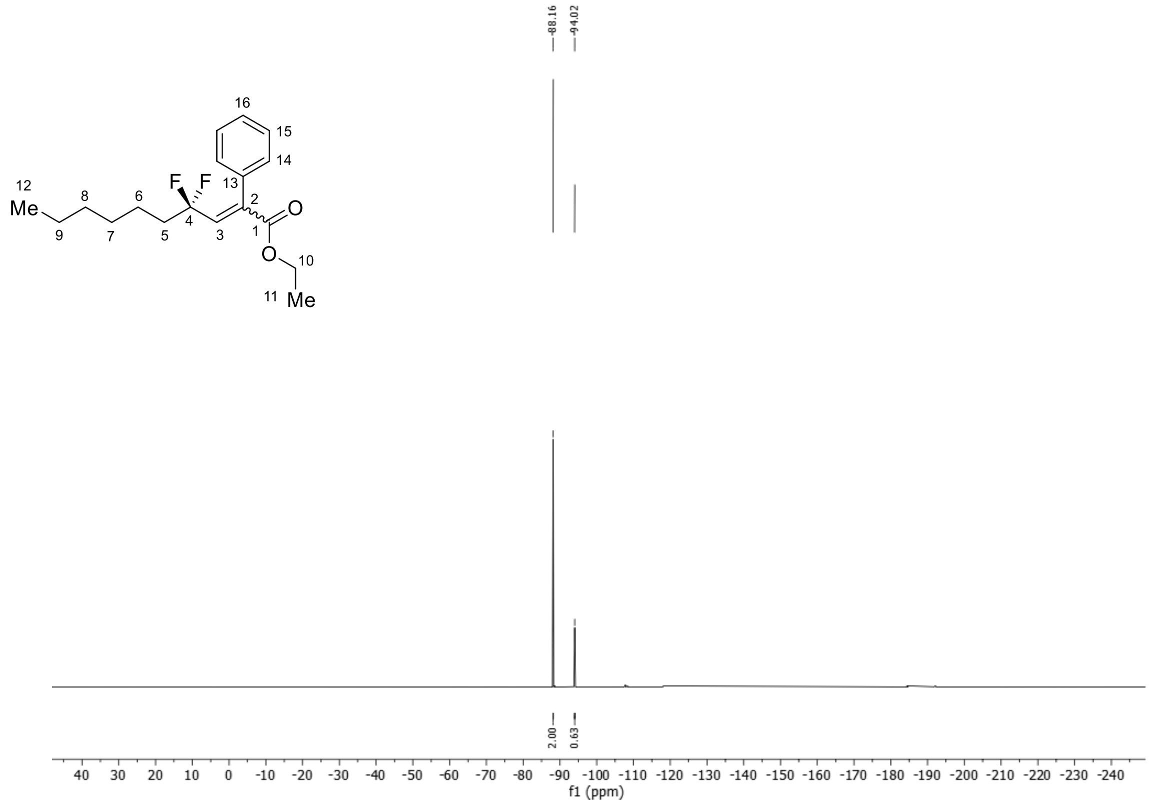

Figure S185. ^{19}F{^{1}H} NMR of **21** (377 MHz, 299 K, $CDCl_3$).

(3*R*,8*R*,10*S*,13*R*)-10,13-Dimethyl-17-((R)-6-methylheptan-2-yl)hexadecahydro-1H-cyclopenta[a]phenanthren-3-yl 4-((E)-3-(4-bromophenyl)-1,1,4,4,4-pentafluorobut-2-en-1-yl)benzoate (22)

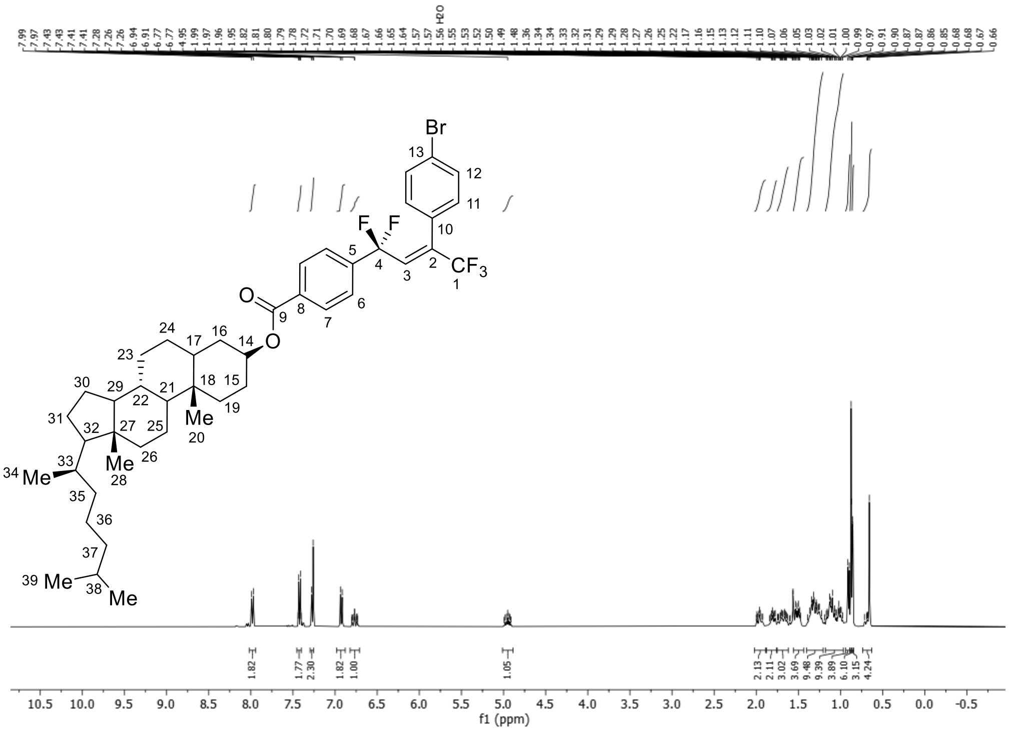

Figure S186. ^{1}H NMR of **22** (400 MHz, 299 K, $CDCl_3$).

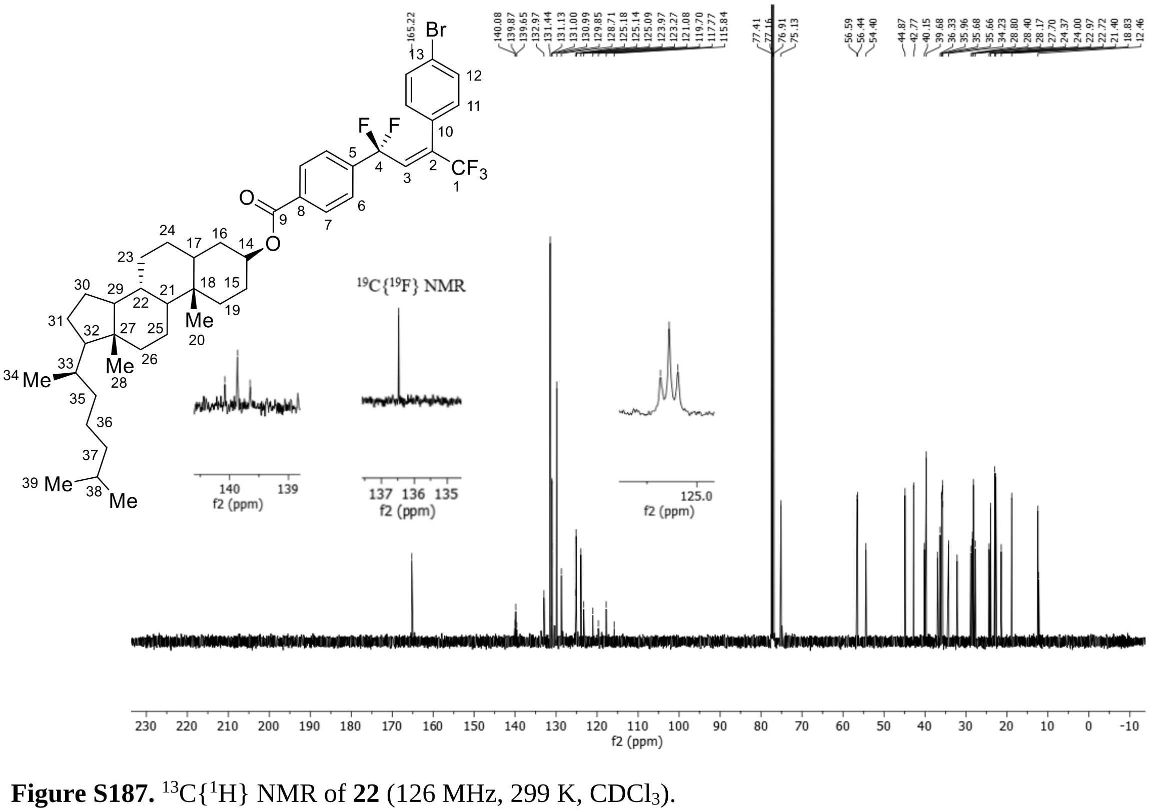

Figure S187. $^{13}C\{^{1}H\}$ NMR of **22** (126 MHz, 299 K, $CDCl_3$).

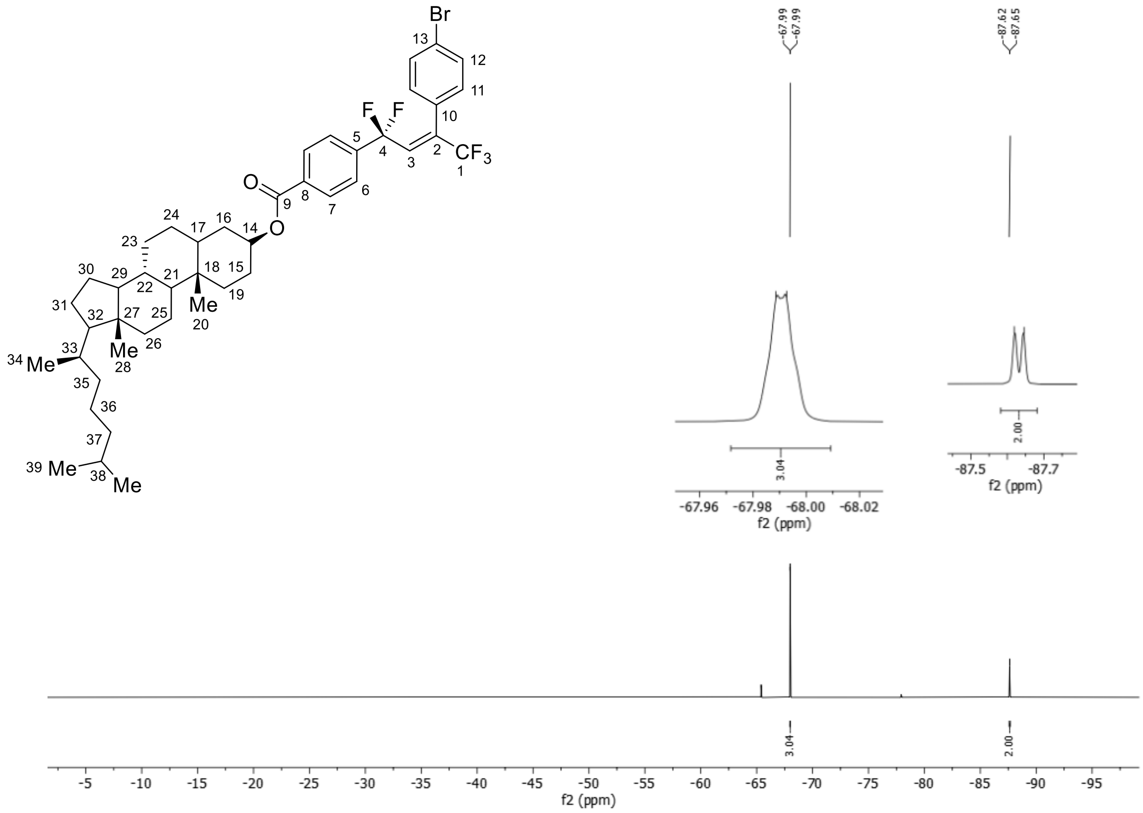

Figure S188. ^{19}F NMR of **22** (470 MHz, 299 K, $CDCl_3$).

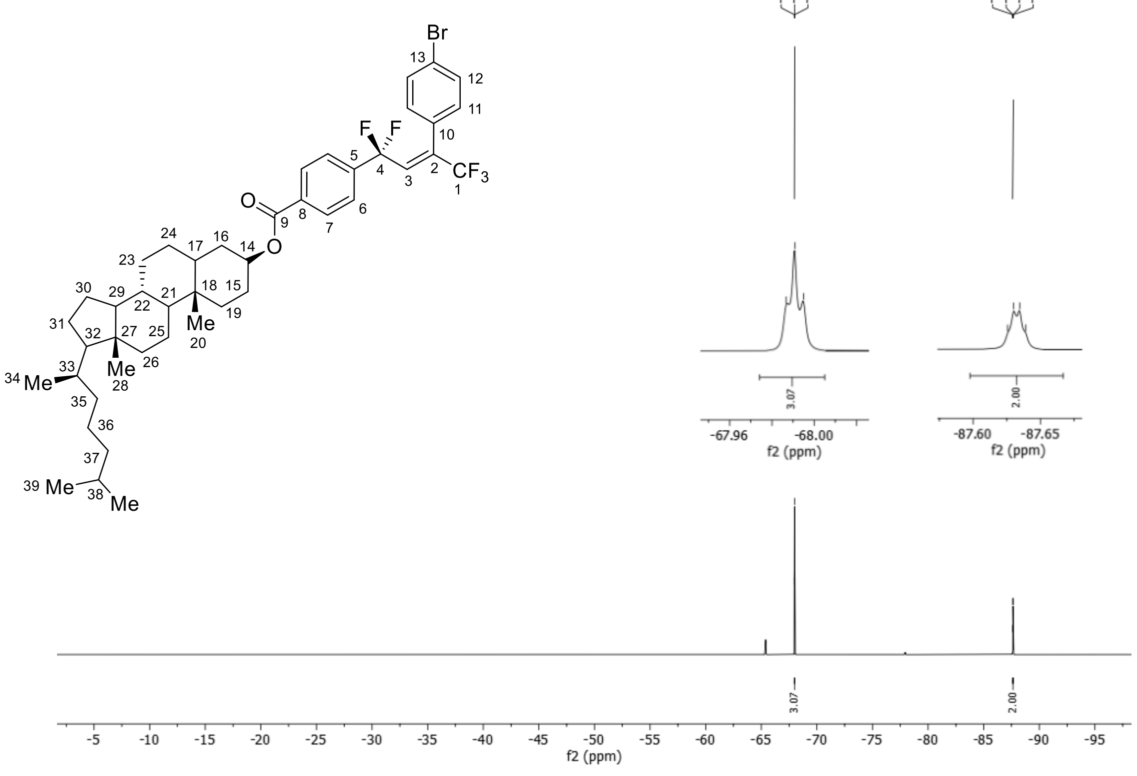

Figure S189. ^{19}F{^{1}H} NMR of **22** (470 MHz, 299 K, $CDCl_3$).

Ethyl (*Z*)-4,4-difluorodec-2-enoate (23)

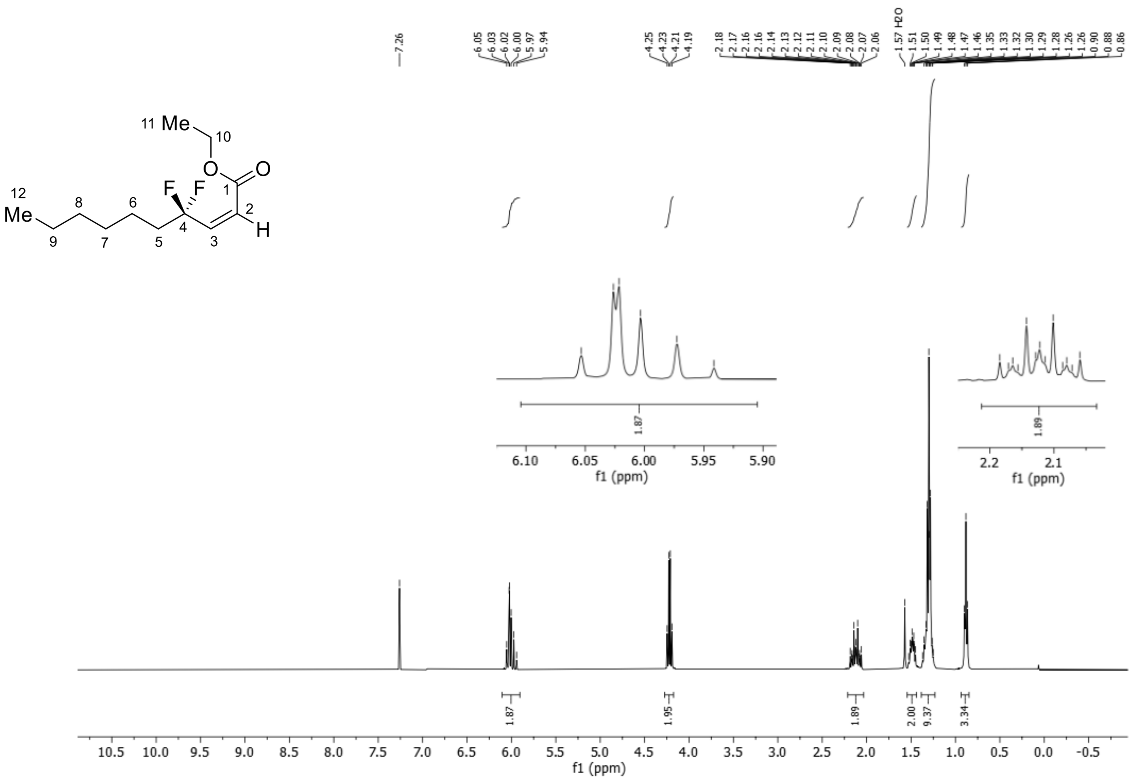

Figure S190. ^{1}H NMR of **23** (400 MHz, 299 K, $CDCl_3$).

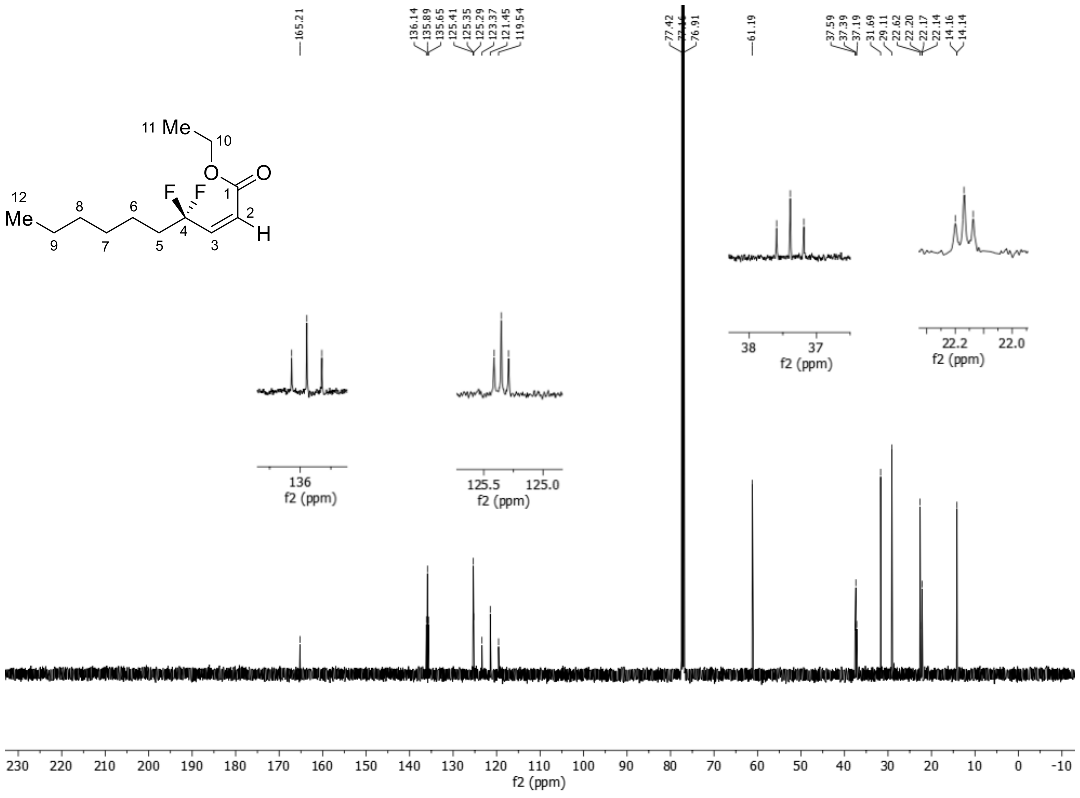

Figure S191. $^{13}C\{^{1}H\}$ NMR of **23** (126 MHz, 299 K, $CDCl_3$).

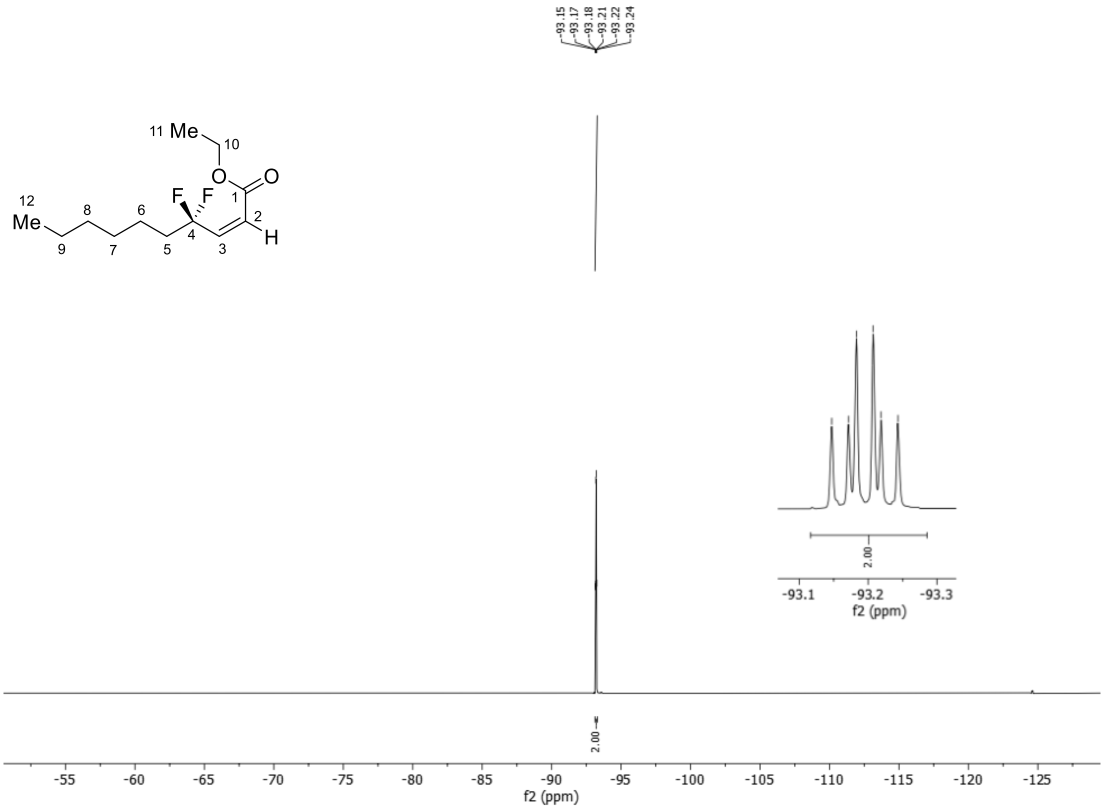

Figure S192. ^{19}F NMR of **23** (470 MHz, 299 K, $CDCl_3$).

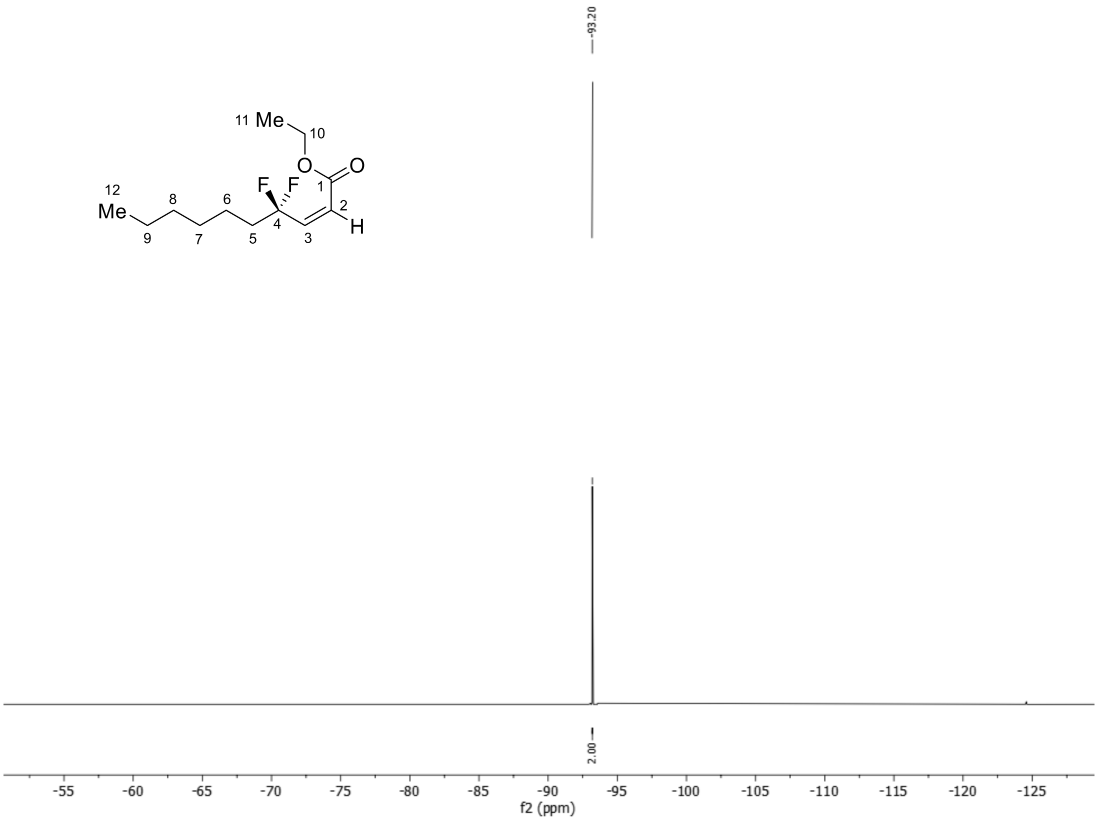

Figure S193. ^{19}F{^{1}H} NMR of **23** (470 MHz, 299 K, $CDCl_3$).

Ethyl (*Z*)-4,4-difluoro-13-hydroxytridec-2-enoate (24)

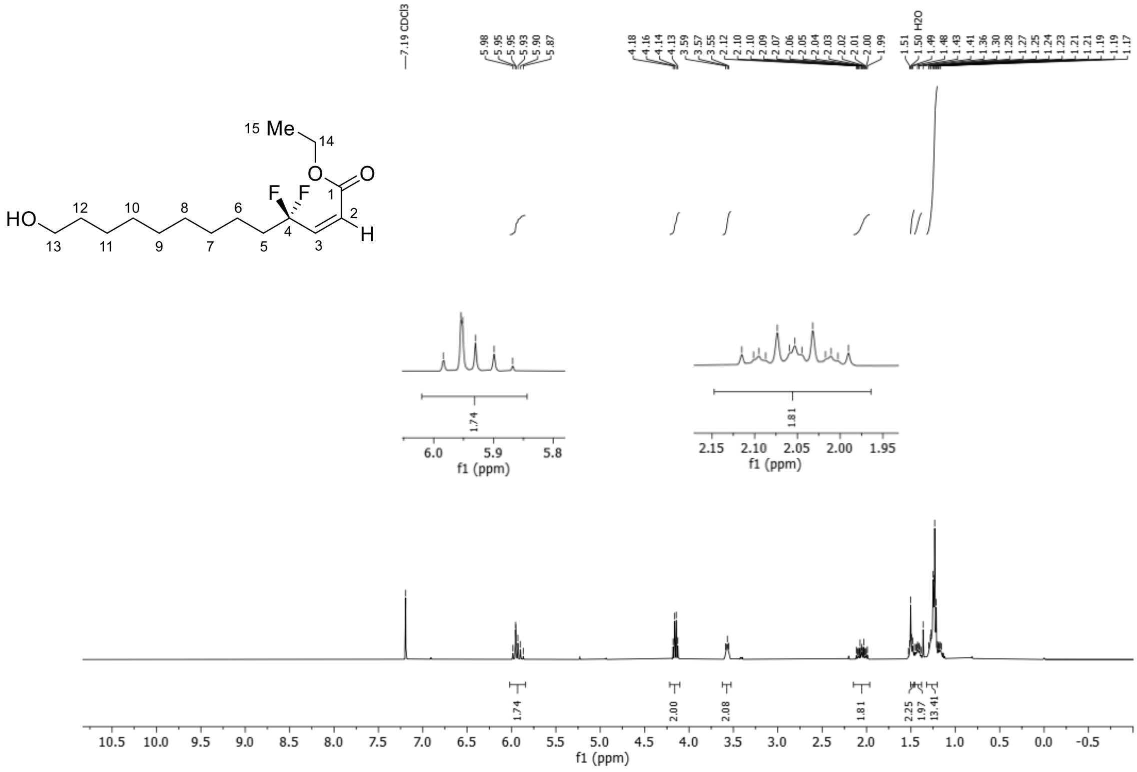

Figure S194. ^{1}H NMR of **24** (400 MHz, 299 K, $CDCl_3$).

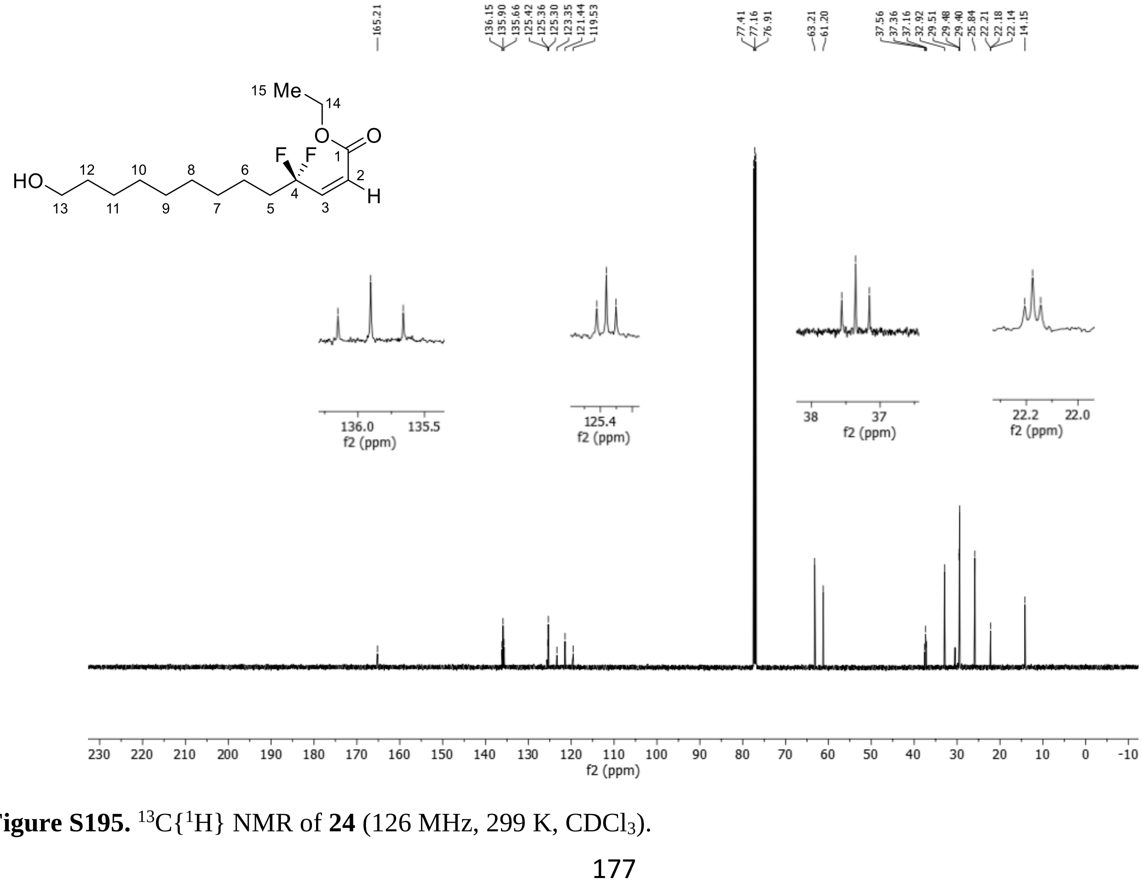

Figure S195. $^{13}C\{^{1}H\}$ NMR of **24** (126 MHz, 299 K, $CDCl_3$).

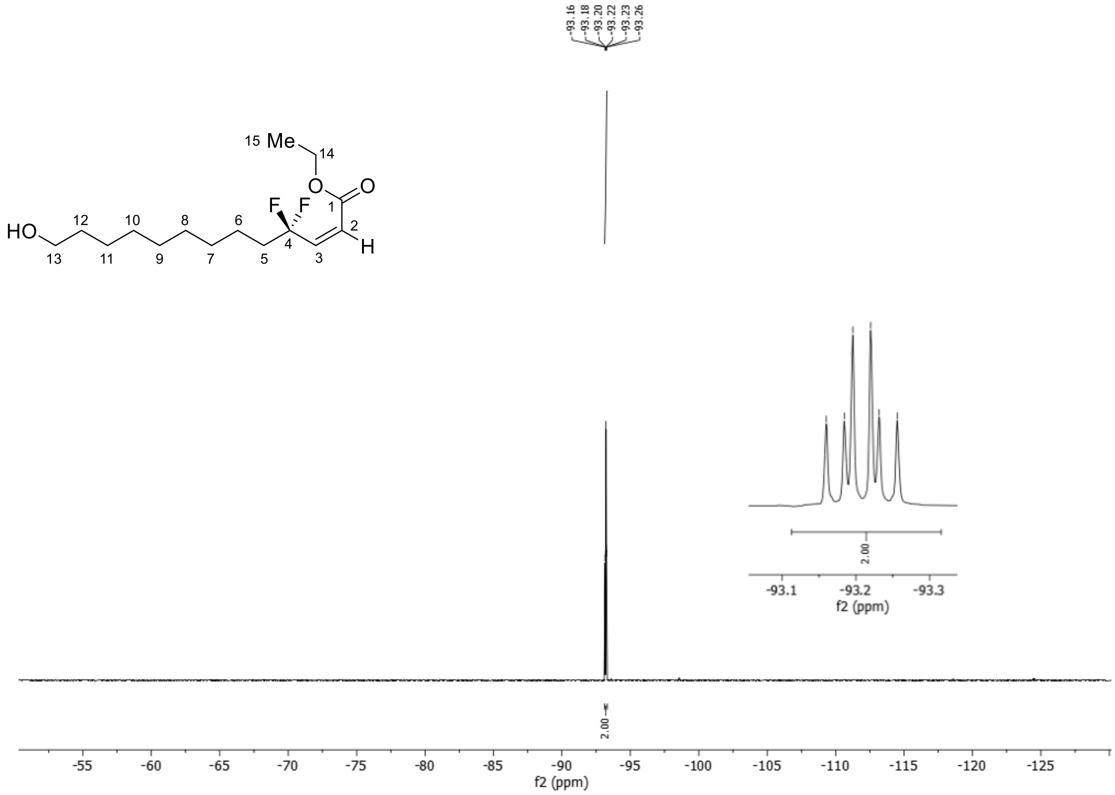

Figure S196. ^{19}F NMR of **24** (470 MHz, 299 K, $CDCl_3$).

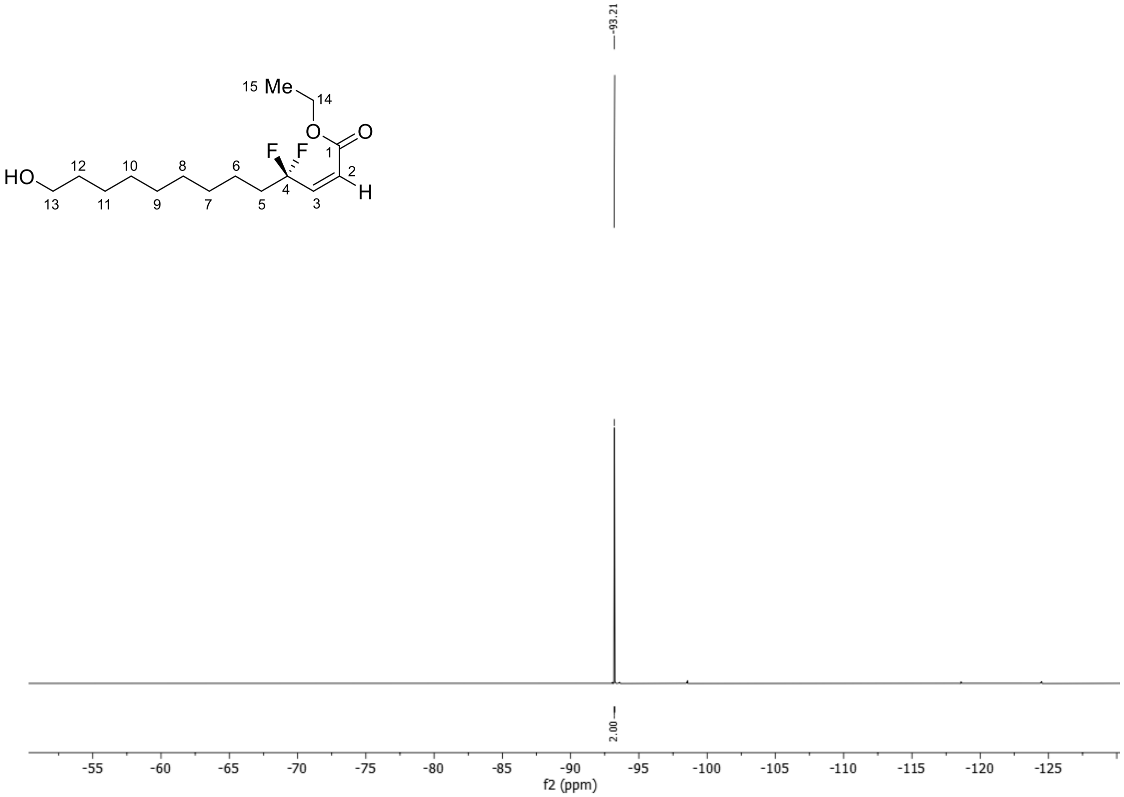

Figure S197. ^{19}F{^{1}H} NMR of **24** (470 MHz, 299 K, $CDCl_3$).

(*Z*)-13-Ethoxy-10,10-difluoro-13-oxotridec-11-en-1-yl nicotinate (25)

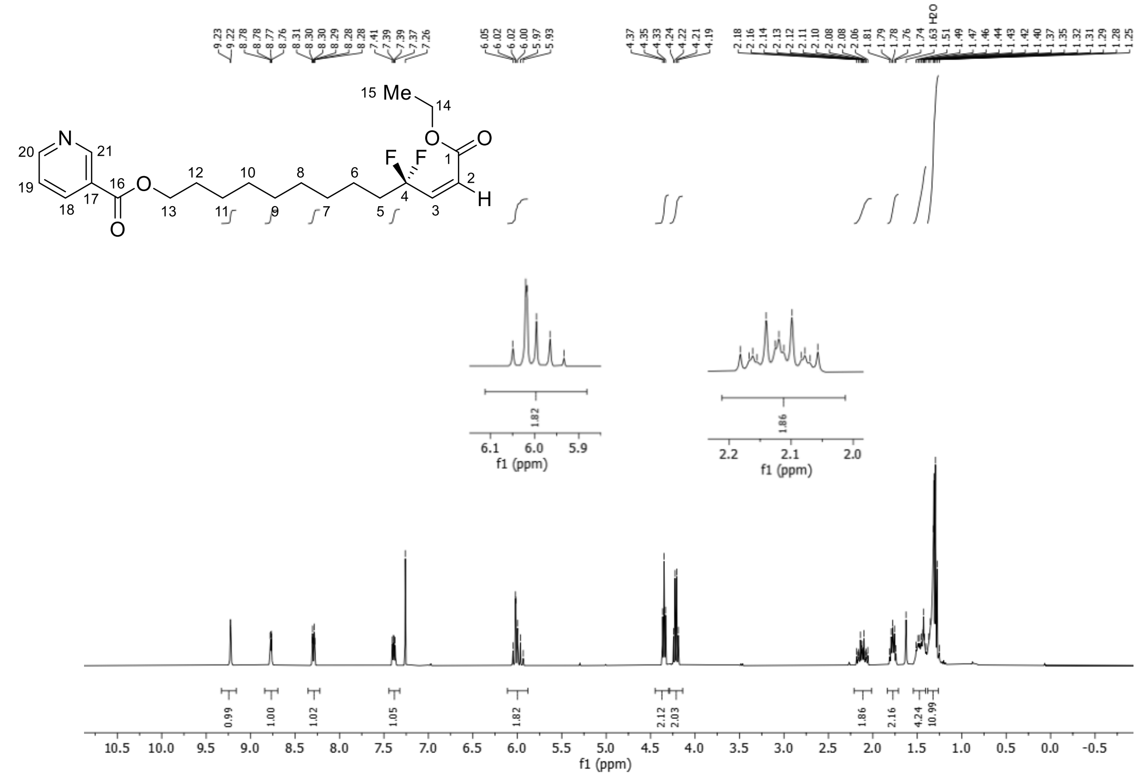

Figure S198. ^{1}H NMR of **25** (400 MHz, 299 K, $CDCl_3$).

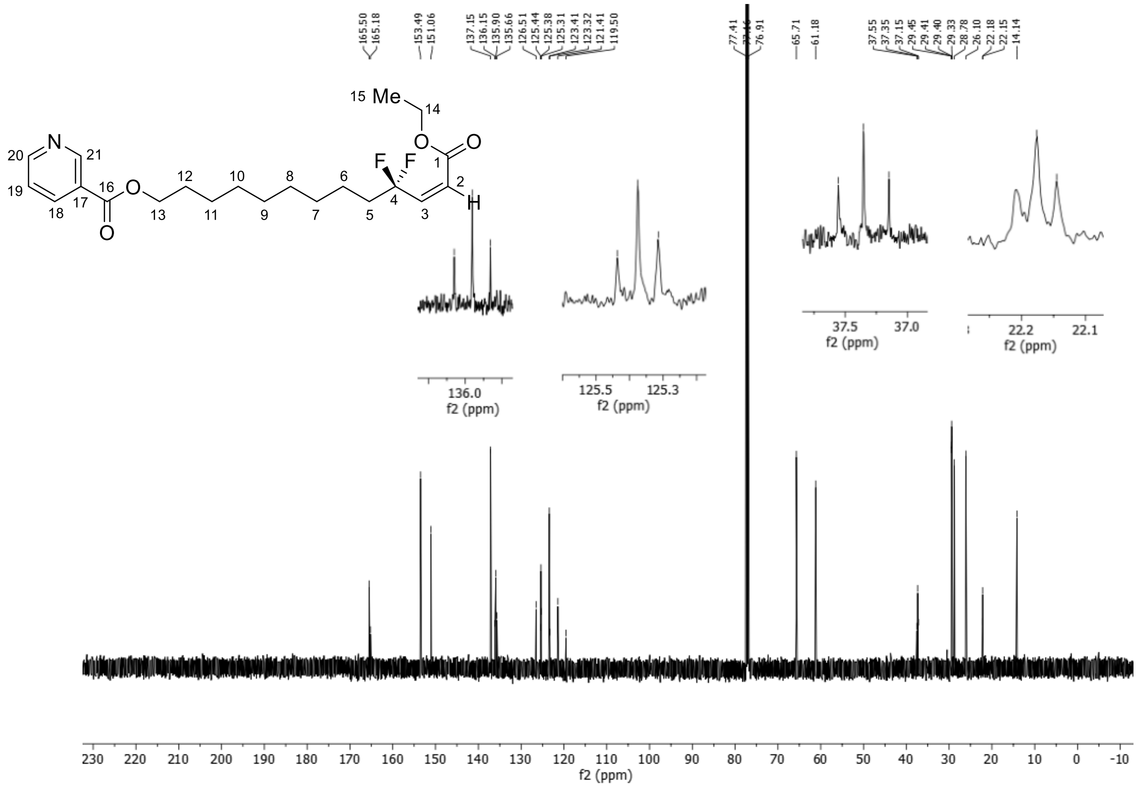

Figure S199. $^{13}C\{^{1}H\}$ NMR of **25** (126 MHz, 299 K, $CDCl_3$).

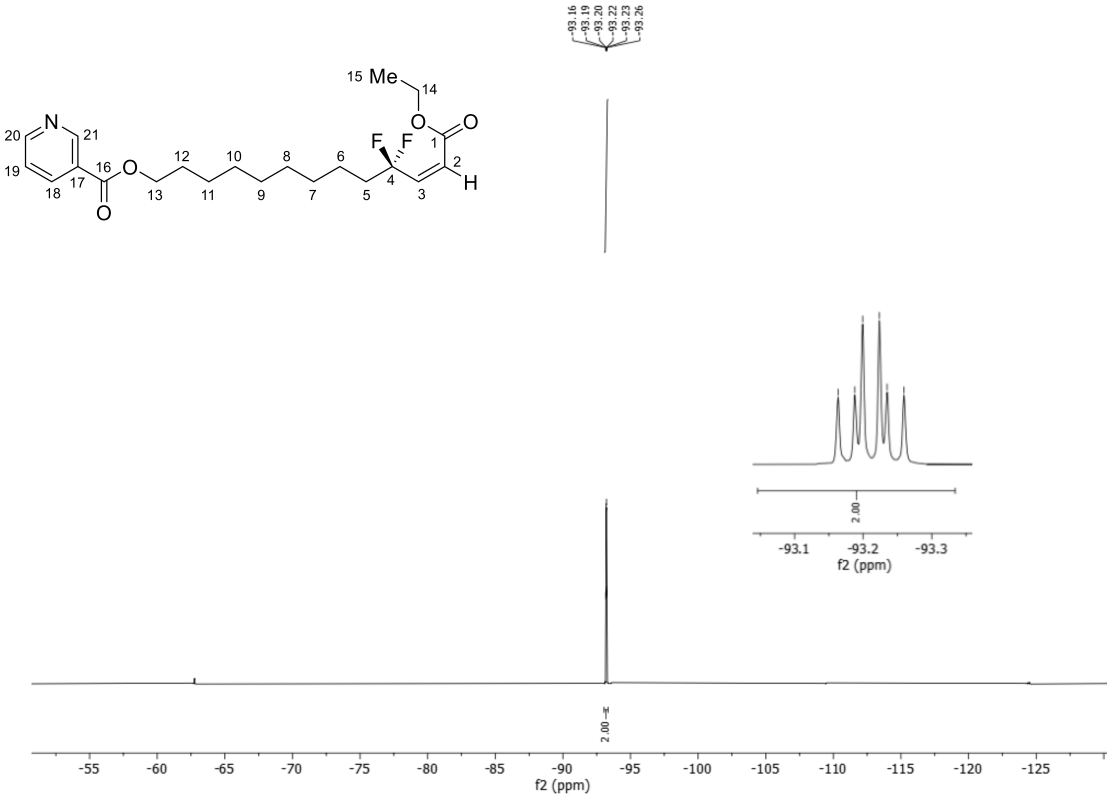

Figure S200. ^{19}F NMR of **25** (470 MHz, 299 K, $CDCl_3$).

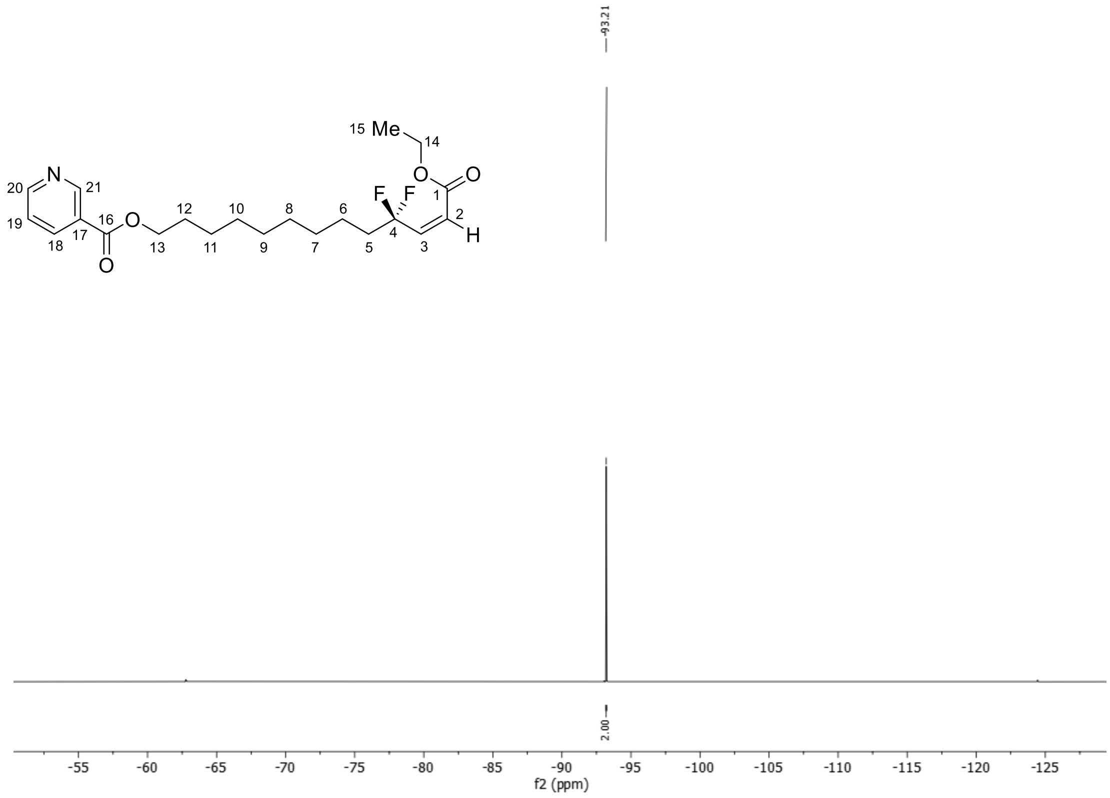

Figure S201. $^{19}F\{^1H\}$ NMR of **25** (470 MHz, 299 K, $CDCl_3$).

Methyl 2-(difluoro(phenyl)methyl)-3-methylbut-2-enoate (26)

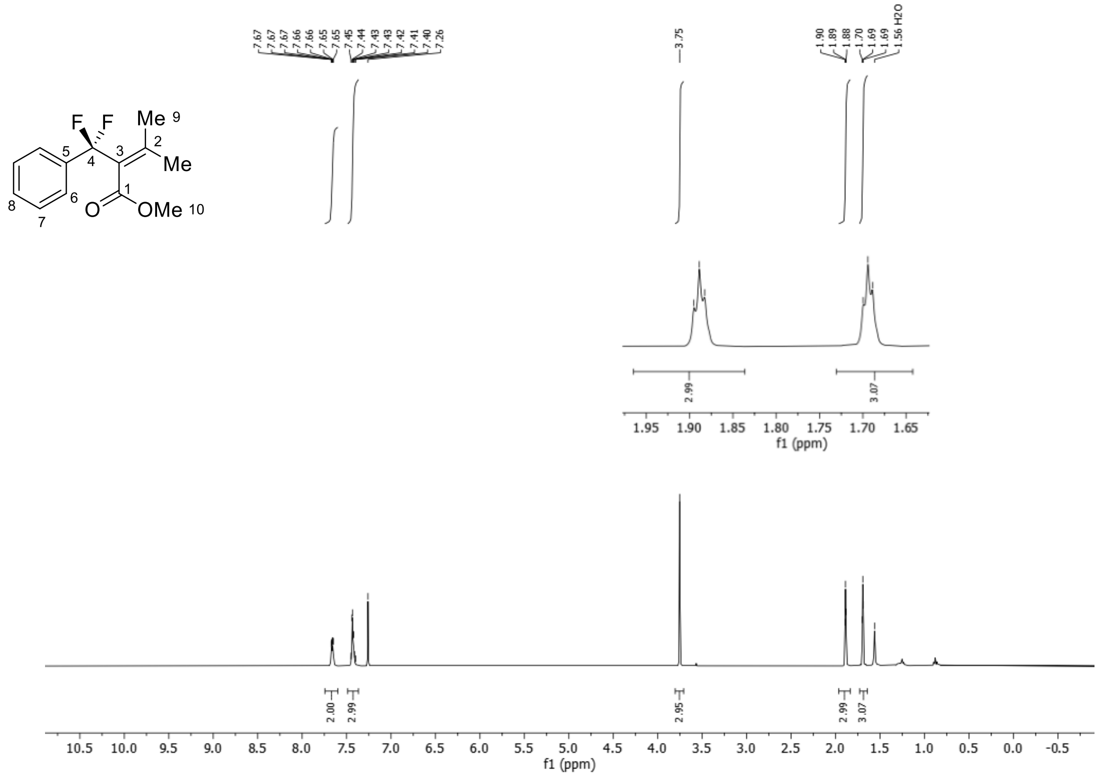

Figure S202. ^{1}H NMR of **26** (400 MHz, 299 K, $CDCl_3$).

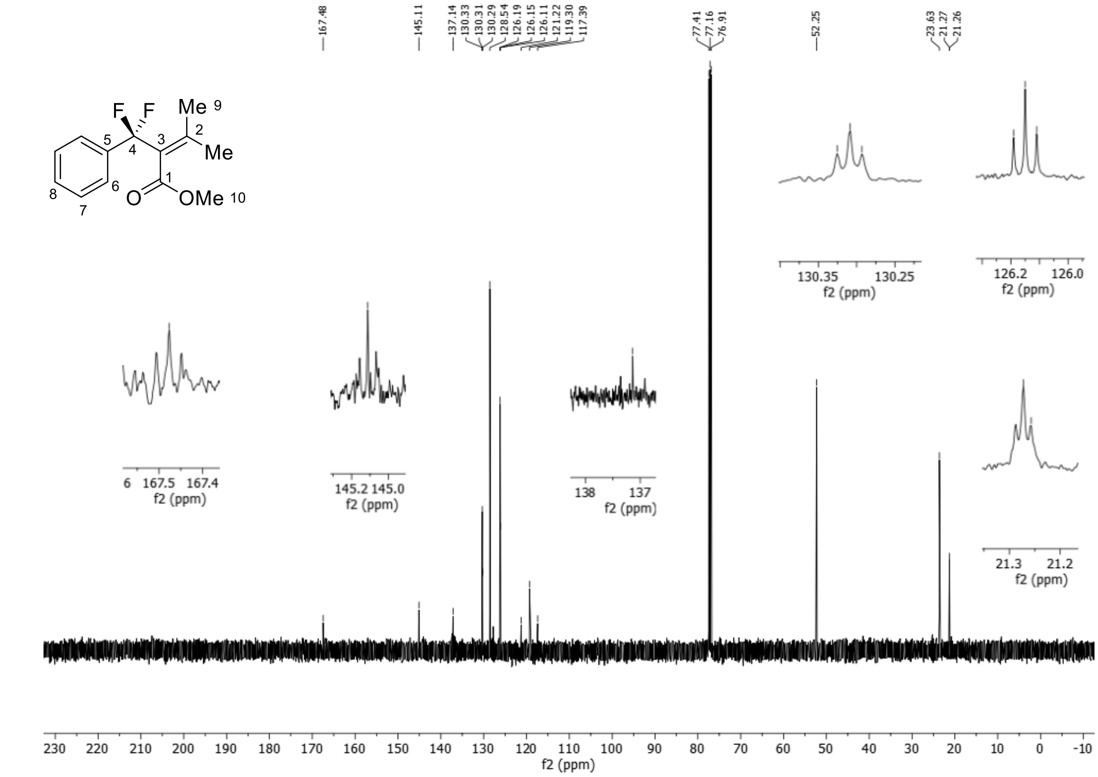

Figure S203. $^{13}C\{^{1}H\}$ NMR of **26** (126 MHz, 299 K, $CDCl_3$).

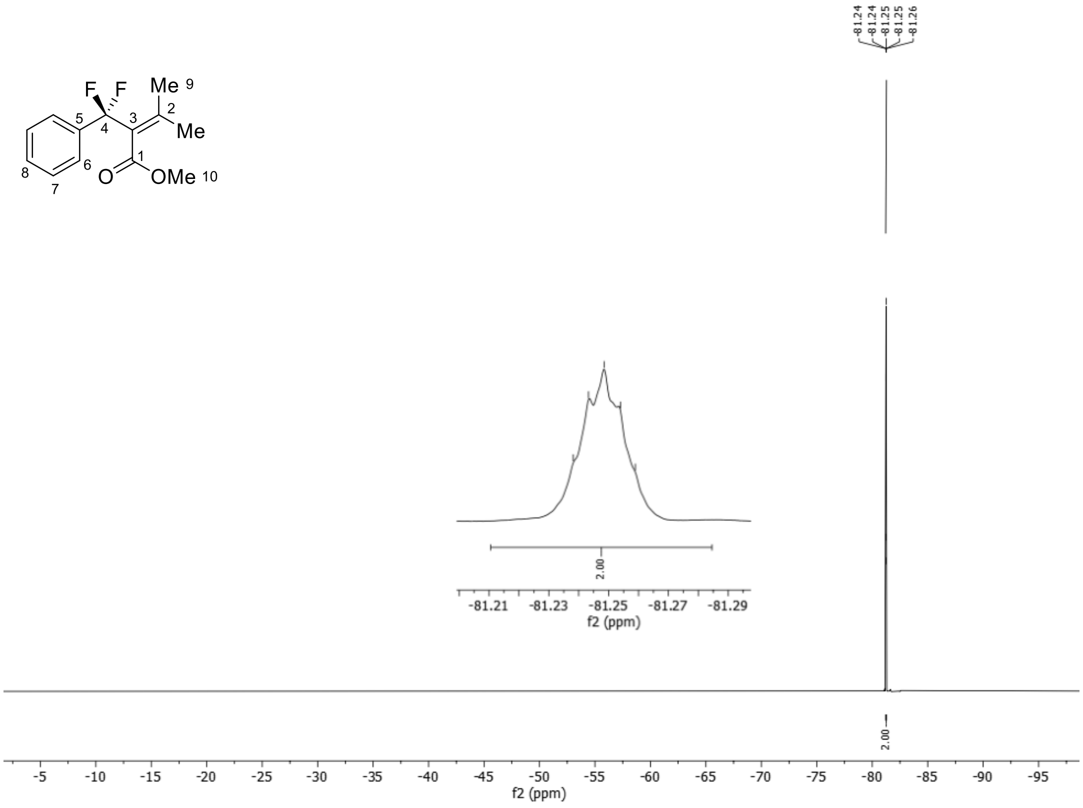

Figure S204. ^{19}F NMR of **26** (470 MHz, 299 K, $CDCl_3$).

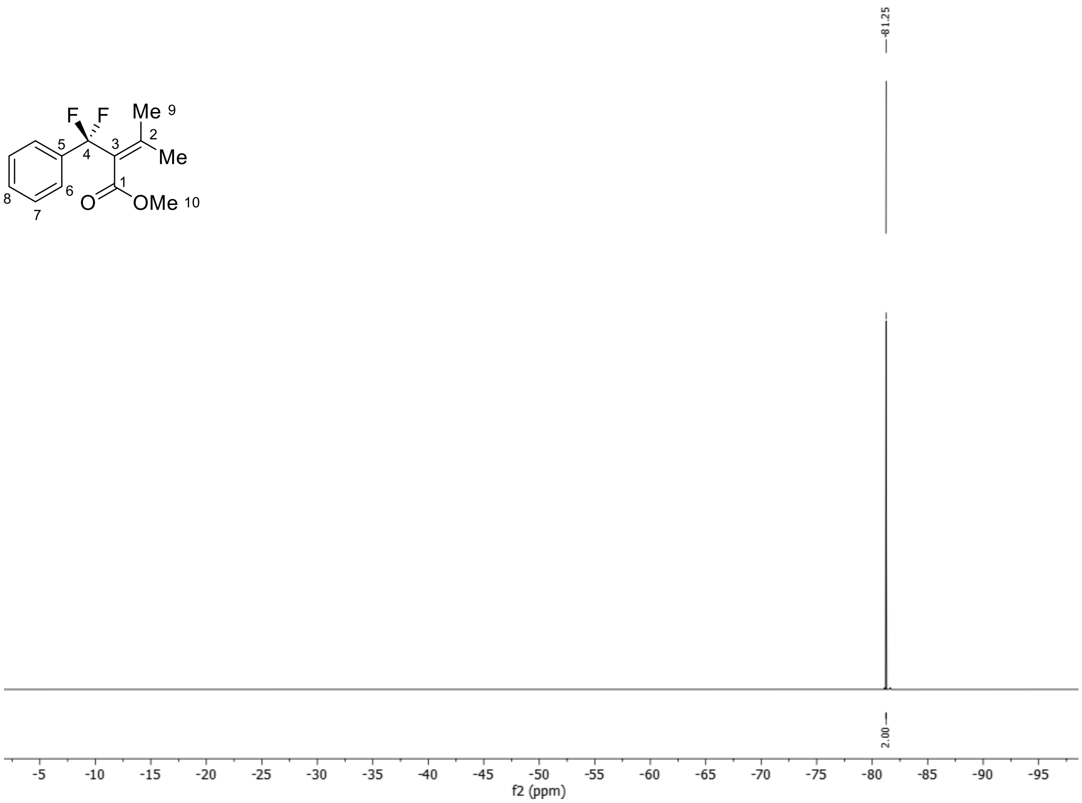

Figure S205. $^{19}F\{^1H\}$ NMR of **26** (470 MHz, 299 K, $CDCl_3$).

Methyl 2-(difluoro(4-(trifluoromethyl)phenyl)methyl)-3-methylbut-2-enoate (27)

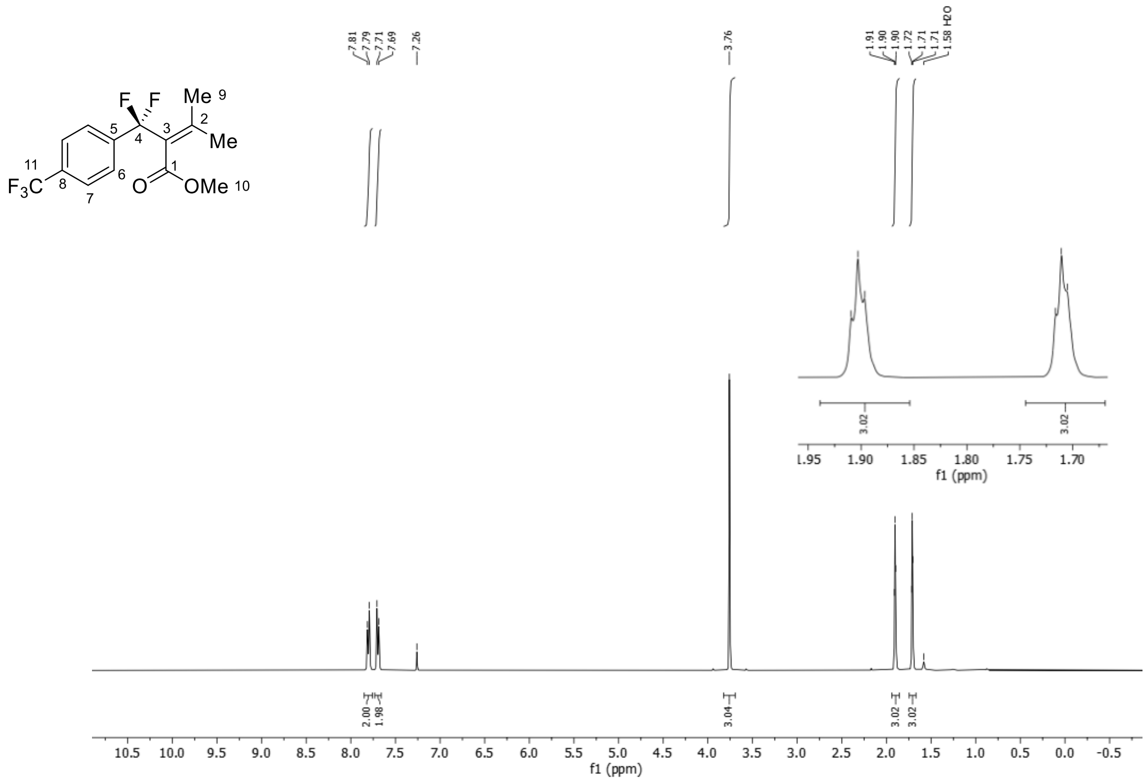

Figure S206. ^{1}H NMR of **27** (400 MHz, 299 K, $CDCl_3$).

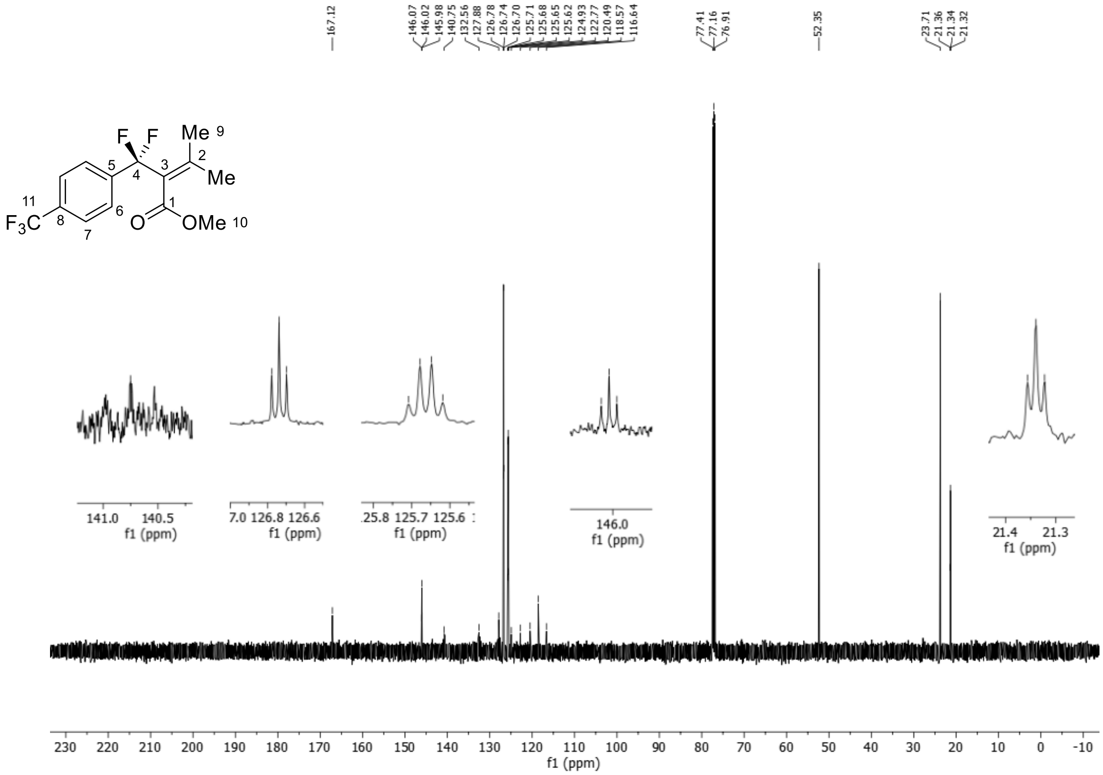

Figure S207. $^{13}C\{^{1}H\}$ NMR of **27** (126 MHz, 299 K, $CDCl_3$).

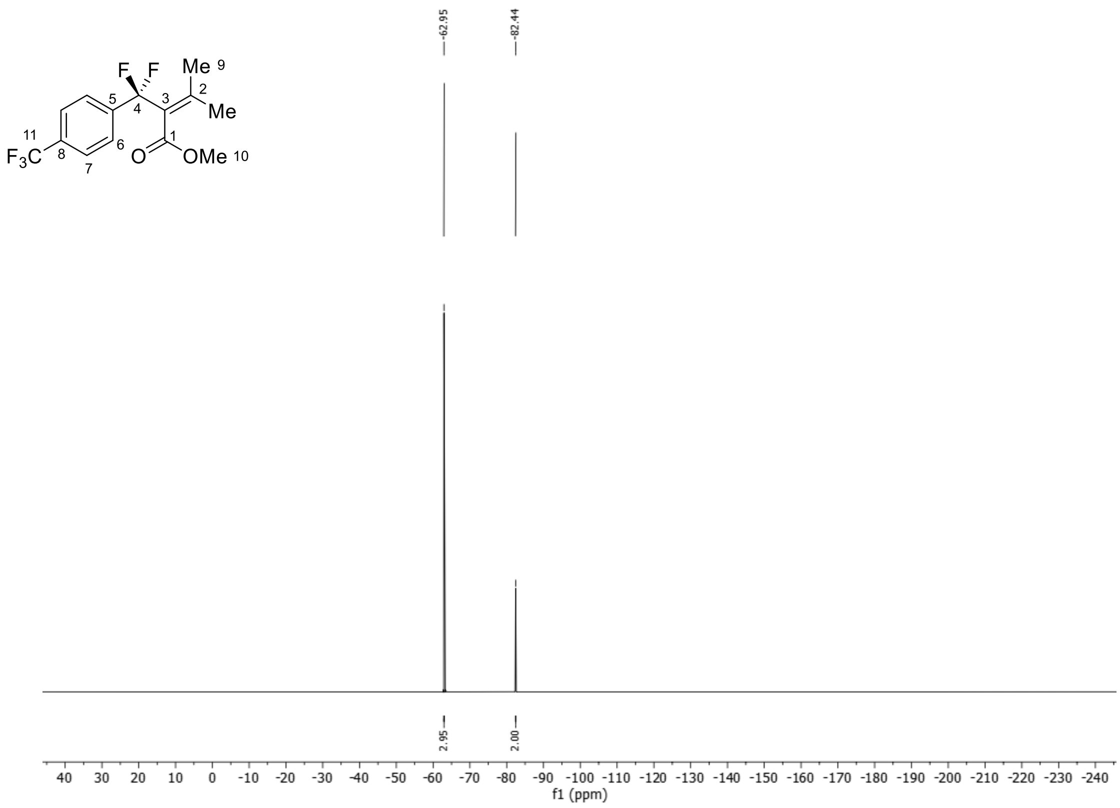

Figure S208. ^{19}F NMR of **27** (470 MHz, 299 K, $CDCl_3$).

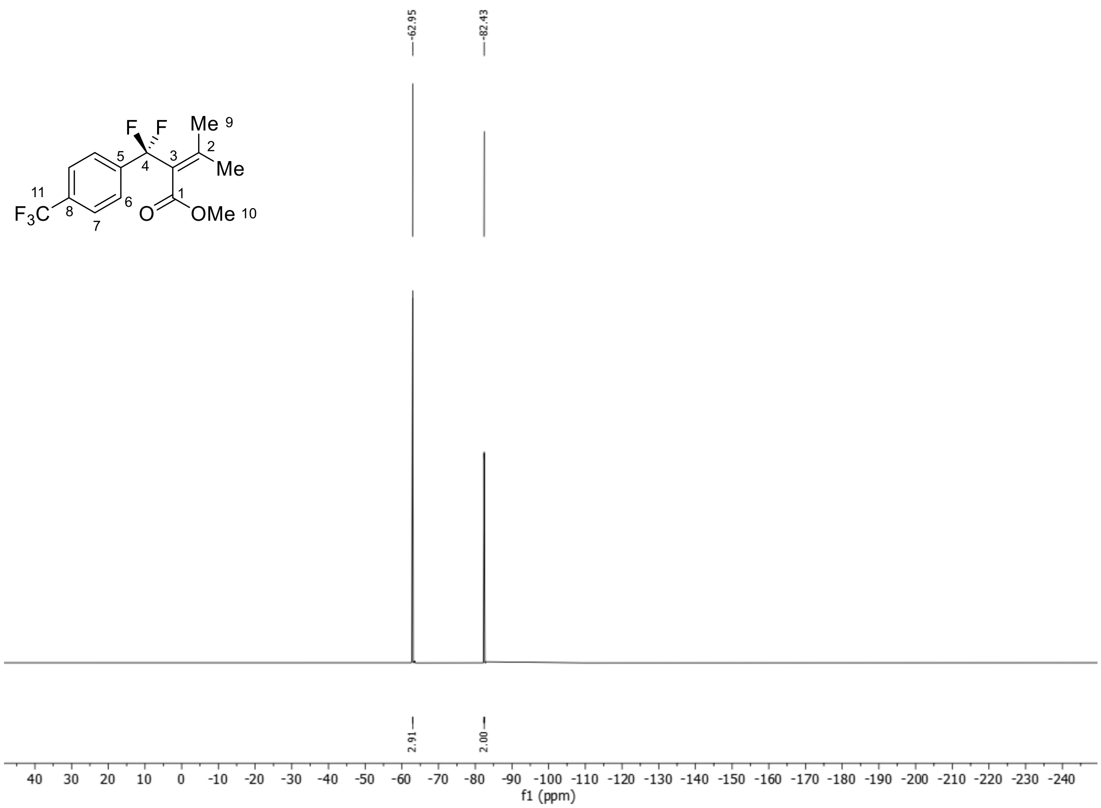

Figure S209. $^{19}F\{^1H\}$ NMR of **27** (377 MHz, 299 K, $CDCl_3$).

2-(Difluoro(4-(trifluoromethyl)phenyl)methyl)-N,N,3-trimethylbut-2-enamide (28)

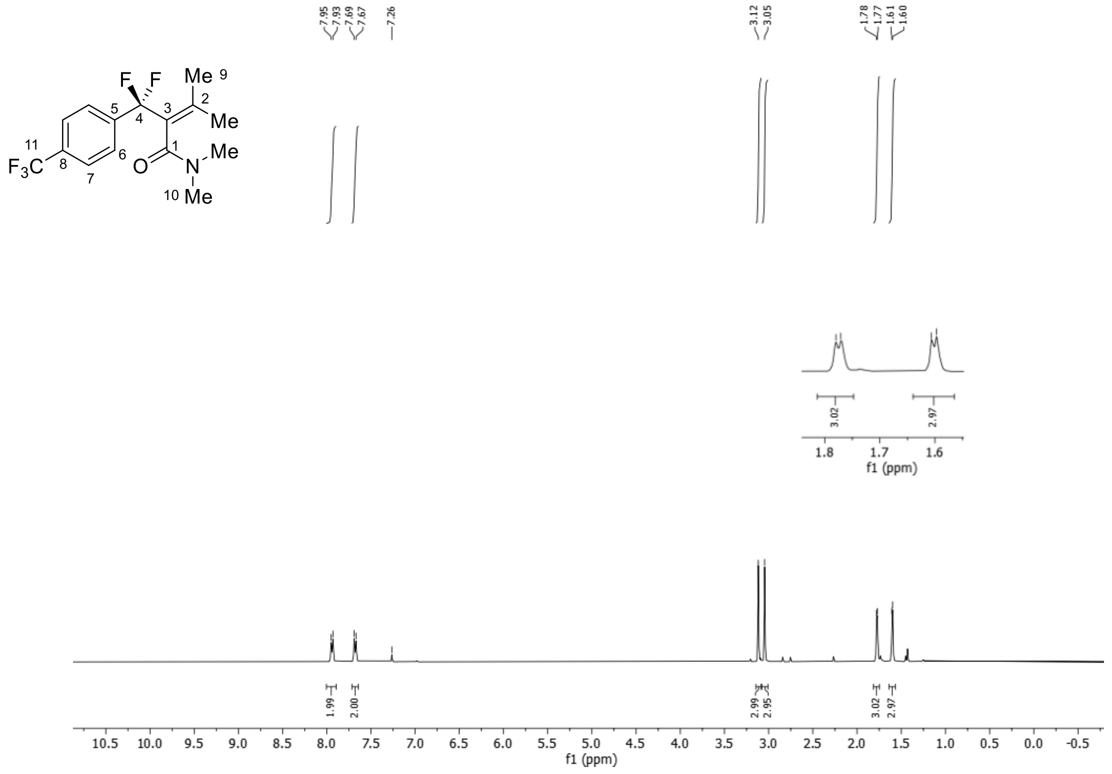

Figure S210. ^{1}H NMR of **28** (400 MHz, 299 K, $CDCl_3$).

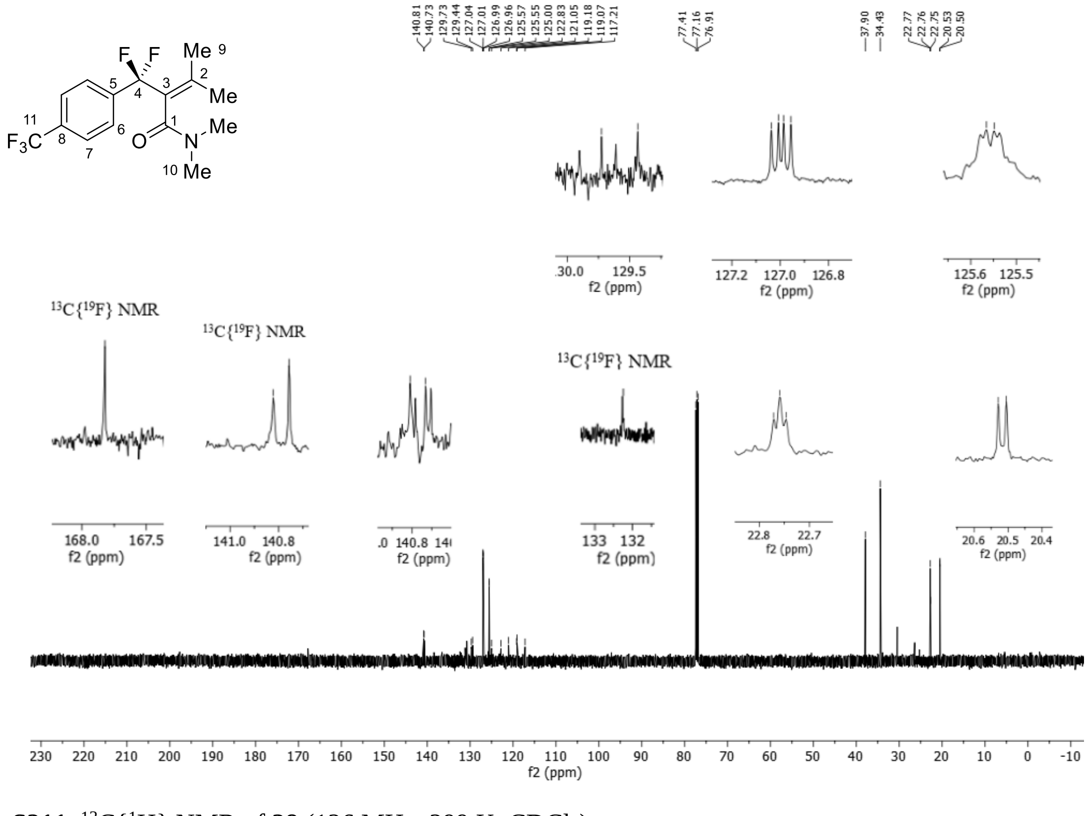

Figure S211. $^{13}C\{^{1}H\}$ NMR of **28** (126 MHz, 299 K, $CDCl_3$).

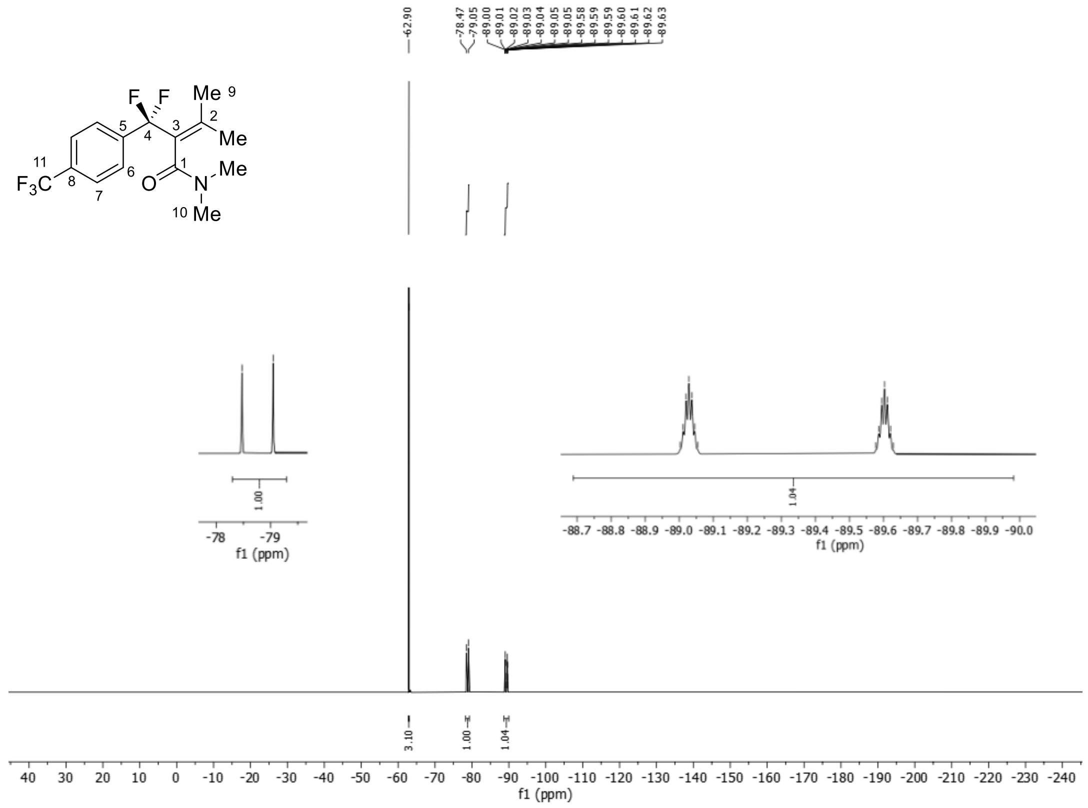

Figure S212. ^{19}F NMR of **28** (470 MHz, 299 K, $CDCl_3$).

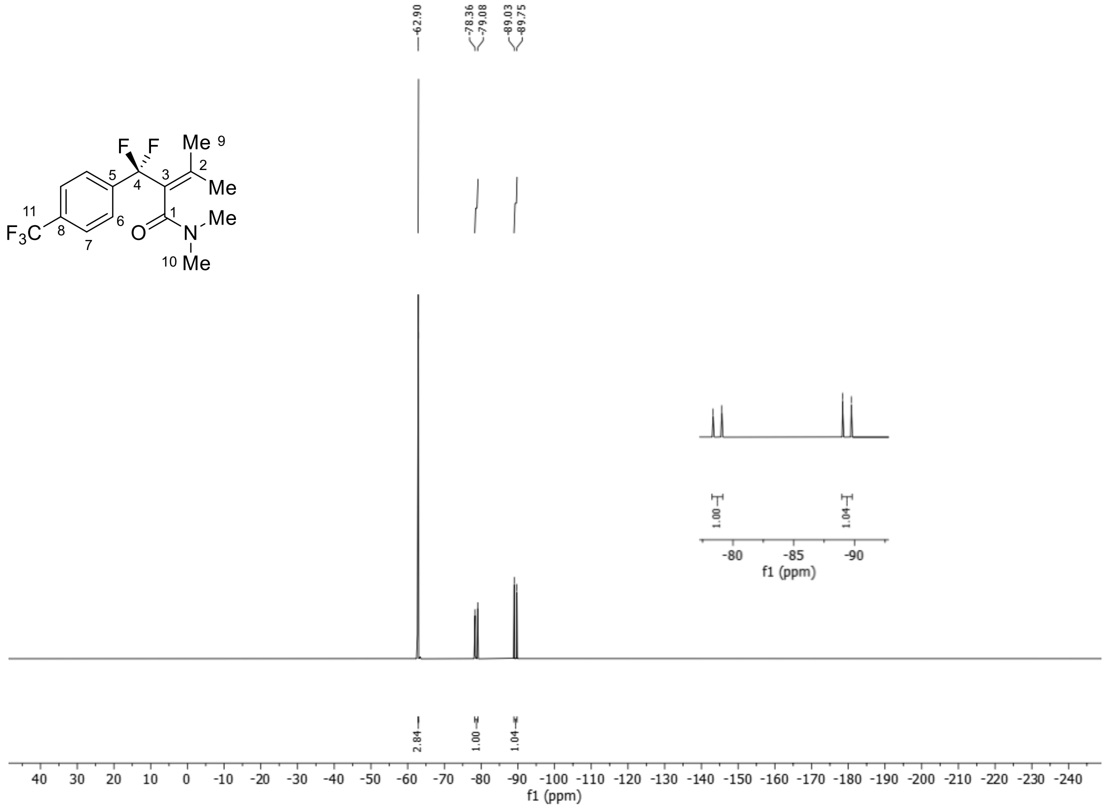

Figure S213. $^{19}F\{^1H\}$ NMR of **28** (377 MHz, 299 K, $CDCl_3$).

Methyl 2-cyclohexylidene-3,3-difluoro-3-(4-(trifluoromethyl)phenyl)propanoate (29)

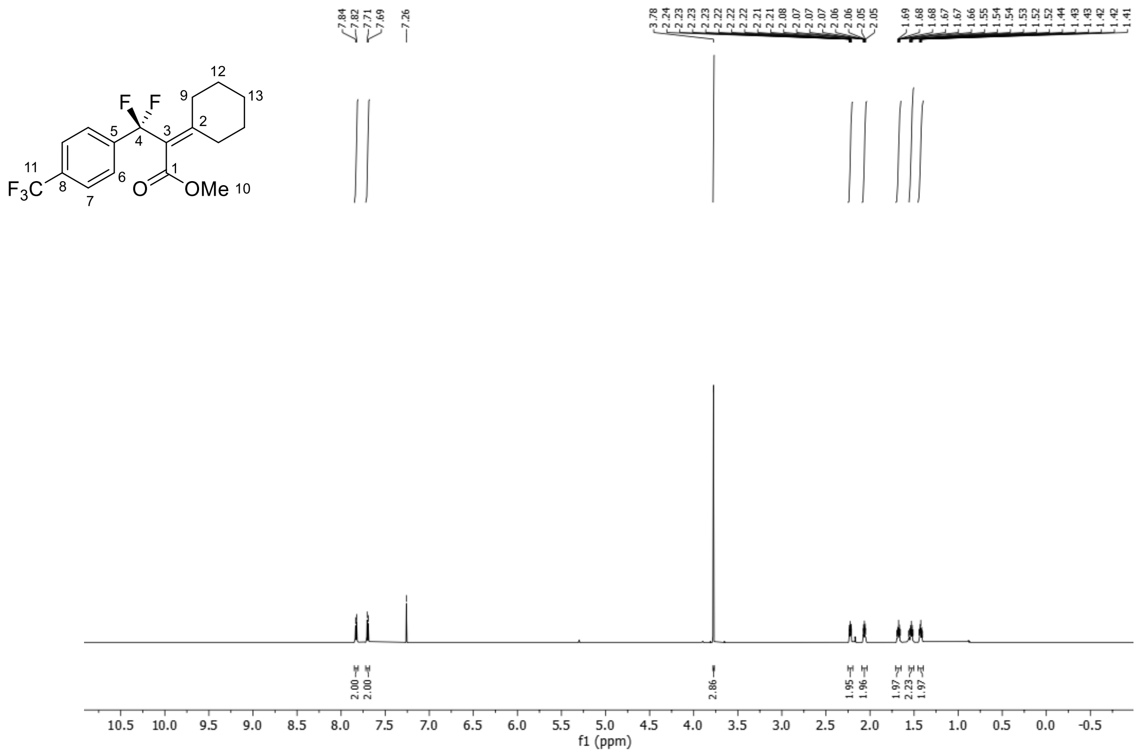

Figure S214. ^{1}H NMR of **29** (599 MHz, 299 K, $CDCl_3$).

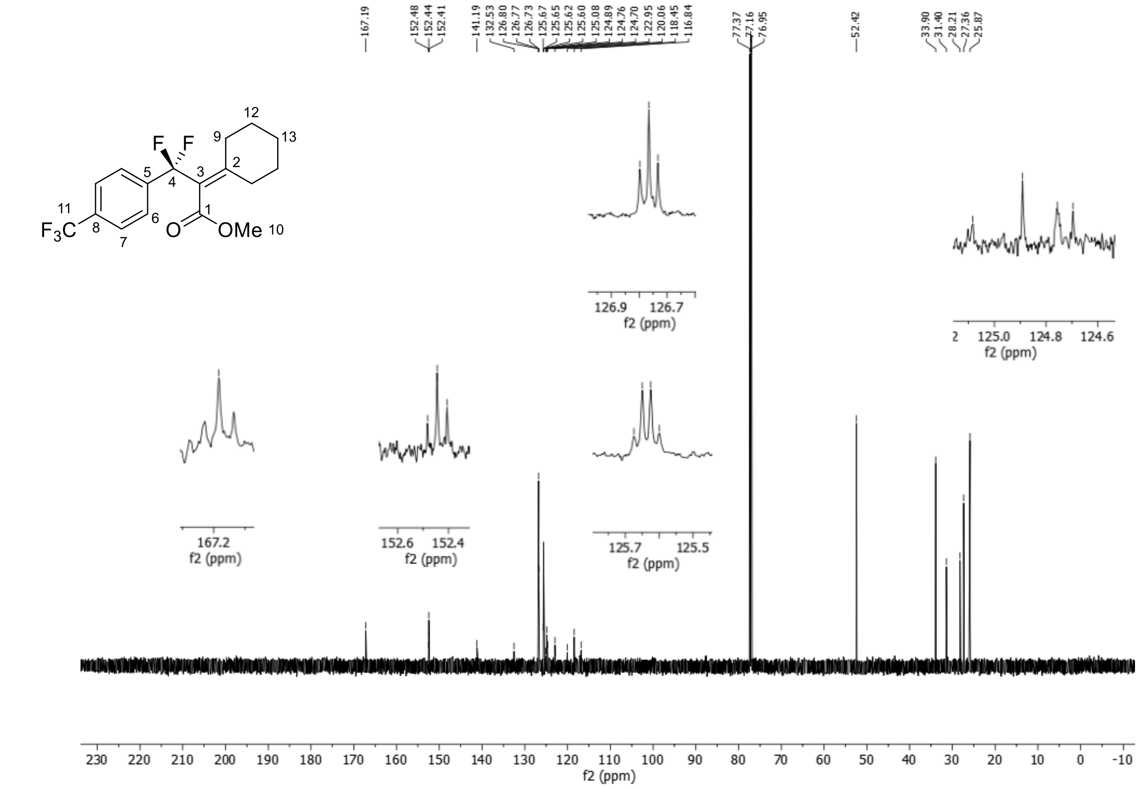

Figure S215. $^{13}C\{^{1}H\}$ NMR of **29** (151 MHz, 299 K, $CDCl_3$).

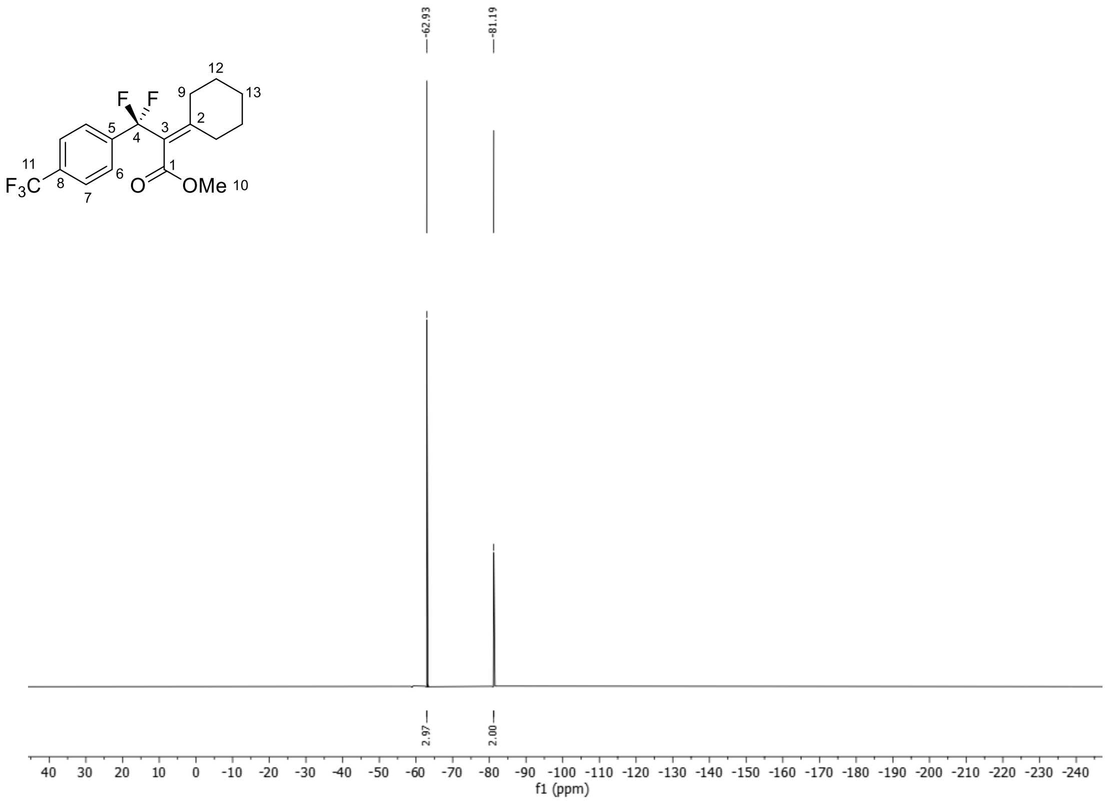

Figure S216. ^{19}F NMR of **29** (564 MHz, 299 K, $CDCl_3$).

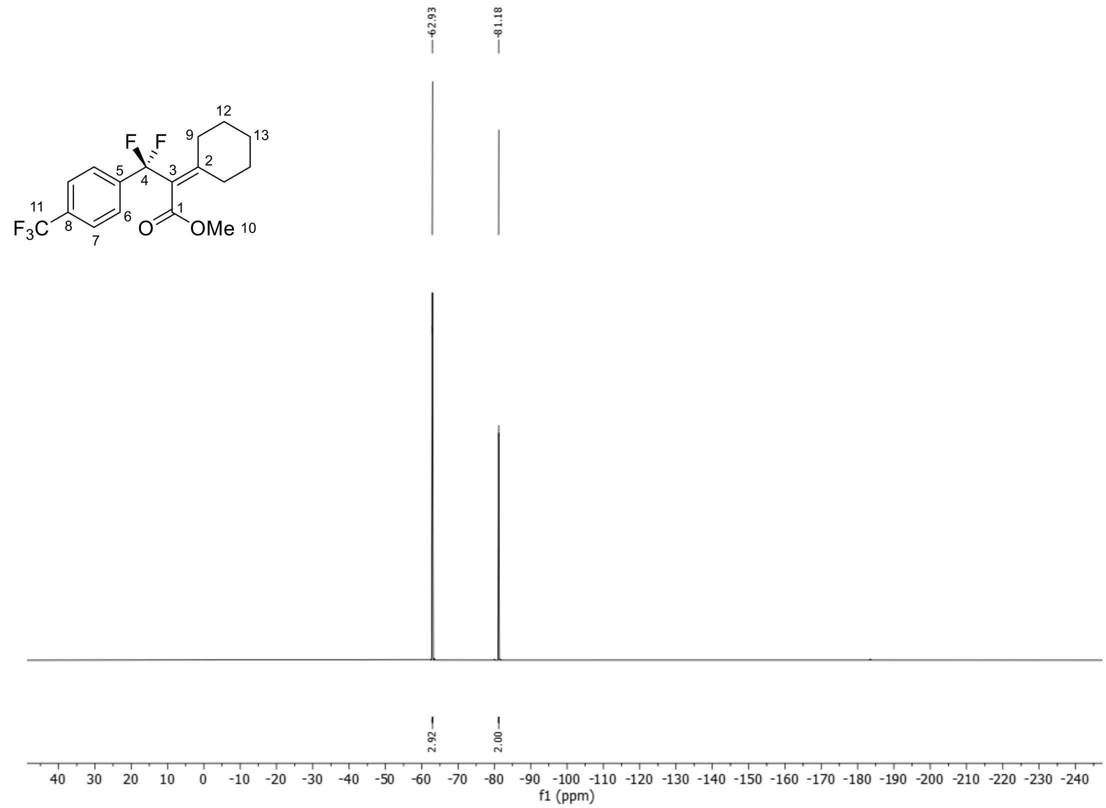

Figure S217. $^{19}F\{^1H\}$ NMR of **29** (377 MHz, 299 K, $CDCl_3$).

1,1,1,4,4-Pentafluoro-2-phenyl-4-(4-(trifluoromethyl)phenyl)butane-2,3-diol (30)

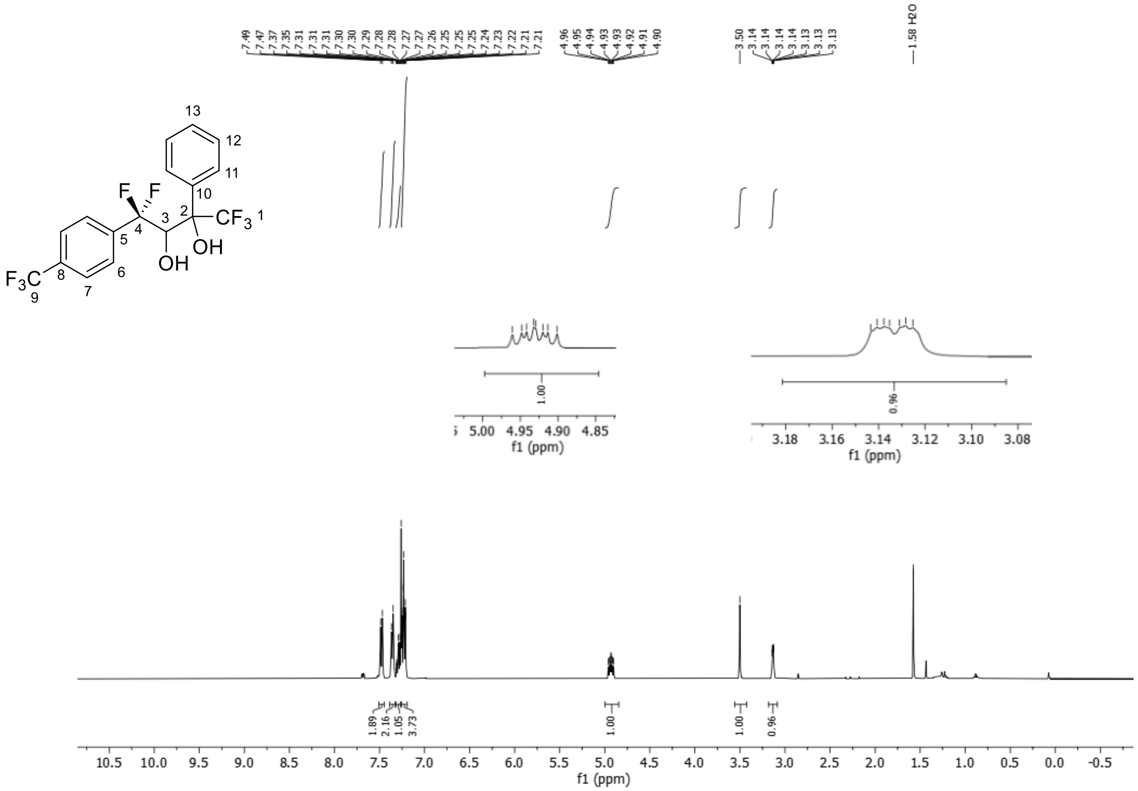

Figure S218. ^{1}H NMR of **30** (400 MHz, 299 K, $CDCl_3$).

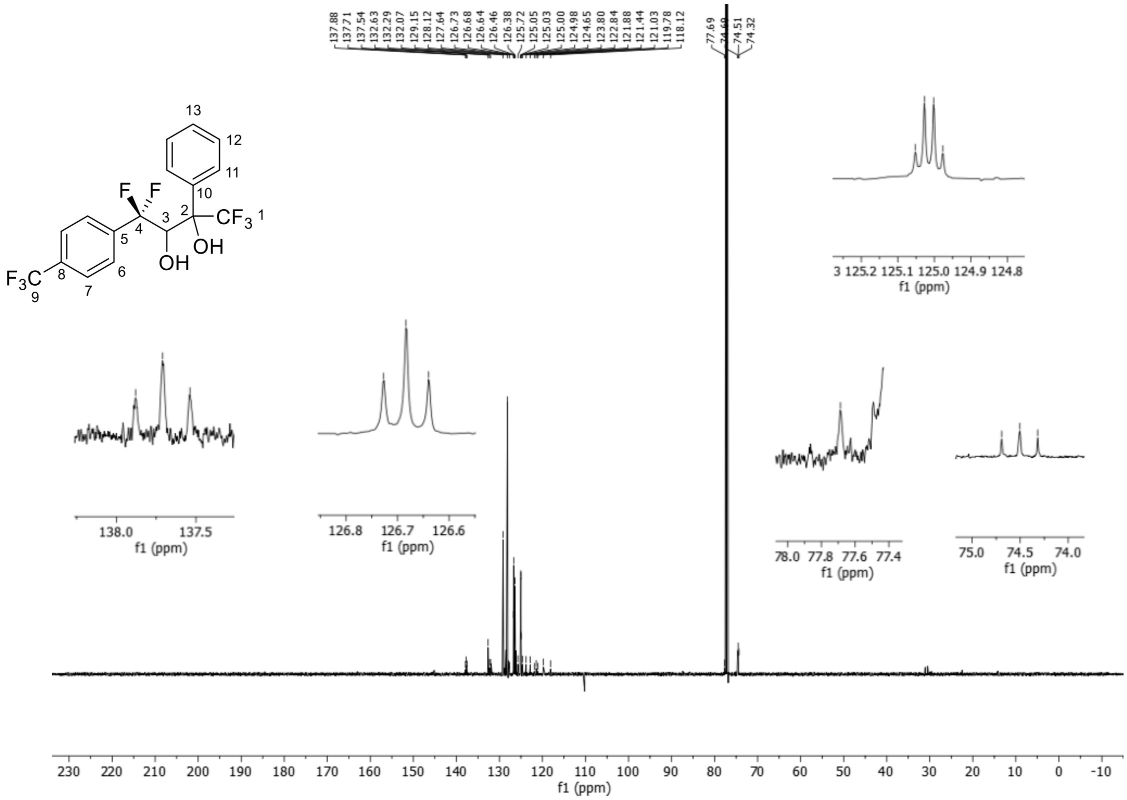

Figure S219. $^{13}C\{^{1}H\}$ NMR of **30** (126 MHz, 299 K, $CDCl_3$).

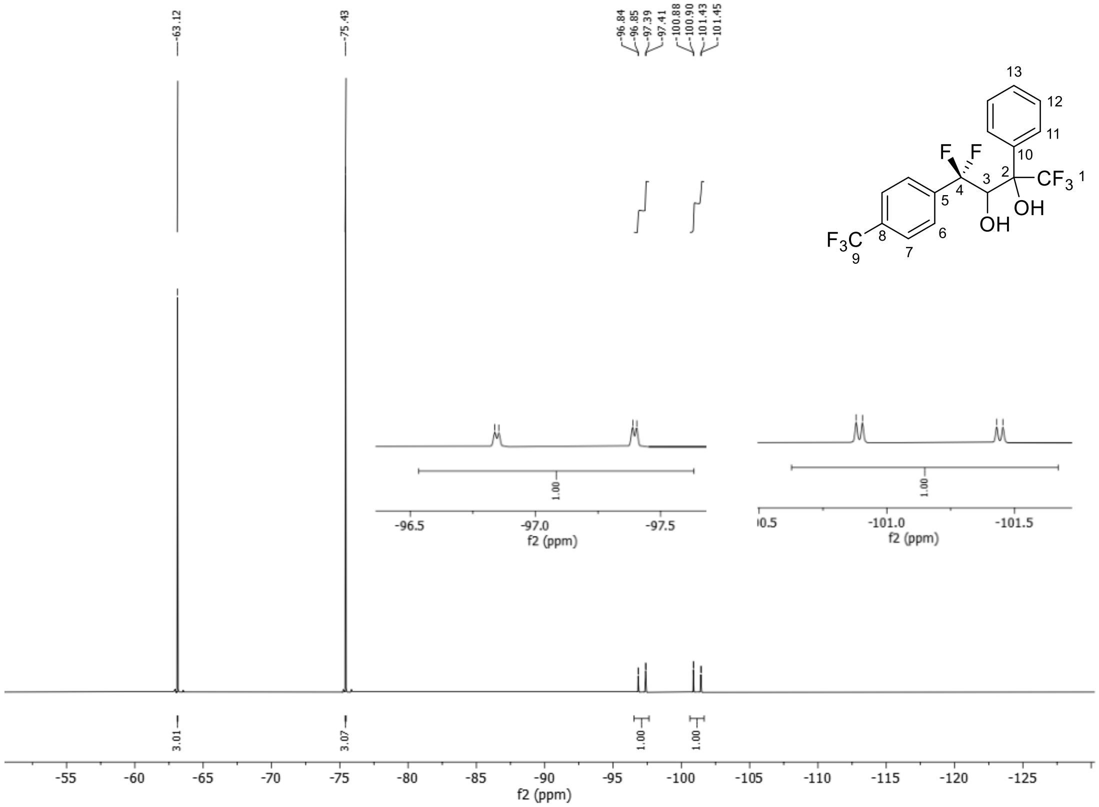

Figure S220. ^{19}F NMR of **30** (470 MHz, 299 K, $CDCl_3$).

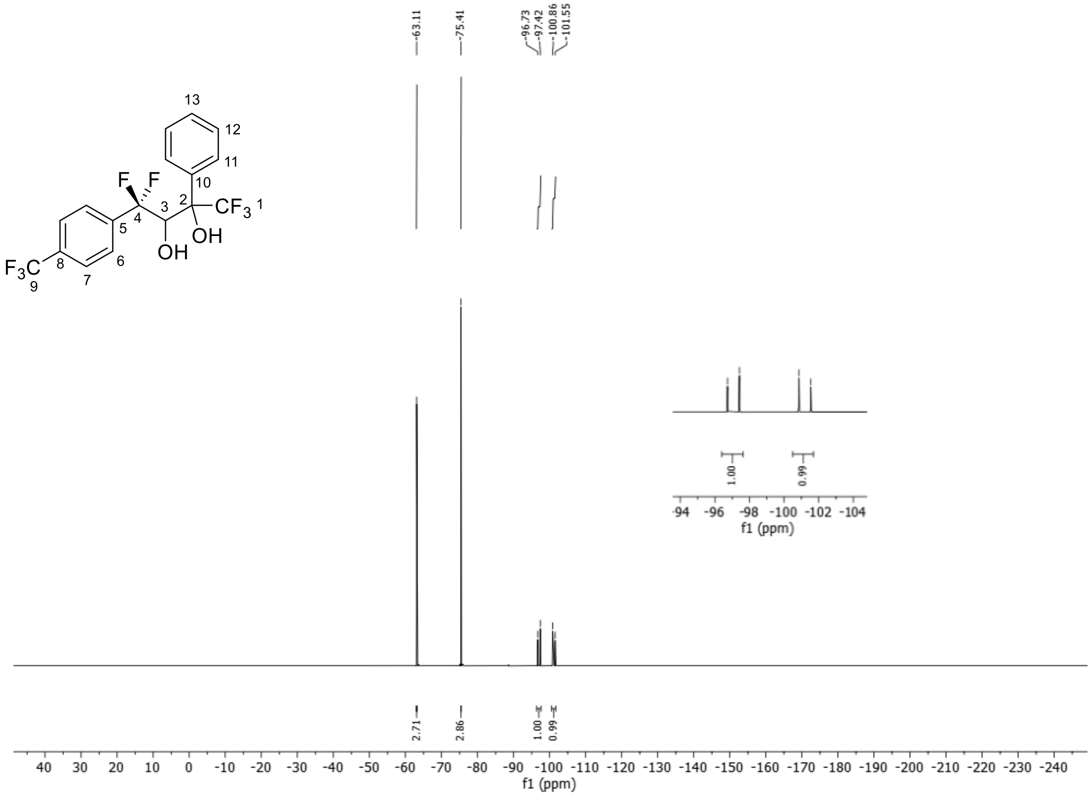

Figure S221. ^{19}F{^{1}H} NMR of **30** (377 MHz, 299 K, $CDCl_3$).

(*E*)-1-(1,1,4-Trifluoro-3-phenylbut-3-en-1-yl)-4-(trifluoromethyl)benzene (31)

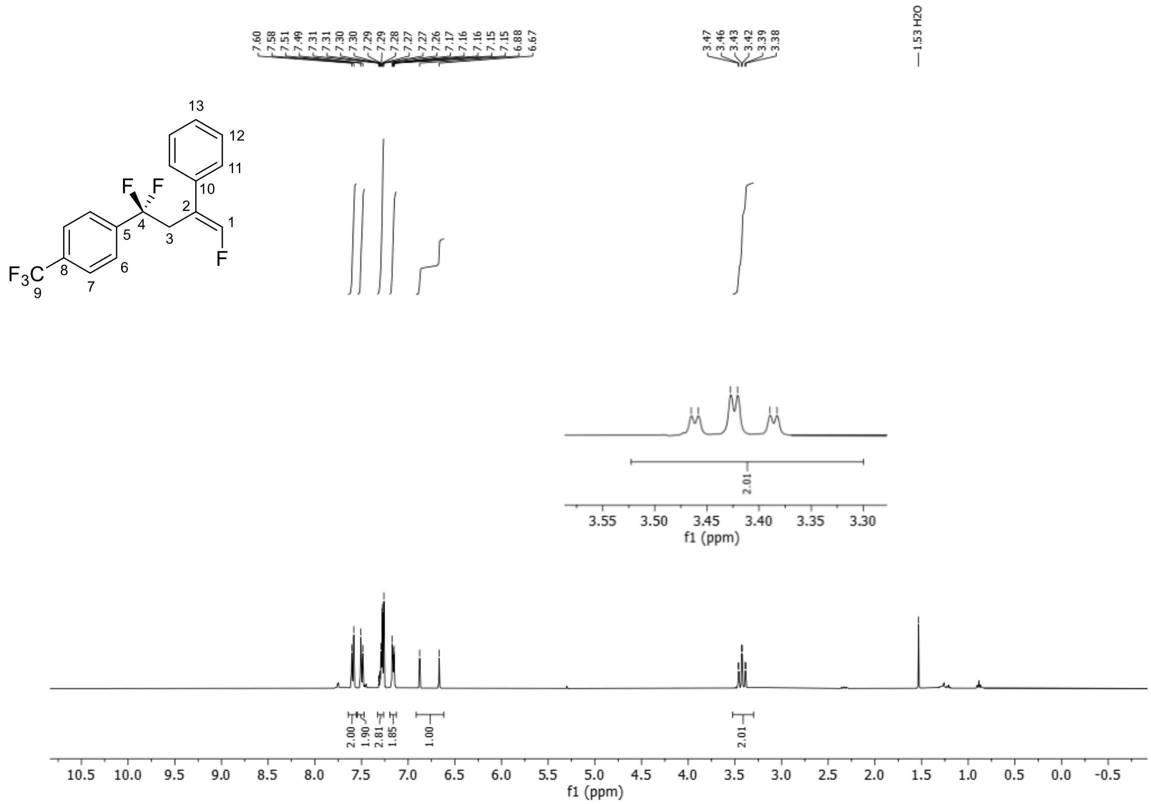

Figure S222. ^{1}H NMR of **31** (400 MHz, 299 K, $CDCl_3$).

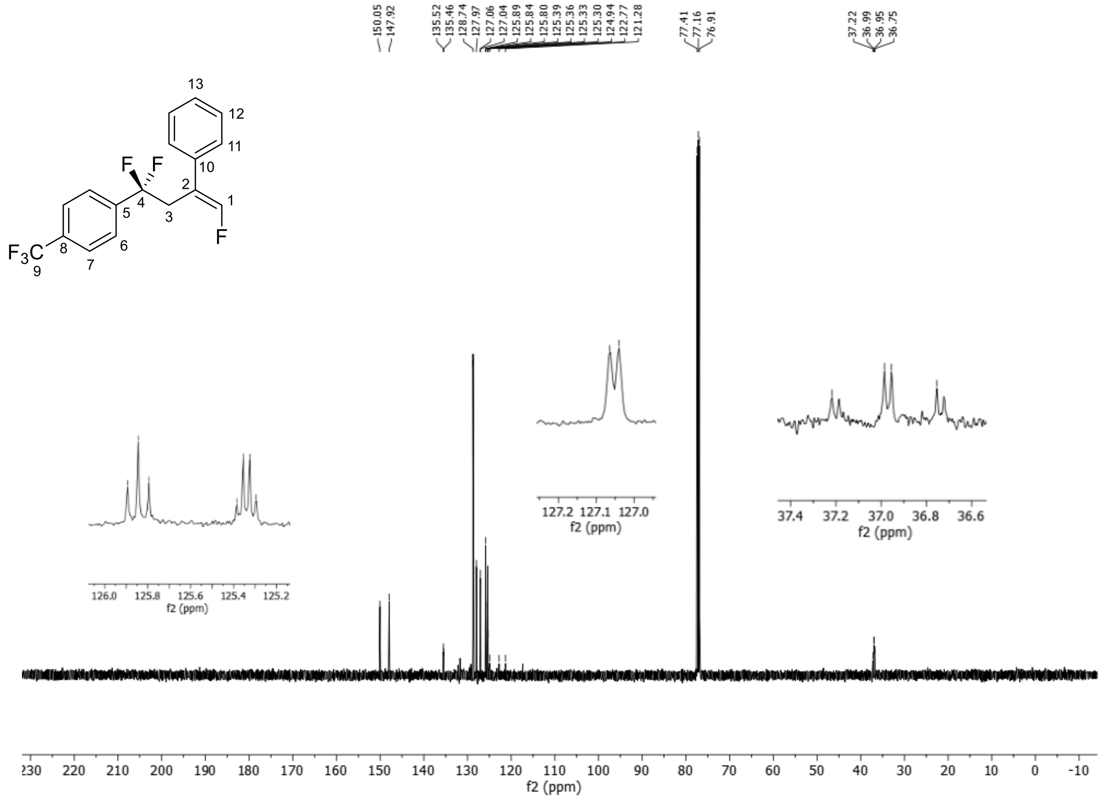

Figure S223. $^{13}C\{^{1}H\}$ NMR of **31** (126 MHz, 299 K, $CDCl_3$).

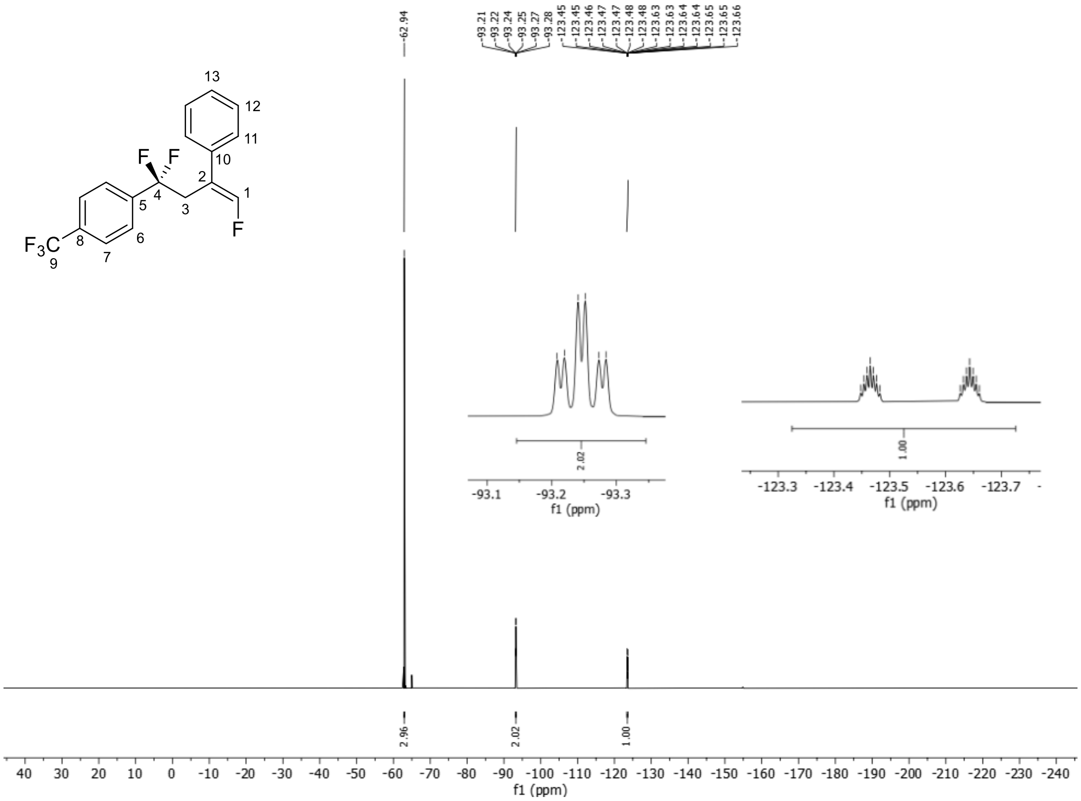

Figure S224. ^{19}F NMR of **31** (470 MHz, 299 K, $CDCl_3$).

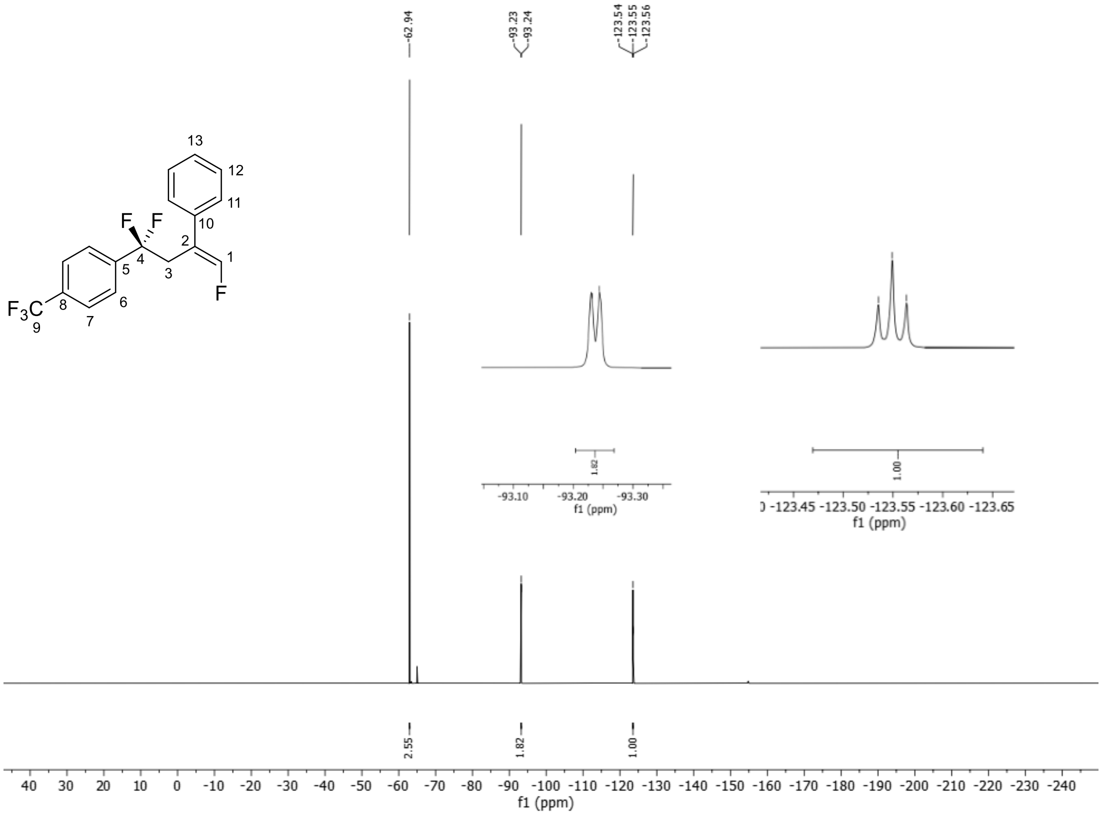

Figure S225. $^{19}F\{^1H\}$ NMR of **31** (377 MHz, 299 K, $CDCl_3$).

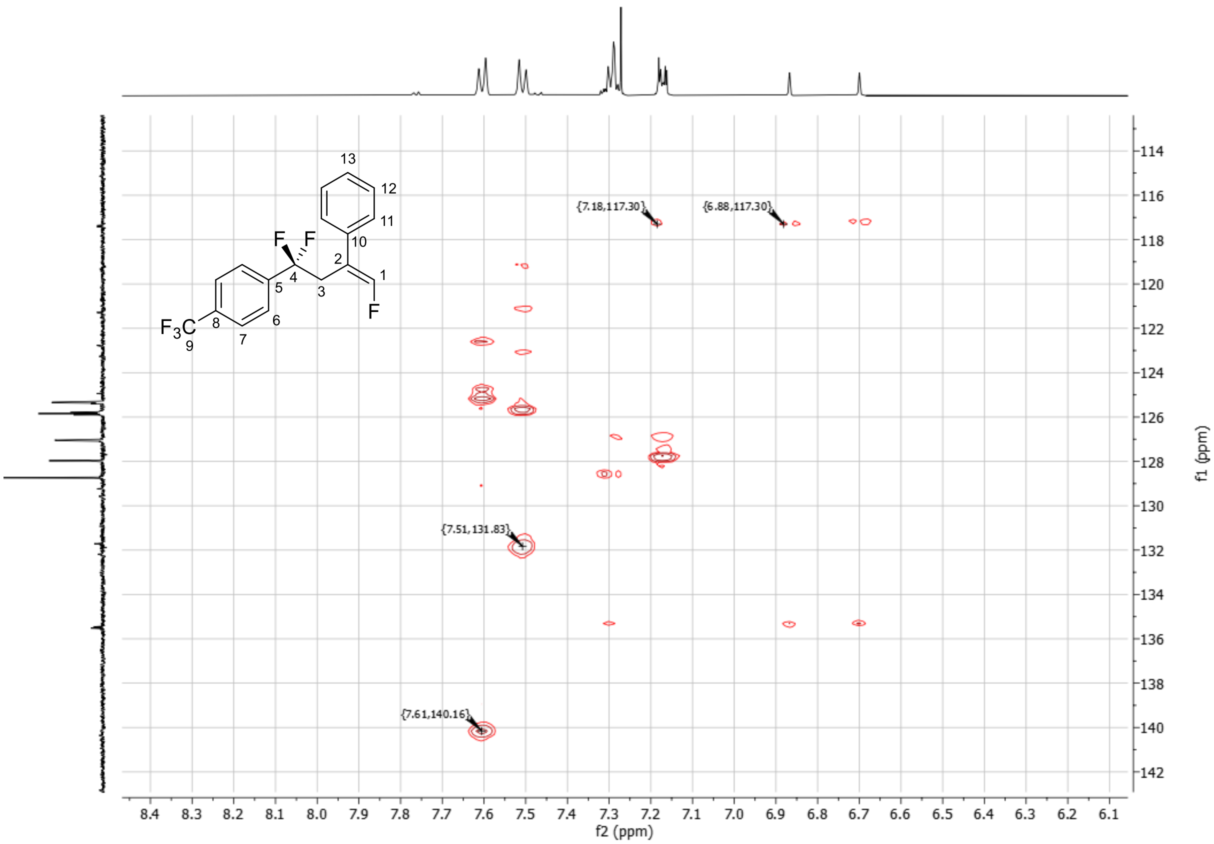

Figure S226. HMBC NMR of **31**.

Diethyl 3-(difluoro(4-(trifluoromethyl)phenyl)methyl)oxirane-2,2-dicarboxylate (32)

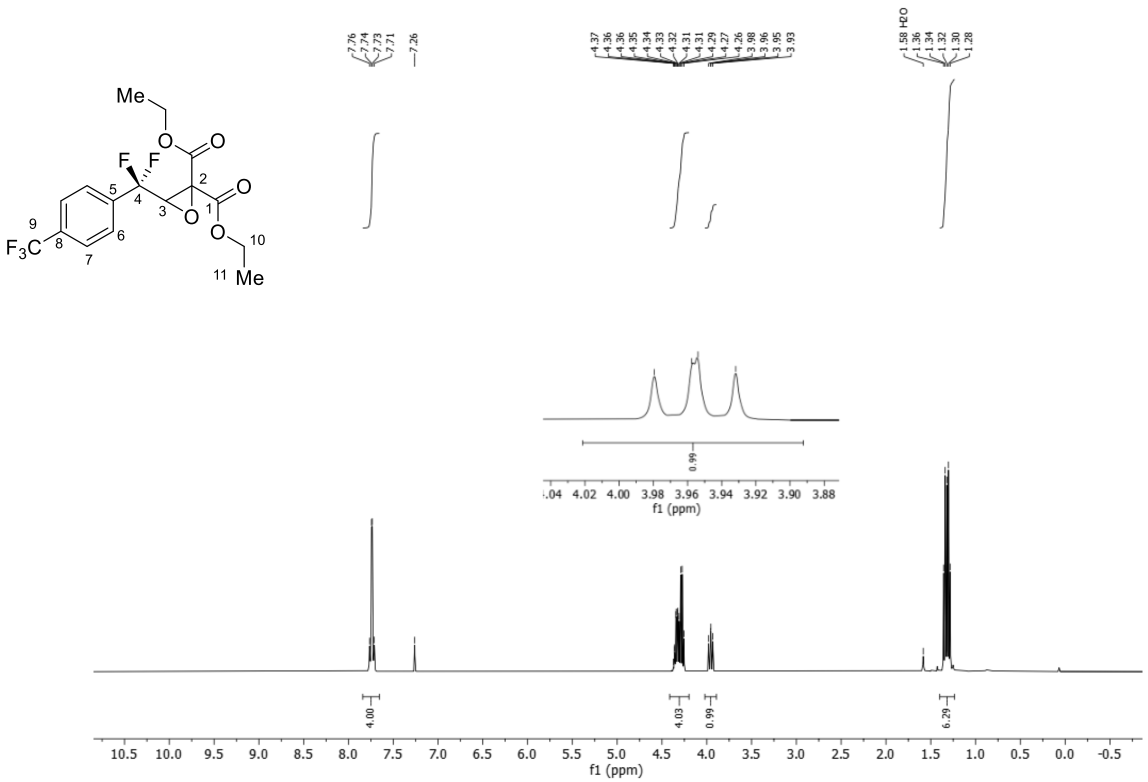

Figure S227. ^{1}H NMR of **32** (400 MHz, 299 K, $CDCl_3$).

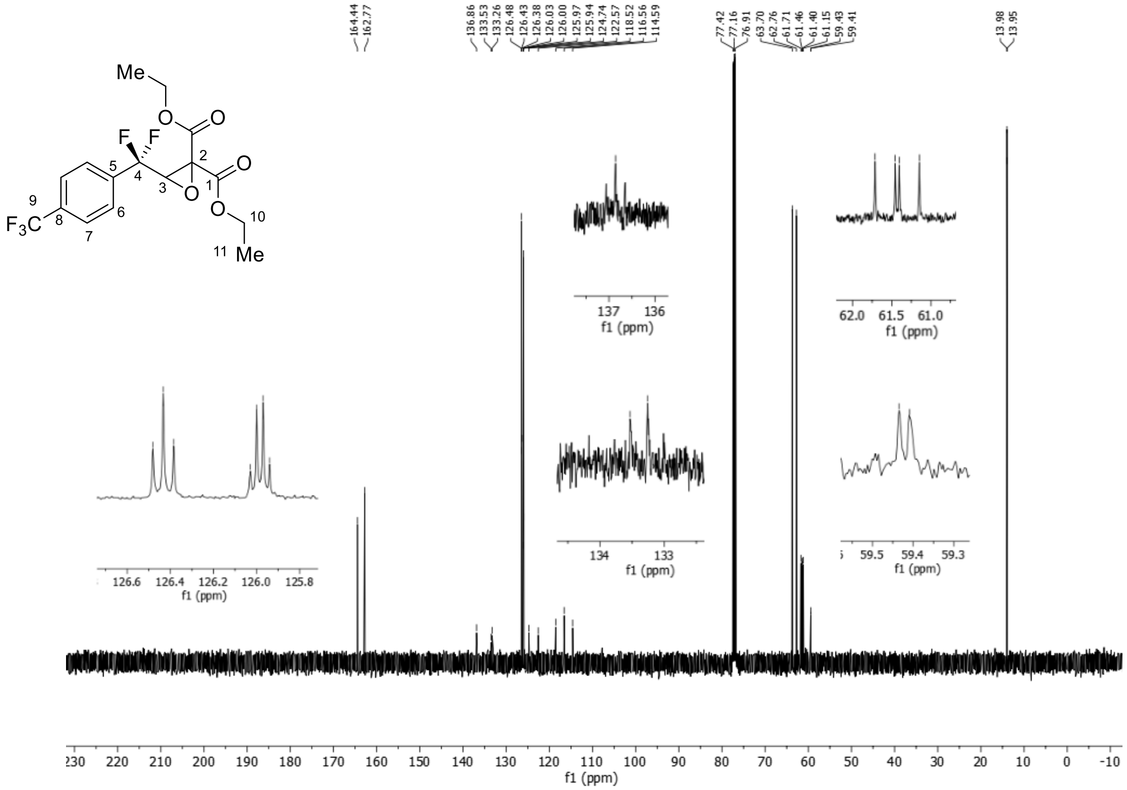

Figure S228. $^{13}C\{^{1}H\}$ NMR of **32** (126 MHz, 299 K, $CDCl_3$).

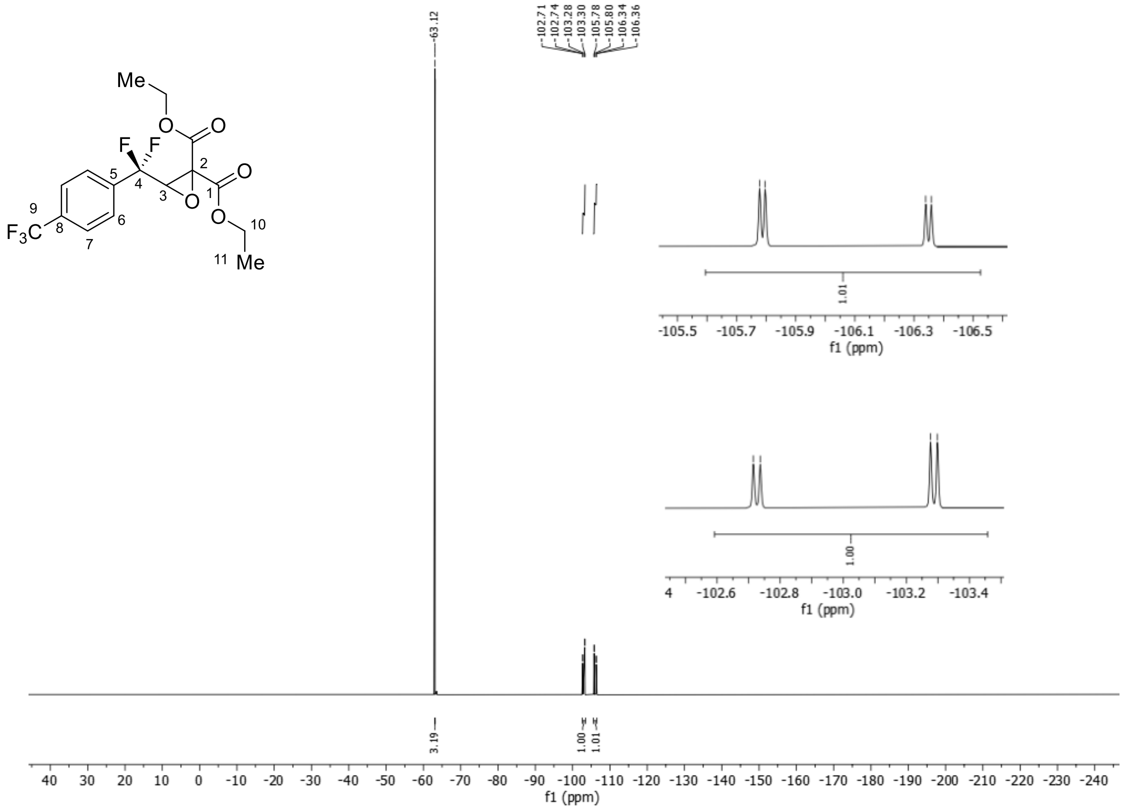

Figure S229. ^{19}F NMR of **32** (470 MHz, 299 K, $CDCl_3$).

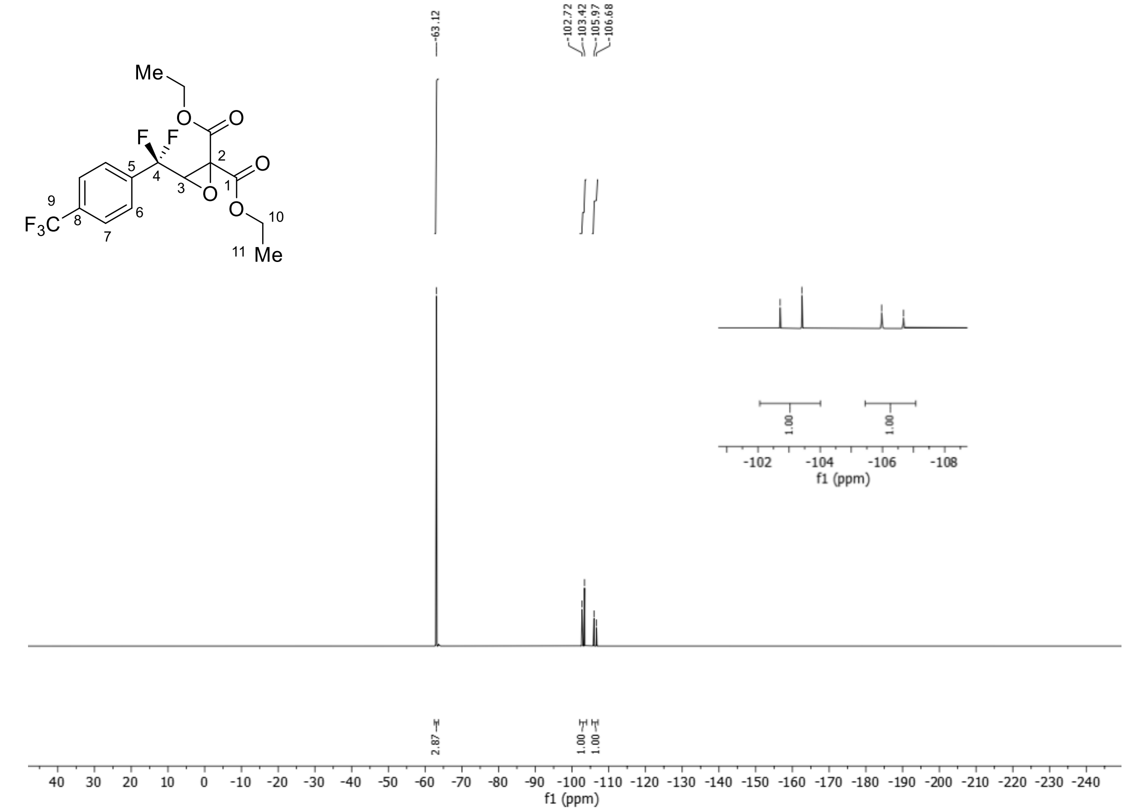

Figure S230. $^{19}F\{^1H\}$ NMR of **32** (377 MHz, 299 K, $CDCl_3$).

(*Z*)-4-Fluoro-2-methyl-4-(4-(trifluoromethyl)phenyl)but-3-en-1-ol (33)

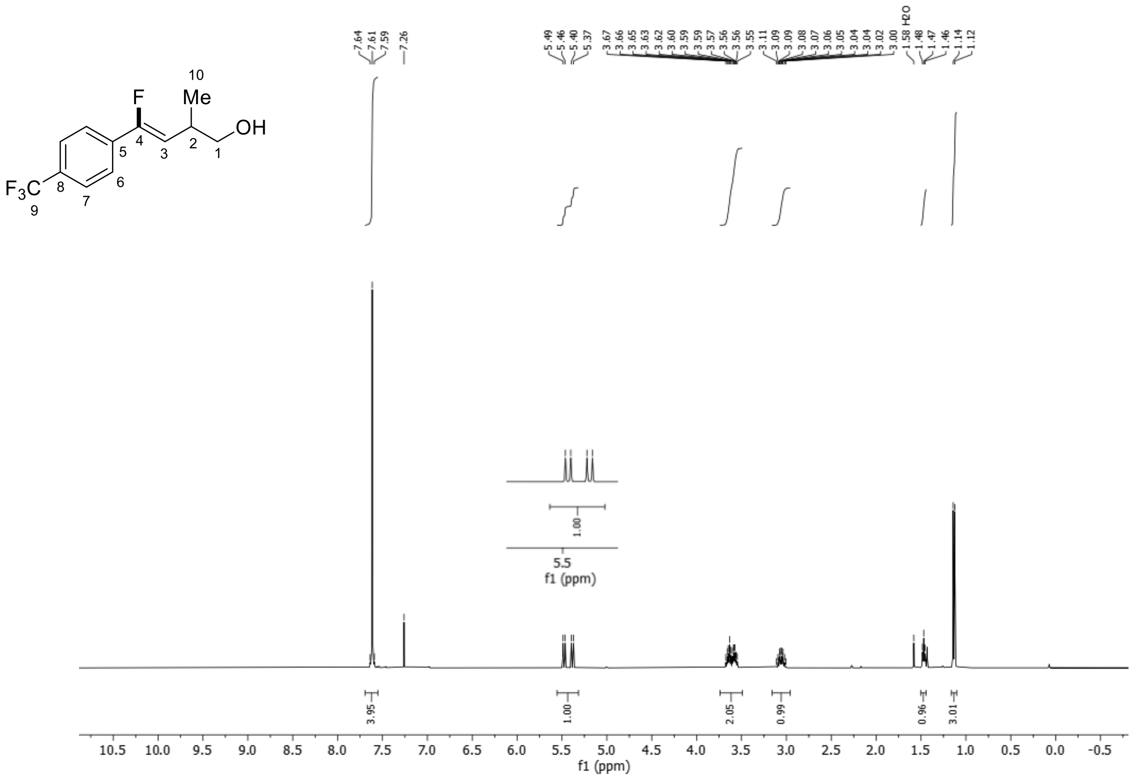

Figure S231. ^{1}H NMR of **33** (400 MHz, 299 K, $CDCl_3$).

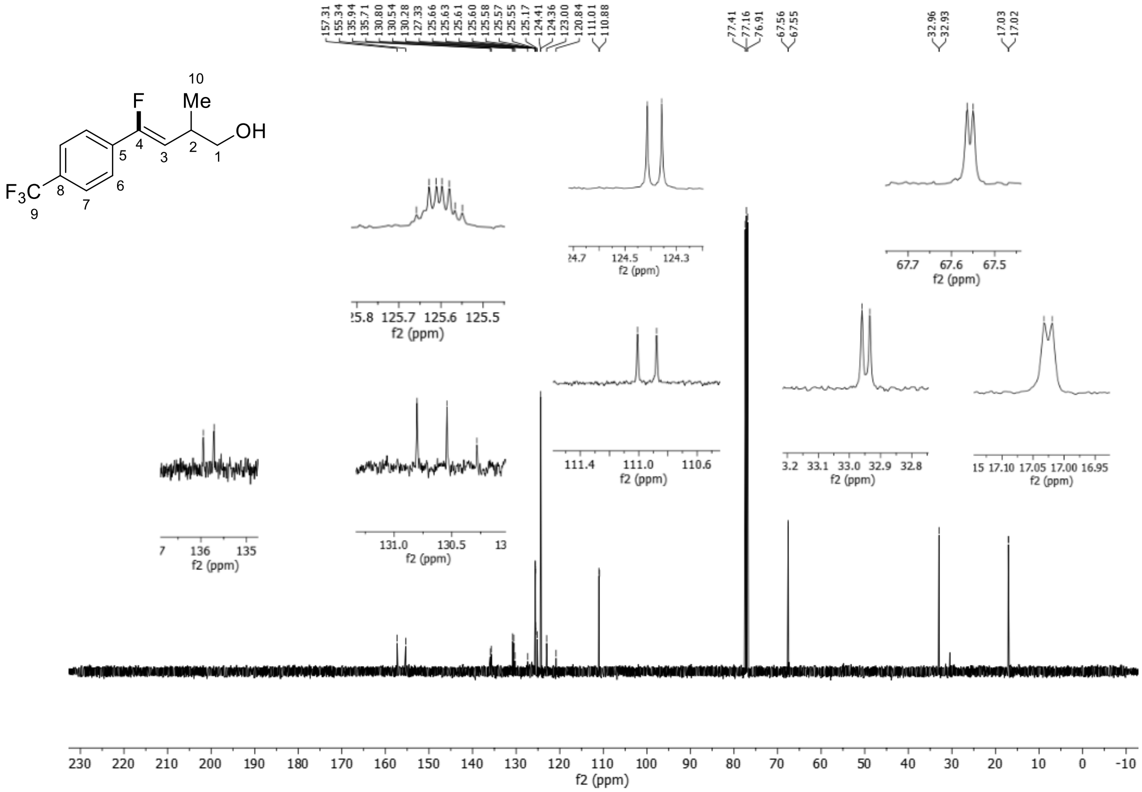

Figure S232. $^{13}C\{^{1}H\}$ NMR of **33** (126 MHz, 299 K, $CDCl_3$).

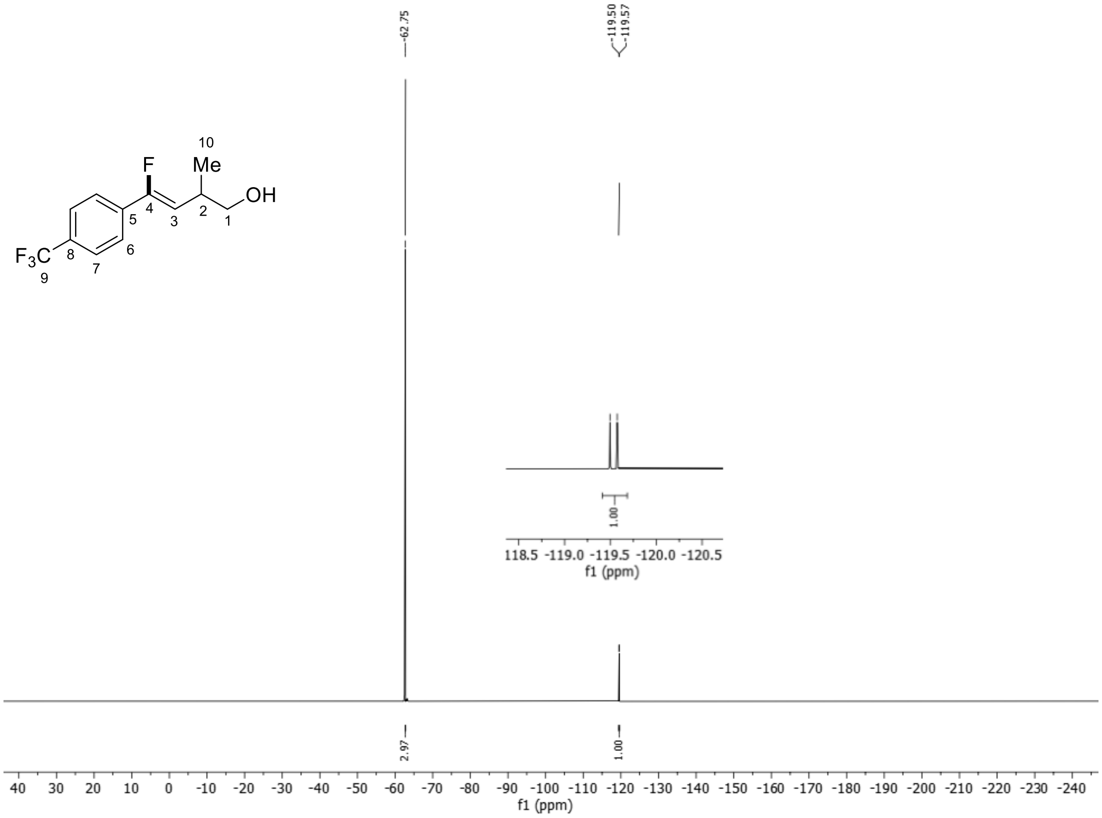

Figure S233. ^{19}F NMR of **33** (470 MHz, 299 K, $CDCl_3$).

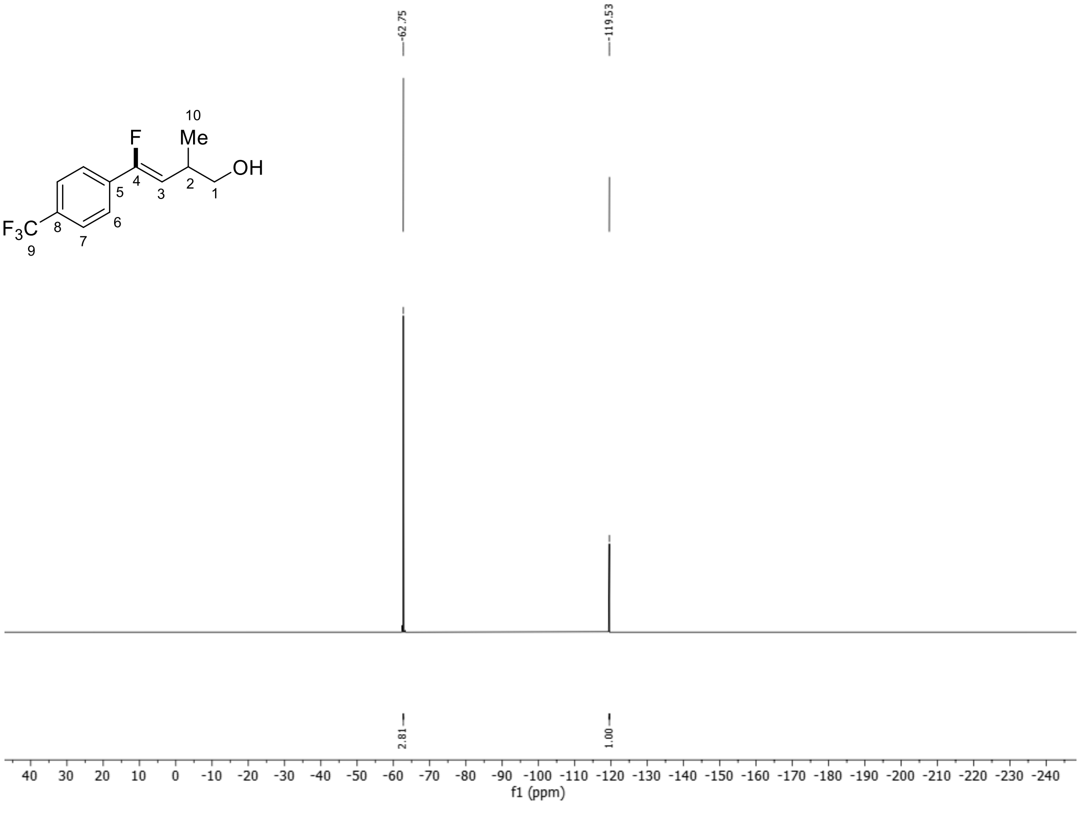

Figure S234. $^{19}F\{^1H\}$ NMR of **33** (377 MHz, 299 K, $CDCl_3$).

Diethyl 2-(2,2-difluoro-2-(4-(trifluoromethyl)phenyl)ethyl)malonate (34)

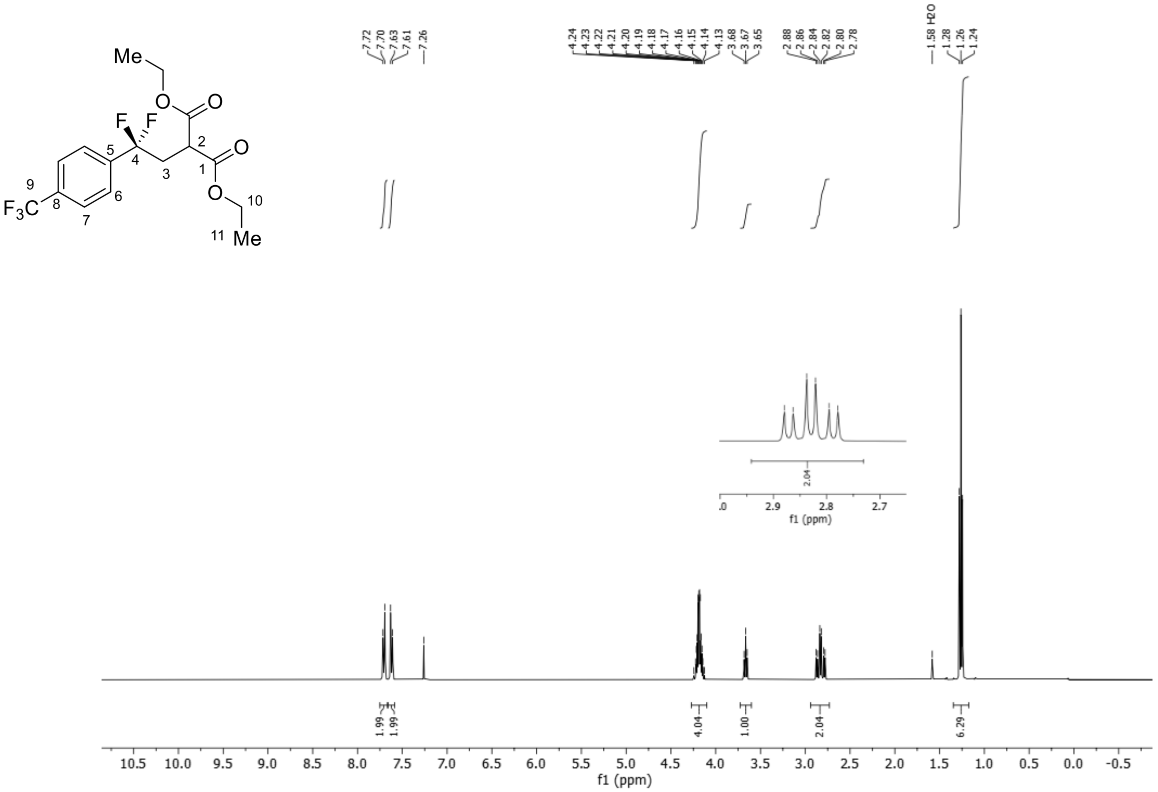

Figure S235. ^{1}H NMR of **34** (400 MHz, 299 K, $CDCl_3$).

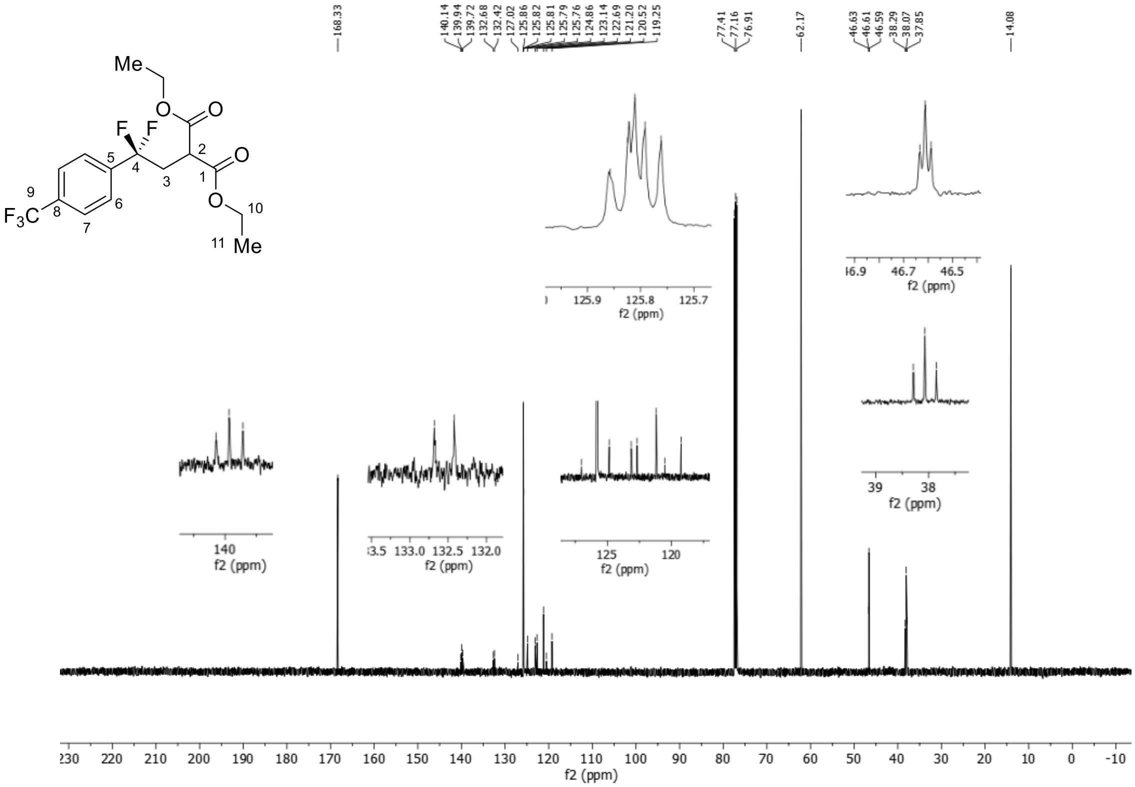

Figure S236. $^{13}C\{^{1}H\}$ NMR of **34** (126 MHz, 299 K, $CDCl_3$).

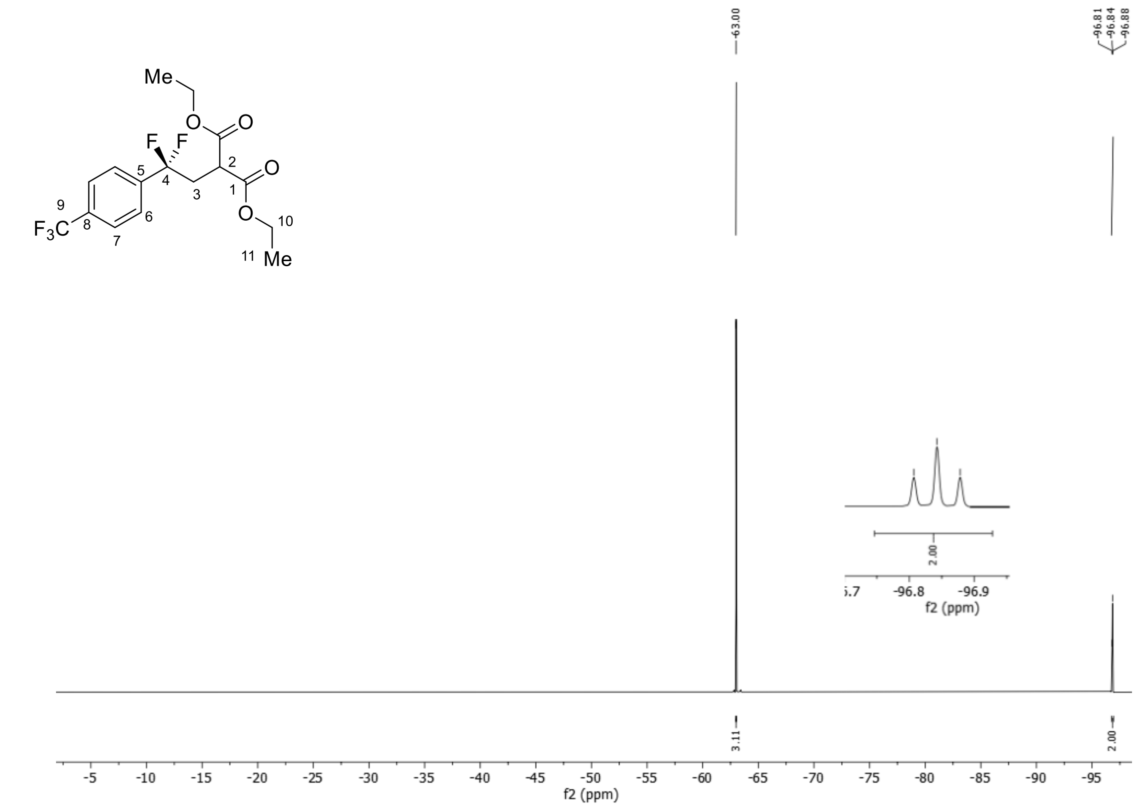

Figure S237. ^{19}F NMR of **34** (470 MHz, 299 K, $CDCl_3$).

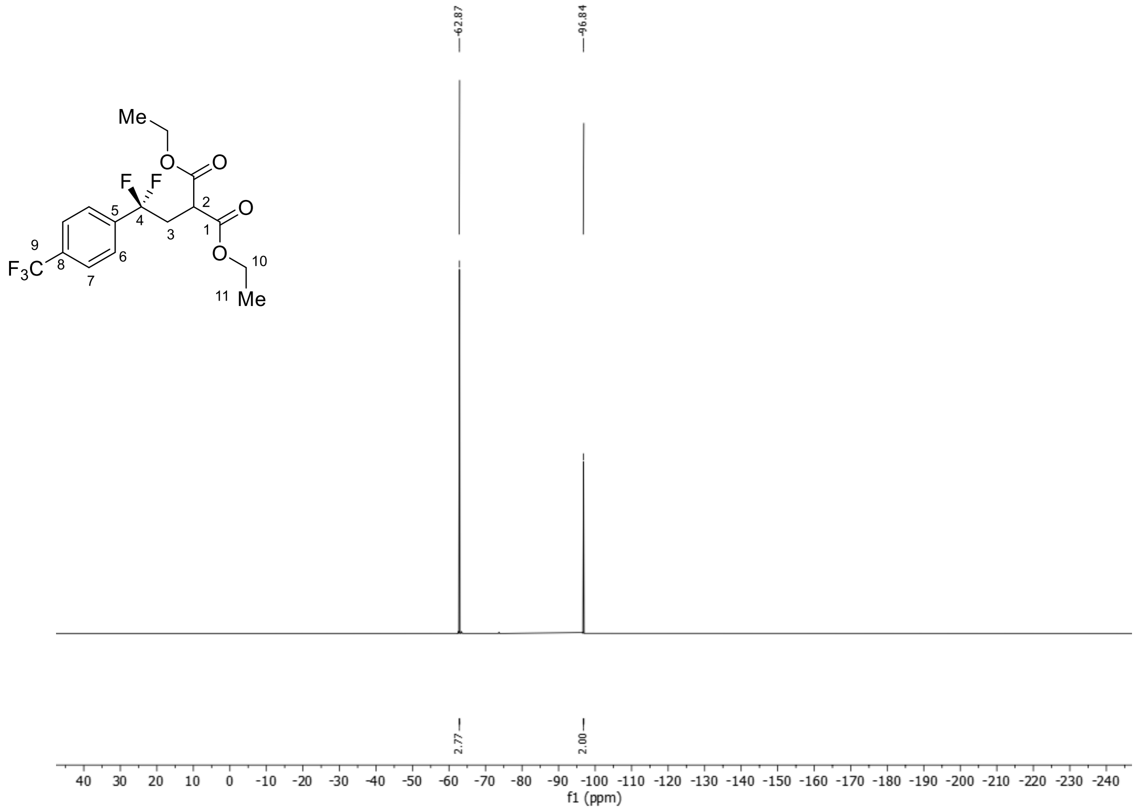

Figure S238. $^{19}F\{^1H\}$ NMR of **34** (377 MHz, 299 K, $CDCl_3$).

Diethyl 2-(difluoro(4-(trifluoromethyl)phenyl)methyl)cyclopropane-1,1-dicarboxylate (35)

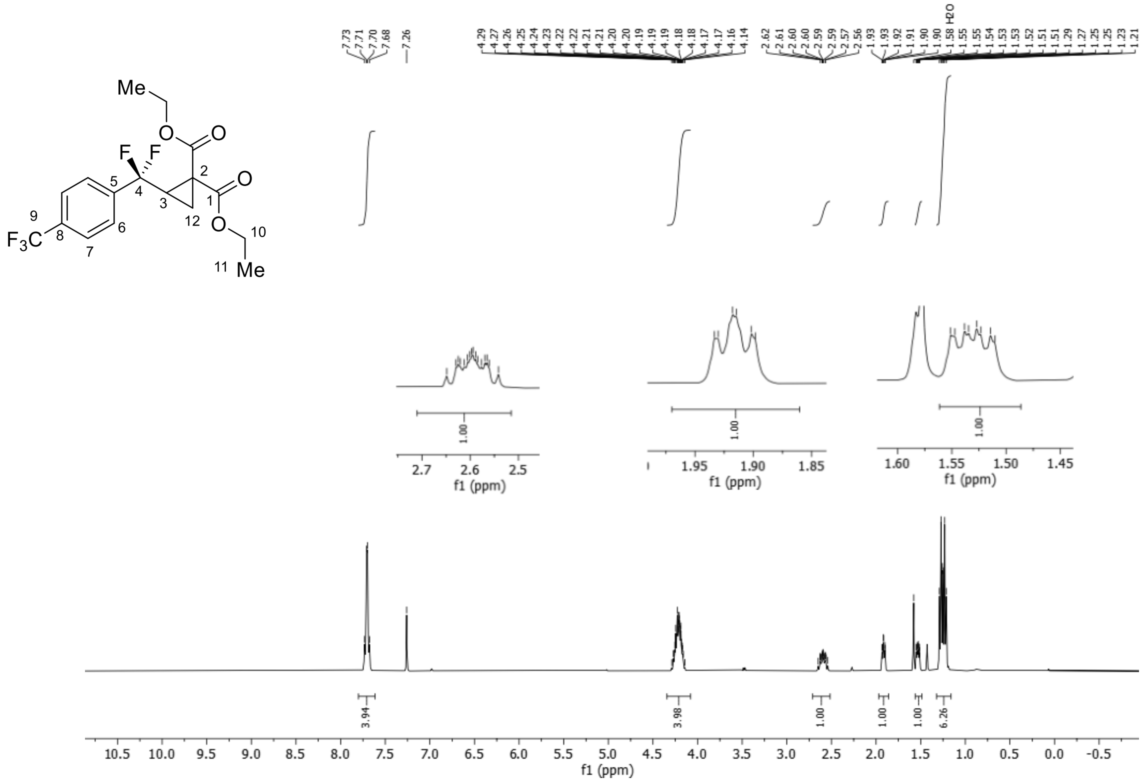

Figure S239. ^{1}H NMR of **35** (400 MHz, 299 K, $CDCl_3$).

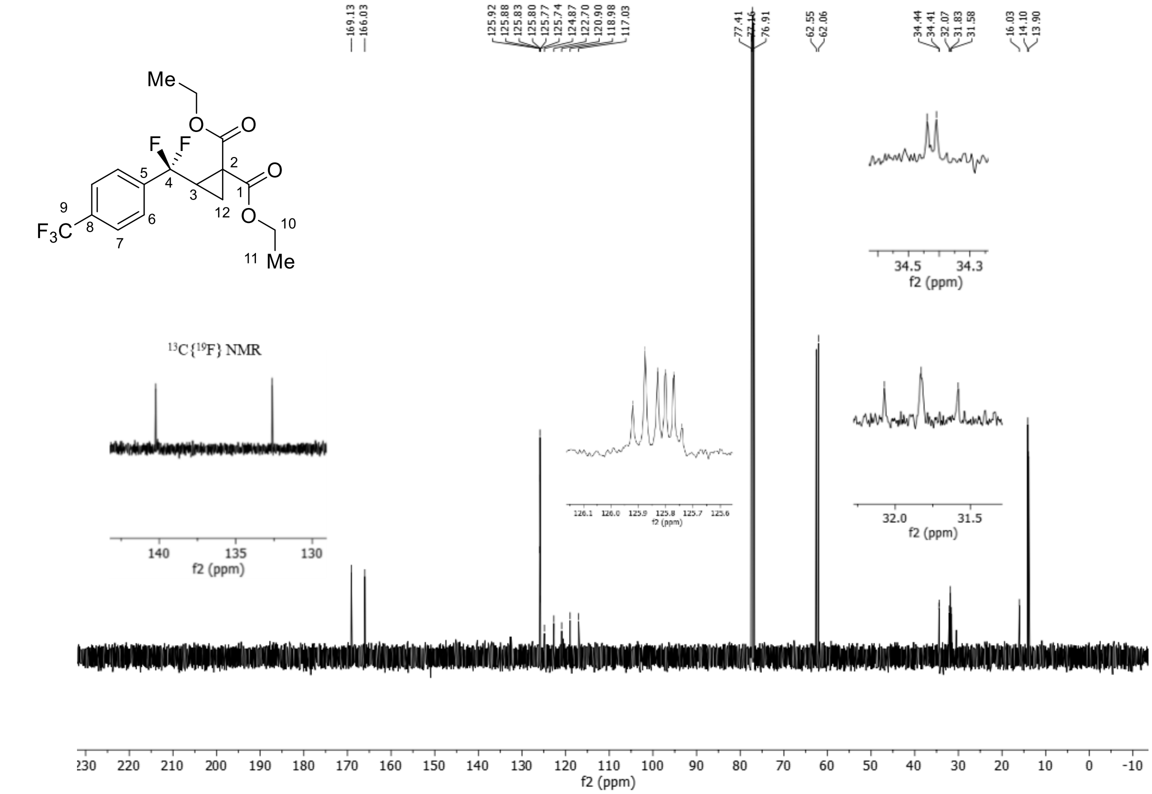

Figure S240. $^{13}C\{^{1}H\}$ NMR of **35** (126 MHz, 299 K, $CDCl_3$).

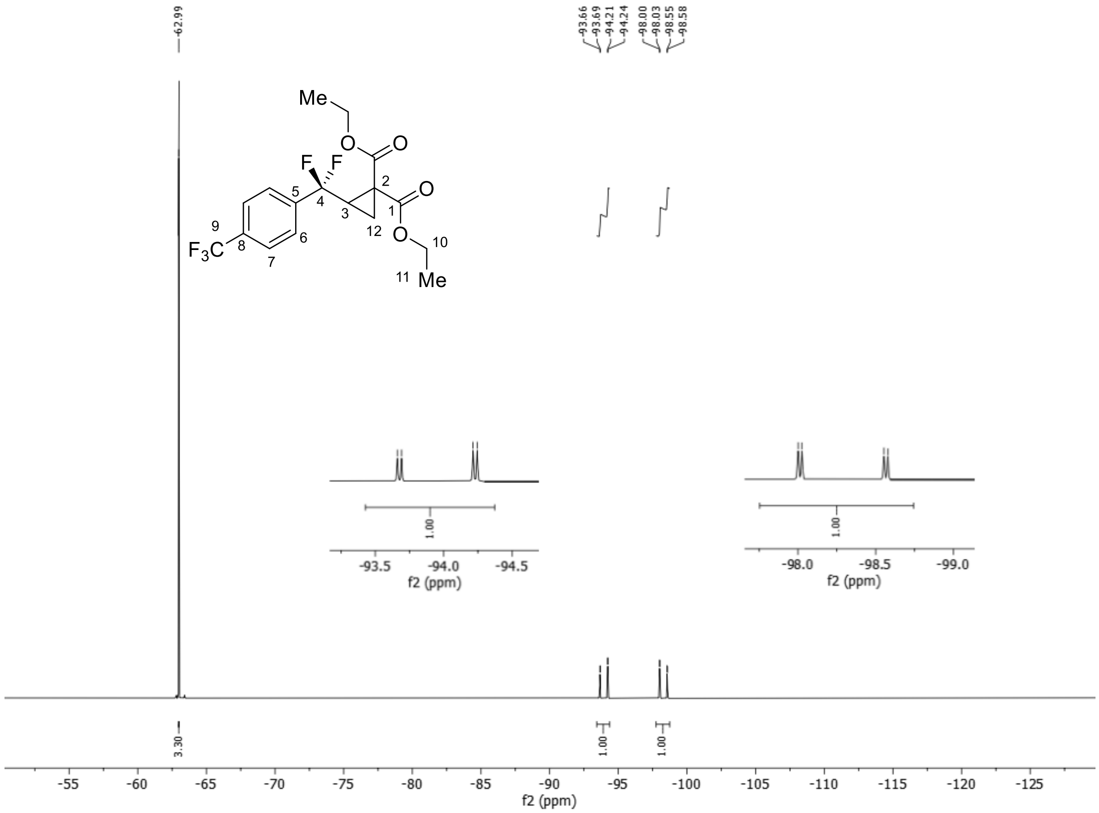

Figure S241. ^{19}F NMR of **35** (470 MHz, 299 K, $CDCl_3$).

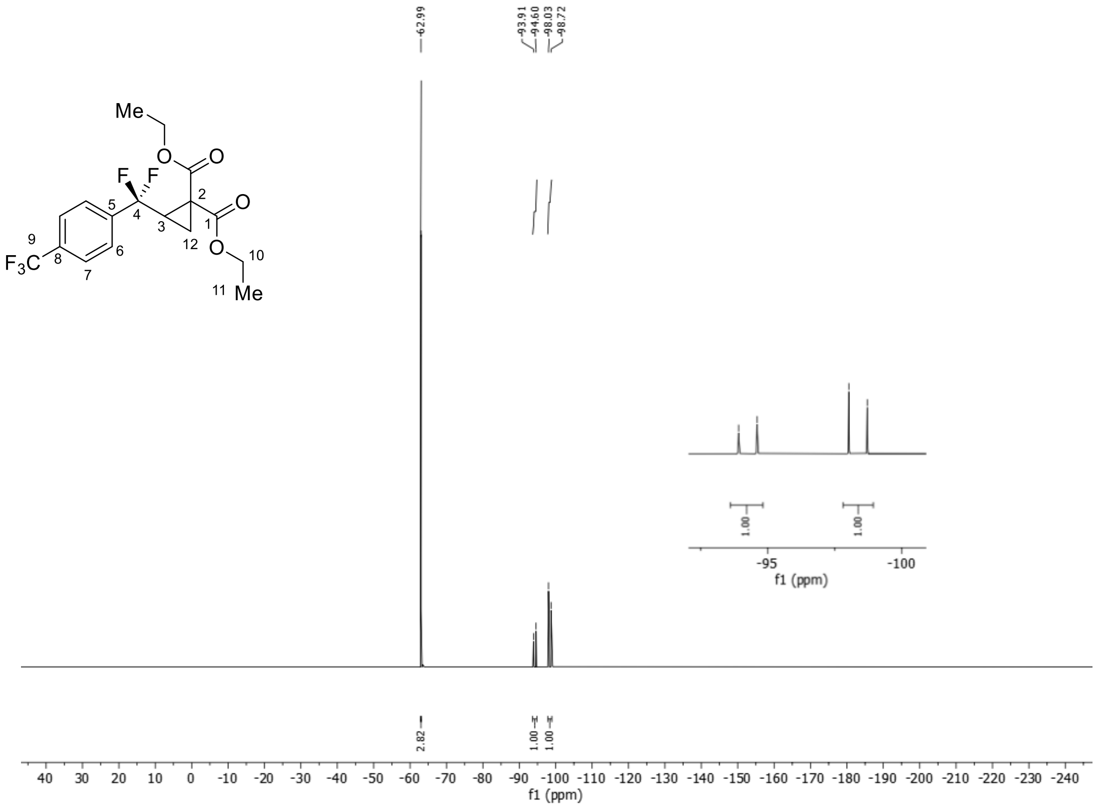

Figure S242. $^{19}F\{^1H\}$ NMR of **35** (377 MHz, 299 K, $CDCl_3$).

1-(2-Hexyl-3-phenyl-3-(trifluoromethyl)cycloprop-1-en-1-yl)-4-(trifluoromethyl)benzene (S36)

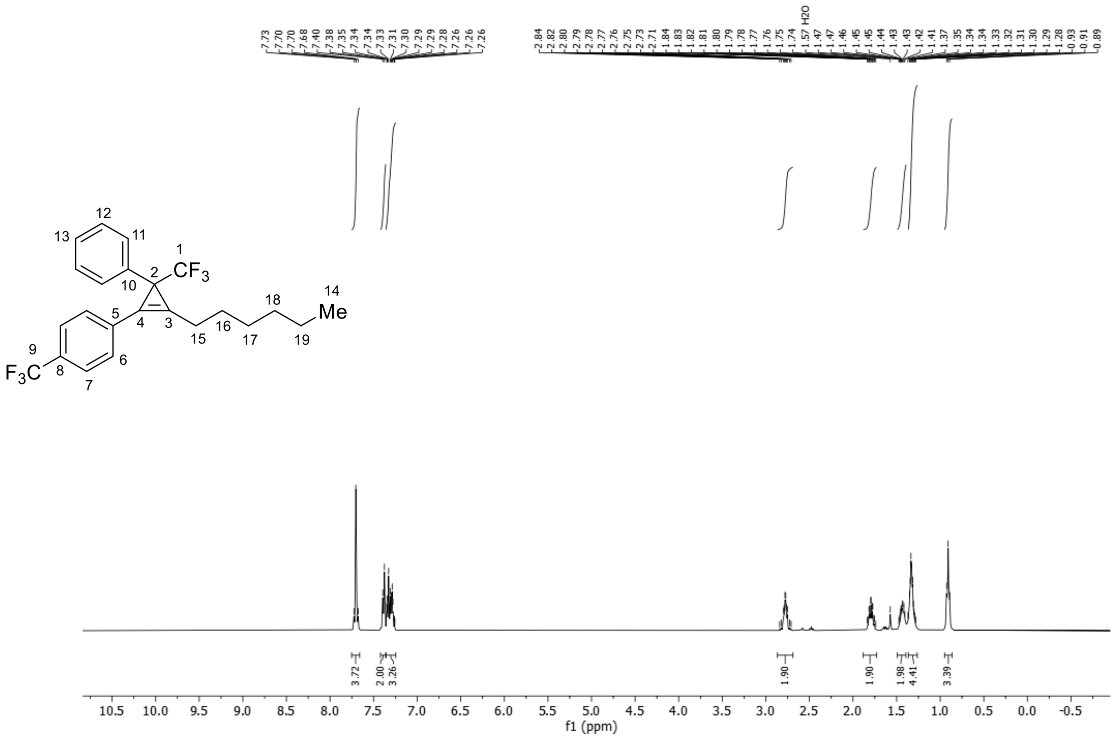

Figure S243. ^{1}H NMR of **S36** (400 MHz, 299 K, $CDCl_3$).

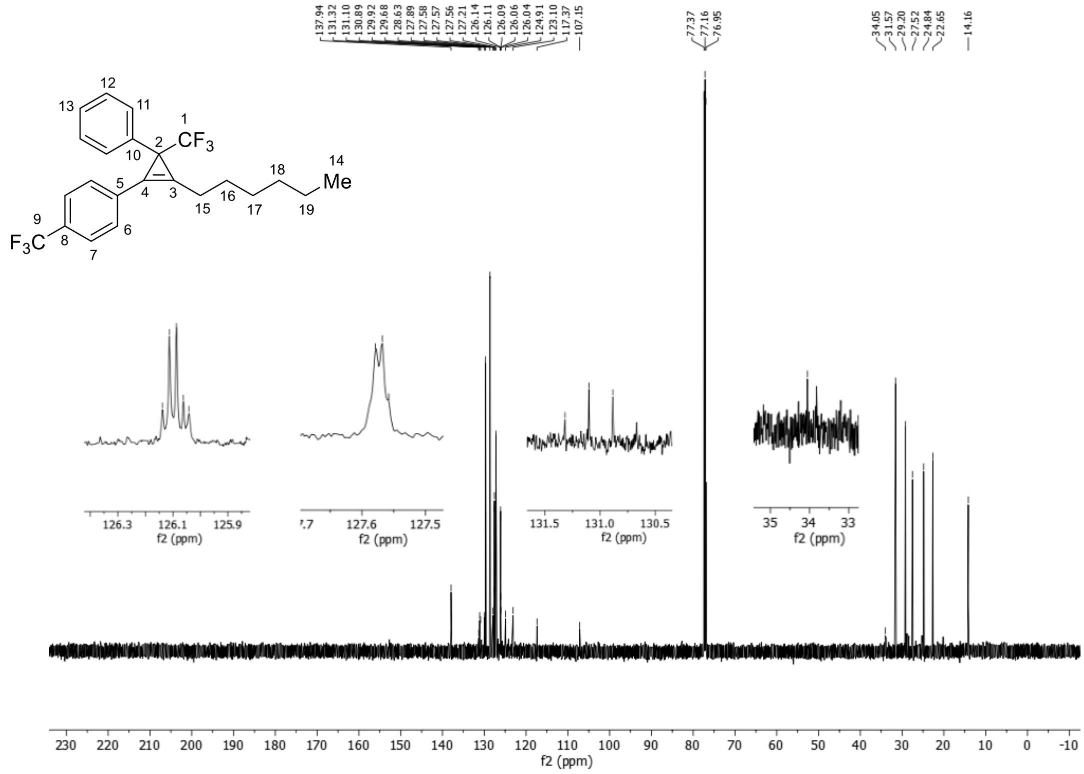

Figure S244. $^{13}C\{^1H\}$ NMR of **S36** (151 MHz, 299 K, $CDCl_3$).

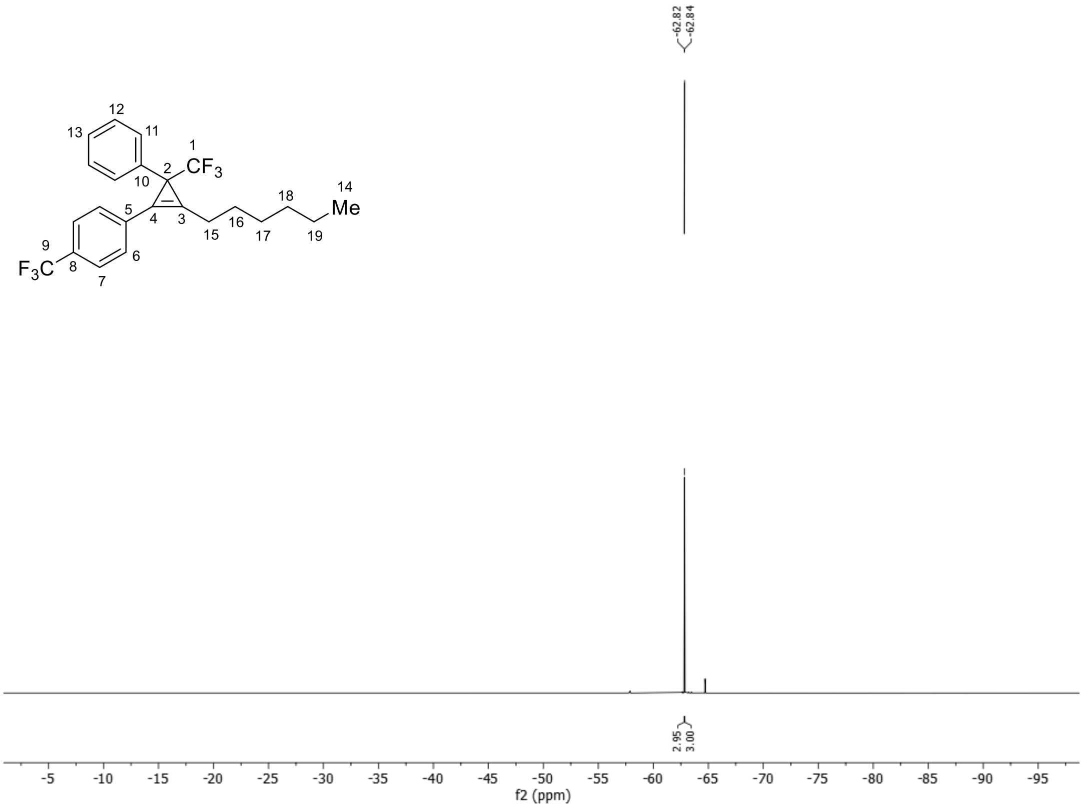

Figure S245. ^{19}F NMR of **S36** (564 MHz, 299 K, $CDCl_3$).

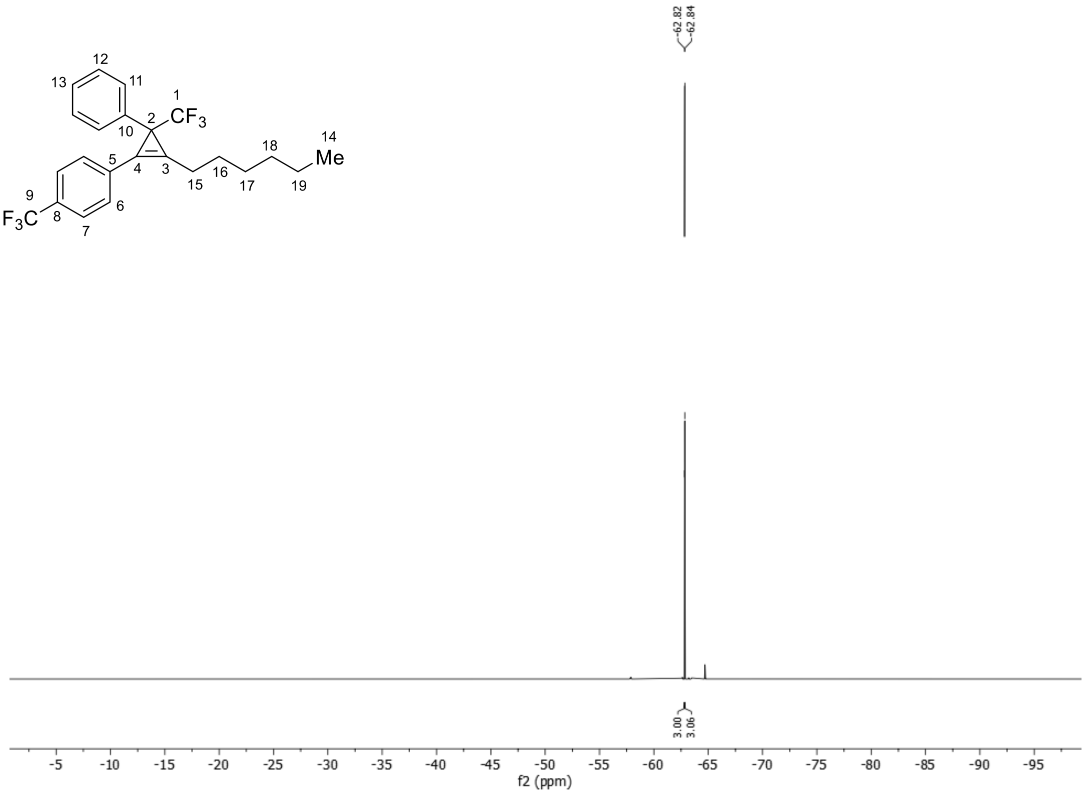

Figure S246. ^{19}F{^{1}H} NMR of **S36** (564 MHz, 299 K, $CDCl_3$).

Methyl 4-(2-hexyl-3-phenyl-3-(trifluoromethyl)cycloprop-1-en-1-yl)benzoate (S37)

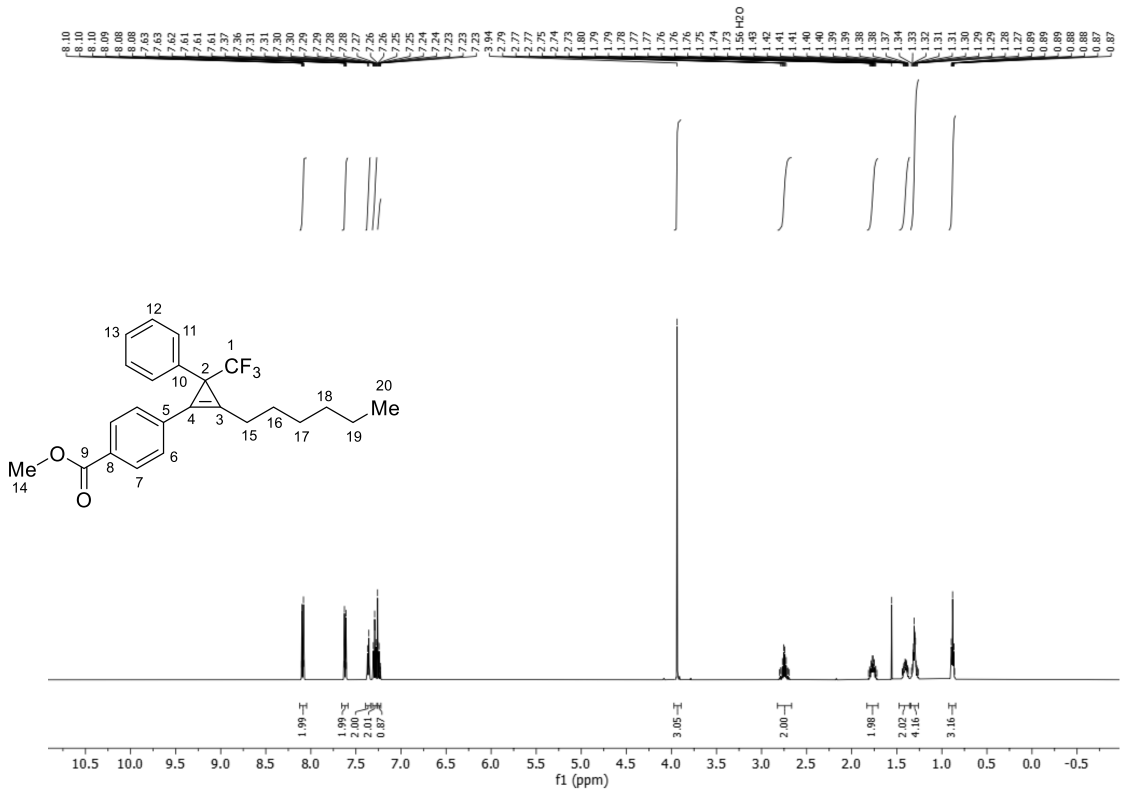

Figure S247. ^{1}H NMR of **S37** (500 MHz, 299 K, $CDCl_3$).

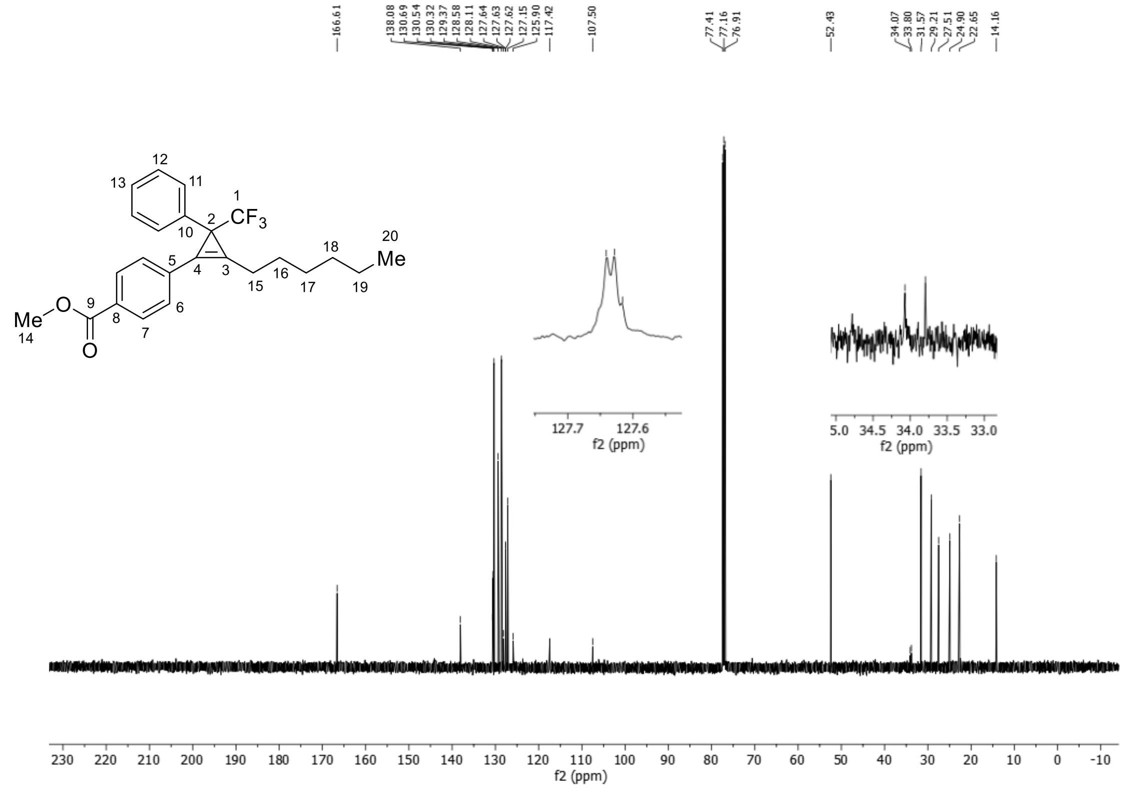

Figure S248. $^{13}C\{^{1}H\}$ NMR of **S37** (126 MHz, 299 K, $CDCl_3$).

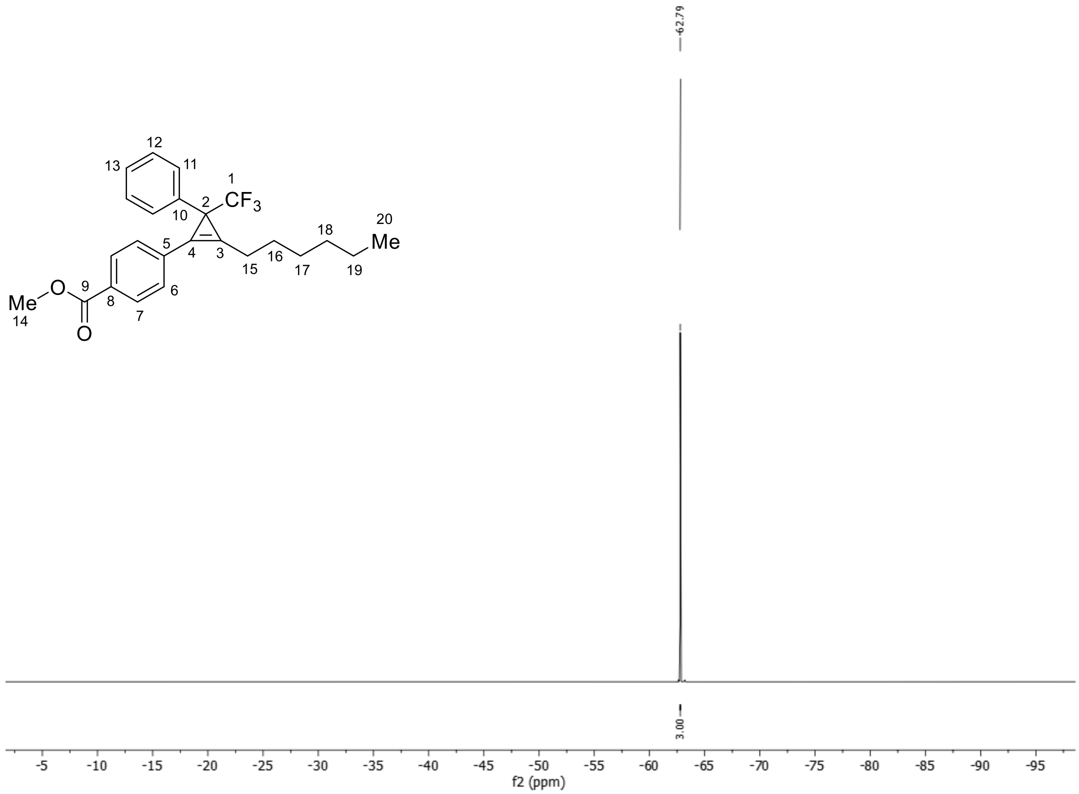

Figure S249. ^{19}F NMR of **S37** (470 MHz, 299 K, $CDCl_3$).

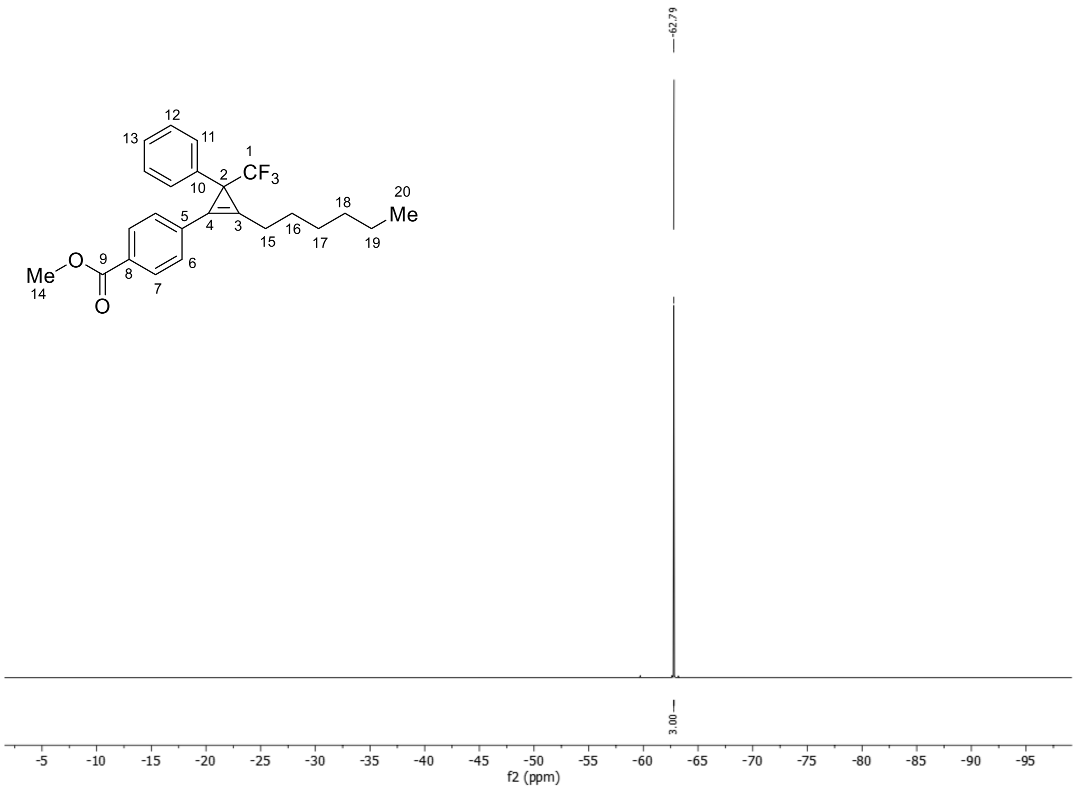

Figure S250. ^{19}F{^{1}H} NMR of **S37** (470 MHz, 299 K, $CDCl_3$).

1-Methyl-4-(2-methyl-1-(trifluoromethyl)-3-(4-(trifluoromethyl)phenyl)cycloprop-2-en-1-yl)benzene (S38)

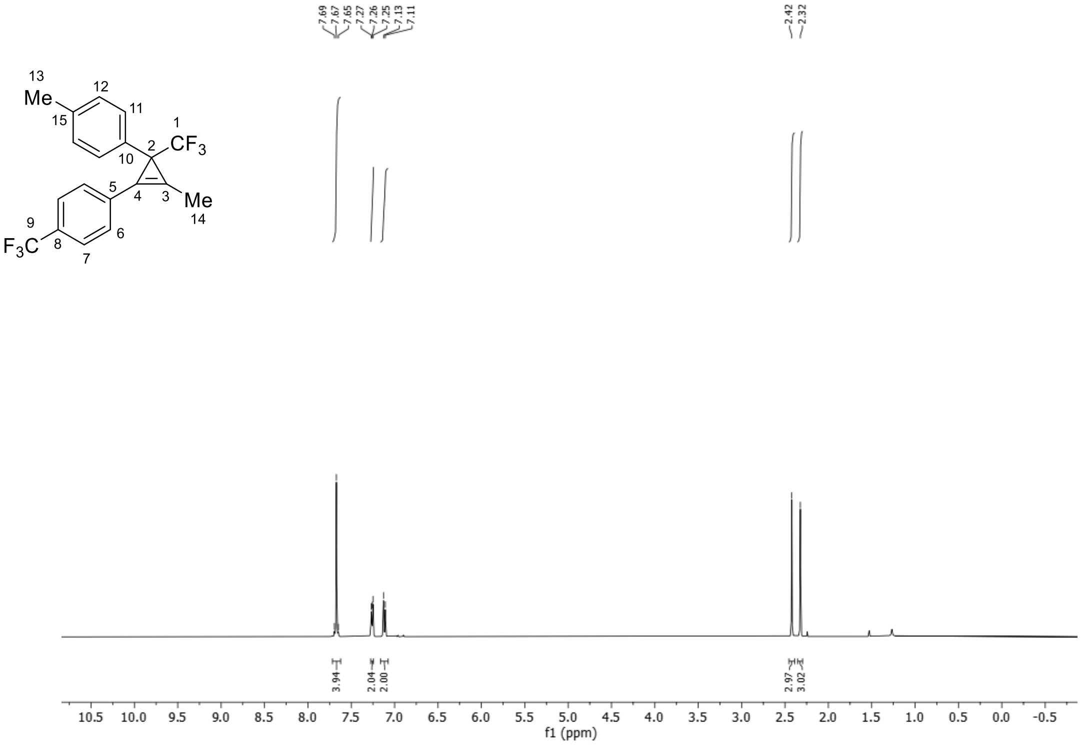

Figure S251. ^{1}H NMR of **S38** (400 MHz, 299 K, $CDCl_3$).

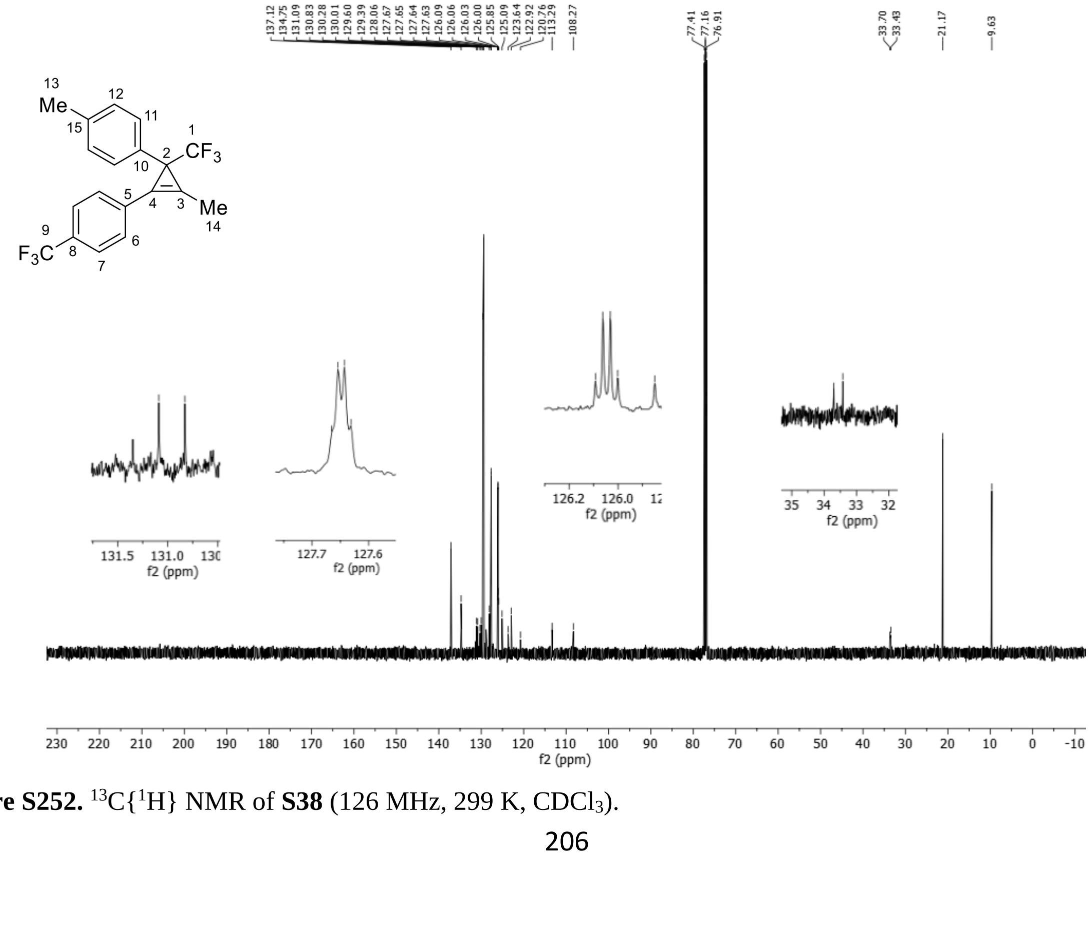

Figure S252. $^{13}C\{^{1}H\}$ NMR of **S38** (126 MHz, 299 K, $CDCl_3$).

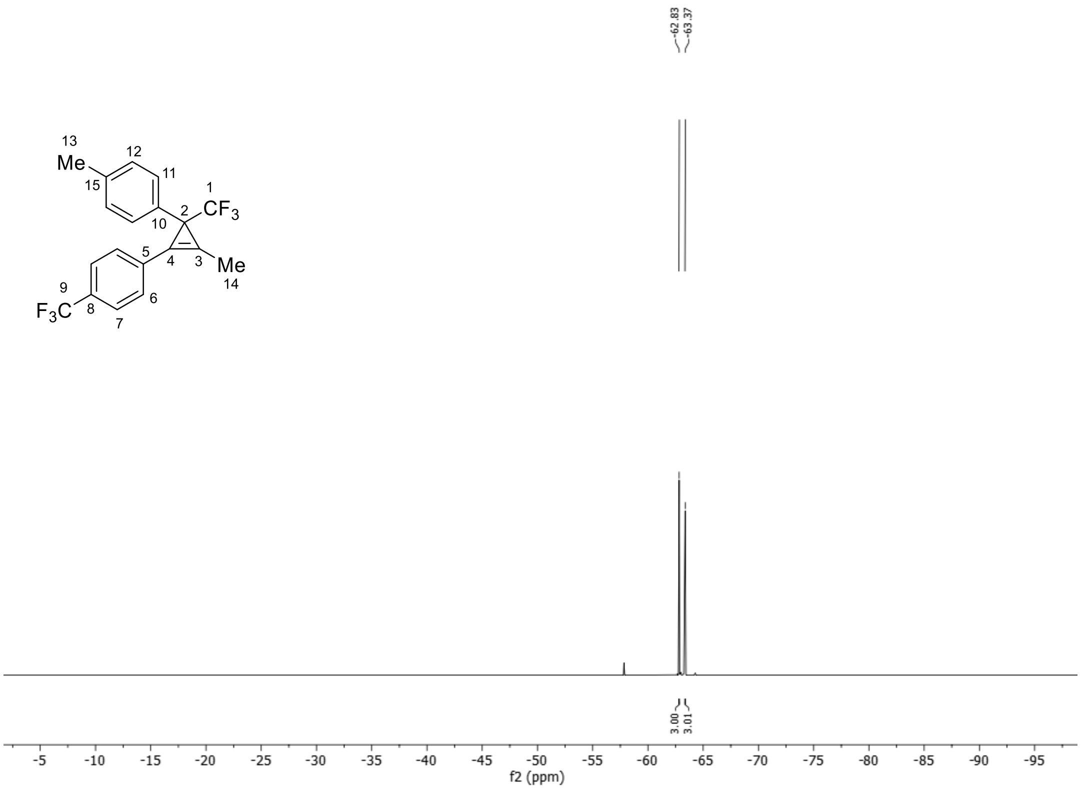

Figure S253. ^{19}F NMR of **S38** (470 MHz, 299 K, $CDCl_3$).

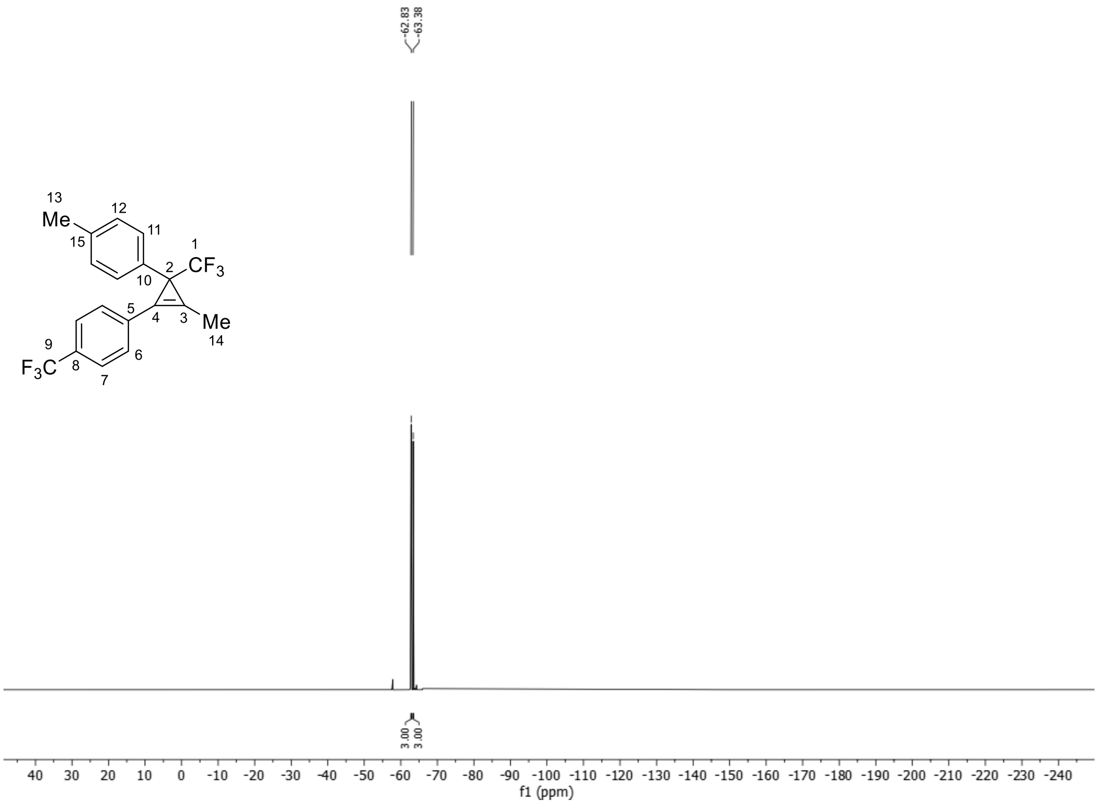

Figure S254. ^{19}F{^{1}H} NMR of **S38** (470 MHz, 299 K, $CDCl_3$).

1-Fluoro-2-hexyl-3-(trifluoromethyl)-1-(4-(trifluoromethyl)phenyl)-1H-indene (36)

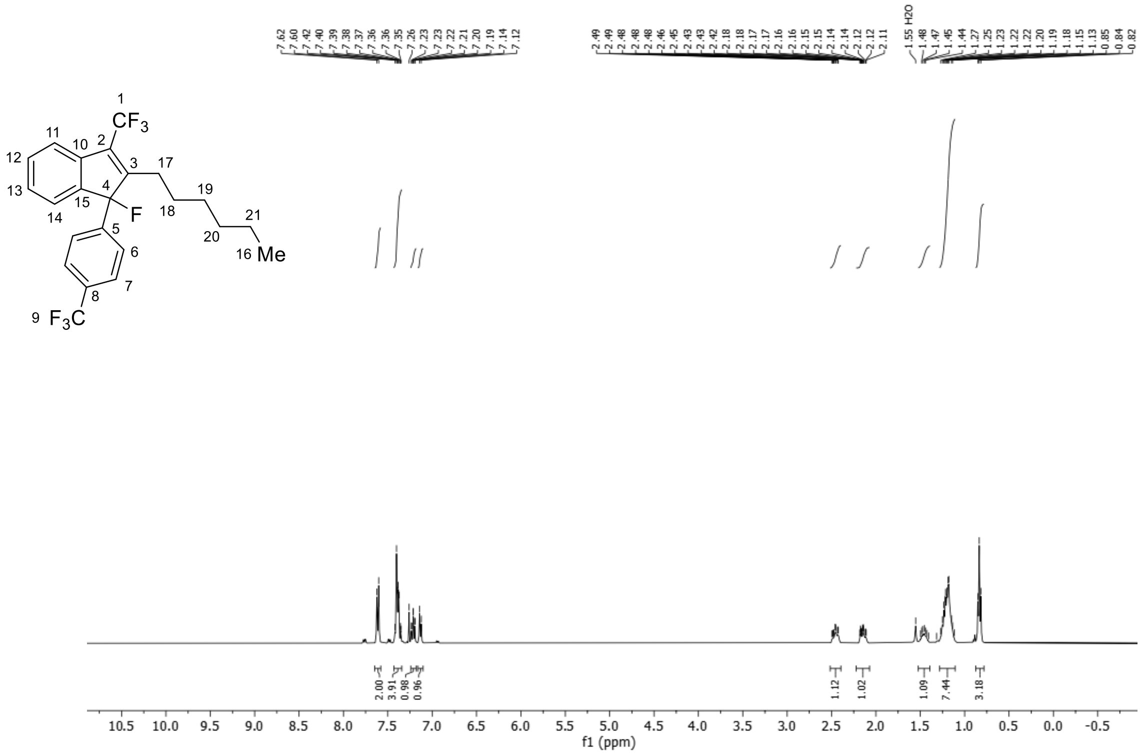

Figure S255. ^{1}H NMR of **36** (400 MHz, 299 K, CDCl$_3$).

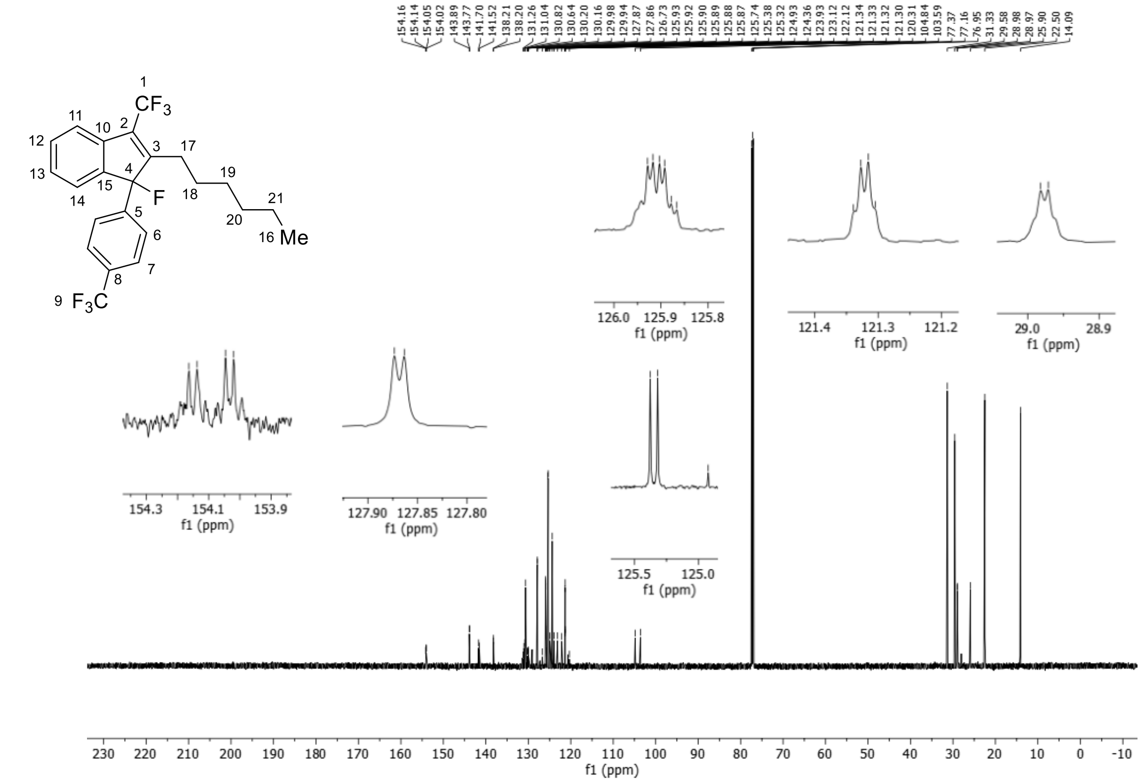

Figure S256. ^{13}C{^{1}H} NMR of **36** (151 MHz, 299 K, CDCl$_3$).

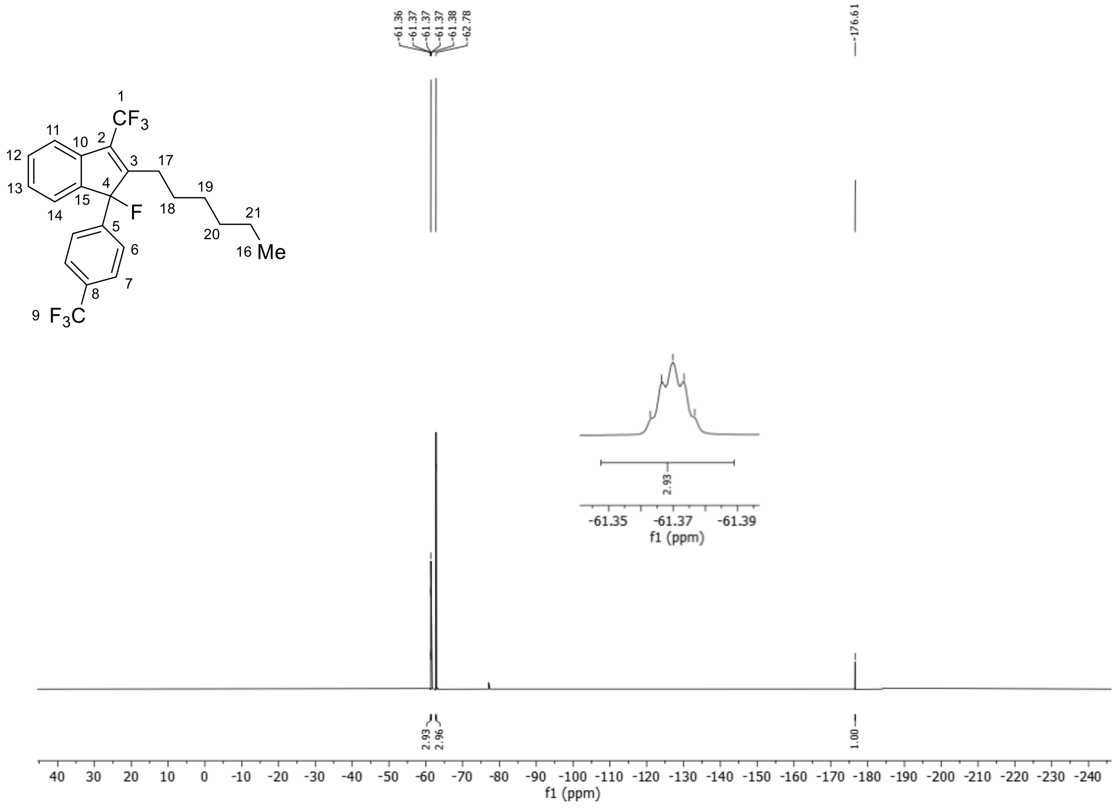

Figure S257. ^{19}F NMR of **36** (564 MHz, 299 K, $CDCl_3$).

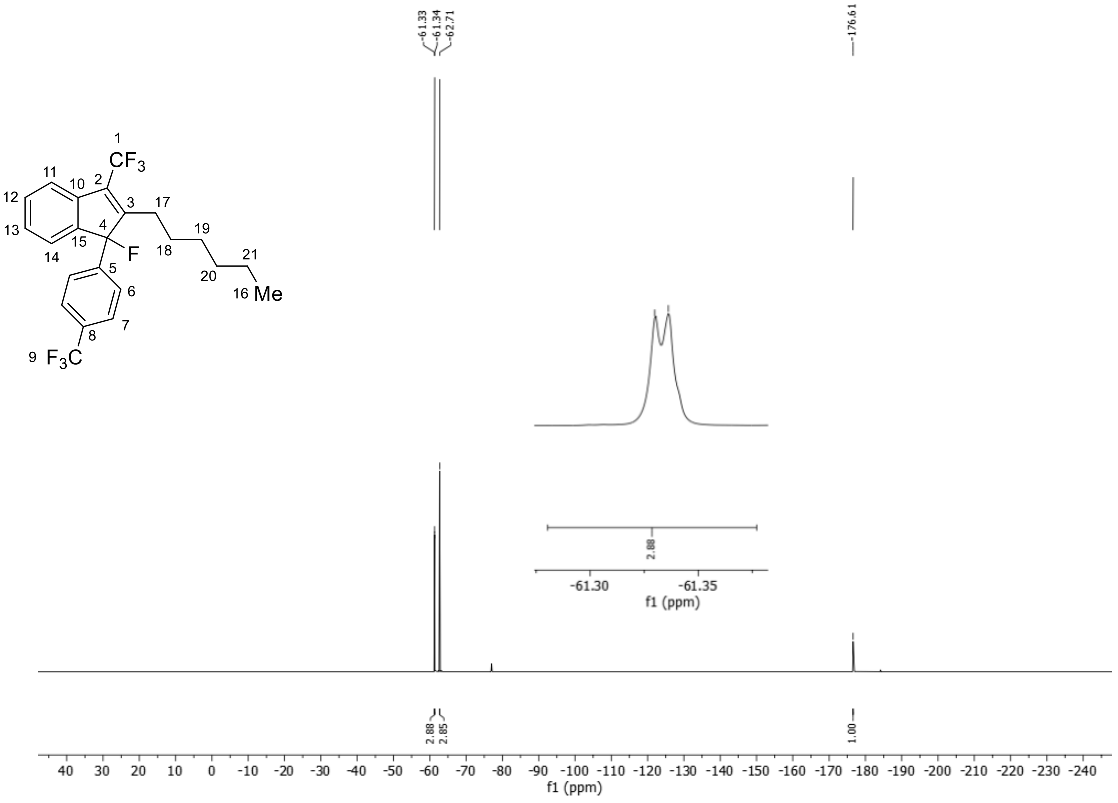

Figure S258. ^{19}F{^{1}H} NMR of **36** (377 MHz, 299 K, $CDCl_3$).

Methyl 4-(1-fluoro-2-hexyl-3-(trifluoromethyl)-1H-inden-1-yl)benzoate (37)

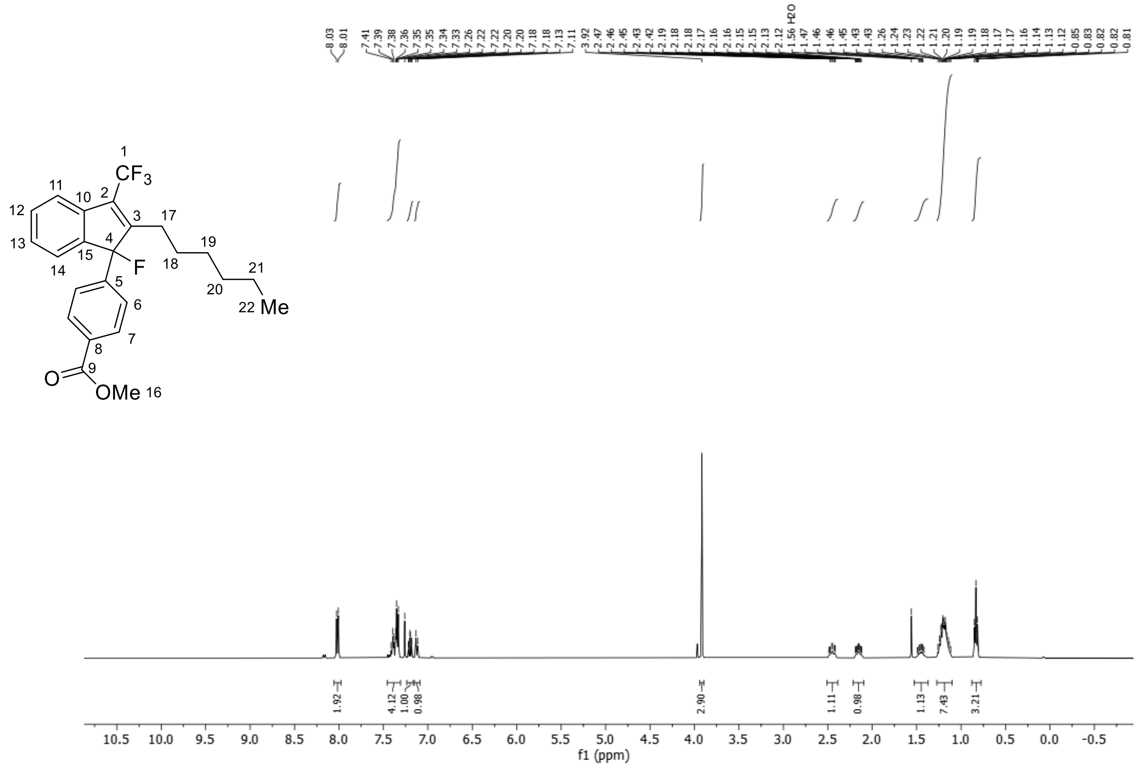

Figure S259. ^{1}H NMR of **37** (400 MHz, 299 K, $CDCl_3$).

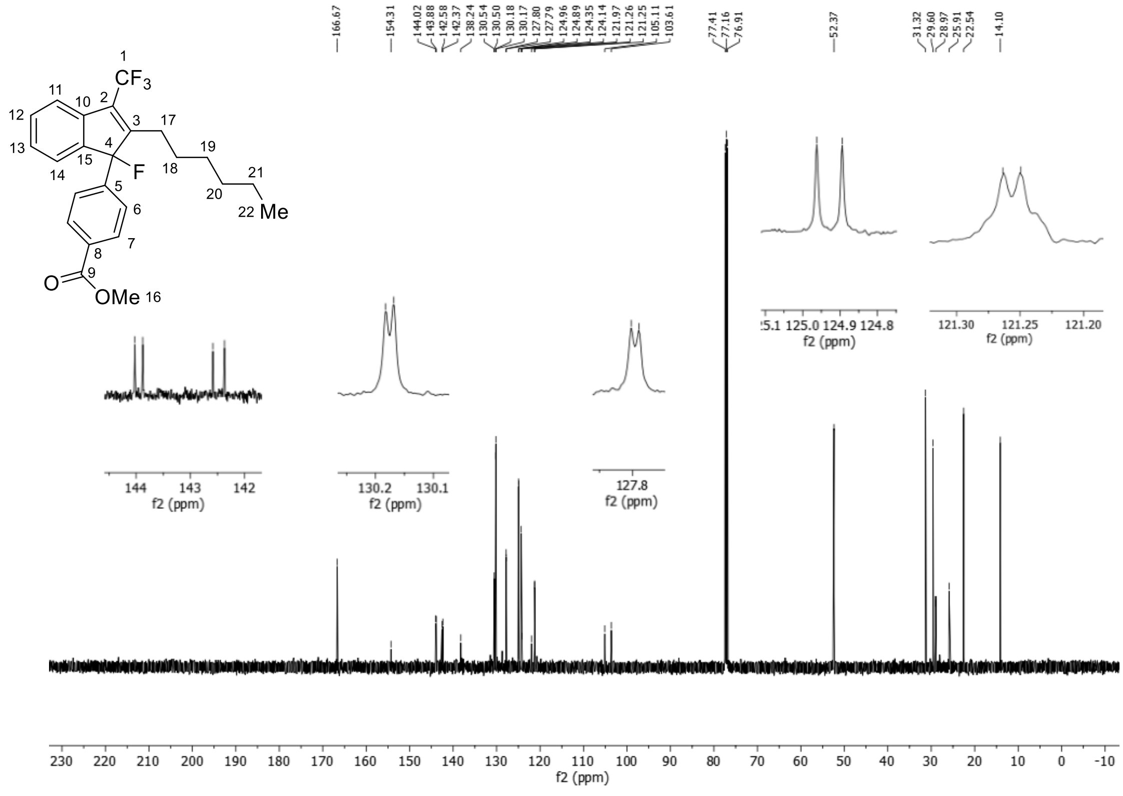

Figure S260. $^{13}C\{^1H\}$ NMR of **37** (126 MHz, 299 K, $CDCl_3$).

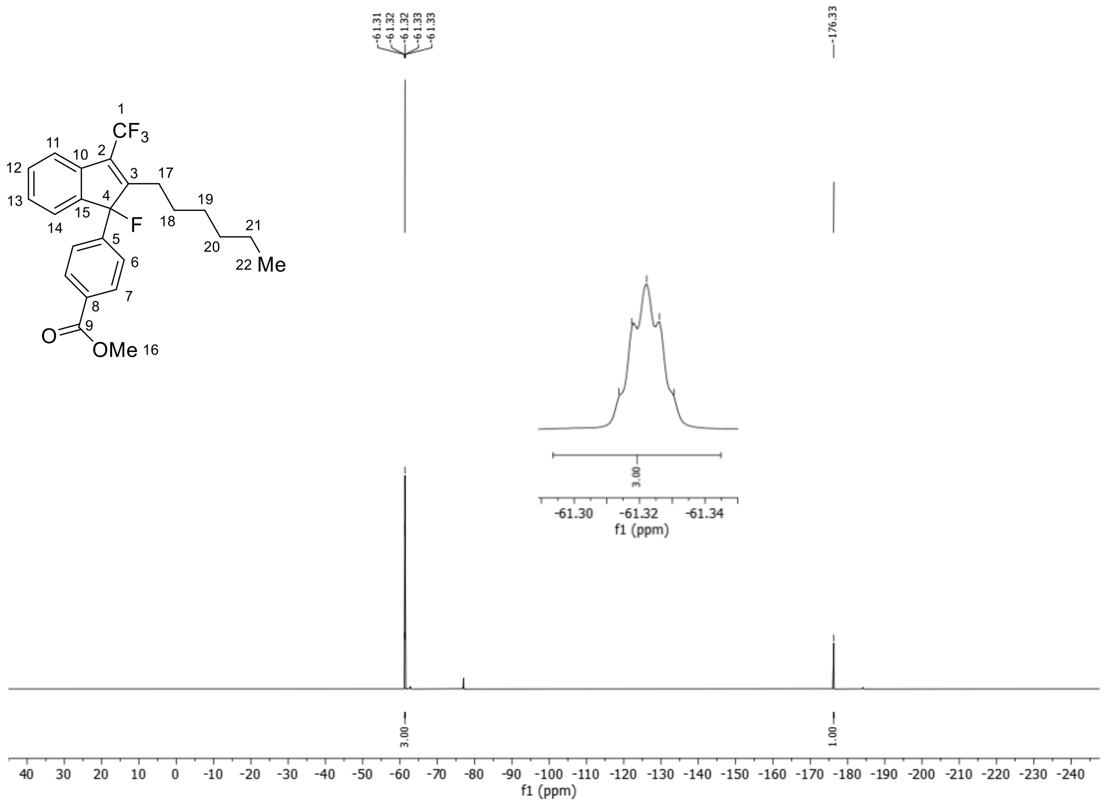

Figure S261. ^{19}F NMR of **37** (470 MHz, 299 K, $CDCl_3$).

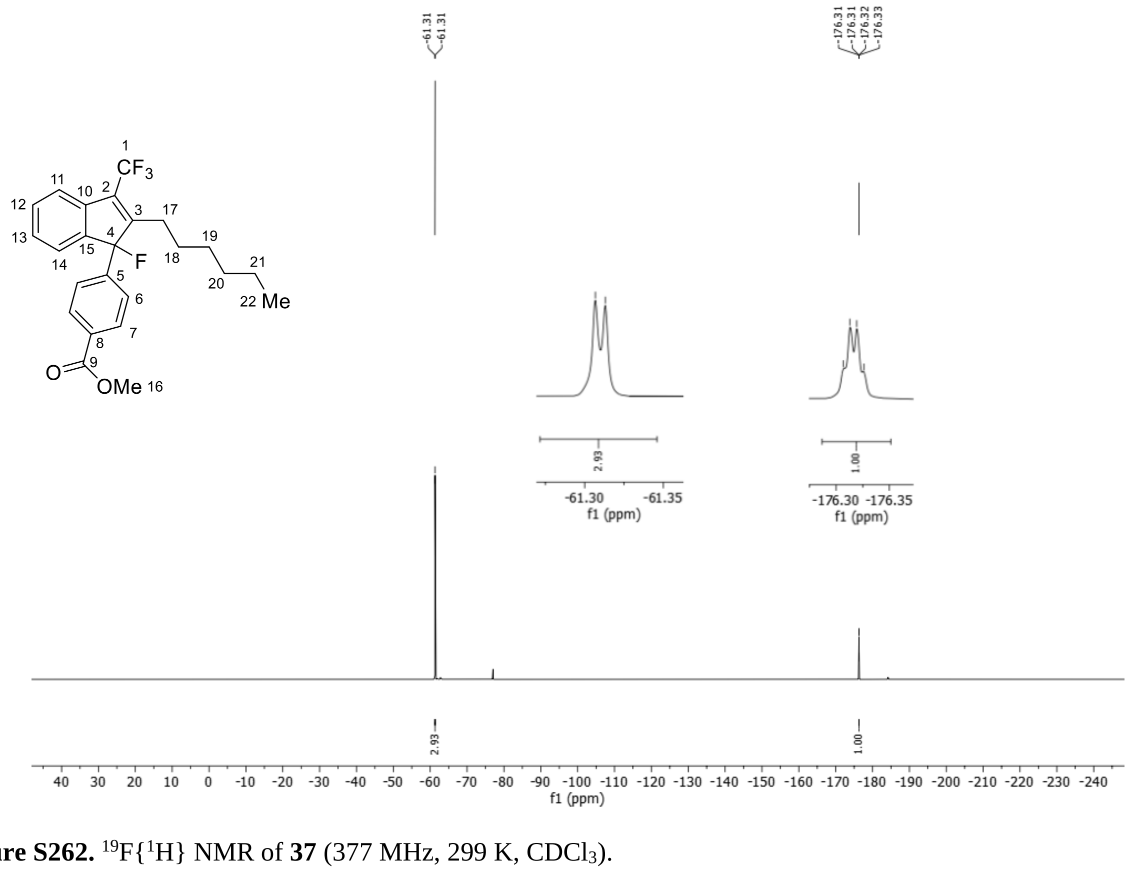

Figure S262. $^{19}F\{^1H\}$ NMR of **37** (377 MHz, 299 K, $CDCl_3$).

1-Fluoro-2,6-dimethyl-3-(trifluoromethyl)-1-(4-(trifluoromethyl)phenyl)-1H-indene (38)

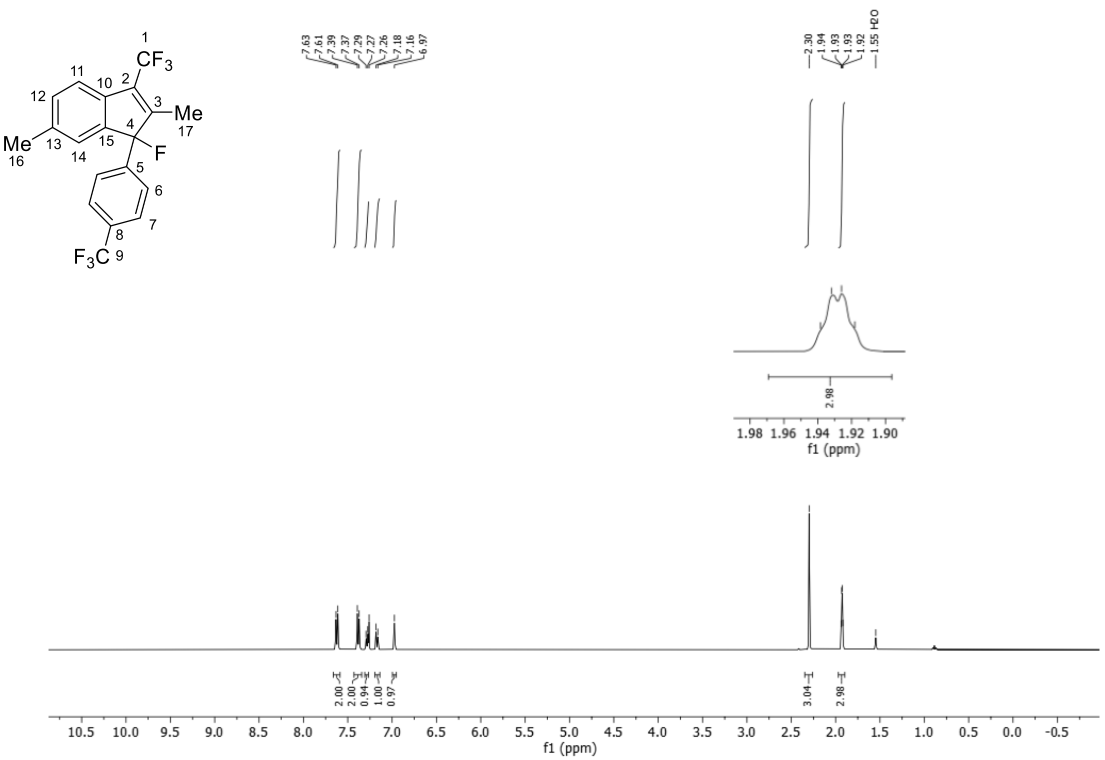

Figure S263. ^{1}H NMR of **38** (400 MHz, 299 K, $CDCl_3$).

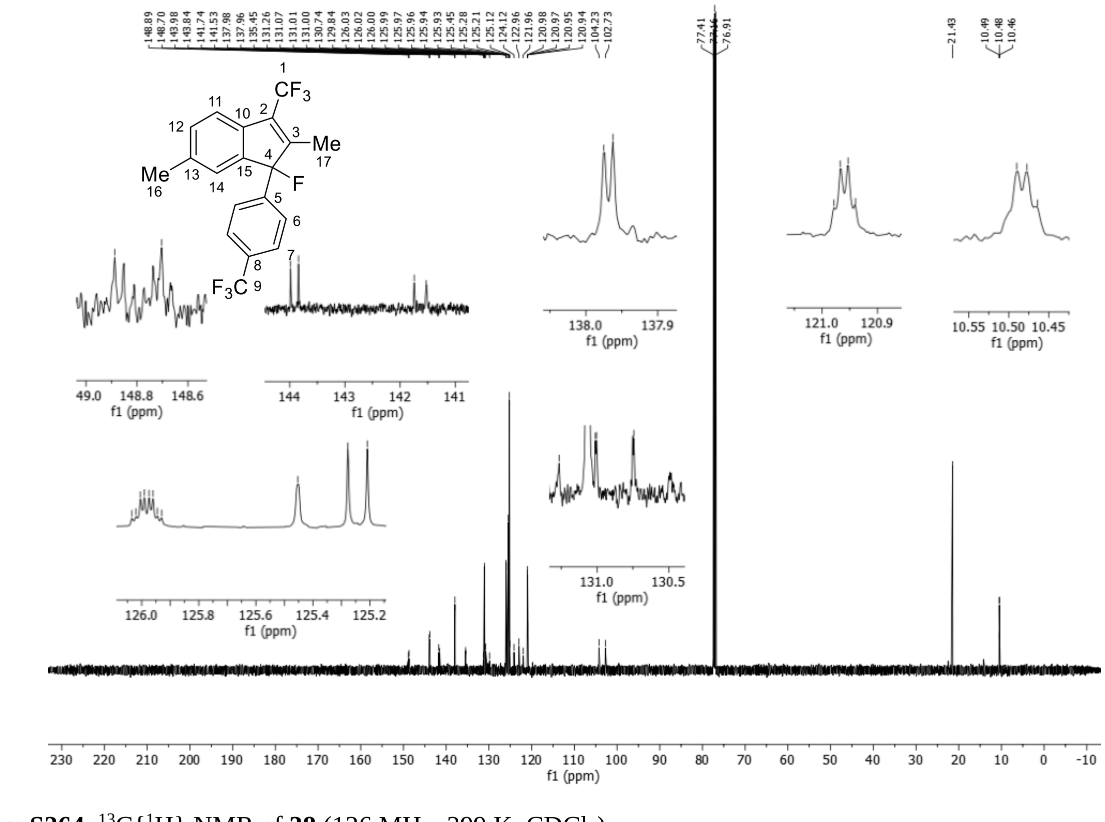

Figure S264. $^{13}C\{^{1}H\}$ NMR of **38** (126 MHz, 299 K, $CDCl_3$).

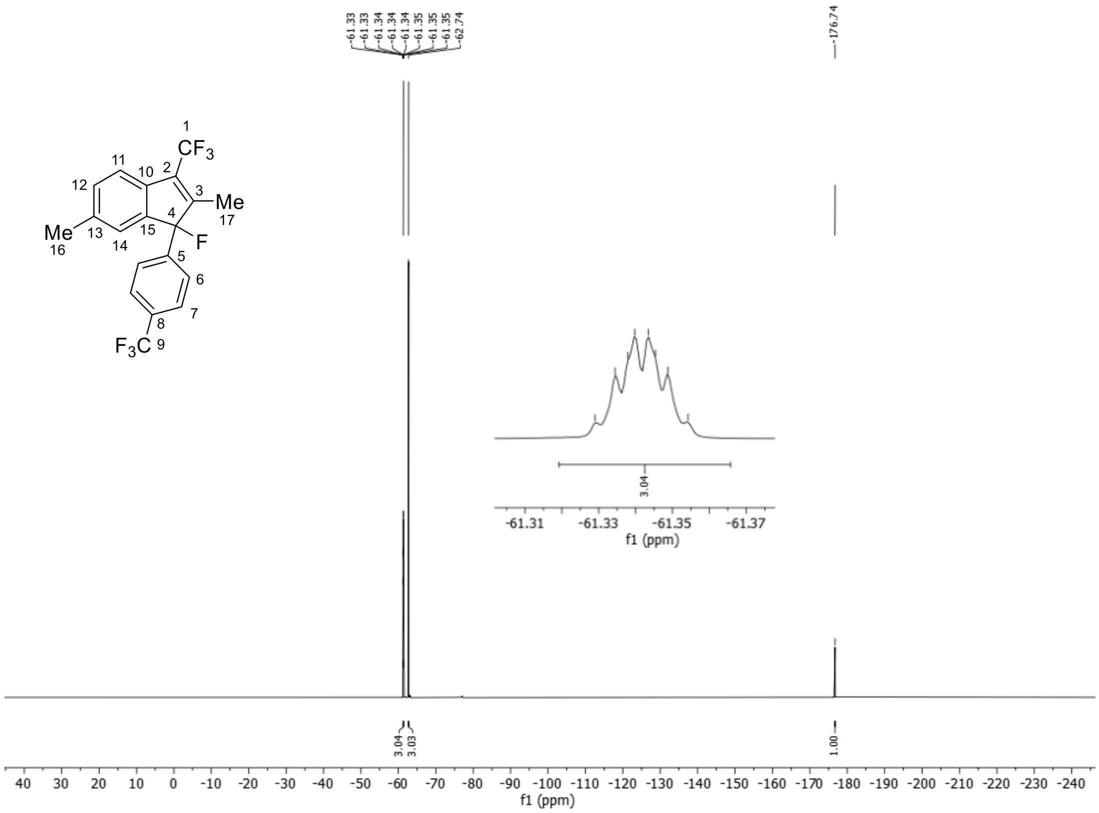

Figure S265. ^{19}F NMR of **38** (470 MHz, 299 K, $CDCl_3$).

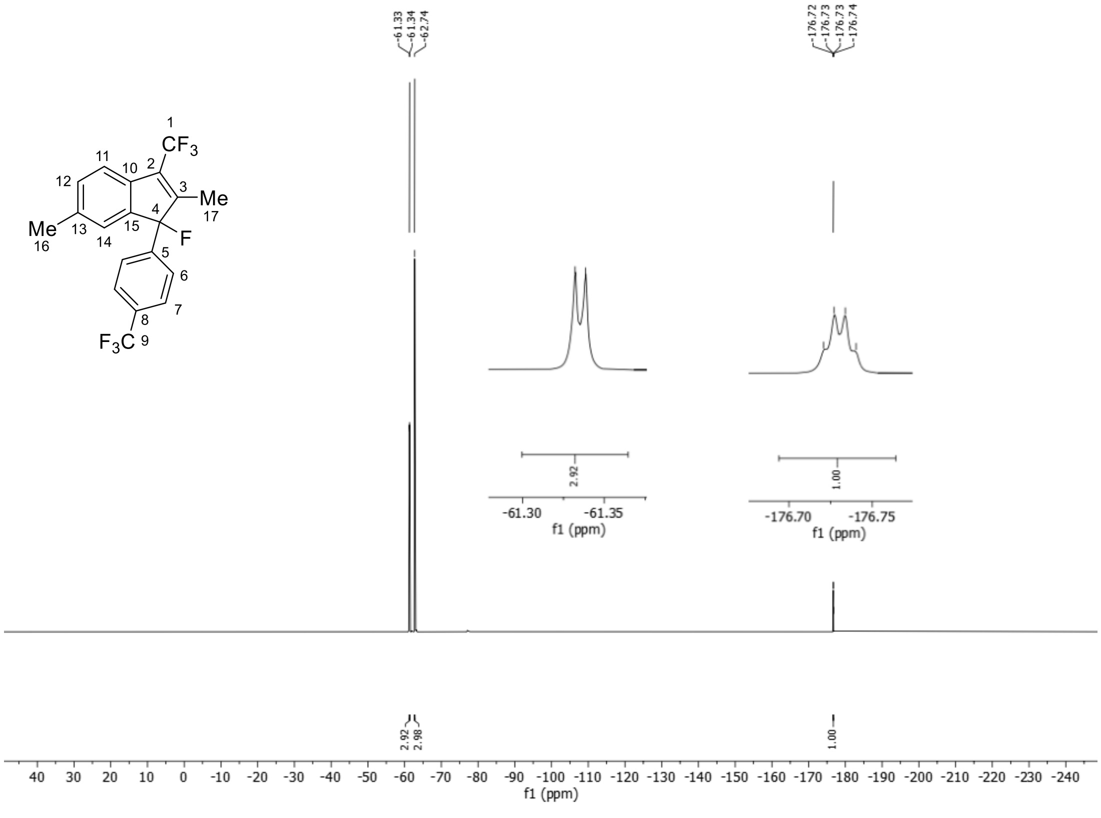

Figure S266. $^{19}F\{^1H\}$ NMR of **38** (377 MHz, 299 K, $CDCl_3$).

2. Supplementary References

1. J. Häfliger, K. Livingstone, C. G. Daniliuc, R. Gilmour, *Chem. Sci.* **2021**, *12*, 6148-6152.
2. B. Muriel, J. Waser, *Angew. Chem. Int. Ed.* **2021**, *60*, 4075-4079.
3. J. F. Briones, H. M. L. Davies, *Org. Lett.* **2011**, *13*, 3984-3987.
4. T. Yasukawa, P. Gilles, J. Martin, J. Boutet, J. Cossy, *ACS Catal.* **2024**, *14*, 6188-6193.
5. E. Emer, J. Twilton, M. Tredwell, S. Calderwood, T. L. Collier, B. Liégault, M. Taillefer, V. Gouverneur, *Org. Lett.* **2014**, *16*, 6004-6007.
6. X. Zhang, C. Tian, Z. Wang, P. Sivaguru, S. P. Nolan, X. Bi, *ACS Catal.* **2021**, *11*, 8527-8537.
7. S. Hyde, J. Veliks, D. M. H. Ascough, R. Szpera, R. S. Paton, V. Gouverneur, *Tetrahedron* **2019**, *75*, 17-25.
8. X. Huang, M. Garcia-Borràs, K. Miao, S. B. J. Kan, A. Zutshi, K. N. Houk, F. H. Arnold, *ACS Central Science* **2019**, *5*, 270-276.
9. V. Myronova, D. Cahard, I. Marek, *Org. Lett.* **2022**, *24*, 9076-9080.
10. O. A. Davis, J. A. Bull, *Angew. Chem. Int. Ed.* **2014**, *53*, 14230-14234.
11. S. Choi, J. Park, E. Yu, J. Sim, C.-M. Park, *Angew. Chem. Int. Ed.* **2020**, *59*, 11886-11891.
12. Z. Yu, Y. Pan, Z. Wang, J. Wang, Q. Lin, *Angew. Chem. Int. Ed.* **2012**, *51*, 10600-10604.
13. V. Tarwade, X. Liu, N. Yan, J. M. Fox, *J. Am. Chem. Soc.* **2009**, *131*, 5382-5383.
14. F. Ye, C. Wang, Y. Zhang, J. Wang, *Angew. Chem. Int. Ed.* **2014**, *53*, 11625-11628.
15. P. Müller, N. Pautex, M. P. Doyle, V. Bagheri, *Helv. Chim. Acta* **1990**, *73*, 1233-1241.
16. P. Yu, A. Bismuto, B. Morandi, *Angew. Chem. Int. Ed.* **2020**, *59*, 2904-2910.
17. L. Pitzer, F. Sandfort, F. Strieth-Kalthoff, F. Glorius, *J. Am. Chem. Soc.* **2017**, *139*, 13652-13655.
18. N. Jeedimalla, C. Jacquet, D. Bahneva, J.-J. Youte Tendoung, S. P. Roche, *J. Org. Chem.* **2018**, *83*, 12357-12373.
19. J. Häfliger, O. O. Sokolova, M. Lenz, C. G. Daniliuc, R. Gilmour, *Angew. Chem. Int. Ed.* **2022**, *61*, e202205277.
20. M. Liu, M. Ye, Y. Xue, G. Yin, D. Wang, J. Huang, *Tetrahedron Lett.* **2016**, *57*, 3137-3139.
21. F. Monnier, F. Turtaut, L. Duroure, M. Taillefer, *Org. Lett.* **2008**, *10*, 3203-3206.
22. Bruker AXS (**2024**) *APEX6 Version 2024.9-0*, *SAINT Version 8.41* and *SADABS Bruker AXS area detector scaling and absorption correction Version 2016/2*, Bruker AXS Inc., Madison, Wisconsin, USA.
23. G. M. Sheldrick, *Acta Cryst* **2015**, *A71*, 3-8.
24. G. M. Sheldrick, *Acta Cryst* **2015**, *C71*, 3-8.
25. Bruker AXS (**1998**) *XP – Interactive molecular graphics*, *Version 5.1*, Bruker AXS Inc., Madison, Wisconsin, USA.
